# The Catalytic Enantioselective Sommelet–Hauser Rearrangement

**DOI:** 10.1021/jacs.6c04476

**Published:** 2026-08-06

**Authors:** Ihssane El Fdali, Jacqueline M. Laddusaw, Kevin Kasten, Aidan P. McKay, David B. Cordes, Paul Ha-Yeon Cheong, Andrew D. Smith

**Affiliations:** † EaStCHEM, School of Chemistry, University of St Andrews, North Haugh, St Andrews KY16 9ST, United Kingdom; ‡ Department of Chemistry, 2694 Oregon State University, 153 Gilbert Hall, Corvallis, Oregon 97331, United States

## Abstract

The Sommelet–Hauser rearrangement of ammonium ylides incorporates a dearomatizing [2,3]-sigmatropic rearrangement of a (hetero)­aromatic substituent, followed by tautomerization (rearomatization), to generate (hetero)­benzylic amine derivatives. Despite widespread interest in this process, asymmetric variants have previously relied upon stoichiometric chiral auxiliary or chirality transfer strategies to induce stereocontrol. In this manuscript, the organocatalytic enantioselective Sommelet–Hauser rearrangement of ammonium ylides bearing heteroaromatic substituents is demonstrated using a Lewis basic isothiourea catalyst, providing a series of unnatural α-heteroaryl α-amino acid derivatives with high to excellent enantioselectivity (up to 98:2 er). The developed process tolerates cyclic and acyclic ammonium salt precursors with indolyl and benzofuranyl substituents. A range of pronucleophiles was employed that lead to amide, ester, and alcohol products. Computational analysis revealed that a stepwise process (involving initial C–N bond cleavage to generate an ion pair, followed by a barrierless C–C bond formation) is favored over a concerted [2,3]-rearrangement, with distortion-interaction analysis providing insight into the factors that govern the mechanism. Crossover experiments, including the use of isotopologue salt derivatives to minimize chemical differentiation of substrates, showed no scrambling. The totality of accumulated computational and experimental evidence suggests a stepwise rearrangement involving a near-instantaneous second recombination step such that the overall process is indistinguishable from, and effectively, a concerted asynchronous rearrangement.

## Graphical abstract

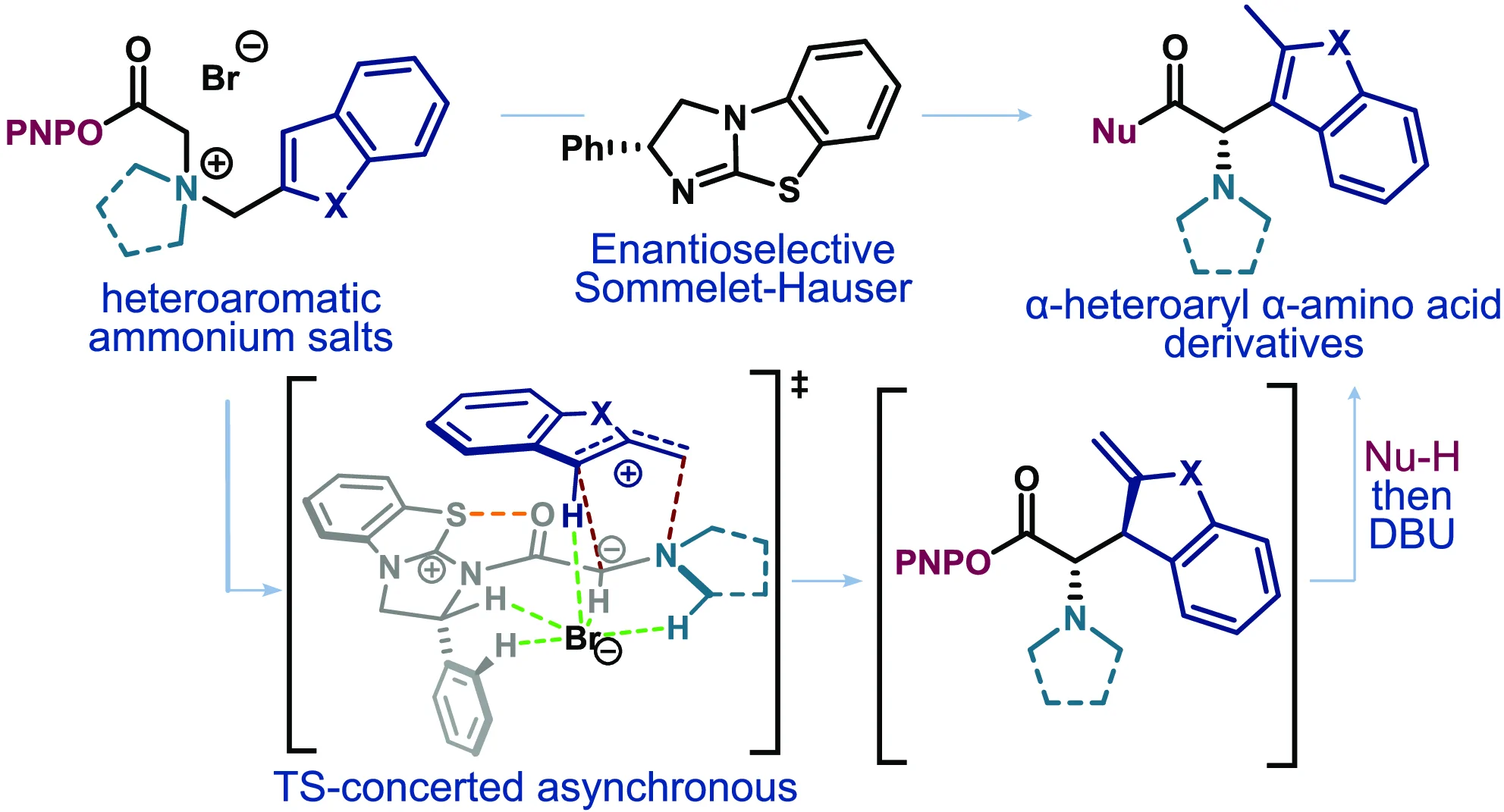

## Introduction

[2,3]-Sigmatropic rearrangements are pivotal in organic synthesis, serving as key methods for the selective construction of carbon–carbon and carbon–heteroatom bonds.^1^ Their high reliability and atom economy make them desirable for building complex molecules.^2–6^ Over the last 40 years, there have been tremendous developments in the field of onium ylide rearrangements, especially through the metal-catalyzed generation of ylide intermediates from diazo compounds.^7–12^ Numerous examples of rearrangements involving sulfonium,^13–16^ oxonium,^17–20^ ammonium,^21–28^ and halonium^29–31^ ylides have been reported, with application to allylic, propargylic, and benzylic systems commonly employed.^9,23^ In these onium ylide systems, two potentially competitive processes (specifically [2,3]- and [1,2]-sigmatropic rearrangements) are widely recognized. Generally, [2,3]-rearrangement processes are favored in allylic and propargylic systems, with selectivity for [1,2]-processes in allylic systems typically enhanced with disubstitution of the allylic terminus and higher reaction temperatures.^32–34^

The first [2,3]-sigmatropic rearrangement of benzylic ammonium ylides was discovered by Sommelet in 1937 when trimethylbenzhydrylammonium hydroxide was stored in a desiccator over P_2_O_5_.^35^ A general mechanism for this process was subsequently described by Hauser and co-workers.^35,36^ In this process, treatment of an ammonium salt such as **1** with a base (traditionally sodium amide in liquid ammonia) leads to *in situ* generation of the respective ylide **2**. Subsequent [2,3]-rearrangement upon the benzylic aromatic substituent is rate-limiting, reflecting the energetic penalty of dearomatization. Tautomerization and rearomatization give product **4** in what is now commonly termed the Sommelet–Hauser rearrangement (Figure 1A).^35^ This process has since been broadened to incorporate heteroaromatic substrates (specifically bearing thiophenyl, pyridine, indole and pyrrole substituents) that has extended the functionality compatible with this rearrangement process.^37,38^ When considering the base-promoted rearrangement of carbonyl-stabilized benzylic ammonium ylides, Sato and co-workers have shown that competitive [1,2]-Stevens and [2,3]-Sommelet–Hauser rearrangements can be achieved through selective manipulation of the electronic nature of the benzylic substituents.^39–42^ Despite this extensive knowledge and widespread interest, only limited asymmetric versions of the Sommelet–Hauser rearrangement have been reported to date.^43^ In this area, Tayama and co-workers have elegantly described a number of stoichiometric approaches to deliver asymmetry in Sommelet–Hauser rearrangement processes (Figure 1B).^44–46^ For example, using an N-to-C chirality transfer strategy,^46–53^ treatment of proline-derived ammonium salt **5** with *t*BuOK in THF led to selective Sommelet–Hauser product **6** in excellent yield and >99:1 er via [2,3]-rearrangement of the derived ylide, while treatment with CsOH in dichloroethane led to the corresponding [1,2]-rearrangement product in 45% yield and >99:1 er.^46^ Alternatively, a chelation-controlled approach with a stereodirecting 2-oxyalkyl substituent was utilized to deliver amine **8** in 43% yield and 95:5 er upon treatment of ammonium salt **7** with *t*BuOK in THF.^54^ Further work from Tayama demonstrated an effective chiral auxiliary approach that used (−)-8-phenylmenthol to dictate stereocontrol within this rearrangement process, although incorporation of an electron-withdrawing substituent (R = CN, CF_3_) was required for optimal reactivity. For example, treatment of ammonium salt **9** with *t*BuOK at −40 °C led to rearrangement and rearomatization, giving **10** in 90% yield and 98:2 dr.^53,55^

**Figure 1:**
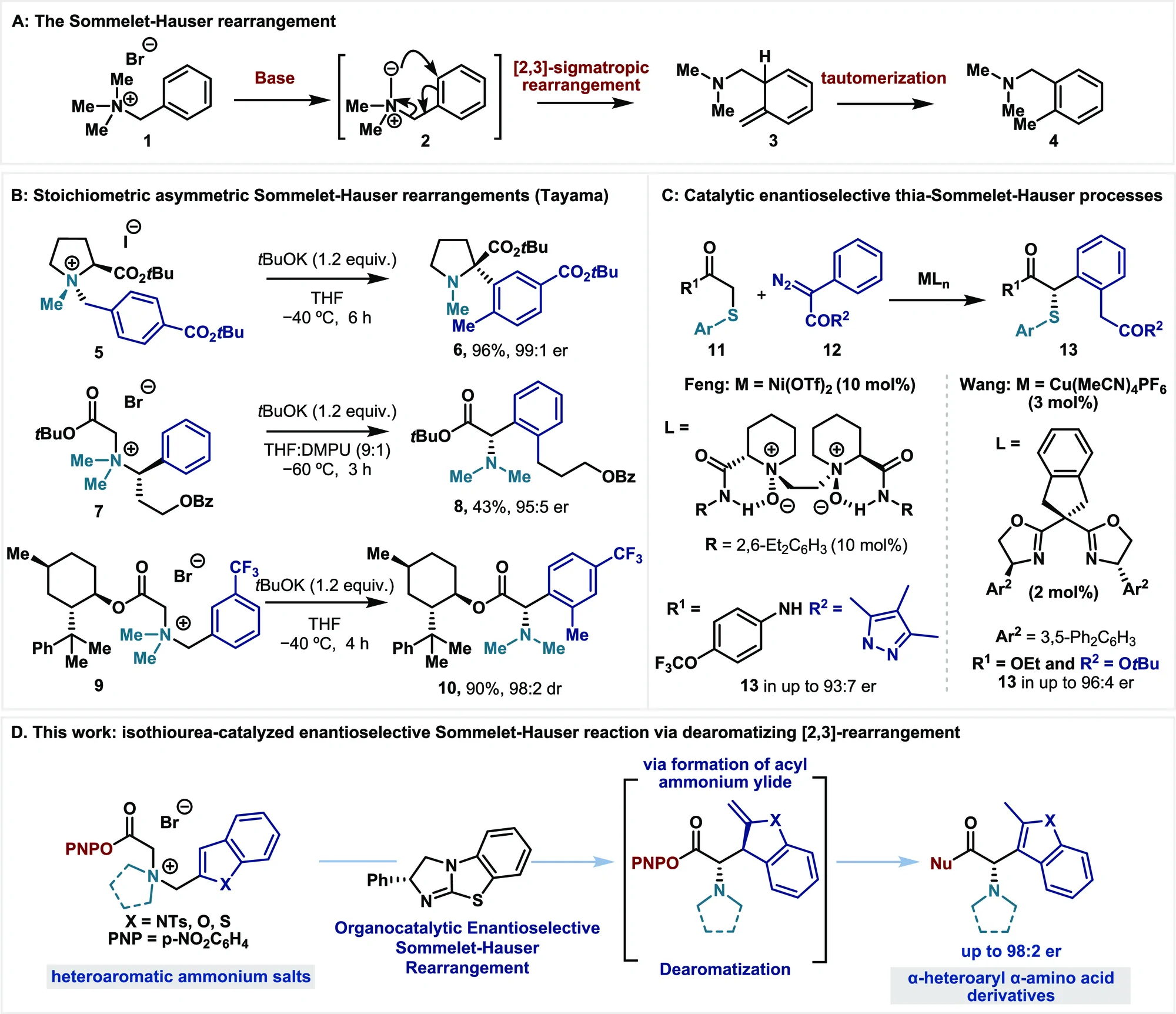
(A). The Sommelet–Hauser rearrangement. (B). Stoichiometric asymmetric Sommelet–Hauser rearrangements (Tayama). (C). Catalytic enantioselective thia-Sommelet–Hauser processes (Feng and Wang). (D). This work: isothiourea-catalyzed enantioselective Sommelet–Hauser reaction via dearomatizing [2,3]-rearrangement.

There are currently no catalytic enantioselective approaches to the Sommelet–Hauser rearrangement, although catalytic enantioselective thia-Sommelet–Hauser approaches have been developed by the groups of Feng and Wang (Figure 1C). The approach from Feng and co-workers^56^ described the rearrangement of sulfonium ylides bearing a pyrazole moiety and using a chiral Ni­(II) catalyst, affording dicarbonyl compounds **13** in up to 88% yield and 93:7 er. In 2020, Wang and co-workers subsequently reported an enantioselective thia-Sommelet–Hauser rearrangement mediated by a Cu­(I)-chiral bisoxazoline catalyst that gave access to chiral sulfur-containing compounds **13** with high enantiocontrol (up to 89% yield and 96:4 er).^57^

In this context, the development of a catalytic enantioselective Sommelet–Hauser rearrangement of ammonium ylides represents a significant and recognized synthetic challenge. Since the first application of isothioureas as organocatalysts by Birman,^58^ these versatile species have been applied to a range of synthetic transformations^59–68^ and extended to the development of the corresponding isoseleno- and isotelluroureas.^69,70^ In previous work, we demonstrated that isothiourea catalysts can promote the enantioselective [2,3]-rearrangement of allylic ammonium ylides,^21–23,71–73^ and recently showed that disubstitution at the allylic terminus allowed the development of an effective enantioselective [1,2]-rearrangement reaction process.^34^ Building upon this experience, this manuscript describes a catalytic enantioselective dearomatizing [2,3]-rearrangement of ammonium ylides bearing a heteroaromatic substituent to facilitate the development of a catalytic enantioselective Sommelet–Hauser rearrangement (Figure 1D). The products generated are enantioenriched α-heteroaryl α-amino acid derivatives, a class of non-natural amino acid building blocks that is attractive for the preparation of differentiated amine-containing scaffolds. This study also addresses the potential competition between [1,2]-Stevens and [2,3]-Sommelet–Hauser pathways,^34,74–79^ and shows that this is predominantly decided by substrate substitution in this catalytic system.

## Results and Discussion

### Initial Optimization and Development of a One-Pot Enantioselective Sommelet–Hauser Rearrangement

The proposed enantioselective Sommelet–Hauser process was initially studied using ammonium salt **14** bearing an *N*-tosyl-protected indole heterocycle. This was informed by the principle that the energetic penalty of dearomatization within heteroaromatic systems is significantly reduced compared to that of benzenoid aromatic substituents.^80–84^ CH_3_CN was chosen as the reaction solvent due to its high polarity, which facilitates solubility of the substrate and isothiourea catalysts, combined with its use in our previous [2,3]- and [1,2]-rearrangements of ammonium ylides.^21–23,65–67^ In proof-of-principle studies, treating salt **14** with (*R*)-benzotetramisole (BTM)^85^ and diisopropylamine in CH_3_CN for 24 h at rt, followed by the addition of benzylamine (due to the expected instability of the *p*-nitrophenyl ester product) afforded a 75:25 mixture of amide **15** (from [2,3]-rearrangement) and the desired compound **16** (arising from the [2,3]-rearrangement, followed by tautomerization) in 88:12 er (Table 1, entry **1**). Notably, the product of a [1,2]-rearrangement pathway was not observed during these studies. Further optimization screened a range of chiral isothiourea catalysts at this temperature to optimize the yield and enantioselectivity of **16**. Substituting (*R*)-BTM with structurally related catalysts generally resulted in diminished enantioinduction. For example, (*S*)-tetramisole gave good chemoselectivity for **16** and a higher yield (56%) but with lower enantioselectivity (17:83 er, entry **2**), while (4b*R*,11a*S*)-fused BTM (entry **3**) and (*S*)-*i*Pr-BTM (entry **4**) afforded similar product yields (53% and 52%, respectively) and enantiocontrol (83:17 and 13:87 er). Alternative catalysts such as (2*S*,3*R*)-HyperBTM and (2*S*,3*R*)-HyperSe (entries **5** and **6**) gave reduced yields (32% and 51%, respectively) and enantioselectivity (57:43 and 78:22 er, respectively) for **16**. As (*R*)-BTM gave the highest product er, the role of the external amine base was investigated next. Among those tested, diisopropylamine and diisopropylethylamine gave a good balance of conversion and enantioselectivity (entries **1** and **7**), giving product **16** in 87:13 and 88:12 er, respectively, while pyridine gave lower conversion to the desired product (3%, entry **8**). The use of Cs_2_CO_3_ resulted in good overall yield (52%) and good enantioselectivity (90:10, entry **9**), alongside a higher ratio of the desired product **16** compared with the dearomatized intermediate **15**. Notably, product **15** was always obtained as a single diastereomer (>95:5 dr). The relative and absolute configurations within **15** were assigned on the basis of computational analysis (Figure 3), with unambiguous determination of its enantiopurity not feasible due to its instability and propensity to rearomatization. Interestingly, the use of DBU (entry **10**) led to complete conversion to the rearomatized product **16**, but in essentially racemic form. While unsuitable for the enantioselective rearrangement step, this observation offered significant mechanistic insight, showing that DBU can be used to effectively drive the rearomatization step to **16**.^86^

Variation in reaction temperature (entries **11–14**) showed that optimal enantiocontrol (95:5 er) for **16** was observed at −20 °C in MeCN, with the rearrangement product **15** obtained in high diastereoselectivity (>95:5 dr). The observation of significantly improved enantiocontrol upon lowering the reaction temperature from 0 °C to −20 °C (compared to the change from rt to 0 °C) is ascribed to reduced base-promoted competitive racemic background reactivity at lower temperatures. However, the dearomatized intermediate is relatively prone to tautomerization to give **16** on purification on silica, leading to decreased reproducibility and challenging isolation. Taken together, these observations allowed the development of a streamlined one-pot, three-step protocol focused on selectively delivering the rearomatized product **16**. The [2,3]-rearrangement was first carried out using the isothiourea (*R*)-BTM and Cs_2_CO_3_, then benzylamine was introduced to generate the corresponding amide, before the addition of DBU to promote rearomatization. This telescoped three-step sequence allowed full conversion to the desired product **16** in 53% isolated yield and 98:2 er (entry **15**). Reducing the catalyst loading led to product with reduced enantiocontrol (10 mol %, 90:10 er; 5 mol %, 83:17 er, entries **16–17**), with control reactions indicating a competitive base-catalyzed background reaction to be the origin of this observation (see the SI for further information). To rationalize the moderate isolated yield of rearrangement product, the pioneering work of Kitching and co-workers was considered. In their synthesis of chiral ammonium species, it was reported that quaternary *N*-aryl-*N*-allyl or *N*-aryl-*N*-benzyl ammonium halide salts racemize *via* a dealkylative S_N_2-type process to generate the corresponding amine and allyl or benzyl halide, and it was considered that this may represent a starting material decomposition pathway in the developed process.^87^ Although a solution of ammonium salt **14** in CDCl_3_ showed no decomposition over 1 week, further control experiments (see the SI) indicate that addition of base can lead to both ester hydrolysis and partial N-dealkylation, which are tentatively assigned as the origin of the moderate isolated yield for **16**.

### Scope and Limitations of the Enantioselective Sommelet–Hauser Rearrangement

With an optimized protocol identified, the scope and limitations of this process were explored (Figure 2). The absolute configuration within (*S*)-**16·HCl** was conclusively proven by X-ray crystallographic analysis, consistent with the stereochemical outcome identified in our previous [2,3]-rearrangement of cinnamyl ammonium ylides, with all examples assigned by analogy.^22^ Initial studies examined the effect of introducing various substituents within the indole motif, with the incorporation of halogenated (Cl, Br and F) as well as electron-donating (Me, MeO) groups explored. Notably, the inclusion of halogenated substituents gave the corresponding 6-F-, 5-Br-, and 5-Cl-substituted amino amides **17–19** in moderate to good yields (52–63%) and with excellent enantiocontrol (>91:9 er). The incorporation of electron-donating substituents (6-Me, 6-OMe) was well tolerated, giving products **20** and **21** in good yields and enantioselectivity (62%, 91:9 er and 47%, 96:4 er, respectively). Variation of N-substituents to include cyclic variants, such as pyrrolidine, piperidine, and morpholine, is also readily accommodated, affording **22**–**24** with excellent enantioselectivity (>92:8 er). Variation of the nucleophile to include pyrrolidine gave the corresponding amide **25** with excellent stereocontrol (50% yield, 96:4 er) while the use of MeOH gave methyl ester **26** in 56% yield but with reduced enantioselectivity (74:26 er), consistent with partial racemization during rearomatization due to the increased acidity of the ester compared to the amide. Subsequent work focused on varying the heterocyclic component to a benzofuran motif. In this series, various N-substituents were readily accommodated, with the dimethylamino-substituted analogue giving the product **27** in 36% yield with excellent enantioselectivity (96:4 er) that could be readily scaled to 1.0 mmol scale, maintaining yield and enantioselectivity (38%, 95:5 er). Variation of the ammonium salt counterion to an iodide analogue was tested under the developed reaction conditions, giving **27** (41%, 97:3 er) with no significant difference in reactivity or product distribution compared to the bromide salt. Variation in acyclic amino substitution was also well tolerated in this series, with *N*,*N*-diethyl and *N*,*N*-di-*n-*butyl substitution giving products with excellent enantiocontrol (**28**–**29**, >92:8 er). Extension to *N*,*N*-diallyl substitution gave **30** exclusively (94:6 er), and notably, no competitive [2,3]-rearrangement product from reaction at the *N*-allyl substituents was observed.^71^ Unsymmetrical *N*-benzyl-*N*-methylamine **31** was also obtained from the respective racemic quaternary ammonium salt, giving the product in 72% yield and 96:4 er. No competitive Sommelet–Hauser reaction upon the N-benzyl substituent was observed, consistent with preferential [2,3]-rearrangement upon the benzofuran substituent. Various cyclic N-substituents encompassing pyrrolidine, piperidine, azepane, morpholine and *N*-Boc-piperazine motifs are readily accommodated, giving functionalized amino amides (**32**–**37**) in good yields and excellent er (35–80% yield, >92:8 er). Finally, the incorporation of different nucleophiles was explored in this series. Reduction of the intermediate ester obtained from the rearrangement using LiAlH_4_ gave the corresponding alcohol **38** in 55% yield with excellent enantioselectivity (97:3 er), with pyrrolidine affording the corresponding amide **39** in good yield with excellent enantiocontrol (60%, 97:3 er). Morpholine gave **40** with a comparable yield (58%) with good but reduced enantiocontrol (91:9 er). Following the initially developed conditions, transesterification with methanol or sodium ethanethiolate in the presence of DMAP, prior to treatment with DBU, proceeded to give **41** and **42**, respectively, but in racemic form. In these cases, racemization during the DBU-mediated rearomatization step was identified as the major contributing factor to the erosion of enantioselectivity.^88,89^ To minimize this effect, subsequent reactions were performed using 0.5 equiv of DBU and a reduced reaction time of 30 min, which led to improved product enantiocontrol, giving **41** in 67% yield and 89:11 er, and the corresponding thioester **42** in 58% yield and 60:40 er. In addition to benzofuran and indole substituents, benzothiophene was also tolerated in the dearomatizing rearrangement, albeit generating the respective product **43** in lower yield and enantioselectivity (21%, 84:16 er) and so represents a limitation of this approach. Further limitations identified while probing the scope of this process showed that benzyl, naphthyl and quinoline substituted ammonium salts **44–46** were not compatible with the reaction conditions, preferentially giving ester hydrolysis products. Similarly, attempts to employ *N*-methyl indole ammonium salt **47** as a substrate were unsuccessful. Notably, <10% product formation was observed using the constitutional isomer **48** of the indole substrate **14**, while azetidinium ammonium salt **49** gave a complex mixture of products. Further limitations showed that the incorporation of an additional substituent at C(3) within both N-Ts indole and benzofuran substrates **50** and **52** required the use of an alternative isothiourea catalyst, tetramisole, for optimal reactivity, but returned exclusively the corresponding [1,2]-rearrangement products **51** and **53** in moderate yield (49–56%) and in racemic form. The preferential formation of these [1,2]-products is consistent with that observed with disubstitution of the allylic terminus in our previous work.^34^

**Table 1:**
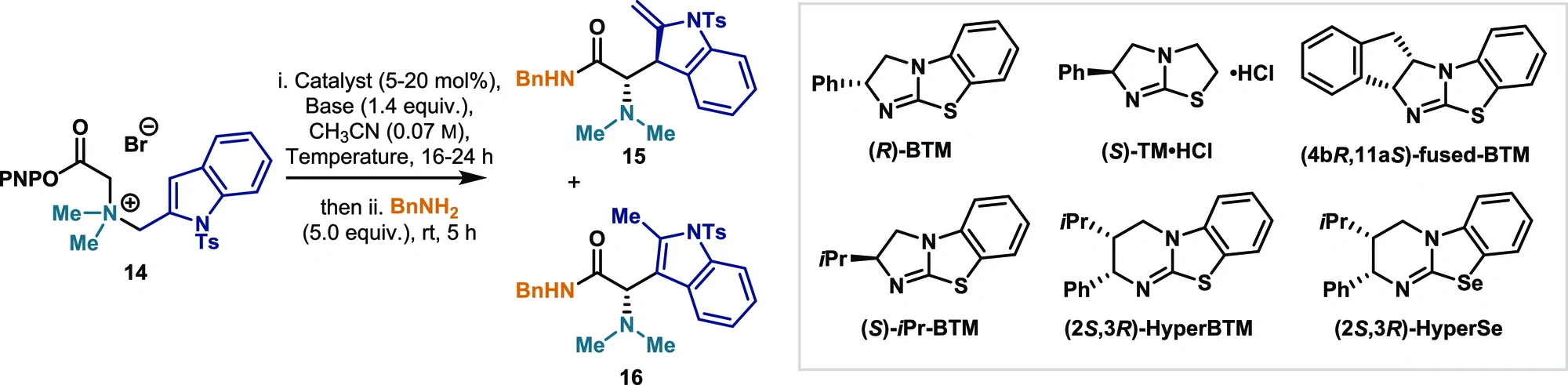
Yields and Rearrangement Product Ratios Determined by ^1^H NMR Analysis of the Crude Reaction Mixture Using 1,3,5-Trimethoxybenzene as Internal Standard^a^

entry	catalyst	base	*T*/°C	yield 15 [%]	yield 16 [%]	er 16
**1**	(*R*)-BTM	(*i*Pr)_2_NH	rt	39	12	88:12
**2**	(*S*)-tetramisole	(*i*Pr)_2_NH	rt	2	56	17:83
**3**	(4b*R*,11a*S*)-FusedBTM	(*i*Pr)_2_NH	rt	8	45	83:17
**4**	(*S*)-*i*Pr-BTM	(*i*Pr)_2_NH	rt	6	46	13:87
**5**	(2*S*,3*R*)-HyperBTM	(*i*Pr)_2_NH	rt	15	17	57:43
**6**	(2*S*,3*R*)-HyperSe	(*i*Pr)_2_NH	rt	22	29	78:22
**7**	(*R*)-BTM	(*i*Pr)_2_NEt	rt	29	16	88:12
**8**	(*R*)-BTM	Pyridine	rt	3	3	72:28
**9**	(*R*)-BTM	Cs_2_CO_3_	rt	23	29	90:10
**10**	(*R*)-BTM	DBU	rt		44	49:51
**11**	(*R*)-BTM	(*i*Pr)_2_NH	0	39	12	88:12
**12**	(*R*)-BTM	(*i*Pr)_2_NH	–20	30	17	95:5
**13**	(*R*)-BTM	(*i*Pr)_2_NH	–30	33	17	94:6
**14**	(*R*)-BTM	(*i*Pr)_2_NH	50	30	14	79:21
**15^b^**	(*R*)-BTM (20 mol %)	Cs_2_CO_3_	–20		53	98:2
**16^b^**	(*R*)-BTM (10 mol %)	Cs_2_CO_3_	–20		46	90:10
**17^b^**	(*R*)-BTM (5 mol %)	Cs_2_CO_3_	–20		42	83:17

a All reactions were carried out on a 0.1 mmol scale and at 0.07 m concentrations. The er was determined by HPLC analysis on a chiral stationary phase.

b Rearrangement was followed by rearomatization upon treatment with DBU (1.0 equiv). See the SI for detailed procedures.

### Computational and Mechanistic Investigations

Computational and mechanistic investigations subsequently aimed to provide an in-depth understanding of this enantioselective catalytic dearomatizing [2,3]-rearrangement. While [2,3]-rearrangements are generally considered to proceed *via* concerted, symmetry-allowed reaction pathways, recent studies have shown that stepwise fragmentation and recombination pathways *via* polar or radical mechanisms should also be considered.^22,34,90^ We therefore performed an in-depth DFT analysis involving the complete computed catalytic cycle probing several mechanistic possibilities, prior to conducting crossover experiments to validate the computational studies. M06-D3/6–31G*/SMD­(acetonitrile) as implemented in Gaussian 16 ^91^ with thermal corrections at −20 °C was used to investigate the reaction mechanism and origins of stereoselectivity with (*R*)-BTM and ammonium salt substrate **54**. Anion complexation to transition structures is recognized to have an important energetic impact. Consequently, bromide counterion coordination *via* stabilizing noncovalent interactions around the transition structure was exhaustively sampled to ensure that the most energetically favorable structures were included in the analyses. The proposed catalytic cycle and computed energetics are shown in Figure 3. Acylation of the (*R*)-BTM catalyst by the ammonium salt generates the corresponding acyl ammonium complex **I**. Deprotonation by *p-*nitrophenoxide generates the acyl ammonium ylide **II**. Subsequent dearomatizing rearrangement of the benzofuran substituent on the side opposite to the stereodirecting catalyst phenyl group leads to either the (2*S*,3*S*)- and (2*S*,3*R*)-configured intermediates **III** and **IV**, respectively. Catalyst turnover proceeds by reaction with *p*-nitrophenoxide to regenerate the isothiourea catalyst and release the corresponding ester products. The (2*S*,3*S*)- and (2*S*,3*R*)-configured ester products subsequently undergo amidation and tautomerization upon treatment with benzylamine and then DBU. This leads to stereoconvergence due to the destruction of the secondary stereocenter upon rearomatization, generating the observed (*S*)-amide product.

**Figure 2:**
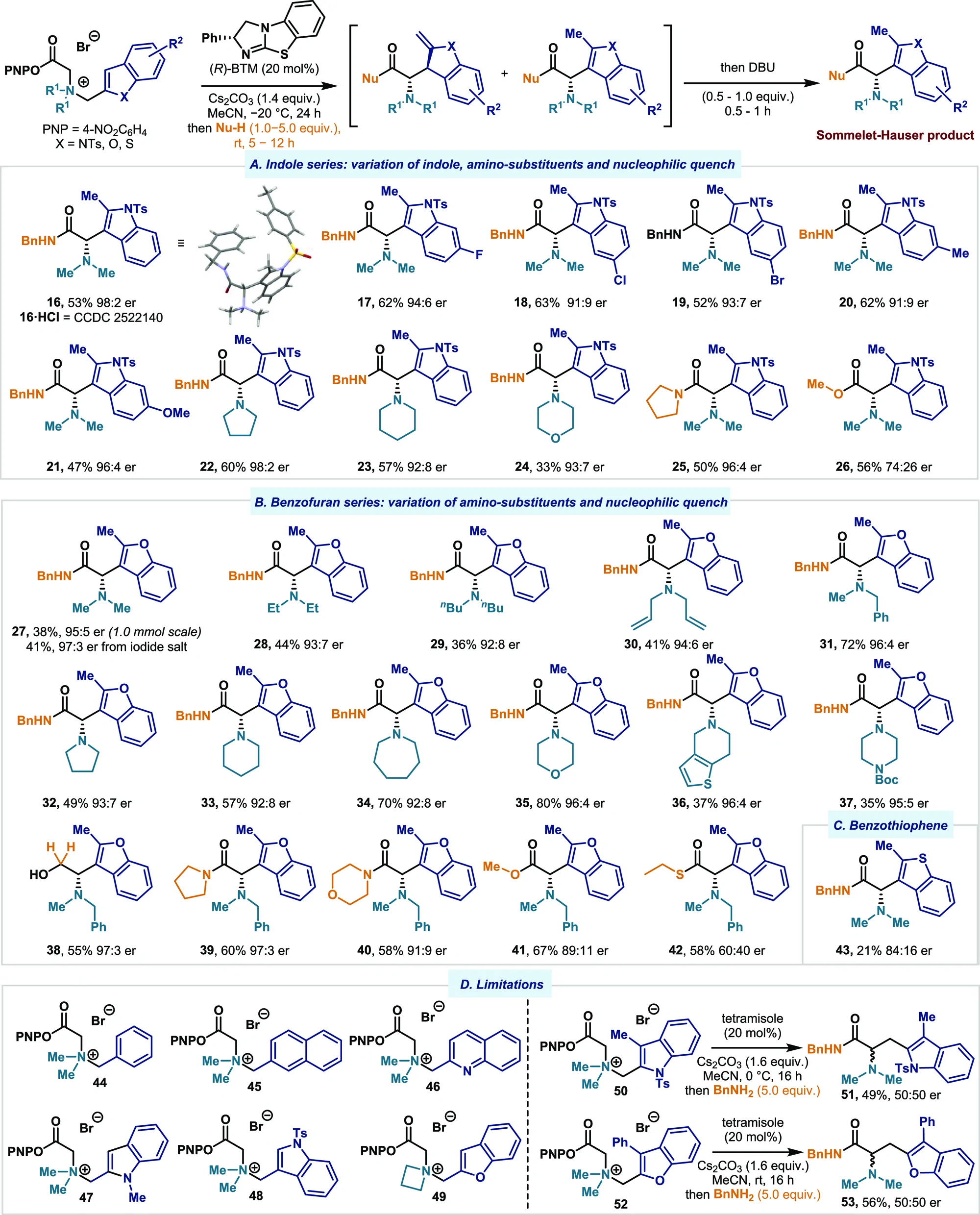
Scope and limitations of the reaction. Isolated yields given. Er determined by HPLC analysis on a chiral stationary phase. (A). Indole series: variation of indole, amino-substituents, and nucleophilic quench. (B). Benzofuran series: variation of amino-substituents and nucleophilic quench. (C). Benzothiophene demonstration. (D). Limitations of the developed process. See the SI for detailed procedures.

Notably, the transition state structures leading to the (2*S*,3*S*)- and (2*S*,3*R*)-configured ester products were shown to proceed *via* stepwise and concerted processes, respectively. The stepwise mechanism to give the (2*S*,3*S*)-stereoisomer was found to be energetically favored by 6.4 kcal/mol. The potential energy surfaces for both processes were next computed to confirm the stepwise and concerted nature of these processes (Figure 3A). In the stepwise process (left), both transition structures were unique stationary points separated by a local minimum. The first transition structure, **C–N Cleavage TS A** (Δ*G* ^‡^ = 7.9 kcal mol^–1^), was the rate-limiting step for this process. Interestingly, the energy of the intermediate that followed (Δ*G* = 5.9 kcal mol^–1^) lay above the following **C–C Forming TS B** (Δ*G* ^‡^ = 5.6 kcal mol^–1^), suggesting a barrierless recombination step. Computational analysis of the potential energy surface for the concerted rearrangement process (right) indicated only a single transition structure (**Concerted [2,3] TS C**, Δ*G* ^‡^ = 14.3 kcal mol^–1^).

To elucidate the origins of these mechanistic differences, a three-component distortion-interaction analysis of the three transition structures was performed (Figure 3B). Each structure was fragmented into its respective neutral catalyst acyl ammonium enolate, benzofuran cation, and bromide anion to compute the distortion necessary from their respective ground-state structures to reach the transition state geometries alongside calculating the stabilizing interaction energies necessary to achieve the transition structure barriers.

Significant structural differences were observed in Br^–^ complexation within the pathways leading to stepwise **C–N Cleavage TS A** and the **Concerted [2,3]-TS C**. In the stepwise **C–N Cleavage TS A** complexation placed the anionic Br^–^ in a pocket of stabilizing nonclassical C–H/N^+^–CH···Br^–^ hydrogen bond interactions with both the catalyst acyl ammonium enolate and benzofuranyl species as previously reported.^22,92–94^ In the concerted rearrangement structure, an N^+^–CH···Br^–^ hydrogen bonding contact as well as two benzofuranyl C–H···Br^–^ interactions are observed. As has been reported previously, Br^–^ complexation was energetically crucial to the stabilization of the transition structures, consistent with previous literature observations that suggest that isothiouronium electrostatic interactions play a significant role in isothiourea-catalyzed transformations.^95^ However, in departure from previous cases, the differential Br^–^ complexation showed limited energetic differentiation between the two transition structures. This can be inferred from the distortion-interaction analysis, where the computed interaction energies for stepwise **C–N Cleavage TS A** and the **Concerted [2,3]-TS C** are similar (−3.8 and −5.1 kcal/mol, respectively).

The most significant differentiating factor between the concerted and stepwise processes was the distortion of the catalyst acyl ammonium enolate complex. This enolate within **Concerted [2,3]-TS C** exhibited significantly greater distortion than in the stepwise **C–N Cleavage TS A** by greater than 6 kcal/mol (13.6 and 6.9 kcal/mol, respectively). This is the dominant factor that favored the stepwise pathway over the concerted pathway. A close examination of the structures revealed that the distortion from planarity of the enolate (as measured by the angle O–C=C–N) within **Concerted [2,3]-TS C** (−15°) was significantly greater than that of the **C–N Cleavage TS A** (5°). Presumably, this arises because the **Concerted [2,3]-TS C** must distort to accommodate the constraints of simultaneous C–C bond formation and C–N bond cleavage, whereas the stepwise transition state need only satisfy the C–N bond cleavage, minimizing perturbation from planarity. Similar factors have been reported in the use of proline-derived catalysts in the organocatalysis literature, where the planarity of the forming iminium dictated the selectivity between the *syn* and *anti*-enamine transition structures.^92–94^

The first step in the stepwise transformation, **C–N Cleavage TS A**, was the rate-determining transition structure. It was less distorted than the second step **C–C Recombination TS B** (11.7 and 20.0 kcal/mol, respectively). However, the significantly greater interaction energies of the second step **C–C Recombination TS B** by >10 kcal/mol (−3.8 and −14.4 kcal/mol, respectively) override this distortion to make it more stable than the first step **C–N Cleavage TS A** by >2 kcal/mol. The greater stabilization of the **C–C Recombination TS B** is considered to arise from electrostatic interactions between the π groups of the cationic benzofuran and the catalyst acyl ammonium enolate in the recombination transition state. In **C–N Cleavage TS A**, the bond breaking led to the production of the cationic benzofuran, creating charge separation between the catalyst acyl ammonium enolate carbon and the cationic benzofuran in the **C–C Recombination TS B**, consequently creating significantly different interaction energies.

To experimentally probe this hypothesis, a series of crossover experiments was designed. Taking an equimolar mixture of two distinct ammonium salts **55** and **56** and treating them under the optimized reaction conditions gave no crossover products by comparison with authentic standards, giving only **33** (97:3 er) and **17** (95:5 er), suggesting an intramolecular process. As the rearrangement product yields are sensitive to structural variation, further validation of this hypothesis sought to minimize the chemical differentiation of substrates in the crossover studies.

**Figure 3:**
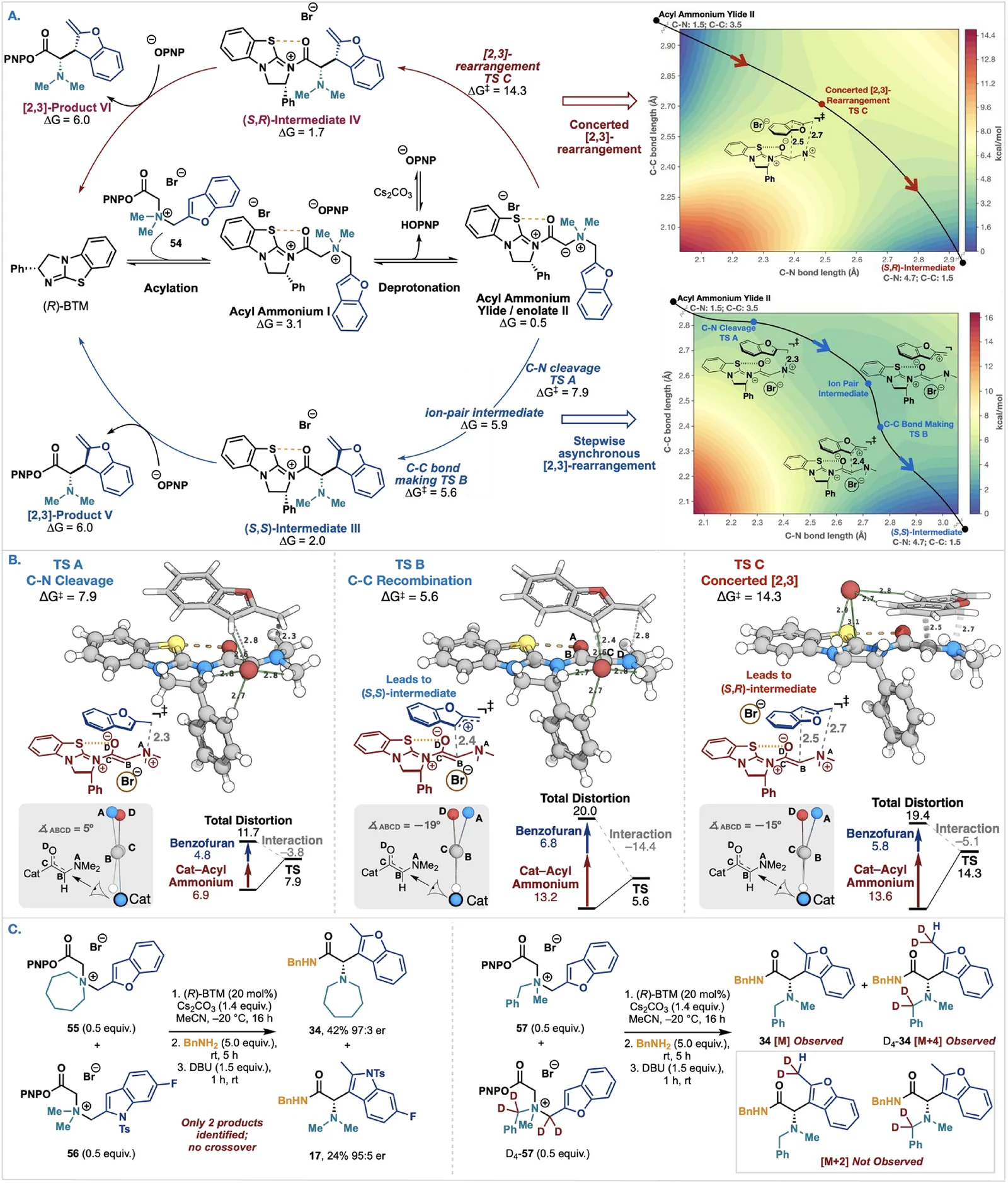
(A). Proposed catalytic cycle and computed energies (in kcal mol^–1^) for the stepwise and concerted [2,3]-rearrangement processes shown on the left (blue), and the minor [2,3]-product forming processes shown on the right (red and orange) alongside computed potential energy surfaces of the stepwise (left) and concerted (right) [2,3]-rearrangement processes. (B). Computed transition states (TS A–C) and energies (in kcal mol^–1^) for stepwise C–N bond cleavage and C–C bond-recombination processes and concerted [2,3]-sigmatropic rearrangement leading to the (*S*,*S*)- and (*S*,*R*)-configured [2,3]-products. (C). Crossover experiments to determine intermolecularity of [2,3]-rearrangement.

The use of ammonium salt isotopologues in crossover studies was considered, with a D_4_-containing isotopically labeled salt prepared (containing D_2_ labeling at the benzylic and heterobenzylic positions) and utilized in a crossover experiment with its H_4_-isomer. Treatment of an equimolar mixture of the isotopologue ammonium salts **57** and D_4_-**57** under the optimized reaction conditions gave the rearrangement product in a 74% yield and high enantioselectivity (96:4 er). ^1^H and ^2^H NMR analyses confirmed a 50:50 ratio of products **31** and D_4_-**31**, with deuterium present within the N-benzyl substituent and the benzofuran methyl substituent (D_4_-**31**). Furthermore, high-resolution mass spectrometry revealed no evidence of deuterium scrambling, which is consistent with an intramolecular rearrangement. To further probe the possibility of intermediate trapping, the [2,3]-rearrangement in the presence of one equivalent of TEMPO, a common trap for radicals, anions, and cations, was investigated.^96–99^ This experiment was designed to assess whether any reactive intermediate—potentially formed *via* fragmentation of the ylide—could be intercepted. Consistent with our previous findings in the studies of [2,3]-rearrangements of allylic ammonium ylides, no TEMPO adducts were detected.^22^ Instead, only the expected rearrangement product was observed. This outcome further supports the hypothesis that any intermediates that form are transient under the reaction conditions employed.

The experimental and computational evidence suggest that this [2,3]-rearrangement preferentially proceeds through a stepwise mechanism involving C–N bond cleavage to generate a discrete ion pair intermediate that is followed by C–C bond formation. In this process, the second C–C bond-forming event is so fast that scrambling is not observed, meaning this is indistinguishable from, and effectively, a concerted asynchronous process. The insight gained from this dual computational and mechanistic investigation emphasizes the importance of exploring sigmatropic rearrangement processes in detail rather than assuming a concerted reaction pathway. These results are significant, as they indicate that a catalytic asymmetric rearrangement conventionally represented as a concerted [2,3]-sigmatropic process can instead operate through a tight ion pair manifold without observable crossover or loss of stereochemical fidelity. Thus, the absence of crossover is not, on its own, sufficient to distinguish a concerted process from a stepwise process followed by rapid in-cage recombination.

## Conclusion

This study demonstrated the feasibility of the enantioselective Sommelet–Hauser reaction of heterobenzylic ammonium ylides using an isothiourea Lewis base catalyst. The process incorporates a dearomatizing [2,3]-sigmatropic rearrangement followed by tautomerization, generating unnatural α-heteroaryl α-amino acid derivatives with high enantioselectivity and good to moderate yields under optimal conditions using an operationally simple protocol. The isothiourea (*R*)-BTM leads to optimal product enantioselectivity, while DBU was employed to effect a one-pot, three-step process. The developed process is tolerant of cyclic and acyclic N-substituents, the inclusion of indolyl and benzofuranyl substituents, as well as a range of pronucleophiles that lead to amide, ester, and alcohol products with excellent enantiocontrol.

Extensive computational analyses showed that Br^–^ complexation was energetically crucial to transition structure stabilization. A stepwise process (involving initial C–N bond cleavage to generate an ion pair, followed by C–C bond formation) is favored over a concerted [2,3]-rearrangement. A three-component distortion-interaction analysis indicates that the concerted [2,3]-transition state must distort to accommodate the constraints of simultaneous C–C bond formation and C–N bond cleavage, whereas the stepwise transition state needs only to satisfy the C–N bond cleavage, minimizing perturbation from planarity. Crossover experiments, including the use of isotopologue salt derivatives to minimize chemical differentiation of substrates, indicate an intramolecular rearrangement process, supporting the concerted asynchronous mechanism suggested through computation. This process offers an insight into the rich mechanistic nature of reactions promoted by isothiourea catalysts and paves the way for further exploration of ammonium ylide rearrangements and synthetic methodologies leveraging the unique properties of heteroaromatic systems.

## Supplementary Material



## Data Availability

The data underpinning this manuscript are available from the University of St Andrews Research Portal, Pure ID: 332675853, “The Catalytic Enantioselective Sommelet-Hauser Rearrangement” https://doi.org/10.17630/af081466-f789-4e13-98e9-e7107ab2f1ff.

## Notes

Experimental procedures, characterization data, NMR spectra, HPLC traces, DFT calculations, and X-ray crystallographic data for **16·HCl** (PDF)

## References

[1] Schreiber, S. L. , Ed., Comprehensive Organic Synthesis. 1: Additions to C-X pi-Bonds, Part 1/Vol. ed.: Stuart L. Schreiber; Pergamon Pr, Oxford, 1993.

[2] Trost B. M. (1995). Atom Economy–A
Challenge for Organic Synthesis: Homogeneous Catalysis Leads the Way. Angew. Chem., Int. Ed..

[3] Schwartz Z., Valiton C., Lovasz M., Roberts A. G. (2024). Recent Applications
of Ammonium Ylide Based [2,3]-Sigmatropic and [1,2]-Stevens Rearrangements
To Transform Amines into Natural Products. Synthesis.

[4] Qamar R., Saeed A. (2018). Pericyclic Reactions
in the Syntheses of Densely Functionalized Heterocycles. Curr. Org. Synth..

[5] Seashore-Ludlow, B. ; Somfai, P. Stereoselective Synthesis of Drugs and Natural Products; John Wiley & Sons, Ltd, 2013; pp 1–26.

[6] Vanecko J. A., Wan H., West F. G. (2006). Recent advances in the Stevens rearrangement of ammonium
ylides. Application to the synthesis of alkaloid natural products. Tetrahedron.

[7] Jana S., Guo Y., Koenigs R. M. (2021). Recent Perspectives
on Rearrangement Reactions of Ylides
via Carbene Transfer Reactions. Chem. –
Eur. J..

[8] Empel C., Jana S., Koenigs R. M. (2021). Advances
in [1,2]-Sigmatropic Rearrangements
of Onium Ylides via Carbene Transfer Reactions. Synthesis.

[9] Nair V. N., Tambar U. K. (2022). Catalytic rearrangements
of onium ylides in aromatic
systems. Org. Biomol. Chem..

[10] Carreras V., Tanbouza N., Ollevier T. (2021). The Power
of Iron Catalysis in Diazo
Chemistry. Synthesis.

[11] Sheng Z., Zhang Z., Chu C., Zhang Y., Wang J. (2017). Transition
metal-catalyzed [2,3]-sigmatropic rearrangements of ylides: An update
of the most recent advances. Tetrahedron.

[12] Banert K. (2020). Functionalized
Allenes: Generation by Sigmatropic Rearrangement and Application in
Heterocyclic Chemistry. Curr. Org. Chem..

[13] Doyle M. P., Griffin J. H., Chinn M. S., Van Leusen D. (1984). Rearrangements
of ylides generated from reactions of diazo compounds with allyl acetals
and thioketals by catalytic methods. Heteroatom acceleration of the
[2,3]-sigmatropic rearrangement. J. Org. Chem..

[14] Aggarwal V. K., Ferrara M., Hainz R., Spey S. E. (1999). [2,3]-Sigmatropic
rearrangement of allylic sulfur ylides derived from trimethylsilyldiazomethane
(TMSD). Tetrahedron Lett..

[15] Carter D. S., Van Vranken D. L. (1999). Metal-catalyzed
ylide formation and [2,3] sigmatropic
rearrangement of allyl sulfides with trimethylsilyldiazomethane. Tetrahedron Lett..

[16] Li L., Chen B., Chen J., Huang Y. (2020). Enantioselective Intramolecular
[2,3]-Sigmatropic Rearrangement of Aldehydes via a Sulfonium Enamine
Intermediate. Angew. Chem., Int. Ed..

[17] Doyle M. P., Tamblyn W. H., Bagheri V. (1981). Highly effective
catalytic methods
for ylide generation from diazo compounds. Mechanism of the rhodium-
and copper-catalyzed reactions with allylic compounds. J. Org. Chem..

[18] Roskamp E. J., Johnson C. R. (1986). Generation and rearrangements of oxonium ylides. J. Am. Chem. Soc..

[19] Li Z., Davies H. M. L. (2010). Enantioselective C–C Bond Formation by Rhodium-Catalyzed
Tandem Ylide Formation/[2,3]-Sigmatropic Rearrangement between Donor/Acceptor
Carbenoids and Allylic Alcohols. J. Am. Chem.
Soc..

[20] Doyle M. P., Forbes D. C., Vasbinder M. M., Peterson C. S. (1998). Enantiocontrol in
the Generation and Diastereoselective Reactions of Catalytically Generated
Oxonium and Iodonium Ylides. Metal-Stabilized Ylides as Reaction Intermediates. J. Am. Chem. Soc..

[21] West T. H., Spoehrle S. S. M., Kasten K., Taylor J. E., Smith A. D. (2015). Catalytic
Stereoselective [2,3]-Rearrangement Reactions. ACS Catal..

[22] West T. H., Walden D. M., Taylor J. E., Brueckner A. C., Johnston R. C., Cheong P. H.-Y., Lloyd-Jones G. C., Smith A. D. (2017). Catalytic Enantioselective [2,3]-Rearrangements of
Allylic Ammonium Ylides: A Mechanistic and Computational Study. J. Am. Chem. Soc..

[23] West T. H., Daniels D. S. B., Slawin A. M. Z., Smith A. D. (2014). An Isothiourea-Catalyzed
Asymmetric [2,3]-Rearrangement of Allylic Ammonium Ylides. J. Am. Chem. Soc..

[24] Clark J. S., Hodgson P. B. (1994). Intramolecular generation and rearrangement of ammonium
ylides from copper carbenoids: a general method for the synthesis
of cyclic amines. J. Chem. Soc., Chem. Commun..

[25] West F. G., Naidu B. N. (1993). New route to substituted
piperidines via the Stevens
[1,2]-shift of ammonium ylides. J. Am. Chem.
Soc..

[26] West F. G., Naidu B. N. (1994). Piperidines via Ammonium Ylide [1,2]-Shifts: A Concise,
Enantioselective Route to (−)-Epilupinine from Proline Ester. J. Am. Chem. Soc..

[27] Clark J. S., Middleton M. D. (2002). Synthesis of Novel α-Substituted and α,α-Disubstituted
Amino Acids by Rearrangement of Ammonium Ylides Generated from Metal
Carbenoids. Org. Lett..

[28] Sançon J., Sweeney J. B. (2010). Probing the Effect
of Allylic Substitution on Cyclic
Ammonium Ylid Rearrangements. Synlett.

[29] Xu B., Tambar U. K. (2016). Ligand-Controlled
Regiodivergence in the Copper-Catalyzed
[2,3]- and [1,2]-Rearrangements of Iodonium Ylides. J. Am. Chem. Soc..

[30] Xu B., Gartman J. A., Tambar U. K. (2017). Copper-catalyzed [1,2]-rearrangements
of allylic iodides and aryl α-diazoacetates. Tetrahedron.

[31] Xu B., Tambar U. K. (2017). Copper-Catalyzed Enantio-, Diastereo-, and Regioselective
[2,3]-Rearrangements of Iodonium Ylides. Angew.
Chem., Int. Ed..

[32] Sweeney J. B. (2009). Sigmatropic
rearrangements of ‘onium’ ylids. Chem. Soc. Rev..

[33] Nakai T., Mikami K. (1986). [2,3]-Wittig sigmatropic rearrangements in organic
synthesis. Chem. Rev..

[34] Hartley W. C., Kasten K., Greenhalgh M. D., Feoktistova T., Wise H. R., Laddusaw J. M., Frost A. B., Ng S., Slawin A. M. Z., Bode B. E., Cheong P. H.-Y., Smith A. D. (2025). In-Cage
Recombination Facilitates the Enantioselective Organocatalytic [1,2]-Rearrangement
of Allylic Ammonium Ylides. J. Am. Chem. Soc..

[35] Sommelet M. (1937). On a particular
mode of intramolecular rearrangement. Compt.
Rend..

[36] Kantor S. W., Hauser C. R. (1951). Rearrangements of
Benzyltrimethylammonium Ion and Related
Quaternary Ammonium Ions by Sodium Amide Involving Migration into
the Ring. J. Am. Chem. Soc..

[37] Tayama E., Shimizu G., Nakao R. (2022). Base-induced
Sommelet–Hauser
rearrangement of *N*-(pyridinylmethyl) tetraalkylammonium
salts. Tetrahedron.

[38] Shen Y., Huang A., Lu X., Jia A., Luo S., Li X.-X., Tang S. (2025). Substituent-Controlled
Regiodivergent
Rearrangement of Gramine Ammonium Ylide. J.
Org. Chem..

[39] Nishimura T., Zhang C., Maeda Y., Shirai N., Ikeda S.-i., Sato Y. (1999). Comparison of the reaction of benzylammonium N-methylides with that
of benzylsulfonium S-methylides. Chem. Pharm.
Bull..

[40] Zhang C., Maeda Y., Sato Y. (1998). Reaction of
N,N-Dialkyl-N-(trimethylsilyl)­methyl-γ-substituted
Allylammonium Salts with Cesium Fluoride. Chem.
Pharm. Bull..

[41] Maeda Y., Sato Y. (1997). Mechanism of the Stevens
rearrangement of ammonium ylides. J. Chem. Soc.,
Perkin Trans. 1.

[42] Tanaka T., Shirai N., Sato Y. (1992). Substituent
Effect on the Benzyl
Groups in Sommelet-Hauser Rearrangement. Chem.
Pharm. Bull..

[43] Tayama E. (2022). Recent Advances
in the Generation of Onium Ylides for Sommelet–Hauser Rearrangements. Synthesis.

[44] Pan C., Guo W., Gu Z. (2018). Unusual biaryl torsional strain promotes reactivity
in Cu-catalyzed Sommelet–Hauser rearrangement. Chem. Sci..

[45] Tayama E., Sotome S. (2018). Dearomative [2,3] sigmatropic rearrangement of ammonium
ylides followed by 1,4-elimination to form α-(ortho-vinylphenyl)­amino
acid esters. Org. Biomol. Chem..

[46] Tayama E., Otoyama S., Tanaka H. (2009). Resolution
of nitrogen-centered chiral
tetraalkylammonium salts: application to [1,2] Stevens rearrangements
with *N*-to-*C* chirality transmission. Tetrahedron: Asymmetry.

[47] Hiroi K., Nakazawa K. (1980). Asymmetric Induction in the [2,3]
Sigmatropic Rearrangement
via Chiral Ammonium Ylides. Chem. Lett..

[48] Glaeske K. W., West F. G. (1999). Chirality Transfer
from Carbon to Nitrogen to Carbon
via Cyclic Ammonium Ylides. Org. Lett..

[49] Arboré A. P. A., Cane-Honeysett D. J., Coldham I., Middleton M. L. (2000). A Rapid
Approach to Amino-Acid Derivatives by [2,3]-Stevens Rearrangement. Synlett.

[50] Tayama E., Nanbara S., Nakai T. (2006). Asymmetric [1,2] Stevens Rearrangement
of (S)-N-Benzylic Proline-derived Ammonium Salts under Biphasic Conditions. Chem. Lett..

[51] Sweeney J. B., Tavassoli A., Workman J. A. (2006). Asymmetric ammonium ylid rearrangements:
the effect of nitrogen asymmetry. Tetrahedron.

[52] Tayama E., Watanabe K., Matano Y. (2016). Ring-Strain
Effects in Base-Induced
Sommelet–Hauser Rearrangement: Application to Successive Stereocontrolled
Transformations. Eur. J. Org. Chem..

[53] Tayama E., Kimura H. (2007). Asymmetric Sommelet–Hauser
Rearrangement of
N-Benzylic Ammonium Salts. Angew. Chem., Int.
Ed..

[54] Tayama E., Hirano K., Baba S. (2020). Base-induced Sommelet–Hauser
rearrangement of *N*-(α-(2-oxyethyl)­branched)­benzylic
glycine ester-derived ammonium salts via a chelated intermediate. Tetrahedron.

[55] Tayama E., Takedachi K., Iwamoto H., Hasegawa E. (2010). Remarkable
enhancement
effect of potassium *tert*-butoxide/THF solution in
base-induced Sommelet–Hauser rearrangements. Tetrahedron.

[56] Lin X., Yang W., Yang W., Liu X., Feng X. (2019). Asymmetric
Catalytic [2,3] Stevens and Sommelet–Hauser Rearrangements
of α-Diazo Pyrazoleamides with Sulfides. Angew. Chem., Int. Ed..

[57] Li S.-S., Wang J. (2020). Cu­(I)/Chiral Bisoxazoline-Catalyzed Enantioselective Sommelet–Hauser
Rearrangement of Sulfonium Ylides. J. Org. Chem..

[58] Birman V. B., Li X., Han Z. (2007). Nonaromatic
Amidine Derivatives as Acylation Catalysts. Org. Lett..

[59] McLaughlin C., Smith A. D. (2021). Generation
and Reactivity of C(1)-Ammonium Enolates
by Using Isothiourea Catalysis. Chem. –
Eur. J..

[60] Morrill L. C., Smith A. D. (2014). Organocatalytic
Lewis base functionalisation of carboxylic
acids, esters and anhydrides via C1-ammonium or azolium enolates. Chem. Soc. Rev..

[61] Bitai J., Westwood M. T., Smith A. D. (2021). α,β-Unsaturated acyl
ammonium species as reactive intermediates in organocatalysis: an
update. Org. Biomol. Chem..

[62] Greenhalgh M. D., Qu S., Slawin A. M. Z., Smith A. D. (2018). Multiple roles of aryloxide leaving
groups in enantioselective annulations employing α,β-unsaturated
acyl ammonium catalysis. Chem. Sci..

[63] Vellalath S., Romo D. (2016). Asymmetric Organocatalysis:
The Emerging Utility of α,β-Unsaturated
Acylammonium Salts. Angew. Chem., Int. Ed..

[64] Biswas A., Mondal H., Maji M. S. (2020). Synthesis
of Heterocycles by isothiourea
organocatalysis. J. Heterocycl. Chem..

[65] Hartley W. C., O’Riordan T. J. C., Smith A. D. (2017). Aryloxide-Promoted
Catalyst Turnover in Lewis Base Organocatalysis. Synthesis.

[66] Merad J., Pons J.-M., Chuzel O., Bressy C. (2016). Enantioselective Catalysis
by Chiral Isothioureas. Eur. J. Org. Chem..

[67] Birman V. B. (2016). Amidine-based
catalysts (ABCs): Design, development, and applications. Aldrichimica Acta.

[68] Taylor J. E., Bull S. D., Williams J. M. J. (2012). Amidines, isothioureas, and guanidines
as nucleophilic catalysts. Chem. Soc. Rev..

[69] Young C. M., Elmi A., Pascoe D. J., Morris R. K., McLaughlin C., Woods A. M., Frost A. B., de la Houpliere A., Ling K. B., Smith T. K., Slawin A. M. Z., Willoughby P. H., Cockroft S. L., Smith A. D. (2020). The Importance
of 1,5-Oxygen···Chalcogen
Interactions in Enantioselective Isochalcogenourea Catalysis. Angew. Chem., Int. Ed..

[70] Stockhammer L., Kasten K., Eitzinger A., Vogl L. S., Piringer M., Weinzierl D., Ofial A. R., Smith A. D., Waser M. (2025). From Oxygen
to Tellurium: The Impact of the Chalcogen on Nucleophilicities and
Basicities of Isochalcogenourea Catalysts. Angew.
Chem., Int. Ed..

[71] West T. H., Spoehrle S. S. M., Smith A. D. (2017). Isothiourea-catalysed chemo- and
enantioselective [2,3]-sigmatropic rearrangements of *N*, *N*-diallyl allylic ammonium ylides. Tetrahedron.

[72] Spoehrle S. S. M., West T. H., Taylor J. E., Slawin A. M. Z., Smith A. D. (2017). Tandem
Palladium and Isothiourea Relay Catalysis: Enantioselective Synthesis
of α-Amino Acid Derivatives via Allylic Amination and [2,3]-Sigmatropic
Rearrangement. J. Am. Chem. Soc..

[73] Kasten K., Slawin A. M. Z., Smith A. D. (2017). Enantioselective
Synthesis of β-Fluoro-β-aryl-α-aminopentenamides
by Organocatalytic [2,3]-Sigmatropic Rearrangement. Org. Lett..

[74] Biswas B., Singleton D. A. (2015). Controlling Selectivity by Controlling
the Path of
Trajectories. J. Am. Chem. Soc..

[75] Biswas B., Collins S. C., Singleton D. A. (2014). Dynamics
and a Unified Understanding
of Competitive [2,3]- and [1,2]-Sigmatropic Rearrangements Based on
a Study of Ammonium Ylides. J. Am. Chem. Soc..

[76] Jaber D. M., Burgin R. N., Helper M., Zavalij P. Y., Doyle M. P. (2012). Competitive
[2,3]- and [1,2]-Oxonium Ylide Rearrangements. Concerted or Stepwise?. Org. Lett..

[77] Ollis W. D., Rey M., Sutherland I. O. (1983). Base catalysed rearrangements involving
ylide intermediates. Part 15. The mechanism of the Stevens [1,2] rearrangement. J. Chem. Soc., Perkin Trans. 1.

[78] Heard G. L., Yates B. F. (1996). Competing Rearrangements of Ammonium Ylides: A Quantum
Theoretical Study. J. Org. Chem..

[79] Ghigo G., Cagnina S., Maranzana A., Tonachini G. (2010). The mechanism
of the Stevens and Sommelet–Hauser Rearrangements. A Theoretical
Study. J. Org. Chem..

[80] Combs J. R., Carter D. S., Gu F., Jin X., Martin C. M., Resendiz E. S., Van Vranken D. L. (2023). Rhodium­(II)-Catalyzed
Hinsberg Dearomatization
Using Trimethylsilyldiazomethane. Org. Lett..

[81] Martin N. H., Rowe J. E., Pittman E. L. (2010). Computed
NMR shielding increments
over benzo-analogs of unsaturated five-membered ring heterocyclic
compounds as a measure of aromaticity. J. Mol.
Graphics Modell..

[82] Matos, M. A. R. ; Liebman, J. F. Aromaticity in Heterocyclic Compounds; Krygowski, T. M. ; Cyrański, M. K. , Eds.; Springer: Berlin, Heidelberg, 2009; pp 1–26.

[83] Mandado M., González-Moa M. J., Mosquera R. A. (2007). QTAIM n-center delocalization
indices as descriptors of aromaticity in mono and poly heterocycles. J. Comput. Chem..

[84] Kleinpeter E., Klod S., Koch A. (2007). Visualization of through space NMR
shieldings of aromatic and anti-aromatic molecules and a simple means
to compare and estimate aromaticity. J. Mol.
Struct.: Theochem.

[85] Birman V. B., Li X. (2006). Benzotetramisole: A
Remarkably Enantioselective Acyl Transfer Catalyst. Org. Lett..

[86] Tanaka T., Shirai N., Sugimori J., Sato Y. (1992). Selection of a Sommelet-Hauser
or a Stevens rearrangement pathway of N,N-dimethyl­(substituted benzyl)­ammonium
N-alkylides. J. Org. Chem..

[87] Walsh M. P., Phelps J. M., Lennon M. E., Yufit D. S., Kitching M. O. (2021). Enantioselective
synthesis of ammonium cations. Nature.

[88] Rios A., Amyes T. L., Richard J. P. (2000). Formation
and Stability of Organic
Zwitterions in Aqueous Solution: Enolates of the Amino Acid Glycine
and Its Derivatives. J. Am. Chem. Soc..

[89] Bordwell F. G., Fried H. E. (1981). Acidities of the
hydrogen-carbon protons in carboxylic
esters, amides, and nitriles. J. Org. Chem..

[90] Kang T., O’Yang J., Kasten K., Allsop S. S., Lewis-Atwell T., Farrar E. H. E., Juhl M., Cordes D. B., McKay A. P., Grayson M. N., Smith A. D. (2026). The catalytic enantioselective [1,2]-Wittig
rearrangement cascade of allylic ethers. Nat.
Chem..

[91] Frisch, M. J. ; Trucks, G. W. ; Schlegel, H. B. ; Scuseria, G. E. ; Robb, M. A. ; Cheeseman, J. R. ; Scalmani, G. ; Barone, V. ; Petersson, G. A. ; Nakatsuji, H. ; Li, X. ; Caricato, M. ; Marenich, A. V. ; Bloino, J. ; Janesko, B. G. ; Gomperts, R. ; Mennucci, B. ; Hratchian, H. P. ; Ortiz, J. V. ; Izmaylov, A. F. ; Sonnenberg, J. L. ; Williams; Ding, F. ; Lipparini, F. ; Egidi, F. ; Goings, J. ; Peng, B. ; Petrone, A. ; Henderson, T. ; Ranasinghe, D. ; Zakrzewski, V. G. ; Gao, J. ; Rega, N. ; Zheng, G. ; Liang, W. ; Hada, M. ; Ehara, M. ; Toyota, K. ; Fukuda, R. ; Hasegawa, J. ; Ishida, M. ; Nakajima, T. ; Honda, Y. ; Kitao, O. ; Nakai, H. ; Vreven, T. ; Throssell, K. ; Montgomery Jr, J. A. ; Peralta, J. E. ; Ogliaro, F. ; Bearpark, M. J. ; Heyd, J. J. ; Brothers, E. N. ; Kudin, K. N. ; Staroverov, V. N. ; Keith, T. A. ; Kobayashi, R. ; Normand, J. ; Raghavachari, K. ; Rendell, A. P. ; Burant, J. C. ; Iyengar, S. S. ; Tomasi, J. ; Cossi, M. ; Millam, J. M. ; Klene, M. ; Adamo, C. ; Cammi, R. ; Ochterski, J. W. ; Martin, R. L. ; Morokuma, K. ; Farkas, O. ; Foresman, J. B. ; Fox, D. J. Gaussian 16, Revision A.03; Gaussian, Inc.: Wallingford CT, 2016.

[92] Bahmanyar S., Houk K. N. (2001). The Origin of Stereoselectivity
in Proline-Catalyzed
Intramolecular Aldol Reactions. J. Am. Chem.
Soc..

[93] Houk K. N., List B. (2004). Asymmetric Organocatalysis. Acc. Chem. Res..

[94] Cheong P.
H.-Y., Legault C. Y., Um J. M., Çelebi-Ölçüm N., Houk K. N. (2011). Quantum Mechanical Investigations of Organocatalysis:
Mechanisms, Reactivities, and Selectivities. Chem. Rev..

[95] Greenhalgh M. D., Smith S. M., Walden D. M., Taylor J. E., Brice Z., Robinson E. R. T., Fallan C., Cordes D. B., Slawin A. M. Z., Richardson H. C., Grove M. A., Cheong P. H.-Y., Smith A. D. (2018). A C = O···Isothiouronium
Interaction
Dictates Enantiodiscrimination in Acylative Kinetic Resolutions of
Tertiary Heterocyclic Alcohols. Angew. Chem.,
Int. Ed..

[96] Vogler T., Studer A. (2008). Applications of TEMPO in Synthesis. Synthesis.

[97] Leifert D., Studer A. (2023). Organic Synthesis Using Nitroxides. Chem. Rev..

[98] Whitesides G. M., Newirth T. L. (1975). Reaction of n-butyllithium and 2,2,6,6-tetramethylpiperidine
nitroxyl. J. Org. Chem..

[99] Nagashima T., Curran D. P. (1996). Reactions of TEMPO
with Alkylsamarium and Other Organometallic
Reagents. Synlett.

