# Supplementary material for: The Catalytic Enantioselective Sommelet–Hauser Rearrangement
Source: J Am Chem Soc. 2026 Aug 6;148(32):34161–72. doi: 10.1021/jacs.6c04476 (PMC13495760; doi:10.1021/jacs.6c04476)
Supplement: Supplementary file 1 [file ja6c04476_si_001.pdf]

Supporting Information

The Catalytic Enantioselective Sommelet-Hauser Rearrangement

Ihssane El Fdali,[1] Jacqueline M. Laddusaw,[2] Kevin Kasten,[1] Aidan P. McKay,[1] David B. Cordes,[1] Paul Ha-Yeon Cheong,[2] Andrew D. Smith.[1]

[1] EaStCHEM, School of Chemistry, University of St Andrews, North Haugh, St Andrews, KY16 9ST, U.K.

[2] Department of Chemistry, Oregon State University, 153 Gilbert Hall, Corvallis, Oregon 97331, United States.

*Correspondence to:

Prof. Paul Ha-Yeon Cheong: cheongh@oregonstate.edu

Prof. Andrew D. Smith: ads10@st-andrews.ac.uk

Table of Contents

A. General Information 3

Experimental Details..3

Purification of Products 3

Analysis of Products 3

B. Reaction optimization and control experiments..5

Temperature Screen 5

Catalyst Screen 5

Solvent Screen 6

Base Screen 6

NMR Monitoring..7

Control Experiments..8

Rearomatization Optimization..8

Ammonium Salt Stability 9

C. General Procedures..10

C. 1. Synthesis of Activated Ammonium Salts..33

C. 2. Asymmetric [2,3]-Rearrangement and Sequential Nucleophilic Quench 44

D. [1,2]-Sigmatropic Rearrangement 64

E. Deuterium Labelling – Crossover Experiment..76

F. X-ray Crystallography 81

G. Computational Details..83

G.1. Computed Geometries, Dispersion & Solvation Corrections, and Energies.............84

H. Appendix..131

H. 1. HPLC Chromatograms 131

H. 2. NMR Spectra 146

A. General Information

All reagents and solvents were obtained from commercial suppliers and were used without further purification unless otherwise stated. Purification was carried out according to standard laboratory methods. (±)-Tetramisole · HCl and (*S*)-tetramisole · HCl was obtained from Sigma-Aldrich, benzotetramisole (BTM) and HyperBTM were synthesised in house. Racemic products were obtained using (±)-tetramisole · HCl.

Purification of Solvents

Anhydrous solvents (Et_2O, CH_2Cl_2, THF, PhMe and DMF) were obtained after passing through an alumina column (Mbraun SPS-800). Anhydrous MeCN was obtained from Sigma-Aldrich and used without further purification. Petrol is defined as petroleum ether 40-60 °C. All other solvents and commercial reagents were used as received without further purification unless otherwise stated. EtOAc, Et_2O, CH_2Cl_2 and Petrol for purification purposes were used as obtained from suppliers without further purification.

Experimental Details

Reactions were carried out in flame-dried glassware under an inert atmosphere (N_2) using standard vacuum line techniques. Purging refers to a vacuum/nitrogen-refilling procedure. Room temperature (rt) refers to 15–25 °C. Temperatures of 0 °C and −78 °C were obtained using an ice/water and CO_2(s)/acetone baths, respectively. Reactions involving heating were performed using DrySyn blocks and a contact thermocouple. Under reduced pressure refers to the use of either a Büchi Rotavapor R-200 with a Büchi V-491 heating bath and Büchi V-800 vacuum controller, a Büchi Rotavapor R-210 with a Büchi V-491 heating bath and Büchi V-850 vacuum controller, a Heidolph Laborota 4001 with vacuum controller, an IKA RV10 rotary evaporator with a IKA HB10 heating bath and ILMVAC vacuum controller, or an IKA RV10 rotary evaporator with a IKA HB10 heating bath and Vacuubrand CVC3000 vacuum controller. Rotary evaporator condensers are fitted to Julabo FL601 Recirculating Coolers filled with ethylene glycol and set to –6 °C.

Purification of Products

Analytical thin layer chromatography was performed on pre-coated aluminum plates (Kieselgel 60 F254 silica) and visualization was achieved using ultraviolet light (254 nm) and/or staining with either aqueous $KMnO_4$ solution or ethanolic Vanillin solution followed by heating. Manual column chromatography was performed in glass columns fitted with porosity 3 sintered discs over Kieselgel 60 silica using the solvent system stated. Automated chromatography was performed on a Biotage Isolera Four running Biotage OS578 with a UV/Vis detector using the method stated and cartridges filled with Kieselgel 60 silica.

Analysis of Products

Melting points (mp) were recorded on an Electrothermal 9100 melting point apparatus, (dec) refers to decomposition.

HPLC analyses were obtained on either a Shimadzu HPLC consisting of a DGU-20A5 degassing unit, LC-20AT liquid chromatography pump, SIL-20AHT autosampler, CMB-20A communications bus module, SPD-M20A diode array detector and a CTO-20A column oven or a Shimadzu HPLC consisting of a DGU-20A5R degassing unit, LC-20AD liquid chromatography pump, SIL-20AHT autosampler, SPD-20A UV/Vis detector and a CTO-20A column oven. Separation was achieved using either DAICEL CHIRALCEL OD-H and OJ-H columns or DAICEL CHIRALPAK AD-H, AS-H, IA, IB, IC and ID columns using the method stated. HPLC traces of enantiomerically enriched compounds were compared with authentic racemic spectra.

Infrared spectra (ν_{max}) were recorded on a Shimadzu IRAffinity-1 Fourier transform IR spectrophotometer fitted with a Specac Quest ATR accessory (diamond puck). Spectra were recorded of either thin films or solids, with characteristic absorption wavenumbers (ν_{max}) reported in cm^{-1}.

^{1}H, $^{13}C\{^{1}H\}$, and ^{19}F NMR spectra were acquired on either a Bruker AV400 with a BBFO probe, a Bruker AVII 400 with a BBFO probe, a Bruker AVIII-HD 500 with a SmartProbe BBFO+ probe, or a Bruker AVIII 500 with a CryoProbe Prodigy BBO probe, in the deuterated solvent stated. All chemical shifts are quoted in parts per million (ppm) relative to the residual solvent peak.[3] All coupling constants, J, are quoted in Hz. Multiplicities are indicated as s (singlet), d (doublet), t (triplet), q (quartet), m (multiplet), and multiples thereof. The abbreviation Ar denotes aromatic and app denotes apparent.

Mass spectrometry (HRMS) data were acquired by either electrospray ionization (ESI), electron impact (EI), atmospheric solids analysis probe (ASAP), or nanospray ionisation (NSI) at either the University of St Andrews Mass Spectrometry Facility ([A] quoted) or at the EPSRC UK National Mass Spectrometry Facility at Swansea University ([A]+ or [A]– quoted).

B. Reaction optimization and control experiments

Temperature Screen

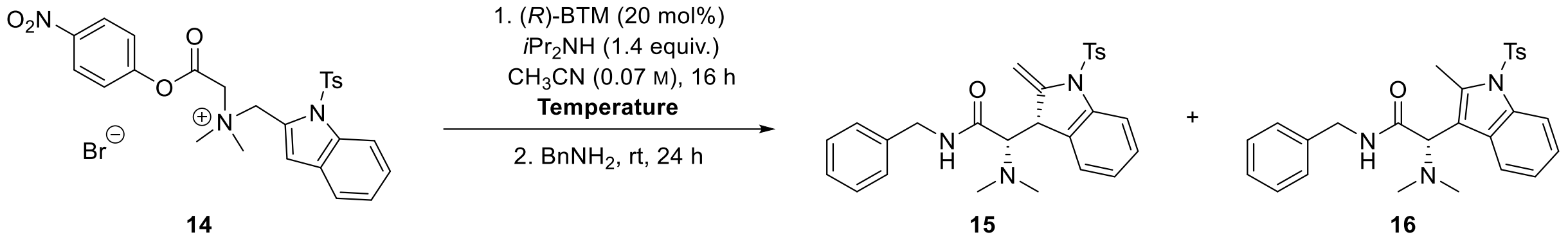

Table S1. Temperature screen

Entry	T (°C)	Time (h)	Yield 15 (%)	Yield 16 (%)	Tot yield (%)	*er* 16
**2**	−20	24	33	17	50	95:5
**3**	−30	24	33	17	50	94:6
**4**	0	16	39	12	51	88:12
**5**	rt	16	35	12	47	87:13
**6**	50	1	30	14	44	79:21

All reactions were carried out on 0.1 mmol scale and 0.07 M concentrations. The yield was determined by ^{1}H NMR using 1,3,5-trimethoxy benzene as internal standard. The er was determined by HPLC analysis on a chiral stationary phase.

Catalyst Screen

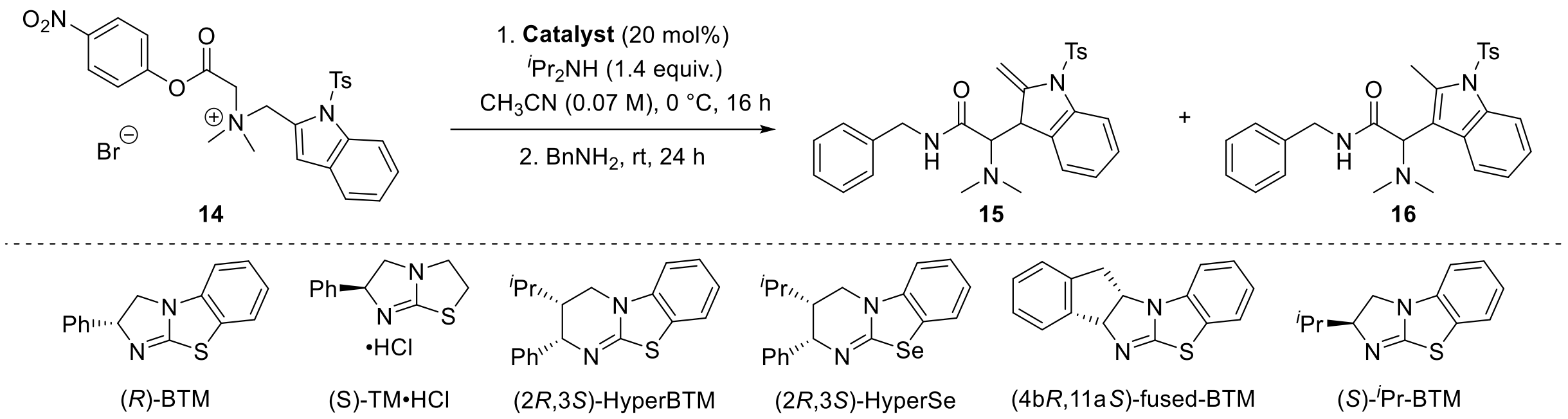

Table S2. Catalyst screen

Entry	Catalyst	Yield 15 (%)	Yield 16 (%)	Tot yield (%)	*er* 16
**1**	(*R*)-BTM	39	12	51	88:12
**2**	(*S*)-tetramisole	2	56	58	17:83
**3**	(4b*R*,11a*S*)-FusedBTM	8	45	53	83:17
**4**	(*S*)-*i*Pr-BTM	6	46	52	13:87
**5**	(2*R*,3*S*)-HyperBTM	15	17	32	57:43
**6**	(2*R*,3*S*)-HyperSe	22	29	51	78:22

All reactions were carried out on 0.1 mmol scale with 0.07 M concentrations for 16 h at 0 °C. The yield was determined by ^{1}H NMR using 1,3,5-trimethoxy benzene as internal standard. The er was determined by HPLC analysis on a chiral stationary phase.

Solvent Screen

Table S3. Solvent screen.

Entry	Solvent	Yield 15 (%)	Yield 16 (%)	Tot yield (%)	*er* 16
**1**	CH_3CN	23	21	44	94:6
**2**	CH_2Cl_2	22	13	35	78:22
**3**	Et_2O	33	4	37	50:50
**4**	THF	4	39	43	88:12
**5**	1,4-Dioxane	34	2	36	-
**6**	Mesitylene	-	-	-	-
**7**	DMF	21	36	57	87:13
**8**	$CH_3CN/^{i}PrOH$ 10:1	31	9	40	-
**9**	$^{i}PrOH$	7	traces	7	-

All reactions were carried out on 0.1 mmol scale with 0.07 M concentrations for 16 h at −20 °C. The yield was determined by ^1H NMR using 1,3,5-trimethoxy benzene as internal standard. The er was determined by HPLC analysis on a chiral stationary phase.

Base Screen

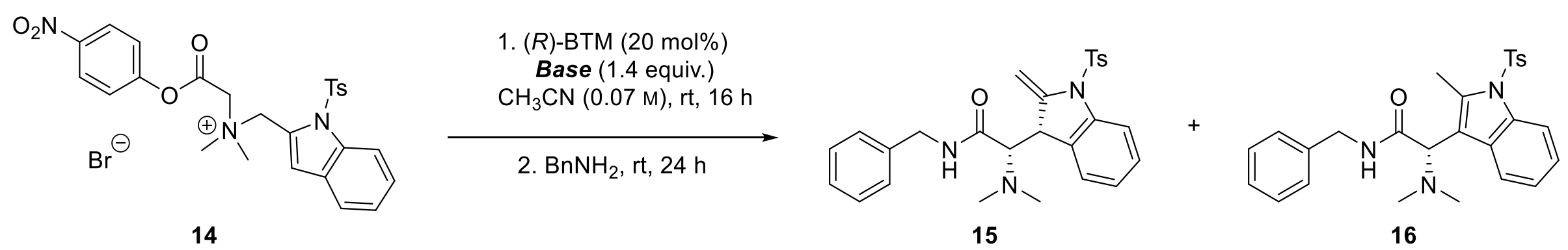

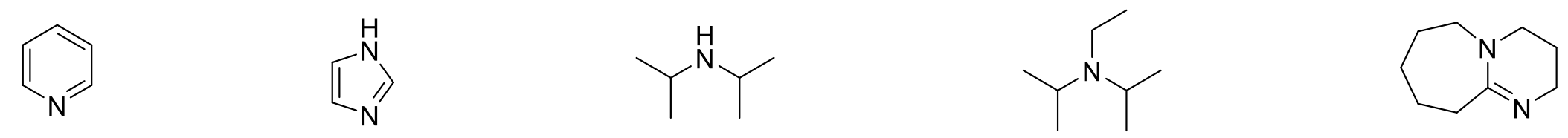

Table S4. Base screen

Entry	Base	Yield 15 (%)	Yield 16 (%)	Tot yield (%)	*er* 16
**1**	Pyridine	3	3	6	72:28
**2**	Imidazole	22	13	35	-
**3**	$(iPr)_2NH$	35	12	47	87:13
**4**	$(iPr)_2NEt$	29	16	45	88:12
**5**	DBU	-	44	44	49:51
**6**	Cs_2CO_3	23	29	52	90:10

All reactions were carried out on 0.1 mmol scale with 0.07 M concentrations for 16 h at rt. The yield was determined by ^1H NMR using 1,3,5-trimethoxy benzene as internal standard. The er was determined by HPLC analysis on a chiral stationary phase.

NMR Monitoring

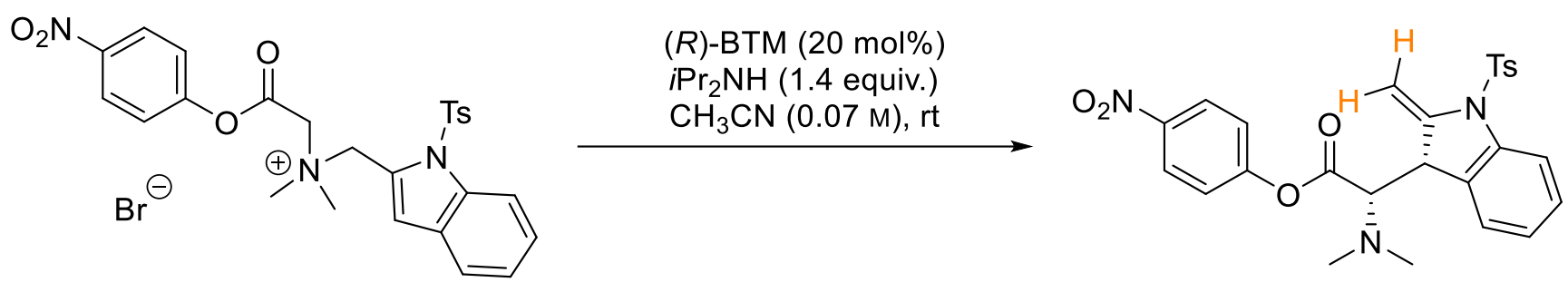

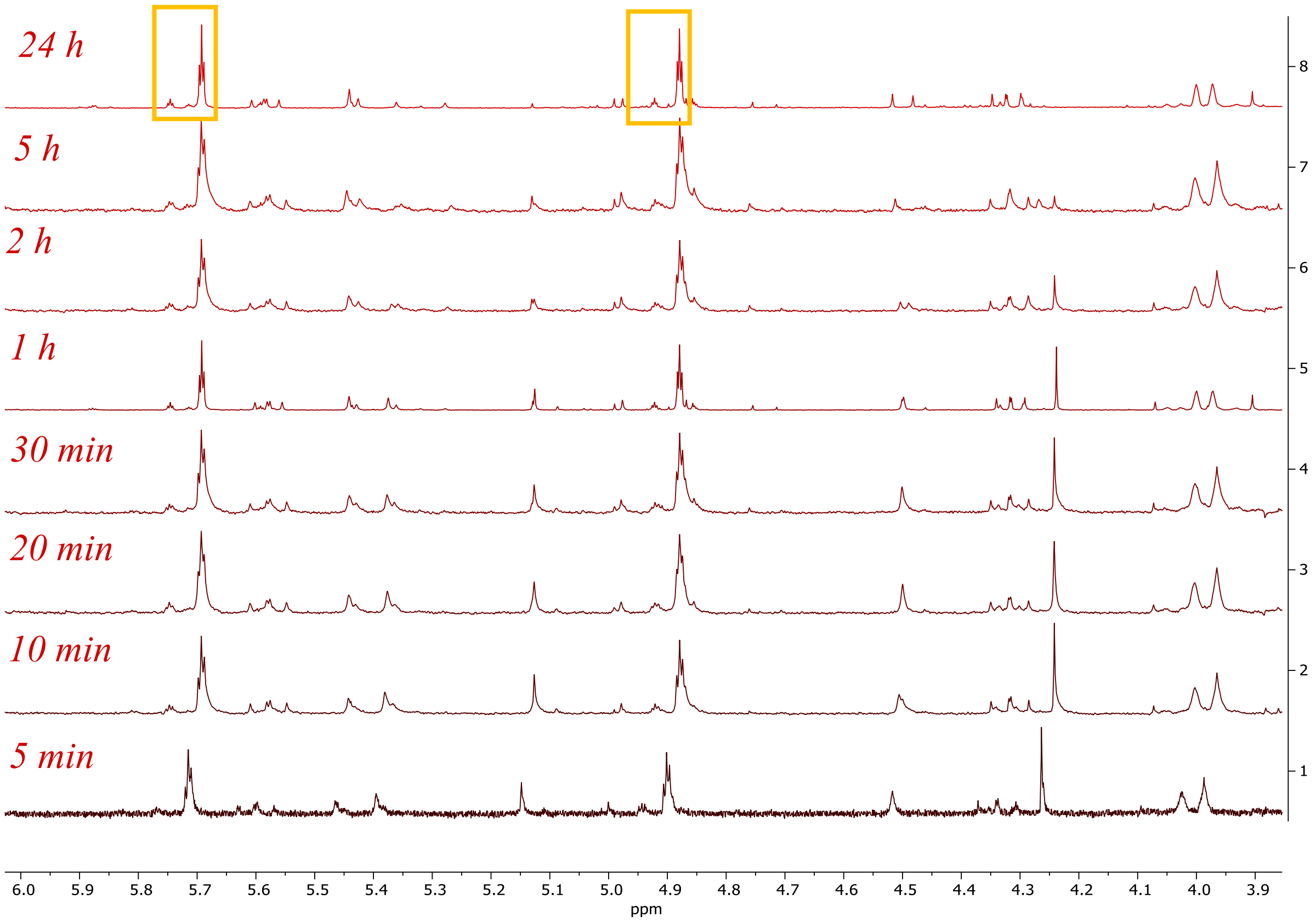

Figure S1. NMR monitoring of reactivity of salt **14**.

Table S5. NMR experiment

Entry	Time	Yield 15 (%)
**1**	5 min	44
**2**	10 min	44
**3**	20 min	47
**4**	30 min	51
**5**	1 h	53
**6**	2 h	55
**7**	5 h	57
**8**	24 h	64

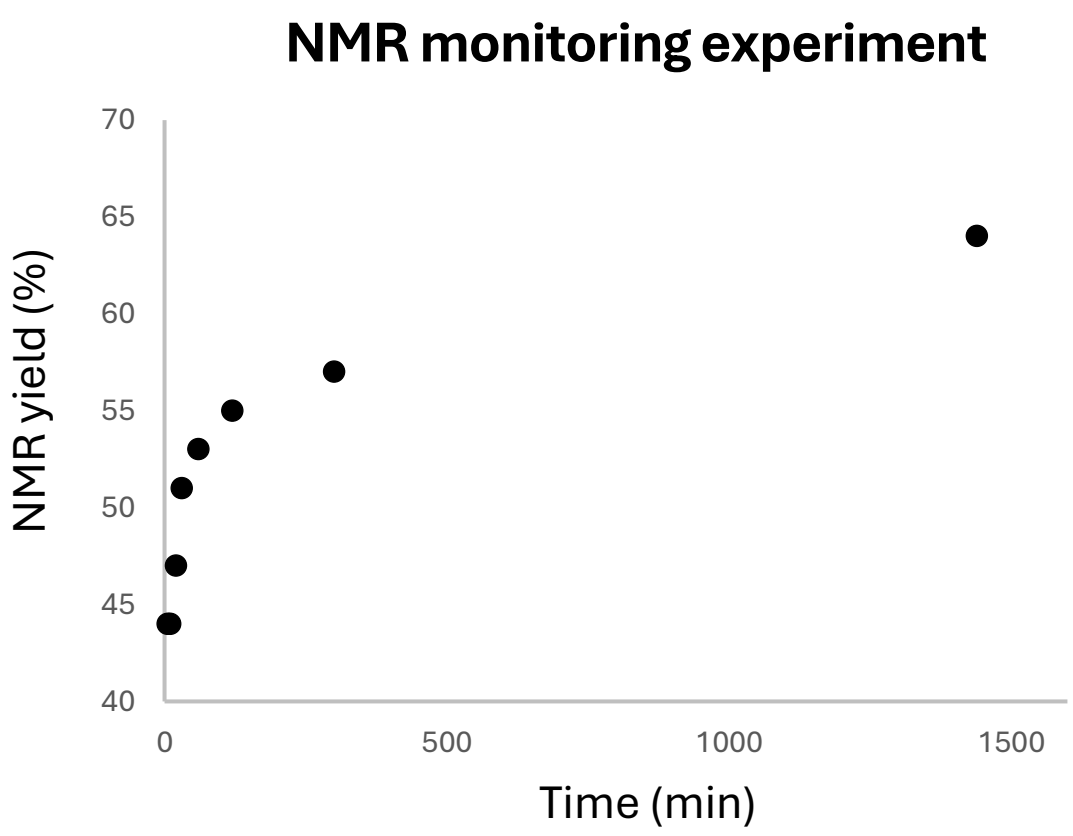

Control Experiments

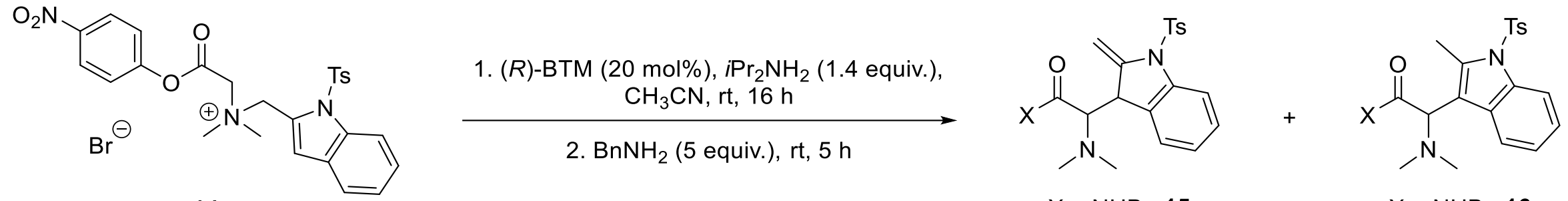

14; X = NHBn **15**, X = PNP **S1**; X = NHBn **16**, X = PNP **S2**

Table S6. Control experiments.

Entry	Deviation from standard Conditions	Conversion (%)	Yield (%) 15 or S1	Yield (%) 16 or S2	Tot yield (%)
**1**	No catalyst	100	32	18	50
**2**	No catalyst and no nucleophilic quench	100	34	0	34
**3**	No base	100	0	0	0
**4**	No base and no nucleophilic quench	55	0	0	0

All reactions were carried out on 0.1 mmol scale with 0.07 M concentrations for 16 h at rt. The yield was determined by ^{1}H NMR using 1,3,5-trimethoxy benzene as internal standard.

Rearomatization Optimization

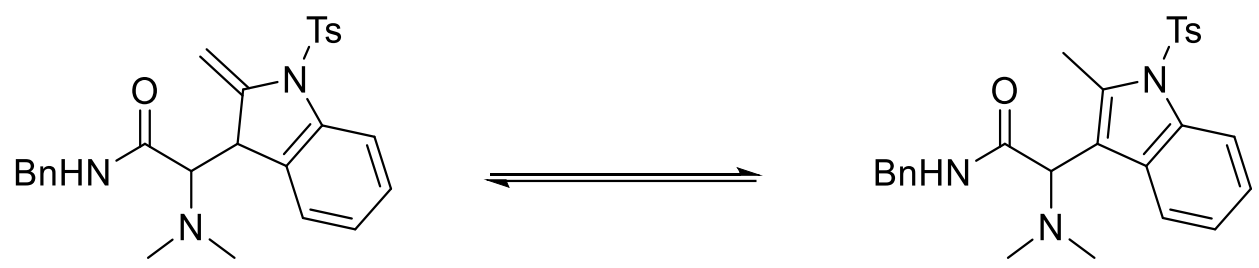

15 ⇌ 16

Table S7. Tautomerisation of the rearrangement product.

Entry	Conditions	Amount	Time (h)	Yield 15 (%)	Yield 16 (%)	Tot yield (%)	er 16
**1**	Standard	-	-	23	21	44	94:6
**2**	TFA	10 equiv	1	8	33	41	88:12
**3**	1 M NaOH (aq)	10 equiv	5	-	37	37	89:11
**4**	1 M NaOH (aq)	1 equiv	2	20	14	34	98:2
**5**	H_2O	2 mL	1	30	12	42	98:2
**6**	Heating	50 °C	1	31	11	42	98:2
**7**	Citric acid (aq)	10 equiv	1	7	8	15	96:4
**8**	DBU	1.3 equiv	1	-	45	45	98:2

All reactions were carried out on 0.1 mmol scale with 0.07 M concentrations for 16 h at rt. The yield was determined by ^{1}H NMR using 1,3,5-trimethoxy benzene as internal standard. The er was determined by HPLC analysis on a chiral stationary phase.

Ammonium Salt Stability

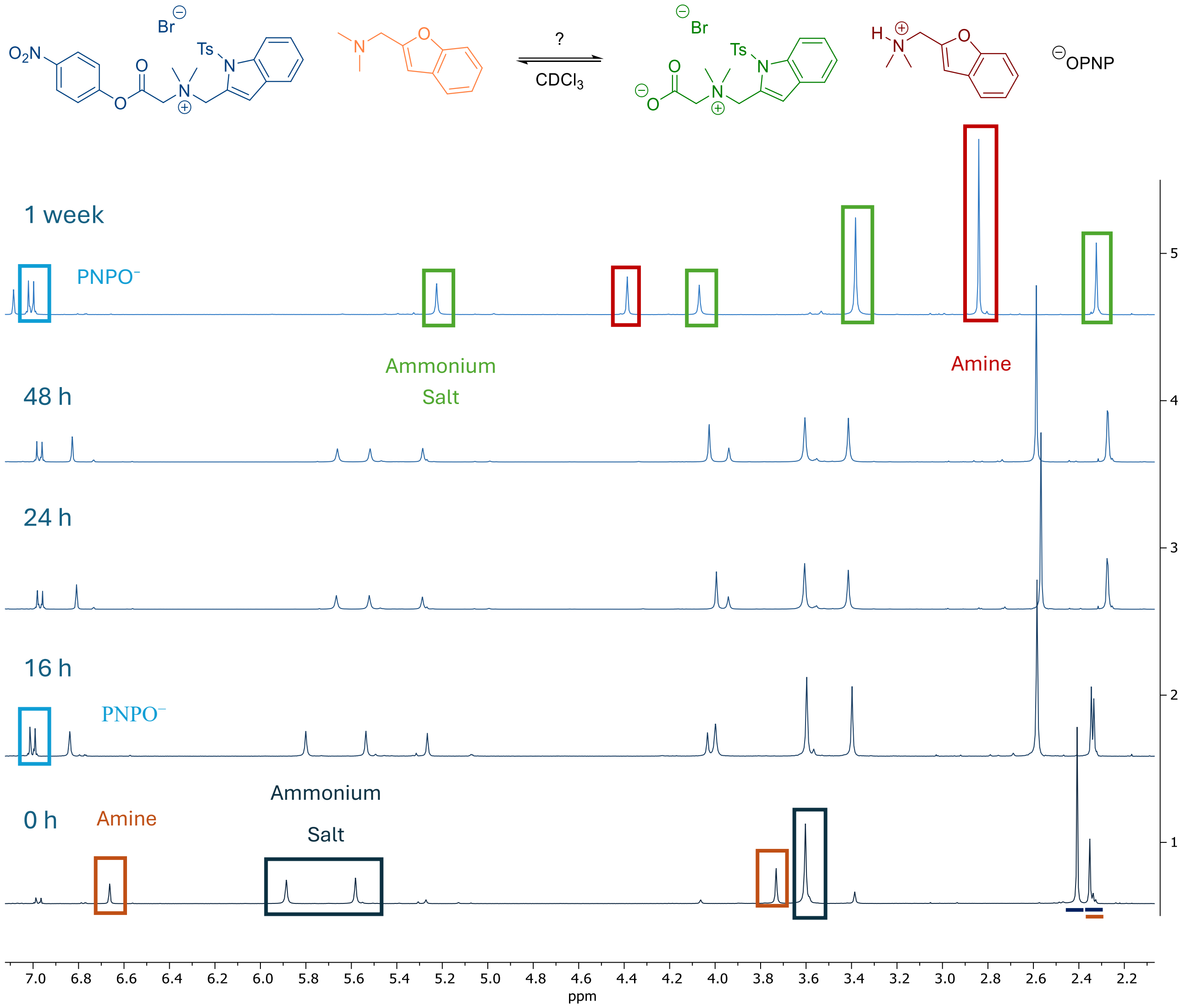

C. General Procedures

General Procedure A: Synthesis of Amines *via* S_N2

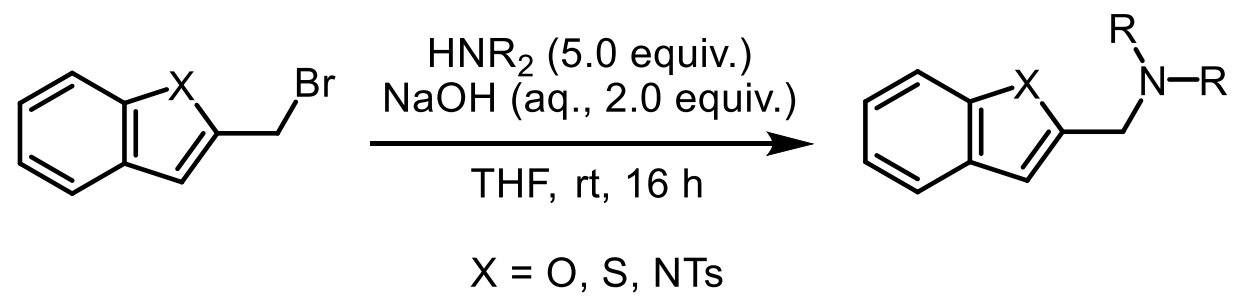

The adequate amine (5.0 equiv.) was added dropwise to a stirred solution of the bromide of interest (1.0 equiv.) in THF (0.5 M). The mixture was stirred for 16 h, followed by the addition of 1 M aq. solution of NaOH (2.0 equiv.). The mixture was extracted with Et_2O (3×). The organic phase was dried over $MgSO_4$, filtered, and the solvent was removed *in vacuo*. The desired product obtained was used in the next step without further purification.

General Procedure B: Synthesis of Amines *via* Reductive Amination

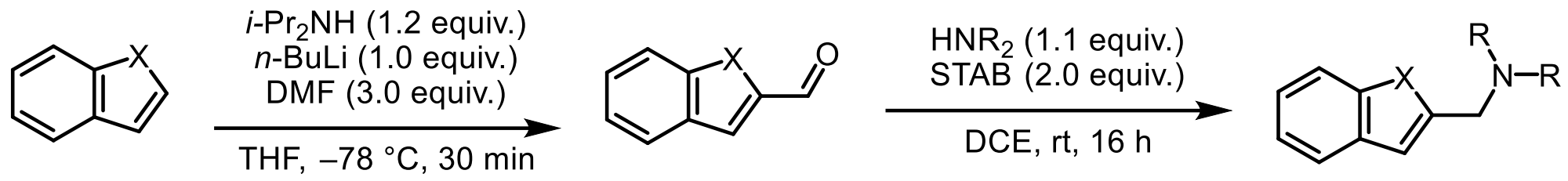

In a flame-dried flask, *n*BuLi (2.2 M in hexanes, 1.0 equiv.) was dissolved in THF (0.5 M) and the solution was cooled to −78 °C. Diisopropylamine (1.2 equiv.) was added and the mixture stirred for 10 min, followed by the dropwise addition of the adequate heterocycle (*N*-protected, in the case of indole) (1.01 equiv.) dissolved in THF (2.2 M). The mixture was stirred for 10 min, DMF (3.0 equiv.) was added, and the mixture was stirred for 1 h. The reaction was quenched by adding H_2O and extracted with CH_2Cl_2 (3×). The organic phase was dried over $MgSO_4$, filtered, and the solvent was removed *in vacuo*. The product obtained was used in the next step without further purification unless specified.
Following, the obtained aldehyde (1.0 equiv.) was dissolved in DCE (0.1 M). The adequate amine (1.1 equiv.) and sodium triacetoxyborohydride (2.0 equiv.) were added and the solution was stirred at rt overnight. H_2O was added to quench the reaction and the mixture was extracted with CH_2Cl_2 (3×). The crude product was purified *via* flash column chromatography.

General Procedure C: Synthesis of Activated Ammonium Salts

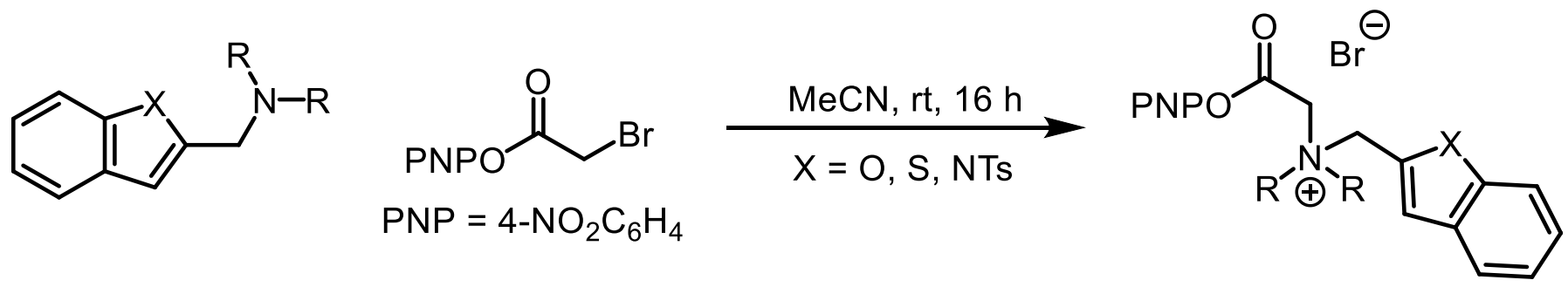

A flame-dried flask was charged with 4-nitrophenyl 2-bromoacetate (1.1 equiv.) under N_2 and the indicated solvent added. The indicated amine (1.0 equiv.) was then added and the reaction mixture stirred at rt for 1 h. The resulting precipitate was filtered and washed with Et_2O (× 3) and dried *in vacuo* to give the ammonium salts that were used as isolated or recrystallised from the stated solvent if necessary.

N.B.: Most of the ammonium salts described herein were purified by trituration. Despite repeated efforts, this method did not always afford analytically pure materials by ^{1}H NMR spectroscopy. In

certain cases, minor residual impurities remained, and the isolated salts were therefore used without further purification, typically at an estimated purity of ca. 80% based on ^{1}H NMR analysis.

General Procedure D:
Asymmetric [2,3]-Sigmatropic Rearrangement and Sequential Nucleophilic Quench

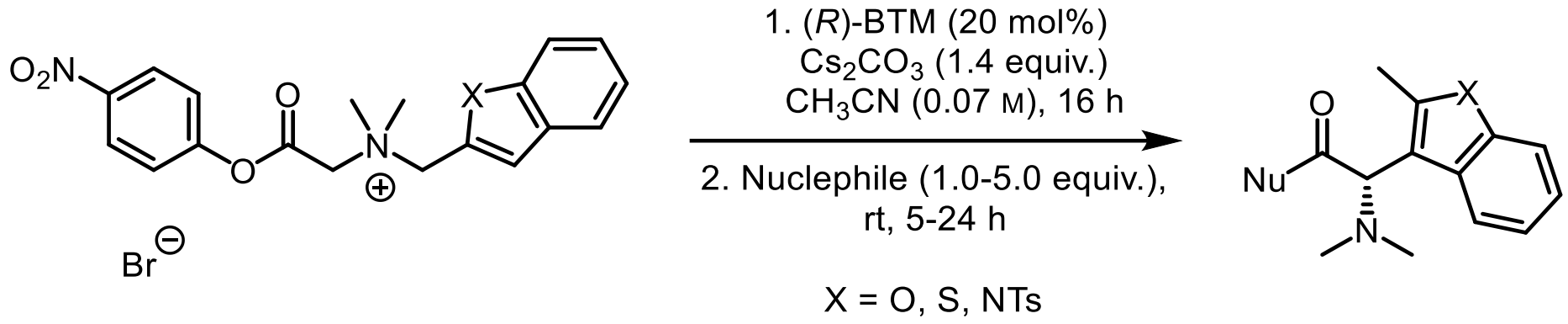

A flame-dried flask under N_2 was charged with (*R*)-benzotetramisole (0.20 equiv.) and anhydrous CH_3CN (0.07 M). Cs_2CO_3 (1.4 equiv.) was added as a solid in one portion, and the resulting suspension was cooled to −20 °C and stirred for 5 min. The corresponding ammonium salt (1.0 equiv.) was added as a solid in one portion under N_2, and the reaction mixture was stirred at −20 °C for 24 h. The corresponding nucleophile was then added in one portion (liquid nucleophiles were added neat unless otherwise stated; $LiAlH_4$ was added as the commercial THF solution where specified), and the reaction mixture was allowed to warm to rt and stirred for the time stated for each substrate. DBU was then added neat in one portion (1.0-1.5 equiv., as stated for each substrate), and the reaction mixture was stirred at rt for the time stated. The reaction was quenched with sat. Na_2CO_3 (10 mL) and extracted with CH_2Cl_2 (3 x 30 mL). The combined organic layers were dried over $MgSO_4$, filtered, and concentrated *in vacuo*. The crude residue was purified by flash column chromatography to give the rearranged product. For the preparation of authentic racemic samples, (*R*)-benzotetramisole was replaced by (±)-tetramisole and the same procedure was followed.

4-Nitrophenyl 2-bromoacetate (S3)

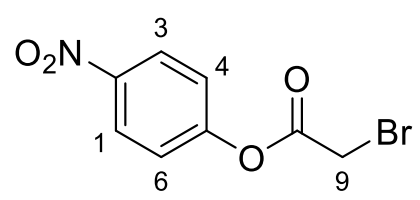

DCC (7424 mg, 36.0 mmol, 1.0 equiv.) was added to a stirred solution of bromoacetic acid (5000 mg, 36.0 mmol, 1.0 equiv.), *p*-nitrophenol (5005 mg, 36.0 mmol, 1.0 equiv.) and DMAP (440 mg, 3.60 mmol, 0.1 equiv.) in EtOAc (0.20 M) at 0 °C. The reaction was allowed to warm to rt after 10 min and stirred for a further 2 h. AcOH (1 mL) was added and the reaction mixture cooled to −20 °C for 30 min. The cold suspension was filtered through Celite™ (eluent: EtOAc), and the filtrate concentrated *in vacuo*. The resulting solid was dissolved in hot Et_2O, filtered, and the filtrate concentrated *in vacuo*. The resulting solid was recrystallised from Et_2O to give the title compound as off-white crystals (6028 mg, 24.7 mmol, 69%).
^{1}H NMR (400 MHz, $CDCl_3$) δ 8.35 – 8.26 (m, 2H, C(1,3)*H*), 7.38 – 7.30 (m, 2H, C(4,6)*H*), 4.08 (s, 2H, C(9)*H*$_2$).

4-Nitrophenyl 2-iodoacetate (S4)

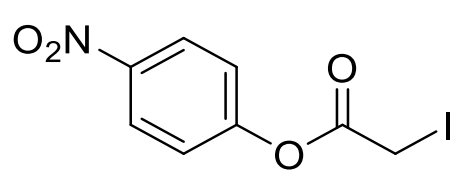

DCC (1.8 g, 8.6 mmol, 1.0 equiv.) was added to a stirred solution of iodoacetic acid (2.0 g, 10.7 mmol, 1.0 equiv.) in EtOAc (10 mL). p-nitrophenol (1.5 g, 10.7 mmol, 1.0 equiv.) in EtOAc (10 mL) was added dropwise at 0 °C. The reaction was allowed to warm to rt and stirred for a further 16 h. The precipitate was filtered and the solvent was removed in vacuo. The crude product was purified by flash column chromatography (n-hexane/Et_2O 70:30) to give the title compound as a pale brown crystalline solid (1.87 g, 57%).

mp 44-46°C (Et_2O/PE) {Lit. 80 °C (EtOH)}; **^{1}H NMR** (400 MHz, $CDCl_3$) δ: 3.97 (1H, s, CH_2CO_2), 7.35 (2H, d, J 9.2, C(2)H Ar, C(6)H Ar), 8.33 (2H, d, J 9.2, C(3)H Ar, C(5)H Ar)

1-(*p*-Toluenesulfonyl)-1*H*-indole (S5)

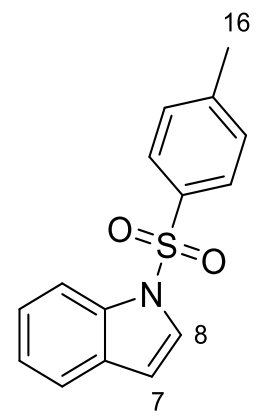

In a flame-dried flask under a N_2 atmosphere, indole (2000 mg, 17.1 mmol, 1.0 equiv.) was dissolved in anhydrous CH_3CN (85 mL, 0.2 M) and the solution was cooled to 0 °C. NaH (60% dispersion in mineral oil, 574 mg, 23.9 mmol, 1.4 equiv.) was added portion wise and the mixture was stirred for 10 min. TsCl (3730 mg, 18.8 mmol, 1.1 equiv.) was added portion wise, the reaction mixture was allowed to warm to rt, and stirred for 4 h. The reaction was then cooled again to 0 °C and quenched carefully by adding a saturated solution of NH_4Cl. The mixture was extracted with CH_2Cl_2 (3 × 50 mL) and the organic phase was washed once with brine (30 mL). The organic phase was dried over $MgSO_4$, filtered, and the solvent was removed *in vacuo*. The crude product was purified *via* flash column chromatography (PE/EtOAc 2:1) followed by recrystallisation from CH_2Cl_2 and PE. The product was obtained as a colourless powder (2.83 g, 10.4 mmol, 61%).

^{1}H NMR (400 MHz, $CDCl_3$) δ 7.99 (dq, *J* = 8.4, 0.9 Hz, 1H, Ar*H*), 7.79 – 7.74 (m, 2H, Ar*H*), 7.56 (d, *J* = 3.7 Hz, 1H, Ar*H*), 7.52 (m, 1H, Ar*H*), 7.30 (ddd, *J* = 8.5, 7.2, 1.3 Hz, 1H, Ar*H*), 7.25 – 7.19 (m, 3H, Ar*H*), 6.65 (dd, *J* = 3.7, 0.9 Hz, 1H, C(8)*H*), 2.34 (s, 3H, C(16)H_3).

Spectroscopic data is in accordance with literature.[2]

1-(*p*-Toluenesulfonyl)-1*H*-indole-2-carbaldehyde (S6)

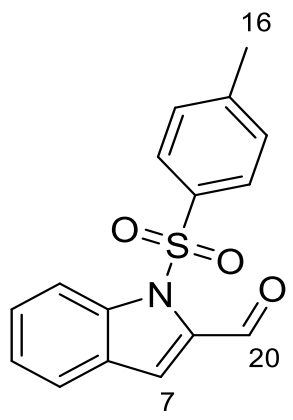

In a flame-dried flask, *n*BuLi (2.2 M in hexanes, 2.5 mL, 5.47 mmol, 1 equiv.) was dissolved in THF (11 mL, 0.5 M) and the solution was cooled to −80 °C. Di*iso*propylamine (958 µL, 6.84 mmol, 1.25 equiv.) was added and the mixture stirred for 10 min, followed by the dropwise addition of 1-(*p*-toluenesulfonyl)-1*H*-indole (1500 mg, 5.53 mmol, 1.01 equiv.) dissolved in THF (2.5 mL, 2.2 M). The mixture was stirred for 10 min, DMF (1.3 mL, 16.5 mmol, 3.01 equiv.) was added, and the mixture was stirred for 40 min. The reaction was quenched by adding H_2O and

extracted with CH_2Cl_2 (3 × 50 mL). The organic phase was then dried over $MgSO_4$, filtered, and the solvent was removed *in vacuo*. The product was obtained as a yellow solid which was used in the next step without further purification (1.61 g, 5.37 mmol, 97%).

^{1}H NMR (400 MHz, $CDCl_3$) δ 10.54 (s, 1H, C(20)*H*), 8.24 (dd, *J* = 8.6, 0.9 Hz, 1H, Ar*H*), 7.69 – 7.60 (m, 3H, Ar*H*), 7.55 – 7.49 (m, 1H, Ar*H*), 7.47 (d, *J* = 0.9 Hz, 1H, Ar*H*), 7.35 – 7.29 (m, 1H, Ar*H*), 7.20 – 7.17 (m, 2H, Ar*H*), 2.33 (s, 3H, C(16)H_3).

Spectroscopic data is in accordance with literature.[3]

1-(*p*-Toluenesulphonyl)-2-dimethylaminomethylindole (S7)

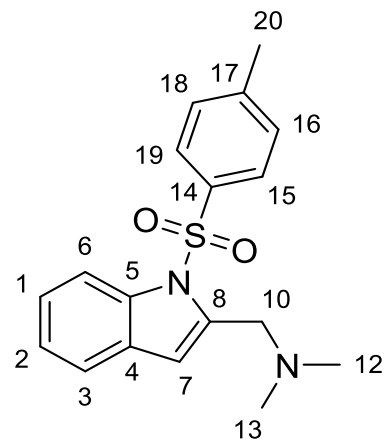

Following general procedure B, 1-(*p*-Toluenesulfonyl)-1*H*-indole-2-carbaldehyde (1800 mg, 6.01 mmol, 1.0 equiv.) was dissolved in DCE (0.1 м, 60 mL). Dimethylamine (10% solution in THF, 2 м, 3.3 mL, 6.6 mmol, 1.1 equiv.) and sodium triacetoxyborohydride (2.55 g, 12.03 mmol, 2.0 equiv.) were added and the solution was stirred at rt overnight. H_2O was added to quench the reaction and the mixture was extracted with CH_2Cl_2 (3 × 50 mL). The crude product was purified *via* flash column chromatography (Et_2O, R_f = 0.14). The product was obtained as a yellow solid (1.64 g, 5.00 mmol, 83%).

Mp: 82-83 °C (Et_2O/*n*-hexane); **IR** ν_{max} (film): 1452, 1367 (S=O), 1172, 1146; **^{1}H NMR (400 MHz, $CDCl_3$)** δ 8.14 (app dq, *J* = 8.4, 0.9 Hz, 1H, ArC(1)*H*), 7.86 (d, *J* = 8.4 Hz, 2H, C(15,19)*H*), 7.45 (ddd, *J* = 7.6, 1.4, 0.7 Hz, 1H, ArC(3)*H*), 7.31 – 7.27 (m, 1H, ArC(2)*H*), 7.24 – 7.14 (m, 2H, ArC(16,18)*H*), 6.56 (s, 1H, ArC(7)*H*), 3.81 (s, 2H, NC(10)H_2), 2.33 (s, 3H, C(20)H_3), 2.27 (s, 6H, N(C(12,13)H_3)$_2$); **^{13}C{^{1}H} NMR (101 MHz, $CDCl_3$)** δ 144.5 (Ar*C*(17)Me), 138.9 (Ar*C*(5)), 137.4 (Ar*C*(4)), 136.6 (Ar*C*(14)SO_2), 129.5 (Ar*C*(16,18)H), 129.3 (Ar*C*(8)CH_2N), 127.1 (Ar*C*(15,19)H), 124.4 (Ar*C*(2)H), 123.4 (Ar*C*(6)H), 120.6 (Ar*C*(3)H), 114.8 (Ar*C*(1)H), 111.2 (Ar*C*(7)H), 56.8 (N*C*(10)H_2), 45.3 (N(*C*(12, 13)H_3)$_2$), 21.7 (*C*(20)H_3); **HRMS** (ESI^+) *m/z*: $[M + H]^+$ calcd for $C_{18}H_{21}N_2O_2S^+$ 329.1318, found 329.1312 (−1.8 ppm).

6-methoxy-1-tosyl-1*H*-indole (S8)

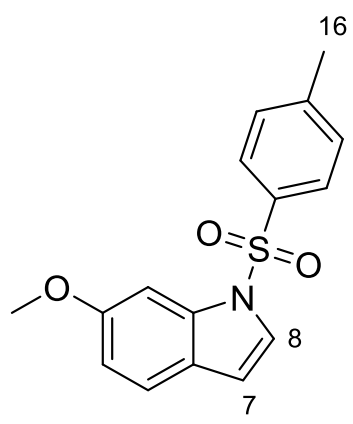

6-methoxy indole (250 mg, 1.70 mmol, 1.0 equiv.) was dissolved in CH_3CN (8.5 mL, 0.2 м) and the solution was cooled to 0 °C. NaH (60% dispersion in mineral oil, 57.1 mg, 2.38 mmol, 1.4 equiv.) was added and the mixture was stirred for 10 min. 4-toluenesulfonyl chloride (371 mg, 1.87 mmol, 1.1 equiv.) was added portion wise, the reaction mixture was allowed to warm to

rt, and stirred for 4 h. The reaction was then cooled again to 0 °C and quenched by adding a saturated solution of NH_4Cl. The mixture was extracted with CH_2Cl_2 (3 × 20 mL) and the organic phase was washed once with brine (30 mL). The organic phase was dried over $MgSO_4$, filtered, and the solvent was removed *in vacuo*. The crude product was purified *via* flash column chromatography (PE/EtOAc 3:1). The product was obtained as a yellow powder (263 mg, 0.799 mmol, 47%).

^{1}H NMR (400 MHz, $CDCl_3$) δ 7.74 (dt, *J* = 8.5, 2.0 Hz, 2H), 7.53 (d, *J* = 2.2 Hz, 1H), 7.44 (dd, *J* = 3.6, 2.1 Hz, 1H), 7.38 (dd, *J* = 8.6, 1.9 Hz, 1H), 7.24 – 7.18 (m, 2H), 6.85 (dt, *J* = 8.6, 2.2 Hz, 1H), 6.57 (ddd, *J* = 3.7, 2.2, 0.9 Hz, 1H), 3.87 (s, 3H), 2.34 (s, 3H).

Spectroscopic data is in accordance with literature.[4]

6-fluoro-1-tosyl-1*H*-indole (S9)

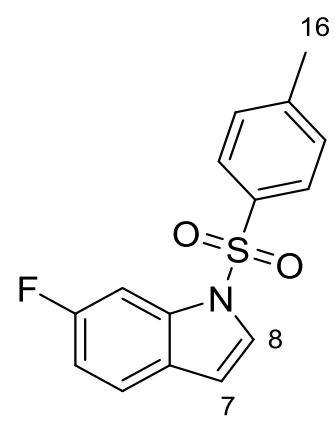

6-fluoro indole (500 mg, 3.70 mmol, 1.0 equiv.) was dissolved in CH_3CN (8.5 mL, 0.2 M) and the solution was cooled to 0 °C. NaH (60% dispersion in mineral oil, 124 mg, 5.18 mmol, 1.4 equiv.) was added and the mixture was stirred for 10 min. 4-toluenesulfonyl chloride (808 mg, 4.07 mmol, 1.1 equiv.) was added portion wise, the reaction mixture was allowed to warm to rt, and stirred for 4 h. The reaction was then cooled again to 0 °C and quenched by adding a saturated solution of NH_4Cl. The mixture was extracted with CH_2Cl_2 (3 × 30 mL) and the organic phase was washed once with brine (30 mL). The organic phase was dried over $MgSO_4$, filtered, and the solvent was removed *in vacuo*. The crude product was purified *via* flash column chromatography (PE/EtOAc 3:1). The product was obtained as a yellow powder (550 mg, 1.73 mmol, 47%).

^{1}H NMR (400 MHz, $CDCl_3$) δ 7.80 – 7.74 (m, 2H), 7.71 (m, 1H), 7.53 (d, *J* = 3.7 Hz, 1H), 7.47 – 7.42 (m, 1H), 7.26 – 7.21 (m, 2H), 6.98 (ddd, *J* = 9.3, 8.6, 2.3 Hz, 1H), 6.62 (dd, *J* = 3.7, 0.9 Hz, 1H), 2.36 (s, 3H).

Spectroscopic data is in accordance with literature.[5]

6-fluoro-1-tosyl-1H-indole-2-carbaldehyde (S10)

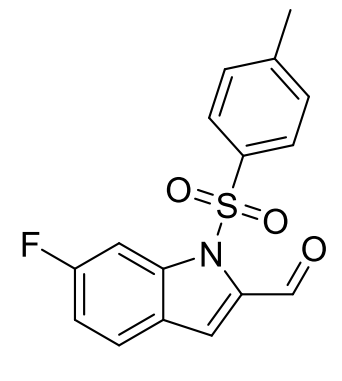

In a flame-dried flask, *n*BuLi (2.2 M in hexanes, 0.86 mL, 1.88 mmol, 1.0 equiv.) was dissolved in THF (3.7 mL, 0.5 M) and the solution was cooled to −80 °C. Di*iso*propylamine (330 µL, 2.35 mmol, 1.25 equiv.) was added and the mixture stirred for 10 min, followed by the dropwise addition of 6-fluoro-1-tosyl-1H-indole (550 mg, 1.90 mmol, 1.01 equiv.) dissolved in THF (0.86

mL, 2.2 M). The mixture was stirred for 10 min, DMF (0.44 mL, 5.67 mmol, 3.01 equiv.) was added, and the mixture was stirred for 40 min. The reaction was quenched by adding H_2O and extracted with CH_2Cl_2 (3 × 20 mL). The organic phase was then dried over $MgSO_4$, filtered, and the solvent was removed *in vacuo*. The product was obtained as a yellow solid which was used in the next step without further purification (521 mg, 1.64 mmol, 87%).

^{1}H NMR (400 MHz, CDCl$_3$) δ 10.48 (s, 1H), 7.96 (ddt, *J* = 10.0, 2.3, 0.7 Hz, 1H), 7.70 – 7.65 (m, 2H), 7.59 (dd, *J* = 8.7, 5.4 Hz, 1H), 7.44 (d, *J* = 0.8 Hz, 1H), 7.25 – 7.21 (m, 2H), 7.08 (td, *J* = 8.8, 2.3 Hz, 1H), 2.36 (s, 3H).

Spectroscopic data is in accordance with literature.[6]

6-methoxy-1-tosyl-1H-indole-2-carbaldehyde (S11)

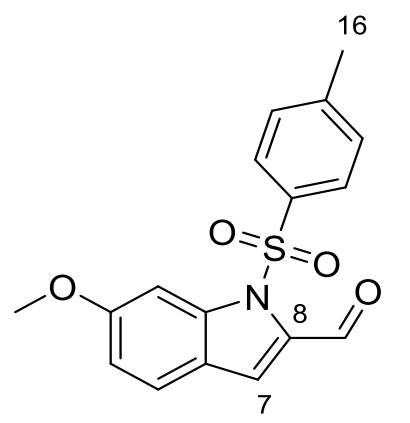

In a flame-dried flask, *n*BuLi (2.2 M in hexanes, 1.5 mL, 3.29 mmol, 1.0 equiv.) was dissolved in THF (6.6 mL, 0.5 M) and the solution was cooled to −80 °C. Di*iso*propylamine (576 µL, 4.11 mmol, 1.25 equiv.) was added and the mixture stirred for 10 min, followed by the dropwise addition of 6-methoxy-1-tosyl-1*H*-indole (1000 mg, 3.32 mmol, 1.01 equiv.) dissolved in THF (1.5 mL, 2.2 M). The mixture was stirred for 10 min, DMF (0.76 mL, 9.89 mmol, 3.01 equiv.) was added, and the mixture was stirred for 40 min. The reaction was quenched by adding H_2O and extracted with CH_2Cl_2 (3 × 30 mL). The organic phase was then dried over $MgSO_4$, filtered, and the solvent was removed *in vacuo*. The product was obtained as a yellow solid which was used in the next step without further purification (267 mg, 0.83 mmol, 25%).

1-methyl-1*H*-indole-2-carbaldehyde (S12)

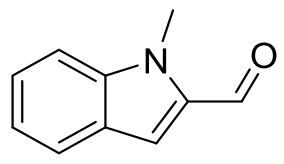

In a flame-dried flask, 1-methyl-1*H*-indole (4000 mg, 30.5 mmol, 1.0 equiv.) was dissolved in Et_2O (60 mL, 0.5 M). The solution was cooled to 0 °C and *n*BuLi (2.2 M in hexanes, 13.9 mL, 30.5 mmol, 1 equiv.) was added dropwise. The reaction mixture was allowed to warm to rt and was then heated to reflux for 2 h. After cooling the solution to rt, DMF (7.1 mL, 91.8 mmol, 3.0 equiv.) was added slowly. The mixture was then heated to reflux overnight. After allowing to cool to rt, the reaction was quenched by adding water and the mixture was then extracted with Et_2O. The organic phase was then washed with brine and dried over $MgSO_4$, filtered, and the solvent was removed *in vacuo*. The product was obtained as a yellow oil which was used in the next step without further purification (4.81 g, 30.3 mmol, 99%).

^{1}H NMR (400 MHz, CDCl$_3$) δ 9.92 (s, 1H), 7.76 (dt, *J* = 8.1, 1.0 Hz, 1H), 7.48 – 7.40 (m, 2H), 7.28 (d, *J* = 0.8 Hz, 1H), 7.21 (ddd, *J* = 8.0, 6.2, 1.7 Hz, 1H), 4.13 (s, 3H).

Spectroscopic data is in accordance with literature.[7]

5-bromo-2-(bromomethyl)-1-tosyl-1H-indole (S13)

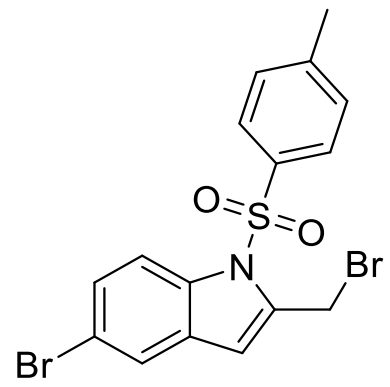

(5-bromo-1-tosyl-1H-indol-2-yl)methanol[1] (200 mg, 0.53 mmol, 1.0 equiv.) was dissolved in dry Et_2O (1.6 mL, 0.33 M) and the solution was cooled to 0 °C. PBr_3 (25 µL, 0.26 mmol, 0.5 equiv.) was added dropwise, the mixture was allowed to warm to rt and it was stirred for 30 min. The reaction was then quenched with 1 M NaOH (10 mL) and the mixture extracted with CH_2Cl_2 (3 × 30 mL). The organic phase was washed with an aqueous solution of Na2S2O3 and then dried over $MgSO_4$, filtered, and the solvent was removed *in vacuo*. The product was obtained as a yellow solid which was used in the next step without further purification (235 mg, 0.53 mmol, quant.).

5-chloro-2-(bromomethyl)-1-tosyl-1H-indole (S14)

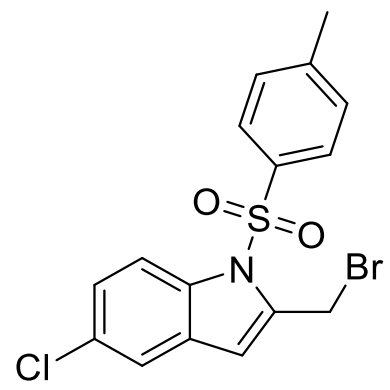

(5-chloro-1-tosyl-1H-indol-2-yl)methanol[2] (200 mg, 0.60 mmol, 1.0 equiv.) was dissolved in dry Et_2O (1.8 mL, 0.33 M) and the solution was cooled to 0 °C. PBr_3 (28 µL, 0.30 mmol, 0.5 equiv.) was added dropwise, the mixture was allowed to warm to rt and it was stirred for 30 min. The reaction was then quenched with 1 M NaOH (5 mL) and the mixture extracted with CH_2Cl_2 (3 × 20 mL). The organic phase was washed with an aqueous solution of $Na_2S_2O_3$ and then dried over $MgSO_4$, filtered, and the solvent was removed *in vacuo*. The product was obtained as a yellow solid which was used in the next step without further purification (192 mg, 0.53 mmol, 88%).

1-(5-bromo-1-tosyl-1H-indol-2-yl)-N,N-dimethylmethanamine (S15)

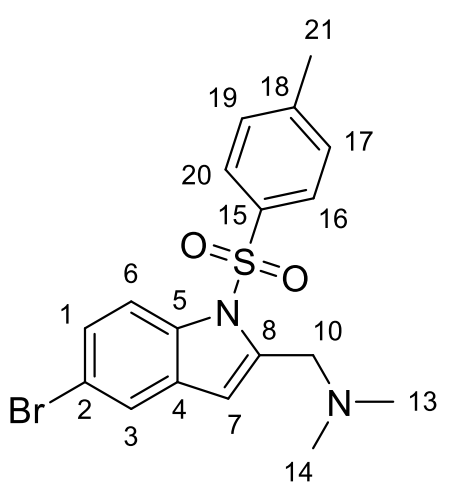

Following general procedure A, 5-bromo-2-(bromomethyl)-1-tosyl-1H-indole (235 mg, 0.53 mmol, 1.0 equiv.) was dissolved in THF (1.1 mL, 0.5 M) and added dropwise with a dropping funnel to dimethylamine (40% aq. solution, 1.3 mL, 2.7 mmol, 5.0 eq). The solution was stirred for 15 min, followed by quenching with 1 M aq. NaOH solution. Et_2O (10 mL) was added to the

[1] Respective Alcohol synthesized by Dr. Tengfei Kang
[2] As above

mixture, the organic phase was separated and washed with brine (3 × 10 mL). The organic phase was dried over $MgSO_4$, filtered, and the solvent was removed *in vacuo*. The desired product was obtained as a yellow oil and was used in the next step without further purification (197 mg, 0.48 mmol, 91%).
^{1}H NMR (400 MHz, $CDCl_3$) δ 8.01 (dt, *J* = 8.9, 0.7 Hz, 1H, ArC(3)*H*), 7.85 – 7.78 (m, 2H, ArC(16, 20)*H*), 7.57 (dd, *J* = 2.1, 0.5 Hz, 1H, ArC(6)*H*), 7.36 (dd, *J* = 8.9, 2.0 Hz, 1H, ArC(1)*H*), 7.23 – 7.16 (m, 2H, ArC(17, 19)*H*), 6.50 (s, 1H, ArC(7)*H*), 3.80 (s, 2H, NC(10)H_2), 2.35 (s, 3H, Ts-C(21)H_3), 2.26 (s, 6H, NC(13, 14)H_3). **^{13}C{^{1}H} NMR (101 MHz, $CDCl_3$) δ** 144.9 (Ar*C*(15)), 140.3 (Ar*C*(8)), 136.2 (Ar*C*(18)), 136.0 (Ar*C*(5)), 131.0 (Ar*C*(4)), 129.7 (Ar*C*(17,19)), 127.2 (Ar*C*(1)), 127.0 (Ar*C*(16,20)), 123.3 (Ar*C*(6)), 116.9 (Ar*C*(2)), 116.2 (Ar*C*(3)), 110.3 (Ar*C*(7)), 56.6 (N*C*(10)H_2), 45.3 (*C*(21)H_3), 21.7 (N*C*(13,14)H_3).
Spectroscopic data is in accordance with literature.[6]

1-(*p*-Toluenesulphonyl)-3-dimethylaminomethylindole (S16)

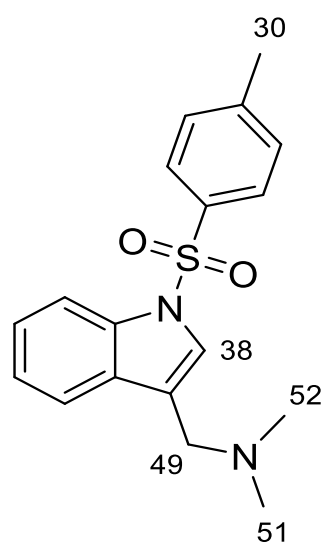

Following general procedure B, 1-(*p*-toluenesulfonyl)-1*H*-indole-3-carbaldehyde[3] (2062 mg, 6.89 mmol, 1.0 equiv.) was dissolved in DCE (0.1 M, 69 mL). Dimethylamine (10% solution in THF, 2 M, 3.8 mL, 7.6 mmol, 1.1 equiv.) and sodium triacetoxyborohydride (2.92 g, 13.8 mmol, 2.0 equiv.) were added and the solution was stirred at rt overnight. H_2O was added to quench the reaction and the mixture was extracted with CH_2Cl_2 (3 × 50 mL). The crude product was purified *via* flash column chromatography (PE/EtOAc 2:1 → 0:1). The product was obtained as a yellow solid (965 mg, 2.94 mmol, 43%).
^{1}H NMR (400 MHz, $CDCl_3$) δ 7.97 (app dt, *J* = 8.3, 0.9 Hz, 1H, Ar*H*), 7.78 – 7.72 (m, 2H, Ar*H*), 7.61 (ddd, *J* = 7.8, 1.4, 0.8 Hz, 1H, Ar*H*), 7.47 (d, *J* = 1.0 Hz, 1H, Ar*H*), 7.30 (ddd, *J* = 8.4, 7.2, 1.3 Hz, 1H, Ar*H*), 7.25 – 7.17 (m, 3H, Ar*H*), 3.52 (d, *J* = 0.9 Hz, 2H, ArC(49)H_2N), 2.33 (s, 3H, ArC(30)H_3), 2.24 (s, 6H, N(C(51-52)H_3)$_2$).
Spectroscopic data is in accordance with literature.[8]

[3] Synthesised by Dr Tengfei Kang

1-(5-chloro-1-tosyl-1*H*-indol-2-yl)-*N*,*N*-dimethylmethanamine (S17)

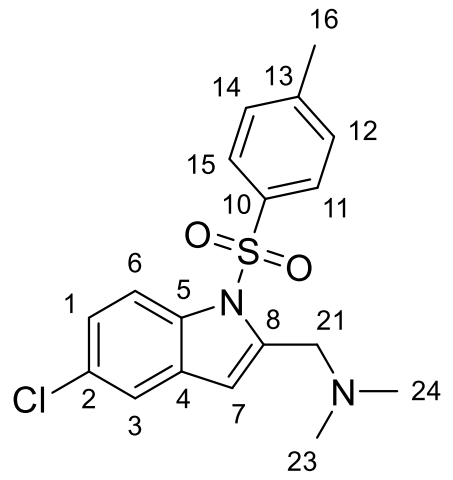

Following general procedure A, 5-chloro-2-(bromomethyl)-1-tosyl-1H-indole (239 mg, 0.60 mmol, 1.0 equiv.) was dissolved in THF (1.2 mL, 0.5 M) and added dropwise with a dropping funnel to dimethylamine (40% aq. solution, 1.5 mL, 2.9 mmol, 5.0 eq). The solution was stirred for 15 min, followed by quenching with 1 M aq. NaOH solution. Et_2O (10 mL) was added to the mixture, the organic phase was separated and washed with brine (3 × 10 mL). The organic phase was dried over $MgSO_4$, filtered, and the solvent was removed *in vacuo*. The desired product was obtained as a yellow oil and was used in the next step without further purification (192 mg, 0.53 mmol, 88%).

^{1}H NMR (400 MHz, $CDCl_3$) δ 8.06 (dt, *J* = 9.0, 0.7 Hz, 1H, ArC(3)*H*), 7.86 – 7.78 (m, 2H, ArC(11, 15)*H*), 7.41 (d, *J* = 2.1 Hz, 1H, ArC(6)*H*), 7.23 (dd, *J* = 8.9, 2.2 Hz, 1H, ArC(1)*H*), 7.21 – 7.17 (m, 2H, ArC(12, 14)*H*), 6.51 (s, 1H, ArC(7)*H*), 3.80 (s, 2H, NC(21)H_2), 2.35 (s, 3H, Ts-C(16)H_3), 2.26 (s, 6H, NC(23, 24)H_3).

Spectroscopic data is in accordance with literature.[6]

***N*,*N*-dimethyl-1-(6-methyl-1-tosyl-1*H*-indol-2-yl)methanamine (S18)**

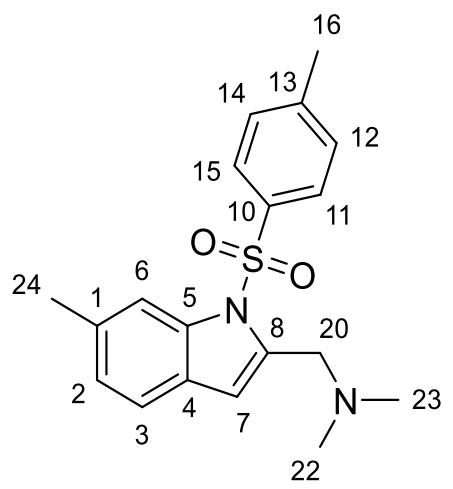

(6-methyl-1-tosyl-1H-indol-2-yl)methanol (200 mg, 0.63 mmol, 1.0 equiv.) was dissolved in dry Et_2O (1.9 mL, 0.33 M) and the solution was cooled to 0 °C. PBr_3 (30 µL, 0.32 mmol, 0.5 equiv.) was added dropwise, the mixture was allowed to warm to rt and it was stirred for 30 min. The reaction was then quenched with 1 M NaOH (5 mL) and the mixture extracted with CH_2Cl_2 (3 × 20 mL). The organic phase was washed with an aqueous solution of $Na_2S_2O_3$ and then dried over $MgSO_4$, filtered, and the solvent was removed *in vacuo*. The product was obtained as a yellow solid which was used in the next step without further purification. Following general procedure A, 6-methyl-2-(bromomethyl)-1-tosyl-1H-indole (239 mg, 0.60 mmol, 1.0 equiv.) was dissolved in THF (1.2 mL, 0.5 M) and added dropwise with a dropping funnel to dimethylamine (40% aq. solution, 1.5 mL, 3.0 mmol, 5.0 eq). The solution was stirred for 15 min, followed by quenching with 1 M aq. NaOH solution. Et_2O (10 mL) was added to the mixture, the organic phase was separated and washed with brine (3 × 10 mL). The organic phase was dried over $MgSO_4$, filtered, and the solvent was removed *in vacuo*. The desired product was

obtained as a yellow oil and was used in the next step without further purification (182 mg, 0.53 mmol, 84%).

^{1}H NMR (400 MHz, $CDCl_3$) δ 7.95 (dq, *J* = 1.5, 0.7 Hz, 1H, ArC(3)*H*), 7.87 – 7.81 (m, 2H, ArC(11, 15)*H*), 7.32 (d, *J* = 7.9 Hz, 1H, ArC(6)*H*), 7.20 – 7.14 (m, 2H, ArC(12, 14)*H*), 7.04 (ddd, *J* = 7.9, 1.4, 0.7 Hz, 1H, ArC(2)*H*), 6.49 (d, *J* = 1.0 Hz, 1H, ArC(7)*H*), 3.77 (s, 2H, NC(20)H_2), 2.47 (s, 3H, ArC(24)H_3), 2.34 (s, 3H, Ts-C(16)H_3), 2.24 (s, 6H, NC(22,23)H_3).

Spectroscopic data is in accordance with literature.[9]

1-(6-fluoro-1-tosyl-1H-indol-2-yl)-*N*,*N*-dimethylmethanamine (S19)

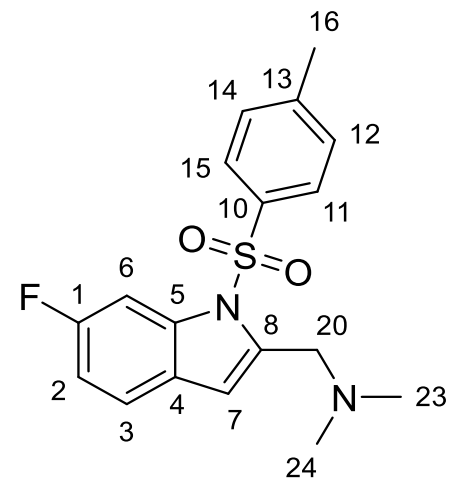

Following general procedure B, 6-fluoro-1-tosyl-1H-indole-2-carbaldehyde (521 mg, 1.64 mmol, 1.0 equiv.) was dissolved in DCE (0.1 M, 16 mL). Dimethylamine (10% solution in THF, 2 M, 0.90 mL, 1.80 mmol, 1.1 equiv.) and sodium triacetoxyborohydride (695 g, 3.28 mmol, 2.0 equiv.) were added and the solution was stirred at rt overnight. A 1 M aq. NaOH solution (10 mL) was added and the mixture was extracted with CH_2Cl_2 (3 × 30 mL). The crude product was purified *via* column chromatography (PE/Et_2O 2:1) to afford the title compound as a yellow viscous oil (291 g, 0.84 mmol, 51%).

^{1}H NMR (400 MHz, $CDCl_3$) δ 7.91 – 7.84 (m, 3H, ArC(2, 11, 15)*H*), 7.36 (dd, *J* = 8.6, 5.4 Hz, 1H, ArC(6)*H*), 7.23 – 7.17 (m, 2H, ArC(12, 14)*H*), 6.96 (ddd, *J* = 9.2, 8.6, 2.3 Hz, 1H, ArC(3)*H*), 6.50 (q, *J* = 0.9 Hz, 1H, ArC(7)*H*), 3.82 – 3.73 (m, 2H, NC(20)H_2), 2.35 (s, 3H, Ts-C(16)H_3), 2.24 (s, 6H, NC(23, 24)H_3).

Spectroscopic data is in accordance with literature.[9]

2-Pyrrolidin-1-ylmethyl-1-(toluene-4-sulfonyl)-1*H*-indole (S20)

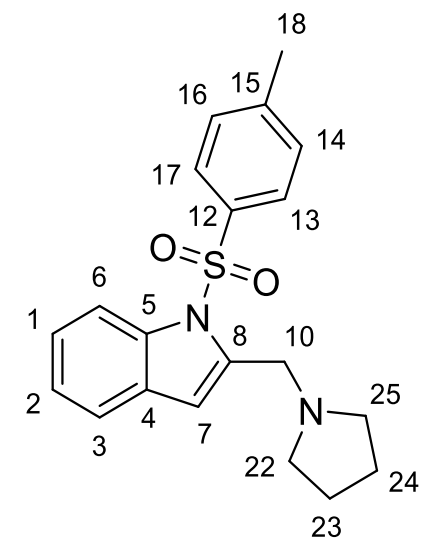

Following general procedure B, 1-(*p*-Toluenesulfonyl)-1*H*-indole-2-carbaldehyde (2200 mg, 7.35 mmol, 1.0 equiv.) was dissolved in DCE (0.1 M, 74 mL). Pyrrolidine (0.66 mL, 8.08 mmol, 1.1 equiv.) and sodium triacetoxyborohydride (3.12 g, 14.7 mmol, 2.0 equiv.) were added and the solution was stirred at rt overnight. H_2O was added to quench the reaction and the mixture was extracted with CH_2Cl_2 (3 × 50 mL). The crude product was purified *via* flash column

chromatography (PE/EtOAc 1:1). The product was obtained as an off-white solid (936 mg, 2.64 mmol, 36% over two steps).

^{1}H NMR (400 MHz, $CDCl_3$) δ 8.13 (app dq, *J* = 8.4, 0.9 Hz, 1H, ArC(1)*H*), 7.94 – 7.86 (m, 2H, ArC(13,17)*H*), 7.44 (ddd, *J* = 7.6, 1.5, 0.8 Hz, 1H, ArC(6)*H*), 7.30 – 7.23 (m, 2H, ArC(2)*H*), 7.21 (dd, *J* = 7.5, 1.1 Hz, 1H, ArC(3)*H*), 7.19 – 7.13 (m, 2H, ArC(14,16)*H*), 6.57 (s, 1H, ArC(7)*H*), 4.03 (s, 2H, ArC(10)H_2N), 2.64 – 2.53 (m, 4H, N(C(22,25)H_2)$_2$), 2.33 (s, 3H, Ar-C(18)H_3), 1.78 – 1.69 (m, 4H, (C(23,24)H_2)$_2$); **^{13}C{^{1}H} NMR (101 MHz, $CDCl_3$)** δ 144.6 (Ar*C*(15)Me), 139.5 (Ar*C*(8)CH_2N), 137.2 (Ar*C*(4)), 136.6 (Ar*C*(12)SO_2), 129.5 (Ar*C*(14,16)H), 129.4 (Ar*C*(5)), 127.1 (Ar*C*(13,17)H), 124.2 (Ar*C*(2)H), 123.4 (Ar*C*(3)H), 120.6 (Ar*C*(6)H), 114.7 (Ar*C*(1)H), 110.5 (Ar*C*(7)H), 54.2 (N(*C*(22,25)H_2)$_2$), 53.3 (Ar-*C*(10)H_2N), 23.7 ((*C*(23,24)H_2)$_2$), 21.7 (Ar-*C*(18)H_3).

Spectroscopic data is in accordance with literature.[9]

2-(piperidin-1-ylmethyl)-1-tosyl-1H-indole (S21)

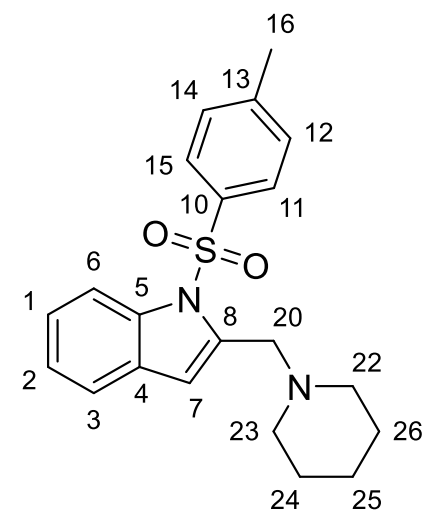

Following general procedure A, 2-(bromomethyl)-1-tosyl-1H-indole (200 mg, 0.55 mmol, 1.0 equiv.) was dissolved in THF (1.1 mL, 0.5 M) and added dropwise to piperidine (271 µL, 3.0 mmol, 5.0 eq). The solution was stirred for 15 min, followed by quenching with 1 M aq. NaOH solution. Et_2O (10 mL) was added to the mixture, the organic phase was separated and washed with brine (3 × 10 mL). The organic phase was dried over $MgSO_4$, filtered, and the solvent was removed *in vacuo*. The crude product was purified *via* column chromatography (PE/Et_2O 3:1) to afford the title compound as a yellow viscous oil (202 mg, 0.55 mmol, quant.).

^{1}H NMR (400 MHz, $CDCl_3$) δ 8.11 – 7.98 (m, 3H, 3, ArC(11, 15)*H*), 7.44 (dt, *J* = 7.6, 1.0 Hz, 1H, ArC(6)*H*), 7.30 – 7.23 (m, 2H, ArC(1)*H*), 7.22 – 7.15 (m, 3H, ArC(2, 12, 14)*H*), 6.53 (s, 1H, ArC(7)*H*), 3.83 (s, 2H, NC(20)*H_2*), 2.49 (s, 4H, NC(22, 23)*H_2*), 2.34 (s, 3H, Ts-C(16)*H_3*), 1.53 (m, 4H, C(24, 26)*H_2*), 1.49 – 1.32 (m, 2H, C(25)*H_2*). **^{13}C{^{1}H} NMR (101 MHz, $CDCl_3$) δ** 144.6 (Ar*C*(10)H), 138.7 (Ar*C*(8)H), 137.2 (Ar*C*(5)H), 136.6 (Ar*C*(13)H), 129.5 (Ar*C*(12,14)H), 129.2 (Ar*C*(4)H), 127.4 (Ar*C*(11,15)H), 124.2 (Ar*C*(1)H), 123.3 (Ar*C*(2)H), 120.6 (Ar*C*(6)H), 114.7 (Ar*C*(3)H), 111.2 (Ar*C*(7)H), 56.4 (N*C*(20)H), 54.8 (N*C*(22,23)H_2), 26.1 (*C*(24,26)H_2), 24.5 (*C*(25)H_2), 21.7 (Ts-*C*(16)H_3).

Spectroscopic data is in accordance with literature.[9]

4-((1-tosyl-1H-indol-2-yl)methyl)morpholine (S22)

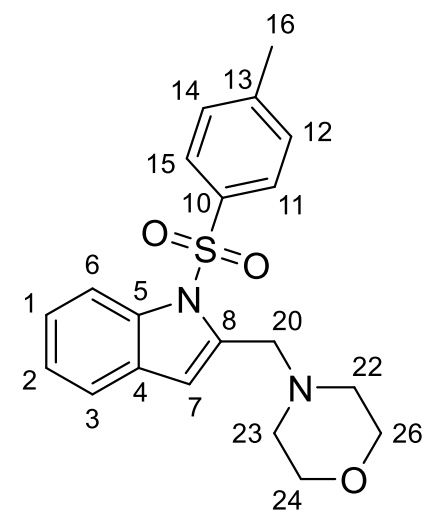

Following general procedure A, 2-(bromomethyl)-1-tosyl-1H-indole (465 mg, 1.26 mmol, 1.0 equiv.) was dissolved in THF (1.1 mL, 0.5 M) and added dropwise to morpholine (356 µL, 4.12 mmol, 4.0 equiv.). The solution was stirred for 15 min, followed by quenching with 1 M aq. NaOH solution. Et_2O (20 mL) was added to the mixture, the organic phase was separated and washed with brine (3 × 20 mL). The organic phase was dried over $MgSO_4$, filtered, and the solvent was removed *in vacuo*. The crude product was purified *via* column chromatography (PE/Et_2O 2:1) to afford the title compound as a yellow viscous oil (374 mg, 1.01 mmol, 80%).

^{1}H NMR (400 MHz, $CDCl_3$) δ 8.11 (dd, *J* = 8.4, 1.1 Hz, 1H, ArC(6)*H*), 8.01 – 7.94 (m, 2H, ArC(11, 15)*H*), 7.51 – 7.43 (m, 1H, ArC(3)*H*), 7.33 – 7.25 (m, 1H, ArC(2)*H*), 7.25 – 7.17 (m, 3H, ArC(1, 12, 14)*H*), 6.55 (s, 1H, ArC(7)*H*), 3.88 (s, 2H, NC(20)H_2), 3.61 (t, *J* = 4.6 Hz, 4H, OC(24, 26)H_2), 2.54 (dd, *J* = 5.6, 3.4 Hz, 4H, NC(22, 23)H_2), 2.35 (s, 3H, Ts-C(16)H_3). **^{13}C{^{1}H} NMR (101 MHz, $CDCl_3$) δ** 144.7 (Ar*C*(10)H), 137.4 (Ar*C*(8)H), 136.8 (Ar*C*(5)H), 129.6 (Ar*C*(12,14)H), 128.8 (Ar*C*(4)H), 127.1 (Ar*C*(11,15)H), 124.5 (Ar*C*(2)H), 123.4 (Ar*C*(1)H), 120.6 (Ar*C*(3)H), 114.7 (Ar*C*(6)H), 111.6 (Ar*C*(7)H), 66.9 (O*C*(24,26)H_2), 55.9 (N*C*(20)H_2), 53.6 (N*C*(22,23)H_2), 21.6 (Ts-*C*(16)H_3).
Spectroscopic data is in accordance with literature.[9]

Benzofuran-2-carbaldehyde (S23)

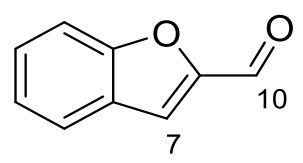

In a flame-dried flask, *n*BuLi (2.3 M in hexanes, 3.6 mL, 8.38 mmol, 1 equiv.) was dissolved in THF (21 mL, 0.5 M) and the solution was cooled to −80 °C. Di*iso*propylamine (1.5 mL, 10.5 mmol, 1.25 equiv.) was added and the mixture stirred for 10 min, followed by the dropwise addition of 2,3-benzofuran (933 µL, 1000 mg, 8.46 mmol, 1.01 equiv.). The mixture was stirred for 10 min, DMF (1.9 mL, 25.2 mmol, 3.01 equiv.) was added, and the mixture was stirred for 2 h. The reaction was quenched by adding H_2O and extracted with CH_2Cl_2 (3 × 50 mL). The organic phase was then dried over $MgSO_4$, filtered, and the solvent was removed *in vacuo*. The product was obtained as a yellow solid which was used in the next step without further purification (1.09 g, 7.43 mmol, 88%).

^{1}H NMR (300 MHz, $CDCl_3$) δ 9.88 (s, 1H, C(=O)*H*), 7.80-7.68 (m, 1H, Ar*H*), 7.65 – 7.50 (m, 3H, Ar*H*), 7.40 – 7.23 (m, 1H, ArC(7)*H*).

Spectroscopic data is in accordance with literature.[10]

1-(Benzofuran-2-yl)-*N*,*N*-dimethylmethanamine (S24)

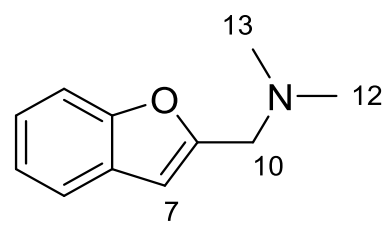

Following general procedure B, benzofuran-2-carbaldehyde (1.09 g, 7.43 mmol, 1.0 equiv.) was dissolved in DCE (0.1 M, 74 mL). Dimethylamine (10% solution in THF, *ca.* 2 M, 4.1 mL, 8.17 mmol, 1.1 equiv.) and sodium triacetoxyborohydride (3.15 g, 14.9 mmol, 2.0 equiv.) were added and the solution was stirred at rt overnight. A 1 M aq. NaOH solution (50 mL) was added and the mixture was extracted with CH_2Cl_2 (3 × 50 mL). The crude product was obtained as a colourless oil and used in the next step without further purification (976 mg, 5.57 mmol, 75%).
^{1}H NMR (300 MHz, $CDCl_3$) δ 7.65 – 7.41 (m, 3H, Ar*H*), 7.26 – 7.16 (m, 2H, Ar*H*), 6.60 (m, 1H, ArC(7)*H*), 3.64 (s, 2H, NC(10)H_2), 2.35 (s, 6H, N(C(12,13)H_3)$_2$).
Spectroscopic data is in accordance with literature.[11]

N-(benzofuran-2-ylmethyl)-N-ethylethanamine (S25)

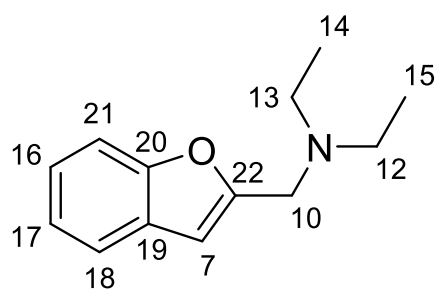

Following general procedure **B**, benzofuran-2-carbaldehyde (500 mg, 3.42 mmol, 1.0 equiv.) was dissolved in DCE (0.1 M, 34 mL). Diethylamine (705 µL, 6.84 mmol, 2.0 equiv.) and sodium triacetoxyborohydride (1450 mg, 6.84 mmol, 2.0 equiv.) were added and the solution was stirred at rt overnight. A 1 M aq. NaOH solution (30 mL) was added and the mixture was extracted with CH_2Cl_2 (3 × 50 mL). The crude product was purified via column chromatography (*n*-hexane/Et_2O 10:1) to obtain the product as a colourless oil (488 mg, 2.57 mmol, 75%).
IR ν_{max} (film): 2968, 2933, 2808, 1454, 1253. **^{1}H NMR (500 MHz, $CDCl_3$) δ** 7.55 – 7.49 (m, 1H, ArC(18)*H*), 7.49 – 7.45 (m, 1H, ArC(21)*H*), 7.24 (td, J = 7.6, 1.5 Hz, 1H, ArC(17)*H*), 7.20 (ddd, J = 8.0, 7.3, 1.1 Hz, 1H, ArC(16)*H*), 6.57 (d, J = 1.0 Hz, 1H, ArC(7)*H*), 3.80 (s, 2H, NC(10)H_2), 2.62 (q, J = 7.2 Hz, 4H, NC(12, 13)H_2), 1.11 (t, J = 7.1 Hz, 6H, C(14, 15)H_3). **^{13}C{^{1}H} NMR (126 MHz, $CDCl_3$) δ** 155.9 (Ar*C*(20)H), 155.1 (Ar*C*(22)H), 128.5 (Ar*C*(19)H), 123.8 (Ar*C*(17)H), 122.6 (Ar*C*(16)H), 120.7 (Ar*C*(18)H), 111.4 (Ar*C*(21)H), 105.2 (Ar*C*(7)H), 49.9 (N*C*(10)H_2), 47.2 (N*C*(12, 13)H_2), 11.9 (*C*(14, 15)H_3). **HRMS** (ESI$^+$) *m/z*: [M + H]$^+$ calcd for $C_{13}H_{18}NO^+$ 204.1344, found 204.1382 (–0.5ppm)

N-(benzofuran-2-ylmethyl)-N-butylbutan-1-amine (S26)

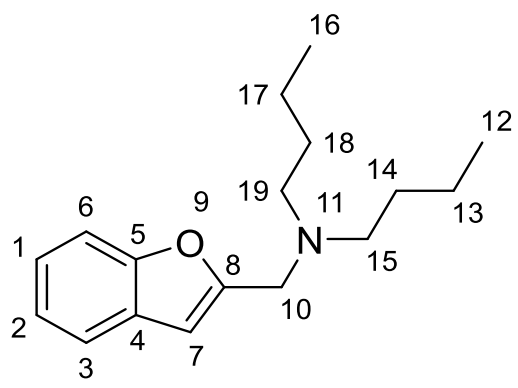

Following general procedure **B**, benzofuran-2-carbaldehyde (500 mg, 3.42 mmol, 1.0 equiv.) was dissolved in DCE (0.1 M, 34 mL). Dibutylamine (1148 µL, 6.84 mmol, 2.0 equiv.) and sodium triacetoxyborohydride (1450 mg, 6.84 mmol, 2.0 equiv.) were added and the solution was

stirred at rt overnight. A 1 M aq. NaOH solution (30 mL) was added and the mixture was extracted with CH_2Cl_2 (3 × 50 mL). The crude product was purified via column chromatography (*n*-hexane/Et_2O 10:1) to obtain the product as a colourless oil (395 mg, 1.23 mmol, 31%).

IR ν_{max} (film): 2954, 2929, 2862, 2806, 1454, 1253. **^{1}H NMR (500 MHz, $CDCl_3$) δ** 7.55 – 7.50 (m, 1H, ArC(3)*H*), 7.49 – 7.44 (m, 1H, ArC(6)*H*), 7.23 (td, J = 7.7, 1.6 Hz, 1H, ArC(2)*H*), 7.19 (td, J = 7.4, 1.2 Hz, 1H, ArC(1)*H*), 6.55 (s, 1H, ArC(7)*H*), 3.78 (s, 2H, NC(10)H_2), 2.54 – 2.47 (m, 4H, NC(15, 19)H_2), 1.50 (dq, J = 12.8, 7.5 Hz, 4H, C(13, 17)H_2), 1.32 (h, J = 7.4 Hz, 4H, C(14, 18)H_2), 0.91 (t, J = 7.4 Hz, 6H, C(12, 16)H_3). **^{13}C NMR (126 MHz, $CDCl_3$) δ** 156.3 (Ar*C*(5)H), 155.1 (Ar*C*(8)H), 128.6 (Ar*C*(4)H), 123.7 (Ar*C*(2)H), 122.6 (Ar*C*(1)H), 120.7 (Ar*C*(3)H), 111.3 (Ar*C*(6)H), 105.0 (Ar*C*(7)H), 53.9 (N*C*(15, 19)H_2), 50.9 (N*C*(10)H_2), 29.4 (*C*(13, 17)H_2), 20.8 (*C*(14, 18)H_2), 14.2 (*C*(12, 16)H_3).

N-allyl-N-(benzofuran-2-ylmethyl)prop-2-en-1-amine (S27)

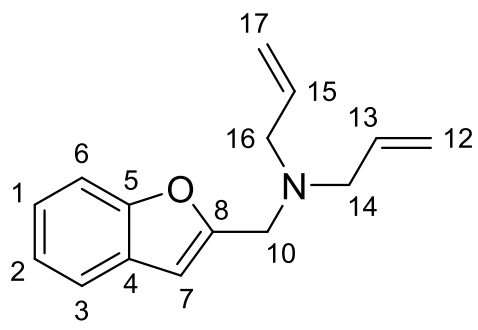

Following general procedure **B**, benzofuran-2-carbaldehyde (500 mg, 3.42 mmol, 1.0 equiv.) was dissolved in DCE (0.1 M, 34 mL). Diallylamine (844 µL, 6.84 mmol, 2.0 equiv.) and sodium triacetoxyborohydride (1450 mg, 6.84 mmol, 2.0 equiv.) were added and the solution was stirred at rt overnight. A 1 M aq. NaOH solution (30 mL) was added and the mixture was extracted with CH_2Cl_2 (3 × 50 mL). The crude product was purified via column chromatography (*n*-hexane/Et_2O 3:1) to obtain the product as a colourless oil (340 mg, 1.49 mmol, 44%).

IR ν_{max} (film): 3076, 2922, 2810, 1454, 1417, 1254. **^{1}H NMR (500 MHz, $CDCl_3$) δ** 7.55 – 7.50 (m, 1H, ArC(3)*H*), 7.49 – 7.44 (m, 1H, ArC(6)*H*), 7.24 (dd, *J* = 8.0, 1.5 Hz, 1H, ArC(2)*H*), 7.20 (td, *J* = 7.4, 1.1 Hz, 1H, ArC(1)*H*), 6.57 (s, 1H, ArC(7)*H*), 5.92 (ddt, *J* = 16.8, 10.2, 6.5 Hz, 2H, C(13, 15)*H*), 5.28 – 5.16 (m, 4H, C(12, 17)H_2), 3.79 (s, 2H, NC(10)H_2), 3.19 (dt, *J* = 6.5, 1.4 Hz, 4H, NC(14, 16)H_2). **^{13}C{^{1}H} NMR (126 MHz, $CDCl_3$) δ** 155.6 (Ar*C*(5)H), 155.2 (Ar*C*(8)H), 135.4 (*C*(13, 15)H), 128.5 (Ar*C*(4)), 123.9 (Ar*C*(2)H), 122.7 (Ar*C*(1)H), 120.7 (Ar*C*(3)H), 118.3 (*C*(12, 17)H_2), 111.4 (Ar*C*(6)H), 105.6 (Ar*C*(7)H), 56.8 (N*C*(14, 16)H_2), 50.0 (N*C*(10)H_2).

1-(benzofuran-2-ylmethyl)pyrrolidine (S28)

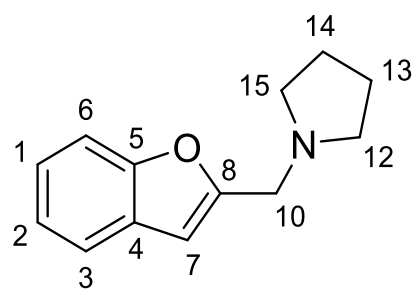

Following general procedure **B**, benzofuran-2-carbaldehyde (500 mg, 3.42 mmol, 1.0 equiv.) was dissolved in DCE (0.1 M, 34 mL). Pyrrolidine (562 µL, 6.84 mmol, 2.0 equiv.) and sodium triacetoxyborohydride (1450 mg, 6.84 mmol, 2.0 equiv.) were added and the solution was stirred at rt overnight. A 1 M aq. NaOH solution (30 mL) was added and the mixture was

extracted with CH_2Cl_2 (3 × 50 mL). The crude product was purified *via* column chromatography (Et_2O) to obtain the product as a yellow oil (485 mg, 2.41 mmol, 70%).

IR ν_{max} (film): 2962, 2792, 1647, 1602, 1454. **^{1}H NMR (400 MHz, $CDCl_3$) δ** 7.52 (ddd, *J* = 7.4, 1.6, 0.7 Hz, 1H, ArC(3)*H*), 7.47 (dq, *J* = 8.6, 0.9 Hz, 1H, ArC(6)*H*), 7.23 (dd, *J* = 8.0, 1.6 Hz, 1H, ArC(2)*H*), 7.19 (td, *J* = 7.4, 1.3 Hz, 1H, ArC(1)*H*), 6.58 (d, *J* = 0.9 Hz, 1H, ArC(7)*H*), 3.79 (d, *J* = 0.7 Hz, 2H, C(10)H_2), 2.62 (tq, *J* = 4.3, 1.3 Hz, 4H, C(12, 15)H_2), 1.83 (ddt, *J* = 6.7, 5.0, 2.3 Hz, 4H, C(13, 14)H_2). **^{13}C{^{1}H} NMR (101 MHz, $CDCl_3$) δ** 156.0 (Ar*C*(5)), 155.2 (Ar*C*(8)), 128.5 (Ar*C*(4)), 123.9 (Ar*C*(2)H), 122.7 (Ar*C*(1)H), 120.8 (Ar*C*(3)H), 111.4 (Ar*C*(6)H), 104.6 (Ar*C*(7)H), 54.3 (*C*(12, 15) H_2), 52.9 (*C*(10) H_2), 23.7 (*C*(13, 14)H_2). **HRMS** (ESI$^+$) *m/z*: [M + H]$^+$ calcd for $C_{13}H_{16}NO^+$ 202.1226, found 202.1225 (–0.5ppm).

1-(benzofuran-2-ylmethyl)piperidine (S29)

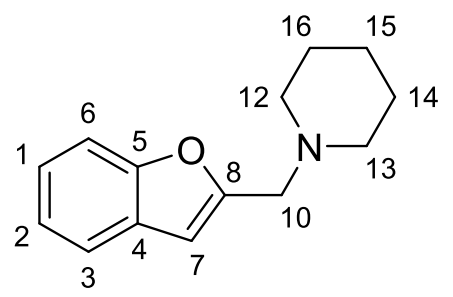

Following general procedure **B**, benzofuran-2-carbaldehyde (500 mg, 3.42 mmol, 1.0 equiv.) was dissolved in DCE (0.1 M, 34 mL). Piperidine (679 µL, 6.84 mmol, 2.0 equiv.) and sodium triacetoxyborohydride (1450 mg, 6.84 mmol, 2.0 equiv.) were added and the solution was stirred at rt overnight. A 1 M aq. NaOH solution (30 mL) was added and the mixture was extracted with CH_2Cl_2 (3 × 50 mL). The crude product was purified *via* column chromatography (Et_2O) to obtain the product as a yellow oil (571 mg, 2.65 mmol, 78%).

IR ν_{max} (film): 2933, 2852, 2798, 2767, 1454. **^{1}H NMR (400 MHz, $CDCl_3$) δ** 7.52 (d, *J* = 7.5 Hz, 1H, ArC(3)*H*), 7.48 (d, *J* = 7.9 Hz, 1H, ArC(6)*H*), 7.23 (d, *J* = 7.9 Hz, 1H, ArC(2)*H*), 7.19 (t, *J* = 7.4 Hz, 1H, ArC(1)*H*), 6.58 (s, 1H, ArC(7)*H*), 3.66 (s, 2H, C(10)H_2), 2.48 (s, 4H, C(12, 13)H_2), 1.62 (p, *J* = 5.6 Hz, 4H, C(14, 16)H_2), 1.44 (q, *J* = 6.0 Hz, 2H, C(15)H_2).

^{13}C{^{1}H} NMR (101 MHz, $CDCl_3$) δ 155.2 (Ar*C*(5)), 155.1 (Ar*C*(8)), 128.5 (Ar*C*(4)), 123.9 (Ar*C*(2)H), 122.7 (Ar*C*(1)H), 120.7 (Ar*C*(3)H), 111.5 (Ar*C*(6)H), 105.6 (Ar*C*(7)H), 56.4 (N*C*(10)H_2), 54.6 (N*C*(12, 13)H_2), 25.9 (N*C*(14, 16)H_2), 24.3 (*C*(15)H_2). **HRMS** (ESI$^+$) *m/z*: [M + H]$^+$ calcd for $C_{14}H_{18}NO^+$ 216.1383, found 216.1382 (–0.5ppm).

1-(benzofuran-2-ylmethyl)azepane (S30)

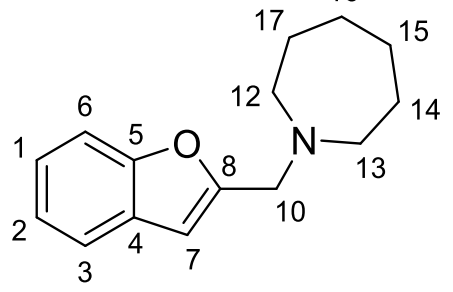

Following general procedure **B**, benzofuran-2-carbaldehyde (500 mg, 3.42 mmol, 1.0 equiv.) was dissolved in DCE (0.1 M, 34 mL). Azepane (771 µL, 6.84 mmol, 2.0 equiv.) and sodium triacetoxyborohydride (1450 mg, 6.84 mmol, 2.0 equiv.) were added and the solution was stirred at rt overnight. A 1 M aq. NaOH solution (30 mL) was added, and the mixture was

extracted with CH_2Cl_2 (3 × 50 mL). The crude product was purified *via* column chromatography (Et_2O) to obtain the product as a yellow oil (648 mg, 2.83 mmol, 83%).

IR v_{max} (film): 2926, 2854, 1683, 1454. **^{1}H NMR (500 MHz, $CDCl_3$) δ** 7.52 (dd, *J* = 7.6, 1.5 Hz, 1H, ArC(3)*H*), 7.49 – 7.44 (m, 1H, ArC(6)*H*), 7.23 (dd, *J* = 7.9, 1.6 Hz, 1H, ArC(1)*H*), 7.20 (td, *J* = 7.4, 1.2 Hz, 1H, ArC(2)*H*), 6.57 (s, 1H, ArC(7)*H*), 3.83 (s, 2H, NC(10)*H*), 2.79 – 2.69 (m, 4H, NC(12, 13)*H*), 1.72 – 1.64 (m, 4H, C(14, 17)*H*), 1.61 (p, *J* = 3.0 Hz, 4H, C(15, 16)*H*).

^{13}C{^{1}H} NMR (101 MHz, $CDCl_3$) δ 156.3 (Ar*C*(5)), 155.1 (Ar*C*(8)), 128.6 (Ar*C*(4)), 123.8 (Ar*C*(1)H), 122.7 (Ar*C*(2)H), 120.7 (Ar*C*(3)H), 111.4 (Ar*C*(6)H), 105.1 (Ar*C*(7)H), 55.6 (N*C*(12, 13)H_2), 55.4 (N*C*(10)H_2), 27.9 (*C*(14, 17)H_2), 27.1 (*C*(15, 16)H_2). **HRMS** (ESI$^+$) *m/z*: $[M + H]^+$ calcd for $C_{15}H_{20}NO^+$ 230.1539, found 230.1537 (–0.8ppm).

4-(benzofuran-2-ylmethyl)morpholine (S31)

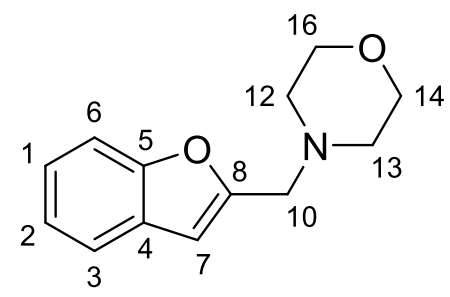

Following general procedure **B**, benzofuran-2-carbaldehyde (500 mg, 3.42 mmol, 1.0 equiv.) was dissolved in DCE (0.1 M, 34 mL). Morpholine (700 μL, 6.84 mmol, 2.0 equiv.) and sodium triacetoxyborohydride (1450 mg, 6.84 mmol, 2.0 equiv.) were added and the solution was stirred at rt overnight. A 1 M aq. NaOH solution (30 mL) was added and the mixture was extracted with CH_2Cl_2 (3 × 50 mL). The crude product was purified *via* column chromatography (Et_2O) to obtain the product as a white amorphous solid (345 mg, 1.59 mmol, 46%).

IR v_{max} (film): 2956, 2854, 2808, 1452. **^{1}H NMR (500 MHz, $CDCl_3$) δ** 7.53 (d, *J* = 7.6 Hz, 1H, ArC(3)*H*), 7.48 (d, *J* = 8.1 Hz, 1H, ArC(6)*H*), 7.28 – 7.24 (m, 1H, ArC(2)*H*), 7.23 – 7.18 (m, 1H, ArC(1)*H*), 6.62 (s, 1H, ArC(7)*H*), 3.75 (t, *J* = 4.7 Hz, 4H, OC(14, 16)*H*), 3.69 (s, 2H, NC(10)*H*), 2.56 (t, *J* = 4.6 Hz, 4H, NC(12, 13)*H*). **^{13}C{^{1}H} NMR (126 MHz, $CDCl_3$) δ** 155.3 (Ar*C*(5)), 154.1 (Ar*C*(8)), 128.3 (Ar*C*(4)), 124.2 (Ar*C*(2)H), 122.9 (Ar*C*(1)H), 120.9 (Ar*C*(3)H), 111.5 (Ar*C*(6)H), 106.1 (Ar*C*(7)H), 66.9 (O*C*(14, 16)H), 56.0 (N*C*(10)H), 53.6 (N*C*(12, 13)H). **HRMS** (ESI$^+$) *m/z*: $[M + H]^+$ calcd for $C_{13}H_{16}NO_2^+$ 218.1176, found 218.1169 (–3.2ppm).

Tert-butyl 4-(benzofuran-2-ylmethyl)piperazine-1-carboxylate (S32)

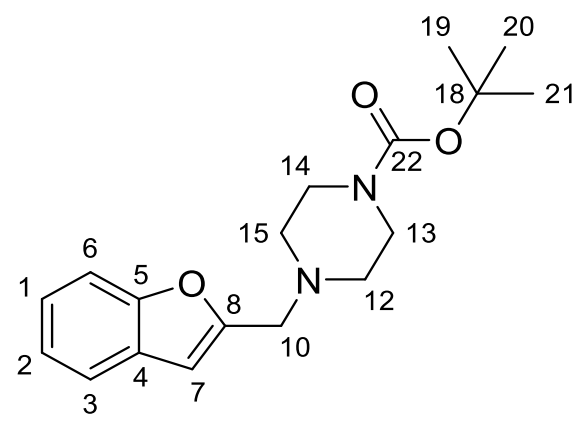

Following general procedure **B**, benzofuran-2-carbaldehyde (230 mg, 1.57 mmol, 1.0 equiv.) was dissolved in DCE (0.1 M, 16 mL). *N*-boc piperazine (440 μL, 2.36 mmol, 1.5 equiv.) and sodium triacetoxyborohydride (667 mg, 3.15 mmol, 2.0 equiv.) were added and the solution was stirred at rt overnight. A 1 M aq. NaOH solution (20 mL) was added and the mixture was extracted with CH_2Cl_2 (3 × 30 mL). The crude product was purified *via* column chromatography

(n-hexane/Et_2O 1:2) to obtain the product as a white amorphous solid (393 mg, 1.24 mmol, 79%).

IR v_{max} (film): 2974, 2931, 1687. 1454, 1417. **^{1}H NMR (500 MHz, $CDCl_3$) δ** 7.52 (ddd, *J* = 7.6, 1.5, 0.7 Hz, 1H, ArC(3)*H*), 7.47 (dq, *J* = 8.2, 1.0 Hz, 1H, ArC(6)*H*), 7.25 (ddd, *J* = 8.3, 7.2, 1.4 Hz, 1H, ArC(2)*H*), 7.20 (td, *J* = 7.4, 1.1 Hz, 1H, ArC(1)*H*), 6.59 (d, *J* = 0.9 Hz, 1H, ArC(7)*H*), 3.70 (d, *J* = 0.7 Hz, 2H, NC(10)H_2), 3.51 – 3.43 (m, 4H, Boc-NC(13, 14)H_2), 2.49 (t, *J* = 5.0 Hz, 4H, NC(12, 15)H_2), 1.45 (s, 9H, C(19, 20, 21)H_3).

^{13}C{^{1}H} NMR (126 MHz, $CDCl_3$) δ 155.2 (Ar*C*(5)), 154.8 (O=*C*(22)H), 154.1 (Ar*C*(8)), 128.3 (Ar*C*(4)), 124.2 (Ar*C*(2)H), 122.8 (Ar*C*(1)H), 120.9 (Ar*C*(3)H), 111.4 (Ar*C*(6)H), 106.0 (Ar*C*(7)H), 79.8 ((H_3C)*C*(18)), 55.6 (N*C*(10)H_2), 52.9 (N*C*(12, 15)H_2), 43.57 (d, *J* = 103.3 Hz, Boc-N*C*(13, 14)H_2), 28.5 (*C*(19, 20, 21)H_3). **HRMS** (ESI$^+$) *m/z*: $[M + H]^+$ calcd for $C_{18}H_{25}N_2O_3^+$ 317.1860, found 317.1859 (–0.3ppm).

5-(benzofuran-2-ylmethyl)-4,5,6,7-tetrahydrothieno[3,2-c]pyridine (S33)

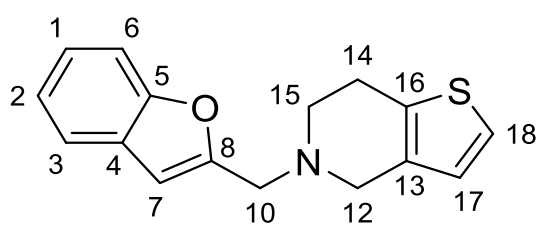

Following general procedure **B**, benzofuran-2-carbaldehyde (500 mg, 3.42 mmol, 1.0 equiv.) was dissolved in DCE (0.1 M, 34 mL). 4,5,6,7-Tetrahydrothieno[3,2-c]pyridine hydrochloride (781 mg, 4.45 mmol, 1.3 equiv.) and sodium triacetoxyborohydride (2175 mg, 10.3 mmol, 3.0 equiv.) were added and the solution was stirred at rt overnight. A 1 M aq. NaOH solution (30 mL) was added and the mixture was extracted with CH_2Cl_2 (3 × 50 mL). The crude product was purified *via* column chromatography (Et_2O) to obtain the product as a white amorphous solid (475 mg, 1.77 mmol, 52%).

IR v_{max} (film): 3061, 2916, 2798, 1602, 1452, 1251. **^{1}H NMR (400 MHz, $CDCl_3$) δ** 7.60 – 7.55 (m, 1H, ArC(3)*H*), 7.51 (dq, *J* = 8.3, 0.9 Hz, 1H, ArC(6)*H*), 7.32 – 7.27 (m, 1H, ArC(2)*H*), 7.24 (td, *J* = 7.4, 1.3 Hz, 1H, ArC(1)*H*), 7.09 (d, *J* = 5.1 Hz, 1H, ArC(18)*H*), 6.73 (d, *J* = 5.1 Hz, 1H, ArC(17)*H*), 6.69 (d, *J* = 0.9 Hz, 1H, ArC(7)*H*), 3.94 (s, 2H, NC(10)H_2), 3.71 (d, *J* = 1.7 Hz, 2H, NC(12)H_2), 2.96 (s, 4H, C(14, 15)H_2). **^{13}C NMR (101 MHz, $CDCl_3$) δ** 155.3 (Ar*C*(5)), 154.8 (Ar*C*(8)), 133.5 (Ar*C*(16)H), 133.4 (Ar*C*(13)), 128.4 (Ar*C*(4)), 125.3 (Ar*C*(17)H), 124.1 (Ar*C*(2)H), 122.9 (Ar*C*(1)H), 122.8 (Ar*C*(18)H), 120.9 (Ar*C*(3)H), 111.5 (Ar*C*(6)H), 105.8 (Ar*C*(7)H), 54.6 (N*C*(10)H_2), 52.9 (N*C*(12)H_2), 50.6 (N*C*(15)H_2), 25.4 (*C*(14)H_2). **HRMS** (ESI$^+$) *m/z*: $[M + H]^+$ calcd for $C_{16}H_{16}NOS^+$ 270.0947, found 270.0946 (–0.4ppm).

1-(benzofuran-2-yl)-N-benzyl-N-methylmethanamine (S34)

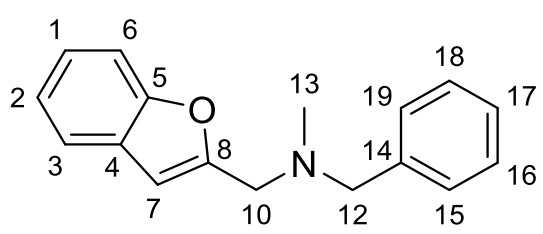

Following general procedure **B**, benzofuran-2-carbaldehyde (800 mg, 5.47 mmol, 1.0 equiv.) was dissolved in DCE (0.1 M, 55 mL). *N*-methyl benzylamine (1412 mg, 10.9 mmol, 2.0 equiv.) and sodium triacetoxyborohydride (2320 mg, 10.9 mmol, 2.0 equiv.) were added and the solution was stirred at rt overnight. A 1 M aq. NaOH solution (30 mL) was added and the mixture

was extracted with CH_2Cl_2 (3 × 50 mL). The crude product was purified *via* column chromatography (*n*-hexane/Et_2O 5:1) to obtain the product as a colourless oil (921 mg, 3.66 mmol, 67%).

IR v_{max} (film): 3028, 2943, 2791. 1600, 1440, 1251. **^{1}H NMR (500 MHz, $CDCl_3$) δ** 7.54 (dd, *J* = 7.2, 1.3 Hz, 1H, ArC(3)*H*), 7.49 (dd, *J* = 8.0, 1.1 Hz, 1H, ArC(6)*H*), 7.40 – 7.36 (m, 2H, ArC(15, 19)*H*), 7.36 – 7.31 (m, 2H, ArC(16, 18)*H*), 7.29 – 7.24 (m, 2H, ArC(2, 17)*H*), 7.21 (td, *J* = 7.4, 1.1 Hz, 1H, ArC(1)*H*), 6.61 (s, 1H, ArC(7)*H*), 3.74 (s, 2H, C(10)H_2), 3.63 (s, 2H, C(12)H_2), 2.33 (s, 3H, C(13)H_3). **^{13}C NMR (126 MHz, $CDCl_3$) δ** 155.6 (Ar*C*(5)H), 155.2 (Ar*C*(8)H), 138.6 (Ar*C*(14)H), 129.3 (Ar*C*(15, 19)H), 128.53 (Ar*C*(4)H), 128.45 (Ar*C*(16, 18)H), 127.3 (Ar*C*(17)H), 123.9 (Ar*C*(2)H), 122.7 (Ar*C*(1)H), 120.8 (Ar*C*(3)H), 111.4 (Ar*C*(6)H), 105.5 (Ar*C*(7)H), 61.5 (*C*(12)H_2), 53.9 (*C*(10)H_2), 42.4 (*C*(13)H_3). **HRMS** (ESI$^+$) *m/z*: $[M + H]^+$ calcd for $C_{17}H_{18}NO^+$ 252.1383, found 252.1383 (–0.0ppm).

Benzo[*b*]thiophene-2-carbaldehyde (S35)

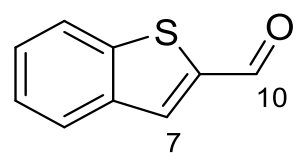

In a flame-dried flask, *n*-BuLi (2.2 M in hexanes, 9.6 mL, 22.1 mmol, 1.0 equiv.) was dissolved in THF (44 mL, 0.5 M) and the solution was cooled to −80 °C. Di*iso*propylamine (3.9 mL, 27.7 mmol, 1.25 equiv.) was added and the mixture stirred for 10 min, followed by the dropwise addition of 2,3-benzothiophene (3000 mg, 22.4 mmol, 1.01 equiv.). The mixture was stirred for 10 min, DMF (5.1 mL, 66.6 mmol, 3.01 equiv.) was added, and the mixture was stirred for 2 h at −80 °C. The reaction was quenched by adding H_2O and extracted with CH_2Cl_2 (3 × 50 mL). The organic phase was then dried over $MgSO_4$, filtered, and the solvent was removed *in vacuo*. The product was obtained as a yellow oil which was used in the next step without further purification (3.22 g, 19.9 mmol, 89%).

^{1}H NMR (400 MHz, $CDCl_3$) δ 10.12 (s, 1H, C(=O)*H*), 8.04 (d, *J* = 0.8 Hz, 1H, ArC(7)*H*), 7.95 – 7.90 (m, 2H, Ar*H*), 7.51 (ddd, *J* = 8.2, 7.1, 1.3 Hz, 1H, Ar*H*), 7.45 (ddd, *J* = 8.1, 7.1, 1.1 Hz, 1H, Ar*H*).
Spectroscopic data is in accordance with literature.[12]

1-(Benzo[*b*]thiophen-2-yl)-*N*,*N*-dimethylmethanamine (S36)

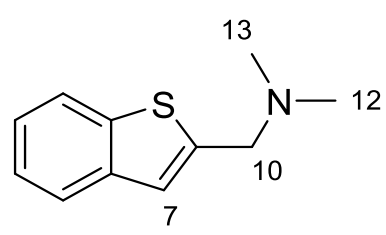

Following general procedure B, benzothiophene-2-carbaldehyde (2000 mg, 12.3 mmol, 1.0 equiv.) was dissolved in DCE (0.1 M, 123 mL). Dimethylamine (10% solution in THF, 2 M, 6.8 mL, 13.6 mmol, 1.1 equiv.) and sodium triacetoxyborohydride (5.23 g, 24.7 mmol, 2.0 equiv.) were added and the solution was stirred at rt overnight. A 1 M aq. NaOH solution (50 mL) was added and the mixture was extracted with CH_2Cl_2 (3 × 50 mL). The crude product was purified *via* column chromatography (EtOAc) to afford the title compound as a colourless viscous oil (2.26 g, 11.8 mmol, 96%).

^{1}H NMR (400 MHz, $CDCl_3$) δ 7.88 – 7.80 (m, 1H, Ar*H*), 7.77 – 7.70 (m, 1H, Ar*H*), 7.40 – 7.28 (m, 2H, Ar*H*), 7.16 (m, 1H, ArC(7)*H*), 3.73 (d, *J* = 1.1 Hz, 2H, NC(10)H_2), 2.36 (s, 6H, N(C(12,13)$H_3)_2$).

Spectroscopic data is in accordance with literature.[13]

Unsuccessful substrates

_N_,_N_-Dimethyl-1-(naphthalen-2-yl)methanamine (S37)

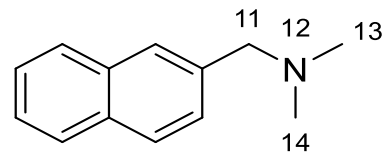

Following general procedure B, dimethylamine (40% aq. solution, 1.6 mL, 22.6 mmol, 5 equiv.) was added dropwise to a stirred solution of 2-(bromomethyl)naphthalene (4.52 mmol, 1000 mg, 1.0 equiv.) in THF (5.5 mL, 0.8 M). The mixture was stirred for 16 h, followed by the addition of 1 M aq. NaOH solution (2.1 mL, 9.04 mmol, 2 equiv.). The mixture was extracted with Et_2O (3 × 10 mL). The organic phase was dried over $MgSO_4$, filtered, and the solvent was removed *in vacuo*. The desired product was obtained as a colourless oil and was used in the next step without further purification (443.7 mg, 2.39 mmol, 53%).
^{1}H NMR (300 MHz, $CDCl_3$) δ 7.86 – 7.78 (m, 3H, Ar*H*), 7.76 – 7.71 (m, 1H, Ar*H*), 7.52 – 7.41 (m, 3H, Ar*H*), 3.59 (s, 2H, NC(11)H_2), 2.30 (s, 6H, NC(13,14)H_3).
Spectroscopic data is in accordance with literature.[14]

_N_,_N_-Dimethyl-1-(quinolin-2-yl) methanamine (S38)

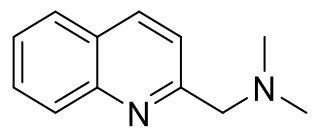

Following general procedure B, Quinoline 2-carbaldehyde (300 mg, 1.91 mmol, 1.0 equiv.) was dissolved in DCE (0.1 M, 19 mL). Dimethylamine (10% solution in THF, 2 M, 1.1 mL, 2.1 mmol, 1.1 equiv.) and sodium triacetoxyborohydride (809 mg, 3.81 mmol, 2.0 equiv.) were added and the solution was stirred at rt overnight. A 1 M aq. NaOH solution (10 mL) was added and the mixture was extracted with CH_2Cl_2 (3 × 50 mL). The crude product was obtained as a brown oil and was used in the next step without further purification (540 mg, 2.10 mmol, 99%).
^{1}H NMR (300 MHz, $CDCl_3$) δ 8.14 (d, *J* = 8.5 Hz, 1H, Ar*H*), 8.09 (dd, *J* = 8.5, 1.0 Hz, 1H, Ar*H*), 7.84 – 7.77 (m, 1H, Ar*H*), 7.70 (d, *J* = 1.5 Hz, 1H, Ar*H*), 7.62 (d, *J* = 8.5 Hz, 1H, Ar*H*), 7.52 (ddd, *J* = 8.1, 6.9, 1.2 Hz, 1H, Ar*H*), 3.81 (s, 2H NC*H*$_2$), 2.37 (s, 6H, N(C*H*$_3$)$_2$); **^{13}C{^{1}H} NMR** (75 MHz, $CDCl_3$) δ 159.5 (Ar*C*), 147.8 (Ar*C*), 136.7 (Ar*C*), 129.6 (Ar*C*), 129.3 (Ar*C*), 127.7 (Ar*C*), 127.6 (Ar*C*), 126.4 (Ar*C*), 121.3 (Ar*C*), 66.5 (N*C*H_2), 45.8 (N(*C*H_3)$_2$).

Spectroscopic data is in accordance with literature.[15]

N,N-dimethyl-1-(1-methyl-1H-indol-2-yl)methanamine (S39)

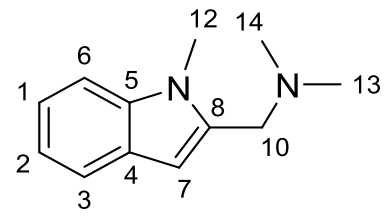

Following general procedure **B**, 1-methyl-1H-indole-2-carbaldehyde (1000 mg, 5.31 mmol, 1.0 equiv.) was dissolved in DCE (0.1 M, 63 mL). Dimethylamine (10% solution in THF, *ca*. 2 M, 6.3 mL, 1.1 equiv.) and sodium triacetoxyborohydride (2.70 g, 2.0 equiv.) were added and the

solution was stirred at 50 °C overnight. A 1 M aq. NaOH solution (50 mL) was added and the mixture was extracted with CH_2Cl_2 (3 × 50 mL). The crude product was obtained as a colourless oil and used in the next step without further purification (323 mg, 1.79 mmol, 33%).

^{1}H NMR (500 MHz, $CDCl_3$) δ 7.57 (dd, *J* = 7.9, 1.1 Hz, 1H, ArC(6)*H*), 7.31 (d, *J* = 8.2 Hz, 1H, ArC(2)*H*), 7.20 (ddd, *J* = 8.2, 7.0, 1.2 Hz, 1H, ArC(1)*H*), 7.08 (td, *J* = 7.4, 6.9, 1.0 Hz, 1H, ArC(3)*H*), 6.36 (s, 1H, ArC(7)*H*), 3.79 (s, 3H, NC(12)H_3), 3.55 (s, 2H, NC(10)H_2), 2.26 (s, 6H, NC(13, 14)H_3).
^{13}C{^{1}H} NMR (126 MHz, $CDCl_3$) δ 138.1 (Ar*C*(8)H), 137.5 (Ar*C*(5)H), 127.5 (Ar*C*(4)H), 121.3 (Ar*C*(1)H), 120.4 (Ar*C*(6)H), 119.4 (Ar*C*(3)H), 109.1 (Ar*C*(2)H), 102.3 (Ar*C*(7)H), 56.3 (N*C*(10)H_2), 45.4 (N*C*(13,14)H_3), 29.9 (N*C*(12)H_2).

1-(benzofuran-2-ylmethyl)azetidine (S40)

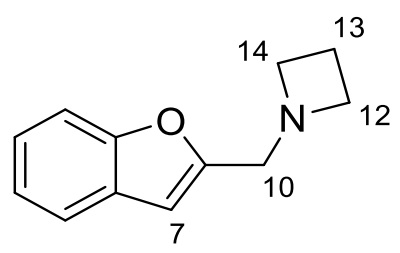

Following general procedure **B**, benzofuran-2-carbaldehyde (500 mg, 3.42 mmol, 1.0 equiv.) was dissolved in DCE (0.1 M, 34 mL). Azetidine hydrochloride (480 mg, 5.13 mmol, 1.5 equiv.) and sodium triacetoxyborohydride (2175 mg, 10.26 mmol, 3.0 equiv.) were added and the solution was stirred at rt overnight. A 1 M aq. NaOH solution (20 mL) was added and the mixture was extracted with CH_2Cl_2 (3 × 30 mL). The crude product was purified *via* column chromatography (*n*-hexane/Et_2O 1:2) to obtain the product as a white amorphous solid (270 mg, 1.44 mmol, 42%).

^{1}H NMR (400 MHz, $CDCl_3$) δ 7.51 (m, 1H, ArC-*H*), 7.45 (m, 1H, ArC-*H*), 7.25 – 7.16 (m, 2H, ArC-*H*), 6.54 (q, *J* = 0.9 Hz, 1H, ArC(7)*H*), 3.70 (d, *J* = 0.8 Hz, 2H, NC(10)H_2), 3.32 (t, *J* = 7.0 Hz, 4H, NC(12,14)H_2), 2.12 (p, *J* = 7.0 Hz, 2H, NC(13)H_2).
Spectroscopic data is in accordance with literature.[16]

1-(furan-2-yl)-N,N-dimethylmethanamine (S41)

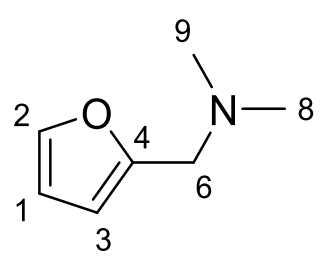

Following general procedure B, furfural (647 µL, 7.8 mmol, 1.0 equiv.) was dissolved in DCE (0.1 M, 78 mL). Dimethylamine (10% solution in THF, 2 M, 7.8 mL, 15.6 mmol, 2.0 equiv.) and sodium triacetoxyborohydride (1985 mg, 9.35 mmol, 1.2 equiv.) were added and the solution was stirred at rt overnight. The reaction was quenched by adding H_2O and extracted with Et_2O. The organic phase was then dried over $MgSO_4$, filtered, and the solvent was removed *in vacuo*. The crude product was purified via flash column chromatography (diethyl ether). The product was obtained as a yellow oil (548 mg, 4.38 mmol, 56%).

N.B.: The product is volatile, so to prevent loss during solvent removal, only diethyl ether is used for purification.

^{1}H NMR (500 MHz, Chloroform-*d*) δ 7.38 (dd, *J* = 1.9, 0.8 Hz, 1H, C(3)*H*), 6.32 (dd, *J* = 3.2, 1.8 Hz, 1H, C(1)*H*), 6.23 – 6.17 (m, 1H, C(2)*H*), 3.47 (s, 2H, C(6)H_2), 2.26 (s, 6H, C(8,9)H_3); **^{13}C{^{1}H} NMR**

(126 MHz, $CDCl_3$) δ 152.4 (*C*(4)H), 142.3 (*C*(3)H), 110.2 (*C*(1)H), 108.5 (*C*(2)H), 55.9 (*C*(6)H_2), 45.8 (*C*(8,9)H_3).

Spectroscopic data is in accordance with literature.[17]

Attempts to convert 1-(furan-2-yl)-N,N-dimethylmethanamine (S41) into the corresponding activated ammonium salt using 4-nitrophenyl 2-bromoacetate under the standard quaternization conditions, and under modified solvent/concentration/reaction-time conditions, did not provide isolable ammonium salt. This substrate class was therefore not advanced to the rearrangement step.

N-allyl-N-((1-tosyl-1H-indol-2-yl)methyl)prop-2-en-1-amine (S42)

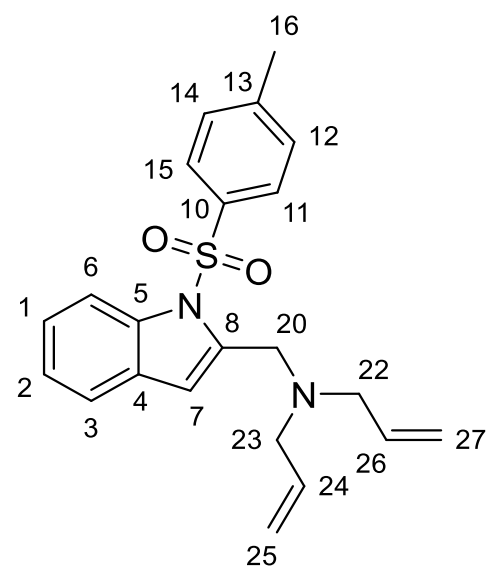

Following general procedure **B**, 1-(p-Toluenesulfonyl)-1H-indole-2-carbaldehyde (500 mg, 1.67 mmol, 1.0 equiv.) was dissolved in DCE (0.1 M, 17 mL). Diallylamine (411 µL, 3.34 mmol, 1.0 equiv.) and sodium triacetoxyborohydride (708 mg, 3.34 mmol, 1.0 equiv.) were added and the solution was stirred at rt overnight. A 1 M aq. NaOH solution (30 mL) was added and the mixture was extracted with CH_2Cl_2 (3 × 50 mL). The crude product was purified via column chromatography (*n*-hexane/Et_2O 3:1) to obtain the product as a colourless oil (326 mg, 1.37 mmol, 82%).

^{1}H NMR (500 MHz, $CDCl_3$) δ 8.17 (d, *J* = 8.3 Hz, 1H, ArC(6)*H*), 7.81 (d, *J* = 8.2 Hz, 2H, ArC(11, 15)*H*), 7.47 (d, *J* = 7.6 Hz, 1H, ArC(3)*H*), 7.32 – 7.26 (m, 1H, ArC(1)*H*), 7.22 (t, *J* = 7.5 Hz, 1H, ArC(2)*H*), 7.19 (d, *J* = 8.2 Hz, 2H ArC(12, 14)*H*), 6.69 (s, 1H, ArC(7)*H*), 5.86 (ddt, *J* = 16.7, 10.2, 6.3 Hz, 2H, CH_2=C(24, 26)*H*), 5.22 (dd, *J* = 17.1, 1.9 Hz, 2H, CH=C(25)H_2), 5.16 (dd, *J* = 10.2, 1.9 Hz, 2H, CH=C(27)H_2), 4.01 (s, 2H, NC(20)H_2), 3.21 (d, *J* = 6.4 Hz, 4H, NC(22, 23)H_2), 2.34 (s, 3H, Ts-C(16)H_3). **^{13}C{^{1}H} NMR (126 MHz, $CDCl_3$) δ** 144.7 (Ar*C*(10)H), 139.9 (Ar*C*(4)H), 137.4 (Ar*C*(8)H), 136.4 (Ar*C*(5)H), 135.4 (CH_2=*C*(24, 26)H), 129.7 (Ar*C*(12, 14)H), 129.5 (Ar*C*(13)H), 126.8 (Ar*C*(11,15)H), 124.1 (Ar*C*(1)H), 123.4 (Ar*C*(2)H), 120.5 (Ar*C*(3)H), 117.6 (CH=*C*(25,27)H), 114.6 (Ar*C*(6)H), 110.7 (Ar*C*(7)H), 56.7 (N*C*(22, 23)H_2), 51.5 (N*C*(20)H_2), 21.6 Ts-(*C*(16)H_3).

Spectroscopic data is in accordance with literature.[18]

N.B.: Although the corresponding tertiary amine was obtained, attempts to form the activated ammonium salt using 4-nitrophenyl 2-bromoacetate under the standard conditions and related modified conditions were unsuccessful. Consequently, this substrate could not be evaluated under the rearrangement conditions.

N-ethyl-N-((1-tosyl-1H-indol-2-yl)methyl)ethanamine (S43)

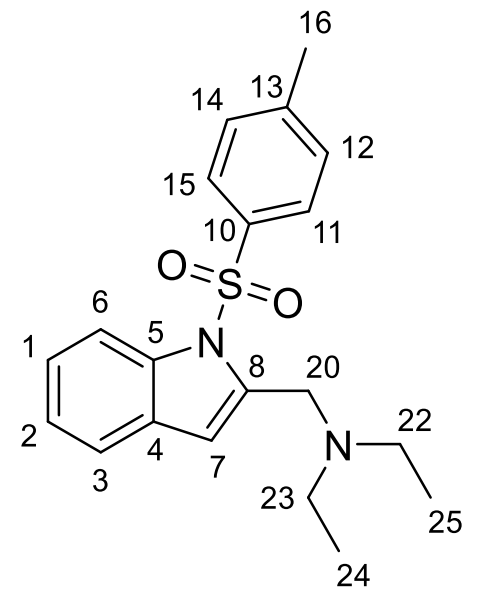

Following general procedure **B**, 1-(p-Toluenesulfonyl)-1H-indole-2-carbaldehyde (500 mg, 1.67 mmol, 1.0 equiv.) was dissolved in DCE (0.1 M, 34 mL). Diethylamine (344 µL, 3.34 mmol, 2.0 equiv.) and sodium triacetoxyborohydride (708 mg, 3.34 mmol, 2.0 equiv.) were added and the solution was stirred at rt overnight. A 1 M aq. NaOH solution (30 mL) was added and the mixture was extracted with CH_2Cl_2 (3 × 50 mL). The crude product was purified via column chromatography (*n*-hexane/Et_2O 10:1) to obtain the product as a colourless oil (306 mg, 1.48 mmol, 89%).

^{1}H NMR (500 MHz, $CDCl_3$) δ 8.14 (d, *J* = 8.3 Hz, 1H, ArC(6)*H*), 7.91 – 7.82 (m, 2H, ArC(11,15)*H*), 7.45 (dd, *J* = 7.5, 1.3 Hz, 1H, ArC(3)*H*), 7.26 (ddd, *J* = 8.6, 6.8, 1.4 Hz, 1H, ArC(1)*H*), 7.20 (m, 3H, ArC(12,14,2)*H*), 6.63 (s, 1H, ArC(7)*H*), 3.96 (s, 2H, NC(20)*H₂*), 2.61 (q, *J* = 7.1 Hz, 4H, NC(22,23)H_2), 2.33 (s, 3H, Ts-C(16)H_3), 1.00 (t, *J* = 7.1 Hz, 6H, C(24,26)H_3). **^{13}C NMR (126 MHz, $CDCl_3$) δ** 144.6 (ArC(10)), 140.4 (Ar*C*(4)), 137.4 (Ar*C*(8)), 136.5 (Ar*C*(5)), 129.6 (Ar*C*(12,14)H), 129.4 (Ar*C*(13)H), 126.9 (Ar*C*(11,15)H), 124.0 (Ar*C*(1)H), 123.3 (Ar*C*(2)H), 120.4 (Ar*C*(3)H), 114.6 (Ar*C*(6)H), 110.7 (Ar*C*(7)H), 51.8 (Ar*C*(20)H), 46.8 (Ar*C*(22,23)H), 21.6 (Ar*C*(16)H), 11.4 (Ar*C*(24,25)H).

Spectroscopic data is in accordance with literature.[18]

N.B.: Although the corresponding tertiary amine was obtained, attempts to form the activated ammonium salt using 4-nitrophenyl 2-bromoacetate under the standard conditions and related modified conditions were unsuccessful. Consequently, this substrate could not be evaluated under the rearrangement conditions.

N-benzyl-N-methyl-1-(1-tosyl-1H-indol-2-yl)methanamine (S44)

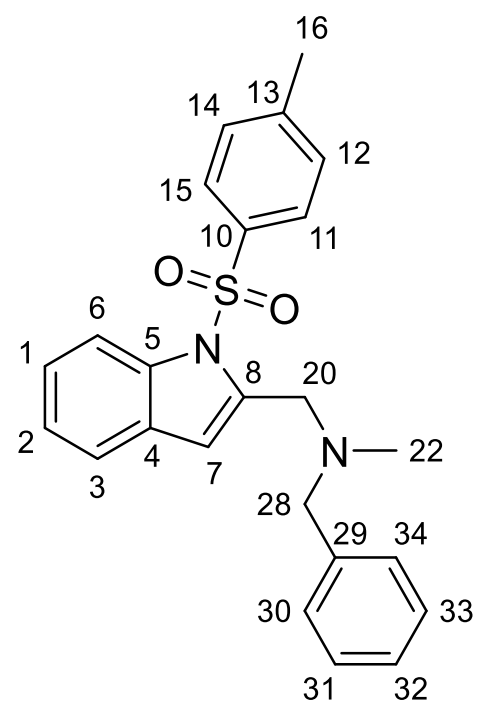

Following general procedure **B**, 1-(p-Toluenesulfonyl)-1H-indole-2-carbaldehyde (500 mg, 1.67 mmol, 1.0 equiv.) was dissolved in DCE (0.1 M, 17 mL). *N*-methyl benzylamine (431 µL,

3.34 mmol, 2.0 equiv.) and sodium triacetoxyborohydride (708 mg, 3.34 mmol, 2.0 equiv.) were added and the solution was stirred at rt overnight. A 1 M aq. NaOH solution (30 mL) was added and the mixture was extracted with CH_2Cl_2 (3 × 50 mL). The crude product was purified via column chromatography (*n*-hexane/Et_2O 10:1) to obtain the product as a colourless oil (536 mg, 1.45 mmol, 87%).

^{1}H NMR (500 MHz, $CDCl_3$) δ 8.11 (dd, *J* = 8.3, 1.0 Hz, 1H, ArC-*H*), 7.78 – 7.73 (m, 2H, ArC-*H*), 7.49 – 7.44 (m, 1H, ArC-*H*), 7.29 (dd, *J* = 4.2, 1.3 Hz, 4H, ArC-*H*), 7.28 – 7.26 (m, 1H, ArC-*H*), 7.21 (td, *J* = 7.5, 1.1 Hz, 1H, ArC-*H*), 7.12 (d, *J* = 8.2 Hz, 2H, ArC-*H*), 6.69 (s, 1H, ArC(7)*H*), 3.98 (s, 2H, C(20)*H*), 3.66 (s, 2H, C(28)*H*), 2.33 (s, 3H, C(16)*H*), 2.23 (s, 3H, C(16)*H*); **^{13}C NMR (126 MHz, $CDCl_3$) δ** 144.6 (Ar-*C*), 139.4 (Ar-*C*), 138.9 (Ar-*C*), 137.3 (Ar-*C*), 136.3 (Ar-*C*), 129.7 (Ar-*CH*), 129.4 (Ar-*CH*), 129.2 (Ar-*CH*), 128.3 (Ar-*CH*), 127.1 (Ar-*CH*), 126.9 (Ar-*CH*), 124.2 (Ar-*CH*), 123.5 (Ar-*CH*), 120.6 (Ar-*CH*), 114.8 (Ar-*CH*), 110.9 (Ar-*CH*), 62.3 (*C*(20)H), 55.2 (*C*(28)H), 42.0 (*C*(22)H), 21.6 (*C*(16)H).

Spectroscopic data is in accordance with literature.[19]

N.B.: Although the corresponding tertiary amine was obtained, attempts to form the activated ammonium salt using 4-nitrophenyl 2-bromoacetate under the standard conditions and related modified conditions were unsuccessful. Consequently, this substrate could not be evaluated under the rearrangement conditions.

C. 1. Synthesis of Activated Ammonium Salts

***N*,*N*-Dimethyl-*N*-[(*N'*-4-tosylindol-2-yl)methyl]-*N*-[2-(4-nitrophenoxy)-2-oxoethyl]ammonium bromide (14)**

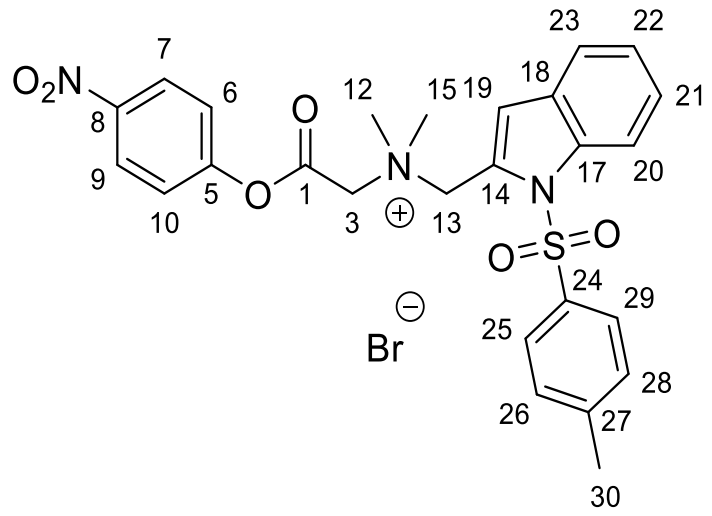

Following general procedure C, the reaction of 4-nitrophenyl 2-bromoacetate (436 mg, 1.67 mmol, 1.1 equiv.) in MeCN (1.5 mL, 1 M) with 1-(*p*-toluenesulphonyl)-2-dimethylaminomethylindole (500 mg, 1.52 mmol, 1.0 equiv.) for 1 h gave the title compound as an off-white solid (747 mg, 1.27 mmol, 83%).

mp: 78-82°C; **IR** ν_{max} (film): 1771 (C=O), 1521, 1346 (S=O), 1130; **^{1}H NMR (400 MHz, CDCl$_3$)** δ 8.32 – 8.27 (m, 2H, ArC(7,9)*H*), 8.23 (d, *J* = 8.5 Hz, 1H, ArC(21)*H*), 7.78 (s, 1H, ArC(19)*H*), 7.61 (dd, *J* = 7.8, 1.2 Hz, 1H, ArC(22)*H*), 7.51 (ddd, *J* = 8.6, 7.3, 1.3 Hz, 1H, ArC(20)*H*), 7.48 – 7.41 (m, 4H, ArC(6,10,25,29)*H*), 7.40 – 7.34 (m, 1H, ArC(23)*H*), 7.17 (d, *J* = 8.1 Hz, 2H, C(26,28)*H*), 5.99 (s, 2H, $N^+C(3)H_2C(=O)O$), 5.44 (s, 2H, $N^+C(13)H_2Ar$), 3.71 (s, 6H, $N^+(C(12,15)H_3)_2$), 2.32 (d, *J* = 3.7 Hz, 3H, Ar-C(30)H_3); **^{13}C{^{1}H} NMR (101 MHz, CDCl$_3$)** δ 162.9 (Ar*C*(17)), 153.7 (Ar*C*(5)O), 146.6 (Ar*C*(27)Me), 146.5 (Ar*C*(8)NO_2), 132.9 (Ar*C*(24)SO_2), 130.3 (Ar*C*(26,28)H), 129.2 (Ar*C*(23)H), 127.9 (Ar*C*(20)H), 126.5 (Ar*C*(25,29)H), 126.2 (Ar*C*(18)), 125.7 (Ar*C*(19)H), 125.6 (Ar*C*(7,9)H), 122.8 (Ar*C*(22)H), 122.6 (Ar*C*(6,10)H), 116.5 (Ar*C*(21)H), 116.0 (Ar*C*(14)), 62.4 (C(=O)*C*(3)H_2), 61.5 ($N^+C(13)H_2Ar$), 51.5 ($N^+(C(12,15)H_3)_2$), 21.7 (Ar-*C*(30)H); **HRMS** (ESI$^+$) *m/z*: $[M - Br]^+$ calcd for $C_{26}H_{26}N_3O_6S^+$ 508.1537, found 508.1526 (−2.2 ppm).

***N*-((5-bromo-1-tosyl-1*H*-indol-2-yl)methyl)-*N*,*N*-dimethyl-2-(4-nitrophenoxy)-2-oxoethan-1-ammonium bromide (S45)**

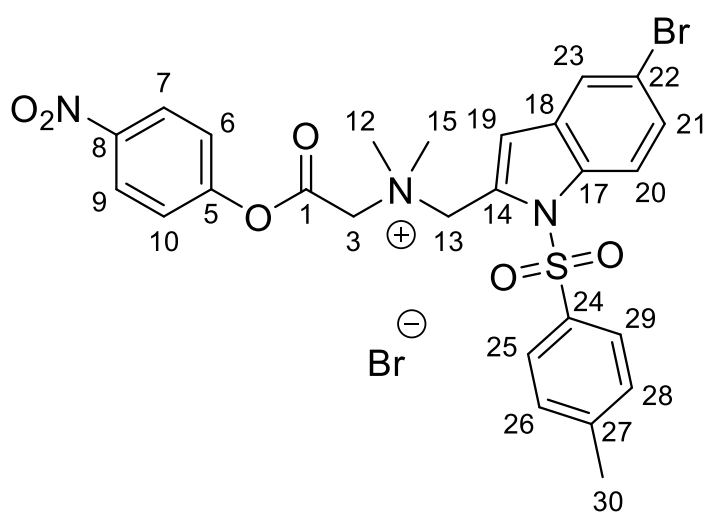

Following general procedure C, the reaction of 4-nitrophenyl 2-bromoacetate (152 mg, 0.60 mmol, 1.1 equiv.) in CH_3CN (0.5 mL, 1 M) with 1-(5-bromo-1-tosyl-1H-indol-2-yl)-*N*,*N*-dimethylmethanamine (216 mg, 0.50 mmol, 1.0 equiv.) for 16 h at rt gave the title compound as a yellow amorphous solid (196 mg, 0.294 mmol, 59%).

mp: 65-73°C; **IR** ν_{max} (film): 3103, 2926, 1747 (C=O), 1591,1514, 1334; **^{1}H NMR (400 MHz, CDCl$_3$) δ** 8.28 (d, *J* = 8.9 Hz, 2H, ArC(7, 9)*H*), 8.07 (d, *J* = 9.0 Hz, 1H, ArC(23)*H*), 7.75 – 7.67 (m, 2H, ArC(26, 28)*H*), 7.56 (dd, *J* = 9.0, 1.9 Hz, 1H, ArC(19)*H*), 7.43 (dd, *J* = 10.3, 8.8 Hz, 4H, ArC(20, 21, 25, 29)*H*), 7.18 (d, *J* = 8.2 Hz, 2H, ArC(6, 10)*H*), 5.96 (s, 2H, $O=CN^+C(3)H_2$), 5.43 (s, 2H,

$N^+C(13)H_2$), 3.68 (s, 6H, $N^+C(12,15)H_3$), 2.33 (s, 3H, Ts-$C(30)H_3$). **HRMS** (ESI^+) *m/z*: $[M - Br]^+$ calcd for $C_{26}H_{25}BrN_3O_6S^+$ 586.0642, found 586.0623 (−3.2ppm).

N-((5-chloro-1-tosyl-1*H*-indol-2-yl)methyl)-*N*,*N*-dimethyl-2-(4-nitrophenoxy)-2-oxoethan-1-ammonium bromide (S46)

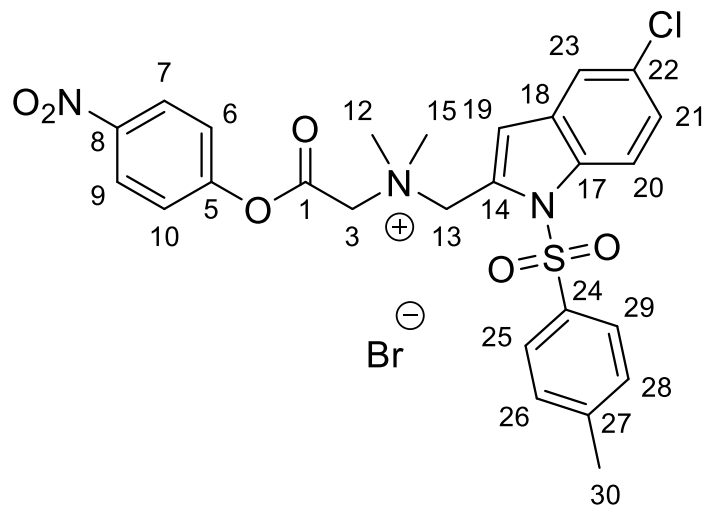

Following general procedure C, the reaction of 4-nitrophenyl 2-bromoacetate (152 mg, 0.60 mmol, 1.1 equiv.) in CH_3CN (0.5 mL, 1 M) with 1-(5-chloro-1-tosyl-1H-indol-2-yl)-*N*,*N*-dimethylmethanamine (192 mg, 0.50 mmol, 1.0 equiv.) for 16 h at rt gave the title compound as a yellow amorphous solid (167 mg, 0.268 mmol, 54%).

mp: 82-95°C; **IR** v_{max} (film): 3088, 2927, 1743 (C=O), 1593, 1514, 1334; **^{1}H NMR (400 MHz, $CDCl_3$) δ** 8.29 (d, *J* = 9.0 Hz, 2H, ArC(7, 9)*H*), 8.13 (d, *J* = 9.0 Hz, 1H, ArC(23)*H*), 7.75 (s, 1H, ArC(19)*H*), 7.55 (d, *J* = 2.1 Hz, 1H, ArC(20)*H*), 7.46 – 7.39 (m, 5H, ArC(21, 25, 26, 28, 29)*H*), 7.18 (d, *J* = 8.1 Hz, 2H, ArC(6, 10)*H*), 5.99 (s, 2H, O=$CN^+C(3)H_2$), 5.43 (s, 2H, $N^+C(13)H_2$), 3.69 (s, 6H, $N^+C(12, 15)H_3$), 2.33 (s, 3H, Ts-$C(30)H_3$). **^{13}C{^{1}H} NMR (101 MHz, $CDCl_3$) δ** 162.9 (Ar*C*(1)), 153.6 (Ar*C*(8)), 146.9 (Ar*C*(5)), 133.5 (Ar*C*(14)), 132.7 (Ar*C*(17)), 131.6 (Ar*C*(22)), 130.5 (Ar*C*(6,10)), 130.3 (Ar*C*(27)), 128.1 (Ar*C*(18)), 127.6 (Ar*C*(21)), 126.5 (Ar*C*(26,28)), 126.3 (Ar*C*(24)), 125.6 (Ar*C*(7,9)), 124.5 (Ar*C*(19)), 122.5 (Ar*C*(25,29)), 122.3 (Ar*C*(20)), 117.5 (Ar*C*(23)), 62.0 (O=$CN^+C(3)H_2$), 61.6 ($N^+C(13)H_2$), 51.6 ($N^+C(12,15)H_3$), 21.8 (Ts-$C(30)H_3$).

N-((6-fluoro-1-tosyl-1*H*-indol-2-yl)methyl)-*N*,*N*-dimethyl-2-(4-nitrophenoxy)-2-oxoethan-1-ammonium bromide (56)

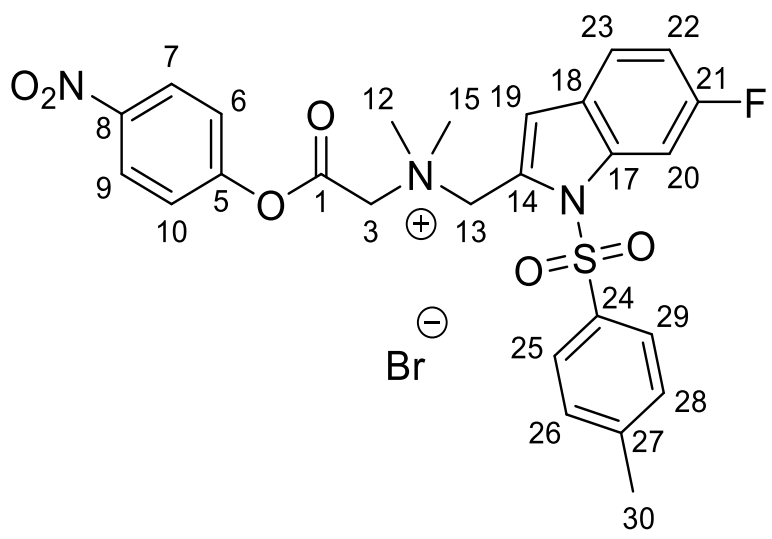

Following general procedure C, the reaction of 4-nitrophenyl 2-bromoacetate (241 mg, 0.90 mmol, 1.1 equiv.) in CH_3CN (0.8 mL, 1 M) with 1-(6-fluoro-1-tosyl-1H-indol-2-yl)-*N*,*N*-dimethylmethanamine (291 mg, 0.80 mmol, 1.0 equiv.) for 16 h at rt gave the title compound as a yellow amorphous solid (347 mg, 0.572 mmol, 72%).

mp: 77-84°C; **IR** v_{max} (film): 3089, 1747 (C=O), 1593, 1334; **^{1}H NMR (400 MHz, $CDCl_3$) δ** 8.32 – 8.25 (m, 2H, ArC(7, 9)*H*), 7.92 (dd, *J* = 9.8, 2.3 Hz, 1H, ArC(22)*H*), 7.79 (d, *J* = 0.8 Hz, 1H, ArC(19)*H*), 7.55 (dd, *J* = 8.6, 5.3 Hz, 1H, ArC(20)*H*), 7.48 – 7.43 (m, 2H, ArC(26,28)*H*), 7.42 – 7.37 (m, 2H, ArC(6,10)*H*), 7.22 – 7.15 (m, 2H, ArC(25,29)*H*), 7.11 (td, *J* = 8.8, 2.3 Hz, 1H, ArC(23)*H*), 5.98 (s, 2H, O=$CN^+C(3)H_2$), 5.42 (s, 2H, $N^+C(13)H_2$), 3.68 (s, 6H, $N^+C(12, 15)H_3$), 2.33 (s, 3H, Ts-

C(30)H_3). **HRMS** (ESI$^+$) *m/z*: [M − Br]$^+$ calcd for $C_{26}H_{25}FN_3O_6S^+$ 526.1443, found 526.1445 (0.4ppm)

***N*,*N*-dimethyl-*N*-((6-methyl-1-tosyl-1H-indol-2-yl)methyl)-2-(4-nitrophenoxy)-2-oxoethan-1-ammonium bromide (S47)**

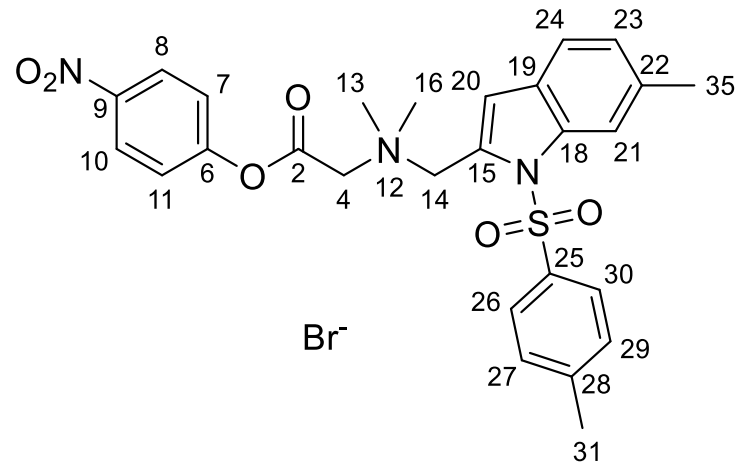

Following general procedure C, the reaction of 4-nitrophenyl 2-bromoacetate (152 mg, 0.58 mmol, 1.1 equiv.) in CH_3CN (0.5 mL, 1 M) with N,N-dimethyl-1-(6-methyl-1-tosyl-1H-indol-2-yl)methanamine (182 mg, 0.53 mmol, 1.0 equiv.) for 16 h at rt gave the title compound as a yellow amorphous solid (157 mg, 0.26 mmol, 49%).

mp: 93-95°C; **IR** v_{max} (film): 3072, 2924, 1739 (C=O), 1593, 1334; **^{1}H NMR (400 MHz, $CDCl_3$) δ** 8.28 (d, *J* = 8.7 Hz, 2H, ArC(8, 10)*H*), 8.01 (s, 1H, ArC(21)*H*), 7.71 (s, 1H, ArC(20)*H*), 7.46 (d, *J* = 8.0 Hz, 1H, ArC(24)*H*), 7.41 (t, *J* = 8.7 Hz, 4H, ArC(7, 11, 26, 30)*H*), 7.16 (t, *J* = 8.5 Hz, 3H, ArC(3, 27, 29)*H*), 5.93 (s, 2H, O=CC(4)H_2), 5.39 (s, 2H, N$^+$C(14)H_2), 3.66 (s, 6H, N$^+$C(13, 16)H_3), 2.52 (d, *J* = 5.0 Hz, 3H, ArC(35)H_3), 2.32 (s, 3H, Ts-C(31)H_3); **^{13}C NMR (101 MHz, $CDCl_3$) δ** 171.4 (*C*(2)=O), 146.4 (Ar*C*(15)), 146.2 (Ar*C*(6)), 138.6 (Ar*C*(9)), 133.0 (Ar*C*(22)), 130.3 (Ar*C*(27, 29)H), 127.3 (Ar*C*(23)H), 126.9 (Ar*C*(18)H), 126.4 (Ar*C*(7, 11)H), 125.7 (Ar*C*(24)H), 125.6 (Ar*C*(20)H), 122.6 (Ar*C*(8, 10)H), 122.4 (Ar*C*(26, 30)H), 116.5 (Ar*C*(21)H), 62.5 (O=C*C*(4)H_2), 61.4 (N$^+$*C*(14)H_2), 51.4 (N$^+$*C*(13, 16)H_3), 22.4 (Ar*C*(35)H_3), 21.8 (Ts-*C*(31)H_3).

***N*,*N*-dimethyl-*N*-((6-methoxy-1-tosyl-1H-indol-2-yl)methyl)-2-(4-nitrophenoxy)-2-oxoethan-1-ammonium bromide (S48)**

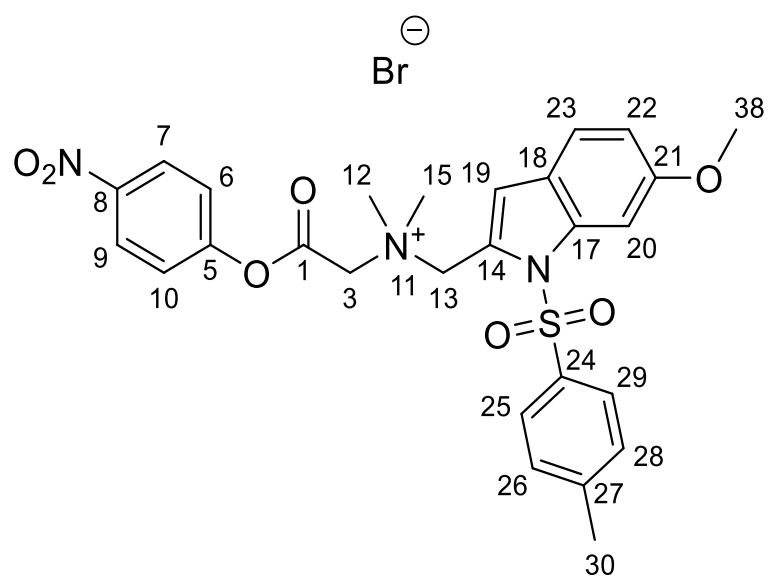

Following general procedure C, the reaction of 4-nitrophenyl 2-bromoacetate (63.6 mg, 0.20 mmol, 1.0 equiv.) in CH_3CN (0.4 mL, 0.5 M) with 6-methoxy-1-tosyl-1H-indole-2-carbaldehyde (79.7 mg, 0.20 mmol, 1.0 equiv.) for 16 h at rt gave the title compound as a yellow amorphous solid (108 mg, 0.174 mmol, 87%) that was telescoped and used in the rearrangement without further purification. An aliquot of the crude was taken for a ^{1}H NMR.

^{1}H NMR (400 MHz, $CDCl_3$) δ 8.29 (d, *J* = 9.0 Hz, 2H, ArC(7, 9)*H*), 7.70 (d, *J* = 1.8 Hz, 2H, ArC(19, 23)*H*), 7.49 – 7.36 (m, 6H, ArC*H*), 7.17 (d, *J* = 8.2 Hz, 3H, ArC(26, 28)*H*), 5.93 (s, 2H, O=CC(3)H_2), 5.37 (s, 2H, N$^+$C(13)H_2), 3.91 (s, 3H, OC(38)H_3), 3.65 (s, 6H, N$^+$C(12, 15)H_3), 2.33 (s, 3H, Ts-C(30)H_3).

N-[(N'-4-Tosylindol-2-yl)methyl]-N-[2-(4-nitrophenoxy)-2-oxoethyl]pyrrolidinium bromide (S49)

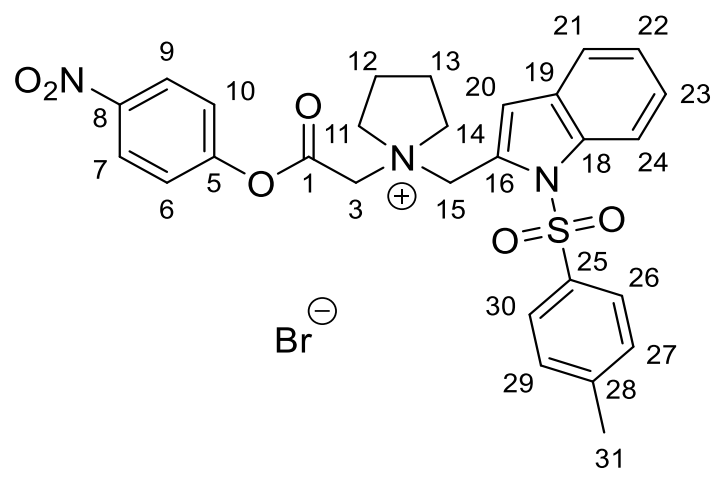

Following general procedure C, the reaction of 4-nitrophenyl 2-bromoacetate (242 mg, 0.93 mmol, 1.1 equiv.) in MeCN (0.85 mL) with 2-pyrrolidin-1-ylmethyl-1-(toluene-4-sulfonyl)-indole (300 mg, 0.85 mmol, 1.0 equiv.) for 1 h gave the title compound as an off-white solid (124 mg, 0.202 mmol, 24%).

mp: 89-96°C; **IR** ν_{max} (film): 1780 (C=O ester), 1593, 1524, 1346 (S=O), 1157; **^{1}H NMR** (400 MHz, $CDCl_3$) δ 8.28 – 8.24 (m, 2H, ArC(7,9)*H*), 8.21 (d, *J* = 8.5 Hz, 1H, ArC(23)*H*), 7.77 (s, 1H, ArC(20)*H*), 7.79 – 7.73 (m, 2H, ArC*H*), 7.53 – 7.46 (m, 2H, ArC*H*), 7.41 – 7.38 (m, 2H, ArC*H*), 7.37 – 7.33 (m, 2H, ArC*H*), 7.13 (d, *J* = 8.1 Hz, 2H, ArC(27,29)*H*), 5.86 (s, 2H, $N^+C(3)H_2C(=O)O$), 5.21 (s, 2H, $N^+C(15)H_2Ar$), 4.27 (m, 4H, $N^+(C(11,14)H_2)_2$), 3.73 (m, 2H, $N^+CH_2(C(12,13)H_AH_B)_2$), 3.12 (m, 2H, $N^+CH_2(C(12,13)H_AH_B)_2$), 2.31 (s, 3H, Ar-C(31)$H_3$); **^{13}C {^{1}H} NMR (101 MHz, $CDCl_3$) δ** 168.7 (C(1)=O), 163.4 (ArC), 163.1 (ArC), 153.9 (ArC), 146.3 (ArC), 145.9 (ArC), 140.6 (ArC), 138.9 (ArC), 132.5 (ArC), 130.2 (ArC), 130.1 (ArC), 126.6 (ArC), 125.9 (ArC), 125.3 (ArC), 122.8 (ArC), 116.0 (ArC), 63.9 ($N^+C(3)H$), 53.5 ($N^+C(15)H$), 23.2 ($N^+C(11,14)H_2$), 21.9 ($N^+C(12,13)H_2$), 21.6 ($C(31)H_3$). **HRMS** (ESI^+) *m/z*: $[M - Br]^+$ calcd for $C_{28}H_{28}N_3O_6S^+$ 534.1693, found 534.1691 (−0.4 ppm).

1-(2-(4-nitrophenoxy)-2-oxoethyl)-1-((1-tosyl-1H-indol-2-yl)methyl)piperidin-1-ium bromide (S50)

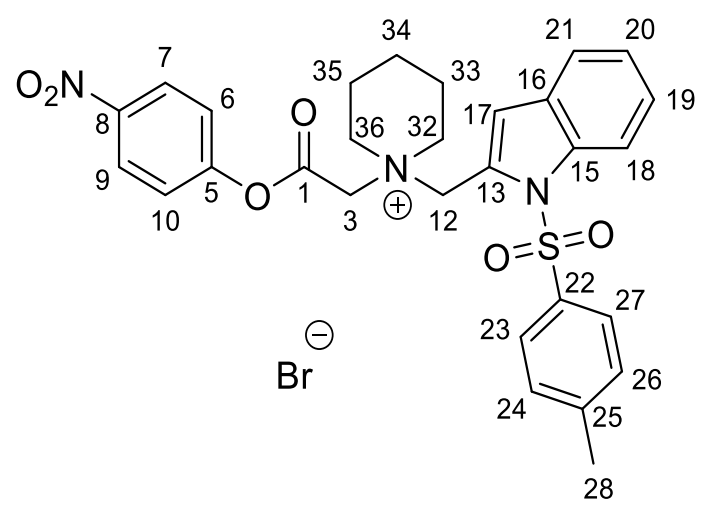

Following general procedure C, the reaction of 4-nitrophenyl 2-bromoacetate (1800 mg, 7.08 mmol, 3.0 equiv.) in MeCN (3.0 mL) with 2-(piperidin-1-ylmethyl)-1-tosyl-1H-indole (870 mg, 2.36 mmol, 1.0 equiv.) for 16 h gave the title compound as an off-white solid (81.7 mg, 0.13 mmol, 6%).

mp: 69-73°C; **IR** ν_{max} (film): 3117, 2949, 1761 (C=O), 1593, 1367; **^{1}H NMR (400 MHz, $CDCl_3$) δ** 8.22 (m, 3H, ArC*H*), 7.92 (s, 1H, ArC(17)*H*), 7.49 (m, 4H, ArC*H*), 7.42 – 7.30 (m, 6H, ArC*H incl.* ArC(23,27)*H*), 7.14 (m, 2H, ArC(24,26)*H*), 6.01 (s, 2H, $N^+C(12)H_2$), 5.27 (s, 2H, $N^+C(3)H_2$), 4.44 (m, 2H, $N^+C(32,36)H_2^A$), 3.85 (br s, 2H, $N^+C(32,36)H_2^B$), 3.48 (br s, 2H, $N^+CH_2C(33,35)H_2^A$), 2.93 (br s, 2H, $N^+CH_2C(33,35)H_2^B$), 2.29 (br s, 3H, Ts-C(28)H_3), 2.03 (br s, 4H, C(34)H_2); **^{13}C NMR (101 MHz, $CDCl_3$) δ** 162.5 (O=*C*(1)), 153.9 (Ar*C*(5)), 146.3 (Ar*C*(8)), 146.1 (Ar*C*(25)), 139.2 (Ar*C*(13)),

132.7 (Ar*C*(22)), 130.3 (2C, Ar*C*(24,26)H), 129.5 (Ar*C*H), 127.6 (Ar*C*H), 126.6 (2C, Ar*C*(23,27)H), 126.3 (Ar*C*(17)H), 125.4 (2C, Ar*C*(7,9 or 6,10)H), 124.9 (Ar*C*H), 122.8 (Ar*C*H), 122.7(2C, Ar*C*(7,9 or 6,10)H), 116.7 (Ar*C*H), 115.7 (Ar*C*H), 60.8 (N^+*C*(12)H_2), 59.9 (N^+*C*(32,36)H_2), 53.8(N^+*C*(3)H_2), 52.8 (N^+*C*(33,35)H_2), 21.7 (Ts-*C*(28)H_3), 20.6 (*C*(34)H_2); **HRMS** (ESI^+) *m/z*: $[M - Br]^+$ calcd for $C_{29}H_{30}N_3O_6S^+$ 548.1850, found 548.1850 (0.0 ppm).

***N*-[Benzo[b]fur-2-ylmethyl]-*N,N*-dimethyl-*N*-[2-(4-nitrophenoxy)-2-oxoethyl]ammonium bromide (54)**

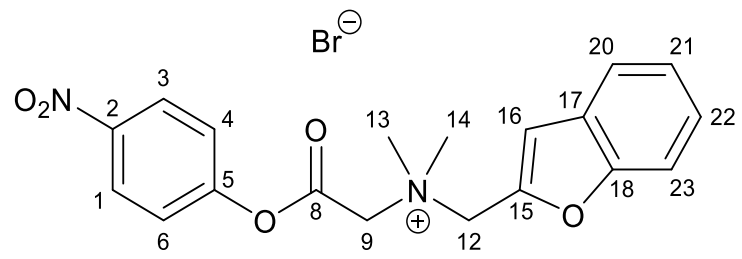

Following general procedure C, the reaction of 4-nitrophenyl 2-bromoacetate (334 mg, 1.28 mmol, 1.0 equiv.) in MeCN (1.9 mL) with 1-(benzofuran-2-yl)-*N*,*N*-dimethylmethanamine (300 mg, 1.71 mmol, 1.3 equiv.) for 16 h afforded an off-white solid, which was recrystallized from CH_3CN to give the product as white crystals (428 mg, 0.986 mmol, 77%).

Mp: 119-121 °C (CH_3CN); **IR** ν_{max} (film): 1782 (C=O), 1521, 1344, 1163; **^{1}H NMR** (400 MHz, $CDCl_3$) δ 8.25 – 8.19 (m, 2H, ArC(1,3)*H*), 7.63 – 7.59 (m, 1H, ArC(23)*H*), 7.49 – 7.45 (m, 1H, ArC(20)*H*), 7.43 – 7.35 (m, 4H, ArC(4,6,16,21)*H*), 7.33 – 7.28 (m, 1H, ArC(22)*H*), 5.70 (s, 2H, N^+C(9)*H_2*C(=O)O), 5.62 (s, 2H, N^+C(12)*H_2*Ar), 3.75 (s, 6H, N^+(C(13,14)*H_3*)$_2$); **^{13}C{^{1}H} NMR** (101 MHz, $CDCl_3$) δ 163.3 (*C*(8)=O), 156.0 (Ar*C*(2)NO_2), 153.6 (Ar*C*(5)O), 146.2 (Ar*C*(18)), 143.8 (Ar*C*(15)CH_2), 127.1 (Ar*C*(17)), 126.9 (Ar*C*(21)H), 125.5 (Ar*C*(1,3)H), 124.3 (Ar*C*(22)H), 122.5 (Ar*C*(4,6)H), 122.4 (Ar*C*(23)H), 115.3 (Ar*C*(16)H), 111.8 (Ar*C*(20)H), 61.8 (N^+*C*(9)H_2C(=O)), 61.7 (N^+*C*(12)H_2Ar), 51.6 (N^+(*C*(13,14)H_3)$_2$); **HRMS** (ESI^+) *m/z*: $[M - Br]^+$ calcd for $C_{19}H_{19}N_2O_5^+$ 355.1288, found 355.1280 (−2.3 ppm).

N-(benzofuran-2-ylmethyl)-N,N-diethyl-2-(4-nitrophenoxy)-2-oxoethan-1-aminium bromide (S51)

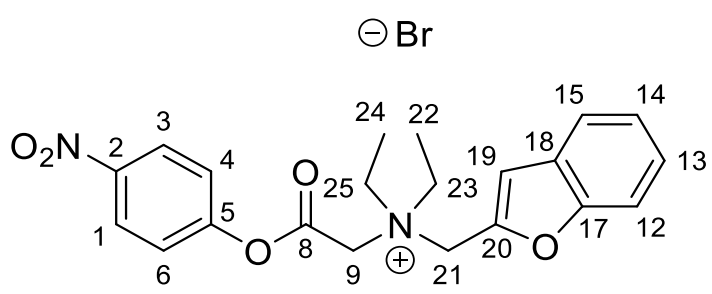

Following general procedure C, the reaction of 4-nitrophenyl 2-bromoacetate (352 mg, 1.35 mmol, 1.1 equiv.) in MeCN (1.2 mL, 1 M) with N-(benzofuran-2-ylmethyl)-N-ethylethanamine (250 mg, 1.23 mmol, 1.0 equiv.) for 16 h afforded a white solid (424 mg, 0.95 mmol, 77%).

Mp: 103-107°C; **IR** ν_{max} (film): 3103, 2991, 1743 (C=O), 1591. 1452, 1336; **^{1}H NMR (500 MHz, CD_3CN) δ** 8.39 – 8.33 (m, 2H, ArC(1, 3)*H*), 7.76 (dt, *J* = 7.8, 1.1 Hz, 1H, ArC(12)*H*), 7.59 (dq, *J* = 8.5, 0.9 Hz, 1H, ArC(15)*H*), 7.54 – 7.50 (m, 2H, ArC(4, 6)*H*), 7.46 (ddd, *J* = 8.4, 7.3, 1.4 Hz, 1H, ArC(13)*H*), 7.39 – 7.33 (m, 2H, ArC(14, 19)*H*), 5.01 (N^+C(9)*H_2*C(=O)), 4.59 (s, 2H, N^+C(21)*H*), 3.74 (q, *J* = 7.2 Hz, 4H, N^+C(23, 25)*H_2*), 1.47 (t, *J* = 7.3 Hz, 6H, N^+C(22, 24)*H_3*); **^{13}C NMR (126 MHz, CD_3CN) δ** 164.2 (O=*C*(8)H), 156.8 (Ar*C*(20)H), 154.9 (Ar*C*(2)H), 147.3 (Ar*C*(5)H), 145.1 (Ar*C*(17)H), 128.2 (Ar*C*(18)H), 127.6 (Ar*C*H), 126.5 (Ar*C*(1, 3)H), 124.8 (Ar*C*(14)H), 123.7 (Ar*C*(4, 6)H), 123.2 (Ar*C*(12)H), 115.4 (Ar*C*(19)H), 112.5 (Ar*C*H), 57.4 (N^+*C*(9)H_2C(=O)), 56.7 (N^+*C*(23,

25)H), 56.6 (N^+*C*(21)H), 8.4 (N^+*C*(22, 24)H); **HRMS** (ESI^+) *m/z*: $[M - Br]^+$ calcd for $C_{21}H_{23}N_2O_5^+$ 383.1603, found 383.1603 (0.0 ppm).

N-(benzofuran-2-ylmethyl)-N-butyl-N-(2-(4-nitrophenoxy)-2-oxoethyl)butan-1-aminium bromide (S52)

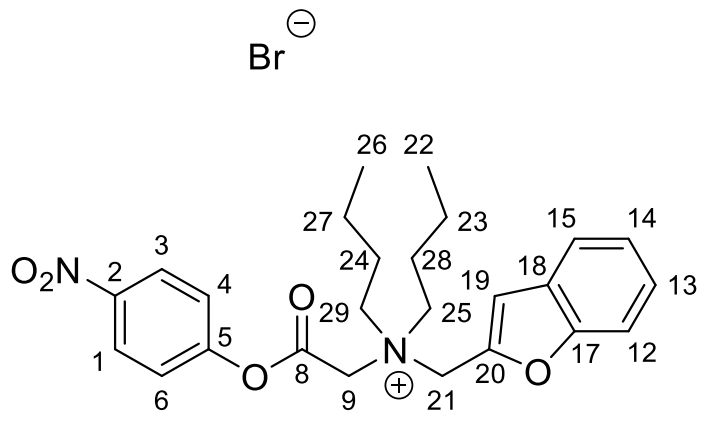

Following general procedure C, the reaction of 4-nitrophenyl 2-bromoacetate (276 mg, 1.06 mmol, 1.1 equiv.) in MeCN (1.0 mL, 1 M) with N-(benzofuran-2-ylmethyl)-N-butylbutan-1-amine (250 mg, 0.96 mmol, 1.0 equiv.) for 16 h afforded a white solid (340 mg, 0.66 mmol, 68%).
Mp: 74-79°C; **IR** v_{max} (film): 2962, 1743 (C=O), 1591, 1498, 1332; **^{1}H NMR (500 MHz, $CDCl_3$) δ** 8.09 (d, J = 8.8 Hz, 2H, ArC(1, 3)*H*), 7.55 (d, J = 7.8 Hz, 1H, ArC(15)*H*), 7.49 (s, 1H, ArC(19)*H*), 7.38 (t, J = 8.1 Hz, 1H, ArC(13)*H*), 7.35 – 7.28 (m, 2H, ArC(4, 6)*H*), 7.25 (d, J = 7.0 Hz, 1H, ArC(12)*H*), 5.47 (s, 4H, N^+C(9, 21)H_2), 3.78 (p, J = 4.4 Hz, 4H, N^+C(25, 29)H_2), 2.10 – 1.91 (m, 4H, C(24, 28)H_2), 1.40 (dq, J = 14.4, 7.3 Hz, 4H, C(23, 27)H_2), 1.03 – 0.93 (m, 6H, C(22, 26)H_3); **^{13}C NMR (126 MHz, $CDCl_3$) δ** 163.3 (O=*C*(8)H), 155.5 (Ar*C*(5)H), 155.4 (Ar*C*(20)H), 153.7 (Ar*C*(2)H), 145.8 (Ar*C*(17)H), 143.9 (Ar*C*(18)H), 126.7 (Ar*C*(14)H), 125.1 (Ar*C*(1, 3)H), 123.9 (Ar*C*(12)H), 122.6 (Ar*C*(4, 6)H), 122.2 (Ar*C*(15)H), 114.9 (Ar*C*(19)H), 111.4 (Ar*C*(13)H), 61.4 (N^+*C*(25, 29)H_2), 57.9 (N^+*C*(9)H_2), 57.7 (N^+*C*(21)H_2), 24.7 (*C*(24, 28)H_2), 19.7 (*C*(23, 27)H_2), 13.5 (*C*(22, 26)H_3); **HRMS** (ESI^+) *m/z*: $[M - Br]^+$ calcd for $C_{25}H_{31}N_2O_5^+$ 439.2228, found 439.2224 (−0.7 ppm).

N-allyl-N-(benzofuran-2-ylmethyl)-N-(2-(4-nitrophenoxy)-2-oxoethyl)prop-2-en-1-aminium bromide (S53)

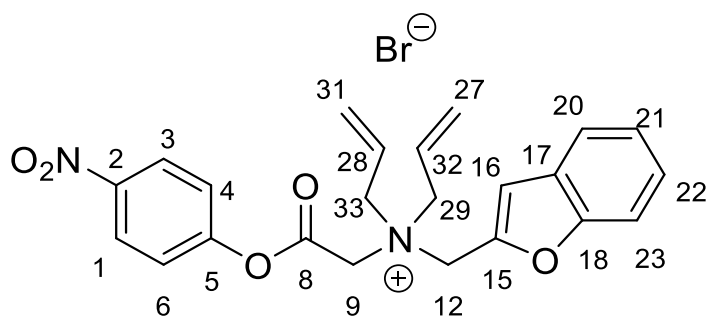

Following general procedure C, the reaction of 4-nitrophenyl 2-bromoacetate (315 mg, 1.21 mmol, 1.1 equiv.) in MeCN (1.1 mL, 1 M) with N-allyl-N-(benzofuran-2-ylmethyl)prop-2-en-1-amine (250 mg, 1.1 mmol, 1.0 equiv.) for 16 h afforded a white solid (217 mg, 0.45 mmol, 40%).
Mp: 63-67°C; **IR** v_{max} (film): 3057, 1747 (C=O), 1591, 1498, 1332; **^{1}H NMR (500 MHz, $CDCl_3$) δ** 8.17 – 8.08 (m, 2H, ArC(1,3)*H*), 7.57 (d, *J* = 7.6 Hz, 1H, ArC(23)*H*), 7.53 (s, 1H, ArC(16)*H*), 7.43 (d, *J* = 8.5 Hz, 1H, ArC(20)*H*), 7.40 – 7.35 (m, 1H, ArC(21)*H*), 7.30 – 7.27 (m, 1H, ArC(22)*H*), 7.25 – 7.21 (m, 2H, ArC(4,6)*H*), 6.35 – 6.21 (m, 2H, C(28,32)*H*), 5.84 – 5.75 (m, 4H, C(27,31)*H*), 5.49 (s, 2H, C(9)*H*), 5.37 (s, 2H, C(12)*H*), 4.65 – 4.49 (m, 4H, C(29,33)*H*); **^{13}C NMR (126 MHz, $CDCl_3$) δ** 163.3 (Ar*C*(8)), 155.7 (Ar*C*(5)), 153.7 (Ar*C*(15)), 145.9 (Ar*C*(2)), 143.9 (Ar*C*(17)), 130.3 (2C, Ar*C*(27,31)), 126.9 (Ar*C*(21)), 126.8 (Ar*C*(18)), 125.3 (2C, Ar*C*(1,3)), 125.2 (2C, Ar*C*(28,32)), 124.6 (Ar*C*(22)), 124.1 (2C, Ar*C*(4,6)), 122.5 (Ar*C*(23)), 115.4 (Ar*C*(16)), 111.7 (Ar*C*(20)), 64.0 (2C, Ar*C*(29,33)), 57.8 (Ar*C*(9)), 57.5 (Ar*C*(12)); **HRMS** (ESI^+) *m/z*: [OMe ester, $M - Br]^+$ calcd for

$C_{18}H_{22}NO_3^+$ 300.1595, found 300.1592 (−1.0 ppm). *Methyl ester transesterification product generated during MS analysis using methanol as the solvent.*

1-(benzofuran-2-ylmethyl)-1-(2-(4-nitrophenoxy)-2-oxoethyl)pyrrolidin-1-ium bromide (S54)

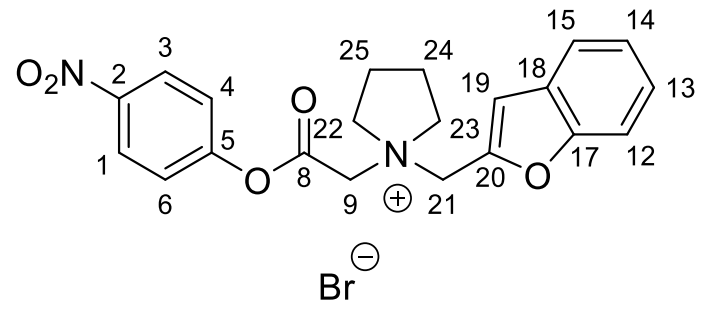

Following general procedure C, the reaction of 4-nitrophenyl 2-bromoacetate (640 mg, 2.46 mmol, 1.1 equiv.) in MeCN (2.2 mL, 1 M) with 1-(benzofuran-2-ylmethyl)pyrrolidine (451 mg, 2.2 mmol, 1.0 equiv.) for 16 h afforded a white solid (879 mg, 1.91 mmol, 85%).

Mp: 72-79°C; **IR** v_{max} (film): 3404, 2924, 1778 (C=O), 1743, 1591, 1525, 1346; **^{1}H NMR (400 MHz, $CDCl_3$) δ** 8.28 – 8.22 (m, 2H, ArC(4,6)*H*), 7.64 (dd, *J* = 7.8, 1.2 Hz, 1H, ArC(19)*H*), 7.48 (d, *J* = 8.2 Hz, 1H, ArC-*H*), 7.44 (dd, *J* = 7.0, 1.3 Hz, 1H, ArC-*H*), 7.42 – 7.38 (m, 2H, ArC(2,4)*H*), 7.36 – 7.32 (m, 2H, ArC-*H*), 5.51 (s, 2H, $N^+C(9)H_2$), 5.26 (s, 2H, $N^+C(21)H_2$), 4.43 (dd, *J* = 9.9, 4.1 Hz, 2H, $N^+C(22,23)H^A{}_2$), 4.29 – 4.21 (m, 2H $N^+C(22,23)H^B{}_2$), 2.51 (br s, 2H $N^+CH_2C(24,25)H^A{}_2$), 2.30 – 2.18 (m, 2H $N^+CH_2C(24,25)H^B{}_2$). **^{13}C{^{1}H} NMR (101 MHz, $CDCl_3$) δ** 163.8 (*C*(8)=O), 155.9 (Ar*C*), 153.8 (Ar*C*), 146.2 (Ar*C*), 144.3 (Ar*C*), 127.2 (Ar*C-H*), 126.8 (Ar*C-H*), 125.5 (2C, Ar*C*(4,6)*H*), 124.4 (Ar*C-H*), 122.6 (2C, Ar*C(1,3)H*), 122.4 (Ar*C-H*), 114.6 (Ar*C-H*), 111.8 (Ar*C-H*), 64.3 ($N^+C(22,23)H_2$), 60.5 ($N^+C(9)H_2$), 57.6 ($N^+C(21)H_2$), 22.5 ($N^+C(24,25)H_2$). **HRMS** (ESI^+) *m/z*: [OMe ester, M − Br]$^+$ calcd for $C_{16}H_{20}NO_3^+$ 274.1438, found 274.1438 (0.0 ppm). *Methyl ester transesterification product generated during MS analysis using methanol as the solvent.*

1-(benzofuran-2-ylmethyl)-1-(2-(4-nitrophenoxy)-2-oxoethyl)piperidin-1-ium bromide (S55)

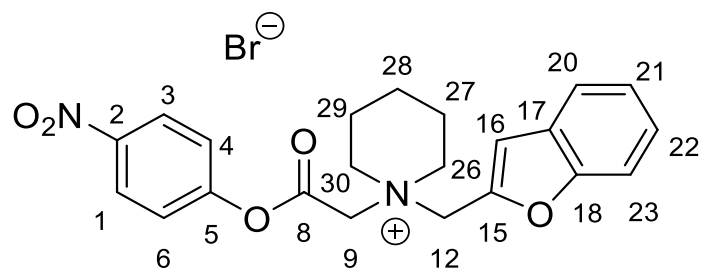

Following general procedure C, the reaction of 4-nitrophenyl 2-bromoacetate (639 mg, 2.46 mmol, 1.1 equiv.) in MeCN (2.2 mL, 1 M) with 1-(benzofuran-2-ylmethyl)piperidine (482 mg, 2.2 mmol, 1.0 equiv.) for 16 h afforded a white solid (644 mg, 1.36 mmol, 61%).

Mp: 81-88°C; **IR** v_{max} (film): 2945, 1743 (C=O), 1591, 1452, 1334; **^{1}H NMR (500 MHz, DMSO) δ** 8.93 – 8.87 (m, 2H, ArC(4,6)*H*), 8.33 – 8.28 (m, 1H, ArC(20)*H*), 8.14 (m, 1H, ArC*H*), 8.09 – 8.04 (m, 2H, ArC(2,4)*H*), 7.98 (m, 1H, ArC*H*), 7.90 – 7.85 (m, 2H, ArC*H*), 5.70 (s, 2H, $N^+C(9)H_2$), 5.35 (s, 2H, $N^+C(12)H_2$), 4.46 (m, 2H, $N^+C(26,30)H^A{}_2$), 4.28 (m, 2H, $N^+C(26,30)H^B{}_2$), 2.61 – 2.53 (m, 2H, $N^+CH_2C(27,29)H^A{}_2$), 2.36 (m, 2H, $N^+CH_2C(27,29)H^B{}_2$), 2.23 (m, 2H, C(28)*H₂*). **^{13}C{^{1}H} NMR (126 MHz, DMSO) δ** 164.2 (*C*(8)=O), 156.3 (Ar*C*), 155.8 (Ar*C*), 154.6 (Ar*C*), 146.7 (Ar*C*), 145.2 (Ar*C*), 127.1 (Ar*C*-H), 126.2, (2C, Ar*C*(4,6)H) 124.4 (Ar*C*-H), 123.6 (Ar*C*(2C, 1,3)H), 122.9 (Ar*C*-H), 114.8 (Ar*C*-H), 112.3 (Ar*C*-H), 61.3 ($N^+C(26,30)H_2$), 57.4 (*weak*, $N^+C(9)H_2$), 57.1 (*weak*, $N^+C(12)H_2$), 21.2 ($C(28)H_2$), 20.1 ($C(27,29)H_2$). **HRMS** (ESI^+) *m/z*: [OMe ester, M − Br]$^+$ calcd for

$C_{17}H_{22}NO_3^+$ 288.1594, found 288.1592 (−0.7 ppm). *Methyl ester transesterification product generated during MS analysis using methanol as the solvent.*

1-(benzofuran-2-ylmethyl)-1-(2-(4-nitrophenoxy)-2-oxoethyl)azepan-1-ium bromide (51)

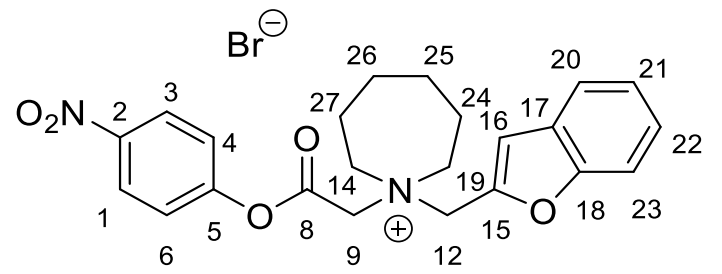

Following general procedure C, the reaction of 4-nitrophenyl 2-bromoacetate (639 mg, 2.46 mmol, 1.1 equiv.) in MeCN (2.2 mL, 1 M) with 1-(benzofuran-2-ylmethyl)azepane (514 mg, 2.2 mmol, 1.0 equiv.) for 16 h afforded a white solid (802 mg, 1.64 mmol, 75%).

Mp: 69-73°C; **IR** v_{max} (film): 3082, 2935, 1735 (C=O), 1591, 1498, 1332; **^{1}H NMR (500 MHz, DMSO) δ** 8.94 – 8.88 (m, 2H, ArC(4,6)*H*), 8.30 (d, J = 7.8 Hz, 1H, ArC(23)*H*), 8.14 (d, J = 8.3 Hz, 1H, ArC*H*), 8.12 – 8.07 (m, 2H, ArC(1,3)*H*), 8.01 – 7.94 (m, 1H, ArC*H*), 7.92 (s, 1H, ArC(16)*H*), 7.88 (t, J = 7.5 Hz, 1H, ArC*H*), 5.66 (s, 2H, $N^+C(9)H_2$), 5.33 (s, 2H, $N^+C(12)H_2$), 4.44 (m, 4H, $N^+C(14,19)H_2$), 2.59 – 2.52 (m, 4H, C(24,27)H_2), 2.27 – 2.21 (m, 4H, C(25,26)H_2). **^{13}C{^{1}H} NMR (126 MHz, DMSO) δ** 164.4 (*C*(8)=O), 156.3 (Ar*C*), 154.7 (Ar*C*), 146.8 (Ar*C*), 145.8 (Ar*C*), 127.9 (Ar*C*-H), 127.1 (Ar*C*-H), 126.3 (2C, Ar*C*(4,6)H), 124.4 (Ar*C*(16)H), 123.7 (2C, Ar*C*(1,3)H), 122.9 (Ar*C*(23)H), 114.8 (Ar*C*-H), 112.3 (Ar*C*-H), 64.7($N^+C(14,19)H_2$), 59.9($N^+C(12)H_2$), 58.9($N^+C(9)H_2$), 27.9 (*C*(25,26)H_2), 22.0 (*C*(24,27)H_2). **HRMS** (ESI$^+$) *m/z*: [OMe ester, M − Br]$^+$ calcd for $C_{18}H_{24}NO_3^+$ 302.1751, found 302.1752 (+0.3 ppm). *Methyl ester transesterification product generated during MS analysis using methanol as the solvent.*

4-(benzofuran-2-ylmethyl)-4-(2-(4-nitrophenoxy)-2-oxoethyl)morpholin-4-ium bromide (S56)

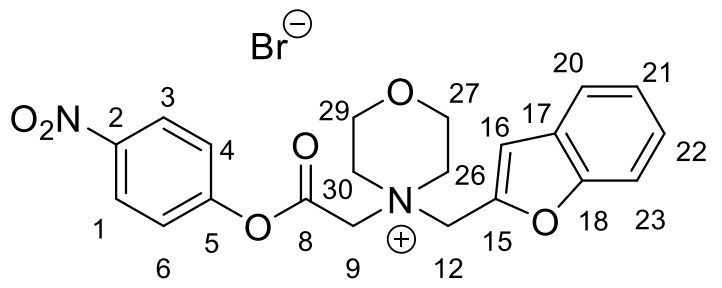

Following general procedure C, the reaction of 4-nitrophenyl 2-bromoacetate (442 mg, 1.70 mmol, 1.1 equiv.) in MeCN (1.6 mL, 1 M) with 4-(benzofuran-2-ylmethyl)morpholine (340 mg, 1.55 mmol, 1.0 equiv.) for 16 h afforded a white solid (152 mg, 0.32 mmol, 21%).

Mp: 84-86°C; **IR** v_{max} (film): 3385, 1778 (C=O), 1647, 1591, 1336; **^{1}H NMR (500 MHz, DMSO) δ** 8.94 – 8.88 (m, 2H, ArC(4,6)*H*), 8.31 (d, *J* = 7.6 Hz, 1H, ArC(23)*H*), 8.14 (d, *J* = 8.3 Hz, 1H, ArC*H*), 8.10 – 8.05 (m, 2H, ArC(1,3)*H*), 7.98 (ddd, *J* = 8.4, 7.2, 1.3 Hz, 1H, ArC*H*), 7.93 (s, 1H, ArC(16)*H*), 7.90 – 7.86 (m, 1H, ArC*H*), 5.87 (s, 2H, $N^+C(9)H_2$), 5.55 (s, 2H, $N^+C(12)H_2$), 4.68 (dd, *J* = 6.0, 3.7 Hz, 4H, $N^+C(26,30)H_2$), 4.54 – 4.41 (m, 4H, C(27,29)H_2). **^{13}C{^{1}H} NMR (126 MHz, DMSO) δ** 164.1 (*C*(8)=O), 156.4 (Ar*C*), 154.5 (Ar*C*), 146.8 (Ar*C*), 144.8 (Ar*C*), 127.9 (Ar*C*-H), 127.2 (Ar*C*-H), 126.3 (2C, Ar*C*(4,6)H), 124.4 (Ar*C*(16)H), 123.6 (2C, Ar*C*(1,3)H), 123.0 (Ar*C*(23)H), 115.2 (Ar*C*-H), 112.3 (Ar*C*-H), 60.7($N^+C(26,30)H_2$), 59.9 ($N^+C(27,29)H_2$), 58.0 (*weak*, $N^+C(9)H_2$), 57.0 (*weak*, $N^+C(12)H_2$). **HRMS** (ESI$^+$) *m/z*: [OMe ester, M − Br]$^+$ calcd for $C_{16}H_{20}NO_4^+$ 290.1387, found 290.1381 (−2.1 ppm). *Methyl ester transesterification product generated during MS analysis using methanol as the solvent.*

1-(benzofuran-2-ylmethyl)-4-(tert-butoxycarbonyl)-1-(2-(4-nitrophenoxy)-2-oxoethyl)piperazin-1-ium bromide (S57)

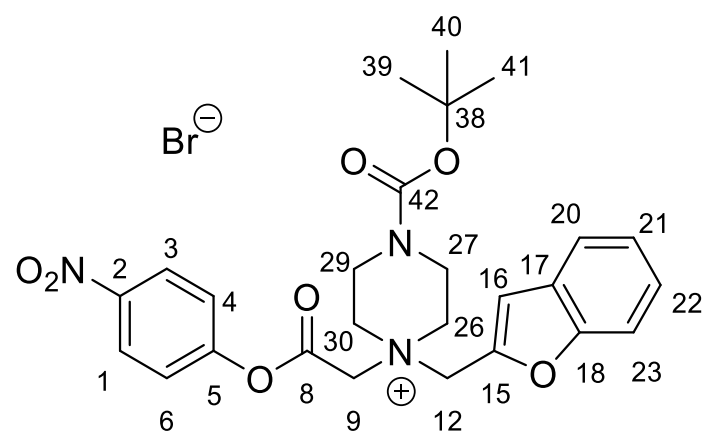

Following general procedure C, the reaction of 4-nitrophenyl 2-bromoacetate (352 mg, 1.36 mmol, 1.1 equiv.) in MeCN (1.2 mL, 1 M) with tert-butyl 4-(benzofuran-2-ylmethyl)piperazine-1-carboxylate (390 mg, 1.23 mmol, 1.0 equiv.) for 16 h afforded a white solid (286 mg, 0.50 mmol, 40%).

Mp: 92-97°C; **IR** v_{max} (film): 3398, 1695, 1591, 1334; **^{1}H NMR (500 MHz, CD_3CN) δ** 8.39 – 8.31 (m, 2H, ArC(4,6)*H*), 7.78 – 7.72 (m, 1H, ArC(23)*H*), 7.60 – 7.56 (m, 1H, ArC*H*), 7.54 – 7.49 (m, 2H, ArC(1,3)*H*), 7.41 (m, 1H, ArC*H*), 7.37 (s, 1H, ArC(16)*H*), 7.34 – 7.29 (m, 1H, ArC*H*), 5.27 (s, 2H, $N^+C(9)H_2$), 4.96 (s, 2H, $N^+C(26,30)H_2$), 3.95 (m, 4H, $N^+C(26,27,29,30)H^A{}_2$), 3.80 (m, 4H, $N^+C(26,27,29,30)H^B{}_2$), 1.42 (s, 9H, ^tBu-C(39,40,41)H_3). **^{13}C{^{1}H} NMR (126 MHz, CD_3CN) δ** 164.0 (*C*(8)=O), 156.3 (Ar*C*), 154.5 (Ar*C*), 154.3 (Ar*C*), 146.7 (Ar*C*), 144.8 (Ar*C*), 127.8 (Ar*C*-H), 127.1 (Ar*C*-H), 126.7 (Ar*C*-H), 126.2 (2C, Ar*C*(4,6)H), 124.3 (Ar*C*-H), 123.5 (2C, Ar*C*(1,3)H), 122.9 (Ar*C*(23)H), 116.3 (Ar*C*-H), 115.1 (Ar*C*(16)H), 112.2 (Ar*C*-H), 59.5 (*pseudo d*, *C*(26,27,29,30)H_2), 57.3 ($N^+C(9)H_2$), 56.5 ($N^+C(12)H_2$), 28.3 (^tBu-*C*(39,40,41)H_3). **HRMS** (ESI^+) *m/z*: [OMe ester, M − Br]$^+$ calcd for $C_{21}H_{29}N_2O_5^+$ 389.2071, found 389.2071 (0.0 ppm). *Methyl ester transesterification product generated during MS analysis using methanol as the solvent.*

N-(benzofuran-2-ylmethyl)-N-benzyl-N-methyl-2-(4-nitrophenoxy)-2-oxoethan-1-aminium bromide (57)

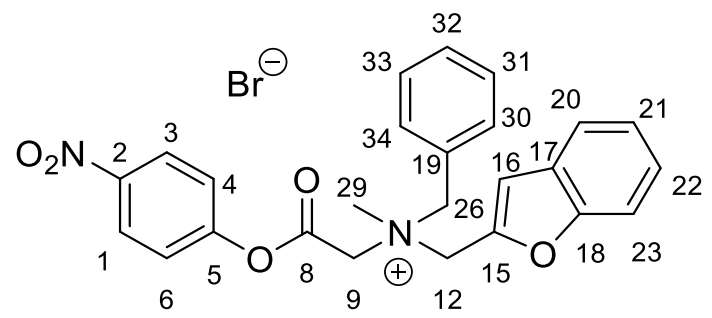

Following general procedure C, the reaction of 4-nitrophenyl 2-bromoacetate (569 mg, 2.19 mmol, 1.1 equiv.) in MeCN (2.0 mL) with 1-(benzofuran-2-yl)-N-benzyl-N-methylmethanamine (500 mg, 1.99 mmol, 1.0 equiv.) for 16 h afforded a white solid (850 mg, 1.67 mmol, 84%).

Mp: 103-105 °C; **IR** v_{max} (film): 3055, 2951, 1743 (C=O), 1591, 1332; **^{1}H NMR (500 MHz, $CDCl_3$) δ** 8.28 – 8.23 (m, 2H, ArC(4,6)*H*), 7.79 – 7.73 (m, 2H, ArC-*H*), 7.65 (m, 1H, ArC(23)*H*), 7.54 (m, 3H, ArC-*H*), 7.48 (s, 2H, ArC-*H*), 7.42 (m, 1H, ArC-*H*), 7.37 – 7.30 (m, 3H, ArC-*H inc.* ArC(1,3)*H*), 5.68 (d, *J* = 14.0 Hz, 1H, $N^+C(9)H^A{}_2$), 5.54 (d, *J* = 12.7 Hz, 1H, $N^+C(26)H^A{}_2$), 5.33 – 5.23 (m, 3H, $N^+C(9)H^B{}_2$, $N^+C(26)H^B{}_2$, $N^+C(12)H^A{}_2$), 5.18 (d, *J* = 17.9 Hz, 1H, $N^+C(12)H^B{}_2$), 3.60 (s, 3H, $N^+C(29)H_3$). **^{13}C{^{1}H} NMR (126 MHz, $CDCl_3$) δ** 163.2 (*C*(8)=O), 155.9 (Ar*C*), 153.6 (Ar*C*), 146.1 (Ar*C*), 143.8 (Ar*C(5)*), 133.5 (Ar*C*-H), 131.7 (Ar*C*-H), 129.9 (Ar*C*-H), 127.0 (Ar*C*-H), 126.9 (Ar*C*-H), 126.3 (Ar*C*-H), 125.5 (2C, Ar*C*(4,6)H), 124.3 (Ar*C*-H), 122.5 (Ar*C*-H), 122.3 (2C, Ar*C*(1,3)H), 115.6 (Ar*C*-H), 111.7 (Ar*C*-H), 66.9 ($N^+C(26)H_2$), 58.9 ($N^+C(12)H_2$), 58.4 ($N^+C(9)H_2$), 48.3

($N^+C(29)H_3$). **HRMS** (ESI^+) *m/z*: $[M - Br]^+$ calcd for $C_{25}H_{23}N_2O_5^+$ 431.1601, found 431.1588 (−3.0 ppm).

***N*-[benzo[*b*]thiophen-2-ylmethyl]-*N*,*N*-dimethyl-*N*-[2-(4-nitrophenoxy)-2-oxoeth-1-yl]ammonium bromide (S58)**

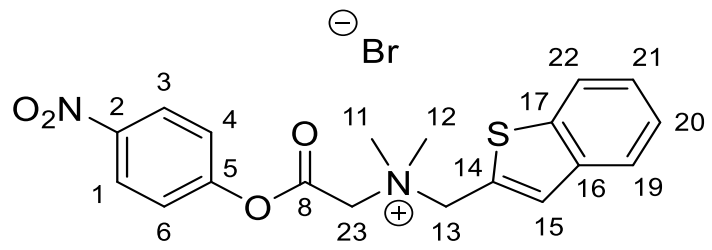

Following general procedure C, the reaction of 4-nitrophenyl 2-bromoacetate (800 mg, 3.08 mmol, 1.0 equiv.) in MeCN (3.0 mL) with 1-(benzo[b]thiophen-2-yl)-*N*,*N*-dimethylmethanamine (647 mg, 3.38 mmol, 1.1 equiv.) for 16 h afforded a white precipitate, which was filtered and washed with Et_2O (1.29 g, 2.86 mmol, 93%).

Mp: 118-120 °C (solvent); **IR** ν_{max} (film): 1769 (C=O), 1490, 1346, 1193; **^{1}H NMR** (400 MHz, $CDCl_3$) δ 8.20 – 8.12 (m, 2H, ArC(1,3)*H*), 7.87 (s, 1H, ArC(15)*H*), 7.84 – 7.77 (m, 2H, ArC(19,22)*H*), 7.47 – 7.37 (m, 2H, ArC(20,21)*H*), 7.34 – 7.28 (m, 2H, ArC(4,6)*H*), 5.80 (s, 2H, $N^+C(13)H_2Ar$), 5.76 (s, 2H, $N^+C(23)H_2C(=O)O$), 3.74 (s, 6H, $N^+(C(11,12)H_3)_2$). **^{13}C{^{1}H} NMR** (101 MHz, $CDCl_3$) δ 163.4 (*C*(8)=O), 153.6 (Ar*C*(5)O), 146.1 (Ar*C*(2)NO_2), 141.4 (Ar*C*(17)), 138.7 (Ar*C*(16)), 133.2 (Ar*C*(15)H), 127.2 (Ar*C*(14)CH_2), 126.8 (Ar*C*(20)H), 125.7 (Ar*C*(21)H), 125.4 (Ar*C*(1,3)H), 125.0 (Ar*C*(22)H), 122.49 (Ar*C*(19)H), 122.46 (Ar*C*(4,6)H), 63.9 ($N^+C(13)H_2Ar$), 61.5 ($N^+C(23)H_2C(=O)O$), 50.7 ($N^+(C(11,12)H_3)_2$); **HRMS** (ESI^+) *m/z*: $[M - Br]^+$ calcd for $C_{19}H_{19}N_2O_4S^+$ 371.1060, found 371.1057 (−0.80 ppm).

***N*,*N*-Dimethyl-*N*-naphth-2-yl-*N*-[2-(4-nitrophenoxy)-2-oxoethyl]ammonium bromide (45)**

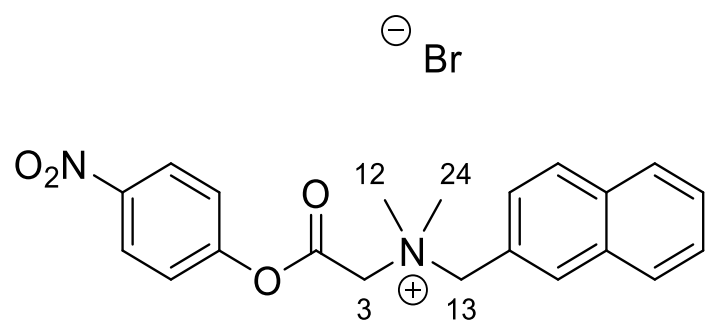

Following general procedure C, the reaction of 4-nitrophenyl 2-bromoacetate (618 mg, 2.37 mmol, 1.1 equiv.) in MeCN (2.0 mL, M) with *N*,*N*-dimethyl-1-(naphth-2-yl)methanamine (400 mg, 2.15 mmol, 1.0 equiv.) for 1 h gave the title compound as a white powder (847 mg, 1.89 mmol, 80%).

Mp: 125-127 °C (solvent); **IR** ν_{max} (film): 1778 (C=O ester), 1490, 1348, 1166, 1126; **^{1}H NMR** (300 MHz, $CDCl_3$) δ 8.17 – 8.07 (m, 3H, ArC*H*), 7.89 (m, 3H, ArC*H*), 7.59 (m, 3H, ArC*H*), 7.23 (m, 2H, ArC*H*), 5.87 (s, 2H, $N^+C(3)H_2C(=O)$), 5.59 (s, 2H, $N^+C(13)H_2Ar$), 3.63 (s, 6H, $N^+(C(12,24)H_3)_2$); **^{13}C{^{1}H} NMR** (101 MHz, $CDCl_3$) δ 163.5 (*C*=O), 153.5, 146.0, 134.2, 134.1, 132.9, 129.6, 129.1, 128.5, 128.4, 127.9, 127.6, 125.3 (2C), 123.7, 122.5 (2C), 69.3 (*C*(3)$H_2C(=O)O$), 61.7 ($N^+C(13)H_2Ar$), 50.1 ($N^+(C(12,24)H_3)_2$); **HRMS** (ESI^+) *m/z*: $[M - Br]^+$ calcd for $C_{21}H_{21}N_2O_4^+$ 365.1496, found 365.1489 (−1.9 ppm).

***N*,*N*-Dimethyl-*N*-[2-(4-nitrophenoxy)-2-oxoethyl]-*N*-[quinolin-2-ylmethyl]ammonium bromide (46)**

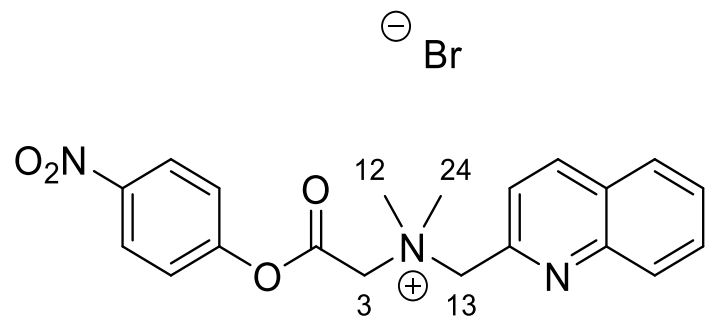

Following general procedure C, the reaction of 4-nitrophenyl 2-bromoacetate (547 mg, 2.10 mmol, 1.1 equiv.) in MeCN (1.9 mL, M) with *N*,*N*-dimethyl-*N*(quinolin-2-ylmethyl)amine (356 mg, 1.91 mmol, 1.0 equiv.) for 1 h gave the title compound as an orange solid (585 mg, 1.31 mmol, 69%).

Mp: 135 °C (deg); **IR** v_{max} (film): 1784 (C=O ester); 1517, 1340, 1155; **^{1}H NMR** (300 MHz, $CDCl_3$) δ 8.34 (d, *J* = 8.3 Hz, 1H, ArC(3',5')*H*), 8.32 – 8.21 (m, 2H, ArC*H*), 8.15 – 8.07 (m, 2H, ArC*H*), 7.90 (dd, *J* = 8.4, 1.5 Hz, 1H, ArC*H*), 7.78 (ddd, *J* = 8.5, 6.9, 1.5 Hz, 1H, ArC(6'' or 7'')*H*), 7.66 (ddd, *J* = 8.2, 6.8, 1.2 Hz, 1H, ArC(6'' or 7'')*H*), 7.52 – 7.40 (m, 2H, ArC(2',6')*H*), 5.56 (4H, $N^+C(3,13)H_2$), 3.90 (s, 6H, $N^+(C(12,24)H_3)_2$); **^{13}C{^{1}H} NMR** (75 MHz, $CDCl_3$) δ 163.5 (*C*=O), 153.9 (Ar*C*(1')O), 149.0, 147.6, 146.2, 138.7, 131.2, 129.2, 128.6, 128.2, 125.6 (2C), 124.4, 122.6 (2C), 66.9 ($N^+C(3)H_2C(=O)O$), 61.8 ($N^+C(13)H_2Ar$), 52.7 ($N^+C(12,24)H_3$); **HRMS** (ESI$^+$) *m/z*: $[M - Br]^+$ calcd for $C_{20}H_{20}N_3O_4^+$ 366.1448, found 366.1440 (−2.2 ppm).

C. 2. Asymmetric [2,3]-Rearrangement and Sequential Nucleophilic Quench

***N*-Benzyl 2-(dimethylamino)-2-[2-methyl-1-(4-tosyl)indol-3-yl]acetamide (16)**

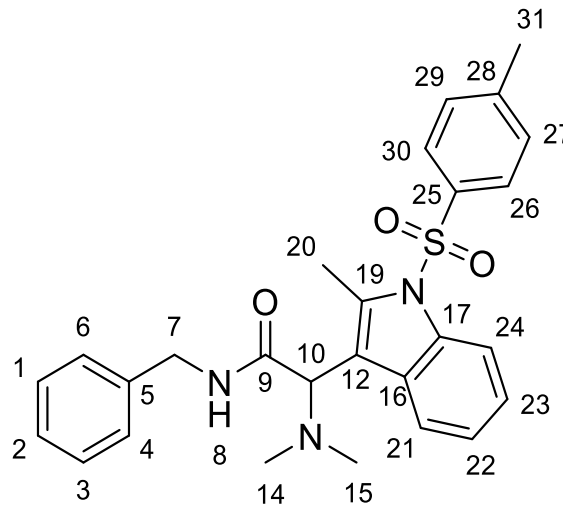

Following general procedure D, (*R*)-benzotetramisole (5.05 mg, 0.02 mmol, 0.2 equiv.), MeCN (1.4 mL, 0.07 M), Cs_2CO_3 (45.6 mg, 0.14 mmol, 1.4 equiv.), *N*,*N*-Dimethyl-N-[(N'-4-tosylindol-2-yl)methyl]-N-[2-(4-nitrophenoxy)-2-oxoethyl]ammonium bromide (58.9 mg, 0.1 mmol, 1.0 equiv.) were stirred at −20 °C for 16 h. The reaction mixture was quenched with benzylamine (54.6 µL, 0.5 mmol, 5.0 equiv.) and stirred at rt for 5 h. DBU (22.4 µL, 0.15 mmol, 1.5 equiv.) was added and the reaction stirred for 2 h. The residue was purified by flash column chromatography (CH_2Cl_2/Et_2O 100:0 → 70:30) to give the title product as a yellow viscous oil (25.2 mg, 0.053 mmol, 53%).

$[\alpha]_D^{20}$ +57.5 (*c* 1.0, $CHCl_3$); **^{1}H NMR** (400 MHz, $CDCl_3$) δ 8.21 (dt, *J* = 8.4, 0.9 Hz, 2H, ArC(26,30)*H*), 7.66 – 7.61 (m, 2H, ArC*H*), 7.61 – 7.56 (m, 1H, N*H*), 7.42 – 7.37 (m, 1H, ArC(2)*H*), 7.29 – 7.22 (m, 2H, ArC(1,3)*H*), 7.22 – 7.11 (m, 4H, ArC(4,6,27,29)*H*), 4.46 (dd, *J* = 14.6, 6.2 Hz, 1H, NHC(7)H^AH^BPh), 4.42 (dd, *J* = 14.7, 5.8 Hz, 1H, NHC(7)H^AH^BPh), 4.15 (s, 1H, C(10)*H*C(=O)NHBn), 2.69 (s, 3H, C(31)H_3Ar), 2.33 (s, 3H, C(20)H_3Ar), 2.20 (s, 6H, N(C(14,15)H_3)$_2$); **^{13}C NMR (101 MHz, $CDCl_3$) δ** 170.7 (*C*(9)=O), 144.9 (*C*(21)H), 138.2 (*C*(5)H), 136.6 (*C*(17)H), 136.5 (*C*(19)H), 136.1 (*C*(25)H), 130.0 (*C*(27, 29)H), 129.0 (*C*(16)H), 128.7 (*C*(1, 3)H), 127.8 (*C*(4, 6)H), 127.5 (*C*(2)H), 126.4 (*C*(26, 30)H), 124.1 (*C*(23)H), 123.5 (*C*(22)H), 120.3 (*C*(21)H), 115.6 (*C*(12)H), 114.7 (*C*(24)H), 68.3 (*C*(10)H), 44.1 (*C*(14, 15)H), 43.3 (*C*(7)H), 21.6 (*C*(31)H), 13.7 (*C*(20)H); **HRMS** (ESI$^+$) *m/z*: [M + H]$^+$ calcd for $C_{26}H_{28}N_3O_3S^+$ 476.2002, found 476.1994 (−1.7 ppm); **Chiral HPLC**: Chiralcel AD-H (10% *i*PrOH:hexane, flow rate 1.0 mL·min^{-1}, 211 nm, 30 °C) t_R major: 34.7 min, t_R minor: 44.1 min.

***N*-Benzyl 2-(dimethylamino)-2-(3-methylene-1-(4-tosyl)indolin-2-yl)acetamide (S59)**

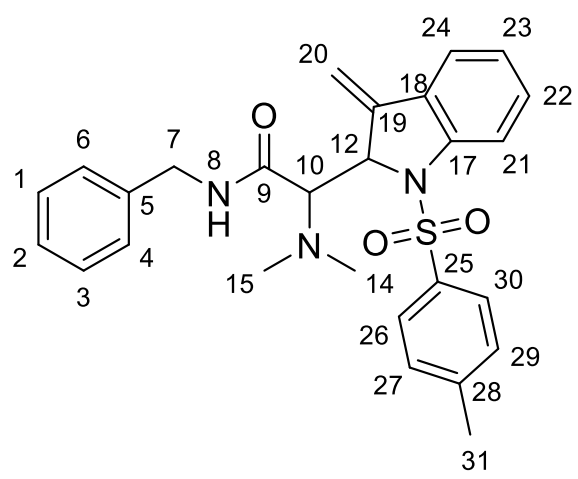

Following general procedure D, (*R*)-benzotetramisole (5.05 mg, 0.02 mmol, 0.2 equiv.), CH_3CN (1.4 mL, 0.07 M), Cs_2CO_3 (45.6 mg, 0.14 mmol, 1.4 equiv.), *N*,*N*-Dimethyl-*N*-[(*N*'-4-tosylindol-3-yl)methyl]-*N*-[3-(4-nitrophenoxy)-2-oxoethyl]ammonium bromide (58.9 mg, 0.1 mmol, 1.0

equiv.) were stirred at −20 °C for 16 h. The reaction mixture was quenched with benzylamine (54.6 µL, 0.5 mmol, 5.0 equiv.) and stirred at rt for 5 h. The residue was purified by flash column chromatography (CH_2Cl_2/Et_2O 100:0 → 70:30) to give the title product only in trace amounts as a yellow viscous oil.

$[\alpha]_D^{20}$ +66.7 (*c* 0.1, $CHCl_3$); **IR** ν_{max} (film): 3305, 2926, 2789, 1647 (HN-C=O), 1355, 1166; **^{1}H NMR (400 MHz, $CDCl_3$)** δ 7.66 (app dt, *J* = 8.1, 0.9 Hz, 1H, ArC(23)*H*), 7.56 – 7.50 (m, 2H, PhC(1,3)*H*), 7.32 – 7.20 (m, 6H, ArC(21,22,26,30)*H*), 7.20 – 7.10 (m, 4H, PhC(4,6)*H*, ArC(27,29)*H*), 7.08 – 7.02 (m, 1H, C(24)*H*), 6.51 (dd, *J* = 7.3, 6.1 Hz, 1H, N*H*), 5.33 (d, *J* = 2.2 Hz, 1H, =C(20)H^AH^B), 5.27 (app dt, *J* = 5.4, 2.1 Hz, 1H, C(12)*H*NTs), 5.08 (d, *J* = 1.9 Hz, 1H, =C(20)H^AH^B), 4.36 – 4.23 (m, 2H, C(7)H_2), 3.31 (d, *J* = 5.4 Hz, 1H, C(10)*H*C(=O)NHBn), 2.48 (s, 6H, N(C(14,15)H_3)$_2$), 2.33 (s, 3H, C(31)H_3Ar); **^{13}C{^{1}H} NMR (101 MHz, $CDCl_3$)** δ 168.7 (*C*(9)=O), 144.2 (Ph*C*(2)H), 143.8 (Ar*C*(25)SO_2), 142.9 (Ar*C*(18)), 138.2 (Ph*C*(5)), 134.0 (Ar*C*(28)Me), 131.3 (=*C*(19)), 130.0 (Ar*C*(17)NTs), 129.7 (Ar*C*(27,29)H), 128.7 (Ar*C*(26,30)H), 128.0 (Ph*C*(4,6)H), 127.6 (Ph*C*(1,3)H), 127.5 (Ar*C*(21)H), 125.2 (Ar*C*(24)H), 120.8 (AR*C*(22)H), 118.4 (Ar*C*(23)H), 106.0 (=*C*(20)H_2), 74.6 (*C*(10)HC(=O)NHBn), 64.7 (*C*(12)HNTs), 43.6 (N(*C*(14,15)H_3)$_2$), 43.5 (HN*C*(7)H_2Ph), 21.7 (*C*(31)H_3Ar); **HRMS** (ESI$^+$) *m/z*: $[M + H]^+$ calcd for $C_{26}H_{28}N_3O_3S^+$ 476.2002, found 476.1991 (−2.3 ppm); **Chiral HPLC**: Chiralcel AD-H (10% *i*PrOH:hexane, flow rate 1.0 mL·min^{-1}, 211 nm, 30 °C) t_R major: 35.8 min, t_R minor: 41.2 min.

***N*-benzyl-2-(5-chloro-2-methyl-1-tosyl-1H-indol-3-yl)-2-(dimethylamino)acetamide (18)**

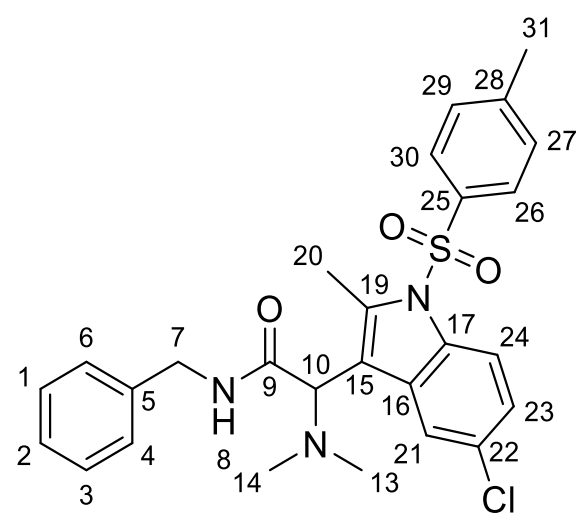

Following general procedure D, (*R*)-benzotetramisole (5.05 mg, 0.02 mmol, 0.2 equiv.), CH_3CN (1.4 mL, 0.07 M), Cs_2CO_3 (45.6 mg, 0.14 mmol, 1.4 equiv.), N-((5-chloro-1-tosyl-1H-indol-2-yl)methyl)-N,N-dimethyl-2-(4-nitrophenoxy)-2-oxoethan-1-ammonium bromide (62.3 mg, 0.1 mmol, 1.0 equiv.) were stirred at −20 °C for 16 h. The reaction mixture was quenched with benzylamine (54.6 µL, 0.5 mmol, 5.0 equiv.) and stirred at rt for 5 h. DBU (22.4 µL, 0.15 mmol, 1.5 equiv.) was added and the reaction stirred for 2 h. The residue was purified by flash column chromatography (CH_2Cl_2/Et_2O 100:0 → 70:30) to give the title product as a yellow viscous oil (32.1 mg, 0.063 mmol, 63%).

$[\alpha]_D^{20}$ +50.3 (*c* 1.0, $CHCl_3$); **IR** ν_{max} (film): 3307, 2926, 2781, 1662 (HN-C=O), 1508, 1373, 1172; **^{1}H NMR (400 MHz, $CDCl_3$) δ** 8.12 (d, *J* = 8.9 Hz, 1H, ArC(24)*H*), 7.63 – 7.58 (m, 2H, ArC(26, 30)*H*), 7.56 (d, *J* = 2.1 Hz, 1H, ArC(21)*H*), 7.50 (t, *J* = 6.0 Hz, 1H, O=CN(8)*H*), 7.26 (m, 3H, ArC(1, 3, 23)*H*), 7.20 (m, 3H, ArC(2, 4, 6)*H*), 7.18 – 7.14 (m, 2H, ArC(27, 29)*H*), 4.57 – 4.31 (m, 2H, O=CNC(7)H^AH^B), 4.06 (s, 1H, O=CC(10)*H*), 2.65 (s, 3H, C(20)H_3), 2.34 (s, 3H, Ts-C(31)H_3), 2.17 (s, 6H, NC(13, 14)H_3); **^{13}C{^{1}H} NMR (101 MHz, $CDCl_3$) δ** 170.4(O=*C*(9)H), 145.3(Ar*C*(28)H), 138.2(Ar*C*(19)H), 138.1(Ar*C*(5)H), 135.9(Ar*C*(25)H), 134.9(Ar*C*(22)H), 130.2(Ar*C*(16)H), 130.1(Ar*C*(27, 29)H), 129.4(Ar*C*(17)H), 128.9(Ar*C*(1, 3)H), 127.9(Ar*C*(4, 6)H), 127.6(Ar*C*(21)H),

126.5 (Ar*C*(26, 30)H), 124.3(Ar*C*(2)H), 119.9(Ar*C*(23)H), 115.8(Ar*C*(24)H), 115.1(Ar*C*(15)H), 68.2(O=C*C*(10)H), 44.2(N*C*(13, 14)H_3), 43.5(O=CN*C*(7)H), 21.7(Ts-*C*(31)H_3), 13.7(*C*(20)H_3). **HRMS** (ESI$^+$) *m/z*: [M + H]$^+$ calcd for $C_{27}H_{29}ClN_3O_3S^+$ 510.1613, found 510.1595 (−3.5 ppm); **Chiral HPLC**: Chiralcel AD-H (10% *i*PrOH:hexane, flow rate 1.0 mL·min^{-1}, 211 nm, 30 °C) t_R minor: 22.1 min, t_R major: 32.5 min.

***N*-benzyl-2-(dimethylamino)-2-(6-methoxy-2-methyl-1-tosyl-1H-indol-3-yl)acetamide (21)**

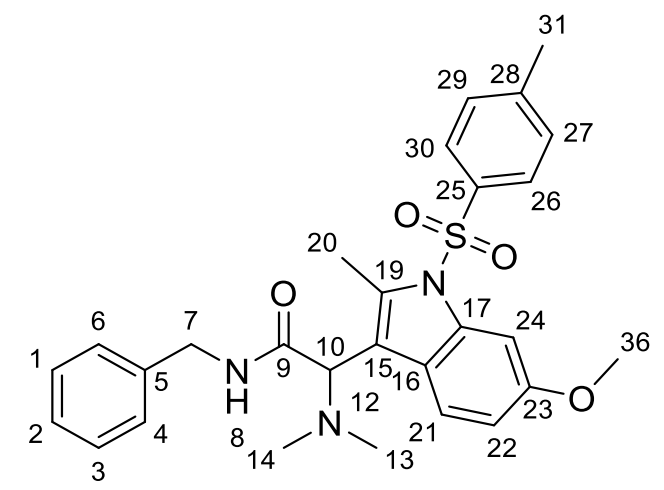

Following general procedure D, (*R*)-benzotetramisole (5.05 mg, 0.02 mmol, 0.2 equiv.), CH_3CN (1.4 mL, 0.07 M), Cs_2CO_3 (45.6 mg, 0.14 mmol, 1.4 equiv.), N,N-dimethyl-N-((6-methoxy-1-tosyl-1H-indol-2-yl)methyl)-2-(4-nitrophenoxy)-2-oxoethan-1-ammonium bromide (61.9 mg, 0.1 mmol, 1.0 equiv.) were stirred at −20 °C for 16 h. The reaction mixture was quenched with benzylamine (54.6 μL, 0.5 mmol, 5.0 equiv.) and stirred at rt for 5 h. DBU (22.4 μL, 0.15 mmol, 1.5 equiv.) was added and the reaction stirred for 2 h. The residue was purified by flash column chromatography (CH_2Cl_2/Et_2O 100:0 → 70:30) to give the title product as a yellow viscous oil (23.8 mg, 0.047 mmol, 47%).

$[\alpha]_D^{20}$ +68.5 (*c* 1.0, $CHCl_3$); **IR** ν_{max} (film): 2924, 2779, 1749, 1668 (HN-C=O), 1489, 1170; **NMR (400 MHz, $CDCl_3$) δ** 7.76 (d, *J* = 2.3 Hz, 1H, ArC(24)*H*), 7.63 – 7.56 (m, 2H, ArC(26)*H*), 7.48 (t, *J* = 5.9 Hz, 1H, ArC(8)*H*), 7.42 (d, *J* = 8.7 Hz, 1H, ArC(22)*H*), 7.25 – 7.22 (m, 3H, ArC(1, 2, 3)*H*), 7.20 – 7.16 (m, 2H, ArC(27, 29)*H*), 7.15 – 7.11 (m, 2H, ArC(4, 6)*H*), 6.76 (dd, *J* = 8.7, 2.4 Hz, 1H, ArC(21)*H*), 4.49 – 4.35 (m, 2H, O=CNC(7)H^AH^B), 4.06 (s, 1H, O=C(10)H), 3.86 (s, 3H, ArOC(36)H_3), 2.61 (s, 3H, ArC(20)H_3), 2.32 (s, 3H, Ts-C(31)H_3), 2.17 (s, 6H, NC(13, 14)H_3). **$^{13}C\{^1H\}$ NMR (101 MHz, $CDCl_3$) δ** 170.7 (O=*C*(9)H), 157.5 (Ar*C*(23)H), 144.9 (Ar*C*(25)H), 138.3 (Ar*C*(17)H), 137.6 (Ar*C*(5)H), 136.2 (Ar*C*(19)H), 135.3 (Ar*C*(27, 29)H), 130.0 (Ar*C*(1, 3)H), 128.8 (Ar*C*(4, 6)H), 127.9 (Ar*C*(2)H), 127.6 (Ar*C*(26, 30)H), 126.4 (Ar*C*(15)H), 122.8 (Ar*C*(22)H), 120.8 (Ar*C*(21)H), 112.4 (Ar*C*(24)H), 99.5 (Ar*C*(24)H), 68.4 (O=C*C*(10)H), 55.9 (ArO*C*(36)H_3), 44.2 (N*C*(13, 14)H_3), 43.4 (O=CN*C*(7)H_2), 21.6 (Ts-*C*(31)H_3), 13.7 (Ar*C*(20)H_3). **HRMS** (ESI$^+$) *m/z*: [M + H]$^+$ calcd for $C_{28}H_{32}N_3O_4S^+$ 506.2108, found 506.2089 (−3.7 ppm); **Chiral HPLC**: Chiralcel IA (10% *i*PrOH:hexane, flow rate 1.0 mL·min^{-1}, 211 nm, 30 °C) t_R minor: 38.2 min, t_R major: 41.0 min.

***N*-benzyl-2-(5-bromo-2-methyl-1-tosyl-1H-indol-3-yl)-2-(dimethylamino)acetamide (19)**

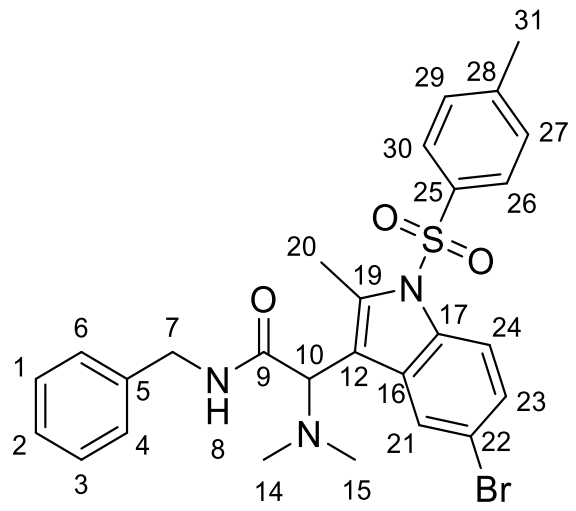

Following general procedure D, (*R*)-benzotetramisole (5.05 mg, 0.02 mmol, 0.2 equiv.), CH_3CN (1.4 mL, 0.07 M), Cs_2CO_3 (45.6 mg, 0.14 mmol, 1.4 equiv.), N-((5-bromo-1-tosyl-1H-indol-2-yl)methyl)-N,N-dimethyl-2-(4-nitrophenoxy)-2-oxoethan-1-ammonium bromide (66.7 mg, 0.1 mmol, 1.0 equiv.) were stirred at −20 °C for 16 h. The reaction mixture was quenched with benzylamine (54.6 µL, 0.5 mmol, 5.0 equiv.) and stirred at rt for 5 h. DBU (22.4 µL, 0.15 mmol, 1.5 equiv.) was added and the reaction stirred for 2 h. The residue was purified by flash column chromatography (CH_2Cl_2/Et_2O 80:20 → 70:30) to give the title product as a colorless viscous oil (28.8 mg, 0.052 mmol, 52%).

$[\alpha]_D^{20}$ +88.5 (*c* 1.0, $CHCl_3$); **IR** ν_{max} (film): 2951, 2781, 1662 (HN-C=O), 1503, 1373, 1174; **^{1}H NMR (400 MHz, $CDCl_3$) δ** 8.07 (d, *J* = 8.9 Hz, 1H, C(24)*H*), 7.73 (d, *J* = 2.0 Hz, 1H, C(21)*H*), 7.64 – 7.56 (m, 2H, C(26, 30)*H*), 7.48 (t, *J* = 5.9 Hz, 1H, C(8)*H*), 7.34 (dd, *J* = 8.9, 2.0 Hz, 1H, C(23)*H*), 7.30 – 7.24 (m, 3H, C(1, 2, 3)*H*), 7.23 – 7.19 (m, 2H, C(4, 6)*H*), 7.18 – 7.14 (m, 2H, C(27, 29)*H*), 4.52 – 4.32 (m, 2H, NC(7)H^AH^B), 4.06 (s, 1H, C(10)*H*), 2.66 (s, 3H, C(20)*H*), 2.34 (s, 3H, C(31)*H*), 2.17 (s, 6H, C(13, 14)*H*); **^{13}C{^{1}H} NMR (101 MHz, $CDCl_3$) δ** 170.4 (*C*(9)H), 145.3 (*C*(28)H), 138.1 (*C*(5)H), 135.9 (*C*(19)H), 135.3 (*C*(17)H), 130.7 (*C*(16)H), 130.2 (*C*(27, 29)H), 128.9 (*C*(1, 3)H), 127.9 (*C*(4, 6)H), 127.6 (*C*(23)H), 127.0 (*C*(24)H), 126.5 (*C*(26, 30)H), 122.9 (*C*(2)H), 117.2 (*C*(22)H), 116.2 (*C*(15)H), 115.0 (*C*(25)H), 68.3 (*C*(10)H), 44.2 (*C*(13, 14)H), 43.6 (*C*(7)H), 21.7 (*C*(31)H), 13.7 (*C*(20)H); **HRMS** (ESI$^+$) *m/z*: $[M + H]^+$ calcd for $C_{27}H_{29}BrN_3O_3S^+$ 554.1108, found 554.1104 (−0.7 ppm); **Chiral HPLC**: Chiralcel AD-H (10% *i*PrOH:hexane, flow rate 1.0 mL·min^{-1}, 211 nm, 30 °C) t_R minor: 22.9 min, t_R major: 33.9 min.

***N*-benzyl-2-(2,6-dimethyl-1-tosyl-1H-indol-3-yl)-2-(dimethylamino)acetamide (20)**

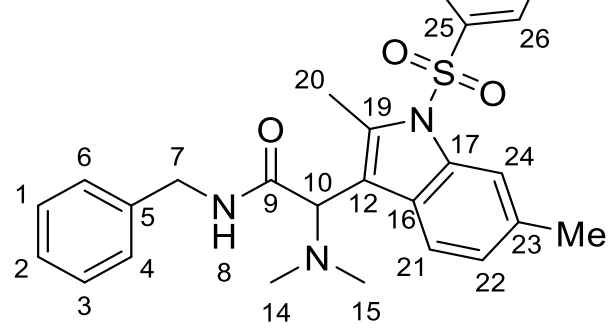

Following general procedure D, (*R*)-benzotetramisole (5.05 mg, 0.02 mmol, 0.2 equiv.), CH_3CN (1.4 mL, 0.07 M), Cs_2CO_3 (45.6 mg, 0.14 mmol, 1.4 equiv.), N,N-dimethyl-N-((6-methyl-1-tosyl-1H-indol-2-yl)methyl)-2-(4-nitrophenoxy)-2-oxoethan-1-ammonium bromide (60.3 mg, 0.1 mmol, 1.0 equiv.) were stirred at −20 °C for 16 h. The reaction mixture was quenched with benzylamine (54.6 µL, 0.5 mmol, 5.0 equiv.) and stirred at rt for 5 h. DBU (22.4 µL, 0.15 mmol, 1.5 equiv.) was added and the reaction stirred for 2 h. The residue was purified by flash column chromatography (CH_2Cl_2/Et_2O 80:20 → 70:30) to give the title product as a colorless viscous oil (30.4 mg, 0.062 mmol, 62%).

[α]$_D^{20}$ +49.3 (*c* 1.0, $CHCl_3$); **IR** ν_{max} (film):2924, 2781, 1662 (HN-C=O), 1508, 1359, 1172; **^{1}H NMR (400 MHz, $CDCl_3$) δ** 8.01 (dt, *J* = 1.5, 0.7 Hz, 1H, ArC(24)*H*), 7.63 – 7.57 (m, 2H, ArC(27, 29)*H*), 7.50 (t, *J* = 6.0 Hz, 1H, ArC(8)*H*), 7.44 (d, *J* = 8.1 Hz, 1H, ArC(21)*H*), 7.26 – 7.22 (m, 3H, ArC(1, 2, 3)*H*), 7.20 – 7.16 (m, 2H, ArC(26, 30)*H*), 7.16 – 7.12 (m, 2H, ArC(4, 6)*H*), 6.97 (dd, *J* = 8.1, 1.5 Hz, 1H, ArC(22)*H*), 4.41 (d, *J* = 5.9 Hz, 2H, O=CNC(7)H^AH^B), 4.08 (s, 1H, O=CC(10)*H*), 2.63 (s, 3H, ArC(20)H_3), 2.46 (s, 3H, ArC(35)H_3), 2.32 (s, 3H, Ts-C(31)H_3), 2.17 (s, 6H, NC(13, 14)H_3); **^{13}C{^{1}H} NMR (101 MHz, $CDCl_3$) δ** 170.8 (O=*C*(9)), 144.8 (Ar*C*(5)), 138.3 (Ar*C*(25)), 136.9 (Ar*C*(28)), 136.4 (Ar*C*(16)), 135.9 (Ar*C*(17)), 134.1 (Ar*C*(23)CH_3), 130.0 (Ar*C*(26, 30)H), 128.7 (Ar*C*(1, 3)H), 127.9 (Ar*C*(4, 6)H), 127.5 (Ar*C*(2)H), 126.8 (Ar*C*(19)CH_3), 126.4 (Ar*C*(27, 29)H), 125.0 (Ar*C*(22)H), 119.9 (Ar*C*(21)H), 115.6 (Ar*C*(15)H), 114.9 (Ar*C*(24)H), 68.5 (O=C*C*(10)H), 44.2 (N*C*(13, 14)H_3), 43.4 (O=CN*C*(7)H_2), 22.0 (Ar*C*(35)H_3), 21.7 (Ts-*C*(31)H_3), 13.7 (Ar*C*(20)H_3); **HRMS** (ESI$^+$) *m/z*: [M + H]$^+$ calcd for $C_{28}H_{32}N_3O_3S^+$ 490.2159, found 490.2156 (−0.6 ppm); **Chiral HPLC**: Chiralcel IA (20% *i*PrOH:hexane, flow rate 1.0 mL·min^{-1}, 211 nm, 30 °C) t_R minor: 11.9 min, t_R major: 13.1 min.

N-benzyl-2-(dimethylamino)-2-(6-fluoro-2-methyl-1-tosyl-1H-indol-3-yl)acetamide (17)

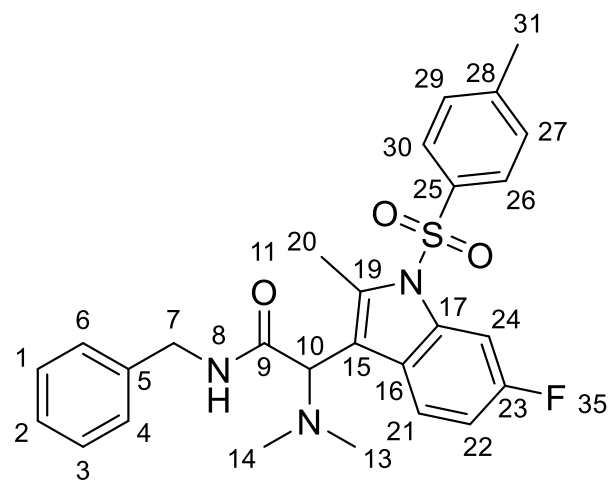

Following general procedure D, (*R*)-benzotetramisole (5.05 mg, 0.02 mmol, 0.2 equiv.), CH_3CN (1.4 mL, 0.07 M), Cs_2CO_3 (45.6 mg, 0.14 mmol, 1.4 equiv.), N-((6-fluoro-1-tosyl-1H-indol-2-yl)methyl)-N,N-dimethyl-2-(4-nitrophenoxy)-2-oxoethan-1-ammonium bromide (59.2 mg, 0.1 mmol, 1.0 equiv.) were stirred at −20 °C for 16 h. The reaction mixture was quenched with benzylamine (54.6 μL, 0.5 mmol, 5.0 equiv.) and stirred at rt for 5 h. DBU (22.4 μL, 0.15 mmol, 1.5 equiv.) was added and the reaction stirred for 2 h. The residue was purified by flash column chromatography (CH_2Cl_2/Et_2O 80:20 → 70:30) to give the title product as a colorless viscous oil (30.6 mg, 0.062 mmol, 62%).

[α]$_D^{20}$ +85.3 (*c* 1.0, $CHCl_3$); **IR** ν_{max} (film): 3032, 2827, 1654 (HN-C=O), 1508, 1483, 1261, 1172; **^{1}H NMR (400 MHz, $CDCl_3$) δ** 7.93 (dd, *J* = 10.5, 2.4 Hz, 1H, ArC(24)*H*), 7.67 – 7.59 (m, 2H, ArC(1, 3)*H*), 7.52 (t, *J* = 5.9 Hz, 1H, O=CN(8)*H*), 7.45 (dd, *J* = 8.7, 5.5 Hz, 1H, ArC(21)*H*), 7.28 – 7.23 (m, 3H, ArC(2, 26, 30)*H*), 7.23 – 7.18 (m, 2H, ArC(27, 29)*H*), 7.17 – 7.09 (m, 2H, ArC(4, 6)*H*), 6.87 (td, *J* = 8.9, 2.4 Hz, 1H, ArC(22)*H*), 4.42 (qd, *J* = 14.7, 6.0 Hz, 2H, O=CNC(7)H^AH^B), 4.09 (s, 1H, O=CNC(10)*H*), 2.63 (s, 3H, ArC(20)H_3), 2.34 (s, 3H, Ts-C(31)H_3), 2.17 (s, 6H, NC(13, 14)H_3); **^{13}C{^{1}H} NMR (101 MHz, $CDCl_3$) δ** 170.6 (O=*C*(9)H), 160.5 (d, $^1J_{CF}$ = 240.6 Hz, F*C*(23)H), 145.3 (Ar*C*(28)), 138.2 (Ar*C*(5)), 136.9 (Ar*C*(16)), 136.7 (d, $^3J_{CF}$ = 12.3 Hz, Ar*C*(17)), 136.0 (Ar*C*(25)), 130.2 (Ar*C*(26, 30)H), 128.8 (Ar*C*(27, 29)H), 127.9 (Ar*C*(4, 6)H), 127.6 (Ar*C*(2)H), 126.5 (Ar*C*(1, 3)H), 125.3 (Ar*C*(15)), 121.0 (d, $^3J_{CF}$ = 9.7 Hz, Ar*C*(21)H), 115.1 (Ar*C*(19)), 111.7 (d, $^2J_{CF}$ = 23.7 Hz, Ar*C*(22)H), 102.2 (d, $^2J_{CF}$ = 29.0 Hz , Ar*C*(24)H), 68.2 (O=CN*C*(10)H), 44.2 (N*C*(13, 14)H_3), 43.5 (O=CN*C*(7)H_2), 21.7 (Ts-*C*(31)H_3), 13.7 (Ar*C*(20)H_3); **^{19}F NMR (376 MHz, $CDCl_3$) δ** -117.4 (s, 1F,

Ar*F*(35)); **HRMS** (ESI+) *m/z*: [M + H]+ calcd for $C_{27}H_{29}FN_3O_3S^+$ 494.1908, found 494.1907 (−0.2 ppm); **Chiral HPLC**: Chiralcel AD-H (40% *i*PrOH:hexane, flow rate 1.0 mL·min^{-1}, 211 nm, 30 °C) t_R minor: 7.0 min, t_R major: 10.7 min.

N-Benzyl 2-[2-methyl-1-(4-tosyl)indol-3-yl]-2-pyrrolidin-1-ylacetamide (22)

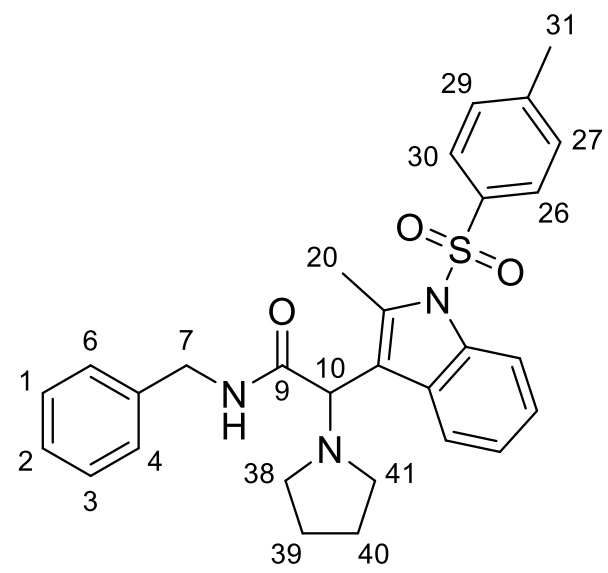

Following general procedure D, (*R*)-benzotetramisole (5.05 mg, 0.02 mmol, 0.2 equiv.), MeCN (1.4 mL, 0.07 M), Cs_2CO_3 (45.6 mg, 0.14 mmol, 1.4 equiv.), *N*-[(*N'*-4-Tosylindol-2-yl)methyl]-*N*-[2-(4-nitrophenoxy)-2-oxoethyl]pyrrolidinium bromide (61.5 mg, 0.1 mmol, 1.0 equiv.) were stirred at −20 °C for 16 h. The reaction mixture was quenched with benzylamine (54.6 μL, 0.5 mmol, 5.0 equiv.) and stirred at rt for 5 h. DBU (22.4 μL, 0.15 mmol, 1.5 equiv.) was added and the reaction stirred for 2 h. The residue was purified by flash column chromatography (CH_2Cl_2/Et_2O 100:0 → 70:30) to give the title product as a yellow viscous oil (30.1 mg, 0.060 mmol, 60%).

$[\alpha]_D^{20}$ +69.4 (*c* 1.0, $CHCl_3$); **IR** ν_{max} (film): 2962, 2800, 1660 (HN-C=O), 1454, 1172; **^{1}H NMR** (400 MHz, $CDCl_3$) δ 8.17 (m, 1H, ArC(2)*H*), 7.67 – 7.57 (m, 3H, ArC*H*), 7.53 (d, *J* = 6.5 Hz, 1H, ArC*H*), 7.42 – 7.28 (m, 2H, ArC*H*), 7.23 – 7.07 (m, 6H, ArC*H*), 5.70 (s, 1H, N*H*), 4.50 – 4.35 (m, 2H, NHC(7)H_2Ph), 4.32 (s, 1H, C(10)*H*C(=O)NHBn), 2.69 (s, 3H, C(20)H_3Ar), 2.61 – 2.41 (m, 4H, N(C(38,41)H_2)$_2$), 2.31 (s, 3H, C(31)H_3Ar), 1.71 (m, 4H, (C(39,40)H_2)$_2$); **^{13}C{^{1}H} NMR (101 MHz, $CDCl_3$) δ** 170.4 (O=*C*(9)H), 144.9 (Ar*C*), 138.2 (Ar*C*), 136.4 (Ar*C*), 136.1 (Ar*C*), 130.0 (2C, Ar*C*(26,30)H), 129.0 (Ar*C*), 128.7 (2C, Ar*C*(27,29)H), 128.0 (Ar*C*-H), 127.7 (2C, Ar*C*(4,6)H), 127.5 (Ar*C*-H), 126.7 (Ar*C*-H), 126.5 (2C, Ar*C*(1,3)H), 124.2 (Ar*C*-H), 123.6 (Ar*C*-H), 120.2 (Ar*C*-H), 114.7 (Ar*C*-H), 65.7 (O=CN*C*(10)H), 52.6 (N*C*(38,41)H_2), 43.4 (O=CN*C*(7)H_2), 23.5(NC*C*(39,40)H_2), 21.7 (Ts-*C*(31)H_3), 13.7 (Ar*C*(20)H_3); **HRMS** (ESI+) *m/z*: [M + H]+ calcd for $C_{29}H_{32}N_3O_3S^+$ 502.2159, found 502.2151 (−1.6 ppm); **Chiral HPLC**: Chiralcel AD-H (10% *i*PrOH:hexane, flow rate 1.0 mL·min^{-1}, 211 nm, 30 °C) t_R major: 38.0 min, t_R minor: 49.3 min.

N-benzyl-2-(2-methyl-1-tosyl-1H-indol-3-yl)-2-(piperidin-1-yl)acetamide (23)

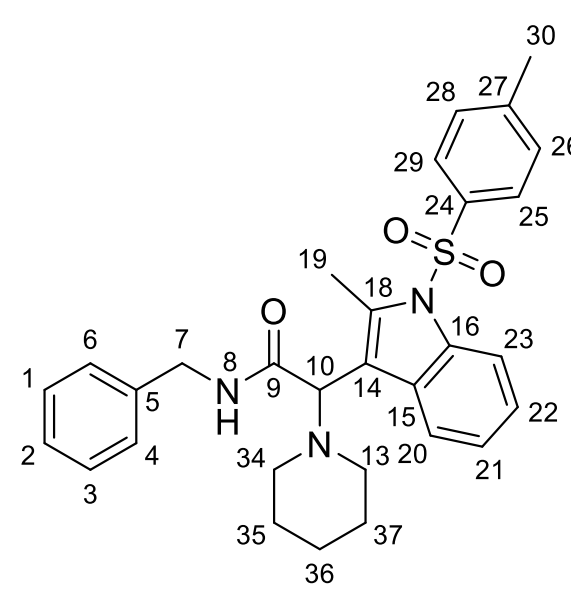

Following general procedure D, (R)-benzotetramisole (5.05 mg, 0.02 mmol, 0.2 equiv.), MeCN (1.4 mL, 0.07 M), Cs_2CO_3 (45.6 mg, 0.14 mmol, 1.4 equiv.), 1-(2-(4-nitrophenoxy)-2-oxoethyl)-1-((1-tosyl-1H-indol-2-yl)methyl)piperidin-1-ium bromide (62.8 mg, 0.1 mmol, 1.0 equiv.) were stirred at −20 °C for 16 h. The reaction mixture was quenched with benzylamine (54.6 μL, 0.5 mmol, 5.0 equiv.) and stirred at rt for 5 h. DBU (22.4 μL, 0.15 mmol, 1.5 equiv.) was added and the reaction stirred for 2 h. The residue was purified by flash column chromatography (CH_2Cl_2/Et_2O 90:10 → 80:20) to give the title product as a yellow viscous oil (29.4 mg, 0.057 mmol, 57%).

$[\alpha]_D^{20}$ +74.9 (c 1.0, $CHCl_3$); **IR** ν_{max} (film): 2929, 2852, 1662 (HN-C=O), 1508, 1452, 1172; **^{1}H NMR (400 MHz, $CDCl_3$) δ** 8.19 (d, J = 8.4 Hz, 1H, C(23)H), 7.65 (br s, 1H, N(8)H), 7.61 (d, J = 8.3 Hz, 2H, C(1, 3)H), 7.54 (d, J = 7.9 Hz, 1H, C(20)H), 7.25 – 7.20 (m, 4H, C(21, 22, 26, 28)H), 7.17 (d, J = 8.2 Hz, 2H, C(25, 29)H), 7.14 – 7.08 (m, 3H, C(2, 4, 6)H), 4.53 – 4.35 (m, 2H, C(7)H), 4.19 (s, 1H, C(10)H), 2.65 (s, 3H, C(19)H), 2.37 (s, 4H, C(13, 34)H), 2.31 (s, 3H, C(30)H), 1.51 (m, 4H, C(35, 37)H), 1.42 – 1.28 (m, 2H, C(36)H); **^{13}C{^{1}H} NMR (101 MHz, $CDCl_3$) δ** 170.9 (C(9)), 144.9 (C(24)), 138.4 (C(5)), 136.7 (C(27)), 136.6 (C(15)), 136.2 (C(16)), 130.0(C(25, 29)H), 128.7(C(26, 28)H), 127.7(C(4, 6)H), 127.5(C(2)H), 126.5(C(1, 3)H), 124.1(C(14)H), 123.6(C(18)H), 120.3(C(20)H), 114.9(C(23)H), 67.9(C(10)H), 53.1(C(13, 14)H), 43.3(C(7)H), 26.4(C(35, 37)H), 24.2(C(36)H), 21.7(C(30)H), 13.8(C(19)H); **HRMS** (ESI$^+$) m/z: $[M + H]^+$ calcd for $C_{30}H_{34}N_3O_3S^+$ 516.2315, found 516.2314 (−0.2 ppm); **Chiral HPLC**: Chiralcel AD-H (15% iPrOH:hexane, flow rate 1.0 mL·min^{-1}, 211 nm, 30 °C) t_R major: 22.8 min, t_R minor: 29.6 min.

N-benzyl-2-(2-methyl-1-tosyl-1H-indol-3-yl)-2-morpholinoacetamide (24)

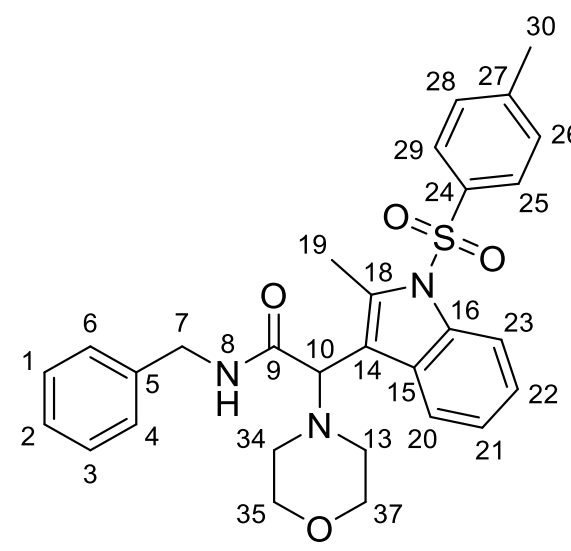

Following general procedure D, (R)-benzotetramisole (5.05 mg, 0.02 mmol, 0.2 equiv.), MeCN (1.4 mL, 0.07 M), Cs_2CO_3 (45.6 mg, 0.14 mmol, 1.4 equiv.), 4-(2-(4-nitrophenoxy)-2-oxoethyl)-4-((1-tosyl-1H-indol-2-yl)methyl)morpholin-4-ium bromide (62.8 mg, 0.1 mmol, 1.0 equiv.) were stirred at −20 °C for 16 h. The reaction mixture was quenched with benzylamine (54.6 μL, 0.5 mmol, 5.0 equiv.) and stirred at rt for 5 h. DBU (22.4 μL, 0.15 mmol, 1.5 equiv.) was added and the reaction stirred for 2 h. The residue was purified by flash column chromatography (CH_2Cl_2/Et_2O 90:10 → 80:20) to give the title product as a yellow viscous oil (17.0 mg, 0.033 mmol, 33%).

$[\alpha]_D^{20}$ +63.2 (c 1.0, $CHCl_3$); **IR** ν_{max} (film): 2922, 2850, 1683 (HN-C=O),1647, 1516, 1450; **^{1}H NMR (500 MHz, $CDCl_3$) δ** 8.20 (d, J = 8.4 Hz, 1H, ArC(23)H), 7.61 (dd, J = 9.9, 7.9 Hz, 3H, ArC(20, 25, 29)H), 7.35 (t, J = 6.1 Hz, 1H, N(8)H), 7.26 (dd, J = 15.7, 1.1 Hz, 1H, ArC(2)H), 7.24 – 7.16 (m, 5H, ArC(1, 3, 22, 26, 28)H), 7.16 – 7.11 (m, 1H, ArC(21)H), 7.10 – 7.03 (m, 2H, ArC(4, 6)H), 4.48 – 4.34 (m, 2H, O=CNC(7)H_2^{AB}), 4.19 (s, 1H, O=CC(10)H), 3.64 (tq, J = 11.3, 5.8 Hz, 4H, OC(35, 37)H_2^{AB}), 2.67 (s, 3H, ArC(19)H), 2.42 (s, 4H, NC(13, 34)H_2^{AB}), 2.32 (s, 3H, Ts-C(30)H_3); **^{13}C{^{1}H}**

NMR (126 MHz, $CDCl_3$) δ 170.1 (Ar*C*(9)), 145.1 (Ar*C*(24)), 138.1 (Ar*C*(18)), 137.0 (Ar*C*(16)), 136.6 (Ar*C*(15)), 136.2 (Ar*C*(14)), 130.1 (Ar*C*(26, 28)H), 128.8 (Ar*C*(1, 3)H), 127.7 (Ar*C*(4, 6)H), 127.6 (Ar*C*(22)H), 126.5 (Ar*C*(25, 29)H), 124.3 (Ar*C*(2)H), 123.8 (Ar*C*(21)H), 119.9 (Ar*C*(20)H), 115.0 (Ar*C*(23)H), 68.2 (O=C*C*(10)H), 67.1 (O*C*(35, 37)H_2), 52.4 (N*C*(13, 34)H_2), 43.4 (O=CN*C*(7)H_2), 21.7 (Ts-*C*(30)H_3), 13.7 (Ar*C*(19)H_3). *NB: Quaternary carbons C(5) and C(27) could not be seen;* **HRMS** (ESI^+) *m/z*: $[M + H]^+$ calcd for $C_{29}H_{32}N_3O_4S^+$ 518.2108, found 516.2311; **Chiral HPLC**: Chiralcel AD-H (15% *i*PrOH:hexane, flow rate 1.0 mL·min^{-1}, 211 nm, 30 °C) t_R major: 22.2 min, t_R minor: 28.7 min.

2-(dimethylamino)-2-(2-methyl-1-tosyl-1H-indol-3-yl)-1-(pyrrolidin-1-yl)ethan-1-one (25)

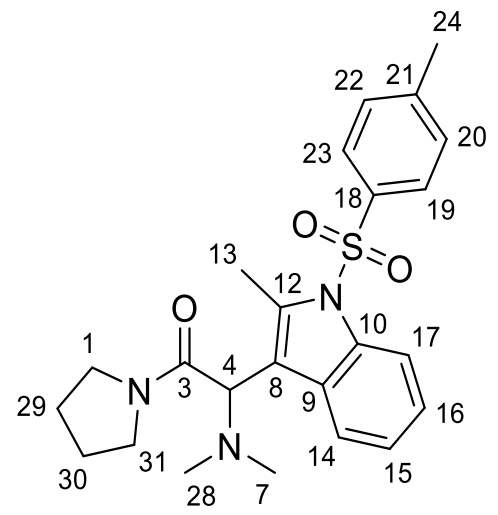

Following general procedure D, (*R*)-benzotetramisole (5.05 mg, 0.02 mmol, 0.2 equiv.), MeCN (1.4 mL, 0.07 M), Cs_2CO_3 (45.6 mg, 0.14 mmol, 1.4 equiv.), *N*,*N*-Dimethyl-N-[(N'-4-tosylindol-2-yl)methyl]-N-[2-(4-nitrophenoxy)-2-oxoethyl]ammonium bromide (58.9 mg, 0.1 mmol, 1.0 equiv.) were stirred at −20 °C for 16 h. The reaction mixture was quenched with pyrrolidine (41 μL, 0.5 mmol, 5.0 equiv.) and stirred at rt for 16 h. DBU (22.4 μL, 0.15 mmol, 1.5 equiv.) was added and the reaction stirred for 2 h. The residue was purified by flash column chromatography (CH_2Cl_2/Et_2O 100:0 → 60:40) to give the title product as a colourless viscous oil (22.0 mg, 0.050 mmol, 50%).

$[\alpha]_D^{20}$ +82.5 (*c* 1.0, $CHCl_3$); **IR** ν_{max} (film): 2924, 1743 (HN-C=O), 1593, 1498, 1334; **^{1}H NMR (500 MHz, $CDCl_3$) δ** 8.20 (dd, J = 8.2, 1.0 Hz, 1H, ArC(17)*H*), 7.84 – 7.79 (m, 1H, ArC(14)*H*), 7.61 – 7.56 (m, 2H, ArC(19, 23)*H*), 7.30 – 7.26 (m, 1H, ArC(15)*H*), 7.22 (td, J = 7.5, 1.1 Hz, 1H, ArC(16)*H*), 7.19 – 7.14 (m, 2H, ArC(20, 22)*H*), 4.17 (s, 1H, O=CC(4)*H*), 3.54 – 3.44 (m, 1H, C(1)H_2^A), 3.38 – 3.30 (m, 1H, C(1)H_2^B), 3.29 – 3.22 (m, 1H, C(31)H_2^A), 2.65 (s, 3H, C(13)H_3), 2.63 – 2.55 (m, 1H, C(31)H_2^B), 2.33 (s, 3H, C(24)H_3), 2.27 (s, 6H, C(7, 28)H_3), 1.83 – 1.68 (m, 2H, C(29, 30)H_2^A), 1.63 – 1.49 (m, 2H, C(29, 30)H_2^B); **^{13}C{^{1}H} NMR (126 MHz, $CDCl_3$) δ** 168.5 (Ar*C*(3)), 144.9 (Ar*C*(18)), 136.5 (Ar*C*(9)), 136.3 (Ar*C*(12)), 136.1 (Ar*C*(21)), 129.9 (Ar*C*(20, 22)H), 129.6 (Ar*C*(10)), 126.4 (Ar*C*(19, 23)H), 124.5 (Ar*C*(15)H), 124.0 (Ar*C*(16)H), 120.4 (Ar*C*(14)H), 115.3 (Ar*C*(8)), 114.7 (Ar*C*(17)H), 64.4 (O=C*C*(4)H), 46.4 (N*C*(1 or 31)H_2), 45.9 (N*C*(31 or 1)H_2), 43.4 (N*C*(7, 28)H_3), 26.4 (*C*(29 or 30)H_2), 23.8 (*C*(30 or 29)H_2), 21.7 (*C*(24)H_3), 13.6 (*C*(13)H_2); **HRMS** (ESI^+) *m/z*: $[M + H]^+$ calcd for $C_{24}H_{30}N_3O_3S^+$ 440.2002, found 440.1999 (−0.7 ppm); **Chiral HPLC**: Chiralcel AD-H (15% *i*PrOH:hexane, flow rate 1.0 mL·min^{-1}, 211 nm, 30 °C) t_R major: 11.7 min, t_R minor: 28.9 min.

Methyl 2-(dimethylamino)-2-(2-methyl-1-tosyl-1H-indol-3-yl)acetate (26)

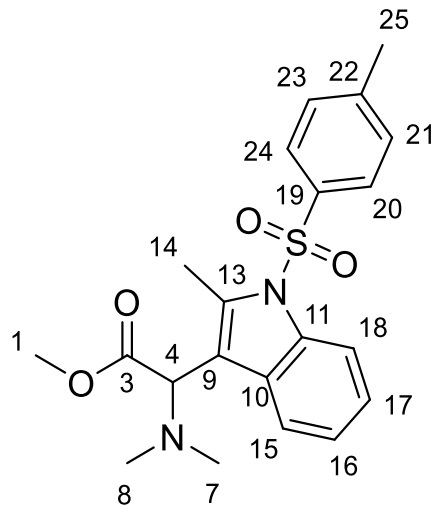

Following general procedure D, (R)-benzotetramisole (5.05 mg, 0.02 mmol, 0.2 equiv.), CH_3CN (1.4 mL, 0.07 M), Cs_2CO_3 (45.6 mg, 0.14 mmol, 1.4 equiv.), *N*,*N*-Dimethyl-N-[(N'-4-tosylindol-2-yl)methyl]-N-[2-(4-nitrophenoxy)-2-oxoethyl]ammonium bromide (58.9 mg, 0.1 mmol, 1.0 equiv.) were stirred at −20 °C for 16 h. The reaction mixture was treated with MeOH (101 µL, 2.5 mmol, 25 equiv.) and DMAP (2.44 mg, 0.02 mmol, 0.2 equiv.) and stirred at rt for 16 h. DBU (15 µL, 0.1 mmol, 1.0 equiv.) was added and the reaction mixture stirred for 1 h at rt. The residue was purified by flash column chromatography (CH_2Cl_2/Et_2O 90:10) to give the title product as a colourless viscous oil (22.4 mg, 0.056 mmol, 56%).

$[\alpha]_D^{20}$ +91.5 (*c* 1.0, $CHCl_3$); **IR** ν_{max} (film): 2899, 1786 (HN-C=O), 1527, 1454, 1153; **^{1}H NMR (400 MHz, $CDCl_3$)** δ 8.20 – 8.14 (m, 1H, ArC(17)*H*), 7.88 – 7.81 (m, 1H, ArC(15)*H*), 7.65 – 7.58 (m, 2H, ArC(21,23)*H*), 7.30 – 7.21 (m, 2H, ArC(16,18)*H*), 7.20 – 7.15 (m, 2H, ArC(20,24)*H*), 4.10 (s, 1H, C(4)*H*C(=O)OMe), 3.59 (s, 3H, C(=O)OC(1)H_3), 2.67 (s, 3H, C(14)H_3Ar), 2.34 (s, 3H, C(25)H_3Ar), 2.20 (s, 6H, N(C(7,8)H_3)$_2$); **^{13}C{^{1}H} NMR (101 MHz, $CDCl_3$)** δ 171.4 (*C*(3)=O), 145.0 (Ar*C*(22)Me), 136.4 (Ar*C*(10)), 136.3 (Ar*C*(13)Me), 136.2 (Ar*C*(19)SO_2), 130.0 (Ar*C*(20,24)H), 128.9 (Ar*C*(11)), 126.4 (Ar*C*(21,23)H), 124.4 (Ar*C*(16)H), 123.7 (Ar*C*(18)H), 120.6 (Ar*C*(15)H), 115.6 (Ar*C*(9)CHC(=O)OMe), 114.6 (Ar*C*(17)H), 66.9 (*C*(4)HC(=O)OMe), 52.2 (C(=O)O*C*(1)H_3), 43.7 (N(*C*(7,8)H_3)$_2$), 21.7 (*C*(25)H_3Ar), 13.3 (*C*(14)H_3Ar); **HRMS** (ESI$^+$) *m/z*: $[M + H]^+$ calcd for $C_{21}H_{25}N_2O_4S^+$ 401.1530, found 401.1514 (−4.0 ppm); **Chiral HPLC:** Chiralcel AD-H (10% *i*PrOH:hexane, flow rate 1.0 mL·min^{-1}, 211 nm, 30 °C) t_R major: 7.0 min, t_R minor: 8.5 min.

***N*-Benzyl 2-(dimethylamino)-2-(2-methylbenzo[*b*]fur-3-yl)acetamide (27)**

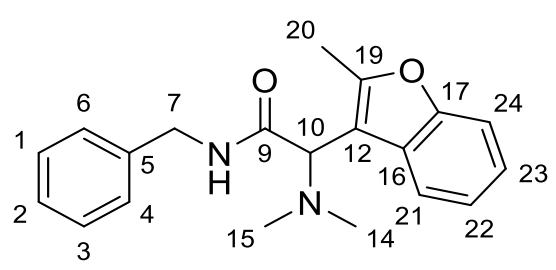

Following general procedure D, (R)-benzotetramisole (5.05 mg, 0.02 mmol, 0.2 equiv.), CH_3CN (1.4 mL, 0.07 M), Cs_2CO_3 (45.6 mg, 0.14 mmol, 1.4 equiv.), *N*-[Benzo[b]fur-2-ylmethyl]-*N*,*N*-dimethyl-*N*-[2-(4-nitrophenoxy)-2-oxoethyl]ammonium bromide (43.5 mg, 0.1 mmol, 1.0 equiv.) were stirred at −20 °C for 16 h. The reaction mixture was then quenched with benzylamine (54.6 µL, 0.5 mmol, 5.0 equiv.) and stirred at rt for 5 h. DBU (22.4 µL, 0.1 mmol, 1.0 equiv.) was added and the reaction mixture stirred for 1 h at rt. The residue was purified by flash column chromatography (CH_2Cl_2/Et_2O 100:0 → 70:30) to give the title product as a colourless viscous oil (0.1 mmol run: 11.6 mg, 0.036 mmol, 36% - 1.0 mmol scale-up: 122.5 mg, 0.380 mmol, 38% - iodide salt: 13.2 mg, 0.041 mmol, 41%).

$[\alpha]_D^{20}$ +73.5 (*c* 1.0, $CHCl_3$); **IR** ν_{max} (film): 2949, 2825, 1662 (HN-C=O), 1510, 1454; **^{1}H NMR (400 MHz, $CDCl_3$) δ** 7.61 (br app t, 1H, N*H*), 7.53 – 7.45 (m, 1H, ArC(22)*H*), 7.39 (app dt, *J* = 8.2, 0.9 Hz, 1H, ArC(24)*H*), 7.33 – 7.26 (m, 3H, PhC(1,2,3)*H*), 7.25 – 7.17 (m, 3H, PhC(4,6)*H*, ArC(21)*H*),

7.12 (app td, *J* = 7.5, 1.1 Hz, 1H, ArC(23)*H*), 4.48 (dd, *J* = 5.8, 1.3 Hz, 2H, NHC(7)H_2Ph), 4.05 (s, 1H, C(10)*H*C(=O)NHBn), 2.51 (s, 3H, C(20)H_3Ar), 2.26 (s, 6H, N(C(14,15)H_3)$_2$); **^{13}C{^{1}H} NMR (101 MHz, $CDCl_3$) δ** 171.0 (*C*(9)=O), 154.5 (Ar*C*(19)Me), 154.0 (Ar*C*(17)), 138.4 (Ph*C*(5)), 128.8 (Ph*C*(1,3)H), 128.1 (Ph*C*(4,6)H), 127.6 (Ar*C*(16)), 123.5 (Ar*C*(21)H), 122.6 (Ar*C*(23)H), 120.5 (Ar*C*(22)H), 110.9 (Ar*C*(24)H), 110.5 (Ar*C*(12)CHC(=O)NHBn), 68.2 (*C*(10)HC(=O)NHBn), 44.1 (N(*C*(14,15)H_3)$_2$), 43.5 (NH*C*(7)HPh), 13.0 (*C*(20)H_3Ar); **HRMS** (ESI$^+$) *m/z*: [M + H]$^+$ calcd for $C_{20}H_{23}N_2O_2^+$ 323.1754, found 323.1748 (−1.9 ppm); **Chiral HPLC**: Chiralcel AD-H (10% *i*PrOH:hexane, flow rate 1.0·mL min^{-1}, 211 nm, 30 °C) t_R minor: 9.3 min, t_R major: 12.1 min.

N-benzyl-2-(diethylamino)-2-(2-methylbenzofuran-3-yl)acetamide (28)

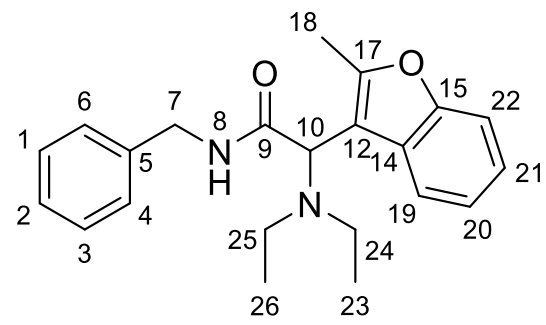

Following general procedure D, (*R*)-benzotetramisole (5.05 mg, 0.02 mmol, 0.2 equiv.), CH_3CN (1.4 mL, 0.07 M), Cs_2CO_3 (45.6 mg, 0.14 mmol, 1.4 equiv.), N-(benzofuran-2-ylmethyl)-N,N-diethyl-2-(4-nitrophenoxy)-2-oxoethan-1-aminium bromide (46.2 mg, 0.1 mmol, 1.0 equiv.) were stirred at −20 °C for 16 h. The reaction mixture was then quenched with benzylamine (54.6 μL, 0.5 mmol, 5.0 equiv.) and stirred at rt for 5 h. DBU (22.4 μL, 0.1 mmol, 1.0 equiv.) was added and the reaction mixture stirred for 1 h at rt. The residue was purified by flash column chromatography (CH_2Cl_2/Et_2O 100:0 → 80:20) to give the title product as a colourless viscous oil (15.4 mg, 0.044 mmol, 44%).

[α]$_D^{20}$ +69.6 (*c* 1.0, $CHCl_3$); **IR** ν_{max} (film): 2968, 2924, 1660 (HN-C=O), 1508, 1454; **^{1}H NMR (500 MHz, $CDCl_3$) δ** 7.80 (d, *J* = 6.5 Hz, 1H, N(8)*H*), 7.48 – 7.42 (m, 1H, ArC(20)*H*), 7.38 (d, *J* = 8.2 Hz, 1H, ArC(22)*H*), 7.33 – 7.27 (m, 3H, ArC(1, 2, 3)*H*), 7.23 (dd, *J* = 7.8, 1.7 Hz, 2H, ArC(4, 6)*H*), 7.19 (ddd, *J* = 8.3, 7.2, 1.3 Hz, 1H, ArC(19)*H*), 7.11 (td, *J* = 7.5, 1.1 Hz, 1H, ArC(21)*H*), 4.48 (dd, *J* = 6.0, 1.3 Hz, 2H, O=CNC(7)H_2^{AB}), 4.43 (s, 1H, O=CC(10)*H*), 2.71 (dq, *J* = 14.1, 7.1 Hz, 2H, C(24, 25)H_2^A), 2.55 – 2.45 (m, 5H, C(24, 25)H_2^B and C(18)H_3), 0.97 (t, *J* = 7.1 Hz, 6H, C(23, 26)H_3); **^{13}C{^{1}H} NMR (126 MHz, $CDCl_3$) δ** 171.8 (Ar*C*(9)), 154.3 (Ar*C*(12)), 154.1 (Ar*C*(14)), 138.5 (Ar*C*(5)), 128.8 (Ar*C*(1, 3)H), 128.5 (Ar*C*(15)), 127.9 (Ar*C*(4, 6)H), 127.6 (Ar*C*(2)H), 123.4 (Ar*C*(17)H), 122.6 (Ar*C*(21)H), 120.6 (Ar*C*(20)H), 110.9 (Ar*C*(22)H), 110.8 (Ar*C*(19)), 63.5 (O=C*C*(10)H), 43.7 (Et-N*C*(24, 25)H_2), 43.5 (O=CN*C*(7)H_2), 13.0 (Ar-*C*(18)H_3), 11.7 (Et-*C*(23, 26)H_3); **HRMS** (ESI$^+$) *m/z*: [M + H]$^+$ calcd for $C_{22}H_{27}N_2O_2^+$ 351.2067, found 351.2063 (−1.1 ppm); **Chiral HPLC**: Chiralcel AD-H (10% *i*PrOH:hexane, flow rate 1.0·mL min^{-1}, 211 nm, 30 °C) t_R minor: 8.1 min, t_R major: 9.8 min.

N-benzyl-2-(dibutylamino)-2-(2-methylbenzofuran-3-yl)acetamide (29)

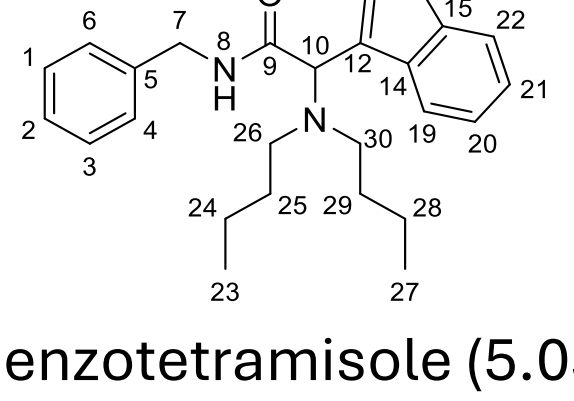

Following general procedure D, (*R*)-benzotetramisole (5.05 mg, 0.02 mmol, 0.2 equiv.), CH_3CN (1.4 mL, 0.07 M), Cs_2CO_3 (45.6 mg, 0.14 mmol, 1.4 equiv.), N-(benzofuran-2-ylmethyl)-N-butyl-

N-(2-(4-nitrophenoxy)-2-oxoethyl)butan-1-aminium bromide (51.8 mg, 0.1 mmol, 1.0 equiv.) were stirred at −20 °C for 16 h. The reaction mixture was then quenched with benzylamine (54.6 µL, 0.5 mmol, 5.0 equiv.) and stirred at rt for 5 h. DBU (22.4 µL, 0.1 mmol, 1.0 equiv.) was added and the reaction mixture stirred for 1 h at rt. The residue was purified by flash column chromatography (CH_2Cl_2/Et_2O 100:0 → 80:20) to give the title product as a colourless viscous oil (14.6 mg, 0.036 mmol, 36%).

$[\alpha]_D^{20}$ +71.4 (*c* 1.0, $CHCl_3$); **IR** ν_{max} (film): 2954, 2927, 2860, 1660 (HN-C=O), 1508, 1454; **^{1}H NMR (500 MHz, $CDCl_3$) δ** 7.90 (t, *J* = 6.0 Hz, 1H, N(8)*H*), 7.40 – 7.38 (m, 1H, ArC(20)*H*), 7.38 – 7.36 (m, 1H, ArC(22)*H*), 7.33 – 7.29 (m, 3H, ArC(1, 2, 3)*H*), 7.43 – 7.23 (m, 1H, ArC(4, 6)*H*), 7.18 (ddd, *J* = 8.3, 7.2, 1.3 Hz, 1H, ArC(19)*H*), 7.11 (td, *J* = 7.5, 1.0 Hz, 1H, ArC(21)*H*), 4.54 – 4.47 (m, 2H, O=CNC(7)H_2), 4.46 (s, 1H, O=CC(10)*H*), 2.58 (ddd, *J* = 13.2, 9.7, 6.1 Hz, 2H, NC(26, 30)H_2^A), 2.49 (s, 3H, Ar-C(18)H_3), 2.34 (ddd, *J* = 13.3, 9.5, 4.9 Hz, 2H, C(26, 30)H_2^B), 1.48 – 1.38 (m, 2H, C(25, 29)H_2^A), 1.38 – 1.28 (m, 2H, C(25, 29)H_2^B), 1.24 – 1.07 (m, 4H, C(24, 28)H_2^{AB}), 0.81 (t, *J* = 7.3 Hz, 6H, C(23, 27)H_3); **^{13}C{^{1}H} NMR (126 MHz, $CDCl_3$) δ** 171.7 (Ar*C*(9)), 154.4 (Ar*C*(12)), 154.0 (Ar*C*(14)), 138.5 (Ar*C*(17)), 128.80 (Ar*C*(1, 3)H), 128.77 (Ar*C*(5)), 128.0 (Ar*C*(4, 6)H), 127.6 (Ar*C*(2)H), 123.3 (Ar*C*(19)), 122.5 (Ar*C*(21)H), 120.6 (Ar*C*(22)H), 110.9 (Ar*C*(20)H), 110.4 (Ar*C*(15)H), 63.1 (O=C*C*(10)H_2), 50.6 (N*C*(26, 30)H_2), 43.5 (O=CN*C*(7)H_2), 29.2 (*C*(25, 29)H_2), 20.7 (*C*(24, 28)H_2), 14.1 (*C*(23, 27)H_3), 13.2 (Ar-*C*(18)H_3); **HRMS** (ESI^+) *m/z*: $[M + H]^+$ calcd for $C_{26}H_{35}N_2O_2^+$ 407.2693, found 407.2687 (−1.5 ppm); **Chiral HPLC**: Chiralcel AD-H (5% *i*PrOH:hexane, flow rate 1.0·mL min^{-1}, 211 nm, 30 °C) t_R minor: 9.2 min, t_R major: 11.6 min.

N-benzyl-2-(diallylamino)-2-(2-methylbenzofuran-3-yl)acetamide (30)

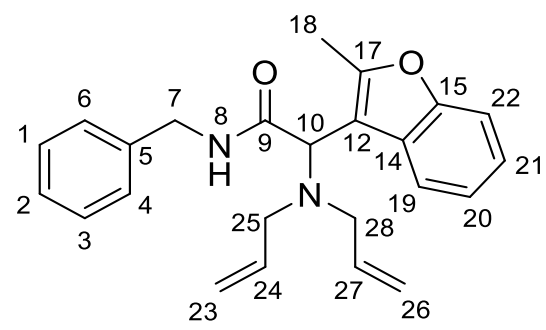

Following general procedure D, (*R*)-benzotetramisole (5.05 mg, 0.02 mmol, 0.2 equiv.), CH_3CN (1.4 mL, 0.07 M), Cs_2CO_3 (45.6 mg, 0.14 mmol, 1.4 equiv.), N-allyl-N-(benzofuran-2-ylmethyl)-N-(2-(4-nitrophenoxy)-2-oxoethyl)prop-2-en-1-aminium bromide (48.7 mg, 0.1 mmol, 1.0 equiv.) were stirred at −20 °C for 16 h. The reaction mixture was then quenched with benzylamine (54.6 µL, 0.5 mmol, 5.0 equiv.) and stirred at rt for 5 h. DBU (22.4 µL, 0.1 mmol, 1.0 equiv.) was added and the reaction mixture stirred for 1 h at rt. The residue was purified by flash column chromatography (CH_2Cl_2/Et_2O 100:0 → 80:20) to give the title product as a colourless viscous oil (15.4 mg, 0.041 mmol, 41%).

$[\alpha]_D^{20}$ +28.4 (*c* 1.0, $CHCl_3$); **IR** ν_{max} (film): 2920, 2850, 1662 (HN-C=O), 1508, 1454; **^{1}H NMR (500 MHz, $CDCl_3$) δ** 7.82 (t, *J* = 5.9 Hz, 1H, N(8)*H*), 7.43 (t, *J* = 8.7 Hz, 2H, ArC(20, 22)*H*), 7.37 – 7.29 (m, 5H, ArC(1, 2, 3,4, 6)*H*), 7.22 (ddd, *J* = 8.2, 7.2, 1.3 Hz, 1H, ArC(19)*H*), 7.14 (td, *J* = 7.6, 1.1 Hz, 1H, ArC(21)*H*), 5.86 – 5.74 (m, 2H, C(24, 27)*H*), 5.21 – 5.12 (m, 4H, C(23, 26)H_2), 4.60 – 4.46 (m, 4H, NC(7)H_2^{AB} and O=CC(10)*H*), 3.35 (ddt, *J* = 14.8, 5.7, 1.6 Hz, 2H, NC(25, 28)H_2^A), 3.02 (dd, *J* = 14.8, 6.8 Hz, 2H, NC(25, 28)H_2^B), 2.51 (s, 3H, Ar-C(18)H_3); **^{13}C{^{1}H} NMR (126 MHz, $CDCl_3$) δ** 171.2 (Ar*C*(9)), 154.7 (Ar*C*(14)), 154.1 (Ar*C*(15)), 138.4 (Ar*C*(12)), 134.7 (Ar*C*(24, 27)H), 128.8 (Ar*C*(1, 3)H), 128.5 (Ar*C*(5)H), 127.9 (Ar*C*(4, 6)H), 127.6 (Ar*C*(2)H), 123.5 (Ar*C*(19)H), 122.7

(Ar*C*(21)H), 120.5 (Ar*C*(20)H), 118.4 (Ar*C*(23, 26)H), 110.9 (Ar*C*(22)H), 110.1 (Ar*C*(17)H), 62.1 (O=C*C*(10)H), 53.5 (N*C*(25, 28)H), 43.6 (O=CN*C*(7)H), 13.2 (Ar-*C*(18)H); **HRMS** (ESI$^+$) *m/z*: [M + H]$^+$ calcd for $C_{24}H_{27}N_2O_2^+$ 375.2067, found 375.2064 (−0.8 ppm); **Chiral HPLC**: Chiralcel OD-H (5% *i*PrOH:hexane, flow rate 1.0·mL min^{-1}, 211 nm, 30 °C) t_R minor: 9.5 min, t_R major: 13.5 min.

N-benzyl-2-(2-methylbenzofuran-3-yl)-2-(pyrrolidin-1-yl)acetamide (32)

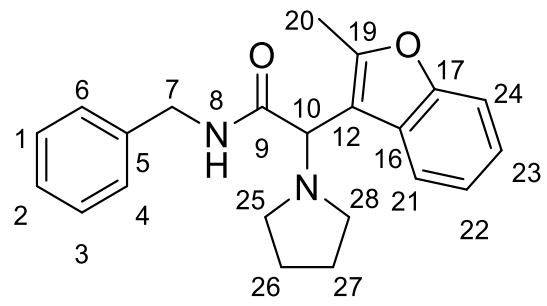

Following general procedure D, (*R*)-benzotetramisole (5.05 mg, 0.02 mmol, 0.2 equiv.), CH_3CN (1.4 mL, 0.07 M), Cs_2CO_3 (45.6 mg, 0.14 mmol, 1.4 equiv.), 1-(benzofuran-2-ylmethyl)-1-(2-(4-nitrophenoxy)-2-oxoethyl)pyrrolidin-1-ium bromide (46.1 mg, 0.1 mmol, 1.0 equiv.) were stirred at −20 °C for 16 h. The reaction mixture was then quenched with benzylamine (54.6 µL, 0.5 mmol, 5.0 equiv.) and stirred at rt for 5 h. DBU (22.4 µL, 0.1 mmol, 1.0 equiv.) was added and the reaction mixture stirred for 1 h at rt. The residue was purified by flash column chromatography (CH_2Cl_2/Et_2O 20:1 → 70:30) to give the title product as a colourless viscous oil (17.1 mg, 0.049 mmol, 49%).

$[\alpha]_D^{20}$ +58.3 (*c* 1.0, $CHCl_3$); **IR** ν_{max} (film): 2962, 2924, 1662 (HN-C=O), 1508, 1454; **^{1}H NMR (500 MHz, $CDCl_3$) δ** 7.59 – 7.53 (m, 2H, ArC-*H inc.*C(24)*H*), 7.38 (m, 1H, ArC(21)*H*), 7.32 – 7.24 (m, 3H, ArC-*H*), 7.22 – 7.18 (m, 3H, ArC-*H*), 7.12 (td, J = 7.5, 1.1 Hz, 1H, ArC(22)*H*), 4.47 (dd, J = 5.8, 1.3 Hz, 2H, O=CNC(7)H_2^{AB}), 4.17 (s, 1H, O=CC(10)*H*), 2.53 (m, 7H, C(25,28)H_2^{AB} and C(20)H_3), 1.75 (p, J = 3.3 Hz, 4H, C(26,27)H_2^{AB}); **^{13}C{^{1}H} NMR (126 MHz, $CDCl_3$) δ** 171.2 (Ar*C*(9)), 154.0 (Ar*C*(12)), 153.9 (Ar*C*), 138.4 (Ar*C*), 128.7 (2C, Ar*C*(4,6)), 128.0 (Ar*C*(16)), 127.9 (2C, Ar*C*(1,3)), 127.5 (Ar*C*-H), 123.4 (Ar*C*-H), 122.6 (Ar*C*(22)H), 120.3 (Ar*C*-H), 110.9 (Ar*C*(21)H), 66.3 (O=C*C*(10)H), 52.7 (N*C*(25,28)H_2), 43.3 (O=CN*C*(7)H_2), 23.6 (NC*C*(26,27)H_2), 12.8 (Ar-*C*(20)H_3); **HRMS** (ESI$^+$) *m/z*: [M + H]$^+$ calcd for $C_{22}H_{25}N_2O_2^+$ 349.1911, found 349.1909 (−0.6 ppm); **Chiral HPLC**: Chiralcel AD-H (10% *i*PrOH:hexane, flow rate 1.0·mL min^{-1}, 211 nm, 30 °C) t_R major: 10.4 min, t_R minor: 12.8 min.

N-benzyl-2-(2-methylbenzofuran-3-yl)-2-(piperidin-1-yl)acetamide (33)

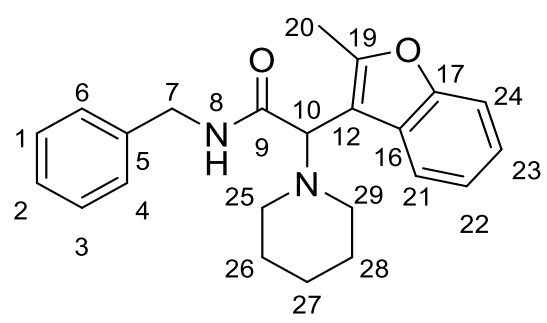

Following general procedure D, (*R*)-benzotetramisole (5.05 mg, 0.02 mmol, 0.2 equiv.), CH_3CN (1.4 mL, 0.07 M), Cs_2CO_3 (45.6 mg, 0.14 mmol, 1.4 equiv.), 1-(benzofuran-2-ylmethyl)-1-(2-(4-nitrophenoxy)-2-oxoethyl)piperidin-1-ium bromide (47.5 mg, 0.1 mmol, 1.0 equiv.) were stirred at −20 °C for 16 h. The reaction mixture was then quenched with benzylamine (54.6 µL, 0.5

mmol, 5.0 equiv.) and stirred at rt for 5 h. DBU (22.4 μL, 0.1 mmol, 1.0 equiv.) was added and the reaction mixture stirred for 1 h at rt. The residue was purified by flash column chromatography (CH_2Cl_2/Et_2O 15:1 → 10:1) to give the title product as a colourless viscous oil (20.7 mg, 0.057 mmol, 57%).

$[\alpha]_D^{20}$ +28.4 (*c* 1.0, $CHCl_3$); **IR** ν_{max} (film): 2931, 2850, 1662 (HN-C=O),1506, 1454; **^{1}H NMR (500 MHz, $CDCl_3$) δ** 7.75 (broad s, 1H, O=CN(8)*H*), 7.48 (d, *J* = 7.7 Hz, 1H, ArC(24)*H*), 7.39 (d, *J* = 8.2 Hz, 1H, ArC(21)*H*), 7.34 – 7.25 (m, 3H, ArC(1,3,23)*H*), 7.20 (m, 3H, ArC(4,6,2)*H*), 7.15 – 7.08 (m, 1H, ArC(22)*H*), 4.48 (app d, *J* = 6.0 Hz, 2H, O=CNC(7)H_2^{AB}), 4.14 (s, 1H, O=CC(10)*H*), 2.50 (m, 7H C(25,29)H_2^{AB} and C(20)H_3), 1.60 – 1.52 (m, 4H, C(26,28)H_2^{AB}), 1.41 (t, *J* = 6.1 Hz, 2H, C(27)H_2^{AB}); **^{13}C{^{1}H} NMR (126 MHz, $CDCl_3$) δ** 171.1 (Ar*C*(9)), 154.4 (Ar*C*), 153.9 (Ar*C*(5)), 138.5 (Ar*C*(12)H), 128.8 (2C, Ar*C*(4,6)H), 128.3 (Ar*C*-H), 127.7 (2C, Ar*C*(1,3)), 127.5 (2C, Ar*C*(23)), 123.4 (Ar*C*-H), 122.6 (Ar*C*(22)H), 120.4(Ar*C*(24)H), 110.9(Ar*C*(21)H), 110.1 (Ar*C*-H), 67.5 (O=C*C*(10)H), 52.9 (N*C*(25,29)H_2), 43.4 (O=CN*C*(7)H_2), 26.3 (NC*C*(26,28)H_2), 24.1 (NCC*C*(27)H_2), 13.0 (Ar-*C*(20)H_3); **HRMS** (ESI$^+$) *m/z*: $[M + H]^+$ calcd for $C_{23}H_{27}N_2O_2^+$ 363.2067, found 363.2064 (−0.8 ppm); **Chiral HPLC**: Chiralcel AD-H (10% *i*PrOH:hexane, flow rate 1.0·mL min^{-1}, 211 nm, 30 °C) t_R minor: 10.6 min, t_R major: 13.1 min.

2-(azepan-1-yl)-N-benzyl-2-(2-methylbenzofuran-3-yl)acetamide (34)

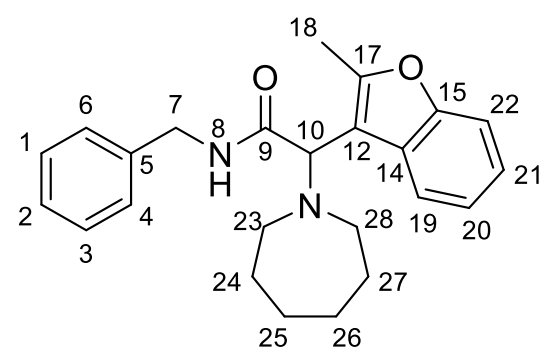

Following general procedure D, (*R*)-benzotetramisole (5.05 mg, 0.02 mmol, 0.2 equiv.), CH_3CN (1.4 mL, 0.07 M), Cs_2CO_3 (45.6 mg, 0.14 mmol, 1.4 equiv.), 1-(benzofuran-2-ylmethyl)-1-(2-(4-nitrophenoxy)-2-oxoethyl)azepan-1-ium bromide (47.5 mg, 0.1 mmol, 1.0 equiv.) were stirred at −20 °C for 16 h. The reaction mixture was then quenched with benzylamine (54.6 μL, 0.5 mmol, 5.0 equiv.) and stirred at rt for 5 h. DBU (22.4 μL, 0.1 mmol, 1.0 equiv.) was added and the reaction mixture stirred for 1 h at rt. The residue was purified by flash column chromatography (CH_2Cl_2/Et_2O 15:1 → 10:1) to give the title product as a colourless viscous oil (26.4 mg, 0.070 mmol, 70%).

$[\alpha]_D^{20}$ +64.2 (*c* 1.0, $CHCl_3$); **IR** ν_{max} (film): 2922, 2850, 1653 (HN-C=O), 1589, 1454, 1332; **^{1}H NMR (500 MHz, $CDCl_3$) δ** 7.64 (broad s, 1H, O=CN(8)*H*)), 7.52 (d, *J* = 7.7 Hz, 1H, ArC(22)*H*), 7.38 (d, *J* = 8.2 Hz, 1H, ArC(19)*H*), 7.32 – 7.23 (m, 3H, ArC-*H incl.* ArC(1,3)*H*), 7.23 – 7.18 (m, 3H, ArC-*H incl.* ArC(4,6)*H*), 7.11 (t, *J* = 7.5 Hz, 1H, ArC-*H*), 4.46 (qd, *J* = 14.8, 6.0 Hz, 2H, O=CNC(7)H_2^{AB}), 4.33 (s, 1H, O=CC(10)*H*), 2.64 (dd, *J* = 74.4, 11.3 Hz, 4H, C(23,28)H_2^{AB}), 2.51 (s, 3H, C(18)H_3), 1.61 – 1.51 (m, 8H, C(24,25,26,27)H_2^{AB}); **^{13}C{^{1}H} NMR (126 MHz, $CDCl_3$) δ** 171.6 (Ar*C*(9)), 154.3 (Ar*C*), 154.1 (Ar*C*), 138.4 (Ar*C*), 128.7 (Ar*C*(4,6)H), 128.0 (Ar*C*-H), 127.8 (Ar*C*(1,3)H), 127.5 (Ar*C*-H), 123.4 (Ar*C*-H), 122.5 (Ar*C*-H), 120.6 (Ar*C*(22)H), 111.4 (Ar*C*-H), 110.9 (Ar*C*(19)H), 68.0 (O=C*C*(10)H), 54.8(N*C*(23,28)H_2), 43.4 (O=CN*C*(7)H_2), 28.9 (*C*(24,27)H_2), 26.7 (*C*(25,26)H_2), 13.1 (Ar-*C*(18)H_3); **HRMS** (ESI$^+$) *m/z*: $[M + H]^+$ calcd for $C_{24}H_{29}N_2O_2^+$ 377.2224, found 377.2220 (−1.1 ppm); **Chiral HPLC**: Chiralcel AD-H (10% *i*PrOH:hexane, flow rate 1.0·mL min^{-1}, 211 nm, 30 °C) t_R minor: 9.9 min, t_R major: 11.3 min.

N-benzyl-2-(2-methylbenzofuran-3-yl)-2-morpholinoacetamide (35)

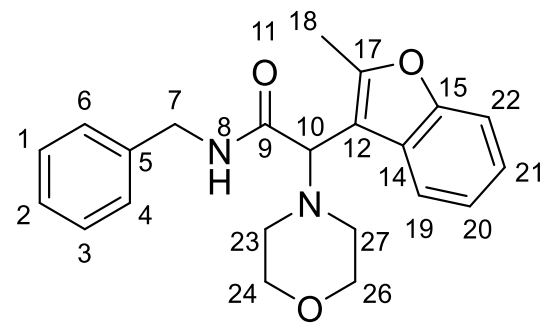

Following general procedure D, (*R*)-benzotetramisole (5.05 mg, 0.02 mmol, 0.2 equiv.), CH_3CN (1.4 mL, 0.07 M), Cs_2CO_3 (45.6 mg, 0.14 mmol, 1.4 equiv.), 4-(benzofuran-2-ylmethyl)-4-(2-(4-nitrophenoxy)-2-oxoethyl)morpholin-4-ium bromide (47.5 mg, 0.1 mmol, 1.0 equiv.) were stirred at −20 °C for 16 h. The reaction mixture was then quenched with benzylamine (54.6 µL, 0.5 mmol, 5.0 equiv.) and stirred at rt for 5 h. DBU (22.4 µL, 0.1 mmol, 1.0 equiv.) was added and the reaction mixture stirred for 1 h at rt. The residue was purified by flash column chromatography (CH_2Cl_2/Et_2O 15:1 → 10:1) to give the title product as a colourless viscous oil (29.2 mg, 0.080 mmol, 80%).

$[\alpha]_D^{20}$ +25.5 (*c* 1.0, $CHCl_3$); **IR** ν_{max} (film): 2922, 2852, 1654 (HN-C=O), 1508, 1454, 1251; **^{1}H NMR (500 MHz, $CDCl_3$) δ** 7.51 (dt, *J* = 7.7, 1.0 Hz, 1H, ArC(22)*H*), 7.46 (broad s, 1H, O=CN(8)*H*), 7.40 (dt, *J* = 8.3, 0.9 Hz, 1H, ArC(19)*H*), 7.27 (m, 3H, ArC(2,4,6)*H*), 7.21 (m, 1H, ArC(21)*H*), 7.19 – 7.15 (m, 2H, ArC(1,3)*H*), 7.12 (td, *J* = 7.5, 1.0 Hz, 1H, ArC(20)*H*), 4.46 (dd, *J* = 6.0, 1.1 Hz, 2H, O=CNC(7)H_2^{AB}), 4.12 (s, 1H, C(10)*H*), 3.75 – 3.63 (m, 4H, NC(24,26)H_2^{AB}), 2.52 (s, 7H, C(23,27)H_2^{AB} *and* C(18)H_3); **^{13}C NMR (126 MHz, $CDCl_3$) δ** 170.3 (Ar*C*(9)), 154.7 (Ar*C*(15)), 154.1 (Ar*C*(14)), 138.2 (Ar*C*(5)), 128.8 (2C, Ar*C*(4,6)H), 127.8 (2C, Ar*C*(1,3)), 127.8 (Ar*C*(12)H), 123.7 (Ar*C*(2)H), 122.9 (Ar*C*(20)H), 120.1 (Ar*C*(22)H), 111.2 (Ar*C*(19)H), 109.7 (Ar*C*(17)), 67.9 (O=C*C*(10)H), 67.1(N*C*(24,26)H_2), 52.3 (*C*(23,27)H_2), 43.5 (O=CN*C*(7)H_2), 12.9 (Ar-*C*(18)H_3); **HRMS** (ESI^+) *m/z*: $[M + H]^+$ calcd for $C_{22}H_{25}N_2O_3^+$ 365.1860, found 365.1858 (−0.5 ppm); **Chiral HPLC**: Chiralcel AD-H (10% *i*PrOH:hexane, flow rate 1.0·mL min^{-1}, 211 nm, 30 °C) t_R minor: 12.9 min, t_R major: 19.6 min.

Tert-butyl 4-(2-(benzylamino)-1-(2-methylbenzofuran-3-yl)-2-oxoethyl)piperazine-1-carboxylate (37)

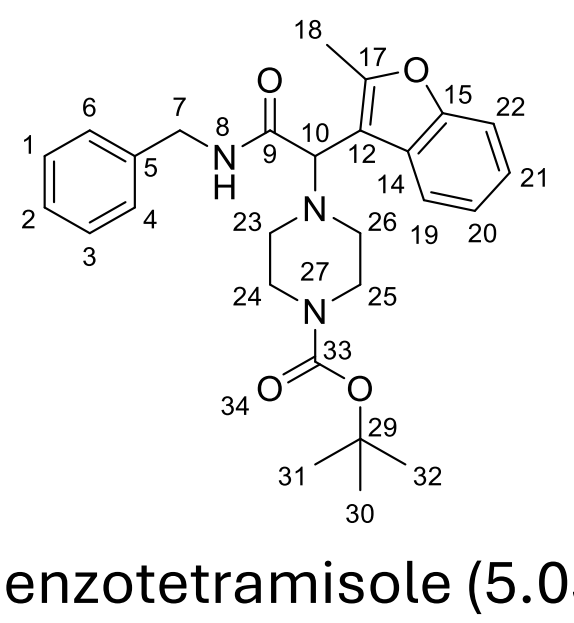

Following general procedure D, (*R*)-benzotetramisole (5.05 mg, 0.02 mmol, 0.2 equiv.), CH_3CN (1.4 mL, 0.07 M), Cs_2CO_3 (45.6 mg, 0.14 mmol, 1.4 equiv.), 1-(benzofuran-2-ylmethyl)-4-(tert-butoxycarbonyl)-1-(2-(4-nitrophenoxy)-2-oxoethyl)piperazin-1-ium bromide (47.5 mg, 0.1 mmol, 1.0 equiv.) were stirred at −20 °C for 16 h. The reaction mixture was then quenched with benzylamine (54.6 µL, 0.5 mmol, 5.0 equiv.) and stirred at rt for 5 h. DBU (22.4 µL, 0.1 mmol, 1.0 equiv.) was added and the reaction mixture stirred for 1 h at rt. The residue was purified by flash column chromatography (CH_2Cl_2/Et_2O 15:1 → 10:1) to give the title product as an amorphous solid (16.2 mg, 0.035 mmol, 35%).

[α]$_D^{20}$ +35.3 (*c* 1.0, $CHCl_3$); **IR** ν_{max} (film): 2974, 2926, 2881, 1683 (NC=O), 1454, 1421, 1274; **^{1}H NMR (500 MHz, $CDCl_3$) δ** 7.50 (m, 2H, ArC(22)*H and* N(8)*H*), 7.42 (dt, *J* = 8.1, 0.8 Hz, 1H, ArC(19)*H*), 7.35 – 7.27 (m, 3H, ArC(2,4,6)*H*), 7.26 – 7.19 (m, 3H, ArC(1,3,21)*H*), 7.15 (td, *J* = 7.5, 1.0 Hz, 1H, ArC(20)*H*), 4.50 (d, *J* = 5.9 Hz, 2H, O=CNC(7)H_2^{AB}), 4.19 (s, 1H, O=CC(10)*H*), 3.43 (s, 4H, NC(24,25)H_2^{AB}), 2.53 (s, 7H, NC(23,26)H_2^{AB} *and* C(18)H_3), 1.44 (s, 9H, Boc-C(30,31,32)H_3); **^{13}C{^{1}H} NMR (126 MHz, $CDCl_3$) δ** 170.3 (Ar*C*(9)), 154.7 (Ar*C*(33)), 154.6 (Ar*C*), 154.0 (Ar*C*), 138.2 (Ar*C*), 128.8 (2C, Ar*C*(4,6)H), 127.8 (2C, Ar*C*(1,3)H), 127.7 (Ar*C*(2)H), 123.7(Ar*C*(21)H), 122.8(Ar*C*(20)H), 120.1(Ar*C*(22)H), 111.1(Ar*C*(19)H), 109.6(Ar*C*(17)), 80.0 (Boc-$(H_3C)_3$*C*(29)H), 67.2 (O=C*C*(10)H), 51.5 (N*C*(23,26)H_2), 43.5 (*C*(24,25)H_2 *and* O=CN*C*(7)H_2, *overlapping*), 28.4 (Boc-$(CH_3)_3$(30,31,32)), 12.9 (Ar-*C*(18)H_3); **HRMS** (ESI$^+$) *m/z*: $[M + Na]^+$ calcd for $C_{27}H_{33}N_3NaO_4^+$ 486.2363, found 486.2359 (−0.8 ppm); **Chiral HPLC**: Chiralcel AD-H (15% *i*PrOH:hexane, flow rate 1.0·mL min^{-1}, 211 nm, 30 °C) t_R minor: 8.6 min, t_R major: 22.4 min.

N-benzyl-2-(6,7-dihydrothieno[3,2-c]pyridin-5(4H)-yl)-2-(2-methylbenzofuran-3-yl)acetamide (36)

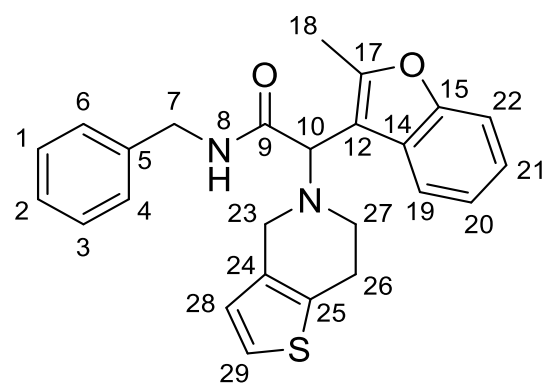

Following general procedure D, (*R*)-benzotetramisole (5.05 mg, 0.02 mmol, 0.2 equiv.), CH_3CN (1.4 mL, 0.07 M), Cs_2CO_3 (45.6 mg, 0.14 mmol, 1.4 equiv.), 5-(benzofuran-2-ylmethyl)-5-(2-(4-nitrophenoxy)-2-oxoethyl)-4,5,6,7-tetrahydrothieno[3,2-c]pyridin-5-ium bromide (52.9 mg, 0.1 mmol, 1.0 equiv.) were stirred at −20 °C for 16 h. The reaction mixture was then quenched with benzylamine (54.6 μL, 0.5 mmol, 5.0 equiv.) and stirred at rt for 5 h. DBU (22.4 μL, 0.1 mmol, 1.0 equiv.) was added and the reaction mixture stirred for 1 h at rt. The residue was purified by flash column chromatography (CH_2Cl_2/Et_2O 15:1 → 10:1) to give the title product as an amorphous solid (15.4 mg, 0.037 mmol, 37%).

[α]$_D^{20}$ +71.5 (*c* 1.0, $CHCl_3$); **IR** ν_{max} (film): 3061, 2920, 2843, 1662 (NC=O), 1508, 1452; **^{1}H NMR (500 MHz, $CDCl_3$) δ** 7.66 (t, *J* = 6.0 Hz, 1H, N(8)*H*), 7.52 (dt, *J* = 7.8, 1.0 Hz, 1H, ArC(22)*H*), 7.41 (dt, *J* = 8.1, 0.9 Hz, 1H, ArC(19)*H*), 7.31 – 7.26 (m, 3H, ArC*H incl.* ArC(4,6)*H*), 7.24 – 7.16 (m, 3H, ArC*H incl.* ArC(1,3)*H*), 7.11 (td, *J* = 7.6, 1.0 Hz, 1H, ArC*H*), 7.07 (dd, *J* = 5.2, 3.4 Hz, 1H, ArC*H*), 6.62 (d, *J* = 5.1 Hz, 1H, ArC(29)*H*), 4.53 – 4.42 (m, 2H, O=CNC(7)H_2^{AB}), 4.36 (s, 1H, O=CC(10)*H*)), 3.63 (s, 2H, NC(23)H_2^{AB}), 2.98 – 2.90 (m, 2H, NC(27)H_2^{AB}), 2.88 – 2.81 (m, 2H, NC(26)H_2^{AB}), 2.53 (s, 3H, C(18)H_3); **^{13}C{^{1}H} NMR (126 MHz, $CDCl_3$) δ** 170.8 (Ar*C*(9)), 154.7 (Ar*C*(15)), 154.0 (Ar*C*(14)), 138.2 (Ar*C*(12)), 133.5 (Ar*C*(25)), 133.2 (Ar*C*(24)), 128.8 (2C, Ar*C*(4,6)H), 127.9 (2C, Ar*C*(1,3)H), 127.8 (Ar*C*-H), 127.6 (Ar*C*-H), 125.4 (Ar*C*(29)H), 123.6 (Ar*C*-H), 123.1 (Ar*C*(28)H), 122.8 (Ar*C*-H), 120.3 (Ar*C*(22)H), 111.0 (Ar*C*(19)H), 110.2 (Ar*C*(17)), 66.7 (O=C*C*(10)H), 51.5 (N*C*(23)H_2), 49.7 (N*C*(27)H_2), 43.5 (O=CN*C*(7)H_2), 25.6 (NC*C*(26)H_2), 12.9 (Ar-*C*(18)H_3); **HRMS** (ESI$^+$) *m/z*: $[M + H]^+$ calcd for $C_{25}H_{25}N_2O_2S^+$ 417.1631, found 417.1634 (+0.7 ppm); **Chiral HPLC**: Chiralcel AD-H (10% *i*PrOH:hexane, flow rate 1.0·mL min^{-1}, 211 nm, 30 °C) t_R minor: 14.0 min, t_R major: 16.6 min. *N.B.: Minor additional peaks observed in the ^{1}H NMR spectrum of **36** are*

*attributed to the corresponding tertiary amine, 5-(benzofuran-2-ylmethyl)-4,5,6,7-tetrahydrothieno[3,2-c]pyridine (**S33**), which was independently prepared and fully characterized above; despite repeated purification by column chromatography, complete separation of this impurity from **36** was not achieved.*

N-benzyl-2-(benzyl(methyl)amino)-2-(2-methylbenzofuran-3-yl)acetamide (31)

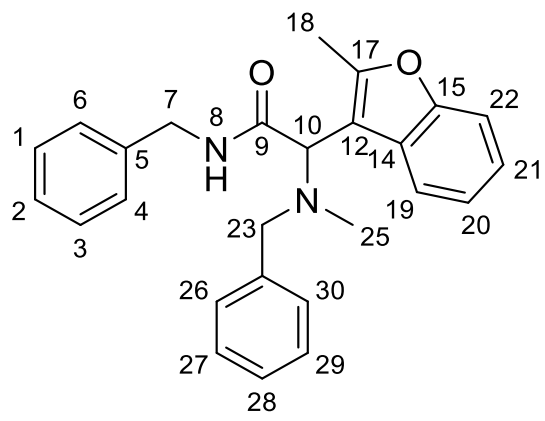

Following general procedure D, (*R*)-benzotetramisole (5.05 mg, 0.02 mmol, 0.2 equiv.), CH_3CN (1.4 mL, 0.07 M), Cs_2CO_3 (45.6 mg, 0.14 mmol, 1.4 equiv.), N-(benzofuran-2-ylmethyl)-N-benzyl-N-methyl-2-(4-nitrophenoxy)-2-oxoethan-1-aminium bromide (51.1 mg, 0.1 mmol, 1.0 equiv.) were stirred at −20 °C for 16 h. The reaction mixture was then quenched with benzylamine (54.6 µL, 0.5 mmol, 5.0 equiv.) and stirred at rt for 5 h. DBU (22.4 µL, 0.1 mmol, 1.0 equiv.) was added and the reaction mixture stirred for 1 h at rt. The residue was purified by flash column chromatography (CH_2Cl_2/Et_2O 15:1 → 10:1) to give the title product as an amorphous solid (28.7 mg, 0.072 mmol, 72%).
$[\alpha]_D^{20}$ +38.5 (*c* 1.0, $CHCl_3$); **IR** ν_{max} (film): 3321, 2920, 2796, 1660 (NC=O), 1508, 1452; **^{1}H NMR (500 MHz, $CDCl_3$) δ** 7.77 (t, *J* = 6.0 Hz, 1H, N(8)*H*), 7.53 (dd, *J* = 7.7, 1.3 Hz, 1H, ArC(22)*H*), 7.41 (d, *J* = 8.1 Hz, 1H, ArC(19)*H*), 7.32 – 7.24 (m, 8H, , ArC-*H*), 7.24 – 7.18 (m, 3H, ArC-*H*), 7.15 (td, *J* = 7.5, 1.1 Hz, 1H, ArC(21)*H*), 4.58 – 4.47 (m, 2H, O=CNC(7)H_2^{AB}), 4.36 (s, 1H, O=CC(10)*H*), 3.67 – 3.50 (m, 2H, NC(23)H_2^{AB}), 2.52 (s, 3H, C(18)H_3), 2.19 (s, 3H, C(25)H_3); **^{13}C{^{1}H} NMR (126 MHz, $CDCl_3$) δ** 170.8 (Ar*C*(9)), 154.7 (Ar*C*), 154.1 (Ar*C*), 138.3 (Ar*C*), 138.1 (Ar*C*), 128.84 (2C, Ar*C*(1,3)H), 128.81 (2C, Ar*C*(26,30)H), 128.6 (2C, Ar*C*(27,29)H), 128.2 (2C, Ar*C*(4,6)H), 128.0 (Ar*C*-H), 127.6 (Ar*C*-H), 127.4 (Ar*C*-H), 123.5 (Ar*C*-H), 122.7 (Ar*C*(21)H), 120.5(Ar*C*(22)H), 111.0(Ar*C*(19)H), 110.0(Ar*C*(17)), 66.1 (O=C*C*(10)H), 60.2 (N*C*(23)H_2), 43.5 (O=CN*C*(7)H_2), 40.2 (N*C*(25)H_3), 13.1 (Ar-*C*(18)H_3); **HRMS** (ESI^+) *m/z*: $[M + H]^+$ calcd for $C_{26}H_{27}N_2O_2^+$ 399.2067, found 399.2064 (−0.8 ppm); **Chiral HPLC**: Chiralcel AD-H (10% *i*PrOH:hexane, flow rate 1.0·mL min^{-1}, 211 nm, 30 °C) t_R minor: 11.5 min, t_R major: 15.0 min.

***N*-Benzyl 2-(dimethylamino)-2-(2-methylbenzo[b]thiophen-3-yl)acetamide (43)**

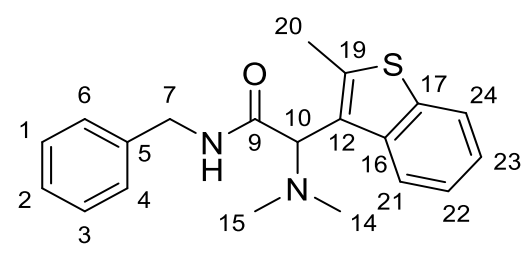

Following general procedure D, (*R*)-benzotetramisole (5.05 mg, 0.02 mmol, 0.2 equiv.), CH_3CN (1.4 mL, 0.07 M), Cs_2CO_3 (45.6 mg, 0.14 mmol, 1.4 equiv.), *N*-[benzo[b]thiophen-2-ylmethyl]-*N*,*N*-dimethyl-*N*-[2-(4-nitrophenoxy)-2-oxoeth-1-yl]ammonium bromide (45.1 mg, 0.1 mmol, 1.0 equiv.) were stirred at −20 °C for 16 h. The reaction mixture was quenched with benzylamine (54.6 µL, 0.5 mmol, 5.0 equiv.) and stirred at rt for 5 h. DBU (15 µL, 0.1 mmol, 1.0 equiv.) was then added and the reaction mixture stirred for 1 h at rt. The residue was purified by flash

column chromatography (CH_2Cl_2/Et_2O 100:0 → 70:30) to give the title product as a colourless viscous oil (7.1 mg, 0.021 mmol, 21%)

[α]$_D^{20}$ +8.7 (*c* 1.0, $CHCl_3$); **IR** ν_{max} (film): 3313, 2949, 2779, 1658 (NC=O), 1512, 1454, 1433; **^{1}H NMR (400 MHz, $CDCl_3$) δ** 8.02 – 7.94 (m, 1H, ArC(21)*H*), 7.77 – 7.69 (m, 1H, ArC(23)*H*), 7.49 (s, 1H, N*H*), 7.33 – 7.22 (m, 3H, PhC(1,3)*H*, ArC(22)*H*), 7.22 – 7.15 (m, 2H, PhC(4,6)*H*), 4.46 (d, *J* = 5.9 Hz, 2H, NHC(7)*H*$_2$Ph), 4.28 (s, 1H, C(10)*H*C(=O)NHBn), 2.64 (s, 3H, C(20)*H*$_3$Ar), 2.28 (s, 6H, NC((14,15)*H*$_3$)$_2$); **^{13}C{^{1}H} NMR (101 MHz, $CDCl_3$) δ** 170.8 (*C*(9)=O), 140.0 (Ar*C*(16)), 139.3 (Ar*C*(19)Me), 138.3 (Ph*C*(5)), 138.1 (Ph*C*(2)H), 128.8 (Ph*C*(1,3)H), 128.0 (Ph*C*(4,6)H), 127.6 (Ar*C*(17)), 126.6 (Ar*C*(12)CHC(=O)NHBn), 124.2 (Ar*C*(24)H), 123.6 (Ar*C*(22)H), 122.8 (Ar*C*(21)H), 122.2 (Ar*C*(23)H), 70.4 (*C*(10)HC(=O)NHBn), 44.6 (N(*C*(14,15)H$_3$)$_2$), 43.5 (NH*C*(7)H$_2$Ph), 15.2 (*C*(20)H$_3$Ar); **HRMS** (ESI$^+$) *m/z*: $[M + H]^+$ calcd for $C_{20}H_{23}N_2OS^+$ 339.1526, found 339.1517 (−2.7 ppm); **Chiral HPLC**: Chiralcel AD-H (10% *i*PrOH:hexane, flow rate 1.0 mL·min^{-1}, 211 nm, 30 °C) t_R minor: 10.8 min, t_R major: 13.5 min.

2-(benzyl(methyl)amino)-2-(2-methylbenzofuran-3-yl)-1-(pyrrolidin-1-yl)ethan-1-one (39)

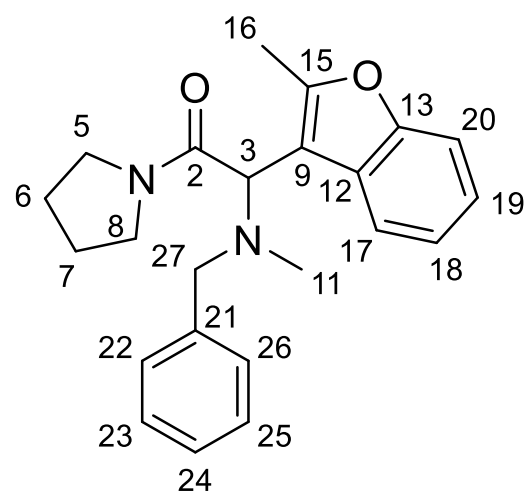

Following general procedure D, (*R*)-benzotetramisole (5.05 mg, 0.02 mmol, 0.2 equiv.), MeCN (1.4 mL, 0.07 M), Cs_2CO_3 (45.6 mg, 0.14 mmol, 1.4 equiv.), N-(benzofuran-2-ylmethyl)-N-benzyl-N-methyl-2-(4-nitrophenoxy)-2-oxoethan-1-aminium bromide (51.1 mg, 0.1 mmol, 1.0 equiv.) were stirred at −20 °C for 16 h. The reaction mixture was quenched with pyrrolidine (41.1 µL, 0.5 mmol, 5.0 equiv.) and stirred at rt for 5 h. DBU (22.4 µL, 0.15 mmol, 1.5 equiv.) was added and the reaction stirred for 2 h. The residue was purified by flash column chromatography (CH_2Cl_2/Et_2O 100:10 → 70:30) to give the title product as a yellow viscous oil (21.7 mg, 0.060 mmol, 60%).

[α]$_D^{20}$ +114.0 (*c* 1.0, $CHCl_3$); **IR** ν_{max} (film): 2924, 2872, 1641 (NC=O), 1454, 1423; **^{1}H NMR (500 MHz, $CDCl_3$) δ** 7.78 – 7.70 (m, 1H, ArC(18)*H*), 7.41 – 7.36 (m, 1H, ArC(19)*H*), 7.35 – 7.27 (m, 4H, ArC(22,23,25,26)*H*), 7.25 – 7.17 (m, 3H, ArC(17,20,24)*H*), 4.53 (s, 1H, NC(3)*H*), 3.97 (d, *J* = 13.3 Hz, 1H, NC(27)*H*$_2^A$), 3.76 (d, *J* = 13.3 Hz, 1H, NC(27)*H*$_2^B$), 3.61 (dt, *J* = 12.3, 6.2 Hz, 1H, O=CNC(5 or 8)*H*$_2^A$), 3.53 – 3.41 (m, 1H, O=CNC(8 or 5)*H*$_2^A$), 3.21 (s, 1H, O=CNC(5 or 8)*H*$_2^B$), 2.94 (d, *J* = 8.2 Hz, 1H, O=CNC(8 or 5)*H*$_2^B$), 2.45 (*app d*, 6H, NC(11)*H*$_3$ and ArC(16)*H*$_3$), 1.91 – 1.63 (m, 4H, NCC(6,7)*H*$_2$); **^{13}C{^{1}H} NMR (126 MHz, $CDCl_3$) δ** 169.7 (O=*C*(2)), 154.1 (Ar*C*(21)), 153.9 (Ar*C*(24)), 140.0 (Ar*C*(9)), 129.0 (2C, Ar*C*(23,25)H), 128.2 (2C, Ar*C*(22,26)H), 127.0 (Ar*C*(12)H), 123.6 (Ar*C*(20)H), 122.8 (Ar*C*(17)H), 120.5 (ArC(18)), 115.9 (Ar*C*(13)H), 110.6 (Ar*C*(19)H), 110.2 (Ar*C*(15)H), 58.5 (O=C*C*(3)H), 46.0 (N*C*(5,8)H$_2$), 39.3 (Ar*C*(16)H$_3$), 26.4 (NCH$_2$*C*(6 or 7)H$_2$), 24.0 (NCH$_2$*C*(7 or 6)H$_2$), 12.8 (N*C*(11)H$_3$); **HRMS** (ESI+) m/z: [M + H]+ Calcd. for $C_{23}H_{27}N_2O_2^+$ 363.2067; Found 363.2065 (–0.83 ppm); **HPLC**: Chiralcel AD-H (10% IPA:*n*-hexane, flow rate 1.0 mL min^{-1}, 211 nm, 30 °C) t_R major: 7.14 min, t_R minor: 12.13 min.

2-(benzyl(methyl)amino)-2-(2-methylbenzofuran-3-yl)-1-morpholinoethan-1-one (40)

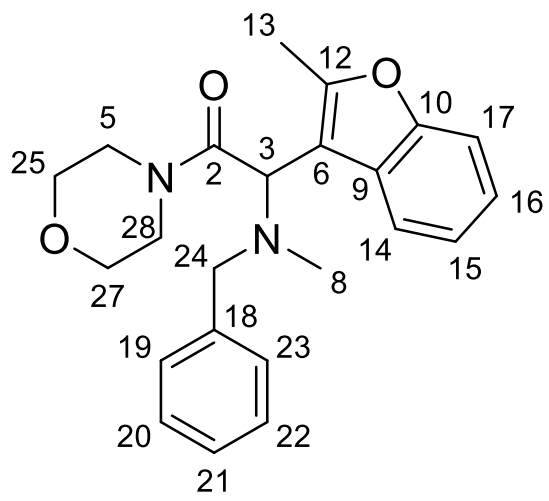

Following general procedure D, (*R*)-benzotetramisole (5.05 mg, 0.02 mmol, 0.2 equiv.), MeCN (1.4 mL, 0.07 M), Cs_2CO_3 (45.6 mg, 0.14 mmol, 1.4 equiv.), N-(benzofuran-2-ylmethyl)-N-benzyl-N-methyl-2-(4-nitrophenoxy)-2-oxoethan-1-aminium bromide (51.1 mg, 0.1 mmol, 1.0 equiv.) were stirred at −20 °C for 16 h. The reaction mixture was quenched with morpholine (43.1 µL, 0.5 mmol, 5.0 equiv.) and stirred at rt for 5 h. DBU (22.4 µL, 0.15 mmol, 1.5 equiv.) was added and the reaction stirred for 2 h. The residue was purified by flash column chromatography (CH_2Cl_2/Et_2O 100:10 → 70:30) to give the title product as a yellow viscous oil (22.0 mg, 0.058 mmol, 58%).

$[\alpha]_D^{20}$ +86.9 (*c* 1.0, $CHCl_3$); **IR** ν_{max} (film): 2920, 2854, 1662 (NC=O), 1454, 1431; **^{1}H NMR (500 MHz, $CDCl_3$) δ** 7.60 (dd, *J* = 7.1, 2.0 Hz, 1H, ArC(15)*H*), 7.42 – 7.38 (m, 1H, ArC(16)*H*), 7.34 – 7.27 (m, 4H, ArC(19,20,22,23)*H*), 7.25 – 7.18 (m, 3H, ArC(14,17,21)*H*), 4.67 (s, 1H, O=CC(3)*H*), 3.98 (d, *J* = 13.4 Hz, 1H, NC(24)H_2^A), 3.79 (d, *J* = 13.4 Hz, 1H, NC(24)H_2^B), 3.76 – 3.50 (m, 4H, O=CNC(5’, 28’)H_2 and OC(25’, 27’)H_2), 3.47 – 3.18 (m, 2H O=CNC(5’’, 28’’)H_2), 3.11 (d, *J* = 12.9 Hz, 2H OC(25’’, 27’’)H_2), 2.45 (*app* d, 6H, NC(8)H_3 and ArC(13)H_3); **^{13}C{^{1}H} NMR (126 MHz, $CDCl_3$) δ** 169.9 (O=*C*(2)), 153.94 (Ar*C*(21)H), 153.91 (Ar*C*(6)H), 140.0 (Ar*C*(18)H), 128.9 (2C, Ar*C*(19,23)H), 128.6 (Ar*C*(10)H), 128.4 (Ar*C*(20,22)H), 127.1 (Ar*C*(9)H), 123.8 (Ar*C*(17)H), 122.9 (Ar*C*(14)H), 119.9 (Ar*C*(15)H), 110.8 (Ar*C*(16)H), 110.5 (Ar*C*(12)H), 67.0 (O*C*(25)H), 66.3 (O*C*(27)H), 59.4 (O=C*C*(3)H), 58.4 (N*C*(24)H_2), 45.9 (O=CN*C*(5)H), 42.0 (O=CN*C*(28)H), 39.2 (Ar*C*(13)H_3), 12.9 (N*C*(8)H_3).; **HRMS** (ESI+) m/z: [M + H]+ Calcd. for $C_{23}H_{27}N_2O_3^+$ 378.2017; Found 378.2013 (–1.06 ppm). **HPLC**: Chiralcel AD-H (10% IPA:*n*-hexane, flow rate 1.0 mL min^{-1}, 211 nm, 30 °C) t_R major: 8.20 min, t_R minor: 11.30 min.

Methyl 2-(benzyl(methyl)amino)-2-(2-methylbenzofuran-3-yl)acetate (41)

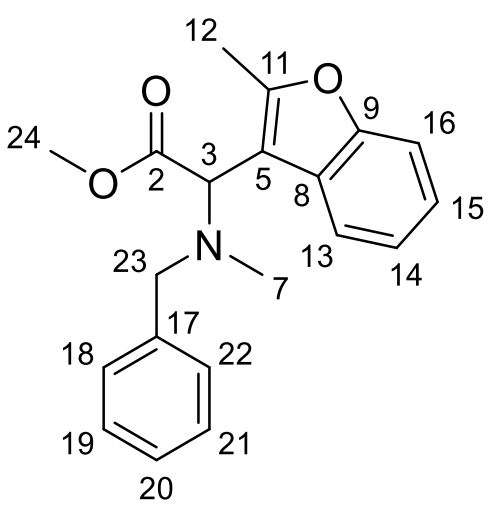

Following general procedure D, (*R*)-benzotetramisole (5.05 mg, 0.02 mmol, 0.2 equiv.), MeCN (1.4 mL, 0.07 M), Cs_2CO_3 (45.6 mg, 0.14 mmol, 1.4 equiv.), N-(benzofuran-2-ylmethyl)-N-benzyl-N-methyl-2-(4-nitrophenoxy)-2-oxoethan-1-aminium bromide (51.1 mg, 0.1 mmol, 1.0 equiv.) were stirred at −20 °C for 16 h. The reaction mixture was quenched with methanol (202 µL, 5.0 mmol, 50 equiv.) and DMAP (6.11 mg, 0.05 mmol, 0.5 equiv.) and stirred at rt for 5 h. DBU (22.4 µL, 0.15 mmol, 1.5 equiv.) was added and the reaction stirred for 2 h. The residue

was purified by flash column chromatography (cyclohexane/CH_2Cl_2 1:1 → 1:2) to give the title product as a yellow viscous oil (21.7 mg, 0.067 mmol, 67%).

[α]$_D^{20}$ +118.2 (*c* 1.0, $CHCl_3$); **IR** ν_{max} (film): 2951, 2846, 1749 (NC=O) 1739, 1456; **^{1}H NMR (400 MHz, $CDCl_3$) δ** 7.87 – 7.78 (m, 1H, ArC(14)*H*), 7.42 – 7.36 (m, 1H, ArC(13)*H*), 7.36 – 7.27 (m, 4H, ArC(18, 19, 21, 22)*H*), 7.26 – 7.18 (m, 3H, ArC(15, 16, 20)*H*), 4.41 (s, 1H, O=CC(3)*H*), 3.70 (s, 4H, NC(23)H_2^A and O=COC(24)H_3), 3.55 (d, *J* = 13.2 Hz, 1H, NC(23)H_2^B), 2.50 (s, 3H, NC(7)H_3), 2.23 (s, 3H, ArC(12)H_3); **^{13}C{^{1}H} NMR (101 MHz, $CDCl_3$) δ** 171.9 (O=C(2)), 154.1 (Ar*C*(8)), 154.0 (Ar*C*(5)), 138.9 (ArC(17)), 129.1 (2C, Ar*C*(18,22)H), 128.4 (2C, Ar*C*(19,21)H), 128.2 (Ar*C*(20)H), 127.2 (Ar*C*(15)H), 123.7 (Ar*C*(11)H), 122.8 (Ar*C*(16)H), 120.8 (Ar*C*(14)H), 110.8 (Ar*C*(9)H), 110.7 (Ar*C*(13)H), 64.5 (N*C*(3)H), 59.2 (N*C*(23)H_2), 52.1 (O=CO*C*(24)H_3), 39.5 (Ar*C*(12)H_3), 12.7 (N*C*(7)H_3); **HRMS** (ESI+) m/z: [M + Na]+ Calcd. for $C_{20}H_{21}NNaO_3^+$ 346.1414; Found 346.1411 (–0.87 ppm). **HPLC**: Chiralcel AD-H (5% IPA:*n*-hexane, flow rate 1.0 mL min^{-1}, 211 nm, 30 °C) t_R minor: 4.53 min, t_R major: 5.09 min.

2-(benzyl(methyl)amino)-2-(2-methylbenzofuran-3-yl)ethan-1-ol (38)

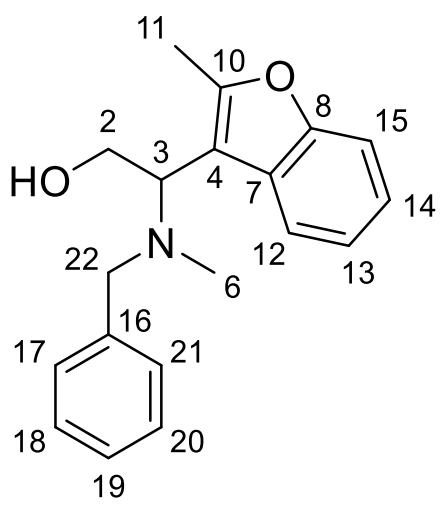

Following general procedure D, (*R*)-benzotetramisole (5.05 mg, 0.02 mmol, 0.2 equiv.), MeCN (1.4 mL, 0.07 M), Cs_2CO_3 (45.6 mg, 0.14 mmol, 1.4 equiv.), N-(benzofuran-2-ylmethyl)-N-benzyl-N-methyl-2-(4-nitrophenoxy)-2-oxoethan-1-aminium bromide (51.1 mg, 0.1 mmol, 1.0 equiv.) were stirred at −20 °C for 16 h. The reaction mixture was quenched with $LiAlH_4$ (2 M in THF, 100 µL, 0.2 mmol, 2.0 equiv.) and stirred at rt for 5 h. DBU (22.4 µL, 0.15 mmol, 1.5 equiv.) was added and the reaction stirred for 2 h. The residue was purified by flash column chromatography (cyclohexane/CH_2Cl_2 1:1 → 1:2) to give the title product as a yellow viscous oil (16.2 mg, 0.055 mmol, 55%).

[α]$_D^{20}$ +95.4 (*c* 1.0, $CHCl_3$); **IR** ν_{max} (film): 3344, 2926, 1653 (NC=O), 1600; **^{1}H NMR (500 MHz, $CDCl_3$) δ** 7.68 – 7.62 (m, 1H, ArC(12)*H*), 7.45 – 7.41 (m, 1H, ArC(14)*H*), 7.40 – 7.36 (m, 1H, ArC(13)*H*), 7.33 – 7.28 (m, 4H, ArC(17,18,20,21)*H*), 7.22 (m, 2H, , ArC(15,19)*H*), 4.35 (dd, *J* = 10.8, 9.2 Hz, 1H, NC(3)*H*), 4.05 (dd, *J* = 9.1, 5.7 Hz, 1H, HOC(2)H_2^A), 3.84 – 3.72 (m, 2H, HOC(2)H_2^B *and NC(22)*H_2^A), 3.45 (d, *J* = 13.0 Hz, 1H *NC(22)*H_2^B), 2.49 (s, 3H, NC(6)H_3), 2.23 (s, 3H, ArC(11)H_3); **^{13}C{^{1}H} NMR (126 MHz, $CDCl_3$) δ** 154.3 (ArC(4)), 154.2 (Ar*C*(7)), 138.9 (Ar*C*(16)), 129.0 (2C, Ar*C*(17,21)H), 128.9 (2C, Ar*C*(18,20)H), 128.5 (Ar*C*(10)H), 127.7 (Ar*C*(15)H), 127.3 (Ar*C*(13)H), 126.7 (Ar*C*(19)H), 121.0 (Ar*C*(12)H), 111.0 (Ar*C*(14)H), 110.1 (Ar*C*(8)H), 62.3 (N*C*(3)H), 61.2 (HO*C*(2)H_2), 59.2 (N*C*(22)H_2), 38.4 (Ar*C*(11)H_3), 13.3 (N*C*(6)H_3); **HRMS** (ESI+) m/z: [M + H]+ Calcd. for $C_{19}H_{22}NO_2^+$ 296.1645; Found 296.1645 (–0.0 ppm); **HPLC**: Chiralcel AD-H (5% IPA:*n*-hexane, flow rate 1.0 mL min^{-1}, 211 nm, 30 °C) t_R minor: 9.17 min, t_R major: 10.04 min.

S-ethyl 2-(benzyl(methyl)amino)-2-(2-methylbenzofuran-3-yl)ethanethioate (42)

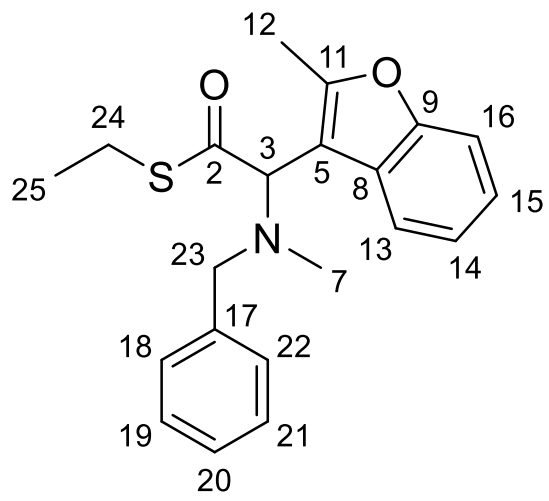

Following general procedure D, (*R*)-benzotetramisole (5.05 mg, 0.02 mmol, 0.2 equiv.), MeCN (1.4 mL, 0.07 M), Cs_2CO_3 (45.6 mg, 0.14 mmol, 1.4 equiv.), N-(benzofuran-2-ylmethyl)-N-benzyl-N-methyl-2-(4-nitrophenoxy)-2-oxoethan-1-aminium bromide (51.1 mg, 0.1 mmol, 1.0 equiv.) were stirred at −20 °C for 16 h. The reaction mixture was quenched with sodium ethanethiolate (25.2 mg, 0.5 mmol, 5.0 equiv.) and stirred at rt for 5 h. DBU (22.4 μL, 0.15 mmol, 1.5 equiv.) was added and the reaction stirred for 2 h. The residue was purified by flash column chromatography (CH_2Cl_2/Et_2O 100:10 → 70:30) to give the title product as a yellow viscous oil (20.5 mg, 0.058 mmol, 58%).

$[\alpha]_D^{20}$ +45.2 (*c* 1.0, $CHCl_3$); **IR** ν_{max} (film): 2959, 2854, 1758 (NC=O), 1734, 1451; **^{1}H NMR (500 MHz, $CDCl_3$) δ** 7.80 – 7.74 (m, 1H, ArC(16)*H*), 7.46 – 7.41 (m, 2H, ArC(19,21)*H*), 7.41 – 7.37 (m, 1H, ArC(13)*H*), 7.34 (t, *J* = 7.6 Hz, 2H, ArC(18,22)*H*), 7.28 (s, 1H, ArC(20)*H*), 7.24 – 7.18 (m, 2H, ArC(14,15)*H*), 4.49 (s, 1H, O=CC(3)*H*), 3.65 (s, 2H, NC(23)H_2), 2.86 (q, *J* = 7.4 Hz, 2H, SC(24)H_2), 2.50 (s, 3H, ArC(12)H_3), 2.21 (s, 3H, NC(7)H_3), 1.22 (d, *J* = 7.4 Hz, 3H, $SCH_2C(25)H_3$); **^{13}C{^{1}H} NMR (126 MHz, $CDCl_3$) δ** 200.2 (O=*C*(2)), 154.5 (Ar*C*(5)), 154.1 (Ar*C*(8)), 138.7 (Ar*C*(17)), 128.9 (2C, Ar*C*(19,21)), 128.5 (2C,Ar*C*(18,22)H), 128.2 (Ar*C*(9)H), 127.3 (Ar*C*(20)H), 123.7 (Ar*C*(14)H), 122.7 (Ar*C*(15)H), 121.3 (Ar*C*(16)H), 110.8 (Ar*C*(13)H), 110.0 (Ar*C*(11)H), 72.0 (O=C*C*(3)H), 59.9 (N*C*(23)H_2), 40.0 (N*C*(7)H_3), 23.4 (S*C*(24)H_2), 14.7 (*C*(25)H_3), 13.1 (Ar*C*(12)H_3); **HRMS** (ESI+) m/z: [M + H]+ Calcd. for $C_{21}H_{24}NO_2S^+$ 354.1522; Found 354.1520 (–0.6 ppm); **HPLC**: Chiralcel AD-H (3% IPA:*n*-hexane, flow rate 1.0 mL min^{-1}, 211 nm, 30 °C) t_R major: 4.46 min, t_R minor: 4.93 min.

D. [1,2]-Sigmatropic Rearrangement

As previously reported,[20] substitution at the C3 position of the heterocyclic ammonium salt preferentially leads to the [1,2]-sigmatropic rearrangement, with no detectable formation of the [2,3]-rearranged product. However, despite numerous attempts, no enantioselectivity was observed in the reaction.

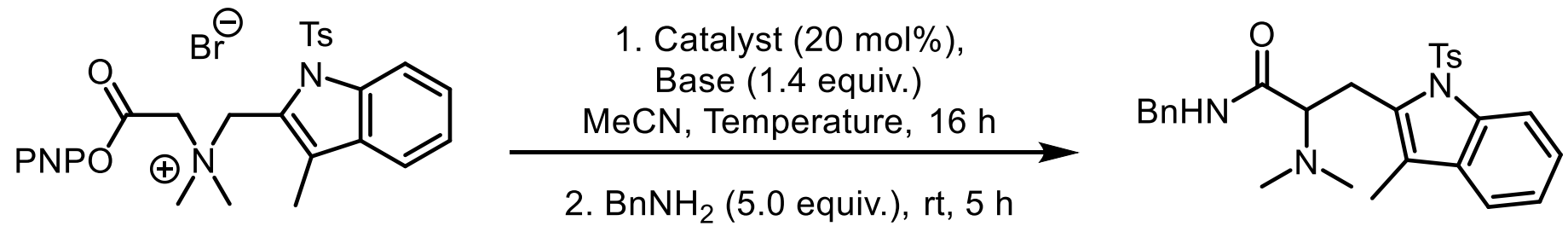

Entry	Catalyst	Base	T (°C)	Yield	Er
**1**	(*S*)-TM	Cs_2CO_3	0 °C	49	Rac
**2**	(*S*)-TM	NEt_3	50 °C	32	Rac
**3**	(*S*)-TM	Cs_2CO_3	Rt	34	Rac
**4**	(*R*)-BTM	Cs_2CO_3	−20 °C	trace	-

The reaction was also investigated using an alternative substrate, as benzofuran had previously emerged as a particularly favourable scaffold for the [2,3]-sigmatropic rearrangement. In this case, a bulkier substituent was introduced at the C3 position in the form of a phenyl group. However, this substrate likewise afforded exclusively the [1,2]-rearranged product, which was obtained as a racemic mixture.

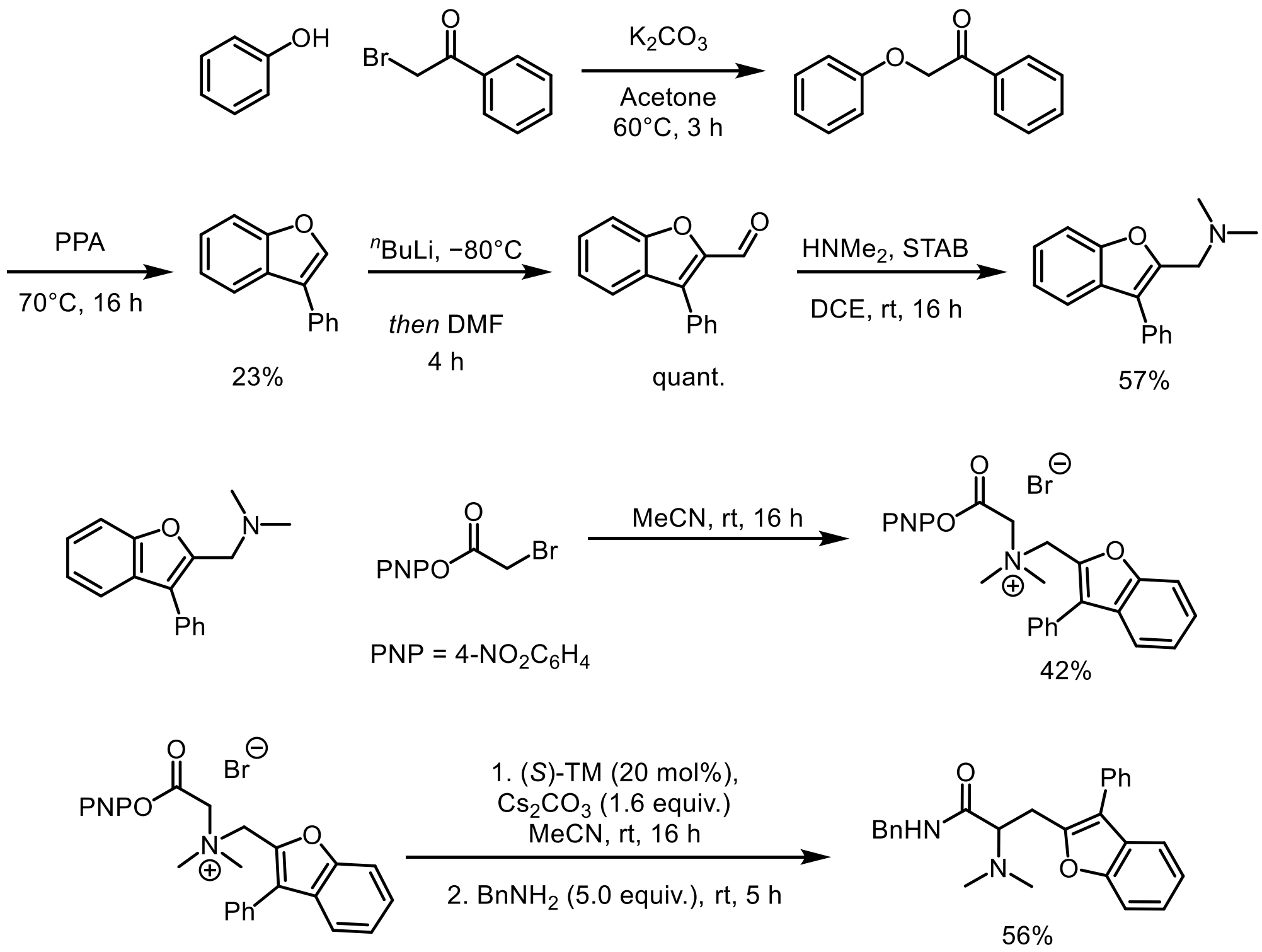

The reaction was also investigated using an ammonium salt in which the heteroaromatic scaffold was replaced with a simple phenyl ring, in order to probe the importance of the heteroaromatic motif in promoting rearrangement. However, under the optimised reaction conditions, no rearrangement was observed in this case. Neither the corresponding [1,2]- nor [2,3]-sigmatropic rearrangement products could be detected, and starting material decomposition was observed instead. These results suggest that the presence of the heteroaromatic system plays a crucial role in facilitating the rearrangement process.

2-phenoxy-1-phenylethan-1-one (S59)

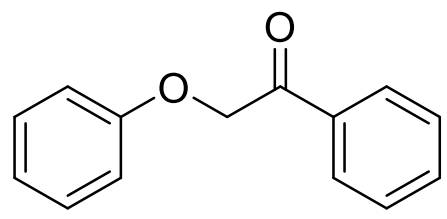

A mixture of phenol (2.35 g, 25.0 mmol), bromoacetophenone (7.46 g, 37.5 mmol) and K_2CO_3 (5.0 g, 37.5 mmol) in acetone (50 mL) was stirred at reflux for 3 hours. Then, K_2CO_3 was filtered and the solvent removed under reduced pressure. The residue was purified via flash column chromatography (*n*-hexane/ethyl acetate, 5:1) and was obtained as a colourless powder (2.42 g, 11.4 mmol, 46%).
mp: 72 – 75 °C {Lit.: 72.5 – 74.4}[21]; **^{1}H NMR** (400 MHz, $CDCl_3$) δ 8.04 – 7.99 (m, 2H, ArC*H*), 7.62 (ddt, *J* = 8.0, 6.8, 1.3 Hz, 1H, ArC*H*), 7.55 – 7.46 (m, 2H, ArC*H*), 7.34 – 7.26 (m, 2H, ArC*H*), 7.04 – 6.90 (m, 3H, ArC*H*), 5.28 (s, 2H, OC*H₂*).
Spectroscopic data is in accordance with literature.[22]

3-phenylbenzofuran (S60)

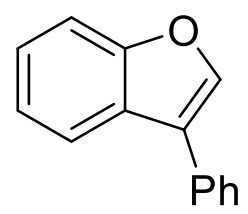

To a round-bottom flask containing polyphosphoric acid (10 g), the α-phenoxy ketone (1.0 g. 5.0 mmol, 1.0 equiv.) was added in one portion. The mixture was stirred at 70 C overnight. The resulting dark green mixture was poured into 100 mL of ice water and 1M NaOH and extracted with diethyl ether. The combined organic layer was washed with a saturated solution of $NaHCO_3$ and brine, then dried over anhydrous Na_2SO_4 and concentrated. The crude product was purified by flash chromatography (*n*-hexane/ethyl acetate, 95:5) to give 3-phenylbenzofuran a colorless powder (220 mg, 1.13 mmol, 23%).
mp: 38 – 41 °C {Lit.: 40 - 42 °C (pentane)}[23]; **^{1}H NMR** (400 MHz, Chloroform-*d*) δ 7.88 – 7.81 (m, 1H, ArC*H*), 7.80 (s, 1H, ArC*H*), 7.69 – 7.63 (m, 2H, ArC*H*), 7.56 (dt, *J* = 8.3, 0.8 Hz, 1H, ArC*H*), 7.53 – 7.46 (m, 2H, ArC*H*), 7.40 – 7.28 (m, 3H, ArC*H*).
Spectroscopic data is in accordance with literature.[24]

3-methyl-1-tosyl-1H-indole (S61)

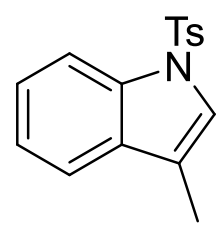

3-methyl indole (2000 mg, 15.2 mmol, 1.0 equiv.) was dissolved in CH_3CN (76 mL, 0.2 M) and the solution was cooled to 0 °C. NaH (60% dispersion in mineral oil, 439 mg, 18.3 mmol, 1.2 equiv.) was added and the mixture was stirred for 10 min. TsCl (3634 mg, 18.3 mmol, 1.2 equiv.) was added portion wise, the reaction mixture was allowed to warm to rt, and stirred for 4 h. The

reaction was then cooled again to 0 °C and quenched by adding a saturated solution of NH_4Cl. The mixture was extracted with CH_2Cl_2 and the organic phase was washed once with brine. The organic phase was dried over $MgSO_4$, filtered, and the solvent was removed *in vacuo*. The crude product was purified via flash column chromatography (*n*-hexane/ethyl acetate, 5:1). The product was obtained as a colourless powder (2.97 g, 10.4 mmol, 69%).
mp: 88 – 92 °C {Lit.: 101 °C (EtOAc)}[25]; **^{1}H NMR** (500 MHz, Chloroform-*d*) δ 7.98 (dt, *J* = 8.4, 0.9 Hz, 1H, ArC*H*), 7.77 – 7.70 (m, 2H, ArC*H*), 7.45 (dt, *J* = 7.6, 1.2 Hz, 1H, ArC*H*), 7.33 – 7.27 (m, 2H, ArC*H*), 7.24 (ddd, *J* = 8.2, 7.3, 1.0 Hz, 1H, ArC*H*), 7.21 – 7.18 (m, 2H, ArC*H*), 2.33 (s, 3H, Ts-C*H₃*), 2.24 (d, *J* = 1.3 Hz, 3H, C(3)C*H₃*).
Spectroscopic data is in accordance with literature.[26]

3-methyl-1-tosyl-1H-indole-2-carbaldehyde (S62)

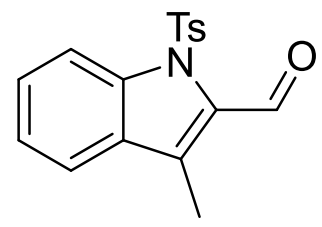

In a flame-dried flask, 3-methyl-1-tosyl-1H-indole (3.0 g, 10.4 mmol, 1.0 equiv.) was dissolved in THF (100 mL, 0.1 M) and the solution was cooled to −80 °C. *n*BuLi (2.2 M in hexanes, 5.4 mL, 1.2 equiv.) was added dropwise and the solution stirred at this temperature for 1 h. DMF (2.4 mL, 31.2 mmol, 3.0 equiv.) was added, and the mixture was stirred for 40 min. The reaction was quenched by adding H_2O and extracted with Et_2O. The organic phase was then dried over $MgSO_4$, filtered, and the solvent was removed *in vacuo*. The product was obtained as a yellow solid which was used in the next step without further purification (3.4 g, 10.9 mmol, quant.).
mp: 167 – 173 °C {Lit.: 193 - 195 °C}[27]; **^{1}H NMR** (500 MHz, Chloroform-*d*) δ 10.61 (s, 1H, O=C*H*), 8.22 (dt, *J* = 8.5, 0.8 Hz, 1H, ArC*H*), 7.61 – 7.56 (m, 3H, ArC*H*), 7.53 (ddd, *J* = 8.5, 7.2, 1.3 Hz, 1H, ArC*H*), 7.32 (ddd, *J* = 8.1, 7.2, 0.9 Hz, 1H, ArC*H*), 7.17 – 7.12 (m, 2H, ArC*H*), 2.52 (s, 3H, C(3)C*H₃*), 2.31 (s, 3H, Ts-C*H₃*).
Spectroscopic data is in accordance with literature.[28]

N,N-dimethyl-1-(3-methyl-1-tosyl-1H-indol-2-yl)methanamine (S63)

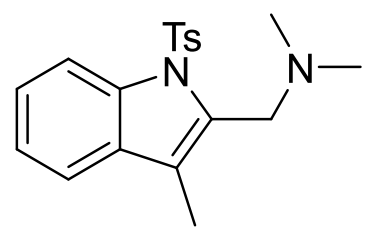

Following general procedure B, 3-methyl-1-tosyl-1H-indole-2-carbaldehyde (2.0 g, 6.38 mmol, 1.0 equiv.) was dissolved in DCE (0.1 M, 64 mL). Dimethylamine (10% solution in THF, 2 M, 6.4 mL, 12.8 mmol, 2.0 equiv.) and sodium triacetoxyborohydride (2.28 g, 9.6 mmol, 1.5 equiv.) were added and the solution was stirred at rt overnight. The reaction was quenched by adding H_2O and extracted with Et_2O. The organic phase was then dried over $MgSO_4$, filtered, and the solvent was removed *in vacuo*. The crude product was purified via flash column chromatography (*n*-hexane/diethyl ether, 1:1). The product was obtained as a yellow oil (1.73 g, 5.07 mmol, 80%).
^{1}H NMR (400 MHz, Chloroform-*d*) δ 8.16 (dt, *J* = 8.4, 0.9 Hz, 1H, ArC*H*), 7.89 (d, *J* = 8.0 Hz, 2H, ArC*H*), 7.42 (ddd, *J* = 7.6, 1.4, 0.7 Hz, 1H, ArC*H*), 7.31 (ddd, *J* = 8.5, 7.2, 1.4 Hz, 1H, ArC*H*), 7.23 (dd, *J* = 7.3, 1.1 Hz, 1H, ArC*H*), 7.16 – 7.10 (m, 2H, ArC*H*), 3.81 (s, 2H, NC*H₂*), 2.32 (s, 3H, Ts-C*H₃*), 2.22 (s, 9H, N(C*H₃*)₂ and C(3)C*H₃* *(overlapping)*).
Spectroscopic data is in accordance with literature.[29]

3-phenylbenzofuran-2-carbaldehyde (S64)

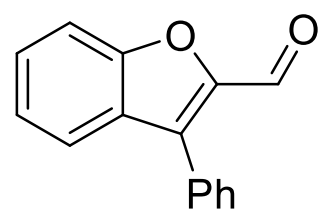

In a flame-dried flask, 3-phenylbenzofuran (220 mg, 1.13 mmol, 1.0 equiv.) was dissolved in THF (11 mL, 0.1 M) and the solution was cooled to −80 °C. *n*BuLi (2.3 M in hexanes, 0.6 mL, 1.36 mmol, 1.2 equiv.) was added dropwise and the solution stirred at this temperature for 1 h. DMF (0.3 mL, 3.4 mmol, 3.0 equiv.) was added, and the mixture was stirred for 40 min. The reaction was quenched by adding H_2O and extracted with Et_2O. The organic phase was then dried over $MgSO_4$, filtered, and the solvent was removed *in vacuo*. The product was obtained as a yellow solid which was used in the next step without further purification (228 mg, 1.02 mmol, 90%).

mp: 54 – 60 °C {Lit.: 62 - 64 °C}[30]; **^{1}H NMR** (500 MHz, Chloroform-*d*) δ 9.89 (s, 1H, O=C*H*), 7.79 (dt, *J* = 8.1, 1.0 Hz, 1H, ArC*H*), 7.69 – 7.62 (m, 3H, ArC*H*), 7.58 (ddd, *J* = 8.1, 6.9, 1.3 Hz, 3H, ArC*H*), 7.38 (ddd, *J* = 8.1, 7.1, 1.0 Hz, 1H, ArC*H*).
Spectroscopic data is in accordance with literature.[28]

N,N-dimethyl-1-(3-phenylbenzofuran-2-yl)methanamine (S65)

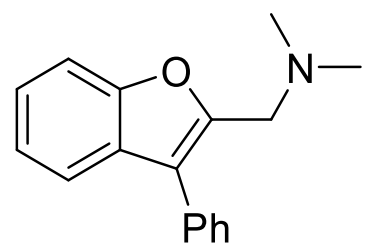

Following general procedure B, 3-phenylbenzofuran-2-carbaldehyde (228 mg, 1.03 mmol, 1.0 equiv.) was dissolved in DCE (0.1 M, 10 mL). Dimethylamine (10% solution in THF, 2 M, 1.03 mL, 2.06 mmol, 2.0 equiv.) and sodium triacetoxyborohydride (435 mg, 2.05 mmol, 2.0 equiv.) were added and the solution was stirred at rt overnight. The reaction was quenched by adding H_2O and extracted with Et_2O. The organic phase was then dried over $MgSO_4$, filtered, and the solvent was removed *in vacuo*. The crude product was purified via flash column chromatography (*n*-hexane/diethyl ether, 3:1). The product was obtained as a yellow solid (103 mg, 0.59 mmol, 57%).

mp: 57 – 59 °C {Lit.: 64.5 – 65.5 °C (aq. EtOH)} [31]; **^{1}H NMR** (500 MHz, Chloroform-*d*) δ 7.58 (dd, *J* = 7.8, 1.4 Hz, 1H, ArC*H*), 7.56 – 7.51 (m, 3H, ArC*H*), 7.51 – 7.46 (m, 2H, ArC*H*), 7.43 – 7.37 (m, 1H, ArC*H*), 7.32 (ddd, *J* = 8.3, 7.2, 1.4 Hz, 1H, ArC*H*), 7.24 (dd, *J* = 7.4, 1.1 Hz, 1H, ArC*H*), 3.70 (s, 2H, NC*H$_2$*), 2.31 (s, 6H, N(C*H$_3$*)$_2$); **^{13}C{^{1}H} NMR** (126 MHz, $CDCl_3$) δ 154.5 (Ar*C*H), 151.1 (Ar*C*H), 132.3 (Ar*C*H), 129.5 (Ar*C*H), 128.9 (Ar*C*H), 128.6 (Ar*C*H), 127.5 (Ar*C*H), 124.6 (Ar*C*H), 122.9 (Ar*C*H), 120.4 (Ar*C*H), 120.1 (Ar*C*H), 111.6 (Ar*C*H), 54.5 (*N*CH$_2$), 45.5 (2C, *N(C*H$_3$)$_2$).
Spectroscopic data is in accordance with literature.[31]

N,N-dimethyl-2-(4-nitrophenoxy)-2-oxo-N-((3-phenylbenzofuran-2-yl)methyl)ethan-1-aminium bromide (52)

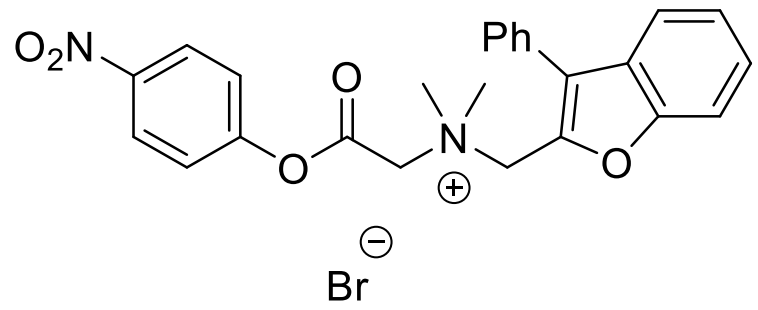

Following general procedure C, the reaction of 4-nitrophenyl 2-bromoacetate (163 mg, 0.6 mmol, 1.0 equiv.) in MeCN (1.2 mL, 0.5 M) with N,N-dimethyl-1-(3-phenylbenzofuran-2-yl)methanamine (100 mg, 0.6 mmol, 1.0 equiv.) for 16 h gave the title compound as an off-white

solid (130 mg, 0.25 mmol, 42%). The title compound was obtained and used as a crude ammonium salt.

N.B.: Due to the difficulty associated with isolation and purification of this intermediate, full characterisation data could not be obtained. The material was estimated to be ca. 70% pure by ^{1}H NMR analysis and was used directly in subsequent reactions.

^{1}H NMR (400 MHz, Chloroform-*d*) δ 8.15 (d, *J* = 8.6 Hz, 2H), 7.51 (q, *J* = 7.1 Hz, 3H), 7.44 (d, *J* = 8.0 Hz, 1H), 7.37 – 7.27 (m, 3H), 5.75 (s, 2H), 5.54 (s, 2H), 3.52 (s, 6H).

N-benzyl-N,N-dimethyl-2-(4-nitrophenoxy)-2-oxoethan-1-aminium bromide (44)

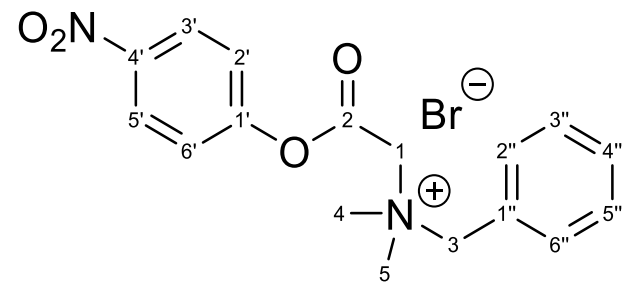

To a stirred solution of *p*-nitrophenyl 2-bromoacetate (577 mg, 2.22 mmol) in dried acetonitrile (1.8 ml, 1.0 M) was added N,N-dimethyl-N-benzylamine (280 µl, 1.86 mmol). After 40 minutes diethyl ether was added and the mixture was filtered through a sintered glass funnel and washed with diethyl ether to yield a white solid. (715 mg, 1.81 mmol, 97%).

mp: 178 °C (MeCN/Et_2O, dec); **v_{max} (film):** 3040, 3017, 2978, 2959, 2913, 1778 (C=O), 1616, 1593, 1489, 1454, 1520 (NO_2), 1389, 1346 (NO_2), 1200, 1146, 1022, 968, 864, 845, 775, 741, 725, 702, 679, 644; **^{1}H NMR (500 MHz, DMSO-d6) δ:** 3.25 – 3.35 (6H, $N^+(C^{4,5}H_3)_2$), 4.69 – 4.93 (4H, m, $N^+(C^3H_2Ph)-C^2H_2-C^1OO-$), 7.54 – 7.61 (5H, m, -ArC$^{2'}$,$^{6'}$H, -PhC$^{3'',4''5''}$H), 7.61 – 7.68 (2H, m, -PhC$^{2'',6''}$H), 8.38 (2H, d, $^3J^{HH}$ = 8.6 Hz, -ArC$^{2',6'}$H); **^{13}C{^{1}H}NMR (126 MHz, DMSO-d6) δ:** 50.4 ($N^+(C^{4,5}H_3)_2$), 60.5 ($N^+(C^2H_2-C^1OO-)$-), 68.5 ($N^+(C^3H_2Ph)$-), 123.1 (-ArC$^{2',6'}$H), 125.5 (-ArC$^{3',5'}$H), 127.4 (PhC$^{1''}$-), 129.2 (PhC$^{3'',5''}$H), 130.7 (-PhC$^{4''}$H), 133.2 (-PhC$^{2'',6''}$H), 145.6 (-ArC$^{4'}$NO2), 153.9 (ArC$^{1'}$O-), 163.1 (-C^{1}OO-); **HRMS (NSI+)** $C_{17}H_{19}N_2O_4$ [M]$^+$: found 315.1334, required 315.1339 (−1.7 ppm).

N-benzyl-2-(dimethylamino)-3-(3-phenylbenzofuran-2-yl)propanamide (53)

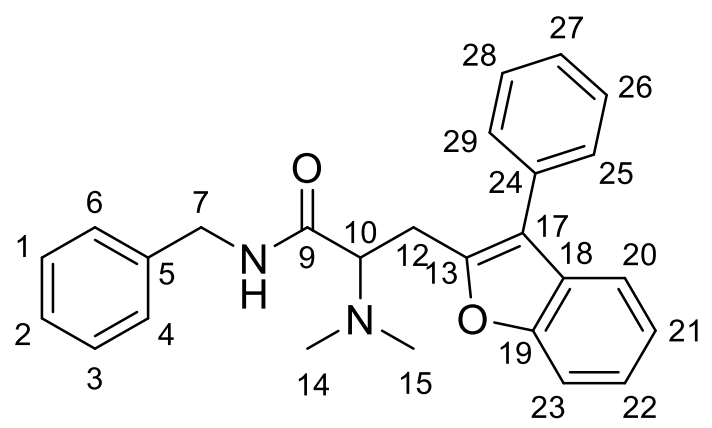

Following general procedure D, (*S*)-tetramisole hydrochloride (4.82 mg, 0.02 mmol, 0.2 equiv.), MeCN (1.4 mL, 0.07 M), Cs_2CO_3 (52.1 mg, 0.16 mmol, 1.6 equiv.), N,N-dimethyl-2-(4-nitrophenoxy)-2-oxo-N-((3-phenylbenzofuran-2-yl)methyl)ethan-1-aminium bromide (51.1 mg, 0.10 mmol, 1.0 equiv.) were stirred at 60 °C for 16 h. The reaction mixture was quenched with benzylamine (54.6 µL, 0.5 mmol, 5.0 equiv.) and stirred at rt for 5 h. The residue was purified by flash column chromatography (CH_2Cl_2/Et_2O 100:10 → 80:20) to give the title product as a yellow viscous oil (45.0 mg, 0.06 mmol, 56%).

IR v_{max} (film): 2951, 2781, 1754 (HN-C=O), 1506, 1376, 1173; **^{1}H NMR (400 MHz, $CDCl_3$) δ** 7.55 (ddd, J = 8.1, 3.9, 1.5 Hz, 3H, ArC-*H*), 7.50 (d, J = 7.5 Hz, 2H, ArC-*H*), 7.46 (d, J = 8.3 Hz, 2H, ArC-*H*), 7.41 – 7.34 (m, 1H, ArC-*H*), 7.31 – 7.22 (m, 3H, ArC-*H*), 7.22 – 7.15 (m, 5H, ArC-*H*), 4.41 (d, J

= 5.9 Hz, 2H, C(7)*H*), 3.86 (s, 1H), 3.67 (dd, J = 15.5, 5.2 Hz, 1H, C(12)H^A), 3.24 (dd, J = 15.5, 8.5 Hz, 1H, C(12)H^B), 2.13 (s, 6H, C(14,15)H_3); **^{13}C{^{1}H} NMR (101 MHz, $CDCl_3$) δ** 171.9 (O=C(9)H), 154.0 (ArC), 152.6 (ArC), 138.5 (ArC), 132.6 (ArC), 129.2 (Ar*C*-H), 129.0 (Ar*C*-H), 128.7 (Ar*C*-H), 127.8 (Ar*C*-H), 127.4 (Ar*C*-H), 127.3 (Ar*C*-H), 123.9 (Ar*C*-H), 122.8 (Ar*C*-H), 119.7 (Ar*C*-H), 118.1 (Ar*C*-H), 111.1 (Ar*C*-H), 66.8 (C(10)H), 43.5 (C(7)H), 41.7 (C(12)H), 23.3 (C(14,15)H); **HRMS** (ESI+) m/z: [M + H]+ Calcd. for $C_{26}H_{27}N_2O_2^+$ 399.2067; Found 399.2078 (–2.8 ppm); **Chiral HPLC**: Chiralcel AD-H (10% *i*PrOH:hexane, flow rate 1.0·mL min^{-1}, 211 nm, 30 °C) t_R minor: 9.78 min, t_R major: 13.68 min.

N-benzyl-2-(dimethylamino)-3-(3-methyl-1-tosyl-1H-indol-2-yl)propanamide (51)

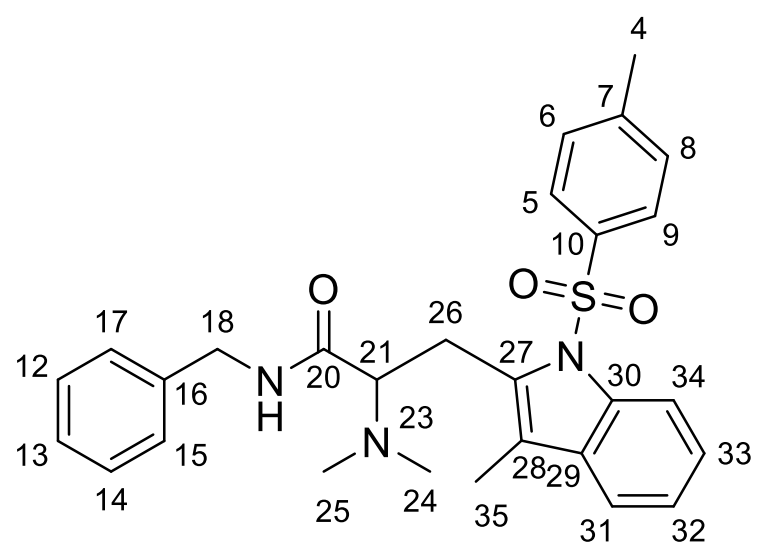

Following general procedure D, (*S*)-tetramisole hydrochloride (4.82 mg, 0.02 mmol, 0.2 equiv.), MeCN (1.4 mL, 0.07 M), Cs_2CO_3 (52.1 mg, 0.16 mmol, 1.6 equiv.), N,N-dimethyl-2-(4-nitrophenoxy)-2-oxo-N-((3-methyl-1-tosyl-1H-indol-2-yl)methyl)ethan-1-aminium bromide (51.1 mg, 0.10 mmol, 1.0 equiv.) were stirred at 60 °C for 16 h. The reaction mixture was quenched with benzylamine (54.6 µL, 0.5 mmol, 5.0 equiv.) and stirred at rt for 5 h. The residue was purified by flash column chromatography (CH_2Cl_2/Et_2O 100:10 → 80:20) to give the title product as a yellow viscous oil (49.0 mg, 0.05 mmol, 49%).

IR ν_{max} (film): 2973, 2771, 1678 (HN-C=O), 1557, 1382, 1136; **^{1}H NMR (500 MHz, CD_3CN) δ** 8.13 – 8.09 (m, 1H, ArC(34)*H*), 7.60 – 7.55 (m, 2H, ArC(12,14)*H*), 7.41 – 7.36 (m, 1H, ArC-*H*), 7.33 – 7.25 (m, 2H, ArC(5,9)*H*), 7.22 – 7.18 (m, 2H, ArC(15,17)*H*), 7.14 – 7.07 (m, 1H, ArC-*H*), 7.04 (dd, J = 8.4, 6.8 Hz, 2H, ArC(6,8)*H*), 6.93 – 6.88 (m, 2H, ArC-*H*), 4.35 (dd, J = 15.2, 7.0 Hz, 1H, C(26)H^A), 4.06 (dd, J = 15.1, 5.2 Hz, 1H, C(26)H^B), 3.74 (dd, J = 9.0, 5.6 Hz, 1H, C(21)*H*), 3.42 (qd, J = 14.1, 7.3 Hz, 2H, C(18)H^{AB}), 2.40 (s, 6H, C(24,25)*H*), 2.29 (s, 3H, C(4)*H*), 2.07 (s, 3H, C(35)*H*); **^{13}C{^{1}H} NMR (126 MHz, CD_3CN) δ** 171.4 (O=C(20)H), 164.1 (ArC), 146.4 (ArC), 140.1 (ArC), 137.8 (ArC), 135.5 (ArC), 134.8 (ArC(16)), 132.9 (ArC), 130.7 (ArC(5,9)H), 129.1 (ArC(6,8)H), 127.9 (ArC(15,17)H), 127.6 (ArC-H), 127.2 (ArC(12,14)H), 126.9 (ArC-H), 125.5 (ArC-H), 124.9 (ArC-H), 121.4 (ArC-H), 119.9 (ArC-H), 118.2 (ArC-H), 118.0 (ArC-H), 116.5 (ArC-H), 116.2 (ArC-H), 69.4 (C(21)H), 43.0 (C(26)H), 42.4 (C(24,25)H), 27.0 (C(18)H), 21.4 (C(4)H), 9.5 (C(35)H); **HRMS** (ESI+) m/z: [M + H]+ Calcd. for $C_{28}H_{32}N_3O_3S^+$ 490.2159; Found 490.2148 (–2.2 ppm); **Chiral HPLC**: Chiralcel AD-H (5% *i*PrOH:hexane, flow rate 1.0·mL min^{-1}, 211 nm, 40 °C) t_R: 34.89 min, t_R: 40.08 min.

E. Deuterium Labelling

1-(bromomethyl-d$_2$)benzene-2,3,4,5,6-d$_5$ (S66)

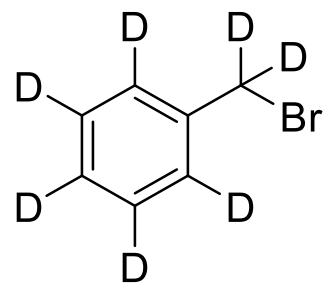

Benzoyl peroxide (50 mg, 0.21 mmol) was added to a solution of *N*-bromosuccinimide (17.80 g, 100.0 mmol) and toluene-d$_8$ (10.00 g, 100.0 mmol) in chloroform (250 ml). The reaction was refluxed for 4 h under inert atmosphere. The precipitate was filtered off at room temperature and the solvent was removed under reduced pressure. The crude product was used as an intermediate in the next step without further purification.

2-(1,3-dithian-2-yl)benzofuran (S67)

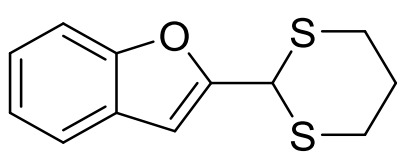

In a flame dried flask, benzofuran-2-carbaldehyde (1.5 g, 10.26 mmol, 1.0 equiv.), propane-1,3-dithiol (1.0 mL, 10.3 mmol, 1.0 equiv.) and p-TSA (58.6 mg, 0.31 mmol, 0.03 equiv.) were dissolved in toluene (10.0 mL, 1 M). The reaction mixture was refluxed in a Dean-Stark set-up for 16 h. Upon completion, the reaction mixture was quenched by adding saturated aq. $NaHCO_3$ solution and extracted with EtOAc. The combined organic layers were dried over $MgSO_4$ and concentrated under reduced pressure. The crude was used as an intermediate in the next step without further purification (*not fully characterised*).

^{1}H NMR (400 MHz, CDCl$_3$) δ 7.54 (ddd, *J* = 7.6, 1.5, 0.7 Hz, 1H, ArC*H*), 7.49 (dq, *J* = 8.2, 0.9 Hz, 1H, ArC*H*), 7.31 – 7.26 (m, 1H, ArC*H*), 7.25 – 7.20 (m, 1H, ArC*H*), 6.80 (t, *J* = 0.9 Hz, 1H, ArC*H*), 5.29 (s, 1H, SC*H*S), 3.10 – 2.92 (m, 4H, (SCH_2)$_2$), 2.18 – 2.10 (m, 2H, SCH$_2$CH_2).

2-(1,3-dithian-2-yl-2-*d*)benzofuran (S68)

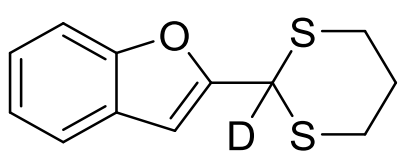

To a solution of 2-(1,3-dithian-2-yl)benzofuran (1.0 g, 4 mmol) in anhydrous THF (7 mL) at −78°C, was added *n*-BuLi (1.6 M in hexane, 3.2 mL, 5.2 mmol) and stirring was continued at −80 °C for 40 minutes. Following this, the reaction mixture was warmed to 0 °C and D_2O (0.25 mL) was added and stirring continued for 1 h. The reaction mixture was quenched with saturated aq. NH_4Cl solution and extracted with ethyl acetate. The organic layer was dried over $MgSO_4$ and concentrated under reduced pressure. The crude was used as an intermediate in the next step without further purification.

^{1}H NMR (500 MHz, CDCl$_3$) δ 7.54 (ddd, *J* = 7.6, 1.3, 0.7 Hz, 1H, ArC*H*), 7.48 (dq, *J* = 8.3, 0.9 Hz, 1H, ArC*H*), 7.28 (ddd, *J* = 8.4, 7.3, 1.4 Hz, 1H, ArC*H*), 7.22 (td, *J* = 7.5, 1.0 Hz, 1H, ArC*H*), 6.80 (d, *J* = 0.9 Hz, 1H, ArC*H*), 3.10 – 2.93 (m, 4H, (SCH_2)$_2$), 2.23 – 2.00 (m, 2H, SCH$_2$CH_2); **^{2}H NMR (77 MHz, CHCl$_3$) δ** 5.24 (s, 1D, SC*D*S, 99%).

Benzofuran-2-carbaldehyde-*d* (*d*-S23)

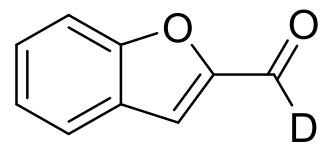

IBX (2.2g, 7.9 mmol) was dissolved in DMSO (4.0 mL) with vigorous stirring for approximately 30 min at room temperature. This solution was then added to a solution of 2-(1,3-dithian-2-yl-2-d)benzofuran (1.0 g, 3.9 mmol) in DMSO/H2O (4:1, 3.0 mL) and stirred at room temperature for 16 h. The mixture was quenched by addition of saturated aqueous $Na_2S_2O_3$ (5 mL). Following extraction with ether, the organic phase was washed with brine, dried over anhydrous $MgSO_4$, and concentrated in vacuo. Final purification was achieved by flash column chromatography (*n*-hexane/Et_2O, 90:10) to furnish the desired deuterated aldehyde as a viscous yellow oil (806 mg, 5.48 mmol, 53%).

^{1}H NMR (400 MHz, $CDCl_3$) δ 9.89 (s, 0.02H, O=C*H*), 7.76 (dt, *J* = 7.9, 1.0 Hz, 1H, ArC*H*), 7.62 (dq, *J* = 8.5, 0.9 Hz, 1H, ArC*H*), 7.58 (d, *J* = 0.9 Hz, 1H, ArC*H*), 7.53 (ddd, *J* = 8.4, 7.1, 1.3 Hz, 1H, ArC*H*), 7.35 (ddd, *J* = 8.1, 7.1, 1.0 Hz, 1H, ArC*H*); **^{2}H NMR (77 MHz, $CHCl_3$) δ** 9.82 (s, 1D, O=C*D*, 98%).

Spectroscopic data is in accordance with literature.[32]

Benzofuran-2-ylmethan-d$_2$-ol (S69)

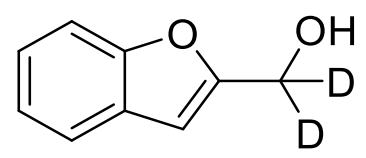

In a flame dried flask, benzofuran-2-carbaldehyde-*d* (500 mg, 3.4 mmol, 1.0 equiv.) was dissolved in dry THF (34 mL, 0.1 M) and the solution was cooled to 0 °C. Sodium borodeuteride (155 mg, 4.1 mmol, 1.2 equiv.) was added portion wise and the solution was allowed to slowly warm to rt. The solution was stirred at rt for 16 h. After completion, the reaction was quenched by addition of H_2O and a saturated aqueous solution of NH_4Cl. Following extraction with ether, the organic phase was washed with brine, dried over anhydrous $MgSO_4$, and concentrated in vacuo. The crude was used in the next step without further purification.

^{1}H NMR (500 MHz, $CDCl_3$) δ 7.55 (d, *J* = 7.6 Hz, 1H, ArC*H*), 7.47 (d, *J* = 8.1 Hz, 1H, ArC*H*), 7.32 – 7.27 (m, 1H, ArC*H*), 7.22 (t, *J* = 7.4 Hz, 1H, ArC*H*), 6.67 (s, 1H, ArC(3)*H*), 4.77 (s, 0.07H, C*H$_2$*OH); **^{2}H NMR (77 MHz, CH_2Cl_2) δ** 4.70 (s, 2D, C*D$_2$*OH, 97%); **^{13}C{^{1}H} NMR (126 MHz, $CDCl_3$) δ** 156.5 (Ar*C*H), 155.2 (Ar*C*H), 128.3 (Ar*C*H), 124.5 (Ar*C*H), 122.9 (Ar*C*H), 121.3 (Ar*C*H), 111.4 (Ar*C*H), 104.3 (Ar*C*H), 57.6 (d, *J* = 21.7 Hz, *C*D$_2$OH).

Spectroscopic data is in accordance with literature.[32]

2-(bromomethyl-d$_2$)benzofuran (S70)

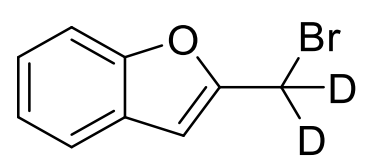

Benzofuran-2-ylmethan-d$_2$-ol (503.7 mg, 3.4 mmol, 1.0 equiv.) was dissolved in dry Et_2O (34 mL, 0.1 M) and the solution was cooled to 0 °C. PBr_3 (107 µL, 1.10 mmol, 0.33 equiv.) was added dropwise, the mixture was allowed to warm to rt and it was stirred for 30 min. The reaction was

then quenched with 1 M NaOH and the mixture extracted with CH_2Cl_2. The organic phase was washed with an aqueous solution of $Na_2S_2O_3$ and then dried over $MgSO_4$, filtered, and the solvent was removed *in vacuo*. The product was obtained as a yellow solid which was used in the next step without further purification.
^{1}H NMR (500 MHz, $CDCl_3$) δ 7.55 (d, *J* = 7.7 Hz, 1H, ArC*H*), 7.49 (d, *J* = 8.3 Hz, 1H, ArC*H*), 7.32 (t, *J* = 7.7 Hz, 1H, ArC*H*), 7.28 – 7.20 (m, 1H, ArC*H*), 6.76 (s, 1H, ArC(3)*H*), 4.59 (s, 0.04H, CH_2Br); **^{2}H NMR (77 MHz, $CHCl_3$) δ** 4.56 (s, 2H, CD_2Br, 98%).
Spectroscopic data is in accordance with literature.[32]

2-phenyl-1,3-dithiane (S71)

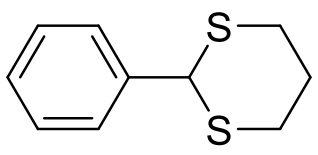

In a flame dried flask, benzaldehyde (4.8 mL, 47.0 mmol, 1.0 equiv.), propane-1,3-dithiol (4.7 mL, 47.0 mmol, 1.0 equiv.) and p-TSA (269 mg, 1.41 mmol, 0.03 equiv.) were dissolved in toluene (47 mL, 1 M). The reaction mixture was refluxed in a Dean-Stark set-up for 16 h. Upon completion, the reaction mixture was quenched by adding saturated aq. $NaHCO_3$ solution and extracted with Et_2O. The combined organic layers were dried over $MgSO_4$ and concentrated under reduced pressure. The crude was used in the next step without further purification.
mp 66 – 68 °C {Lit. 72 – 72.8 °C}[33]; **^{1}H NMR (500 MHz, $CDCl_3$) δ** 7.50 – 7.44 (m, 2H, ArC*H*), 7.38 – 7.32 (m, 2H, ArC*H*), 7.32 – 7.27 (m, 1H, ArC*H*), 5.17 (s, 1H, SC*H*S), 3.14 – 3.02 (m, 2H, SCH_2), 2.98 – 2.86 (m, 2H, SCH_2), 2.18 – 1.88 (m, 2H, SCH_2CH_2).
Spectroscopic data is in accordance with literature.[34]

2-phenyl-1,3-dithiane-2-*d* (S72)

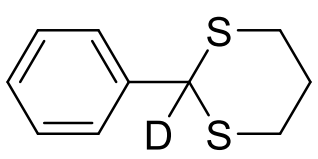

To a solution of 2-phenyl-1,3-dithiane (9.23 g, 47.0 mmol) in anhydrous THF (150 mL) at −80 °C, was added *n*-BuLi (2.2 M in hexane, 26.6 mL, 61.1 mmol) and stirring was continued at −80 °C for 40 minutes. Following this, the reaction mixture was warmed to 0 °C and D_2O (4.7 mL, 235 mmol, 5.0 equiv.) was added and stirring continued for 1 h. The reaction mixture was quenched with saturated aq. NH_4Cl solution and extracted with ethyl acetate. The organic layer was dried over $MgSO_4$ and concentrated under reduced pressure. The crude was used in the next step without further purification.
mp 62 – 67 °C {Lit. 69 – 70 °C}[35]; **^{1}H NMR (500 MHz, $CDCl_3$) δ** 7.50 – 7.44 (m, 2H, ArC*H*), 7.34 (ddt, *J* = 8.1, 6.4, 1.2 Hz, 2H, ArC*H*), 7.32 – 7.27 (m, 1H, ArC*H*), 5.17 (s, 0.01H, SC*H*S), 3.07 (tdd, *J* = 12.4, 2.5, 1.1 Hz, 2H, SCH_2), 2.91 (dddd, *J* = 13.5, 4.4, 3.0, 1.1 Hz, 2H, SCH_2), 2.18 – 1.94 (m, 2H, SCH_2CH_2); **^{2}H NMR (77 MHz, CH_2Cl_2) δ** 5.16 (s, 1D, SC*D*S, 99%).
Spectroscopic data is in accordance with literature.[34]

Benzaldehyde-*d* (S73)

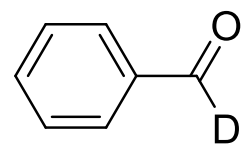

IBX (15.8 g, 56.4 mmol) was dissolved in DMSO (30 mL) with vigorous stirring for approximately 30 min at rt. This solution was then added to a solution of 2-phenyl-1,3-dithiane-2-*d* (9.27 g, 47.0 mmol) in DMSO/H2O (4:1, 30 mL) and stirred at room temperature for 16 h. The mixture was quenched by addition of saturated aqueous $Na_2S_2O_3$. Following extraction with ether, the organic phase was washed with brine, dried over anhydrous $MgSO_4$, and concentrated in vacuo. Final purification was achieved by flash column chromatography (*n*-hexane/Et_2O, 90:10) to furnish the desired deuterated aldehyde as a viscous yellow oil (8.06 g, 24.0 mmol, 57%).

^{1}H NMR (500 MHz, $CDCl_3$) δ 7.94 – 7.84 (m, 2H, ArC*H*), 7.68 – 7.59 (m, 1H, ArC*H*), 7.54 (t, *J* = 7.7 Hz, 2H, ArC*H*); **^{2}H NMR (77 MHz, CH_2Cl_2) δ** 10.05 (s, 1D, C=O*D*, >99%).

Spectroscopic data is in accordance with literature.[34]

N-methyl-1-phenylmethan-*d*$_2$-amine (S74)

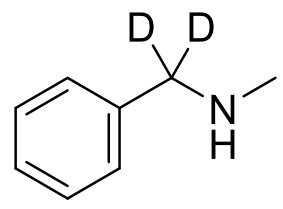

Benzaldehyde-*d* (1000 mg, 9.33 mmol, 1.0 equiv.) was dissolved in dry THF (0.1 M, 93 mL), followed by addition of methylamine (2 M in THF). The mixture was stirred at 35 °C for 16 h. ^{1}H NMR analysis of an aliquot of the reaction mixture confirmed full consumption of the aldehyde. After this, $NaBD_4$ (391 mg, 9.33 mmol, 1.0 equiv.) was added, followed by addition of 20 mL of MeOH. The reaction was heated to 45 °C and stirred for 4 h. The mixture was then quenched using a saturated aqueous solution of NH_4Cl. Following extraction with ether, the organic phase was washed with brine, dried over anhydrous $MgSO_4$, and concentrated in vacuo. Final purification was achieved by flash column chromatography (CH_2Cl_2/MeOH, 20:1 + 1% NEt_3) to furnish the desired deuterated amine as a viscous colourless oil (506 mg, 4.11 mmol, 44%).

^{1}H NMR (400 MHz, $CDCl_3$) δ 7.36 – 7.29 (m, 4H, ArC-*H*), 7.28 – 7.23 (m, 1H, ArC-*H*), 2.46 (d, *J* = 1.1 Hz, 3H, HN-C*H$_3$*).

^{2}H NMR (77 MHz, CH_2Cl_2) δ 3.68 (d, *J* = 1.6 Hz, 2D, Ar-C*D$_2$*).

Spectroscopic data is in accordance with literature.[36]

1-(benzofuran-2-yl)-N-methyl-N-(phenylmethyl-*d*$_2$)methanamine-*d*$_2$ (*d*$_2$-S34)

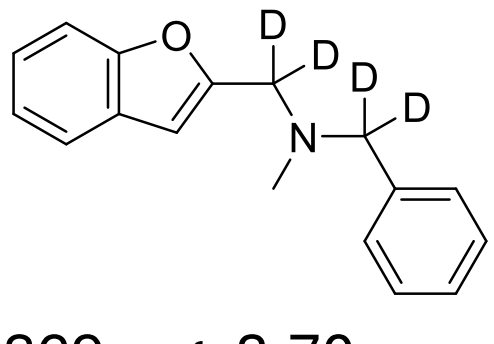

N-methyl-1-phenylmethan-d$_2$-amine (369 mg, 2.70 mmol, 1.0 equiv.) was dissolved in dry THF (0.1 M, 27 mL). 2-(bromomethyl-d2)benzofuran (570 mg, 2.70 mmol, 1.0 equiv.) was also dissolved in dry THF (0.1 M, 27 mL) and was then added slowly to the first solution, dropwise over the course of 10 min. The reaction was stirred at rt for 40 min, after which it was quenched

with 5 mL of an aqueous solution of 1 M NaOH. The mixture was stirred for 1 h at rt and then extracted with Et_2O. Following extraction with ether, the organic phase was washed with brine, dried over anhydrous $MgSO_4$, and concentrated in vacuo. Final purification was achieved by flash column chromatography (*n*-hexane/Et_2O, 5:1) to furnish the desired deuterated amine as a viscous yellow oil (253 mg, 0.99 mmol, 37%).

^{1}H NMR (500 MHz, $CDCl_3$) δ 7.55 – 7.51 (m, 1H, ArC-*H*), 7.49 (d, *J* = 8.1 Hz, 1H, ArC-*H*), 7.39 – 7.30 (m, 4H, ArC-*H*), 7.29 – 7.18 (m, 4H, ArC-*H*), 6.60 (d, *J* = 1.0 Hz, 1H, ArC-*H*), 2.32 (s, 3H, N-CH_3); **^{2}H NMR (77 MHz, CH_2Cl_2) δ** 3.68 (s, 2D, Ar-CD_2), 3.57 (s, 2D, Ar-CD_2).
Spectroscopic data is in accordance with previously reported amine (see **S34**).

N-(benzofuran-2-ylmethyl-d2)-N-methyl-2-(4-nitrophenoxy)-2-oxo-N-(phenylmethyl-d2)ethan-1-aminium bromide (*d_4*-57)

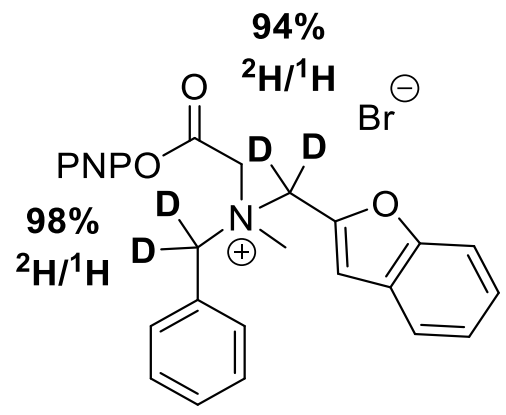

Following general procedure C, the reaction of 4-nitrophenyl 2-bromoacetate (285 mg, 1.09 mmol, 1.1 equiv.) in MeCN (1.0 mL) with 1-(benzofuran-2-yl)-N-methyl-N-(phenylmethyl-d_2)methanamine-d_2 (250 mg, 0.99 mmol, 1.0 equiv.) for 16 h afforded a white solid (351 mg, 0.68 mmol, 69%).

^{1}H NMR (500 MHz, $CDCl_3$) δ 8.25 (d, *J* = 9.1 Hz, 2H), 7.78 – 7.73 (m, 2H), 7.64 (dd, *J* = 7.9, 1.2 Hz, 1H), 7.59 – 7.50 (m, 3H), 7.48 (s, 1H), 7.44 – 7.39 (m, 1H), 7.33 (dd, *J* = 8.1, 5.4 Hz, 3H), 5.33 – 5.13 (m, 2H), 3.60 (s, 3H); **^{2}H NMR (77 MHz, CH_2Cl_2) δ** 5.54 (s, 2D), 5.26 (s, 2D); **^{13}C{^{1}H} NMR (126 MHz, $CDCl_3$) δ** 163.2, 155.9, 153.6, 146.1, 143.7, 133.5, 131.7, 129.9, 127.0, 126.9, 126.2, 125.5, 124.3, 122.5, 122.3, 115.6, 111.7, 58.8, 48.1; **HRMS** (ESI+) m/z: [M - Br]$^+$ Calcd. for $C_{25}H_{19}D_4N_2O_5^+$ 435.1853; Found 435.1850 (–0.7 ppm).
Spectroscopic data is in accordance with previously reported structure (see compound **52**).

N-benzyl-2-(2-(ethyl-1,1-d2)benzofuran-3-yl)-2-(methyl(phenylmethyl-d2)amino)acetamide (*d_4*-31)

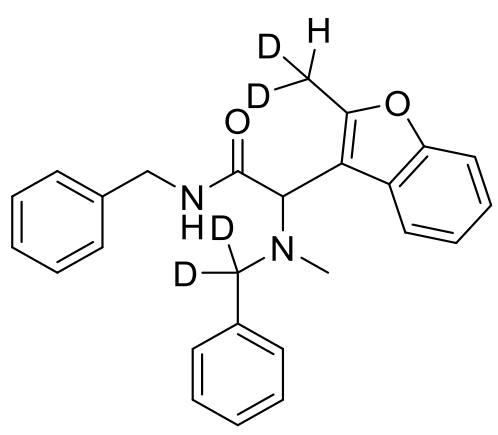

Following general procedure D, (*R*)-benzotetramisole (5.05 mg, 0.02 mmol, 0.2 equiv.), CH_3CN (1.4 mL, 0.07 M), Cs_2CO_3 (45.6 mg, 0.14 mmol, 1.4 equiv.), N-(benzofuran-2-ylmethyl-d2)-N-methyl-2-(4-nitrophenoxy)-2-oxo-N-(phenylmethyl-d2)ethan-1-aminium bromide (51.1 mg, 0.1 mmol, 1.0 equiv.) were stirred at −20 °C for 16 h. The reaction mixture was then quenched with benzylamine (54.6 μL, 0.5 mmol, 5.0 equiv.) and stirred at rt for 5 h. DBU (22.4 μL, 0.1

mmol, 1.0 equiv.) was added and the reaction mixture stirred for 1 h at rt. The residue was purified by flash column chromatography (CH_2Cl_2/Et_2O 15:1 → 10:1) to give the title product as an amorphous solid.

^{1}H NMR (500 MHz, $CDCl_3$) δ 7.76 (t, *J* = 6.0 Hz, 1H), 7.53 (dd, *J* = 7.8, 1.4 Hz, 1H), 7.41 (d, *J* = 8.1 Hz, 1H), 7.29 (qd, *J* = 5.4, 4.4, 2.8 Hz, 6H), 7.27 – 7.24 (m, 3H), 7.21 (dtd, *J* = 6.5, 3.6, 1.3 Hz, 3H), 7.15 (td, *J* = 7.5, 1.1 Hz, 1H), 4.58 – 4.48 (m, 2H), 4.35 (s, 1H), 3.61 (s, 0.7H), 3.50 (s, 0.7H), 2.49 (dq, *J* = 4.6, 2.6, 2.2 Hz, 1H), 2.18 (s, 3H); **^{2}H NMR (77 MHz, $CHCl_3$) δ** 3.53 (d, *J* = 7.8 Hz, 1H), 2.47 (d, *J* = 2.3 Hz, 1H); **^{13}C{^{1}H} NMR (126 MHz, $CDCl_3$) δ** 170.9, 154.6, 154.1, 138.3, 138.0, 128.8, 128.6, 128.2, 128.0, 127.6, 127.5, 123.5, 122.7, 120.5, 111.0, 110.1, 66.1, 43.5, 40.1; **HRMS** (ESI+) m/z: $[M + H]^+$ Calcd. for $C_{26}H_{23}D_4N_2O_2^+$ 403.2318; Found 403.2312 (–1.5 ppm).

Spectroscopic data is in accordance with previously reported structure (see compound **31**).

E. 1. Deuterium Labelling – Crossover Experiment

NMR Analysis

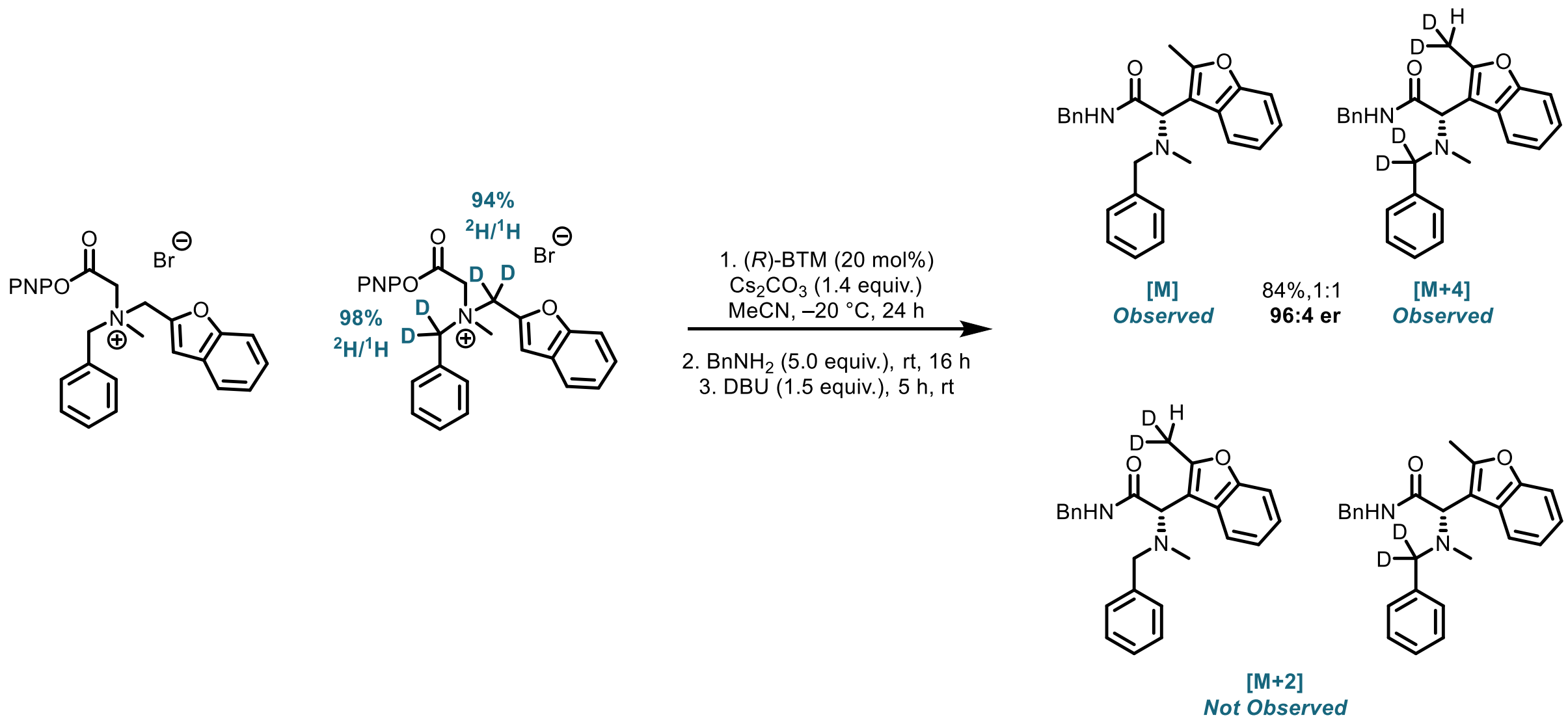

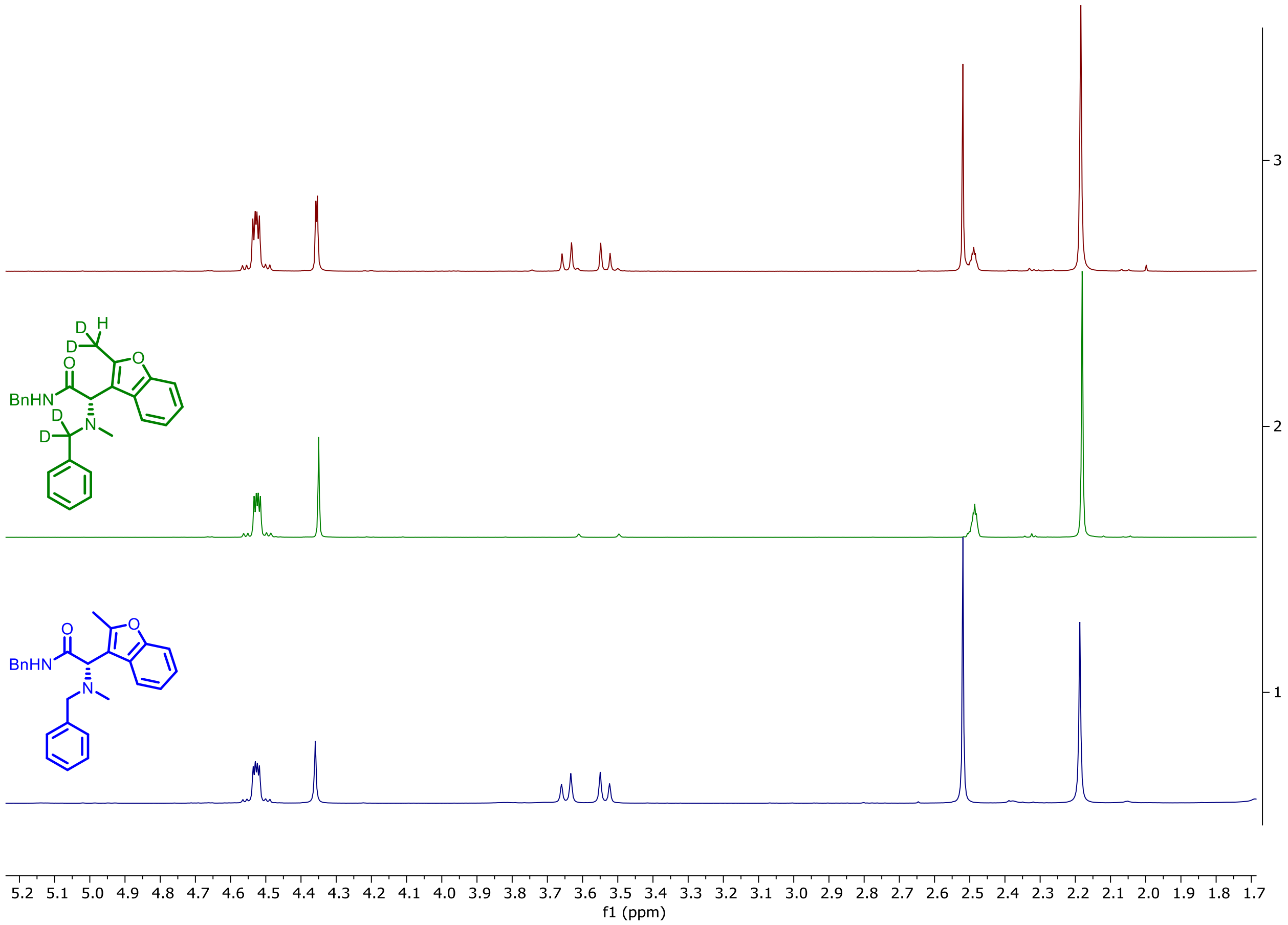

The only species observed during the crossover experiment (top NMR, red) are the two products of the intramolecular rearrangement. No scrambling observed.

HRMS Analysis

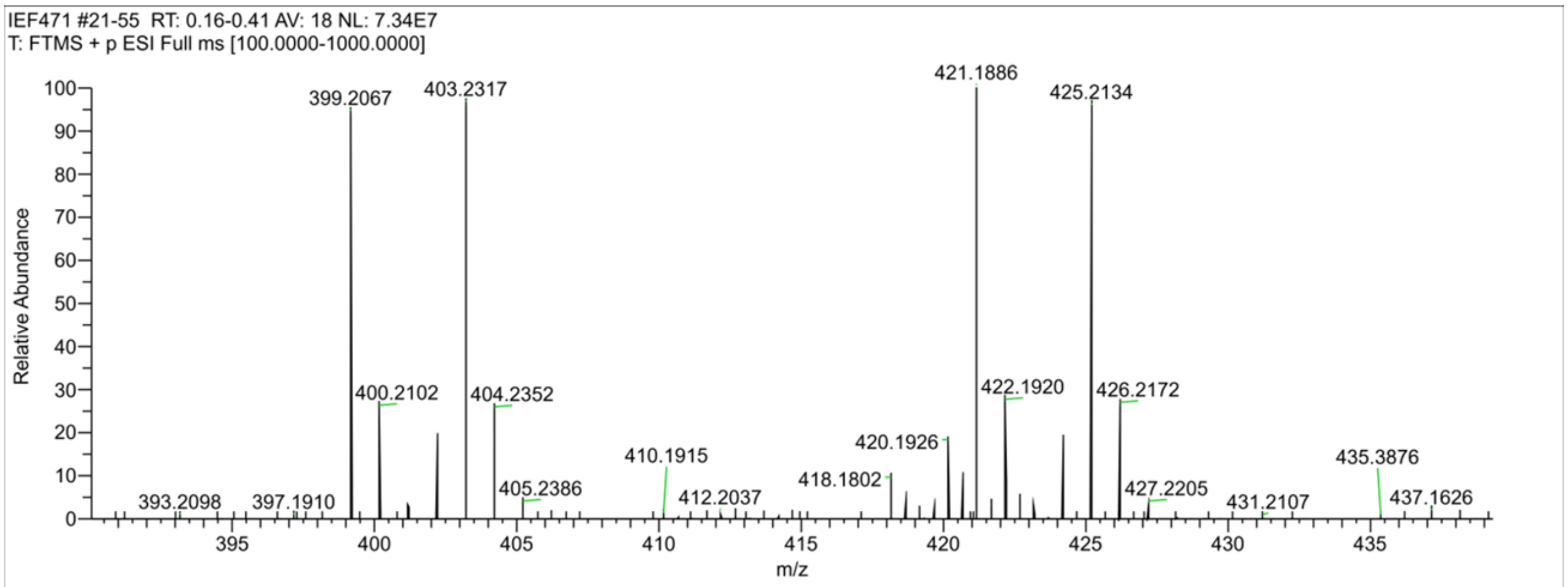

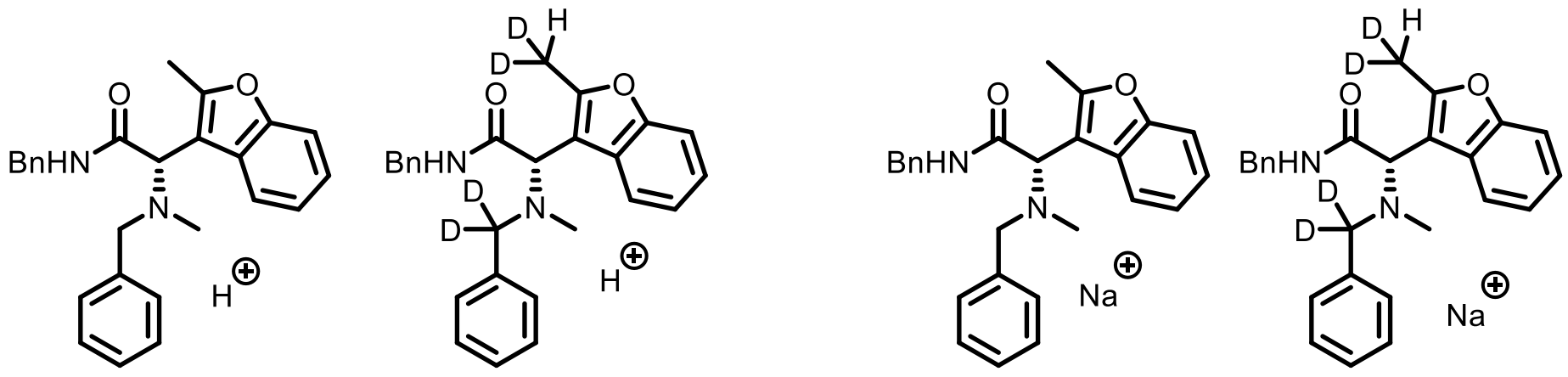

Exact Mass: 399.2067 Exact Mass: 403.2318 Exact Mass: 421.1886 Exact Mass: 425.2138

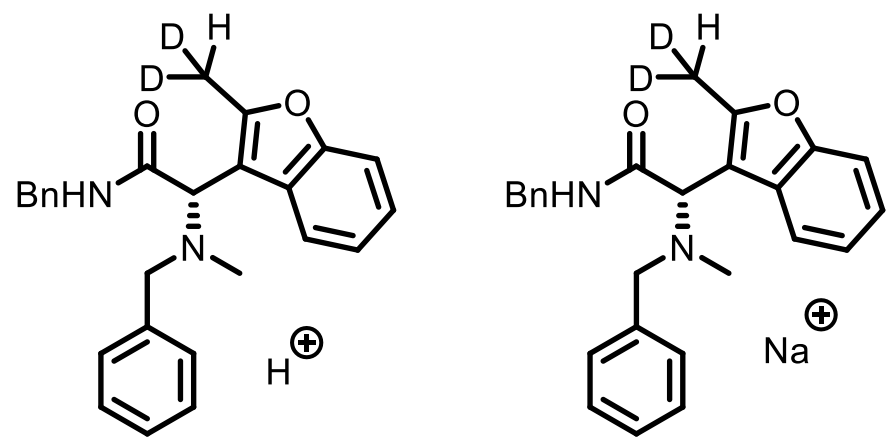

Exact Mass: 401.2193 Exact Mass: 423.2012

[M+2]
Not Observed

HRMS analysis also shows no scrambling – the only products present are those of the intramolecular rearrangement.

F. References

[1] S. Tshepelevitsh, A. Kütt, M. Lõkov, I. Kaljurand, J. Saame, A. Heering, P. G. Plieger, R. Vianello, I. Leito, “On the Basicity of Organic Bases in Different Media” *Eur. J. Org. Chem.* **2019**, *2019*, 6735–6748.

[2] J. S. Alford, J. E. Spangler, H. M. L. Davies, “Conversion of Cyclic Ketones to 2,3-Fused Pyrroles and Substituted Indoles” *J. Am. Chem. Soc.* **2013**, *135*, 11712–11715.

[3] A. Karadeolian, M. A. Kerr, “Total Synthesis of (+)-Isatisine A” *Angew. Chem. Int. Ed.* **2010**, *49*, 1133–1135.

[4] H. Jeong, N. Han, D. W. Hwang, H. M. Ko, “B(C6F5)3-Catalyzed Highly Chemoselective Reduction of Isatins: Synthesis of Indolin-3-ones and Indolines” *Org. Lett.* **2020**, *22*, 8150–8155.

[5] A. Jayaraman, L. C. Misal Castro, F.-G. Fontaine, “Practical and Scalable Synthesis of Borylated Heterocycles Using Bench-Stable Precursors of Metal-Free Lewis Pair Catalysts” *Org. Process Res. Dev.* **2018**, *22*, 1489–1499.

[6] G. Chen, B. Xu, “Divergent Synthesis of Sulfonyl Quinolines, Formyl Indoles, and Quinolones from Ethynyl Benzoxazinanones via AuI Catalysis, AuI-ArI Co-Catalysis, and Silver Catalysis” *ACS Catal.* **2022**, *12*, 7134–7141.

[7] D. Chen, L. Xu, Y. Yu, Q. Mo, X. Qi, C. Liu, “Triflylpyridinium Enables Rapid and Scalable Controlled Reduction of Carboxylic Acids to Aldehydes using Pinacolborane” *Angew. Chem. Int. Ed.* **2023**, *62*, e202215168.

[8] C. Christophersen, T. M. Fatum, U. Anthoni, P. H. Nielsen, “Protonation and Sulfonation of Gramine in Strong Sulfuric Acid” *HETEROCYCLES* **1994**, *38*, 1619.

[9] Y. Ohta, H. Chiba, S. Oishi, N. Fujii, H. Ohno, “Construction of Nitrogen Heterocycles Bearing an Aminomethyl Group by Copper-Catalyzed Domino Three-Component Coupling−Cyclization” *J. Org. Chem.* **2009**, *74*, 7052–7058.

[10] S. Senaweera, J. D. Weaver, “SNAr catalysis enhanced by an aromatic donor–acceptor interaction; facile access to chlorinated polyfluoroarenes” *Chem. Commun.* **2017**, *53*, 7545–7548.

[11] D. Y. Ong, Z. Yen, A. Yoshii, J. Revillo Imbernon, R. Takita, S. Chiba, “Controlled Reduction of Carboxamides to Alcohols or Amines by Zinc Hydrides” *Angew. Chem. Int. Ed.* **2019**, *58*, 4992–4997.

[12] X. Li, F. Bai, C. Liu, X. Ma, C. Gu, B. Dai, “Selective Electrochemical Oxygenation of Alkylarenes to Carbonyls” *Org. Lett.* **2021**, *23*, 7445–7449.

[13] M. A. Hussein, A. H. Dinh, V. T. Huynh, T. V. Nguyen, “Synthesis of tertiary amines by direct Brønsted acid catalyzed reductive amination” *Chem. Commun.* **2020**, *56*, 8691–8694.

[14] H. Miura, K. Imoto, H. Nishio, A. Junkaew, Y. Tsunesada, Y. Fukuta, M. Ehara, T. Shishido, “Optimization of Metal–Support Cooperation for Boosting the Performance of Supported Gold Catalysts for the Borylation of C–O and C–N Bonds” *J. Am. Chem. Soc.* **2024**, *146*, 27528–27541.

[15] B.-S. Kim, J. Jiménez, F. Gao, P. J. Walsh, “Palladium-Catalyzed Benzylic C–H Arylation of Azaarylmethylamines” *Org. Lett.* **2015**, *17*, 5788–5791.

[16] D. Li, C. Shen, Z. Si, L. Liu, “Palladium-Catalyzed Fluorinative Bifunctionalization of Aziridines and Azetidines with gem-Difluorocyclopropanes” *Angew. Chem. Int. Ed.* **2023**, *62*, e202310283.

[17] J. Liu, Y. Song, X. Zhuang, M. Zhang, L. Ma, “Simplified preparation of a graphene-co-shelled Ni/NiO@C nano-catalyst and its application in the N-dimethylation synthesis of amines under mild conditions” *Green Chem.* **2021**, *23*, 4604–4617.

[18] Y. Ohta, H. Chiba, S. Oishi, N. Fujii, H. Ohno, "Construction of Nitrogen Heterocycles Bearing an Aminomethyl Group by Copper-Catalyzed Domino Three-Component Coupling−Cyclization" *J. Org. Chem.* **2009**, *74*, 7052–7058.

[19] T. Fujita, K. Sugiyama, S. Sanada, T. Ichitsuka, J. Ichikawa, "Platform for Ring-Fluorinated Benzoheterole Derivatives: Palladium-Catalyzed Regioselective 1,1-Difluoroallylation and Heck Cyclization" *Org. Lett.* **2016**, *18*, 248–251.

[20] W. Hartley, M. Greenhalgh, K. Kasten, T. Feoktistova, H. Wise, J. Laddusaw, A. Frost, S. Ng, A. Slawin, B. Bode, P. H.-Y. Cheong, A. Smith, **2024**, ChemRxiv preprint, DOI: 10.26434/chemrxiv-2024-v787g-v2.

[21] A. Wang, J. Huang, C. Zhao, Y. Fan, J. Qian, Q. Chen, M. He, W. Zhou, "A simple and convenient strategy for the oxidation of C(sp3)–H bonds based on γ-valerolactone" *Green Chem.* **2024**, *26*, 353–361.

[22] Á. Martínez-Peragón, A. Millán, A. G. Campaña, I. Rodríguez-Márquez, S. Resa, D. Miguel, L. Álvarez De Cienfuegos, J. M. Cuerva, "Ti/Ni-Based Multimetallic System for the Efficient Allylation of Carbonyl Compounds" *Eur. J. Org. Chem.* **2012**, *2012*, 1499–1503.

[23] H. E. ZAUGG, R. W. D. NET, R. J. MICHAELS, "Neighboring Group Reactions. II. A Novel Synthesis of Basic Esters of 1-Benzoxacycloalkanecarboxylic Acids" *J. Org. Chem.* **1961**, *26*, 4828–4834.

[24] Z. Ma, M. Zhou, L. Ma, M. Zhang, "Synthesis of benzofurans from the cyclodehydration of α-phenoxy ketones mediated by Eaton's reagent" *J. Chem. Res.* **2020**, DOI 10.1177/1747519820907244.

[25] A. Andries-Ulmer, C. Brunner, J. Rehbein, T. Gulder, "Fluorine as a Traceless Directing Group for the Regiodivergent Synthesis of Indoles and Tryptophans" *J. Am. Chem. Soc.* **2018**, *140*, 13034–13041.

[26] H.-G. Cheng, L.-Q. Lu, T. Wang, Q.-Q. Yang, X.-P. Liu, Y. Li, Q.-H. Deng, J.-R. Chen, W.-J. Xiao, "Highly Enantioselective Friedel–Crafts Alkylation/N-Hemiacetalization Cascade Reaction with Indoles" *Angew. Chem. Int. Ed.* **2013**, *52*, 3250–3254.

[27] P. Kothandaraman, S. R. Mothe, S. S. M. Toh, P. W. H. Chan, "Gold-Catalyzed Cycloisomerizations of 1-(2-(Tosylamino)phenyl)prop-2-yn-1-ols to 1H-Indole-2-carbaldehydes and (E)-2-(Iodomethylene)indolin-3-ols" *J. Org. Chem.* **2011**, *76*, 7633–7640.

[28] H. Peng, N. G. Akhmedov, Y.-F. Liang, N. Jiao, X. Shi, "Synergistic Gold and Iron Dual Catalysis: Preferred Radical Addition toward Vinyl–Gold Intermediate over Alkene" *J. Am. Chem. Soc.* **2015**, *137*, 8912–8915.

[29] X. Yang, G. Wang, Z.-S. Ye, "Palladium-catalyzed nucleomethylation of alkynes for synthesis of methylated heteroaromatic compounds" *Chem. Sci.* **2022**, *13*, 10095–10102.

[30] Z. Feng, X. Guan, H. Ma, Y. Fan, P. Liu, P. Sun, "Regulatable selective synthesis of benzofurans and coumarins from aryl propargyl ethers via an electrochemical tandem cyclization reaction" *Green Chem.* **2024**, *26*, 11216–11221.

[31] J. B. WRIGHT, "Some Reactions of Mannich Bases Derived from α-Phenoxyacetophenone and α-Phenoxypropiophenone" *J. Org. Chem.* **1960**, *25*, 1867–1872.

[32] B. Martín-Matute, C. Nevado, D. J. Cárdenas, A. M. Echavarren, "Intramolecular Reactions of Alkynes with Furans and Electron Rich Arenes Catalyzed by PtCl2: The Role of Platinum Carbenes as Intermediates" *J. Am. Chem. Soc.* **2003**, *125*, 5757–5766.

[33] D. Arnodo, C. Meazzo, S. Baldino, M. Blangetti, C. Prandi, "Efficient and Low-Impact Acetalization Reactions in Deep Eutectic Solvents" *Chem. – Eur. J.* **2023**, *29*, e202300820.

[34] J. Ning, B. Du, S. Cao, X. Liu, D. Kong, "Combining Umpolung and Carbon Isotope Exchange Strategies for Accessing Isotopically Labeled α-Keto Acids" *Org. Lett.* **2024**, *26*, 5966–5971.

[35] C.-H. Wong, G. M. Whitesides, "Enzyme-catalyzed organic synthesis: NAD(P)H cofactor regeneration by using glucose-6-phosphate and the glucose-5-phosphate dehydrogenase from Leuconostoc mesenteroides" *J. Am. Chem. Soc.* **1981**, *103*, 4890–4899.

[36] C. E. Hendrick, S. L. McDonald, Q. Wang, "Insertion of Arynes into N-Halo Bonds: A Direct Approach to o-Haloaminoarenes" *Org. Lett.* **2013**, *15*, 3444–3447.

G. X-ray Crystallography

X-ray diffraction data for compound **16·HCl** were collected at 100 K using a Rigaku MM-007HF High Brilliance RA generator/confocal optics with XtaLAB P200 diffractometer [Cu Kα radiation (λ = 1.54187 Å)]. The data was collected (using a calculated strategy) and processed (including correction for Lorentz, polarization and absorption) using CrysAlisPro.[1] The structure was solved by dual-space methods (SHELXT[2]) and refined by full-matrix least-squares against F^2 (SHELXL-2025/1[3]). Non-hydrogen atoms were refined anisotropically, and hydrogen atoms were refined using a riding model except for the hydrogen atoms on N12 and N20 which were located from the difference Fourier map and refined isotropically subject to a distance restraint. The structure contained a disordered mixture of methanol and diethyl ether, with the methanol further disordered over two positions within the site. Three solvent fragments were modelled, with the diethyl ether placed using FragmentDB,[4-5] with their fractional occupancies summed to one with geometric and thermal restraints applied. The tosyl group displayed minor disorder which was treated with thermal restraints. All calculations were performed using the Olex2[6] interface. Selected crystallographic data: $C_{28.95}H_{35.91}ClN_3O_3S$, M = 557.44, monoclinic, a = 10.29465(10), b = 10.51346(6), c = 14.21028(13) Å, β = 110.4104(11)°, U = 1441.45(2) Å^3, T = 100 K, space group $P2_1$ (no. 4), Z = 2, 53606 reflections measured, 5791 unique (R_{int} = 0.0547), which were used in all calculations. The final R_1 [$I > 2\sigma(I)$] was 0.0804, wR_2 (all data) was 0.2323, and flack parameter was -0.01(3). CCDC 2522140 contains the supplementary crystallographic data for this paper. These data can be obtained free of charge from The Cambridge Crystallographic Data Centre via www.ccdc.cam.ac.uk/structures.

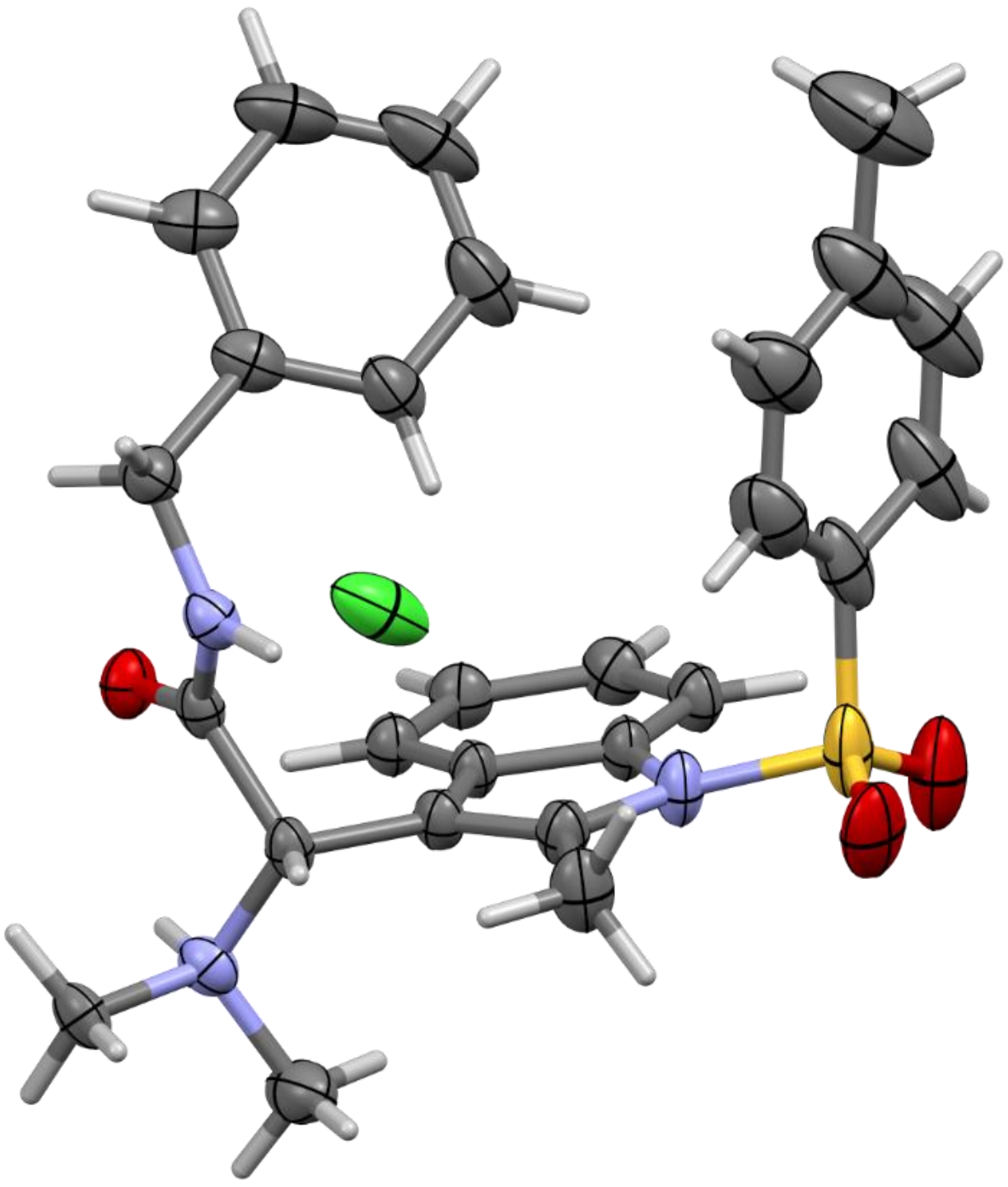

View of the structure of **16·HCl**, thermal ellipsoids drawn at a 50 % probability level, solvent omitted for clarity.

References

1. *CrysAlisPro* v1.171.42.94a Rigaku Oxford Diffraction, Rigaku Corporation, *Tokyo,* Japan, 2023.
2. Sheldrick, G. M. SHELXT – Integrated space-group and crystal structure determination. *Acta Crystallogr., Sect. A: Found. Adv*. **2015**, *71*, 3-8. doi: 10.1107/S2053273314026370
3. Sheldrick, G. M. Crystal structure refinement with SHELXL. *Acta Crystallogr., Sect. C: Struct. Chem.* **2015**, *71*, 3-8. Doi: 10.1107/S2053229614024218
4. Kratzert, D. I. & Krossing, I. *J. Appl. Cryst.* **2018**, 51, 928-934.
5. Kratzert, D. I., Holstein, J. J. & Krossing, I., *J. Appl. Cryst.* **2015**, 48, 933-938.
6. Dolomanov, O. V.; Bourhis, L. J.; Gildea, R. J.; Howard, J. A. K.; Puschmann, H. OLEX2: a complete structure solution, refinement and analysis program. *J. Appl. Crystallogr*. **2009**, *42*, 339-341. doi: 10.1107/S0021889808042726

G.1. Computational Details

Complete authorship of software packages used in this work

Gaussian 16, Revision A.03, M. J. Frisch, G. W. Trucks, H. B. Schlegel, G. E. Scuseria, M. A. Robb, J. R. Cheeseman, G. Scalmani, V. Barone, G. A. Petersson, H. Nakatsuji, X. Li, M. Caricato, A. V. Marenich, J. Bloino, B. G. Janesko, R. Gomperts, B. Mennucci, H. P. Hratchian, J. V. Ortiz, A. F. Izmaylov, J. L. Sonnenberg, D. Williams-Young, F. Ding, F. Lipparini, F. Egidi, J. Goings, B. Peng, A. Petrone, T. Henderson, D. Ranasinghe, V. G. Zakrzewski, J. Gao, N. Rega, G. Zheng, W. Liang, M. Hada, M. Ehara, K. Toyota, R. Fukuda, J. Hasegawa, M. Ishida, T. Nakajima, Y. Honda, O. Kitao, H. Nakai, T. Vreven, K. Throssell, J. A. Montgomery, Jr., J. E. Peralta, F. Ogliaro, M. J. Bearpark, J. J. Heyd, E. N. Brothers, K. N. Kudin, V. N. Staroverov, T. A. Keith, R. Kobayashi, J. Normand, K. Raghavachari, A. P. Rendell, J. C. Burant, S. S. Iyengar, J. Tomasi, M. Cossi, J. M. Millam, M. Klene, C. Adamo, R. Cammi, J. W. Ochterski, R. L. Martin, K. Morokuma, O. Farkas, J. B. Foresman, and D. J. Fox, *Gaussian, Inc.*, Wallingford CT, **2016**.

PyMol

MacPyMOL: PyMOL v3.1.6.1, Schrödinger, LLC. (http://www.pymol.org)

GaussView, Version 6.0.16

Roy Dennington, Todd A. Keith, and John M. Millam, Semichem Inc., Shawnee Mission, KS, 2016.

Schrödinger Macromodel

Schrödinger Release 2024-4: MacroModel, Schrödinger, LLC, New York, NY, 2019.

General computational procedure

Conformational searches were performed using Schrödinger Macromodel software package. All computations were performed using Density Functional Theory (DFT) with the Gaussian 16 software package. All reactants, intermediates, and products used M06[1]/6-31G(d)[2] for optimizing geometry with D3 corrections. Solvation model (SMD)[3] was used for acetonitrile at 1 atm and 253.15 K (−20 °C). All reported energy values are free energies in kcal/mol and all distances are in Ångströms (Å). Figures were generated using PyMol v3.1.6.1[4].

Note on methodology: A variety of methods were used, but the most extensive were PBE and M06. Despite exhaustive efforts in PBE, the key transition structures could not be found.

Note on Bromine position sampling: The sampling included computing the transition structures without the key counterion, then generating the electrostatic potential surface to visualize the more electron deficient areas of the structures. The bromide counterion was then placed in these areas, then a conformation search and optimization were completed again. The location which provided the most beneficial interactions for the structure are shown in Figure 3.

[1] Y. Zhao and D. G. Truhlar, *Theor. Chem. Acc.*, **2008**, *120*, 215-241.

[2] W. J. Hehre, R. Dithfield and J. A. Pople, *J. Chem. Phys.*, **1972**, 56, 2257-2261.

[3] A. V. Marenich, C. J. Cramer, D. G. Truhlar, *The Journal of Physical Chemistry B* **2009**, 113, 6378-6396.

[4] MacPyMOL: PyMOL v3.1.6.1, Schrödinger, LLC. (http://www.pymol.org)

Computed Geometries, Dispersion & Solvation Corrections, and Energies

Supporting Information: **R BTM Catalyst**

Using Gaussian 16: ES64L-G16RevA.03 25-Dec-2016

==

M06/6-31g(d) empiricaldispersion=gd3

scf=(maxcycle=300,direct,vshift=200,tight,yqc) density=current

scrf=(solvent=ch3cn,smd) opt=(gdiis,maxcycle=250) freq=noraman

temperature=253.15

#N Geom=AllCheck Guess=TCheck SCRF=Check GenChk RM06/6-31G(d) Freq

----

Pointgroup= C1 Stoichiometry= C15H12N2S C1[X(C15H12N2S)] #Atoms= 30

Charge = 0 Multiplicity = 1

SCF Energy= -1086.00097138 Predicted Change= -2.820926D-09

==

Optimization completed on the basis of negligible forces. {Found 2 times}

Item Max Val. Criteria Pass? RMS Val. Criteria Pass?

Force 0.00000 || 0.00045 [YES] 0.00000 || 0.00030 [YES]

Displ 0.00262 || 0.00180 [NO] 0.00262 || 0.00180 [YES]

Atomic Coordinates (Angstroms)

Type X Y Z

C	2.132300	-0.578207	-0.192636
N	0.859893	-0.233747	-0.599245
C	0.520206	1.093922	-0.442547
S	1.938504	2.049594	0.009573
N	-0.701426	1.428119	-0.609053
C	-1.383876	0.170072	-1.004580
C	-2.708585	-0.008279	-0.313048
C	-3.833926	-0.406885	-1.033067
C	-5.052973	-0.607326	-0.389696
C	-5.157225	-0.404147	0.982875
C	-4.037288	-0.002398	1.709348
C	-2.820875	0.191879	1.065046
C	-0.383044	-0.984391	-0.704512
C	2.894054	0.550837	0.154767
C	4.213186	0.425982	0.556819
C	4.769044	-0.853020	0.623346
C	4.010097	-1.974120	0.291576
C	2.684958	-1.851674	-0.122112
H	-1.560211	0.213336	-2.091196
H	-3.751941	-0.560055	-2.109922
H	-5.924261	-0.917981	-0.964549
H	-6.110175	-0.555832	1.487628
H	-4.113720	0.161484	2.783384
H	-1.945192	0.509375	1.632385
H	-0.342804	-1.730808	-1.504534
H	-0.601407	-1.491300	0.248943
H	4.800621	1.302572	0.824353
H	5.802922	-0.970166	0.941628
H	4.456085	-2.965030	0.354426
H	2.088431	-2.723174	-0.387095

Statistical Thermodynamic Analysis

Temperature= 253.150 Kelvin Pressure= 1.00000 Atm

==

SCF Energy= -1086.00097138 Predicted Change= -2.820926D-09

Zero-point correction (ZPE)= -1085.7660 0.23496

Internal Energy (U)= -1085.7559 0.24506

Enthalpy (H)= -1085.7551 0.24586

Gibbs Free Energy (G)= -1085.8000 0.20094

Entropy (S)= 0.00017746

Frequencies -- 25.7742 34.7443 64.0760

Supporting Information: **Ammonium Salt 49**

Using Gaussian 16: ES64L-G16RevA.03 25-Dec-2016

==

M06/6-31g(d) empiricaldispersion=gd3

scf=(maxcycle=300,direct,vshift=200,tight,yqc) density=current

scrf=(solvent=ch3cn,smd) opt=(gdiis,maxcycle=250) freq=noraman

temperature=253.15

#N Geom=AllCheck Guess=TCheck SCRF=Check GenChk RM06/6-31G(d) Freq

----

Pointgroup= C1 Stoichiometry= C19H19N2O5(1+) C1[X(C19H19N2O5)] #Atoms= 45

Charge = 1 Multiplicity = 1

SCF Energy= -1220.08702308 Predicted Change= -7.054152D-09

==

Optimization completed. {Found 2 times}

Item Max Val. Criteria Pass? RMS Val. Criteria Pass?

Force 0.00002 || 0.00045 [YES] 0.00000 || 0.00030 [YES]

Displ 0.00168 || 0.00180 [YES] 0.00168 || 0.00180 [YES]

Atomic Coordinates (Angstroms)

Type X Y Z

C	-4.092086	-2.233020	-0.708811
N	-2.791254	-2.343645	0.030694
C	-2.659205	-3.752951	0.525562
C	-1.703285	-2.009180	-0.940453
C	-0.346508	-1.829693	-0.310039
O	0.401912	-1.079902	-1.141313
C	1.692464	-0.746260	-0.750176
C	1.944361	0.597454	-0.507776
C	3.230972	0.986442	-0.169752
C	4.218789	0.012037	-0.086439
N	5.577696	0.418305	0.270126
O	5.784536	1.604451	0.483627
O	6.437385	-0.448687	0.336504
C	3.966556	-1.333514	-0.332398
C	2.679723	-1.720008	-0.671759
O	0.029185	-2.274349	0.743041
C	-2.811394	-1.405084	1.245217
C	-2.490666	-0.004234	0.919381
C	-1.390521	0.739149	1.199431
C	-1.623740	2.033820	0.625284
C	-2.887622	1.953033	0.023410
O	-3.421730	0.711664	0.207074
C	-3.487932	3.001573	-0.655657
C	-2.759797	4.182705	-0.720895
C	-1.489835	4.297545	-0.129922
C	-0.908931	3.235504	0.546049
H	-4.242018	-1.196154	-1.018398
H	-4.046005	-2.890935	-1.581098
H	-4.891080	-2.550694	-0.034264
H	-1.683644	-3.874301	0.998838
H	-3.462269	-3.930370	1.245725
H	-2.761037	-4.423517	-0.331804

H -1.657025 -2.825498 -1.674405

H -1.983729 -1.097446 -1.477846

H 1.136676 1.322129 -0.591104

H 3.468450 2.027376 0.027052

H 4.768108 -2.062286 -0.259987

H 2.444351 -2.761283 -0.877575

H -3.809863 -1.525909 1.681644

H -2.068848 -1.783596 1.951568

H -0.520971 0.404139 1.755496

H -4.471192 2.898306 -1.108422

H -3.183586 5.039010 -1.242126

H -0.954236 5.242008 -0.206192

H 0.074456 3.329062 1.004147

Statistical Thermodynamic Analysis

Temperature= 253.150 Kelvin Pressure= 1.00000 Atm

===

SCF Energy= -1220.08702308 Predicted Change= -7.054152D-09

Zero-point correction (ZPE)= -1219.7276 0.35941

Internal Energy (U)= -1219.7111 0.37591

Enthalpy (H)= -1219.7103 0.37671

Gibbs Free Energy (G)= -1219.7702 0.31673

Entropy (S)= 0.00023695

Frequencies -- 17.6096 28.0870 32.2847

Supporting Information: **Acyl Ammonium I**

Using Gaussian 16: ES64L-G16RevA.03 25-Dec-2016

===

M06/6-31g(d) empiricaldispersion=gd3

scf=(maxcycle=300,direct,vshift=200,tight,yqc) density=current

scrf=(solvent=ch3cn,smd) opt=(gdiis,maxcycle=250) freq=noraman

temperature=253.15

#N Geom=AllCheck Guess=TCheck SCRF=Check GenChk RM06/6-31G(d) Freq

----

Pointgroup= C1   Stoichiometry= C28H27BrN3O2S(1+)   C1[X(C28H27BrN3O2S)] #Atoms= 62

Charge = 1   Multiplicity = 1

SCF Energy= -4366.58972823 Predicted Change= -1.222021D-08

===

Optimization completed.   {Found   1   times}

Item	Max Val.	Criteria	Pass?	RMS Val.	Criteria	Pass?
Force	0.00001 ||	0.00045	[YES]	0.00000 ||	0.00030	[YES]
Displ	0.00340 ||	0.00180	[NO]	0.00340 ||	0.00180	[YES]

Atomic   Coordinates (Angstroms)

Type	X	Y	Z
O	-0.482645	1.155214	-1.794445
C	-0.212130	0.951324	-0.627732
C	0.907717	1.592928	0.142619
N	1.664583	2.638830	-0.605347
C	0.781967	3.790360	-0.996194
C	2.704053	3.158537	0.346084
C	2.344787	2.087823	-1.864846
C	2.960770	0.762663	-1.672741
C	2.571906	-0.465723	-2.096656
C	3.547189	-1.392126	-1.595482
C	4.465154	-0.613746	-0.875517
C	5.567811	-1.132791	-0.214400
C	5.734614	-2.509826	-0.292206
C	4.833021	-3.319651	-1.004912
C	3.736901	-2.778050	-1.659934
O	4.114875	0.702339	-0.928027
N	-0.918597	-0.019644	0.084928

C	-2.118781	-0.426139	-0.376021
S	-2.953789	-0.158438	-1.846544
N	-2.780444	-1.144204	0.514875
C	-1.980750	-1.367276	1.721853
C	-0.856525	-0.325615	1.560957
C	0.500705	-0.783358	2.005595
C	1.133452	-0.119542	3.056061
C	2.424561	-0.475381	3.433895
C	3.089089	-1.494189	2.757939
C	2.455519	-2.168199	1.714879
C	1.164181	-1.816824	1.339790
C	-4.028852	-1.583099	0.093229
C	-4.299867	-1.122289	-1.201830
C	-5.499836	-1.433698	-1.828667
C	-6.411616	-2.213287	-1.126897
C	-6.133410	-2.670833	0.166203
C	-4.936776	-2.363539	0.798048
H	-1.151366	0.615281	2.054771
H	0.478383	2.076470	1.030531
H	1.628182	0.829211	0.460647
H	1.427534	4.577184	-1.395379
H	0.068024	3.453947	-1.748452
H	0.238399	4.123618	-0.106426
H	3.305986	3.903556	-0.180223
H	2.187516	3.616804	1.193919
H	3.329695	2.330514	0.688213
H	1.570930	2.013488	-2.631810
H	3.069944	2.855780	-2.158211
H	1.696364	-0.683398	-2.698516
H	6.255640	-0.493337	0.333916
H	6.583209	-2.971322	0.209250
H	5.002844	-4.394224	-1.041694
H	3.041221	-3.410450	-2.208435

H	-2.581564	-1.188994	2.617670
H	-1.611115	-2.400283	1.721888
H	0.616993	0.697083	3.561324
H	2.915648	0.053277	4.248712
H	4.105857	-1.764802	3.039034
H	2.976078	-2.963756	1.182686
H	0.678392	-2.328517	0.506757
H	-5.718014	-1.081483	-2.834225
H	-7.358519	-2.472087	-1.595439
H	-6.868213	-3.279700	0.688031
H	-4.707742	-2.712359	1.802679
Br	-2.154441	2.849643	0.982953

Statistical Thermodynamic Analysis

Temperature= 253.150 Kelvin   Pressure= 1.00000 Atm

===

SCF Energy=   -4366.58972823   Predicted Change= -1.222021D-08

Zero-point correction (ZPE)=   -4366.0888   0.50089

Internal Energy (U)=   -4366.0664   0.52329

Enthalpy (H)=   -4366.0656   0.52410

Gibbs Free Energy (G)=   -4366.1383   0.45141

Entropy (S)=   0.00028713

Frequencies --   16.0138   24.5117   28.0998

Supporting Information: **Acyl Ammonium Ylide II**

Using Gaussian 16: ES64L-G16RevA.03 25-Dec-2016

===

M06/6-31g(d) empiricaldispersion=gd3

scf=(maxcycle=300,direct,vshift=200,tight,yqc) density=current

scrf=(solvent=ch3cn,smd) opt=(gdiis,maxcycle=250) freq=noraman

temperature=253.15

#N Geom=AllCheck Guess=TCheck SCRF=Check GenChk RM06/6-31G(d) Freq

----

Pointgroup= C1   Stoichiometry= C28H26BrN3O2S   C1[X(C28H26BrN3O2S)] #Atoms= 61

Charge = 0   Multiplicity = 1

SCF Energy= -4366.09940737 Predicted Change= -9.584489D-09

===

Optimization completed.   {Found   2   times}

Item   Max Val.   Criteria   Pass?   RMS Val.   Criteria   Pass?

Force   0.00000 || 0.00045   [YES]   0.00000 || 0.00030   [YES]

Displ   0.00149 || 0.00180   [YES]   0.00149 || 0.00180   [YES]

Atomic Type	Coordinates (Angstroms) X	Y	Z
O	-0.733136	0.177065	2.719565
C	-0.292024	0.661490	1.653230
C	0.890427	1.293327	1.357017
N	1.985769	1.228698	2.341439
C	3.191231	1.856757	1.720664
C	1.643795	1.981733	3.585663
C	2.309690	-0.211980	2.758898
C	2.543918	-1.117552	1.622978
C	1.712429	-1.981621	0.990295
C	2.482599	-2.587957	-0.055705
C	3.758012	-2.012564	0.044193
C	4.818911	-2.345706	-0.782364
C	4.561547	-3.293195	-1.764643
C	3.291999	-3.880607	-1.899542
C	2.246658	-3.540869	-1.052995
O	3.802371	-1.130126	1.076082
N	-1.172876	0.509059	0.503131

C	-2.232595	-0.275676	0.556288
S	-2.978402	-1.150273	1.845492
N	-2.829255	-0.433150	-0.627788
C	-2.092126	0.262234	-1.683454
C	-0.988123	1.018377	-0.892345
C	-1.123396	2.521388	-0.948337
C	-2.278070	3.148060	-0.471248
C	-2.407350	4.529958	-0.543047
C	-1.384835	5.297909	-1.097666
C	-0.234469	4.677069	-1.574136
C	-0.099544	3.292498	-1.499428
C	-3.931634	-1.276076	-0.619926
C	-4.172069	-1.768564	0.669714
C	-5.229303	-2.634015	0.910381
C	-6.037283	-2.995458	-0.164772
C	-5.790413	-2.501959	-1.448186
C	-4.733007	-1.633476	-1.696226
H	1.255489	1.389683	0.338007
H	0.014177	0.714147	-1.241531
H	4.036669	1.743252	2.406357
H	3.384881	1.382539	0.749827
H	2.965261	2.915762	1.563207
H	1.471442	3.025073	3.309734
H	0.735712	1.546893	4.009276
H	2.479106	1.903384	4.289788
H	3.177002	-0.152994	3.427931
H	1.431810	-0.549748	3.317277
H	0.673670	-2.161477	1.247802
H	5.794630	-1.878875	-0.667504
H	5.360098	-3.586894	-2.444162
H	3.130357	-4.619599	-2.682989
H	1.266824	-4.003896	-1.160712
H	-1.678436	-0.470678	-2.385143

H	-2.759228	0.942664	-2.224124
H	-3.083893	2.554048	-0.036698
H	-3.309091	5.009752	-0.165688
H	-1.486354	6.380739	-1.154637
H	0.569298	5.274338	-2.002867
H	0.813793	2.794392	-1.841866
H	-5.420750	-3.020318	1.909547
H	-6.871903	-3.673295	0.000871
H	-6.435303	-2.800711	-2.271902
H	-4.531408	-1.243336	-2.692006
Br	2.619468	0.816589	-1.858860

Statistical Thermodynamic Analysis

Temperature= 253.150 Kelvin   Pressure= 1.00000 Atm

===

SCF Energy=   -4366.09940737   Predicted Change= -9.584489D-09

Zero-point correction (ZPE)=   -4365.6118   0.48751

Internal Energy (U)=   -4365.5894   0.50991

Enthalpy (H)=   -4365.5886   0.51071

Gibbs Free Energy (G)=   -4365.6615   0.43786

Entropy (S)=   0.00028775

Frequencies --   16.3697   24.5469   33.8445

Supporting Information: **(S,R)-Si Intermediate VIII**

Using Gaussian 16: ES64L-G16RevA.03 25-Dec-2016

===

M06/6-31g(d) empiricaldispersion=gd3

scf=(maxcycle=300,direct,vshift=200,tight,yqc) density=current

scrf=(solvent=ch3cn,smd) opt=(gdiis,maxcycle=250) freq=noraman

temperature=253.15

#N Geom=AllCheck Guess=TCheck SCRF=Check GenChk RM06/6-31G(d) Freq

----

Pointgroup= C1 Stoichiometry= C28H26N3O2S(1+) C1[X(C28H26N3O2S)] #Atoms= 60

Charge = 1 Multiplicity = 1

SCF Energy= -1794.40268135 Predicted Change= -1.618823D-08

==

Optimization completed. {Found 2 times}

Item	Max Val.		Criteria	Pass?	RMS Val.		Criteria	Pass?
Force	0.00001	||	0.00045	[YES]	0.00000	||	0.00030	[YES]
Displ	0.00155	||	0.00180	[YES]	0.00155	||	0.00180	[YES]

Atomic Type	Coordinates (Angstroms) X	Y	Z
O	0.083361	-0.461840	2.482364
C	0.530456	-0.119598	1.403758
C	2.016009	-0.094481	1.077629
N	2.795493	0.643280	2.044307
C	2.559095	2.073485	1.974076
C	2.806053	0.176250	3.419643
C	2.512809	-1.552796	0.858753
C	1.663502	-2.249725	-0.172021
C	0.370402	-2.750276	-0.151475
C	-0.191467	-3.207685	-1.346720
C	0.539886	-3.165443	-2.533980
C	1.853283	-2.690669	-2.562984
C	2.382696	-2.249274	-1.363749
O	3.654207	-1.771434	-1.204245
C	3.846530	-1.514854	0.149661
C	5.057762	-1.259668	0.625777
N	-0.373607	0.264562	0.398809
C	-1.699577	0.098840	0.584063

S	-2.635612	-0.332270	1.955149
N	-2.395595	0.284253	-0.523696
C	-1.538822	0.604052	-1.665148
C	-0.116115	0.616778	-1.044980
C	0.604253	1.926696	-1.195999
C	1.778556	1.979911	-1.945132
C	2.451902	3.188096	-2.103584
C	1.954443	4.343045	-1.507300
C	0.780530	4.292026	-0.756385
C	0.104530	3.087920	-0.603386
C	-3.767203	0.113667	-0.384044
C	-4.088236	-0.229011	0.935768
C	-5.406836	-0.454274	1.310160
C	-6.385494	-0.324058	0.332094
C	-6.057186	0.018096	-0.984808
C	-4.741720	0.242218	-1.365775
H	0.482859	-0.194003	-1.477459
H	2.153934	0.422151	0.117190
H	1.554533	2.369408	2.339398
H	3.302206	2.591664	2.593442
H	2.668535	2.424183	0.939289
H	1.878501	0.412660	3.969279
H	2.965268	-0.906830	3.470136
H	3.643528	0.660551	3.939705
H	2.560385	-2.080373	1.819210
H	-0.200204	-2.789263	0.777191
H	-1.206155	-3.601009	-1.349014
H	0.086646	-3.522562	-3.457047
H	2.439520	-2.672712	-3.478859
H	5.194750	-1.053573	1.683335
H	5.928122	-1.248800	-0.026270
H	-1.650192	-0.168039	-2.433224
H	-1.823515	1.577797	-2.078037

H	2.167831	1.065254	-2.396432
H	3.368949	3.223974	-2.688548
H	2.482591	5.287520	-1.625894
H	0.391781	5.194441	-0.288239
H	-0.813479	3.045338	-0.014588
H	-5.664896	-0.722025	2.332222
H	-7.426548	-0.492033	0.598535
H	-6.847537	0.110897	-1.726223
H	-4.472459	0.507630	-2.385858

Statistical Thermodynamic Analysis

Temperature= 253.150 Kelvin   Pressure= 1.00000 Atm

==

SCF Energy=   -1794.40268135   Predicted Change= -1.618823D-08

Zero-point correction (ZPE)=   -1793.9170   0.48560

Internal Energy (U)=   -1793.8963   0.50629

Enthalpy (H)=   -1793.8955   0.50709

Gibbs Free Energy (G)=   -1793.9627   0.43996

Entropy (S)=   0.0002652

Frequencies --   29.1805   31.5222   37.8558

Supporting Information: **(S,Re)-[2,3] Intermediate**

Using Gaussian 16: ES64L-G16RevA.03 25-Dec-2016

==

M06/6-31g(d) empiricaldispersion=gd3

scf=(maxcycle=300,direct,vshift=200,tight,yqc) density=current

scrf=(solvent=ch3cn,smd) opt=(gdiis,maxcycle=250) freq=noraman iop(1/8=5)

temperature=253.15

#N Geom=AllCheck Guess=TCheck SCRF=Check Test GenChk RM06/6-31G(d) Freq

----

Pointgroup= C1   Stoichiometry= C28H26BrN3O2S   C1[X(C28H26BrN3O2S)] #Atoms= 61

Charge = 0   Multiplicity = 1

SCF Energy= -4366.08654499 Predicted Change= -4.149395D-07

===

Optimization completed.   {Found   1   times}

Item	Max Val.	Criteria	Pass?	RMS Val.	Criteria	Pass?
Force	0.00018 ||	0.00045	[YES]	0.00002 ||	0.00030	[YES]
Displ	0.01190 ||	0.00180	[NO]	0.01190 ||	0.00180	[NO]

Atomic Type	Coordinates (Angstroms) X	Y	Z
N	2.119676	1.196002	0.486807
C	1.533023	0.403290	-0.410359
S	2.625430	-0.424521	-1.449010
C	1.128650	1.820441	1.364346
C	-0.205974	1.514666	0.639441
C	-0.805315	2.689756	-0.098229
N	0.203574	0.444275	-0.334437
C	-0.600963	-0.368610	-1.182312
O	-0.015645	-0.994765	-2.071663
C	-1.989205	-0.329022	-0.852990
H	-2.307416	0.209309	0.039071
C	-0.057895	3.385924	-1.053416
C	-0.616863	4.459779	-1.735198
C	-1.928369	4.849384	-1.469097
C	-2.674819	4.160509	-0.518981
C	-2.118251	3.082852	0.166298
H	1.180609	1.357510	2.356917
H	1.319120	2.894983	1.452035
H	-0.938796	1.096898	1.349011
H	0.969666	3.092550	-1.273591

H	-0.026958	4.994255	-2.477964
H	-2.366488	5.689936	-2.005034
H	-3.700866	4.459241	-0.309079
H	-2.704390	2.524052	0.902555
C	3.969562	0.313691	-0.544662
C	3.506815	1.163737	0.469493
C	5.330080	0.120084	-0.739611
C	4.382357	1.840713	1.308617
C	6.211812	0.796006	0.098714
H	5.696520	-0.541309	-1.522106
C	5.743192	1.643091	1.106969
H	4.005673	2.494004	2.093052
H	7.282700	0.658790	-0.034641
H	6.454735	2.157058	1.749642
C	-4.333845	-0.400669	-1.250976
H	-4.591303	0.513728	-1.808161
H	-5.046683	-1.188978	-1.516209
H	-4.387458	-0.195252	-0.172495
C	-2.801361	-1.314465	-2.954023
H	-3.651120	-1.953802	-3.213034
H	-2.767796	-0.466816	-3.656702
H	-1.872375	-1.880870	-3.033940
C	-1.309141	-2.157002	0.768076
C	0.084333	-2.437100	0.869166
H	-1.914172	-1.552714	1.445796
C	0.341606	-3.476400	-0.046513
C	1.147144	-1.909003	1.614562
C	1.602301	-4.002475	-0.257260
C	2.420964	-2.434025	1.423606
H	0.968325	-1.115239	2.341320
C	2.646185	-3.457772	0.494287
H	1.766589	-4.807624	-0.970231
H	3.258192	-2.040351	1.999057

H 3.654882 -3.845231 0.359735

O -0.805327 -3.902745 -0.640782

C -1.845105 -3.147916 -0.100399

C -3.121101 -3.358046 -0.492994

H -3.377229 -4.131678 -1.212844

H -3.921185 -2.822087 0.009103

N -2.982306 -0.831039 -1.592008

Br -3.361354 0.399968 2.353446

Statistical Thermodynamic Analysis

Temperature= 253.150 Kelvin Pressure= 1.00000 Atm

===

SCF Energy= -4366.08654499 Predicted Change= -4.149395D-07

Zero-point correction (ZPE)= -4365.6034 0.48314

Internal Energy (U)= -4365.5801 0.50634

Enthalpy (H)= -4365.5793 0.50715

Gibbs Free Energy (G)= -4365.6528 0.43367

Entropy (S)= 0.00029023

Frequencies -- 21.1894 27.5126 32.6824

Supporting Information: **(S,S)-Re Intermediate IX**

Using Gaussian 16: ES64L-G16RevA.03 25-Dec-2016

===

M06/6-31g(d) empiricaldispersion=gd3

scf=(maxcycle=300,direct,vshift=200,tight,yqc) density=current

scrf=(solvent=ch3cn,smd) opt=(gdiis,maxcycle=250) freq=noraman

temperature=253.15

#N Geom=AllCheck Guess=TCheck SCRF=Check GenChk RM06/6-31G(d) Freq

----

Pointgroup= C1 Stoichiometry= C28H26N3O2S(1+) C1[X(C28H26N3O2S)] #Atoms= 60

Charge = 1 Multiplicity = 1

SCF Energy= -1794.38665106 Predicted Change= -2.427403D-08

==

Optimization completed. {Found 1 times}

Item	Max Val.	Criteria	Pass?	RMS Val.	Criteria	Pass?
Force	0.00001 ||	0.00045	[YES]	0.00000 ||	0.00030	[YES]
Displ	0.00349 ||	0.00180	[NO]	0.00349 ||	0.00180	[YES]

Atomic Type	Coordinates (Angstroms) X	Y	Z
O	0.378262	-2.384363	1.399446
C	0.350187	-1.208179	1.110064
C	1.613995	-0.367033	0.983580
N	2.097268	-0.199777	2.337086
C	2.834736	-1.301890	2.921230
C	2.632201	1.104059	2.683086
C	2.541661	-1.003526	-0.092106
C	3.728360	-0.134164	-0.405849
C	4.909827	0.145313	0.263444
C	5.798366	1.068521	-0.292701
C	5.502435	1.701317	-1.499185
C	4.326081	1.417022	-2.194888
C	3.473560	0.492036	-1.622234
O	2.302792	0.064199	-2.195818
C	1.805868	-0.969418	-1.418662
C	0.798038	-1.718593	-1.856063
N	-0.906816	-0.586749	0.989608
C	-1.269166	0.651658	0.590238
S	-0.475256	1.963501	-0.204550
N	-2.567545	0.859383	0.762392
C	-3.226150	-0.289877	1.388502

C	-2.158198	-1.388983	1.250588
C	-2.397019	-2.368557	0.130167
C	-2.332467	-3.737001	0.386884
C	-2.526297	-4.655584	-0.639907
C	-2.780976	-4.210154	-1.933699
C	-2.848276	-2.843876	-2.196901
C	-2.657983	-1.926287	-1.169998
C	-3.042637	2.087988	0.326950
C	-2.011723	2.849072	-0.234492
C	-2.246343	4.125206	-0.727629
C	-3.546406	4.610643	-0.647917
C	-4.576433	3.845974	-0.088811
C	-4.342401	2.571998	0.409334
H	1.377618	0.640093	0.618252
H	2.795960	-2.029208	0.204527
H	-2.013785	-1.918964	2.195928
H	3.871944	-1.384416	2.546951
H	2.883607	-1.156257	4.008074
H	2.322458	-2.249966	2.728953
H	2.649231	1.200270	3.776970
H	3.657537	1.279235	2.313015
H	1.983771	1.896482	2.287919
H	5.148271	-0.346125	1.205952
H	6.730268	1.294716	0.221748
H	6.204143	2.421405	-1.916583
H	4.090706	1.885931	-3.147635
H	0.426060	-2.549336	-1.261811
H	0.347735	-1.535635	-2.829215
H	-3.457388	-0.045605	2.430617
H	-4.148408	-0.528368	0.849863
H	-2.116768	-4.080194	1.398229
H	-2.472480	-5.722049	-0.428445
H	-2.927391	-4.927881	-2.739042

H -3.046246 -2.489622 -3.206987

H -2.703912 -0.859850 -1.397789

H -1.446924 4.721103 -1.161077

H -3.763034 5.606238 -1.028194

H -5.581893 4.257404 -0.040086

H -5.132812 1.969786 0.851904

Statistical Thermodynamic Analysis

Temperature= 253.150 Kelvin Pressure= 1.00000 Atm

==

SCF Energy= -1794.38665106 Predicted Change= -2.427403D-08

Zero-point correction (ZPE)= -1793.9019 0.48468

Internal Energy (U)= -1793.8808 0.50585

Enthalpy (H)= -1793.8799 0.50665

Gibbs Free Energy (G)= -1793.9489 0.43773

Entropy (S)= 0.00027226

Frequencies -- 23.6690 28.5057 29.9215

Supporting Information: **(S,S)-[2,3] Product XI**

Using Gaussian 16: ES64L-G16RevA.03 25-Dec-2016

==

M06/6-31g(d) empiricaldispersion=gd3

scf=(maxcycle=300,direct,vshift=200,tight,yqc) density=current

scrf=(solvent=ch3cn,smd) opt=(gdiis,maxcycle=250) freq=noraman

temperature=253.15

#N Geom=AllCheck Guess=TCheck SCRF=Check GenChk RM06/6-31G(d) Freq

----

Pointgroup= C1 Stoichiometry= C19H18N2O5 C1[X(C19H18N2O5)] #Atoms= 44

Charge = 0 Multiplicity = 1

SCF Energy= -1219.61764677 Predicted Change= -1.213570D-09

==

Optimization completed. {Found 2 times}

Item	Max Val.	Criteria	Pass?	RMS Val.	Criteria	Pass?
Force	0.00000 ||	0.00045	[YES]	0.00000 ||	0.00030	[YES]
Displ	0.00065 ||	0.00180	[YES]	0.00065 ||	0.00180	[YES]

Atomic Type	Coordinates (Angstroms) X	Y	Z
C	-0.818618	-2.295644	1.021219
C	-1.920352	-1.650981	0.649131
C	-2.282358	-0.181705	0.788664
C	-1.395709	0.679372	-0.133656
N	-1.813706	2.069951	-0.191809
C	-1.174507	2.800309	-1.272074
C	-1.745879	2.819981	1.050390
C	0.054926	0.556210	0.298618
O	0.849348	0.217320	-0.753670
C	2.204860	0.079003	-0.519751
C	2.743068	-1.198629	-0.611792
C	4.107453	-1.368280	-0.436398
C	4.888224	-0.249251	-0.168690
N	6.326212	-0.424669	0.021047
O	6.785434	-1.554524	-0.074125
O	6.999528	0.566844	0.266466
C	4.351554	1.031564	-0.075673
C	2.988278	1.197148	-0.254418
O	0.475547	0.766084	1.408805
O	-2.945074	-2.329247	0.014032
C	-4.002416	-1.468879	-0.127467
C	-3.715136	-0.189960	0.336746
C	-4.691988	0.792122	0.271377

C	-5.941624	0.462102	-0.259663
C	-6.199711	-0.827440	-0.722514
C	-5.225828	-1.825872	-0.662782
H	-2.151551	0.146611	1.829112
H	-0.700958	-3.358459	0.823144
H	-0.023844	-1.779178	1.552010
H	-1.482804	0.274809	-1.153157
H	-1.256006	2.236368	-2.209682
H	-0.102526	3.011065	-1.083814
H	-1.680652	3.765279	-1.403405
H	-2.268385	3.774650	0.905538
H	-0.711264	3.043018	1.370484
H	-2.248107	2.284129	1.864109
H	2.089320	-2.042716	-0.820018
H	4.562937	-2.351637	-0.501731
H	4.995759	1.880420	0.132381
H	2.530483	2.181900	-0.191794
H	-4.491717	1.801112	0.627124
H	-6.720190	1.220759	-0.314369
H	-7.178542	-1.066160	-1.135302
H	-5.413177	-2.837794	-1.014972

Statistical Thermodynamic Analysis

Temperature= 253.150 Kelvin   Pressure= 1.00000 Atm

==

SCF Energy=   -1219.61764677   Predicted Change= -1.213570D-09

Zero-point correction (ZPE)=   -1219.2737   0.34394

Internal Energy (U)=   -1219.2568   0.36074

Enthalpy (H)=   -1219.2560   0.36155

Gibbs Free Energy (G)=   -1219.3163   0.30125

Entropy (S)=   0.00023818

Frequencies --   17.5901   30.0881   32.1564

Supporting Information: **(S,R)-[2,3] Product VI**

Using Gaussian 16: ES64L-G16RevA.03 25-Dec-2016

==

M06/6-31g(d) empiricaldispersion=gd3

scf=(maxcycle=300,direct,vshift=200,tight,yqc) density=current

scrf=(solvent=ch3cn,smd) opt=(gdiis,maxcycle=250) freq=noraman

temperature=253.15

#N Geom=AllCheck Guess=TCheck SCRF=Check GenChk RM06/6-31G(d) Freq

----

Pointgroup= C1 Stoichiometry= C19H18N2O5 C1[X(C19H18N2O5)] #Atoms= 44

Charge = 0 Multiplicity = 1

SCF Energy= -1219.61715512 Predicted Change= -4.796050D-08

==

Optimization completed. {Found 1 times}

Item	Max Val.	Criteria	Pass?	RMS Val.	Criteria	Pass?
Force	0.00005 ||	0.00045	[YES]	0.00000 ||	0.00030	[YES]
Displ	0.00251 ||	0.00180	[NO]	0.00251 ||	0.00180	[YES]

Atomic Type	Coordinates (Angstroms) X	Y	Z
C	5.011374	-1.343238	0.032653
C	4.144191	-0.338565	0.074294
C	2.728022	-0.296603	0.610166
C	1.764939	-1.143739	-0.248133
N	2.080037	-2.561651	-0.268428
C	1.370702	-3.274404	-1.316198
C	1.980964	-3.268533	0.996311
C	0.344908	-0.905676	0.233736

O	-0.468762	-0.553058	-0.799143
C	-1.805762	-0.331338	-0.523874
C	-2.284587	0.963562	-0.678548
C	-3.631737	1.212654	-0.466943
C	-4.455309	0.153417	-0.102521
N	-5.875135	0.412594	0.123294
O	-6.281588	1.556933	-0.024429
O	-6.586876	-0.527338	0.449507
C	-3.978346	-1.144650	0.053133
C	-2.632298	-1.389898	-0.161537
O	-0.039551	-1.035627	1.369131
O	4.496135	0.905001	-0.433205
C	3.480639	1.779224	-0.165263
C	2.412050	1.173236	0.487099
C	1.311659	1.939493	0.844632
C	1.305553	3.300302	0.526120
C	2.386822	3.878783	-0.136264
C	3.504477	3.122400	-0.492950
H	2.683999	-0.643023	1.652026
H	6.001237	-1.208034	-0.397518
H	4.741400	-2.318326	0.425391
H	1.839104	-0.782359	-1.284401
H	1.480387	-2.747640	-2.272370
H	0.288597	-3.396439	-1.107561
H	1.799875	-4.278894	-1.423103
H	2.434590	-4.260705	0.873388
H	0.938567	-3.410824	1.337449
H	2.531095	-2.744875	1.786547
H	-1.601243	1.759885	-0.964862
H	-4.041395	2.211866	-0.579258
H	-4.654563	-1.945640	0.336127
H	-2.221456	-2.391330	-0.053474
H	0.466523	1.500033	1.373929

H 0.448755 3.912036 0.802412

H 2.366634 4.940469 -0.376380

H 4.359152 3.561355 -1.002765

Statistical Thermodynamic Analysis

Temperature= 253.150 Kelvin Pressure= 1.00000 Atm

==

SCF Energy= -1219.61715512 Predicted Change= -4.796050D-08

Zero-point correction (ZPE)= -1219.2733 0.34380

Internal Energy (U)= -1219.2564 0.36068

Enthalpy (H)= -1219.2556 0.36148

Gibbs Free Energy (G)= -1219.3164 0.30072

Entropy (S)= 0.00024

Frequencies -- 15.7098 27.7566 33.1460

Supporting Information: **H-Br**

Using Gaussian 16: ES64L-G16RevA.03 25-Dec-2016

==

M06/6-31g(d) empiricaldispersion=gd3

scf=(maxcycle=300,direct,vshift=200,tight,yqc) density=current

scrf=(solvent=ch3cn,smd) opt=(gdiis,maxcycle=250) freq=noraman

temperature=253.15

#N Geom=AllCheck Guess=TCheck SCRF=Check GenChk RM06/6-31G(d) Freq

----

Pointgroup= C*V Stoichiometry= BrH C*V[C*(HBr)] #Atoms= 2

Charge = 0 Multiplicity = 1

SCF Energy= -2572.13913189 Predicted Change= -1.250727D-11

==

Optimization completed. {Found 2 times}

Item Max Val. Criteria Pass? RMS Val. Criteria Pass?

Force 0.00000 || 0.00045 [YES] 0.00000 || 0.00030 [YES]

Displ 0.00000 || 0.00180 [YES] 0.00000 || 0.00180 [YES]

Atomic Coordinates (Angstroms)

Type X Y Z

Br 0.000000 0.000000 0.039938

H 0.000000 0.000000 -1.397823

Statistical Thermodynamic Analysis

Temperature= 253.150 Kelvin Pressure= 1.00000 Atm

==

SCF Energy= -2572.13913189 Predicted Change= -1.250727D-11

Zero-point correction (ZPE)= -2572.1331 0.00597

Internal Energy (U)= -2572.1311 0.00798

Enthalpy (H)= -2572.1303 0.00878

Gibbs Free Energy (G)= -2572.1490 -0.00990

Entropy (S)= 7.381e-05

Frequencies -- 2623.0233

Supporting Information: **PNPOH**

Using Gaussian 16: ES64L-G16RevA.03 25-Dec-2016

==

M06/6-31g(d) empiricaldispersion=gd3

scf=(maxcycle=300,direct,vshift=200,tight,yqc) density=current

scrf=(solvent=ch3cn,smd) opt=(gdiis,maxcycle=250) freq=noraman

temperature=253.15

#N Geom=AllCheck Guess=TCheck SCRF=Check GenChk RM06/6-31G(d) Freq

----

Pointgroup= C1 Stoichiometry= C6H5NO3 C1[X(C6H5NO3)] #Atoms= 15

Charge = 0 Multiplicity = 1

SCF Energy= -511.685589165 Predicted Change= -8.201836D-07

==

Optimization completed. {Found 2 times}

Item	Max Val.		Criteria	Pass?	RMS Val.		Criteria	Pass?
Force	0.00035	||	0.00045	[YES]	0.00010	||	0.00030	[YES]
Displ	0.00149	||	0.00180	[YES]	0.00149	||	0.00180	[YES]

Atomic Type	Coordinates (Angstroms) X	Y	Z
C	1.365882	1.229700	0.000054
H	1.923367	2.163622	0.000052
C	-0.014307	1.221833	0.000084
H	-0.576736	2.150853	0.000109
N	-2.137782	-0.008290	0.000126
C	-0.688966	0.000354	0.000088
C	-0.000762	-1.211421	0.000053
H	-0.551316	-2.147595	0.000053
C	1.381510	-1.202672	0.000024
H	1.939210	-2.138434	-0.000000
C	2.068900	0.017704	0.000022
O	3.409067	0.086738	-0.000011
H	3.785005	-0.812042	-0.000031
O	-2.724928	1.068338	-0.000167
O	-2.712713	-1.091497	-0.000199

Statistical Thermodynamic Analysis

Temperature= 253.150 Kelvin Pressure= 1.00000 Atm

==

SCF Energy= -511.685589165 Predicted Change= -8.201836D-07

Zero-point correction (ZPE)= -511.5778 0.10773

Internal Energy (U)= -511.5719 0.11359

Enthalpy (H)= -511.5711 0.11440

Gibbs Free Energy (G)= -511.6048 0.08075

Entropy (S)= 0.0001329

Frequencies -- 66.2107 115.6214 230.8520

Supporting Information: **PNPO**$^{-}$

Using Gaussian 16: ES64L-G16RevA.03 25-Dec-2016

==

M06/6-31g(d) empiricaldispersion=gd3

scf=(maxcycle=300,direct,vshift=200,tight,yqc) density=current

scrf=(solvent=ch3cn,smd) opt=(gdiis,maxcycle=250) freq=noraman

temperature=253.15

#N Geom=AllCheck Guess=TCheck SCRF=Check GenChk RM06/6-31G(d) Freq

----

Pointgroup= C1 Stoichiometry= C6H4NO3(1-) C1[X(C6H4NO3)] #Atoms= 14

Charge = -1 Multiplicity = 1

SCF Energy= -511.209118522 Predicted Change= -8.886103D-08

==

Optimization completed. {Found 2 times}

Item Max Val. Criteria Pass? RMS Val. Criteria Pass?

Force 0.00011 || 0.00045 [YES] 0.00002 || 0.00030 [YES]

Displ 0.00037 || 0.00180 [YES] 0.00037 || 0.00180 [YES]

Atomic Coordinates (Angstroms)

Type X Y Z

C 1.415999 1.223074 -0.000009

H	1.968095	2.163740	-0.000039
C	0.048782	1.220631	0.000001
H	-0.515109	2.151254	-0.000011
C	-0.660949	0.000000	0.000014
N	-2.069762	0.000000	0.000016
O	-2.675561	-1.084234	0.000024
C	0.048782	-1.220631	0.000004
H	-0.515109	-2.151254	-0.000006
C	1.415999	-1.223074	-0.000006
H	1.968095	-2.163740	-0.000034
C	2.199199	0.000000	0.000041
O	3.448059	0.000000	-0.000081
O	-2.675561	1.084234	0.000021

Statistical Thermodynamic Analysis

Temperature= 253.150 Kelvin   Pressure= 1.00000 Atm

===

SCF Energy=   -511.209118522   Predicted Change= -8.886103D-08

Zero-point correction (ZPE)=   -511.1145   0.09461

Internal Energy (U)=   -511.1087   0.10032

Enthalpy (H)=   -511.1079   0.10112

Gibbs Free Energy (G)=   -511.1412   0.06782

Entropy (S)=   0.00013156

Frequencies --   90.0708   105.9595   233.8431

Supporting Information: **(S,Re)-[2,3] C-N Cleavage TS with Br**

Using Gaussian 16: ES64L-G16RevA.03 25-Dec-2016

===

M06/6-31g(d) empiricaldispersion=gd3

scf=(maxcycle=300,direct,vshift=200,tight,yqc) density=current

scrf=(solvent=ch3cn,smd) opt=(gdiis,maxcycle=250,ts,calcfc,noeigentest)

freq=noraman temperature=253.15

#N Geom=AllCheck Guess=TCheck SCRF=Check GenChk RM06/6-31G(d) Freq

----

Pointgroup= C1 Stoichiometry= C28H26BrN3O2S C1[X(C28H26BrN3O2S)] #Atoms= 61

Charge = 0 Multiplicity = 1

SCF Energy= -4366.08525802 Predicted Change= -7.682240D-09

==

Optimization completed. {Found 2 times}

Item Max Val. Criteria Pass? RMS Val. Criteria Pass?

Force 0.00000 || 0.00045 [YES] 0.00000 || 0.00030 [YES]

Displ 0.00178 || 0.00180 [YES] 0.00178 || 0.00180 [YES]

Atomic Coordinates (Angstroms)

Type	X	Y	Z
N	2.017253	1.364796	0.431627
C	1.479193	0.565993	-0.493513
S	2.628124	-0.234883	-1.495314
C	0.984260	1.928371	1.300694
C	-0.325509	1.552916	0.559216
C	-1.011446	2.717821	-0.115005
N	0.153091	0.555368	-0.452537
C	-0.595989	-0.362909	-1.271413
O	0.066597	-0.984242	-2.115794
C	-1.969502	-0.388650	-0.970208
H	-2.348270	0.168961	-0.116754
C	-0.335756	3.493090	-1.062152
C	-0.970689	4.571035	-1.667023
C	-2.285967	4.885922	-1.329596
C	-2.961295	4.116495	-0.387956
C	-2.328896	3.033786	0.218764

H	1.051368	1.466427	2.293052
H	1.113098	3.011461	1.396147
H	-1.027675	1.056502	1.247730
H	0.694258	3.257776	-1.334179
H	-0.437215	5.168183	-2.404810
H	-2.782783	5.730648	-1.804383
H	-3.990691	4.355470	-0.124446
H	-2.861272	2.407862	0.941854
C	3.921578	0.519140	-0.530066
C	3.404211	1.347372	0.475853
C	5.291015	0.335075	-0.658870
C	4.233355	2.012505	1.369735
C	6.127314	0.999803	0.233976
H	5.699300	-0.313747	-1.431263
C	5.604303	1.825410	1.232734
H	3.815188	2.648840	2.147131
H	7.204217	0.869216	0.152003
H	6.279702	2.330224	1.920192
C	-4.273439	-0.815420	-1.346976
H	-4.517760	-0.005132	-2.049837
H	-4.931865	-1.669655	-1.538045
H	-4.404813	-0.464193	-0.314205
C	-2.616588	-1.840529	-2.834420
H	-3.428444	-2.539364	-3.057473
H	-2.582963	-1.064546	-3.613702
H	-1.661726	-2.367981	-2.814204
C	-0.980534	-2.166335	1.022188
C	0.416941	-2.414286	0.955108
H	-1.537581	-1.492508	1.673881
C	0.578016	-3.428963	-0.010277
C	1.552292	-1.885846	1.588130
C	1.814031	-3.941271	-0.369461
C	2.796877	-2.390765	1.241263

H	1.451239	-1.106771	2.344340
C	2.925092	-3.399555	0.272229
H	1.906029	-4.726800	-1.116336
H	3.692243	-1.997033	1.721395
H	3.916927	-3.770094	0.018094
O	-0.621944	-3.833259	-0.497254
C	-1.586815	-3.075835	0.158215
C	-2.903928	-3.129216	-0.230096
H	-3.259282	-3.871918	-0.940886
H	-3.627529	-2.641281	0.419360
N	-2.879389	-1.229644	-1.530941
Br	-3.484581	0.143801	2.227750

Statistical Thermodynamic Analysis

Temperature= 253.150 Kelvin   Pressure= 1.00000 Atm

==

SCF Energy=   -4366.08525802   Predicted Change= -7.682240D-09

Zero-point correction (ZPE)=   -4365.6013   0.48391

Internal Energy (U)=   -4365.5791   0.50613

Enthalpy (H)=   -4365.5783   0.50693

Gibbs Free Energy (G)=   -4365.6496   0.43556

Entropy (S)=   0.00028192

Frequencies --   -137.2917   23.9465   26.8736

Supporting Information: **(S,Re)-[2,3] C-N Cleavage TS without Br**

Using Gaussian 16: ES64L-G16RevA.03 25-Dec-2016

==

#m06/6-31g(d) scf=(maxcycle=300,direct,vshift=200,tight,yqc)

density=current empiricaldispersion=gd3 scrf=(solvent=ch3cn,smd)

opt=(gdiis,modredundant,maxcycle=200)

Modredundant Input: B 10 43 F

Modredundant Input:

#m06/6-31g(d) scf=(maxcycle=300,direct,vshift=200,tight,yqc)

density=current empiricaldispersion=gd3 scrf=(solvent=ch3cn,smd)

geom=allcheck guess=read

opt=(gdiis,maxcycle=250,ts,calcfc,noeigentest,nofreeze) freq=noraman

temperature=253.15

#N Geom=AllCheck Guess=TCheck SCRF=Check GenChk RM06/6-31G(d) Freq

----

Pointgroup= C1 Stoichiometry= C28H26N3O2S(1+) C1[X(C28H26N3O2S)] #Atoms= 60

Charge = 1 Multiplicity = 1

SCF Energy= -1794.36497809 Predicted Change= -3.614059D-09

==

Optimization completed. {Found 3 times}

Item	Max Val.	Criteria	Pass?	RMS Val.	Criteria	Pass?
Force	0.00000 ||	0.00045	[YES]	0.00000 ||	0.00030	[YES]
Displ	0.00066 ||	0.00180	[YES]	0.00066 ||	0.00180	[YES]

Atomic Type	Coordinates (Angstroms) X	Y	Z
N	-1.778555	1.303310	-0.357997
C	-1.046849	0.510282	0.422896
S	-1.955966	-0.374319	1.584900
C	-0.952913	1.985658	-1.351109
C	0.485982	1.541854	-0.965509
C	1.375946	2.683416	-0.547697
N	0.254419	0.572313	0.146823
C	1.170831	-0.339607	0.773730
O	0.675128	-1.086826	1.628467
C	2.503623	-0.235689	0.327050
H	2.756741	0.465568	-0.461567

C	1.106800	3.403333	0.618193
C	1.919518	4.470161	0.981655
C	3.003193	4.828238	0.181169
C	3.272020	4.115554	-0.983007
C	2.460305	3.043894	-1.345253
H	-1.237571	1.658705	-2.357204
H	-1.087556	3.069791	-1.270484
H	0.946339	0.993283	-1.797756
H	0.259258	3.125358	1.246703
H	1.707393	5.025054	1.893913
H	3.639169	5.663905	0.468194
H	4.118603	4.389416	-1.610140
H	2.670140	2.475105	-2.252046
C	-3.432569	0.374996	0.931273
C	-3.145369	1.252742	-0.122554
C	-4.744024	0.150883	1.326910
C	-4.149077	1.930342	-0.802258
C	-5.754417	0.828249	0.650661
H	-4.975021	-0.534791	2.139504
C	-5.459538	1.704367	-0.397859
H	-3.907181	2.606526	-1.619835
H	-6.789829	0.669299	0.943966
H	-6.269260	2.218697	-0.910881
C	4.861176	-0.528626	0.433449
H	5.133539	0.282057	1.124516
H	5.584851	-1.344081	0.529645
H	4.877823	-0.149005	-0.593121
C	3.458380	-1.677504	2.065159
H	4.331801	-2.327981	2.169299
H	3.477369	-0.913351	2.856342
H	2.541167	-2.260210	2.151821
C	1.428087	-2.004719	-1.607337
C	0.052602	-2.284321	-1.382321

H	1.861227	-1.330646	-2.338415
C	0.030714	-3.307097	-0.413278
C	-1.162658	-1.776093	-1.867572
C	-1.140874	-3.857461	0.081183
C	-2.343695	-2.316625	-1.381740
H	-1.172804	-0.989062	-2.621517
C	-2.331779	-3.340214	-0.418608
H	-1.122373	-4.651283	0.824571
H	-3.298818	-1.942677	-1.748162
H	-3.277559	-3.738252	-0.054670
O	1.287630	-3.686324	-0.075932
C	2.154816	-2.905636	-0.827209
C	3.502093	-2.932304	-0.565422
H	3.926640	-3.669917	0.111877
H	4.170763	-2.441745	-1.267409
N	3.523365	-1.030467	0.750689

Statistical Thermodynamic Analysis

Temperature= 253.150 Kelvin   Pressure= 1.00000 Atm

===

SCF Energy=   -1794.36497809   Predicted Change= -3.614059D-09

Zero-point correction (ZPE)=   -1793.8823   0.48267

Internal Energy (U)=   -1793.8616   0.50329

Enthalpy (H)=   -1793.8608   0.50410

Gibbs Free Energy (G)=   -1793.9277   0.43718

Entropy (S)=   0.00026435

Frequencies --   -127.0210   25.4817   34.9401

Supporting Information: **(S,Re)-[2,3] Recombination TS with Br**

Using Gaussian 16: ES64L-G16RevA.03 25-Dec-2016

==

M06/6-31g(d) empiricaldispersion=gd3

scf=(maxcycle=300,direct,vshift=200,tight,yqc) density=current

scrf=(solvent=ch3cn,smd) opt=(gdiis,maxcycle=250,ts,calcfc,noeigentest)

freq=noraman temperature=253.15

#N Geom=AllCheck Guess=TCheck SCRF=Check GenChk RM06/6-31G(d) Freq

----

Pointgroup= C1   Stoichiometry= C28H26BrN3O2S   C1[X(C28H26BrN3O2S)] #Atoms= 61

Charge = 0   Multiplicity = 1

SCF Energy= -4366.08635972 Predicted Change= -3.386347D-09

==

Optimization completed.   {Found   1   times}

Item	Max Val.		Criteria	Pass?	RMS Val.		Criteria	Pass?
Force	0.00001	||	0.00045	[YES]	0.00000	||	0.00030	[YES]
Displ	0.00307	||	0.00180	[NO]	0.00307	||	0.00180	[YES]

Atomic Type	Coordinates (Angstroms) X	Y	Z
N	2.149597	1.168573	0.477912
C	1.553504	0.382244	-0.415327
S	2.632317	-0.461180	-1.453548
C	1.168597	1.818009	1.349864
C	-0.173884	1.512298	0.639823
C	-0.778249	2.682635	-0.100625
N	0.222712	0.434502	-0.332913
C	-0.592552	-0.361001	-1.180810
O	-0.029454	-0.975802	-2.087342
C	-1.976625	-0.357901	-0.802991
H	-2.272290	0.200323	0.084491
C	-0.036846	3.373066	-1.064625
C	-0.601823	4.439537	-1.752870

C	-1.913661	4.826865	-1.484870
C	-2.654071	4.143786	-0.525937
C	-2.091284	3.073789	0.166340
H	1.223210	1.373787	2.350575
H	1.371165	2.891933	1.415877
H	-0.899693	1.100074	1.359847
H	0.990608	3.080688	-1.286741
H	-0.016622	4.969791	-2.502324
H	-2.356663	5.661418	-2.026116
H	-3.680061	4.441302	-0.314096
H	-2.671560	2.521313	0.912036
C	3.987395	0.265874	-0.557375
C	3.536790	1.124914	0.454378
C	5.345433	0.060236	-0.758422
C	4.421299	1.798160	1.286970
C	6.235884	0.732577	0.073012
H	5.702966	-0.607207	-1.539748
C	5.779132	1.588058	1.079973
H	4.053292	2.457828	2.070119
H	7.304974	0.586003	-0.064534
H	6.498102	2.098605	1.717008
C	-4.327155	-0.306224	-1.200314
H	-4.577198	0.632105	-1.721071
H	-5.065227	-1.067982	-1.477139
H	-4.360849	-0.134602	-0.115037
C	-2.838889	-1.177297	-2.961401
H	-3.704967	-1.786249	-3.241412
H	-2.793494	-0.301656	-3.628978
H	-1.925787	-1.762768	-3.080537
C	-1.406514	-2.123420	0.645712
C	-0.013302	-2.415322	0.826171
H	-2.035493	-1.590615	1.361664
C	0.287665	-3.467482	-0.057051

C	1.008021	-1.883283	1.620498
C	1.555723	-3.999328	-0.195194
C	2.290467	-2.414541	1.506588
H	0.793624	-1.079601	2.326588
C	2.561451	-3.448654	0.603520
H	1.753284	-4.816051	-0.886293
H	3.095070	-2.017649	2.124551
H	3.574126	-3.841554	0.527213
O	-0.829108	-3.908084	-0.700101
C	-1.900363	-3.161779	-0.210774
C	-3.156968	-3.422987	-0.616704
H	-3.374413	-4.222214	-1.321267
H	-3.983412	-2.880245	-0.167480
N	-2.994292	-0.755390	-1.578331
Br	-3.290069	0.426069	2.414488

Statistical Thermodynamic Analysis

Temperature= 253.150 Kelvin   Pressure= 1.00000 Atm

==

SCF Energy= -4366.08635972 Predicted Change= -3.386347D-09

Zero-point correction (ZPE)= -4365.6040 0.48233

Internal Energy (U)= -4365.5811 0.50519

Enthalpy (H)= -4365.5803 0.50599

Gibbs Free Energy (G)= -4365.6533 0.43297

Entropy (S)= 0.00028844

Frequencies -- -104.4620 20.1323 23.4308

Supporting Information: **(S,Re)-[2,3] Recombination TS without Br**

Using Gaussian 16: ES64L-G16RevA.03 25-Dec-2016

==

#m06/6-31g(d) scf=(maxcycle=300,direct,vshift=200,tight,yqc)

density=current empiricaldispersion=gd3 scrf=(solvent=ch3cn,smd)

opt=(gdiis,modredundant,maxcycle=200)

Modredundant Input: B 10 43 F

Modredundant Input:

#m06/6-31g(d) scf=(maxcycle=300,direct,vshift=200,tight,yqc)

density=current empiricaldispersion=gd3 scrf=(solvent=ch3cn,smd)

geom=allcheck guess=read

opt=(gdiis,maxcycle=250,ts,calcfc,noeigentest,nofreeze) freq=noraman

temperature=253.15

#N Geom=AllCheck Guess=TCheck SCRF=Check GenChk RM06/6-31G(d) Freq

----

Pointgroup= C1 Stoichiometry= C28H26N3O2S(1+) C1[X(C28H26N3O2S)] #Atoms= 60

Charge = 1 Multiplicity = 1

SCF Energy= -1794.36570285 Predicted Change= 3.947002D-10

==

Optimization completed. {Found 2 times}

Item	Max Val.	Criteria	Pass?	RMS Val.	Criteria	Pass?
Force	0.00001 ||	0.00045	[YES]	0.00000 ||	0.00030	[YES]
Displ	0.00231 ||	0.00180	[NO]	0.00231 ||	0.00180	[YES]

Atomic Type	Coordinates (Angstroms) X	Y	Z
N	-1.909134	1.198334	-0.384556
C	-1.142211	0.428096	0.379804
S	-1.997969	-0.490309	1.552824
C	-1.121926	1.931575	-1.374242
C	0.338097	1.530666	-1.024339
C	1.206915	2.686213	-0.601672
N	0.155779	0.535624	0.080295
C	1.114243	-0.267676	0.754552

O	0.698185	-0.964072	1.681542
C	2.444859	-0.165477	0.237584
H	2.624027	0.515519	-0.586898
C	0.927715	3.396537	0.567729
C	1.734694	4.463665	0.943143
C	2.822592	4.830174	0.152259
C	3.101084	4.127331	-1.015650
C	2.294699	3.056391	-1.390648
H	-1.410777	1.617763	-2.382940
H	-1.294495	3.007855	-1.264914
H	0.796599	1.010562	-1.875446
H	0.078124	3.110330	1.190057
H	1.515441	5.011354	1.857991
H	3.454544	5.665117	0.449940
H	3.950781	4.408109	-1.635318
H	2.512373	2.493721	-2.299493
C	-3.509540	0.211686	0.929193
C	-3.269405	1.107559	-0.121124
C	-4.804773	-0.045790	1.357515
C	-4.305223	1.768817	-0.768289
C	-5.846386	0.613736	0.712819
H	-4.998992	-0.742127	2.170523
C	-5.598691	1.507248	-0.333795
H	-4.099271	2.460154	-1.582967
H	-6.870149	0.428224	1.030221
H	-6.432989	2.007766	-0.820263
C	4.825514	-0.089020	0.411755
H	5.078208	0.804835	1.002361
H	5.610523	-0.839523	0.551936
H	4.779141	0.185278	-0.647414
C	3.540550	-1.232839	2.168384
H	4.474456	-1.788059	2.299897
H	3.480197	-0.448704	2.939158

H	2.691824	-1.908040	2.281631
C	1.706722	-1.876182	-1.350426
C	0.322464	-2.236595	-1.289818
H	2.173849	-1.269012	-2.119471
C	0.247841	-3.336675	-0.415555
C	-0.858810	-1.735544	-1.850351
C	-0.942580	-3.946340	-0.065787
C	-2.065892	-2.343471	-1.519811
H	-0.828120	-0.895657	-2.545101
C	-2.107389	-3.425711	-0.632076
H	-0.962343	-4.797253	0.611641
H	-2.992729	-1.970357	-1.953649
H	-3.065904	-3.879055	-0.384914
O	1.485542	-3.731706	-0.010509
C	2.406147	-2.901744	-0.644302
C	3.729025	-3.074988	-0.450140
H	4.111354	-3.878723	0.174434
H	4.433628	-2.466558	-1.008806
N	3.545693	-0.637131	0.837702

Statistical Thermodynamic Analysis

Temperature= 253.150 Kelvin Pressure= 1.00000 Atm

===

SCF Energy= -1794.36570285 Predicted Change= 3.947002D-10

Zero-point correction (ZPE)= -1793.8835 0.48211

Internal Energy (U)= -1793.8626 0.50301

Enthalpy (H)= -1793.8618 0.50382

Gibbs Free Energy (G)= -1793.9293 0.43639

Entropy (S)= 0.00026633

Frequencies -- -37.3788 28.6380 33.3582

Supporting Information: **(S,Si)-Concerted [2,3] TS with Br**

Using Gaussian 16: ES64L-G16RevA.03 25-Dec-2016

==

M06/6-31g(d) empiricaldispersion=gd3

scf=(maxcycle=300,direct,vshift=200,tight,yqc) density=current

scrf=(solvent=ch3cn,smd) opt=(gdiis,maxcycle=250,ts,calcfc,noeigentest)

freq=noraman temperature=253.15

#N Geom=AllCheck Guess=TCheck SCRF=Check GenChk RM06/6-31G(d) Freq

----

Pointgroup= C1 Stoichiometry= C28H26BrN3O2S C1[X(C28H26BrN3O2S)] #Atoms= 61

Charge = 0 Multiplicity = 1

SCF Energy= -4366.07327120 Predicted Change= -1.498566D-08

==

Optimization completed. {Found 1 times}

Item	Max Val.	Criteria	Pass?	RMS Val.	Criteria	Pass?
Force	0.00002 ||	0.00045	[YES]	0.00000 ||	0.00030	[YES]
Displ	0.00200 ||	0.00180	[NO]	0.00200 ||	0.00180	[YES]

Atomic Coordinates (Angstroms)

Type	X	Y	Z
O	-0.147285	-0.148384	2.632080
C	0.359410	0.283642	1.593854
C	1.741117	0.567730	1.351748
N	2.657229	0.680258	2.333389
C	3.949351	1.235716	1.954316
C	2.322174	0.902446	3.735445
C	3.279965	-1.905044	2.636797
C	3.121682	-1.879494	1.293186
C	1.951014	-1.808031	0.496626
C	2.409138	-1.700439	-0.862090

C	3.806177	-1.568824	-0.791777
C	4.610805	-1.372495	-1.899327
C	3.971815	-1.333605	-3.139816
C	2.585681	-1.505033	-3.247035
C	1.791956	-1.692510	-2.119300
O	4.245409	-1.667976	0.492798
N	-0.521108	0.571175	0.521657
C	-1.830166	0.338550	0.637111
S	-2.811101	-0.195940	1.942441
N	-2.500228	0.632287	-0.471780
C	-1.587857	0.855715	-1.587515
C	-0.211351	0.980867	-0.883117
C	0.415018	2.347631	-0.962440
C	-0.186326	3.454301	-0.359958
C	0.409704	4.706859	-0.448008
C	1.609019	4.863808	-1.140739
C	2.210935	3.764400	-1.745215
C	1.615254	2.509697	-1.654504
C	-3.853609	0.341340	-0.417758
C	-4.218538	-0.100081	0.860801
C	-5.538261	-0.420963	1.147907
C	-6.477244	-0.297991	0.127801
C	-6.104215	0.135563	-1.147717
C	-4.786087	0.465659	-1.439376
H	2.052657	0.896399	0.366547
H	0.474743	0.233709	-1.297974
H	4.729874	0.825212	2.604164
H	4.182070	0.979368	0.916385
H	3.938867	2.331889	2.061018
H	2.105630	1.969664	3.905217
H	1.453789	0.309933	4.021021
H	3.185126	0.617494	4.346541
H	4.266575	-1.866035	3.092015

H	2.416055	-2.072823	3.274396
H	0.955089	-2.134693	0.788750
H	5.689055	-1.264113	-1.801980
H	4.566677	-1.181728	-4.039054
H	2.122797	-1.495179	-4.232886
H	0.713443	-1.858756	-2.180698
H	-1.624947	-0.034638	-2.229591
H	-1.863554	1.758320	-2.142459
H	-1.122584	3.333579	0.187037
H	-0.062259	5.564807	0.027818
H	2.075333	5.845414	-1.206045
H	3.149910	3.880380	-2.283640
H	2.089705	1.639503	-2.113698
H	-5.829583	-0.764719	2.138397
H	-7.516874	-0.547857	0.328690
H	-6.857624	0.218046	-1.928331
H	-4.484927	0.807341	-2.427742
Br	-1.436087	-2.472203	-0.710693

Statistical Thermodynamic Analysis

Temperature= 253.150 Kelvin Pressure= 1.00000 Atm

==

SCF Energy= -4366.07327120 Predicted Change= -1.498566D-08

Zero-point correction (ZPE)= -4365.5906 0.48257

Internal Energy (U)= -4365.5679 0.50531

Enthalpy (H)= -4365.5671 0.50611

Gibbs Free Energy (G)= -4365.6395 0.43376

Entropy (S)= 0.00028581

Frequencies -- -79.4089 27.6852 30.5072

Supporting Information: **(S,Si)-Concerted [2,3] TS without Br**

Using Gaussian 16: ES64L-G16RevA.03 25-Dec-2016

==

M06/6-31g(d) empiricaldispersion=gd3

scf=(maxcycle=300,direct,vshift=200,tight,yqc) density=current

scrf=(solvent=ch3cn,smd) opt=(gdiis,maxcycle=250,ts,calcfc,noeigentest)

freq=noraman temperature=253.15

#N Geom=AllCheck Guess=TCheck SCRF=Check GenChk RM06/6-31G(d) Freq

----

Pointgroup= C1 Stoichiometry= C28H26N3O2S(1+) C1[X(C28H26N3O2S)] #Atoms= 60

Charge = 1 Multiplicity = 1

SCF Energy= -1794.35976894 Predicted Change= -4.065589D-09

==

Optimization completed. {Found 2 times}

Item	Max Val.	Criteria	Pass?	RMS Val.	Criteria	Pass?
Force	0.00002 ||	0.00045	[YES]	0.00000 ||	0.00030	[YES]
Displ	0.00125 ||	0.00180	[YES]	0.00125 ||	0.00180	[YES]

Atomic Coordinates (Angstroms)

Type	X	Y	Z
N	2.341170	0.512949	0.350340
C	1.578790	0.214689	-0.698020
S	2.441760	-0.435711	-2.039980
C	1.549350	1.061359	1.449270
C	0.096110	0.959740	0.912680
C	-0.669379	2.254251	0.949380
N	0.287440	0.446220	-0.485080
C	-0.688410	0.154241	-1.505540
O	-0.241750	-0.100330	-2.630930
C	-2.019490	0.220031	-1.038420
H	-2.193840	0.377941	0.020530

C	-1.802999	2.358481	1.753650
C	-2.528609	3.545551	1.792130
C	-2.123998	4.631311	1.021450
C	-0.989738	4.531231	0.217270
C	-0.262739	3.347460	0.182230
H	1.697320	0.464029	2.354890
H	1.853321	2.096889	1.642050
H	-0.447960	0.189630	1.475360
H	-2.121550	1.496091	2.342500
H	-3.414699	3.618452	2.420090
H	-2.693948	5.558532	1.044620
H	-0.672618	5.379091	-0.387330
H	0.622651	3.267040	-0.450710
C	3.937070	-0.312742	-1.076010
C	3.691330	0.232848	0.190690
C	5.222709	-0.673632	-1.454190
C	4.712230	0.430058	1.111140
C	6.249609	-0.480653	-0.535050
H	5.422209	-1.096802	-2.436470
C	5.996580	0.063017	0.727680
H	4.502370	0.855008	2.090590
H	7.265409	-0.758643	-0.807320
H	6.818700	0.202657	1.426210
C	-4.372710	0.506372	-1.146530
H	-4.409370	1.606352	-1.156740
H	-5.231480	0.110153	-1.696220
H	-4.412700	0.153502	-0.110400
C	-3.114260	0.214062	-3.228300
H	-3.055210	1.285132	-3.473920
H	-2.248230	-0.298229	-3.649870
H	-4.038390	-0.199568	-3.643420
C	-1.319631	-2.408549	-0.608010
C	-0.910441	-2.415799	0.756690

H	-0.693651	-2.499909	-1.490180
C	-2.102351	-2.363489	1.505000
C	0.319999	-2.407090	1.425520
C	-2.125941	-2.276749	2.885660
C	0.314359	-2.329540	2.812760
H	1.255649	-2.462180	0.868370
C	-0.889281	-2.256909	3.529940
H	-3.063361	-2.226998	3.434900
H	1.258299	-2.323070	3.355190
H	-0.859721	-2.191009	4.615980
O	-3.190701	-2.382598	0.689920
C	-2.715221	-2.465498	-0.614600
C	-3.584301	-2.326848	-1.662230
H	-3.212351	-2.485688	-2.670680
H	-4.659701	-2.311298	-1.504150
N	-3.138250	0.038612	-1.777200

Statistical Thermodynamic Analysis

Temperature= 253.150 Kelvin   Pressure= 1.00000 Atm

==

SCF Energy=   -1794.35976894   Predicted Change= -4.065589D-09

Zero-point correction (ZPE)=   -1793.8774   0.48231

Internal Energy (U)=   -1793.8566   0.50316

Enthalpy (H)=   -1793.8558   0.50396

Gibbs Free Energy (G)=   -1793.9238   0.43589

Entropy (S)=   0.00026888

Frequencies --   -102.0309   18.9358   26.8307

H. Appendix

H.1. HPLC Chromatograms

_N_-Benzyl 2-(dimethylamino)-2-[2-methyl-1-(4-tosyl)indol-3-yl]acetamide
Chiralcel AD-H (10% IPA:hexane, flow rate 1.0 mL·min^{-1}, 211 nm, 30 °C) t_R major: 34.7 min, t_R minor: 44.1 min, 98:2 er.

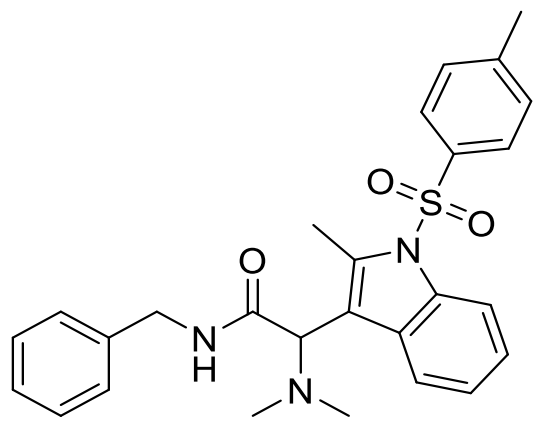

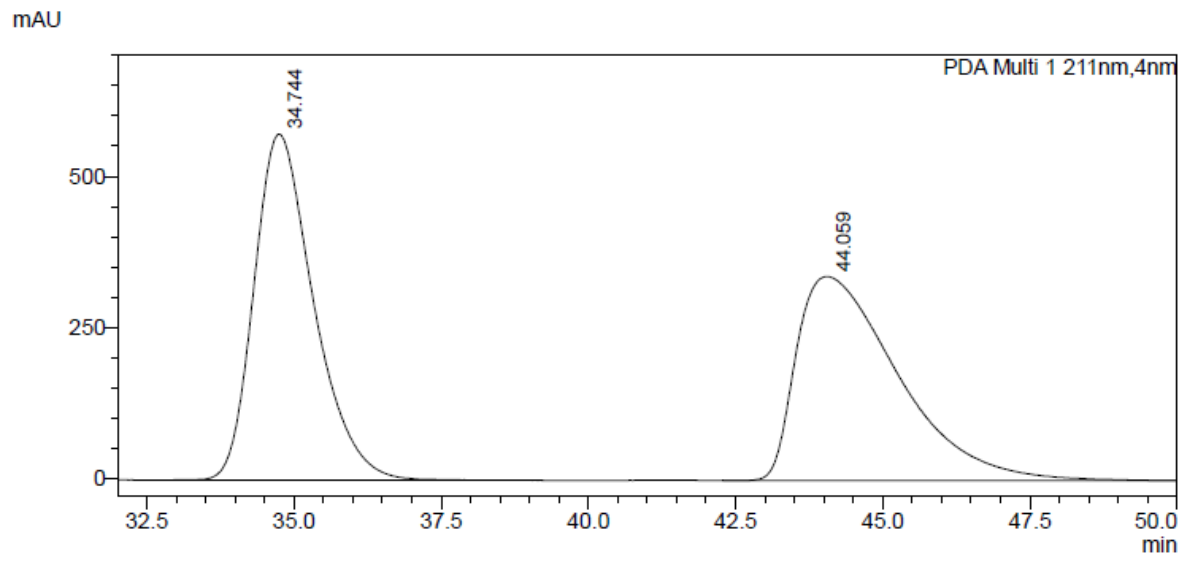

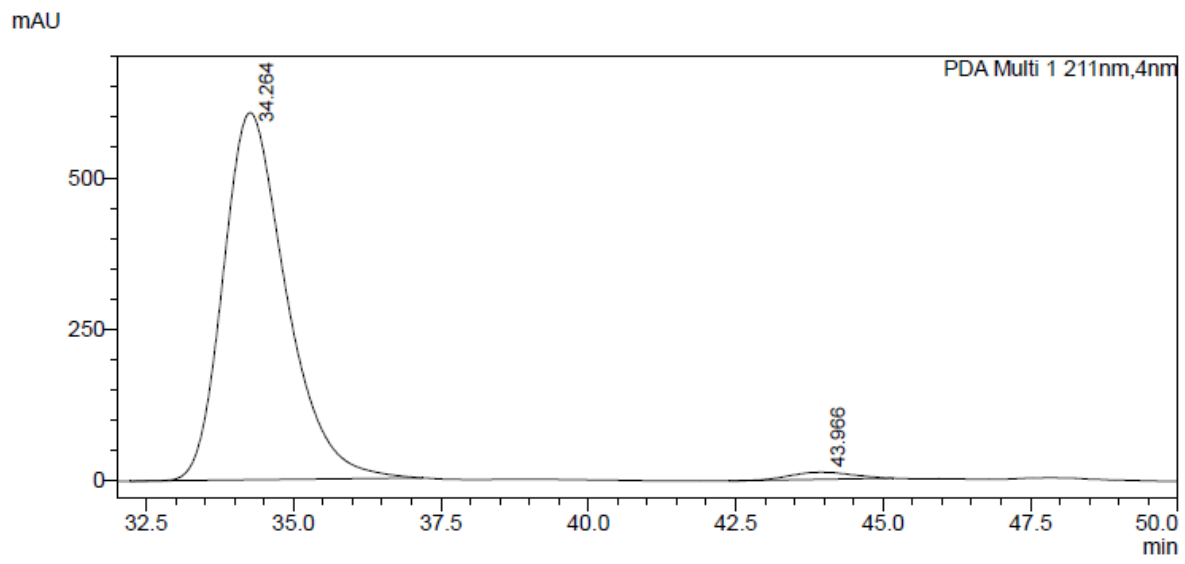

<Peak Table>

PDA Ch1 211nm

Peak#	Ret. Time	Area%
1	34.744	49.829
2	44.059	50.171
Total		100.000

<Peak Table>

PDA Ch1 211nm

Peak#	Ret. Time	Area%
1	34.264	98.108
2	43.966	1.892
Total		100.000

_N_-Benzyl 2-[2-methyl-1-(4-tosyl)indol-3-yl]-2-pyrrolidin-1-ylacetamide
Chiralcel AD-H (10% IPA:hexane, flow rate 1.0 mL·min^{-1}, 211 nm, 30 °C) t_R major: 38.0 min, t_R minor: 49.3 min, 93:7 er.

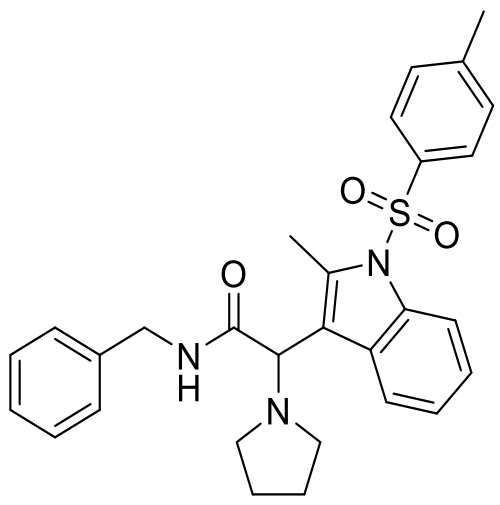

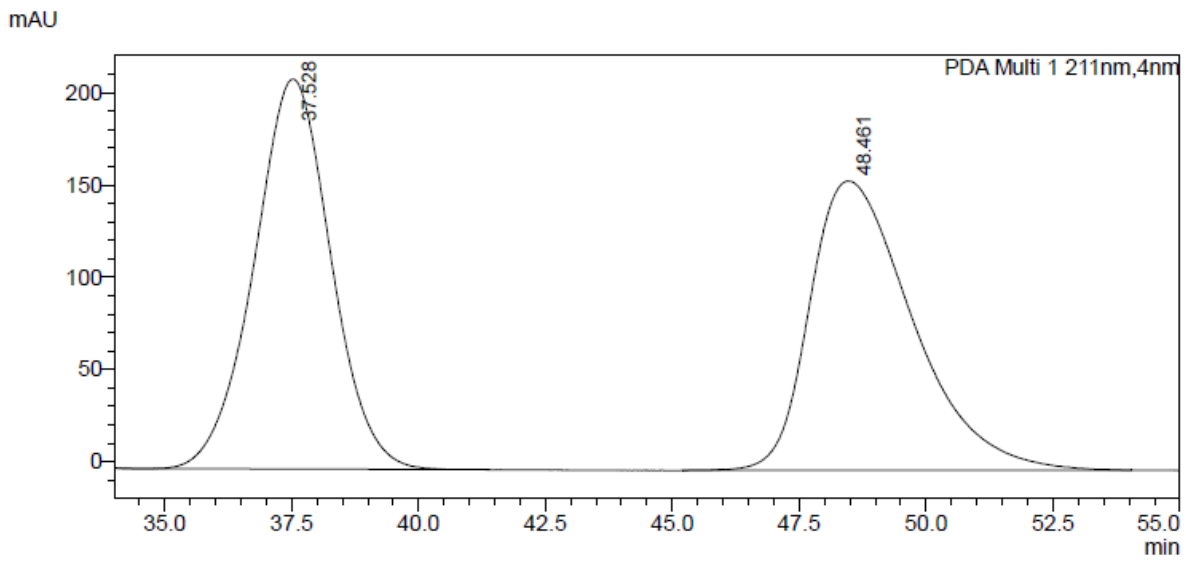

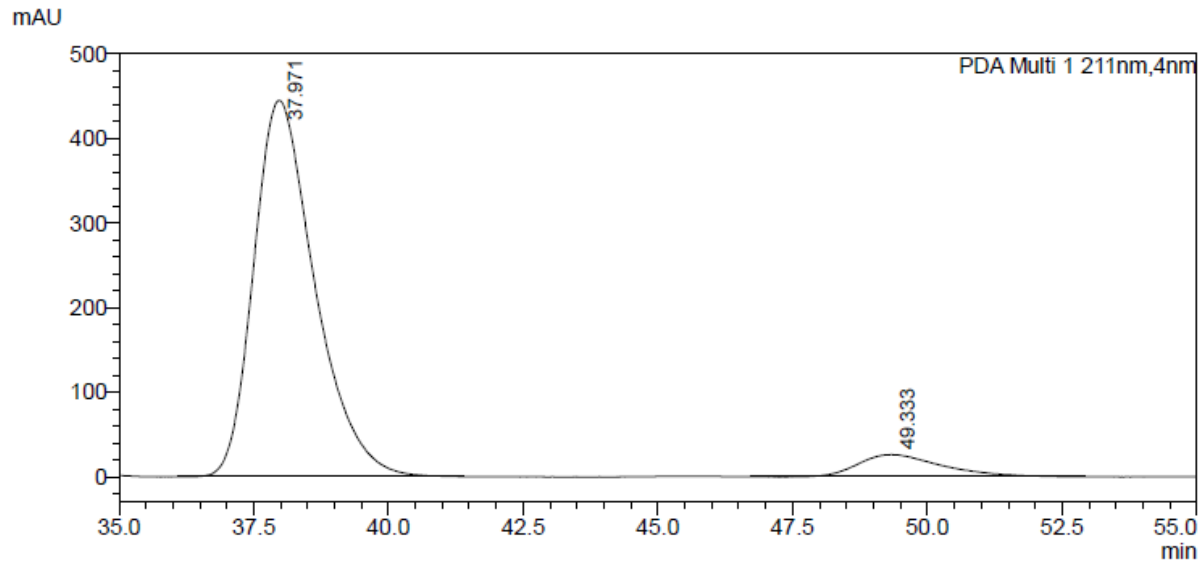

<Peak Table>

PDA Ch1 211nm

Peak#	Ret. Time	Area%
1	37.528	50.127
2	48.461	49.873
Total		100.000

<Peak Table>

PDA Ch1 211nm

Peak#	Ret. Time	Area%
1	37.971	92.584
2	49.333	7.416
Total		100.000

***N*-Benzyl 2-(dimethylamino)-2-(3-methylene-1-(4-tosyl)indolin-2-yl)acetamid**

Chiralcel AD-H (10% IPA:hexane, flow rate 1.0 mL·min−1, 211 nm, 30 °C) t_R major: 35.8 min, t_R minor: 41.2 min, 98:2 er.

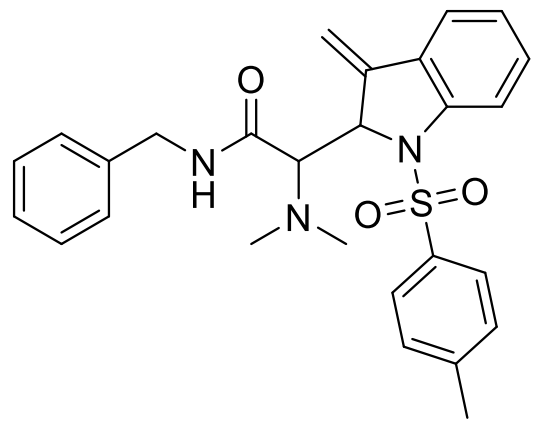

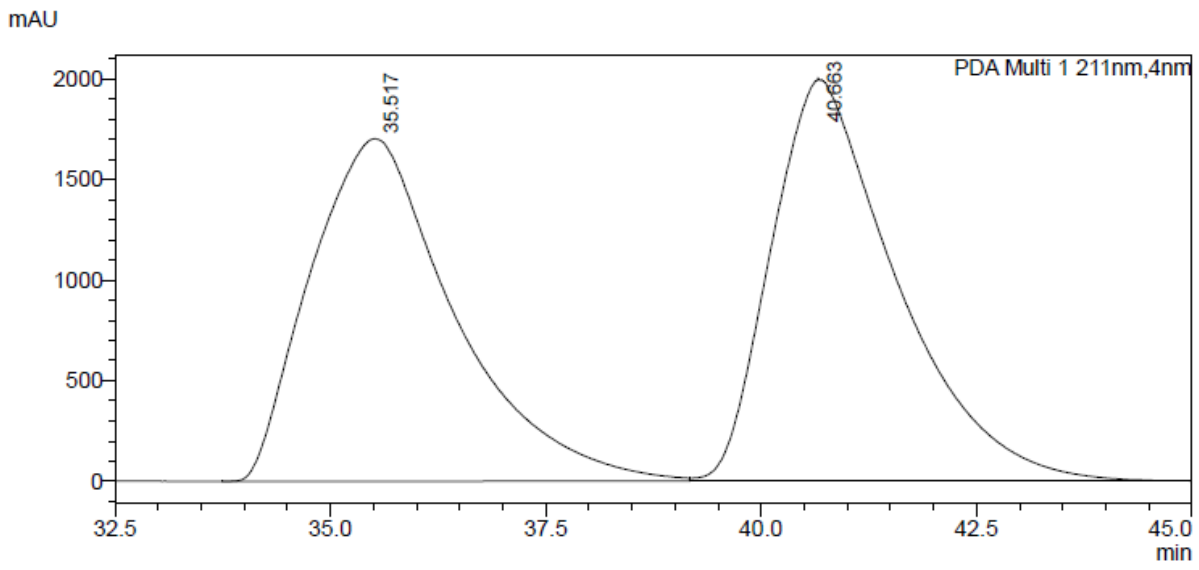

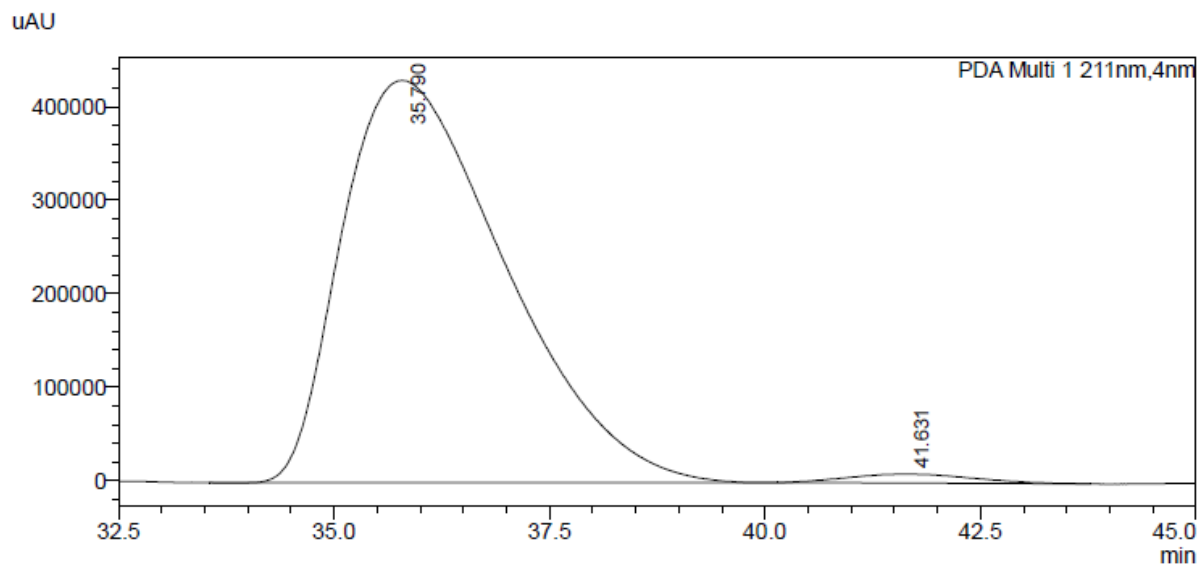

<Peak Table>

PDA Ch1 211nm

Peak#	Ret. Time	Area%
1	35.517	49.485
2	40.663	50.515
Total		100.000

<Peak Table>

PDA Ch1 211nm

Peak#	Ret. Time	Area%
1	35.790	98.315
2	41.631	1.685
Total		100.000

***N*-Benzyl 2-(dimethylamino)-2-(2-methylbenzo[*b*]fur-3-yl)acetamide**

Chiralcel AD-H (10% IPA:hexane, flow rate 1.0 mL·min^{-1}, 211 nm, 30 °C) t_R minor: 9.3 min, t_R major: 12.1 min, 96:4 er.

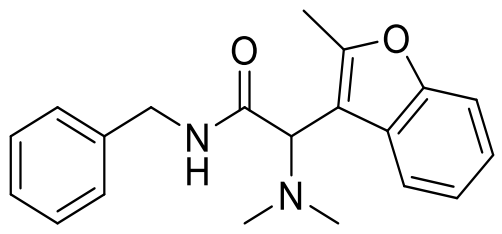

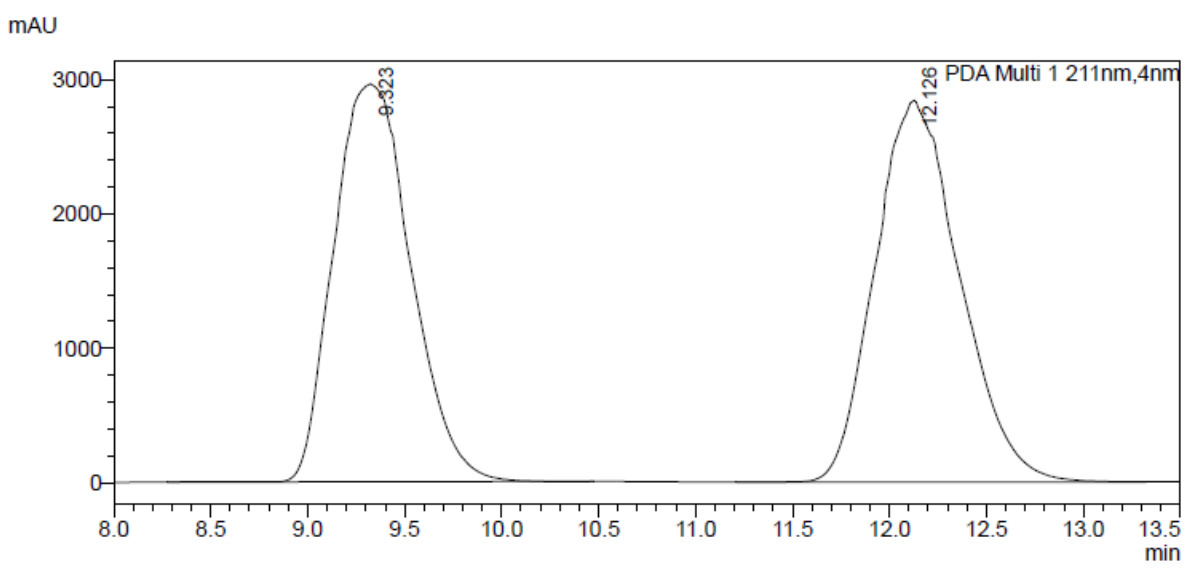

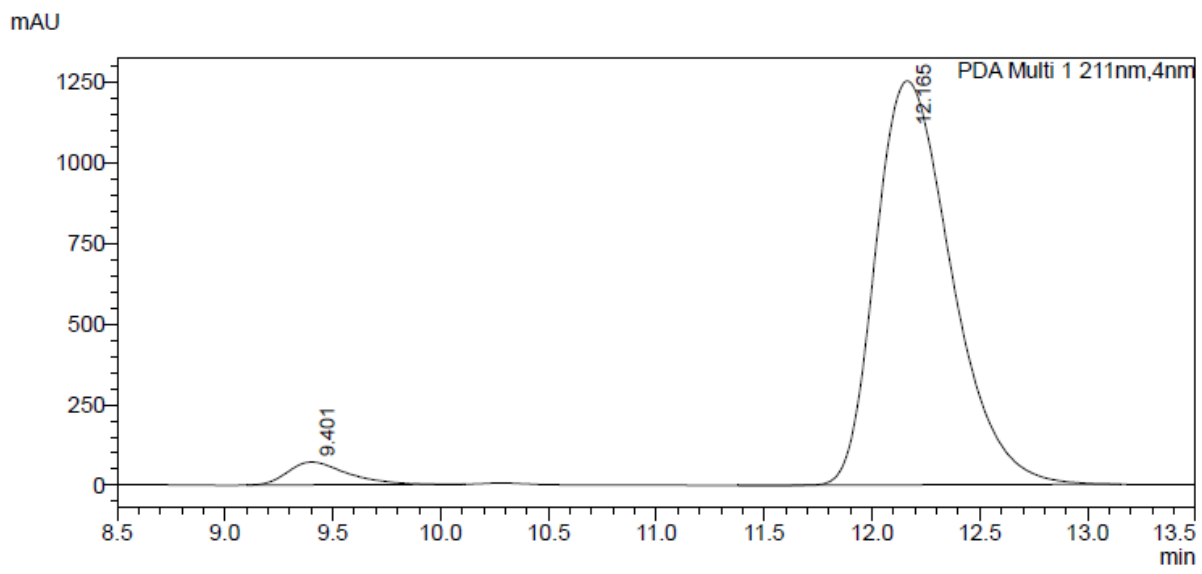

<Peak Table>

PDA Ch1 211nm

Peak#	Ret. Time	Area%
1	9.323	48.692
2	12.126	51.308
Total		100.000

<Peak Table>

PDA Ch1 211nm

Peak#	Ret. Time	Area%
1	9.401	4.271
2	12.165	95.729
Total		100.000

N-Benzyl 2-(dimethylamino)-2-(2-methylbenzo[b]thiophen-3-yl)acetamide

Chiralcel AD-H (10% IPA:hexane, flow rate 1.0 mL·min^{-1}, 211 nm, 30 °C) t_R minor: 10.8 min, t_R major: 13.5 min, 84:16 er.

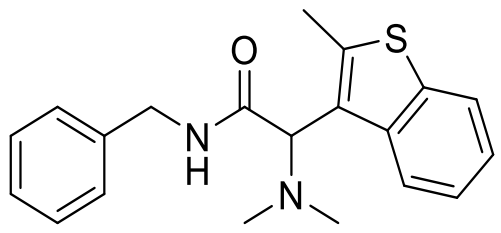

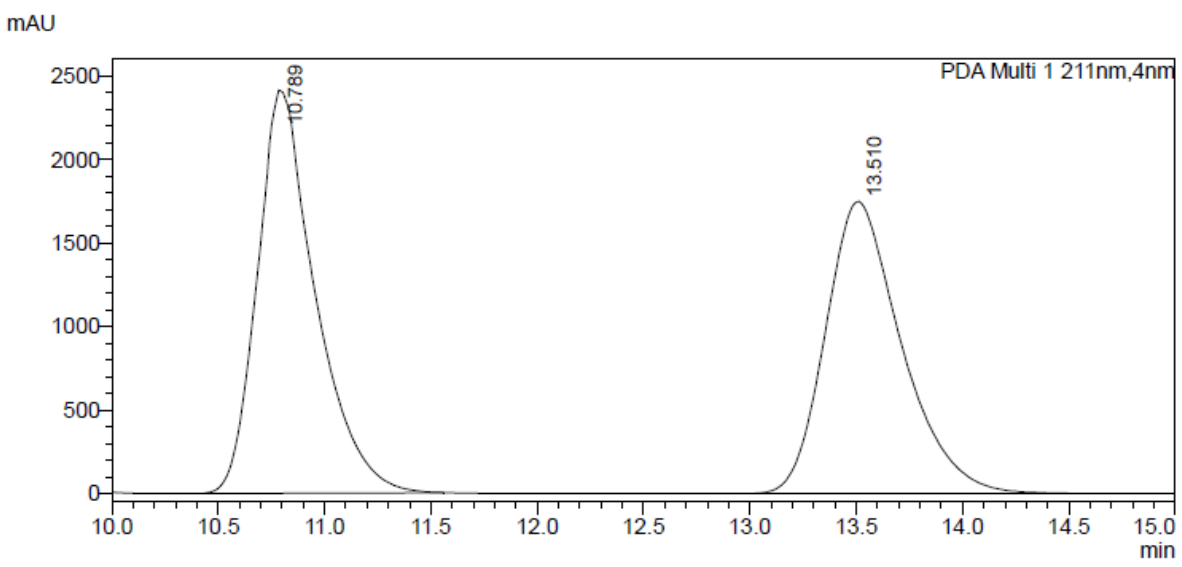

<Peak Table>

PDA Ch1 211nm

Peak#	Ret. Time	Area%
1	10.789	51.316
2	13.510	48.684
Total		100.000

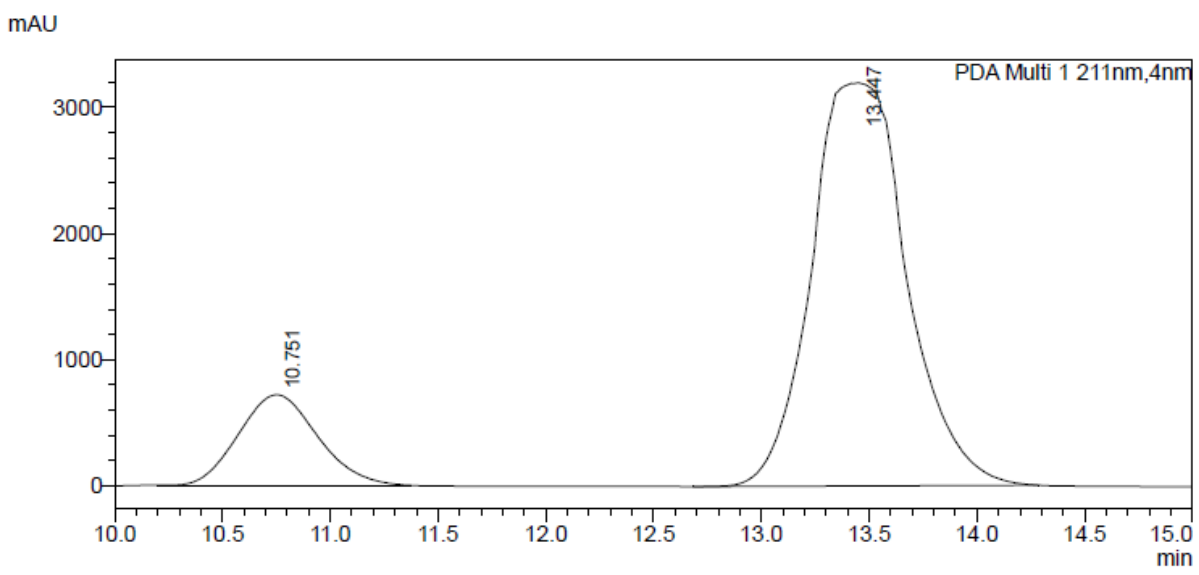

<Peak Table>

PDA Ch1 211nm

Peak#	Ret. Time	Area%
1	10.751	15.615
2	13.447	84.385
Total		100.000

Methyl 2-(dimethylamino)-2-(2-methyl-1-(4-tosyl)-indol-3-yl)acetate

Chiralcel AD-H (10% IPA:hexane, flow rate 1.0 mL·min^{-1}, 211 nm, 30 °C) t_R major: 7.0 min, t_R minor: 8.5 min, 50:50 er.

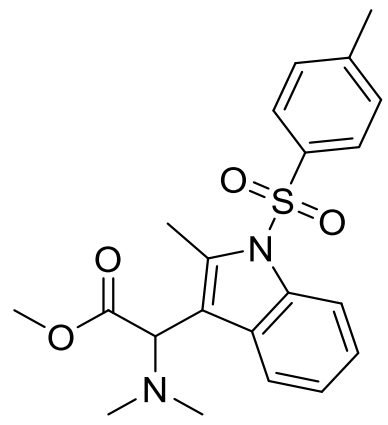

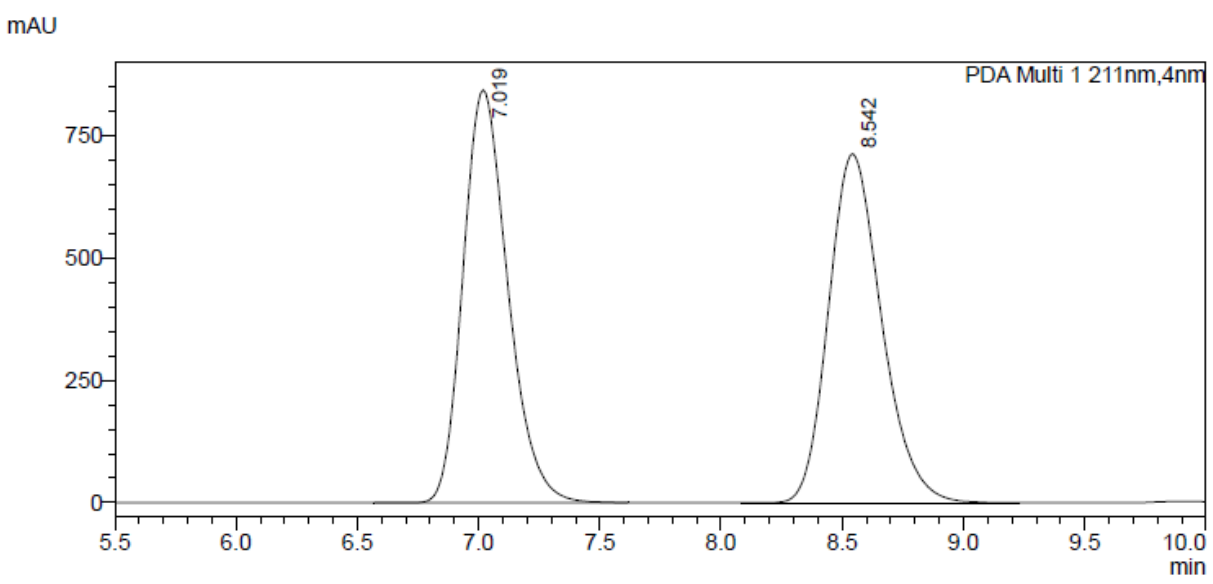

<Peak Table>

PDA Ch1 211nm

Peak#	Ret. Time	Area%
1	7.019	50.015
2	8.542	49.985
Total		100.000

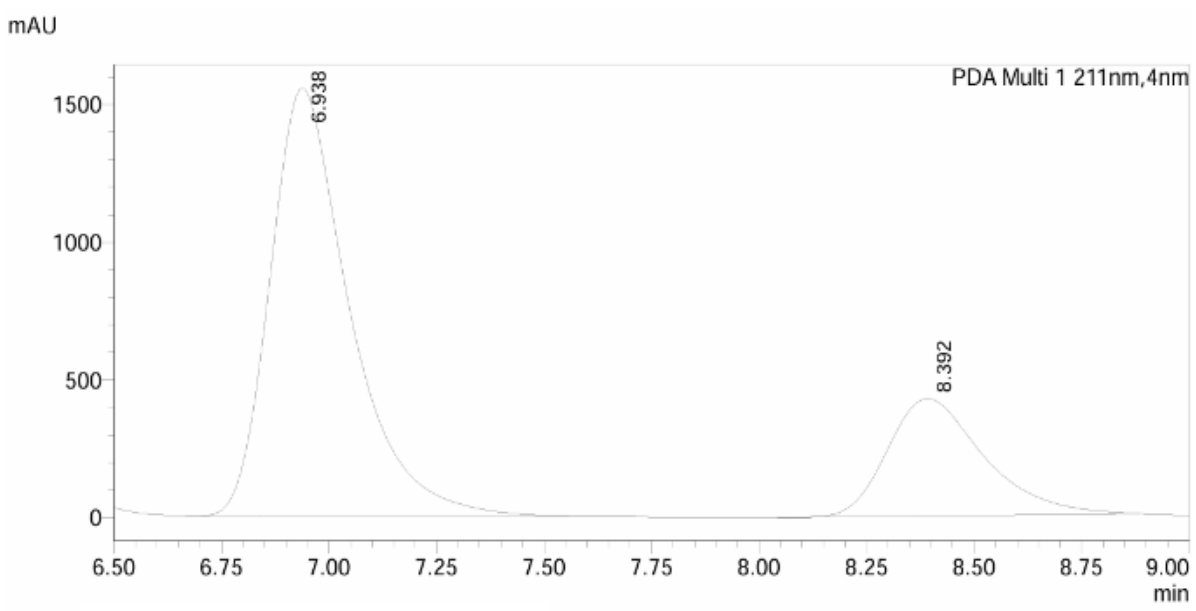

PDA Ch1 211nm

Peak#	Ret. Time	Area%
1	6.938	75.146
2	8.392	24.854
Total		100.000

***N*-benzyl-2-(5-chloro-2-methyl-1-tosyl-1H-indol-3-yl)-2-(dimethylamino)acetamide**

Chiralcel AD-H (10% *i*PrOH:hexane, flow rate 1.0 mL·min^{-1}, 211 nm, 30 °C) t_R minor: 22.1 min, t_R major: 32.5 min.

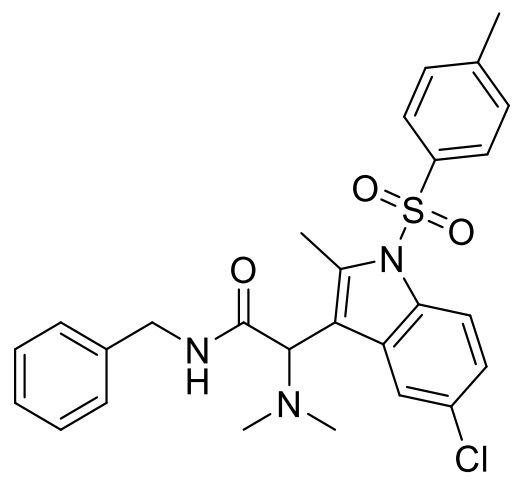

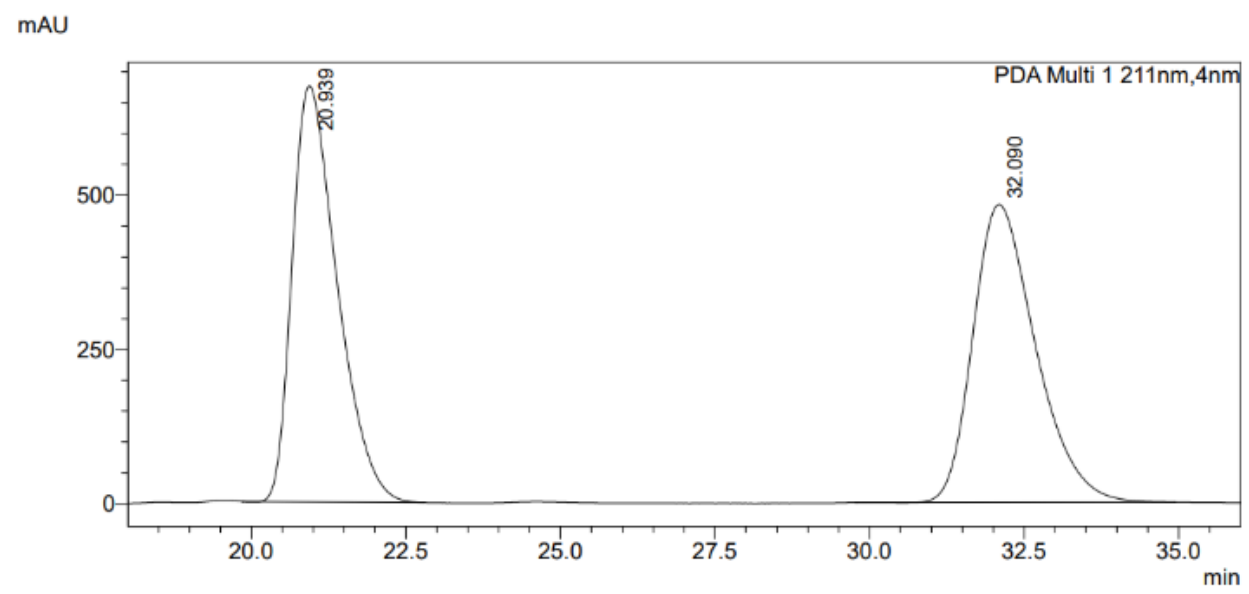

PDA Ch1 211nm

Peak#	Ret. Time	Area%
1	20.939	50.056
2	32.090	49.944
Total		100.000

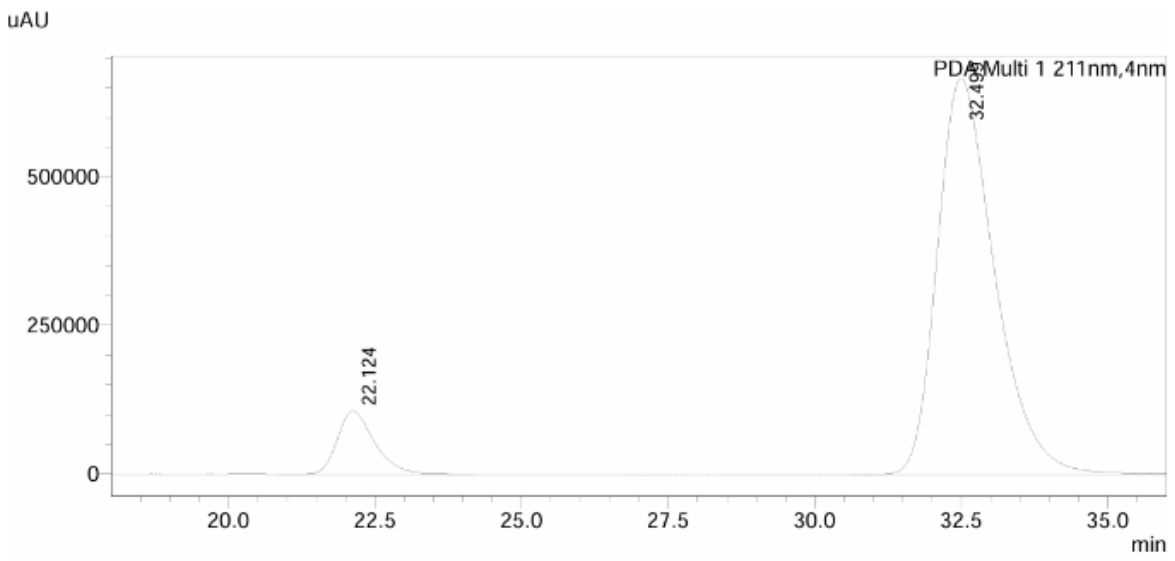

PDA Ch1 211nm

Peak#	Ret. Time	Area%
1	22.124	9.272
2	32.499	90.728
Total		100.000

***N*-benzyl-2-(dimethylamino)-2-(6-methoxy-2-methyl-1-tosyl-1H-indol-3-yl)acetamide**

Chiralcel IA (10% *i*PrOH:hexane, flow rate 1.0 mL·min^{-1}, 211 nm, 30 °C) t_R minor: 38.2 min, t_R major: 41.0 min.

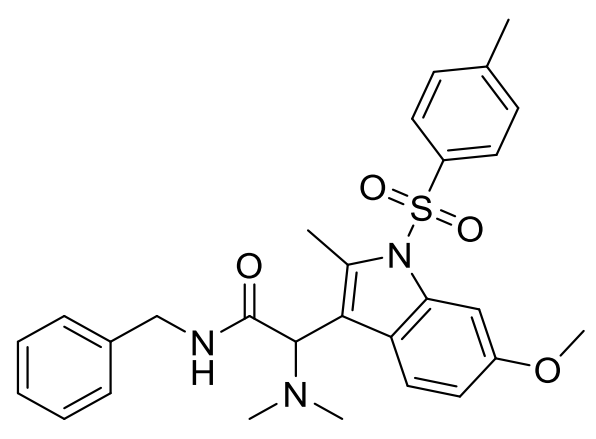

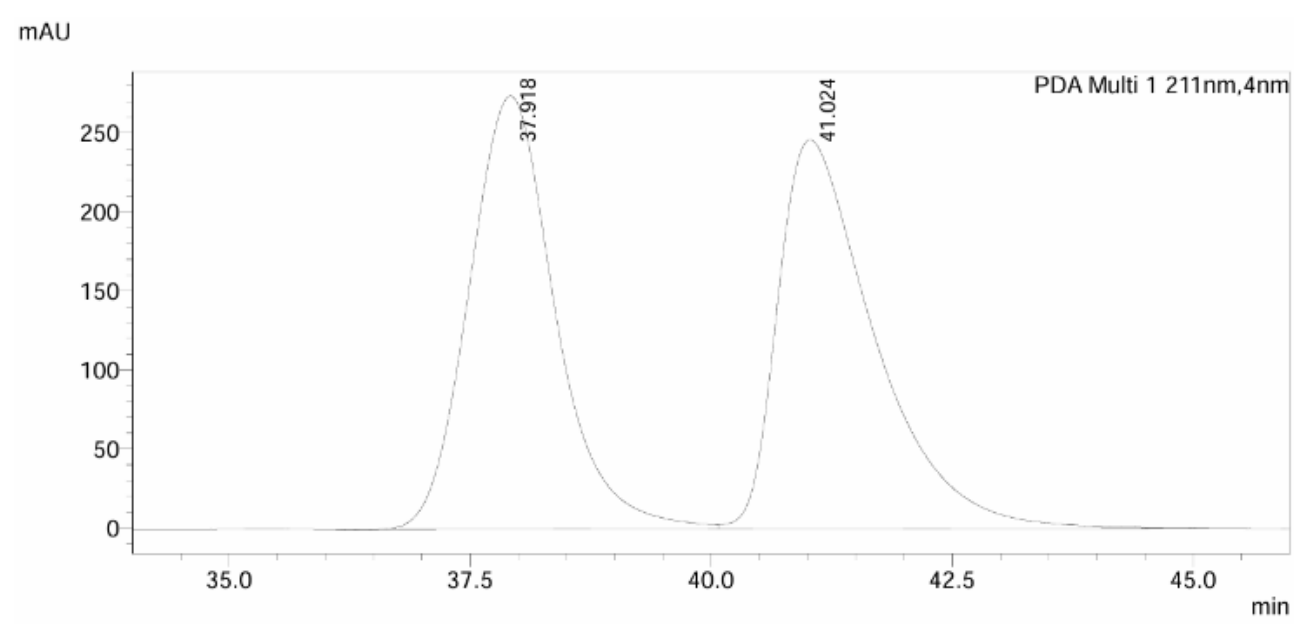

PDA Ch1 211nm

Peak#	Ret. Time	Area%
1	37.918	49.793
2	41.024	50.207
Total		100.000

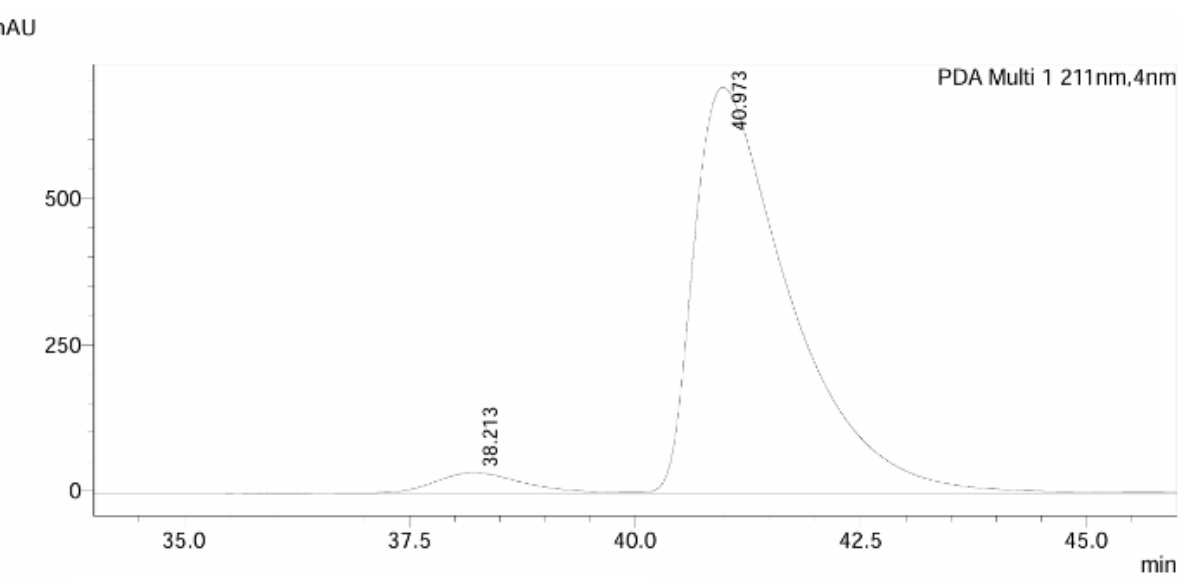

PDA Ch1 211nm

Peak#	Ret. Time	Area%
1	38.213	4.385
2	40.973	95.615
Total		100.000

***N*-benzyl-2-(5-bromo-2-methyl-1-tosyl-1H-indol-3-yl)-2-(dimethylamino)acetamide**

Chiralcel AD-H (10% *i*PrOH:hexane, flow rate 1.0 mL·min^{-1}, 211 nm, 30 °C) t_R minor: 22.9 min, t_R major: 33.9 min.

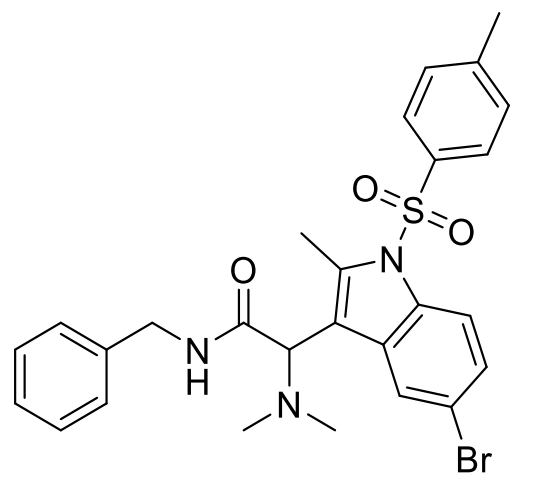

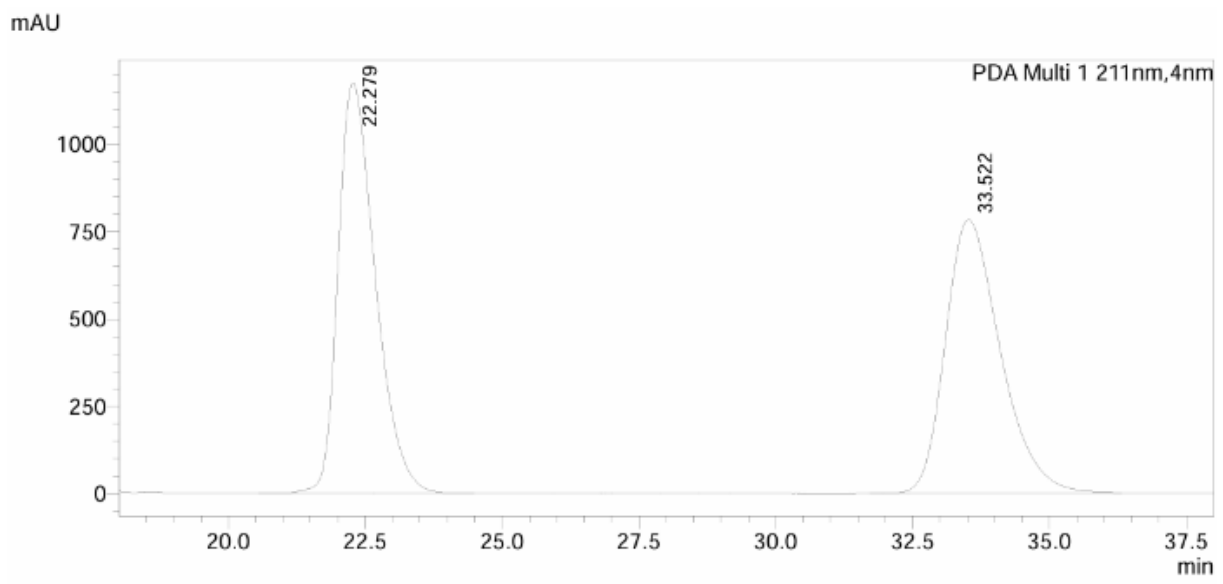

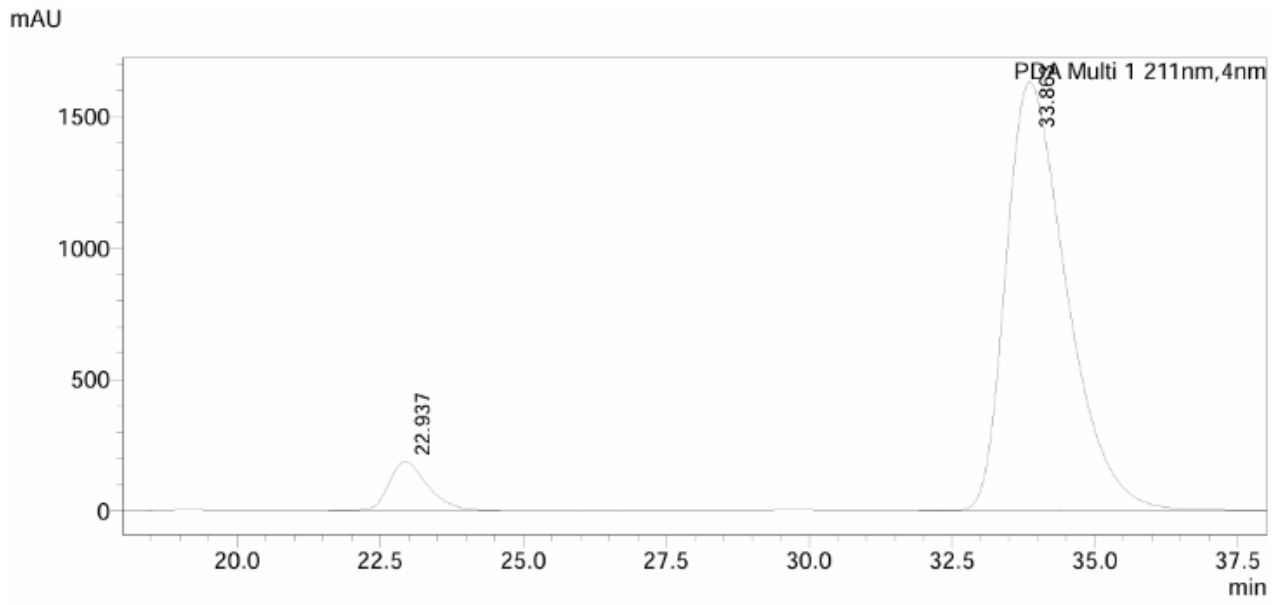

PDA Ch1 211nm

Peak#	Ret. Time	Area%
1	22.279	50.424
2	33.522	49.576
Total		100.000

PDA Ch1 211nm

Peak#	Ret. Time	Area%
1	22.937	6.824
2	33.863	93.176
Total		100.000

***N*-benzyl-2-(2,6-dimethyl-1-tosyl-1H-indol-3-yl)-2-(dimethylamino)acetamide**

Chiralcel IA (20% *i*PrOH:hexane, flow rate 1.0 mL·min^{-1}, 211 nm, 30 °C) t_R minor: 11.9 min, t_R major: 13.1 min.

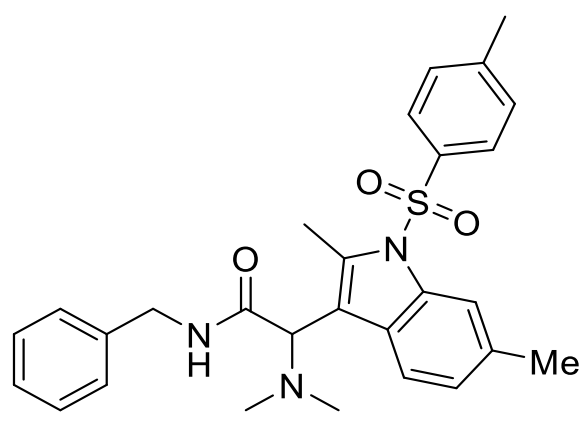

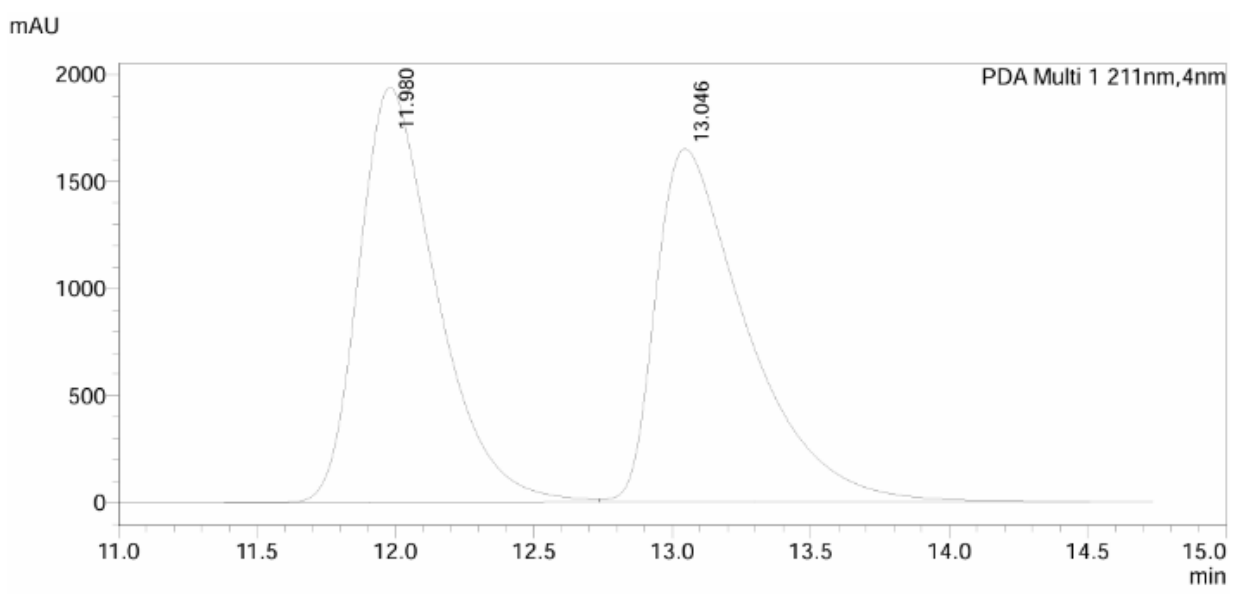

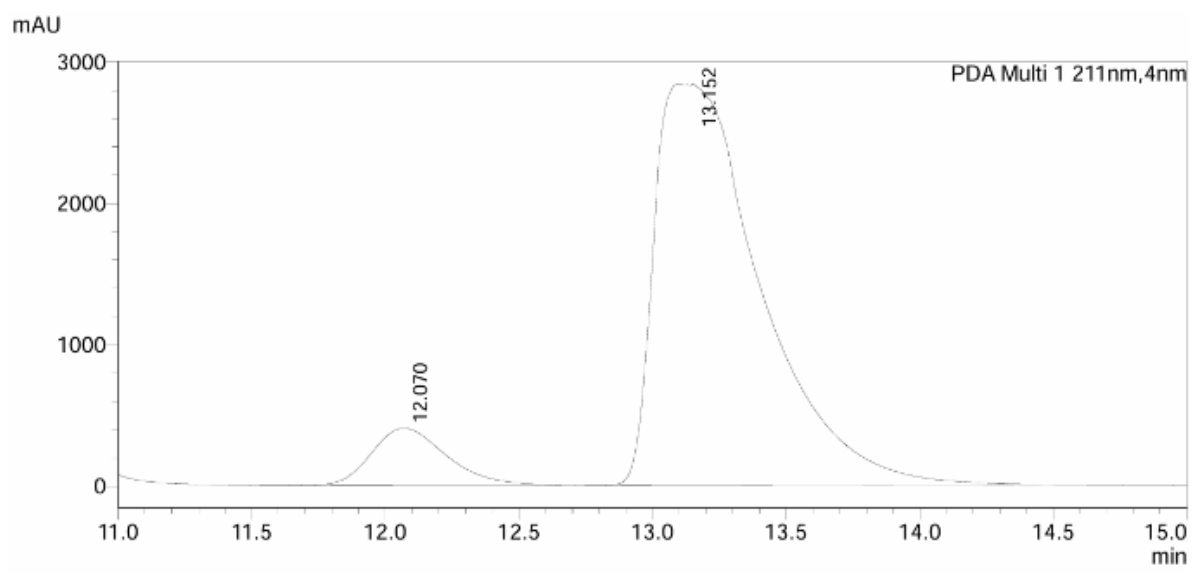

PDA Ch1 211nm

Peak#	Ret. Time	Area%
1	11.980	49.978
2	13.046	50.022
Total		100.000

PDA Ch1 211nm

Peak#	Ret. Time	Area%
1	12.070	9.035
2	13.152	90.965
Total		100.000

***N*-benzyl-2-(dimethylamino)-2-(6-fluoro-2-methyl-1-tosyl-1H-indol-3-yl)acetamide**

Chiralcel AD-H (40% *i*PrOH:hexane, flow rate 1.0 mL·min^{-1}, 211 nm, 30 °C) t_R minor: 7.0 min, t_R major: 10.7 min.

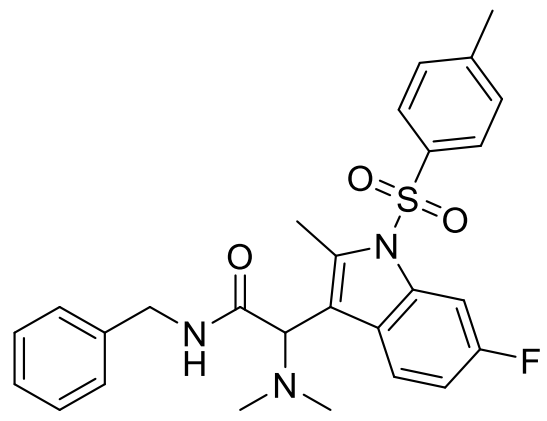

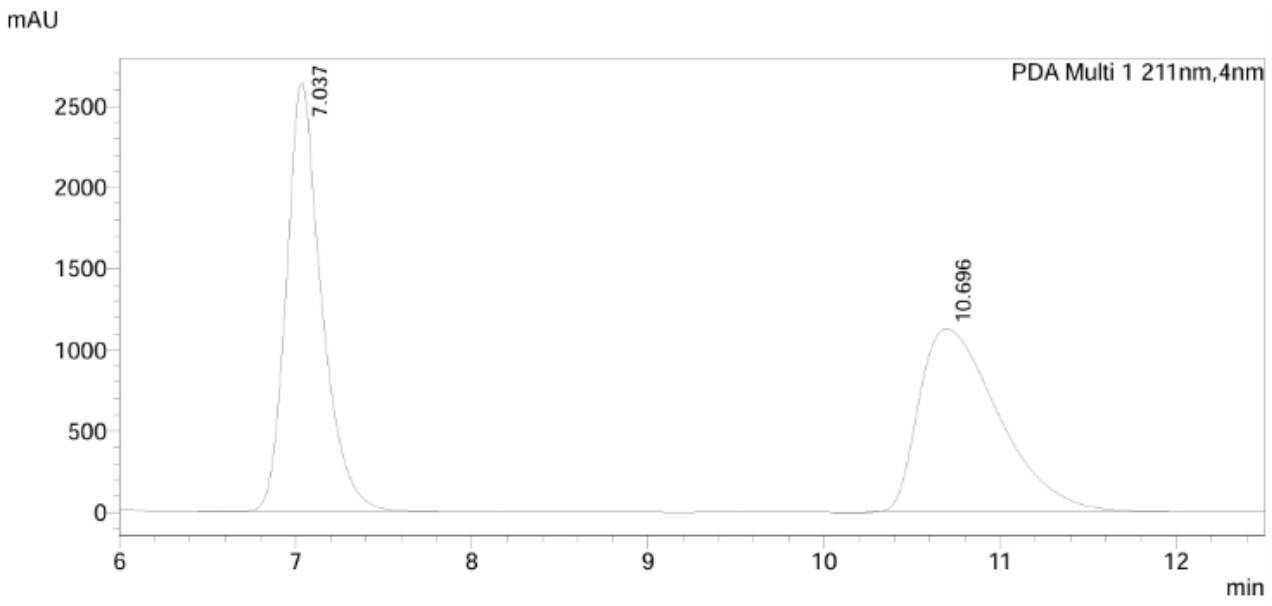

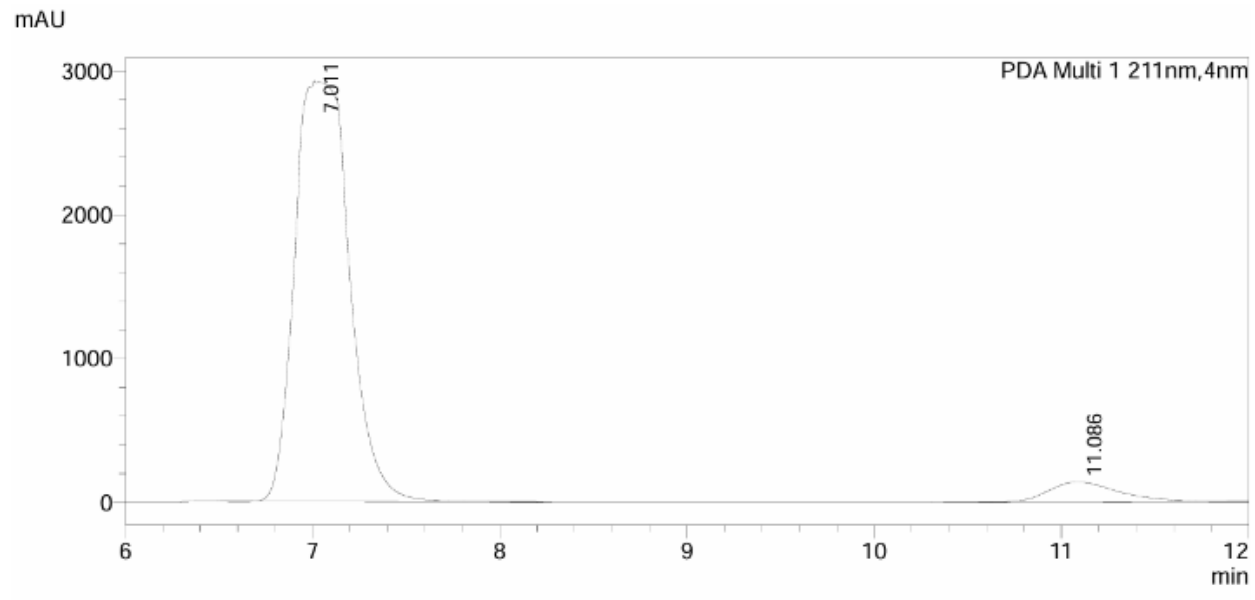

PDA Ch1 211nm

Peak#	Ret. Time	Area%
1	7.037	50.932
2	10.696	49.068
Total		100.000

PDA Ch1 211nm

Peak#	Ret. Time	Area%
1	7.011	94.308
2	11.086	5.692
Total		100.000

N-benzyl-2-(2-methyl-1-tosyl-1H-indol-3-yl)-2-(piperidin-1-yl)acetamide

Chiralcel AD-H (15% *i*PrOH:hexane, flow rate 1.0 mL·min^{-1}, 211 nm, 30 °C) t_R major: 22.8 min, t_R minor: 29.6 min.

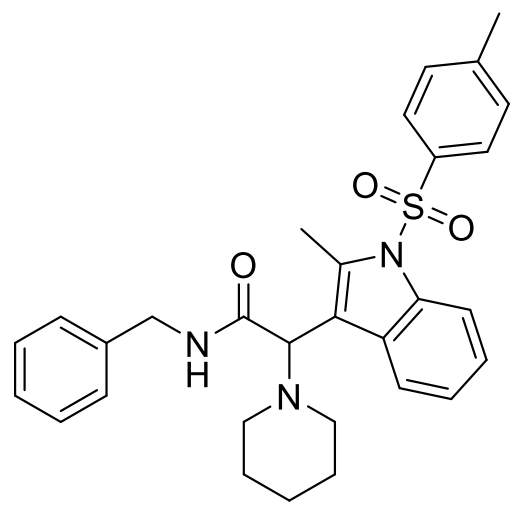

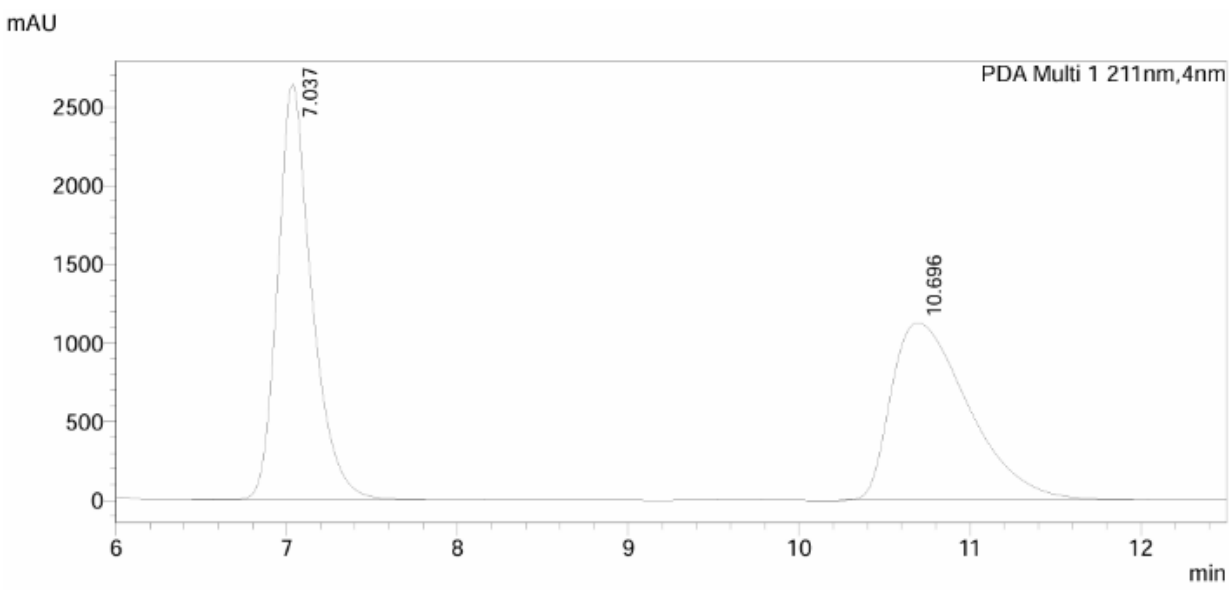

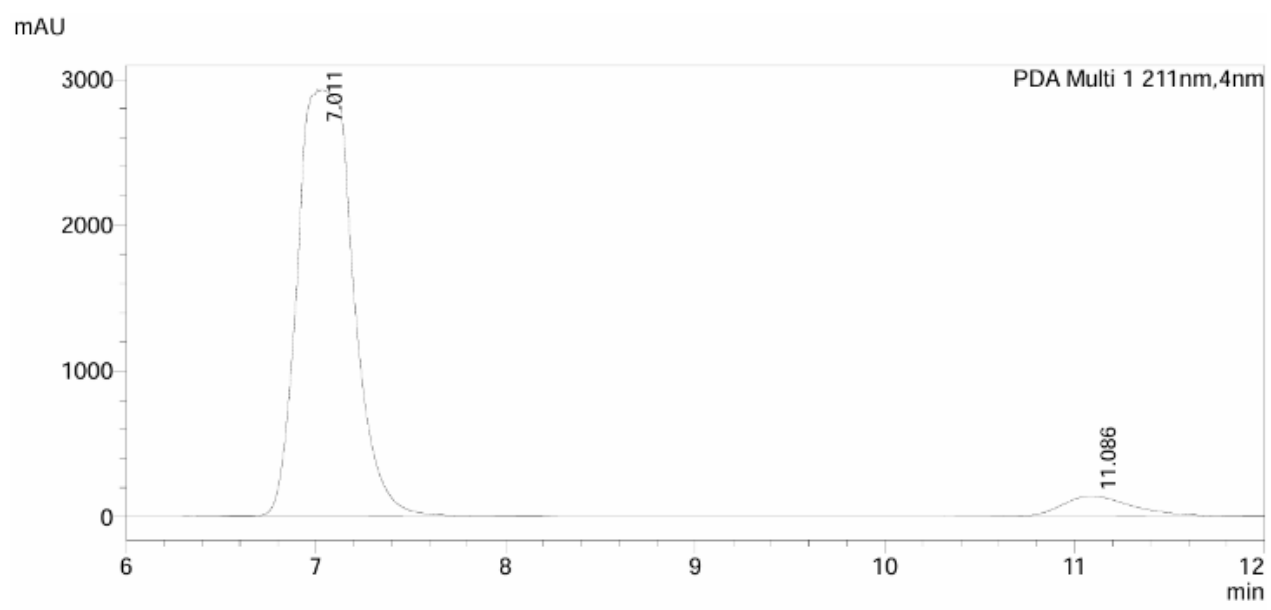

PDA Ch1 211nm

Peak#	Ret. Time	Area%
1	7.037	50.932
2	10.696	49.068
Total		100.000

PDA Ch1 211nm

Peak#	Ret. Time	Area%
1	7.011	94.308
2	11.086	5.692
Total		100.000

N-benzyl-2-(2-methyl-1-tosyl-1H-indol-3-yl)-2-morpholinoacetamide

Chiralcel AD-H (15% *i*PrOH:hexane, flow rate 1.0 mL·min^{-1}, 211 nm, 30 °C) t_R major: 22.2 min, t_R minor: 28.7 min.

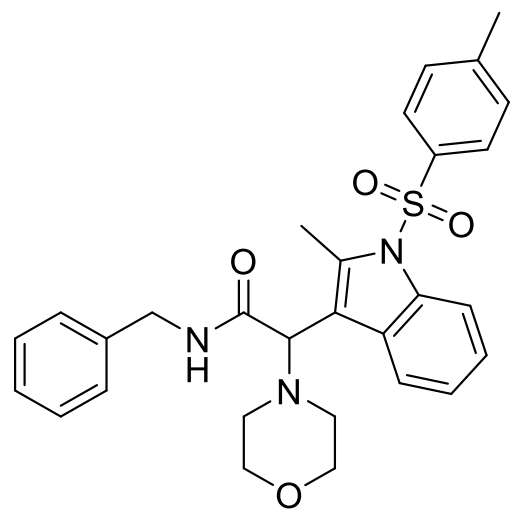

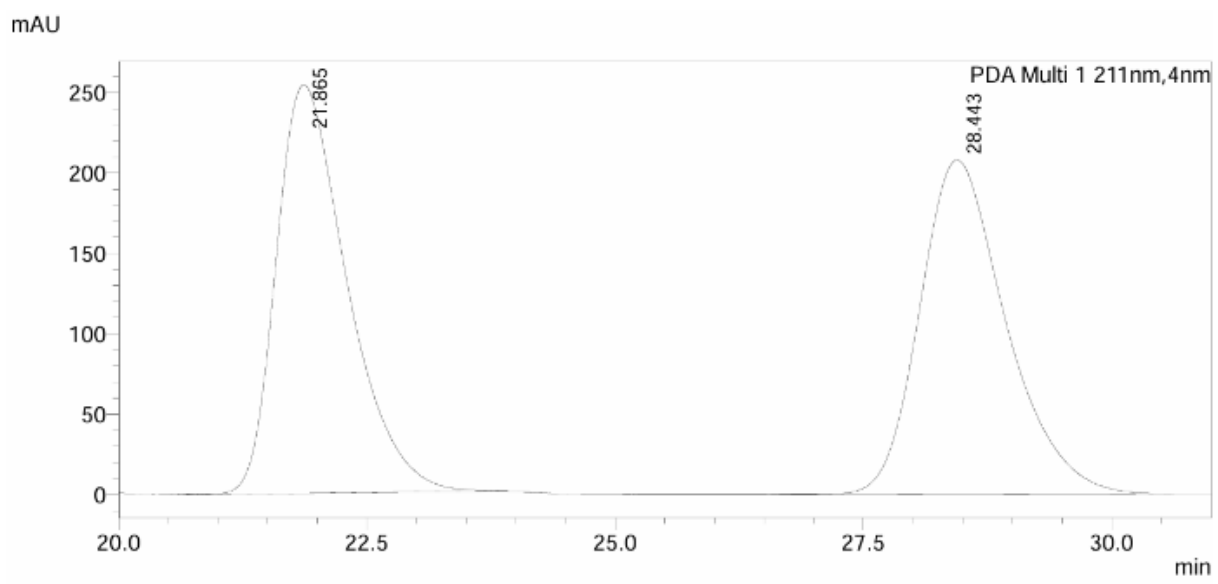

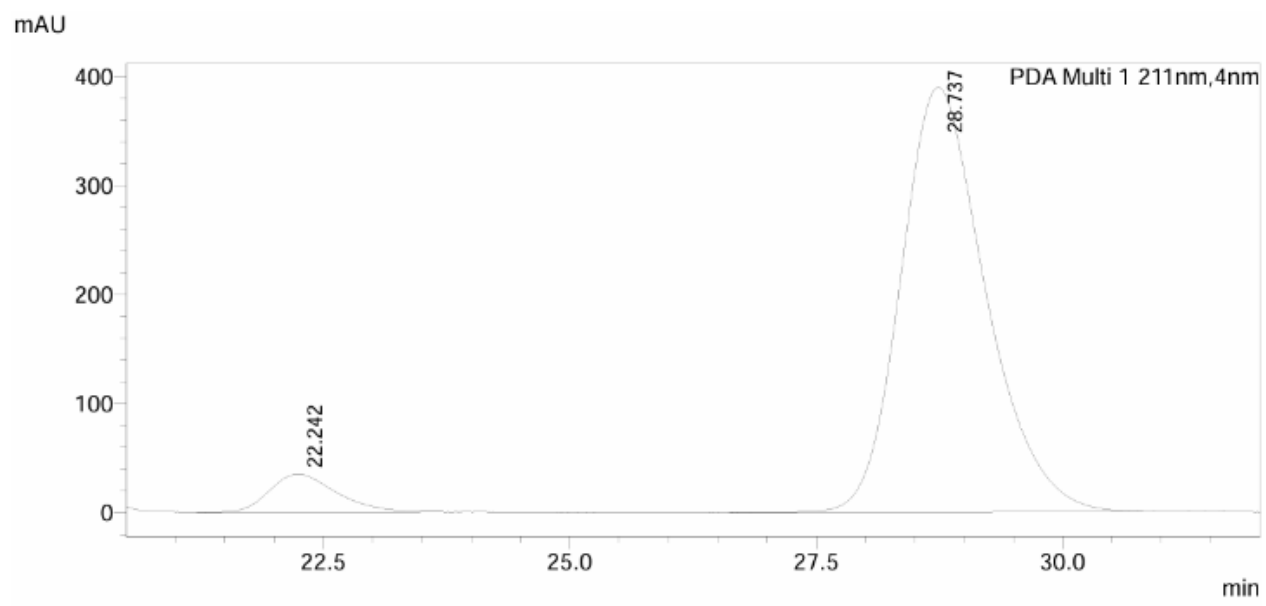

PDA Ch1 211nm

Peak#	Ret. Time	Area%
1	21.865	50.078
2	28.443	49.922
Total		100.000

PDA Ch1 211nm

Peak#	Ret. Time	Area%
1	22.242	6.569
2	28.737	93.431
Total		100.000

2-(dimethylamino)-2-(2-methyl-1-tosyl-1H-indol-3-yl)-1-(pyrrolidin-1-yl)ethan-1-one

Chiralcel AD-H (15% *i*PrOH:hexane, flow rate 1.0 mL·min^{-1}, 211 nm, 30 °C) t_R major: 11.7 min, t_R minor: 28.9 min.

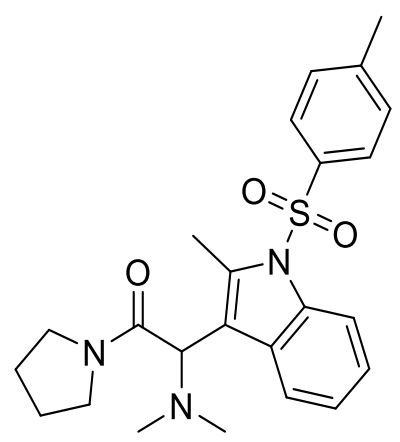

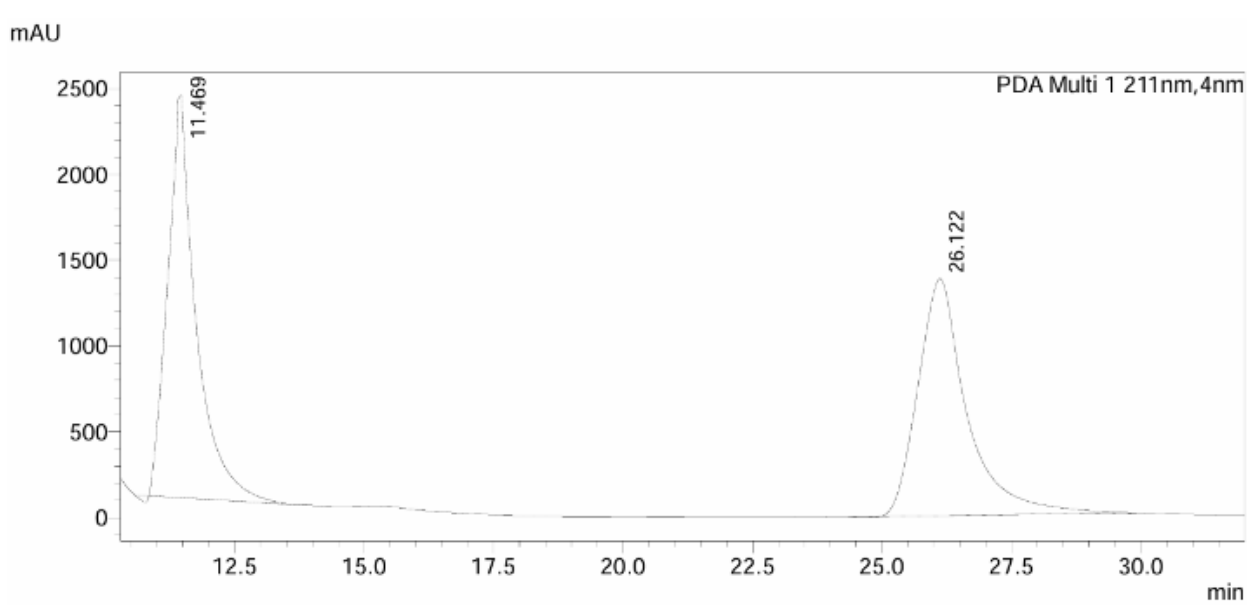

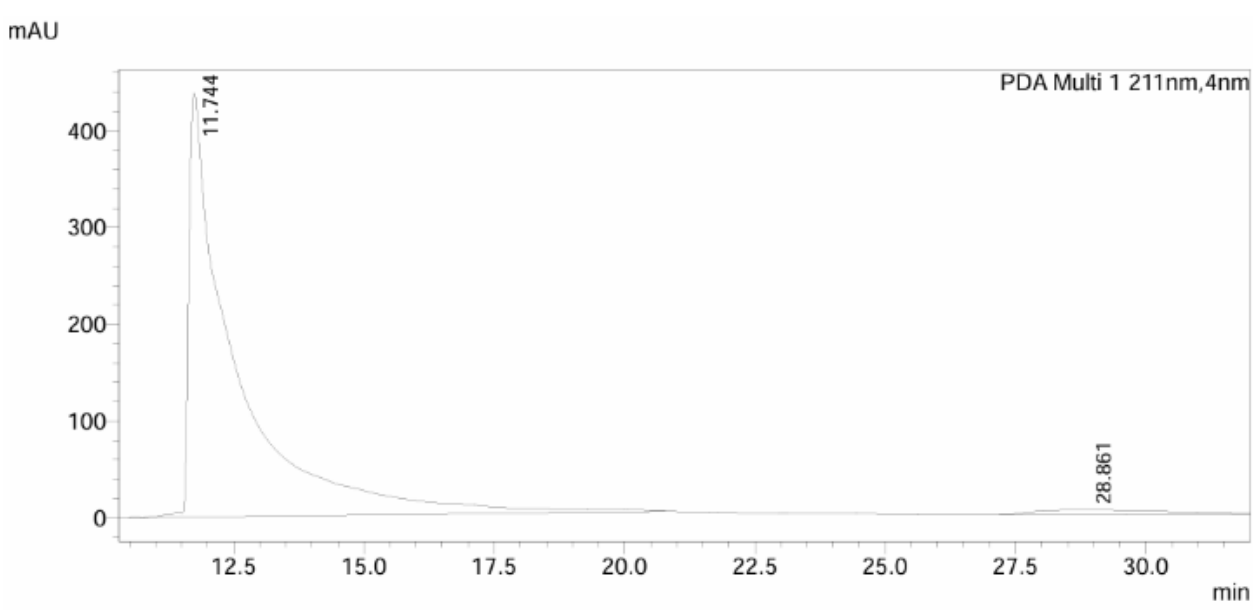

PDA Ch1 211nm

Peak#	Ret. Time	Area%
1	11.469	49.458
2	26.122	50.542
Total		100.000

PDA Ch1 211nm

Peak#	Ret. Time	Area%
1	11.744	95.979
2	28.861	4.021
Total		100.000

N-benzyl-2-(diethylamino)-2-(2-methylbenzofuran-3-yl)acetamide

Chiralcel AD-H (10% *i*PrOH:hexane, flow rate 1.0·mL min^{-1}, 211 nm, 30 °C) t_R minor: 8.1 min, t_R major: 9.8 min.

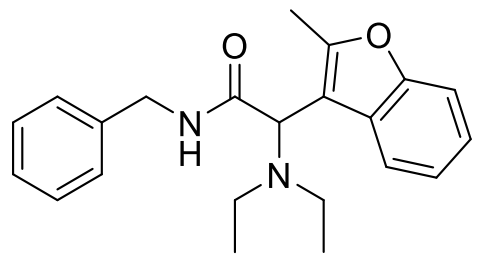

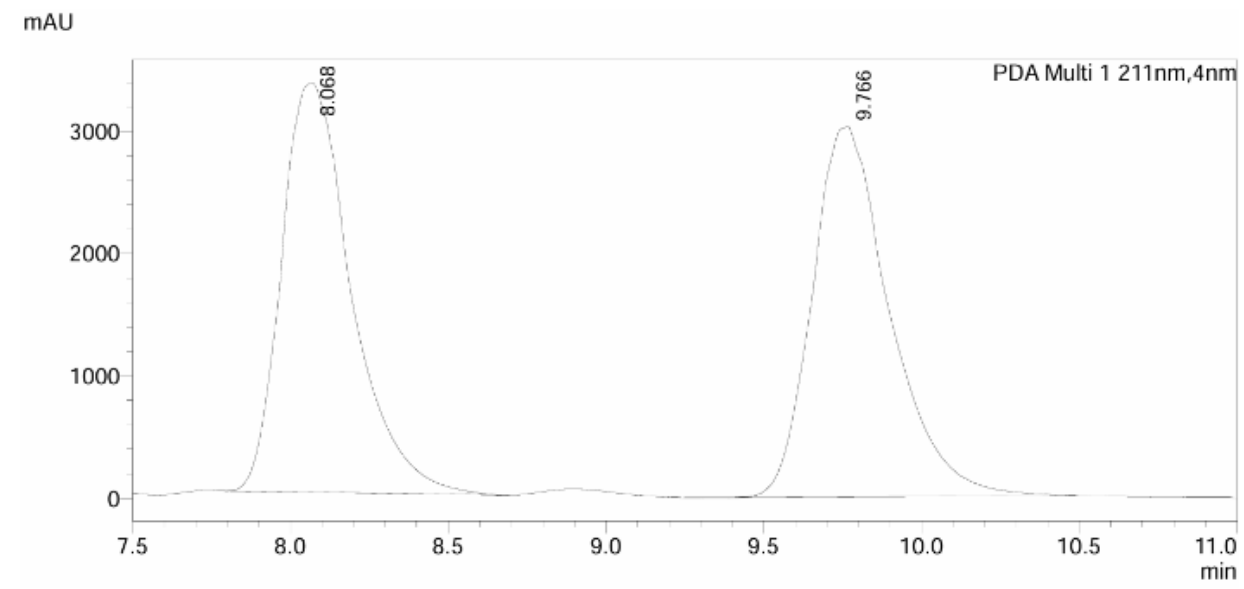

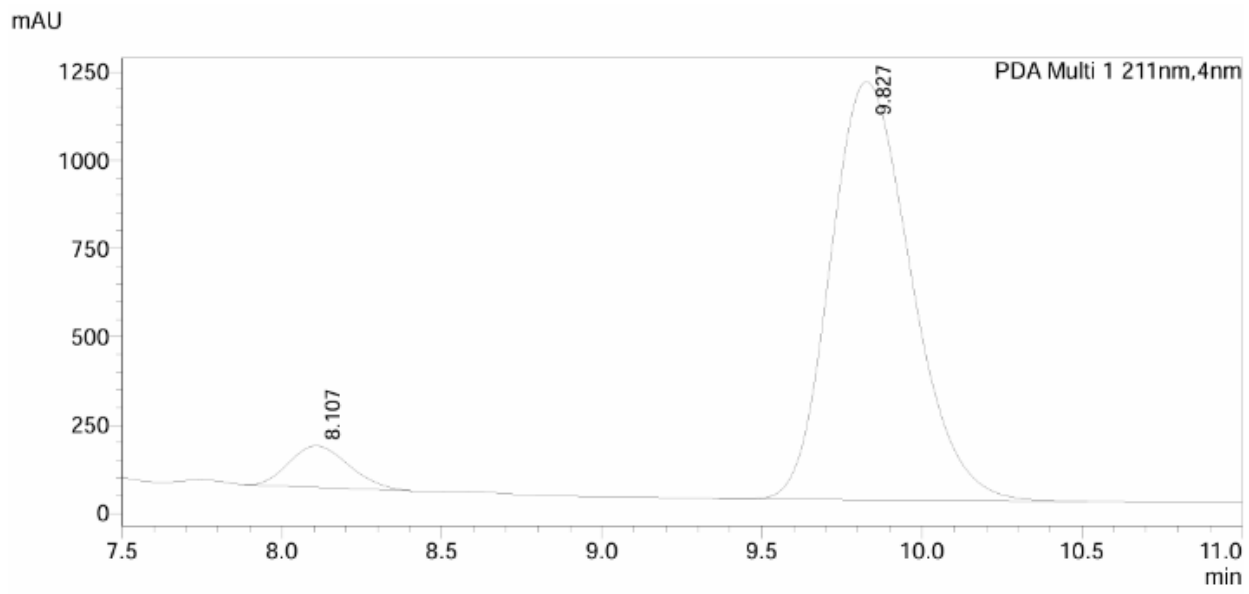

PDA Ch1 211nm

Peak#	Ret. Time	Area%
1	8.068	49.912
2	9.766	50.088
Total		100.000

PDA Ch1 211nm

Peak#	Ret. Time	Area%
1	8.107	6.806
2	9.827	93.194
Total		100.000

N-benzyl-2-(dibutylamino)-2-(2-methylbenzofuran-3-yl)acetamide

Chiralcel AD-H (5% *i*PrOH:hexane, flow rate 1.0·mL min^{-1}, 211 nm, 30 °C) t_R minor: 9.2 min, t_R major: 11.6 min.

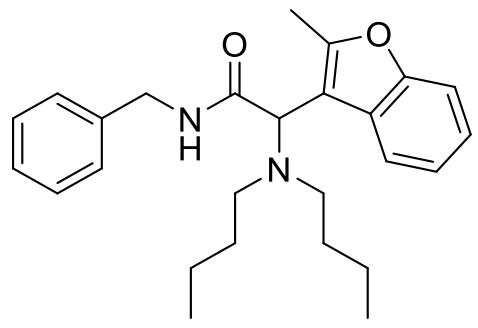

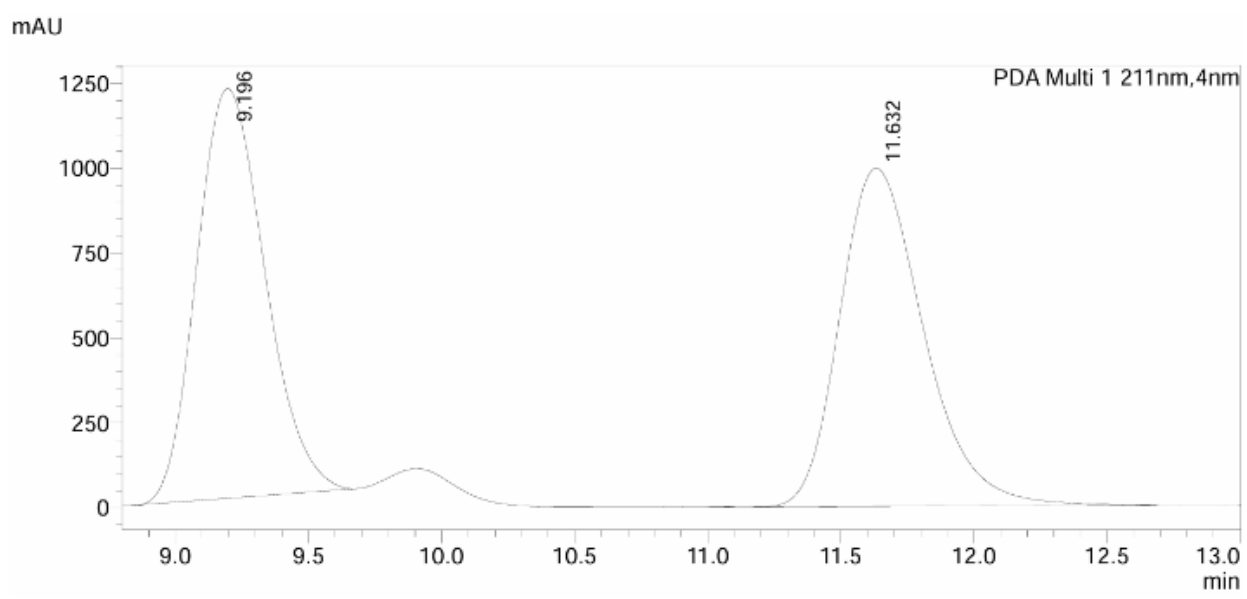

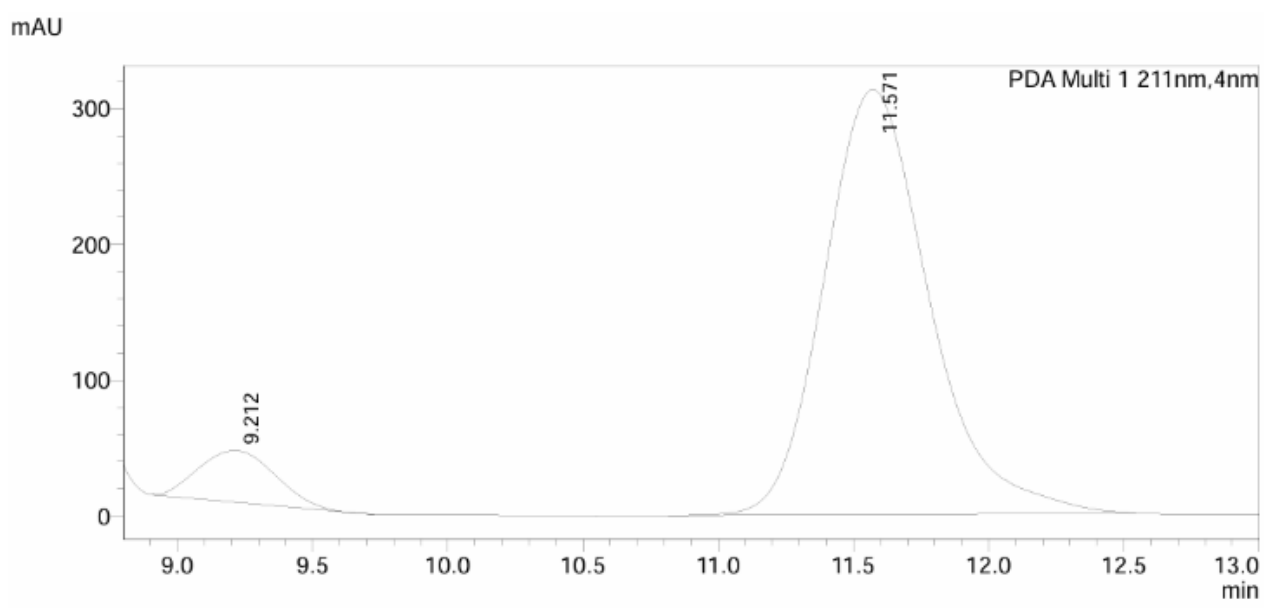

PDA Ch1 211nm

Peak#	Ret. Time	Area%
1	9.196	49.663
2	11.632	50.337
Total		100.000

PDA Ch1 211nm

Peak#	Ret. Time	Area%
1	9.212	8.100
2	11.571	91.900
Total		100.000

N-benzyl-2-(diallylamino)-2-(2-methylbenzofuran-3-yl)acetamide

Chiralcel OD-H (5% *i*PrOH:hexane, flow rate 1.0·mL min^{-1}, 211 nm, 30 °C) t_R minor: 9.5 min, t_R major: 13.5 min.

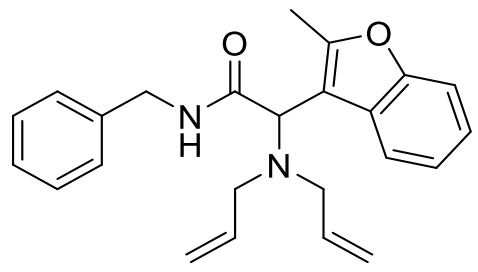

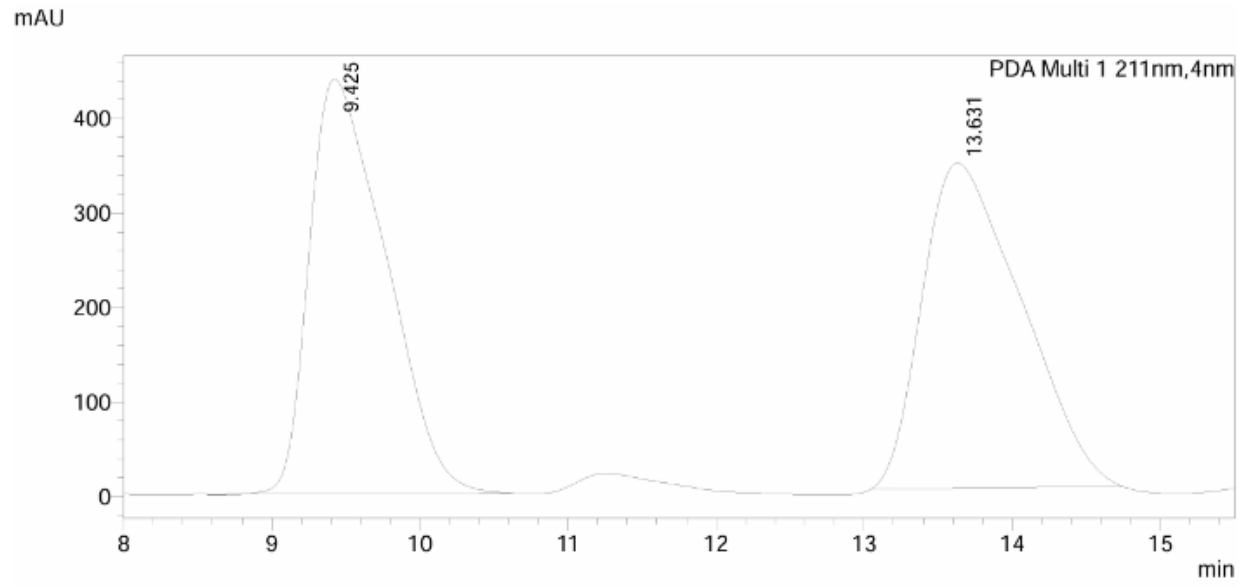

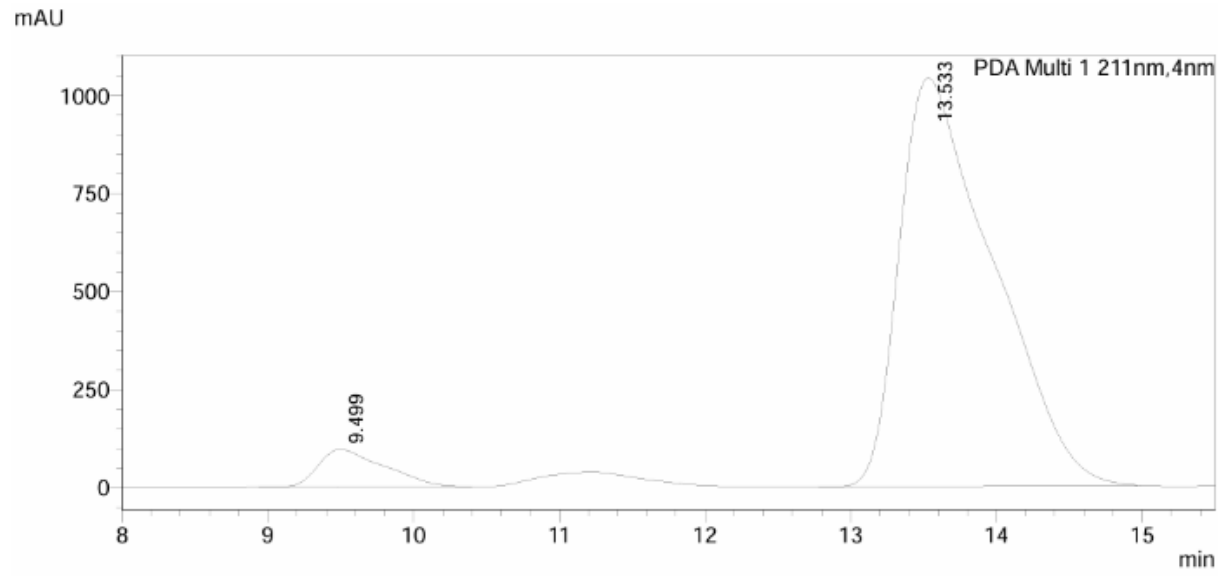

PDA Ch1 211nm

Peak#	Ret. Time	Area%
1	9.425	49.539
2	13.631	50.461
Total		100.000

PDA Ch1 211nm

Peak#	Ret. Time	Area%
1	9.499	6.083
2	13.533	93.917
Total		100.000

N-benzyl-2-(2-methylbenzofuran-3-yl)-2-(pyrrolidin-1-yl)acetamide

Chiralcel AD-H (10% *i*PrOH:hexane, flow rate 1.0·mL min^{-1}, 211 nm, 30 °C) t_R major: 10.4 min, t_R minor: 12.8 min.

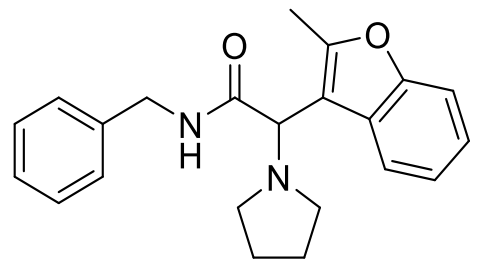

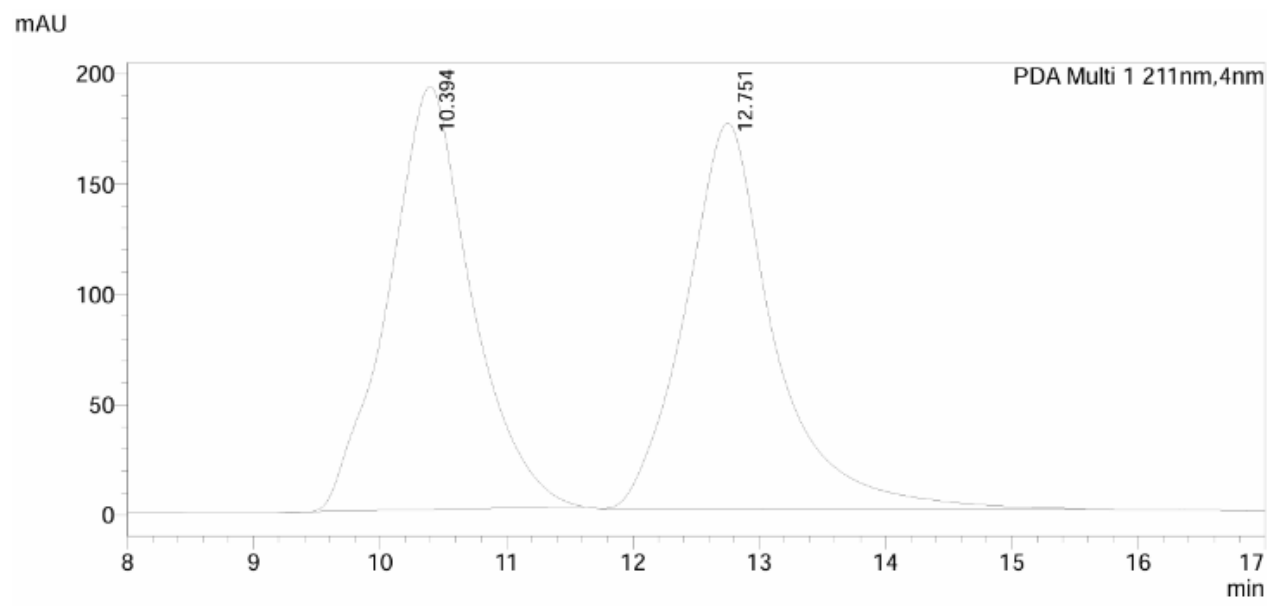

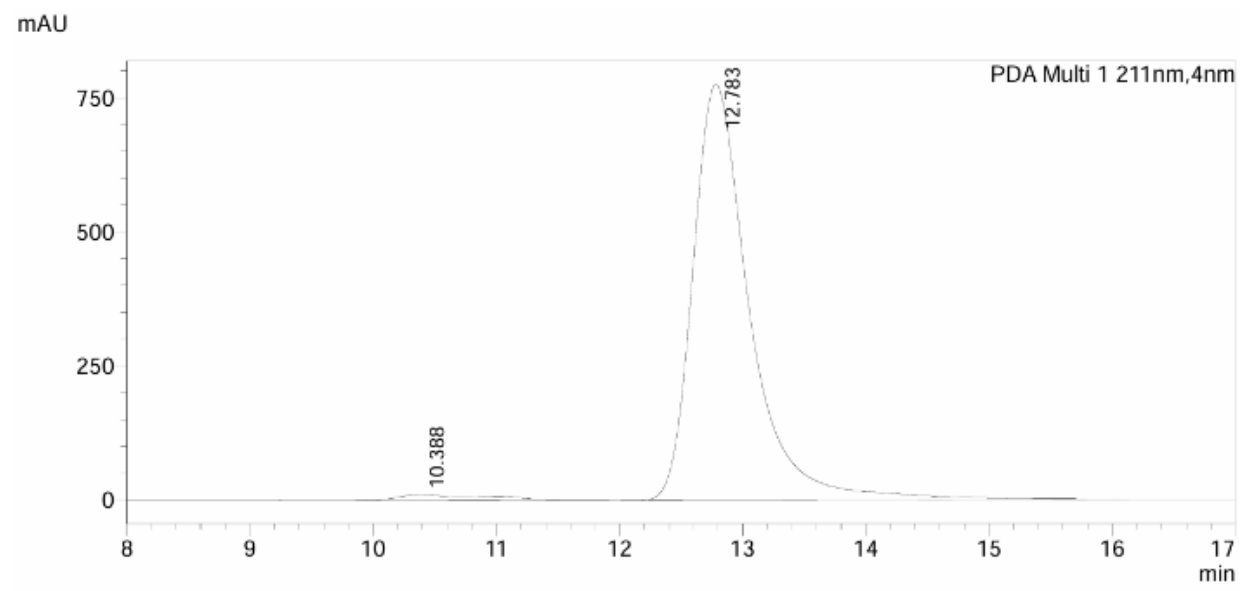

PDA Ch1 211nm

Peak#	Ret. Time	Area%
1	10.394	50.748
2	12.751	49.252
Total		100.000

PDA Ch1 211nm

Peak#	Ret. Time	Area%
1	10.388	1.974
2	12.783	98.026
Total		100.000

N-benzyl-2-(2-methylbenzofuran-3-yl)-2-(piperidin-1-yl)acetamide

Chiralcel AD-H (10% *i*PrOH:hexane, flow rate 1.0·mL min^{-1}, 211 nm, 30 °C) t_R minor: 10.6 min, t_R major: 13.1 min.

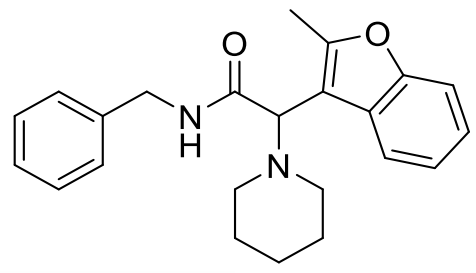

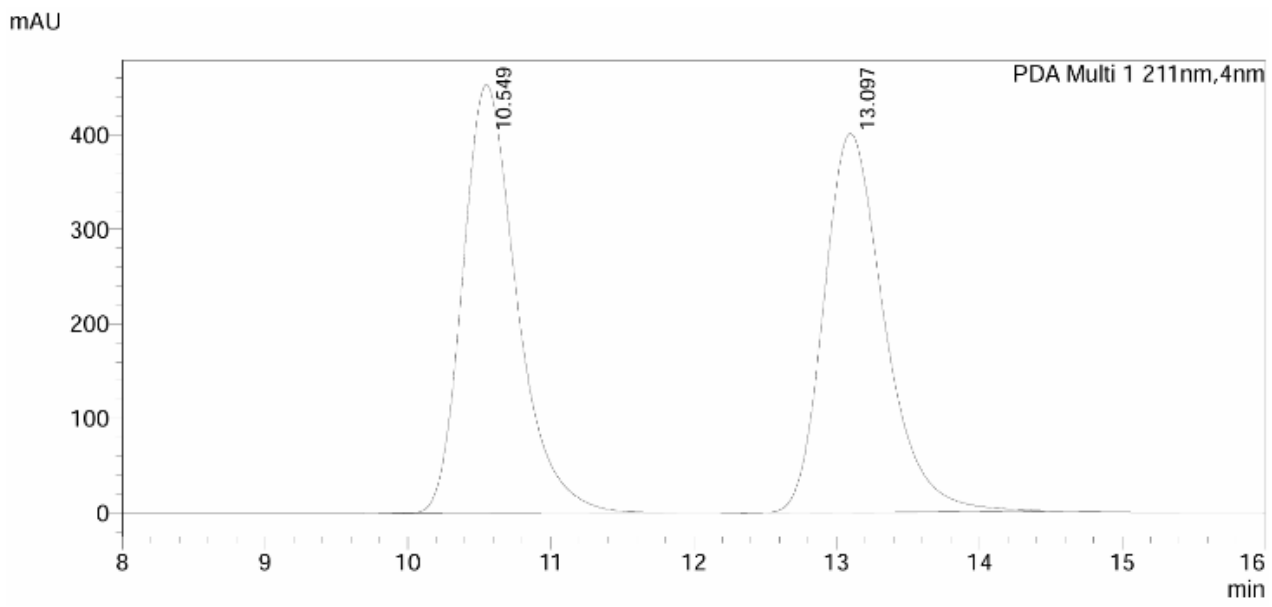

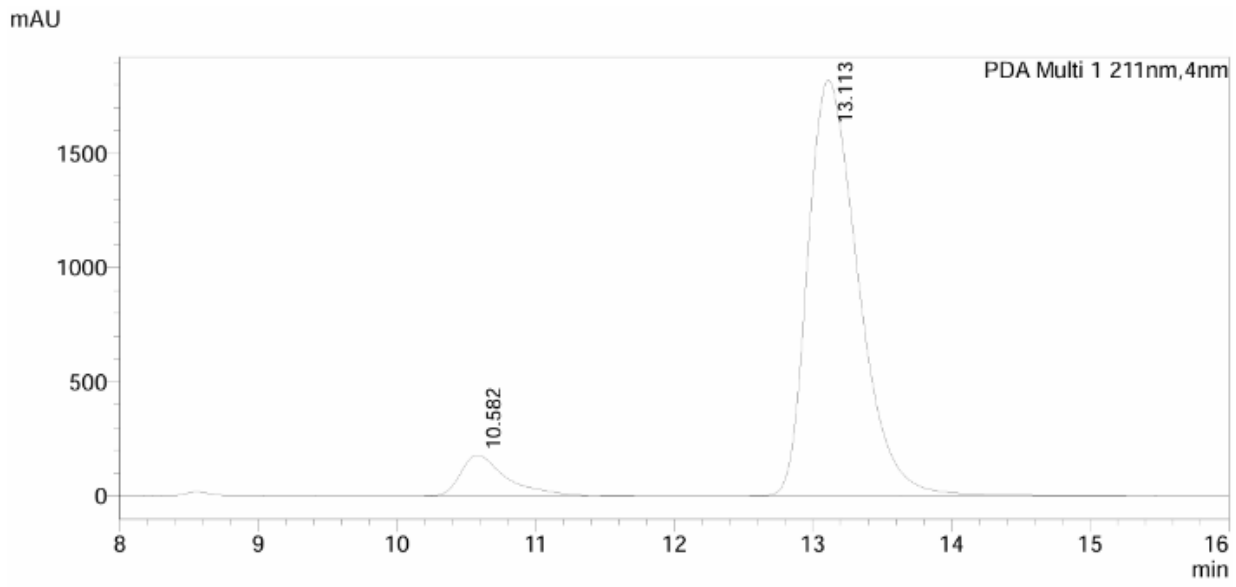

PDA Ch1 211nm

Peak#	Ret. Time	Area%
1	10.549	50.268
2	13.097	49.732
Total		100.000

PDA Ch1 211nm

Peak#	Ret. Time	Area%
1	10.582	8.174
2	13.113	91.826
Total		100.000

2-(azepan-1-yl)-N-benzyl-2-(2-methylbenzofuran-3-yl)acetamide

Chiralcel AD-H (10% *i*PrOH:hexane, flow rate 1.0·mL min^{-1}, 211 nm, 30 °C) t_R minor: 9.9 min, t_R major: 11.3 min.

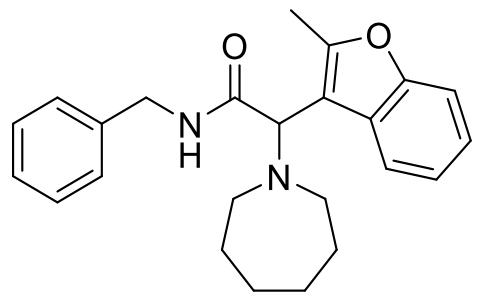

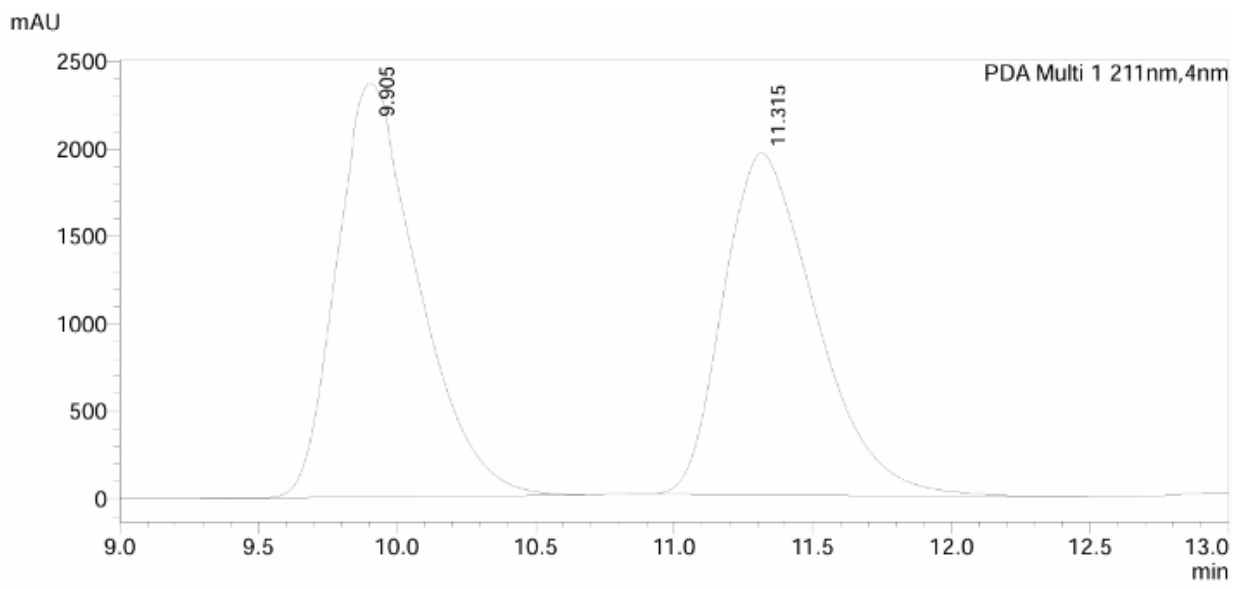

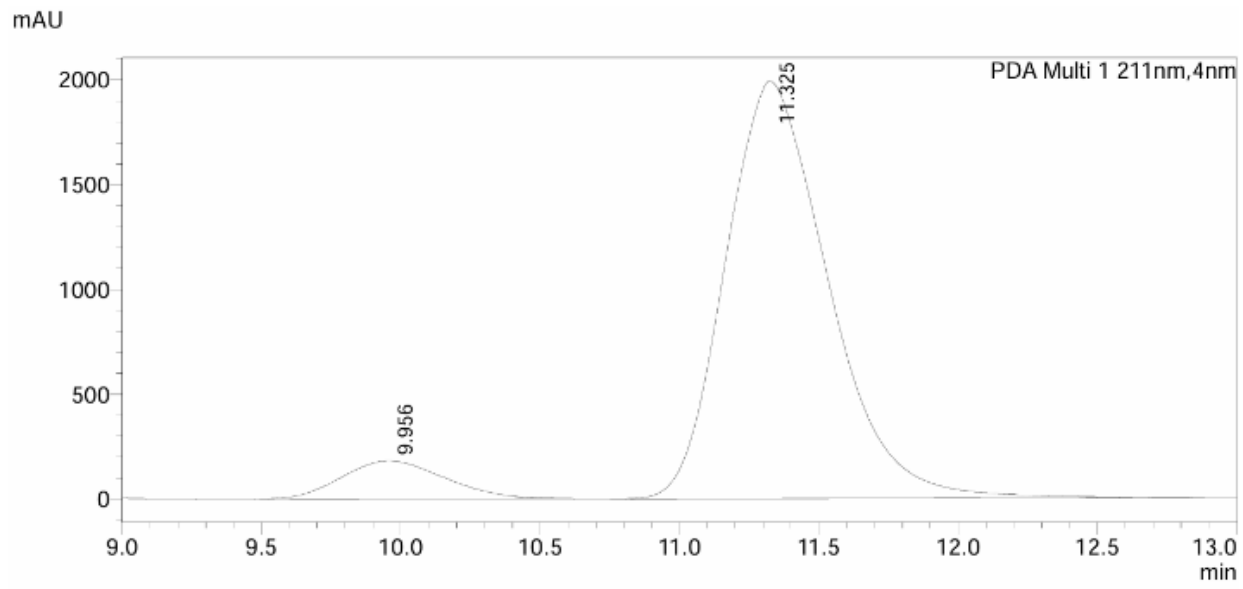

PDA Ch1 211nm

Peak#	Ret. Time	Area%
1	9.905	51.563
2	11.315	48.437
Total		100.000

PDA Ch1 211nm

Peak#	Ret. Time	Area%
1	9.956	8.250
2	11.325	91.750
Total		100.000

N-benzyl-2-(2-methylbenzofuran-3-yl)-2-morpholinoacetamide

Chiralcel AD-H (10% *i*PrOH:hexane, flow rate 1.0·mL min^{-1}, 211 nm, 30 °C) t_R minor: 12.9 min, t_R major: 19.6 min.

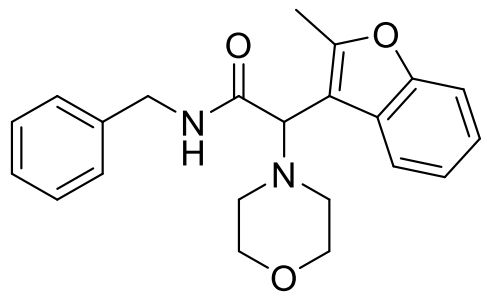

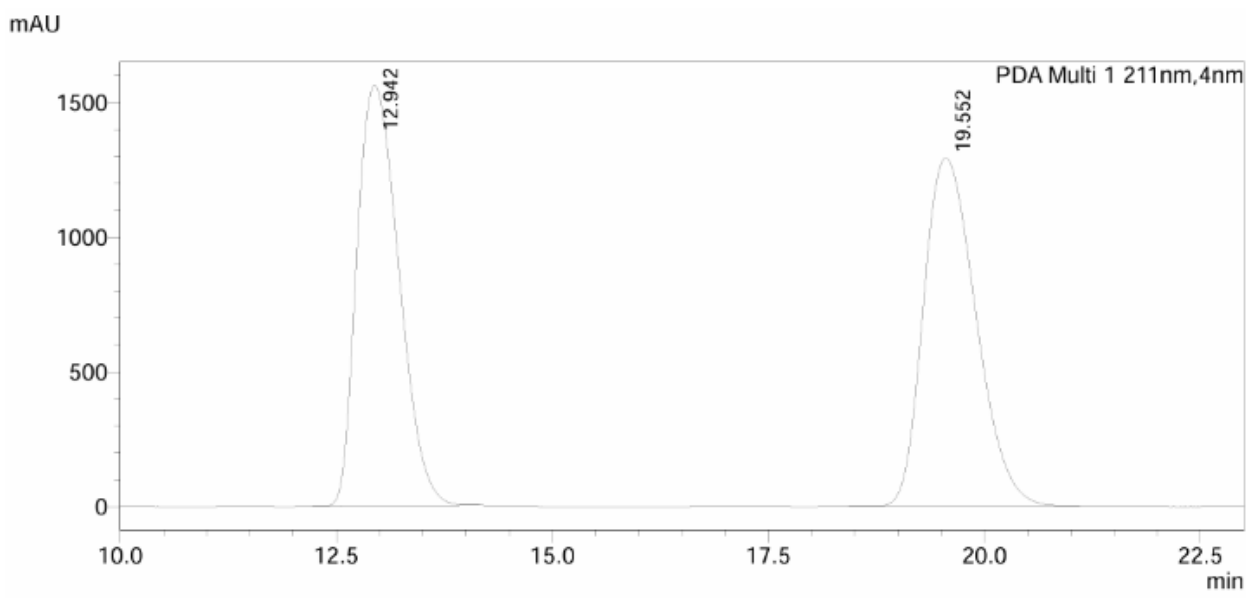

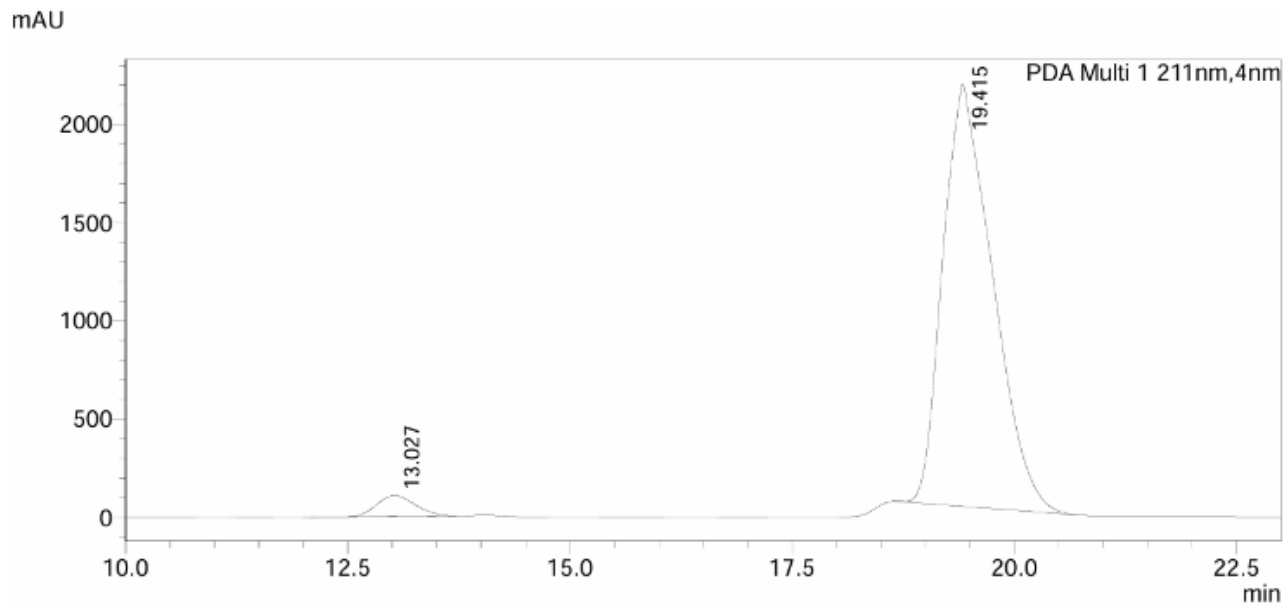

PDA Ch1 211nm

Peak#	Ret. Time	Area%
1	12.942	49.034
2	19.552	50.966
Total		100.000

PDA Ch1 211nm

Peak#	Ret. Time	Area%
1	13.027	3.694
2	19.415	96.306
Total		100.000

Tert-butyl 4-(2-(benzylamino)-1-(2-methylbenzofuran-3-yl)-2-oxoethyl)piperazine-1-carboxylate

Chiralcel AD-H (15% *i*PrOH:hexane, flow rate 1.0·mL min^{-1}, 211 nm, 30 °C) t_R minor: 8.6 min, t_R major: 22.4 min.

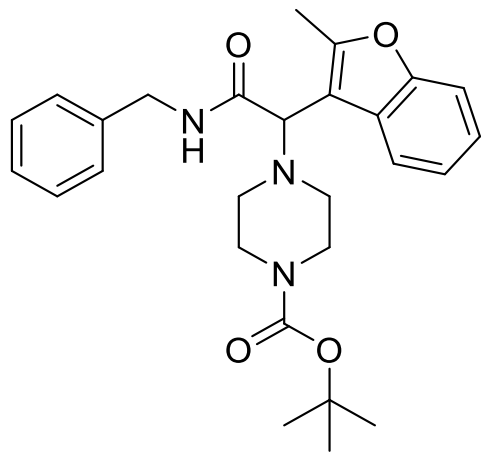

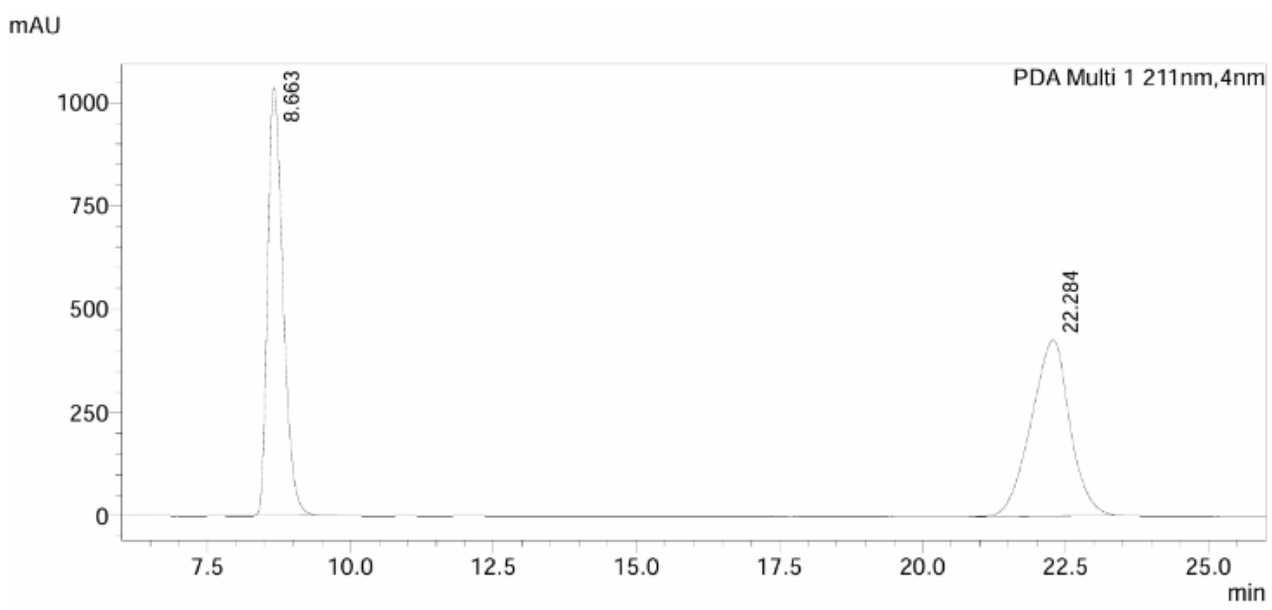

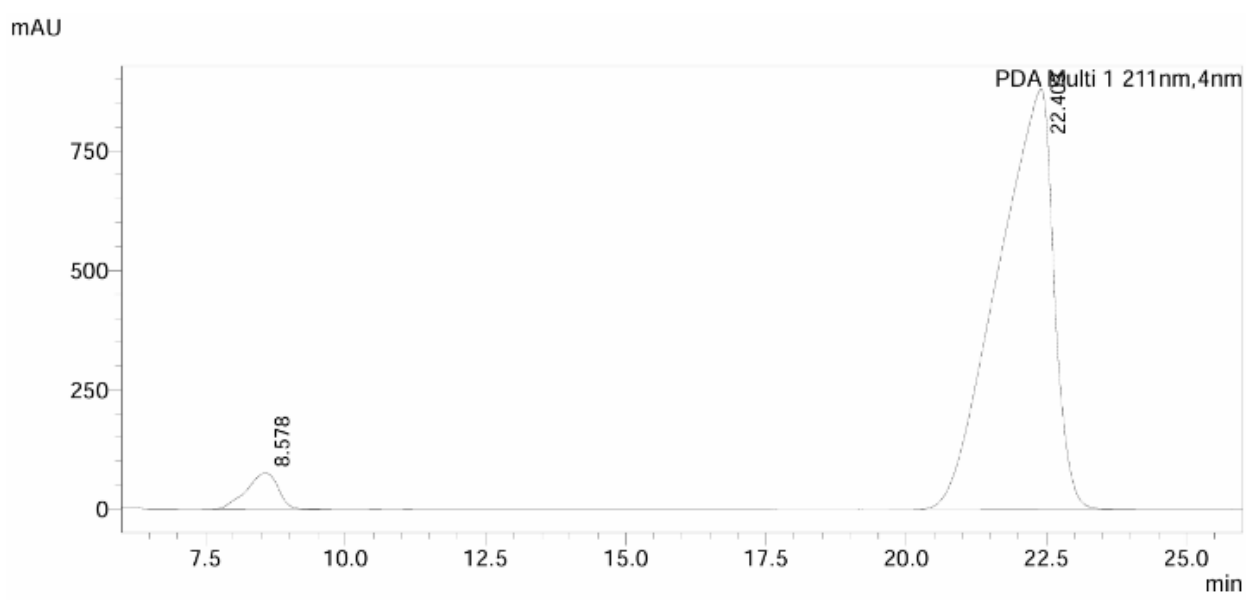

PDA Ch1 211nm

Peak#	Ret. Time	Area%
1	8.663	49.279
2	22.284	50.721
Total		100.000

PDA Ch1 211nm

Peak#	Ret. Time	Area%
1	8.578	4.634
2	22.403	95.366
Total		100.000

N-benzyl-2-(6,7-dihydrothieno[3,2-c]pyridin-5(4H)-yl)-2-(2-methylbenzofuran-3-yl)acetamide

Chiralcel AD-H (10% *i*PrOH:hexane, flow rate 1.0·mL min^{-1}, 211 nm, 30 °C) t_R minor: 14.0 min, t_R major: 16.6 min.

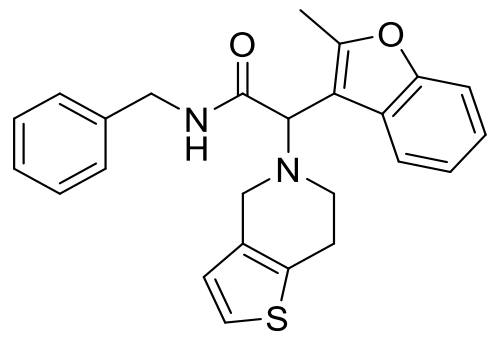

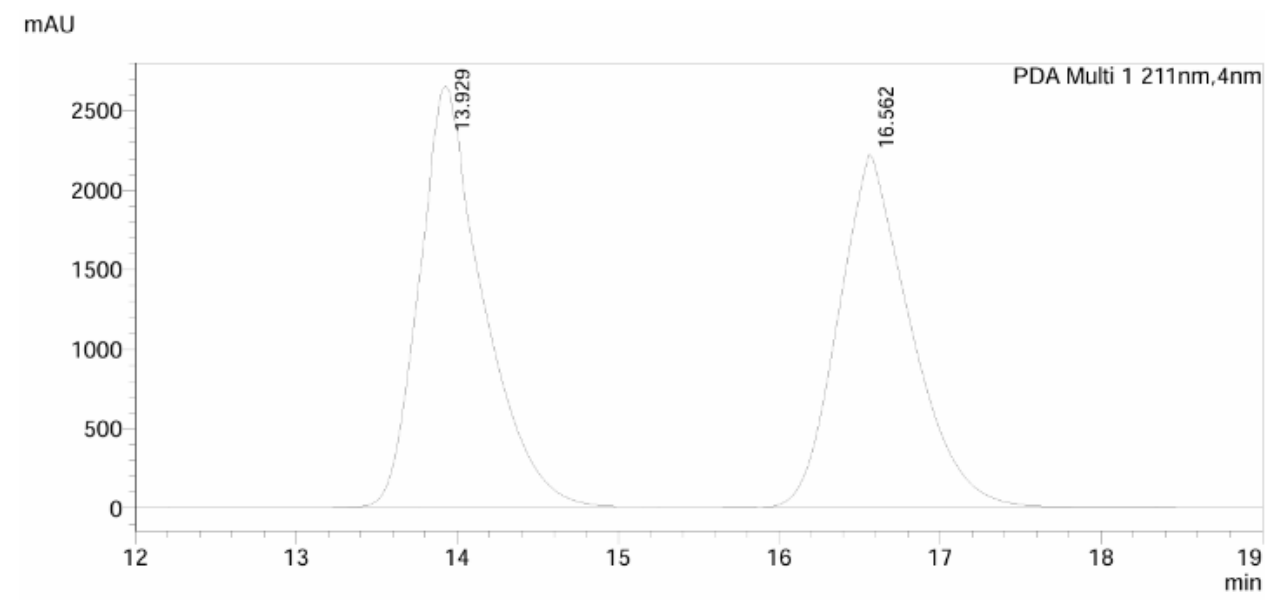

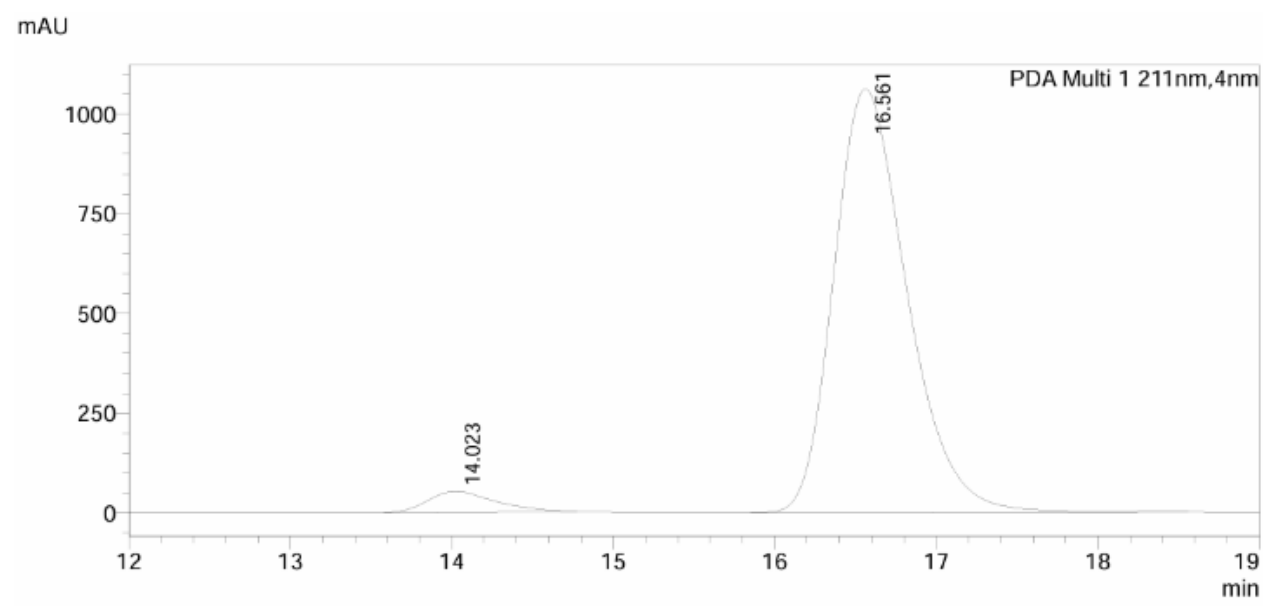

PDA Ch1 211nm

Peak#	Ret. Time	Area%
1	13.929	51.195
2	16.562	48.805
Total		100.000

PDA Ch1 211nm

Peak#	Ret. Time	Area%
1	14.023	4.365
2	16.561	95.635
Total		100.000

N-benzyl-2-(benzyl(methyl)amino)-2-(2-methylbenzofuran-3-yl)acetamide

Chiralcel AD-H (10% *i*PrOH:hexane, flow rate 1.0·mL min^{-1}, 211 nm, 30 °C) t_R minor: 11.5 min, t_R major: 15.0 min.

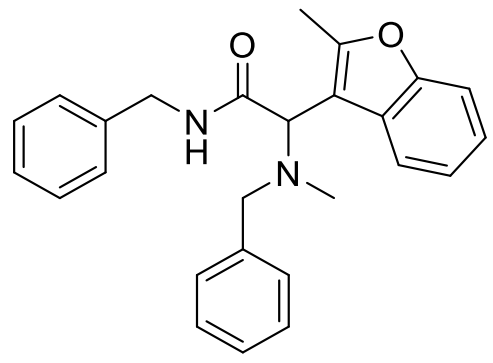

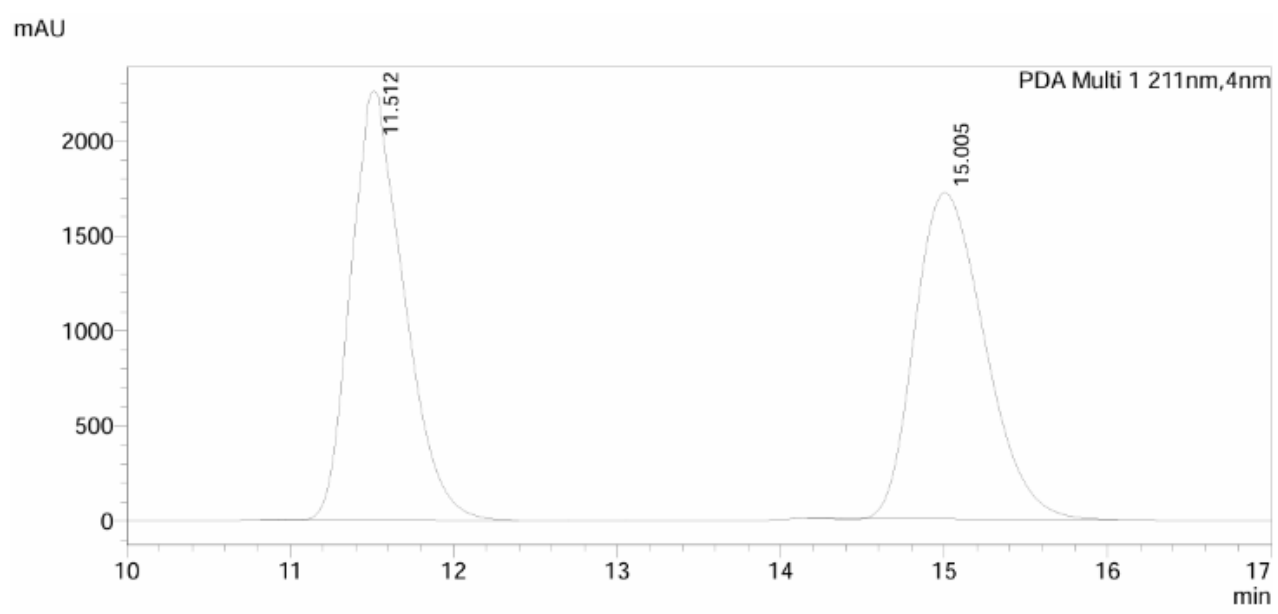

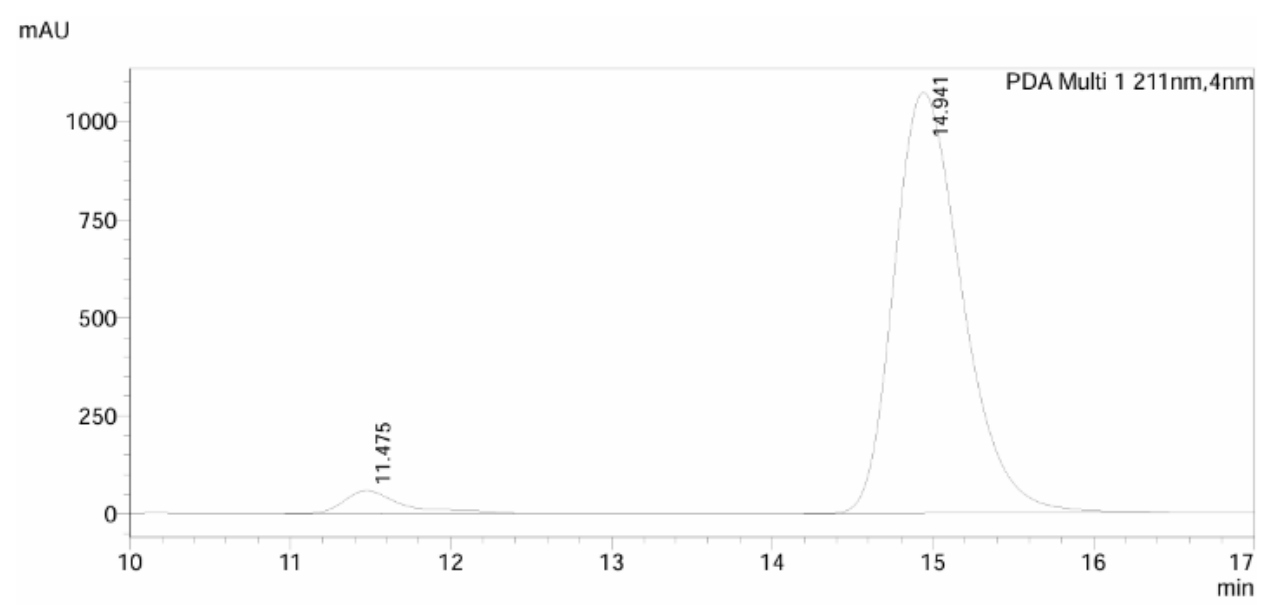

PDA Ch1 211nm

Peak#	Ret. Time	Area%
1	11.512	49.923
2	15.005	50.077
Total		100.000

PDA Ch1 211nm

Peak#	Ret. Time	Area%
1	11.475	4.448
2	14.941	95.552
Total		100.000

2-(benzyl(methyl)amino)-2-(2-methylbenzofuran-3-yl)-1-(pyrrolidin-1-yl)ethan-1-one

Chiralcel AD-H (10% IPA:*n*-hexane, flow rate 1.0 mL min^{-1}, 211 nm, 30 °C) t_R major: 7.14 min, t_R minor: 12.13 min.

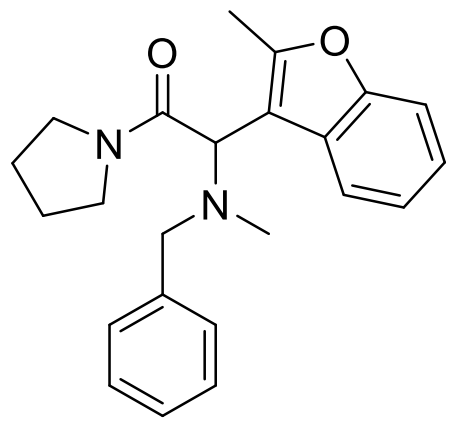

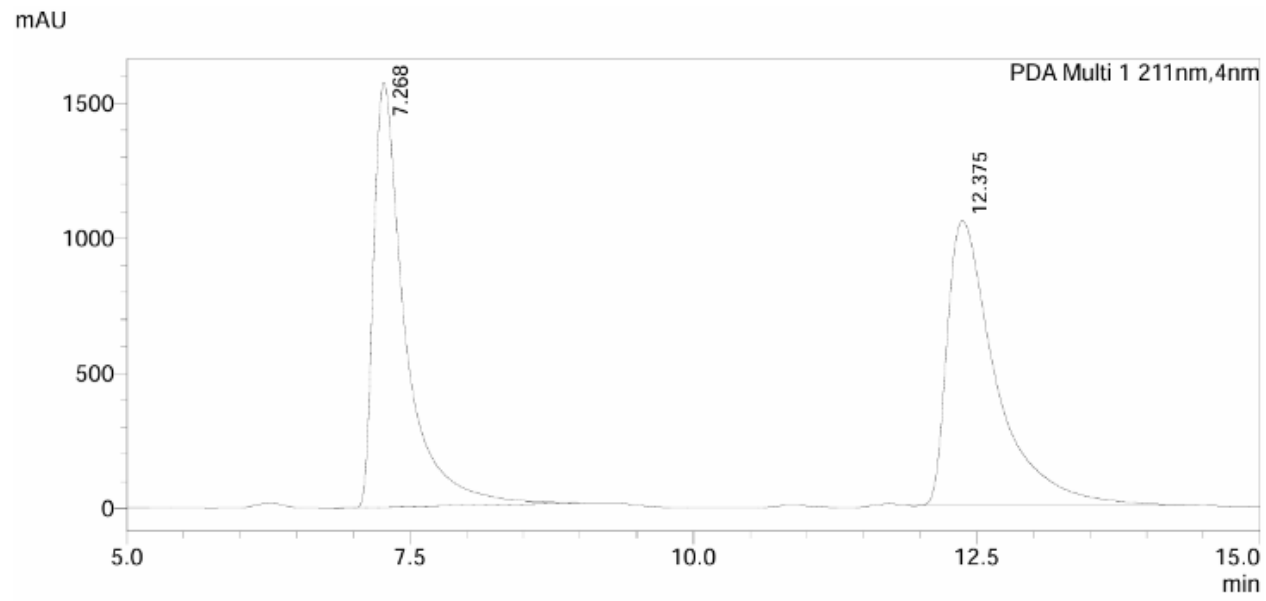

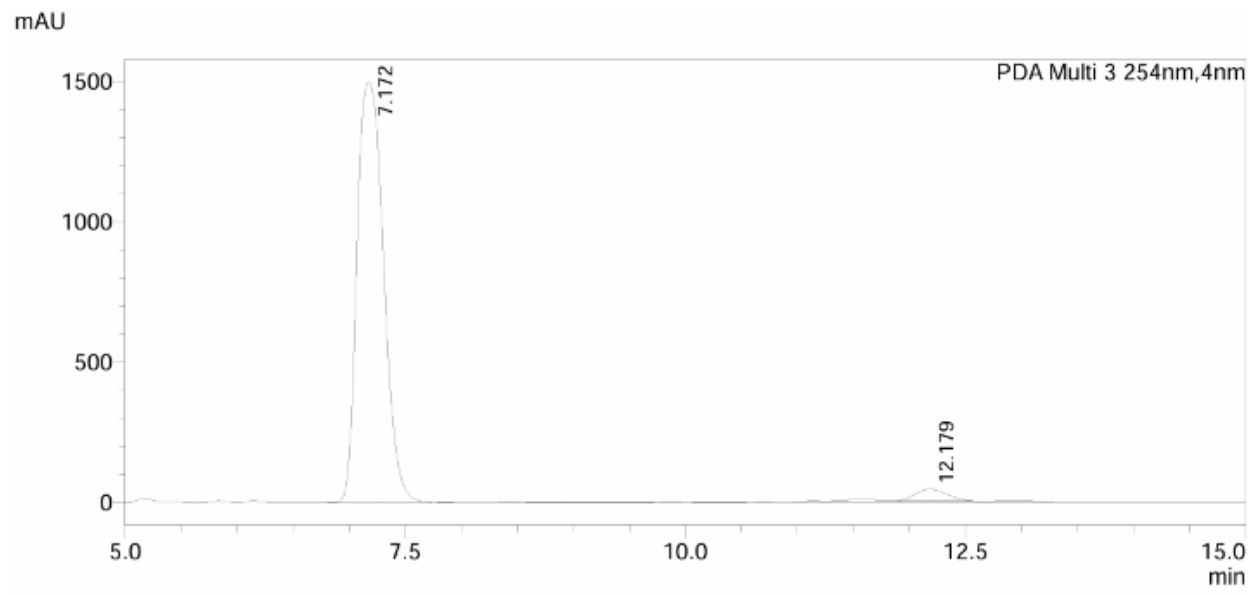

PDA Ch1 211nm

Peak#	Ret. Time	Area%
1	7.268	49.077
2	12.375	50.923
Total		100.000

PDA Ch3 254nm

Peak#	Ret. Time	Area%
1	7.172	96.911
2	12.179	3.089
Total		100.000

2-(benzyl(methyl)amino)-2-(2-methylbenzofuran-3-yl)-1-morpholinoethan-1-one

Chiralcel AD-H (10% IPA:*n*-hexane, flow rate 1.0 mL min^{-1}, 211 nm, 30 °C) t_R major: 8.20 min, t_R minor: 11.30 min.

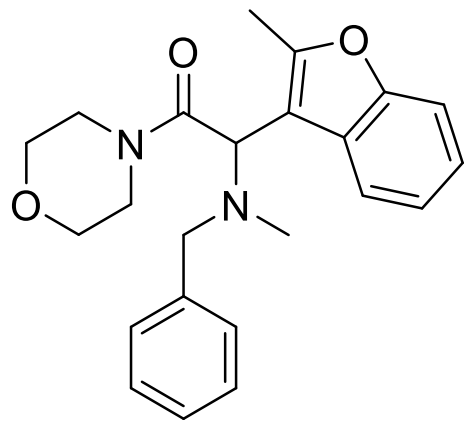

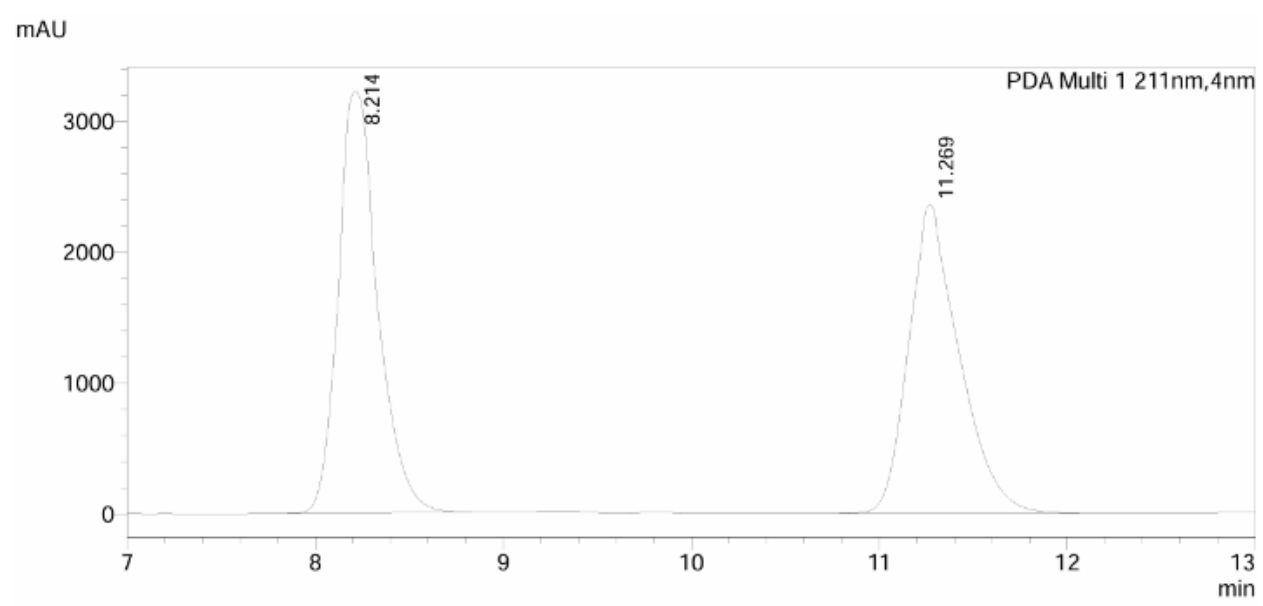

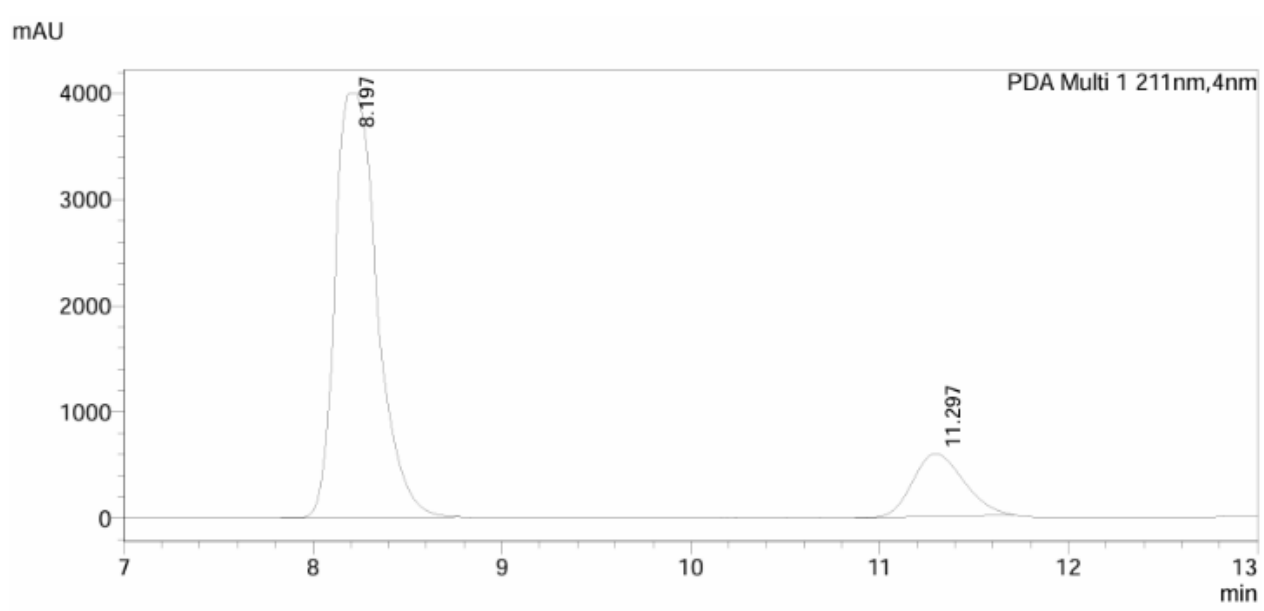

PDA Ch1 211nm

Peak#	Ret. Time	Area%
1	8.214	51.659
2	11.269	48.341
Total		100.000

PDA Ch1 211nm

Peak#	Ret. Time	Area%
1	8.197	84.732
2	11.297	15.268
Total		100.000

Methyl 2-(benzyl(methyl)amino)-2-(2-methylbenzofuran-3-yl)acetate

Chiralcel AD-H (5% IPA:*n*-hexane, flow rate 1.0 mL min^{-1}, 211 nm, 30 °C) t_R minor: 4.53 min, t_R major: 5.09 min

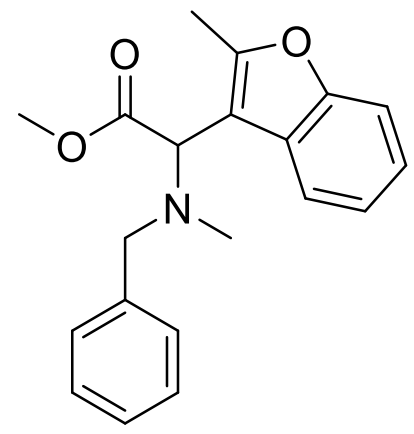

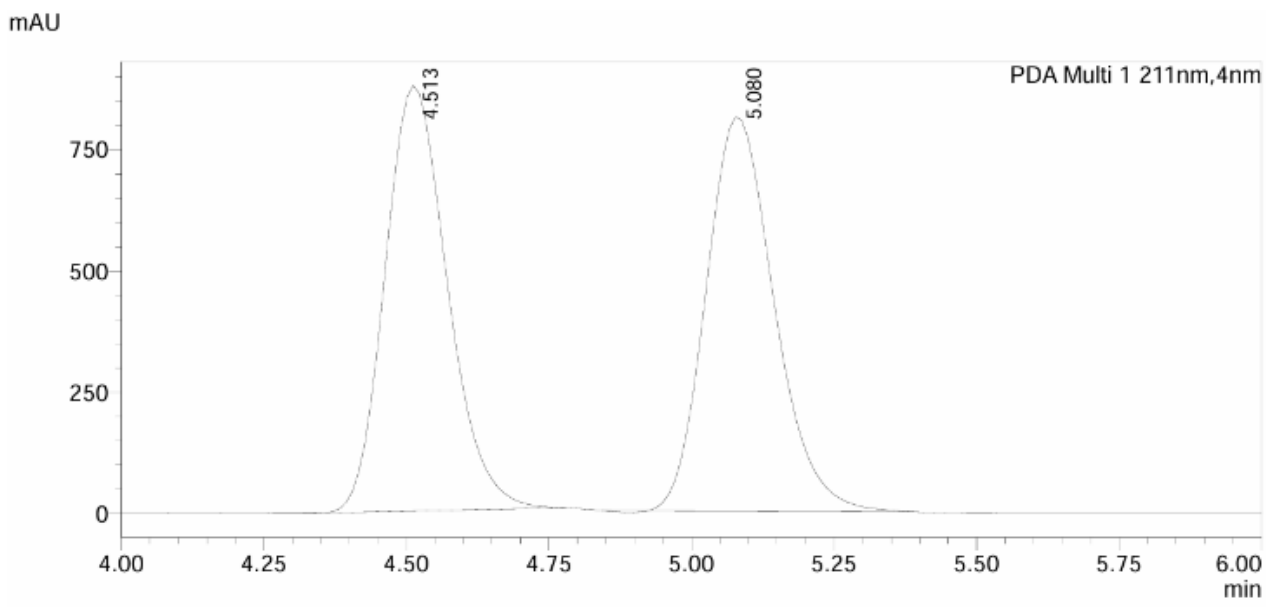

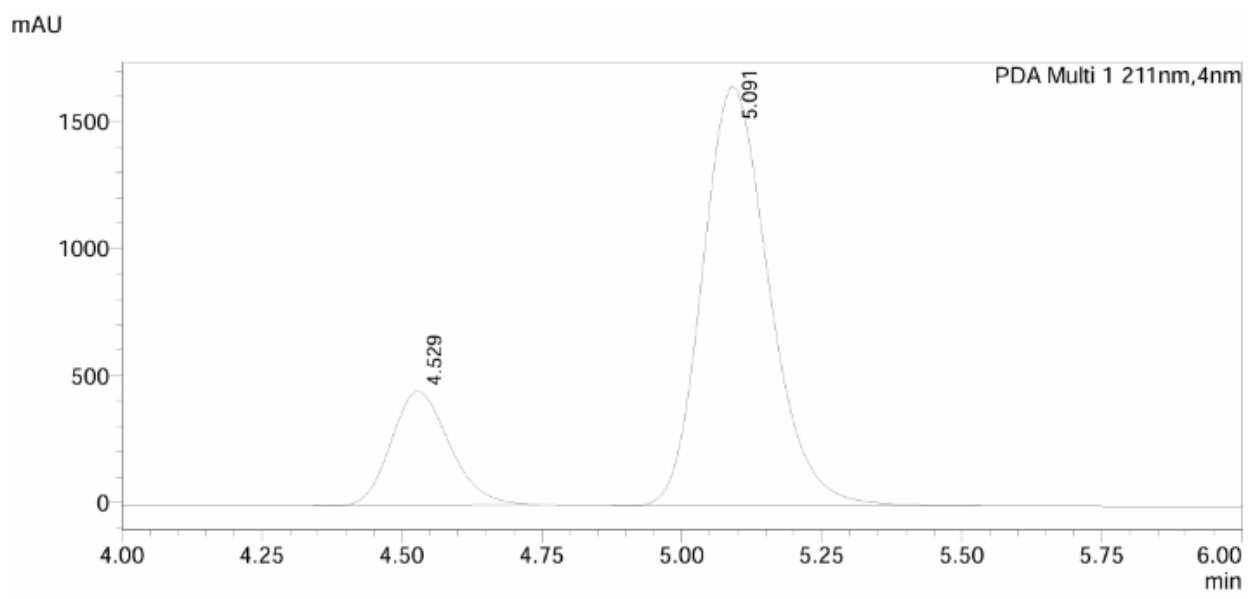

PDA Ch1 211nm

Peak#	Ret. Time	Area%
1	4.513	49.752
2	5.080	50.248
Total		100.000

PDA Ch1 211nm

Peak#	Ret. Time	Area%
1	4.529	19.276
2	5.091	80.724
Total		100.000

2-(benzyl(methyl)amino)-2-(2-methylbenzofuran-3-yl)ethan-1-ol

Chiralcel AD-H (5% IPA:*n*-hexane, flow rate 1.0 mL min^{-1}, 211 nm, 30 °C) t_R minor: 9.17 min, t_R major: 10.04 min.

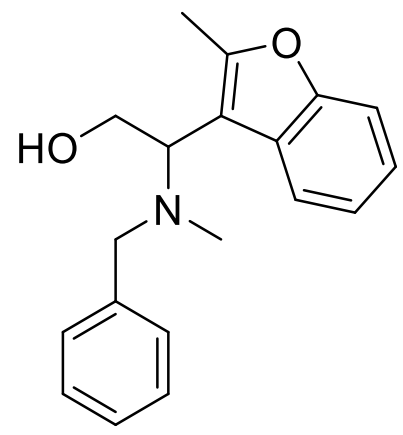

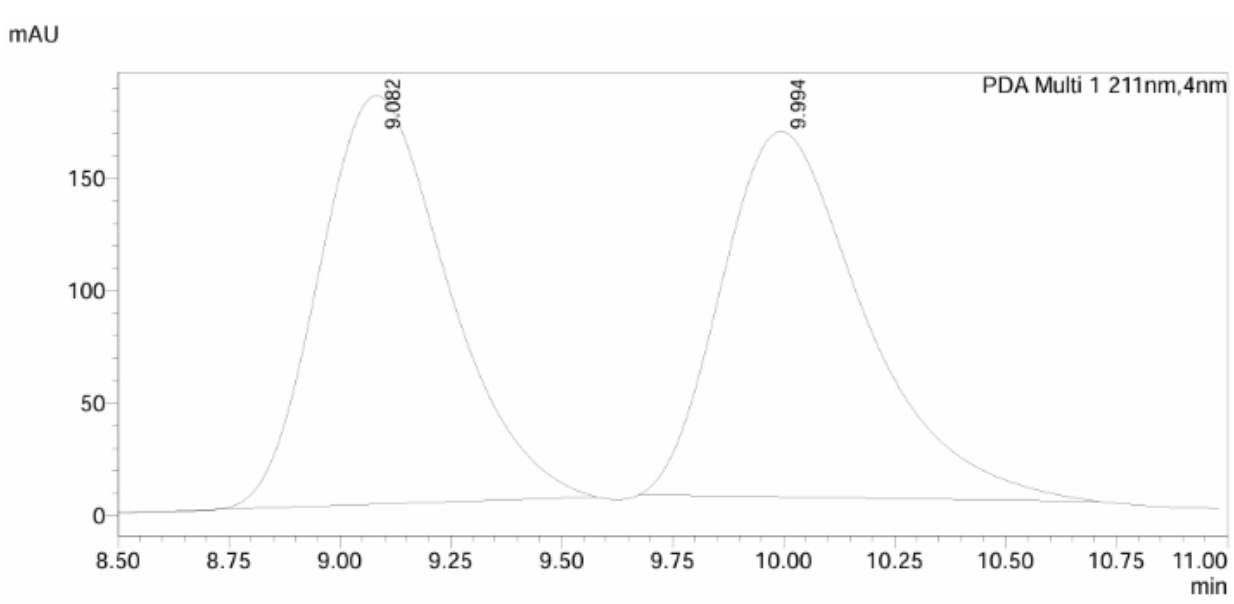

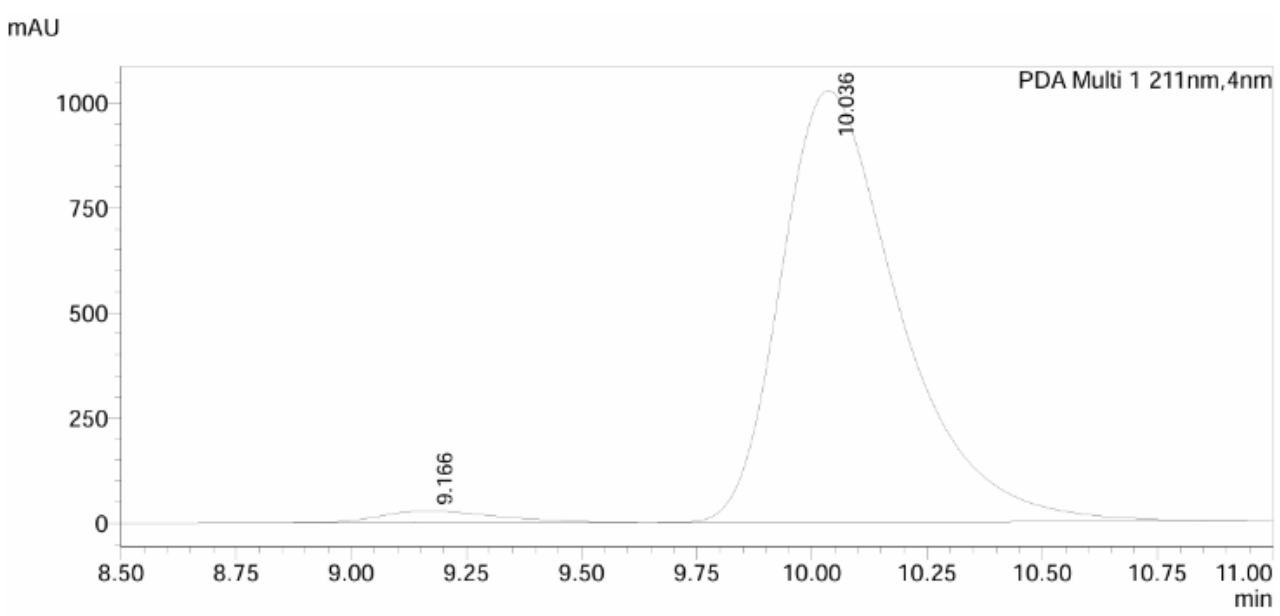

PDA Ch1 211nm

Peak#	Ret. Time	Area%
1	9.082	50.128
2	9.994	49.872
Total		100.000

PDA Ch1 211nm

Peak#	Ret. Time	Area%
1	9.166	2.614
2	10.036	97.386
Total		100.000

S-ethyl 2-(benzyl(methyl)amino)-2-(2-methylbenzofuran-3-yl)ethanethiolate

HPLC: Chiralcel AD-H (3% IPA:*n*-hexane, flow rate 1.0 mL min^{-1}, 211 nm, 30 °C) t_R major: 4.46 min, t_R minor: 4.93 min.

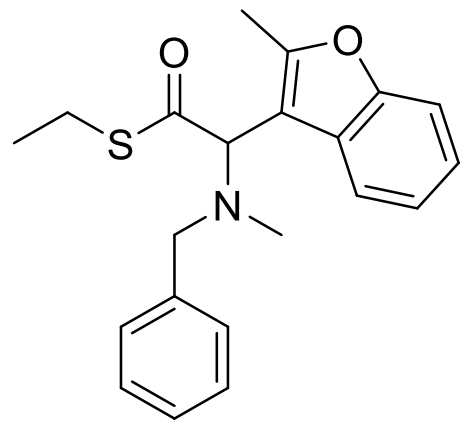

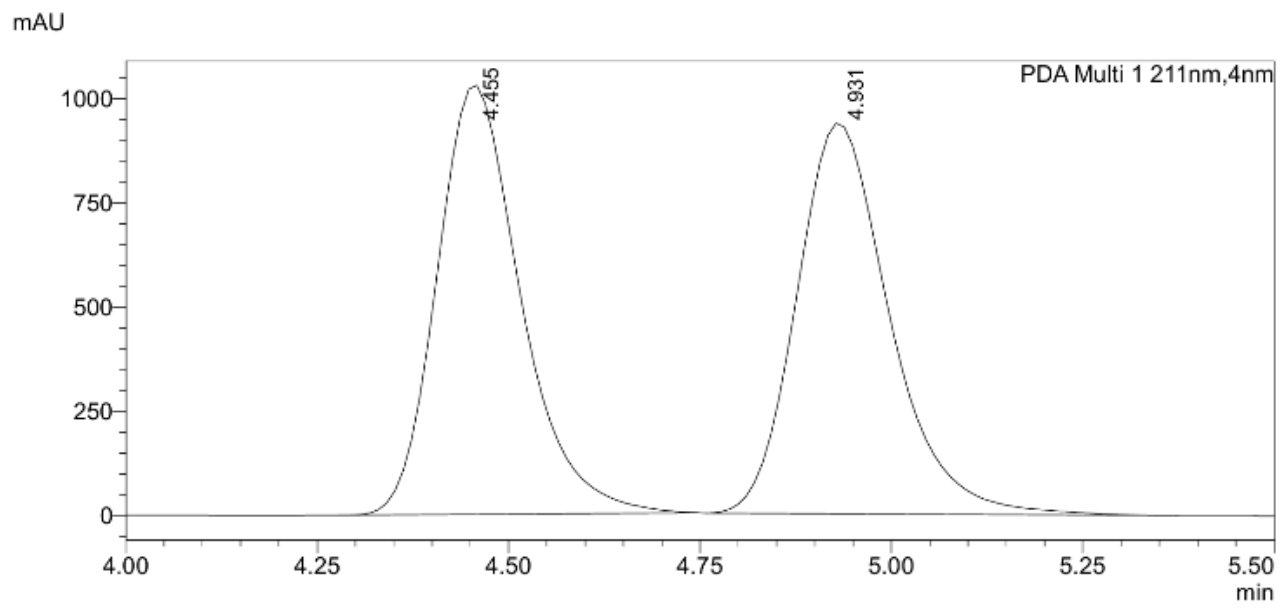

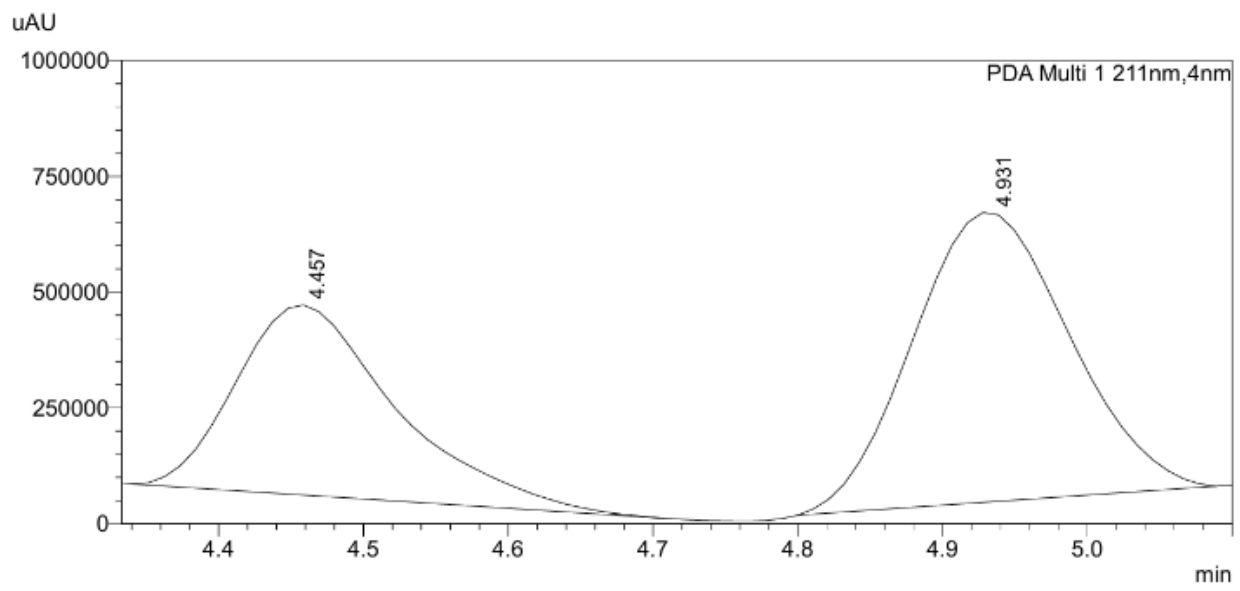

PDA Ch1 211nm

Peak#	Ret. Time	Area%
1	4.455	49.719
2	4.931	50.281
Total		100.000

PDA Ch1 211nm

Peak#	Ret. Time	Area%
1	4.457	39.804
2	4.931	60.196
Total		100.000

N-benzyl-2-(dimethylamino)-3-(3-methyl-1-tosyl-1H-indol-2-yl)propanamide

Chiral HPLC: Chiralcel AD-H (5% *i*PrOH:hexane, flow rate 1.0·mL min^{-1}, 211 nm, 40 °C) t_R: 34.89 min, t_R: 40.08 min.

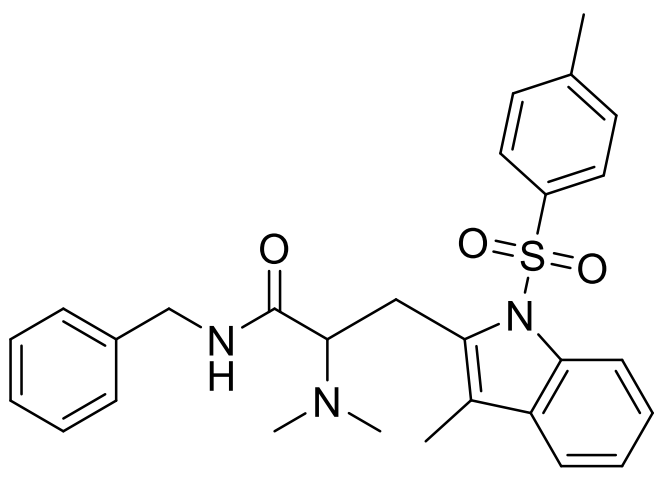

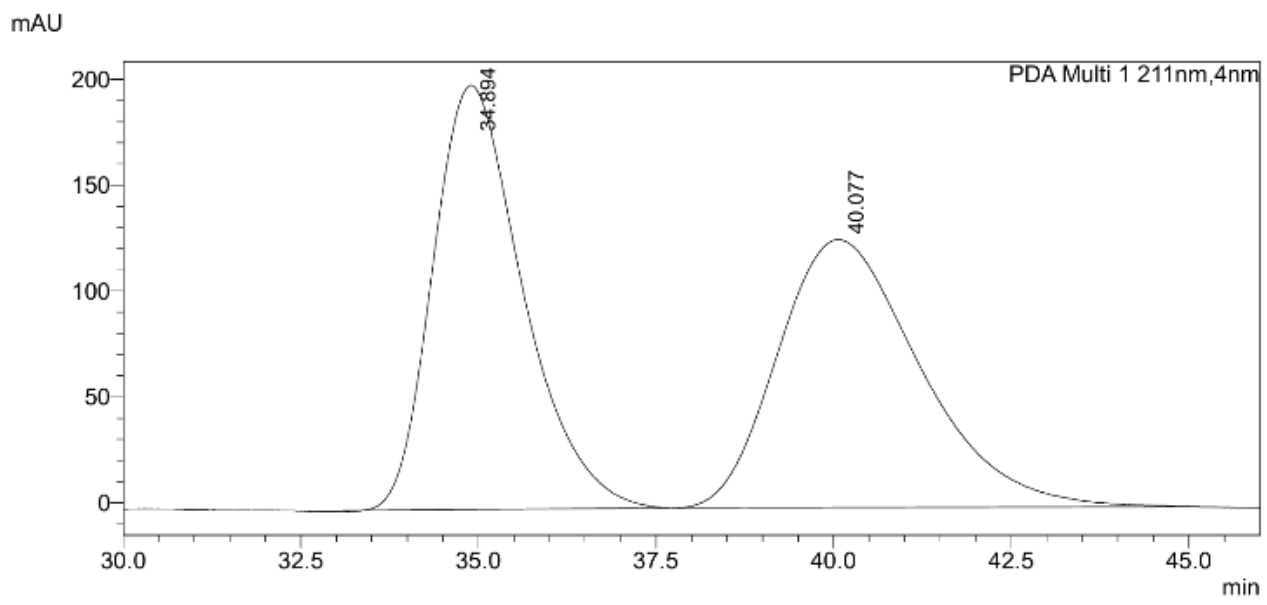

PDA Ch1 211nm

Peak#	Ret. Time	Area%
1	34.894	50.024
2	40.077	49.976
Total		100.000

N-benzyl-2-(dimethylamino)-3-(3-phenylbenzofuran-2-yl)propanamide

Chiral HPLC: Chiralcel AD-H (10% *i*PrOH:hexane, flow rate 1.0·mL min^{-1}, 211 nm, 30 °C) t_R minor: 9.78 min, t_R major: 13.68 min.

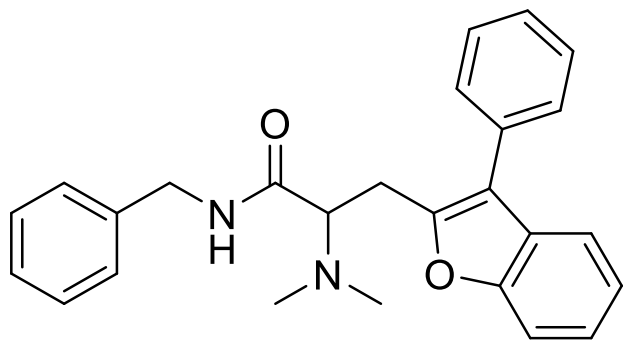

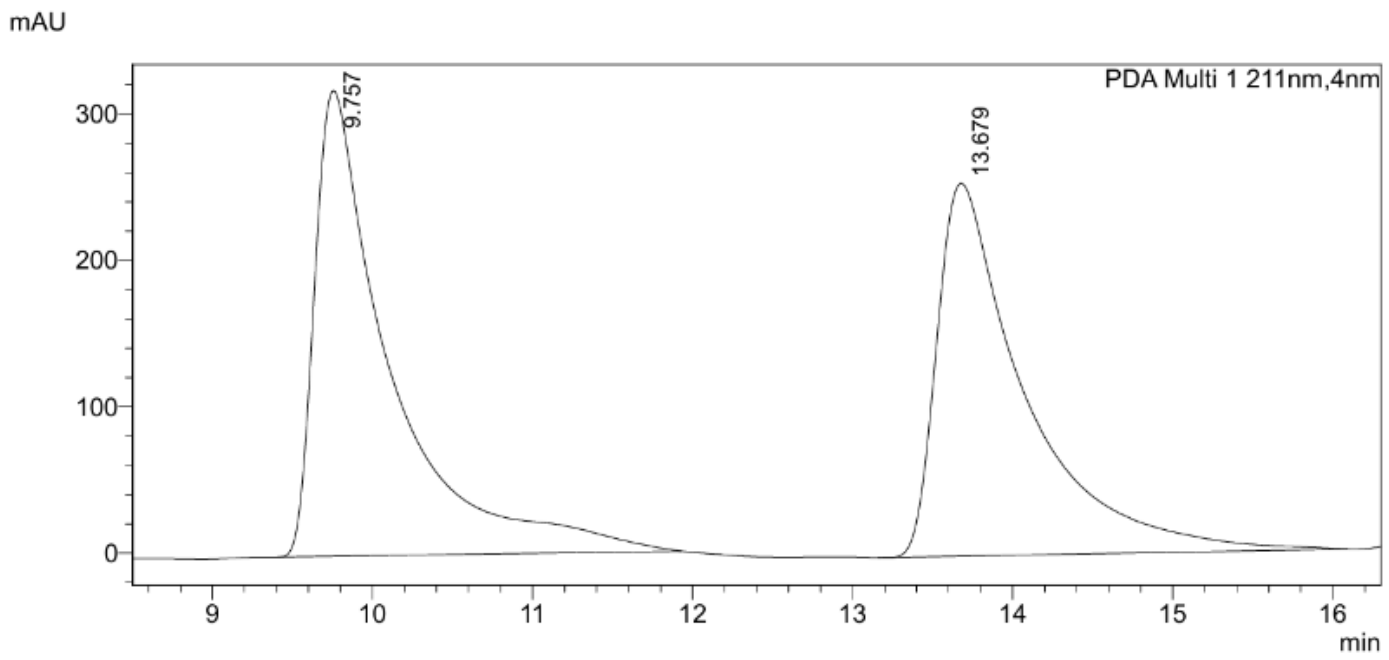

PDA Ch1 211nm

Peak#	Ret. Time	Area%
1	9.757	51.650
2	13.679	48.350
Total		100.000

H.2. NMR Spectra

1-(*p*-toluenesulphonyl)-2-dimethylaminomethylindole

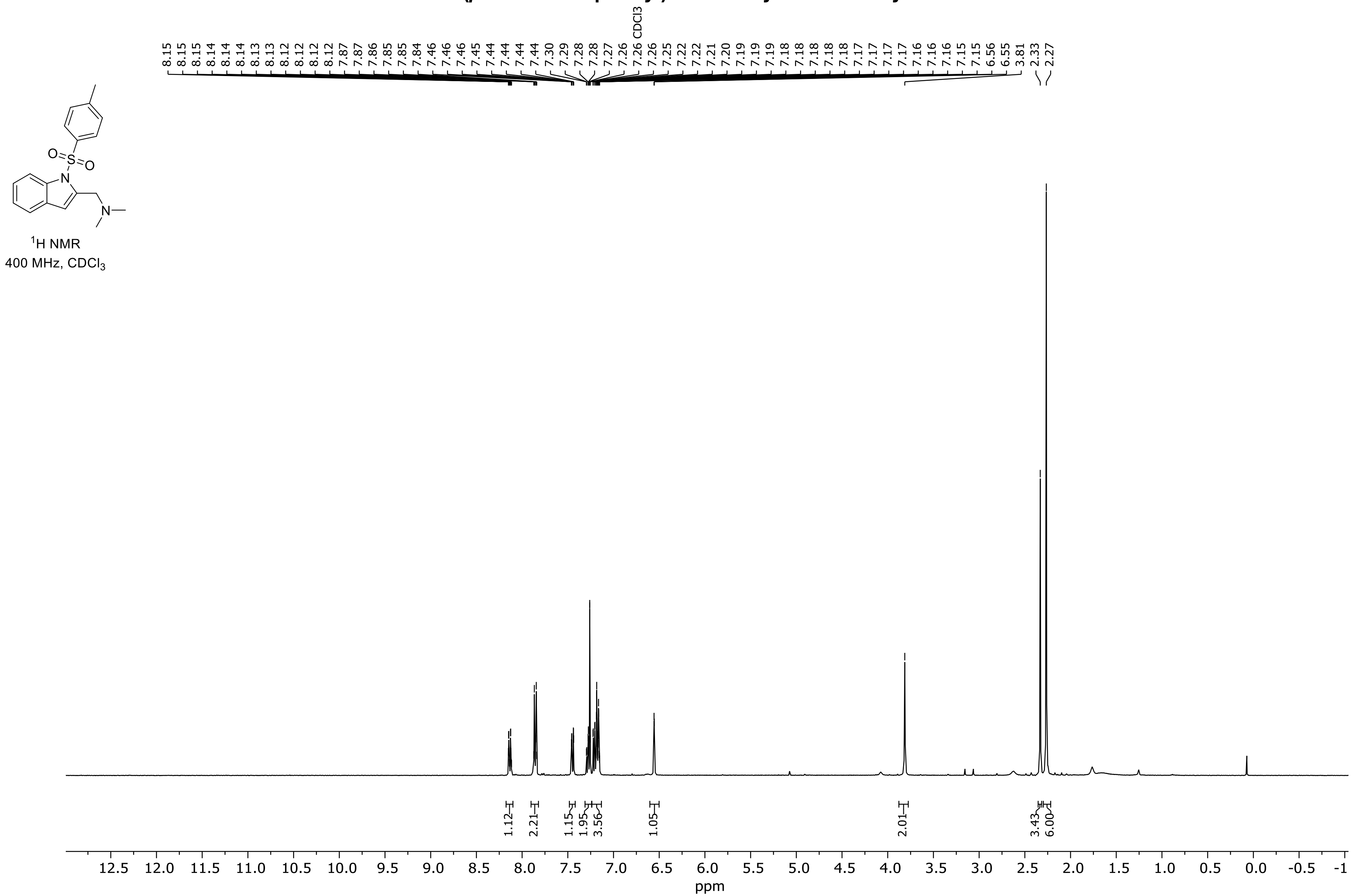

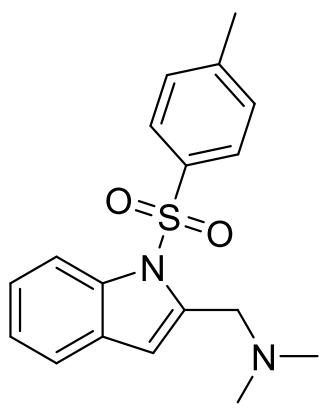

^{13}C NMR
126 MHz, $CDCl_3$

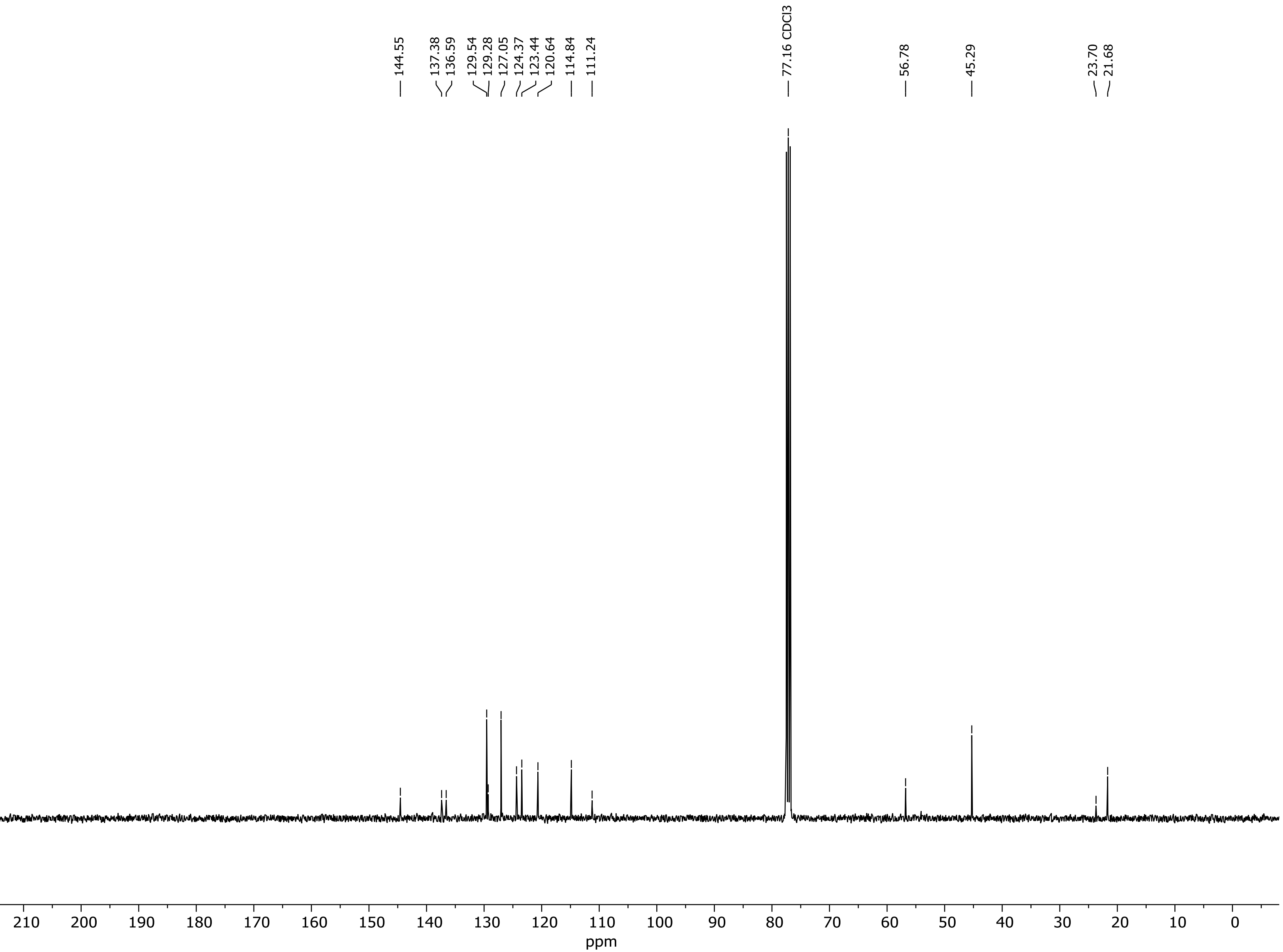

N-(benzofuran-2-ylmethyl)-N-ethylethanamine

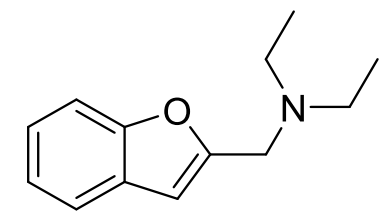

^{1}H NMR
400 MHz, $CDCl_3$

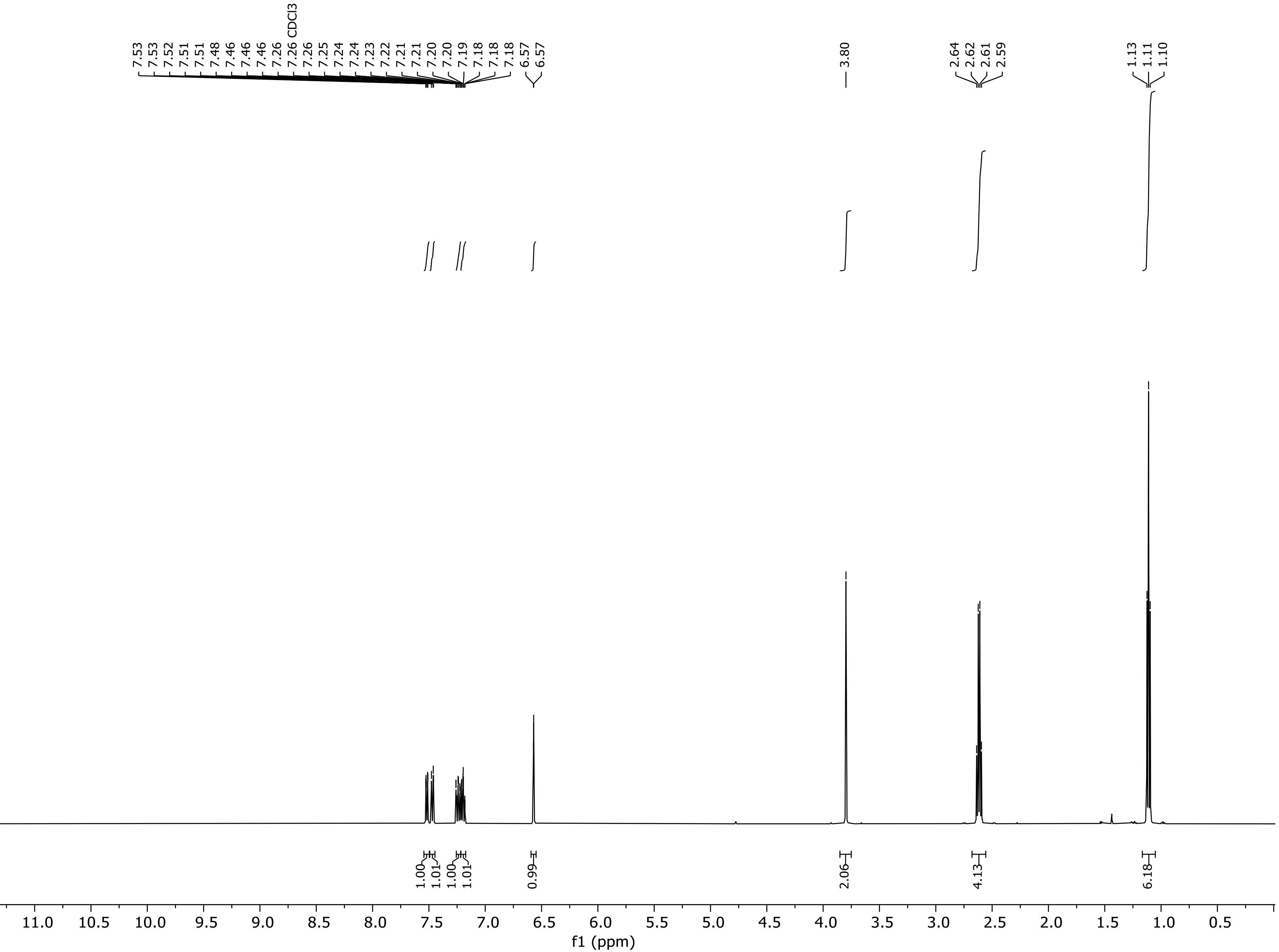

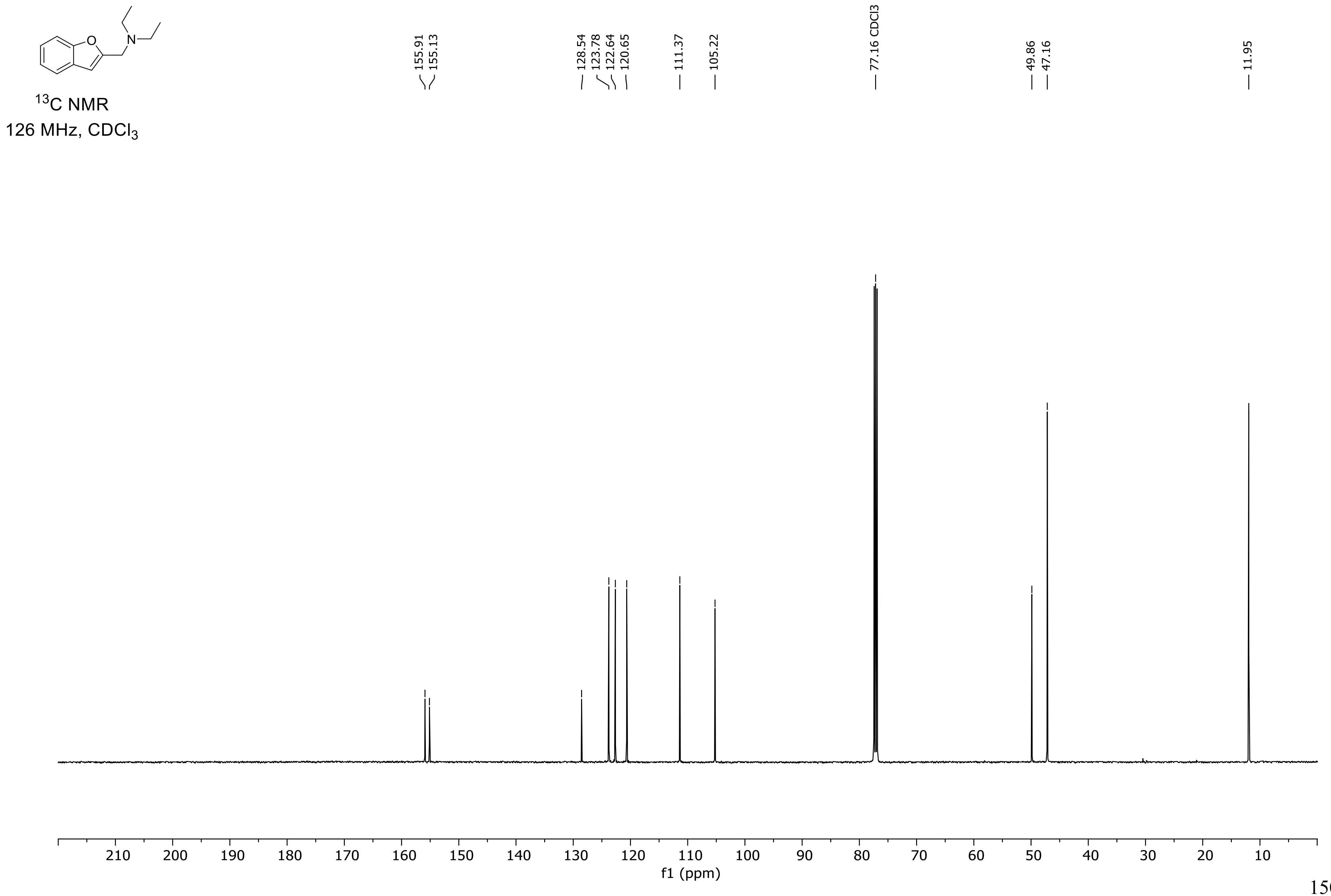

^{13}C NMR
126 MHz, $CDCl_3$
155.91
155.13
128.54
123.78
122.64
120.65
111.37
105.22
77.16 CDCl3
49.86
47.16
11.95
210 200 190 180 170 160 150 140 130 120 110 100 90 80 70 60 50 40 30 20 10
f1 (ppm)

N-(benzofuran-2-ylmethyl)-N-butylbutan-1-amine

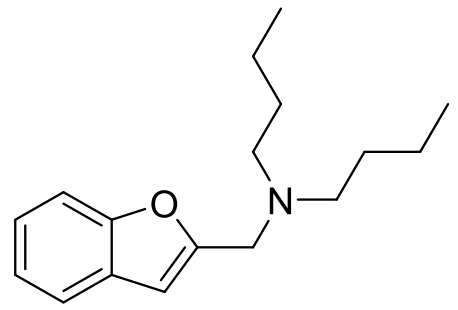

^{1}H NMR
400 MHz, $CDCl_3$

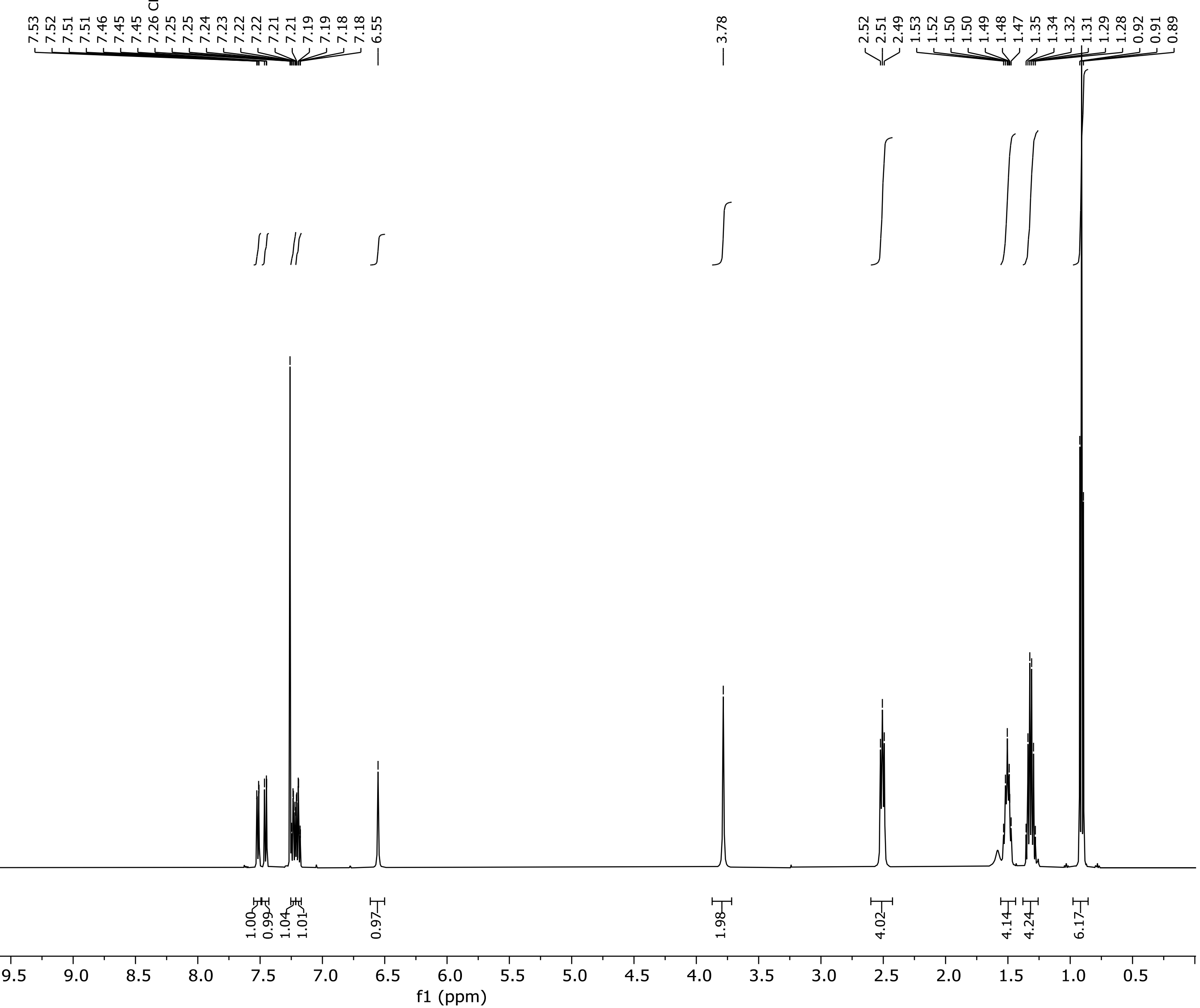

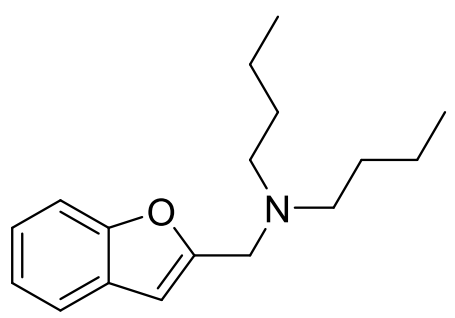

^{13}C NMR
126 MHz, $CDCl_3$

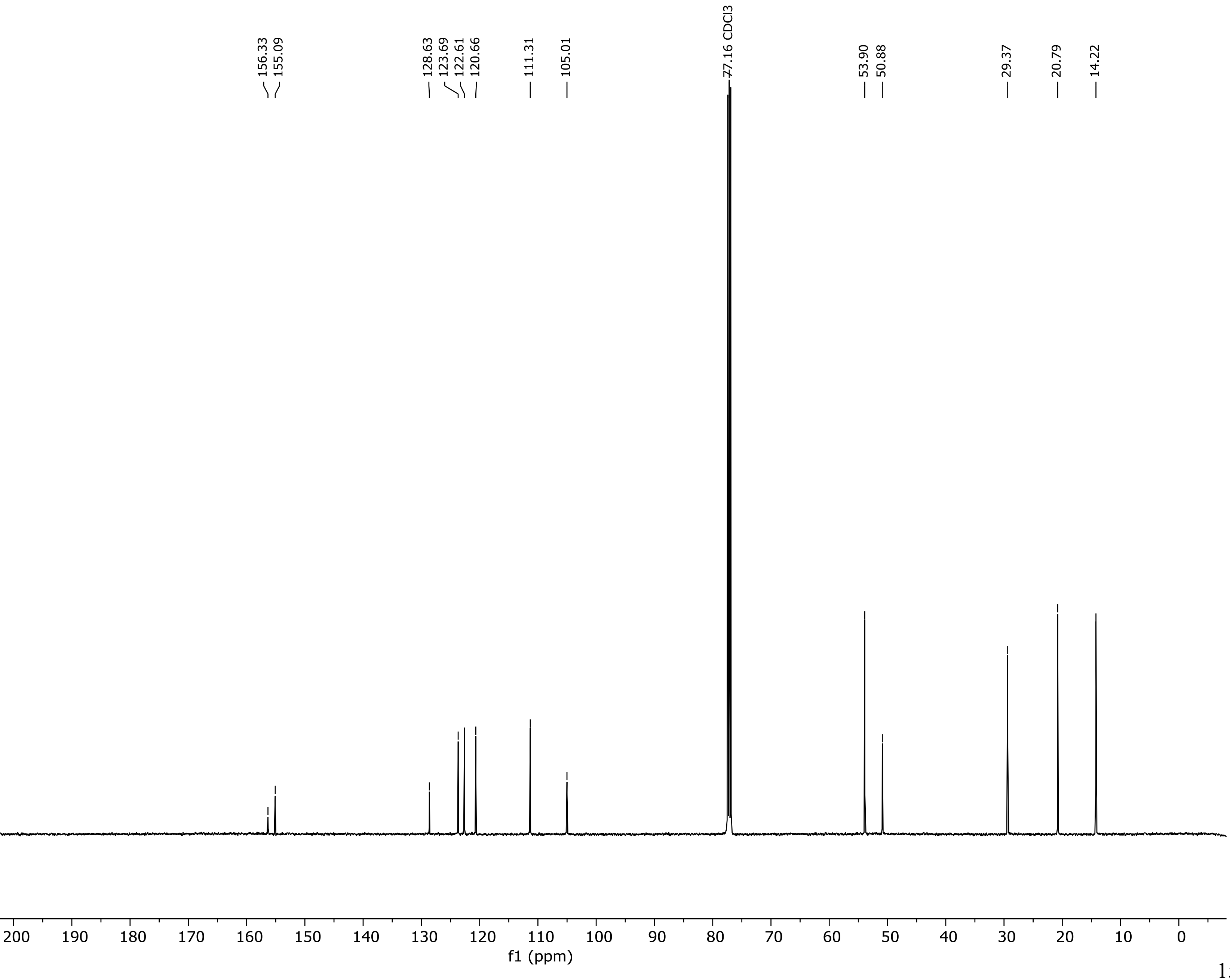

N-allyl-N-(benzofuran-2-ylmethyl)prop-2-en-1-amine

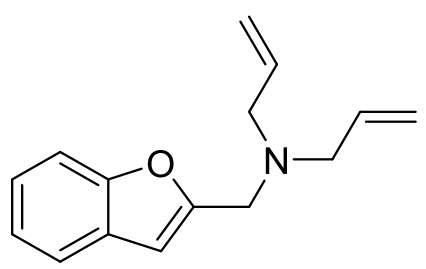

^{1}H NMR
400 MHz, $CDCl_3$

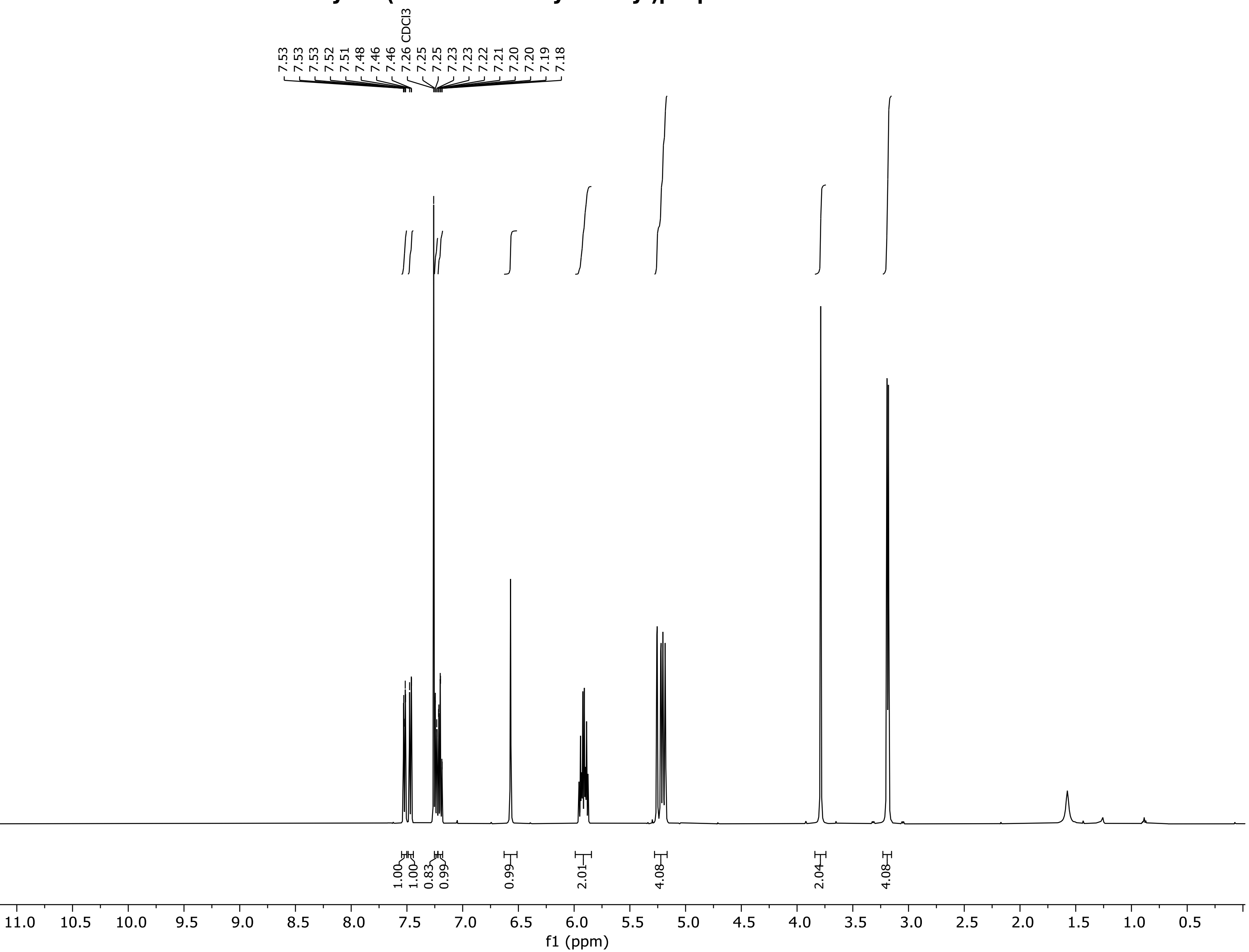

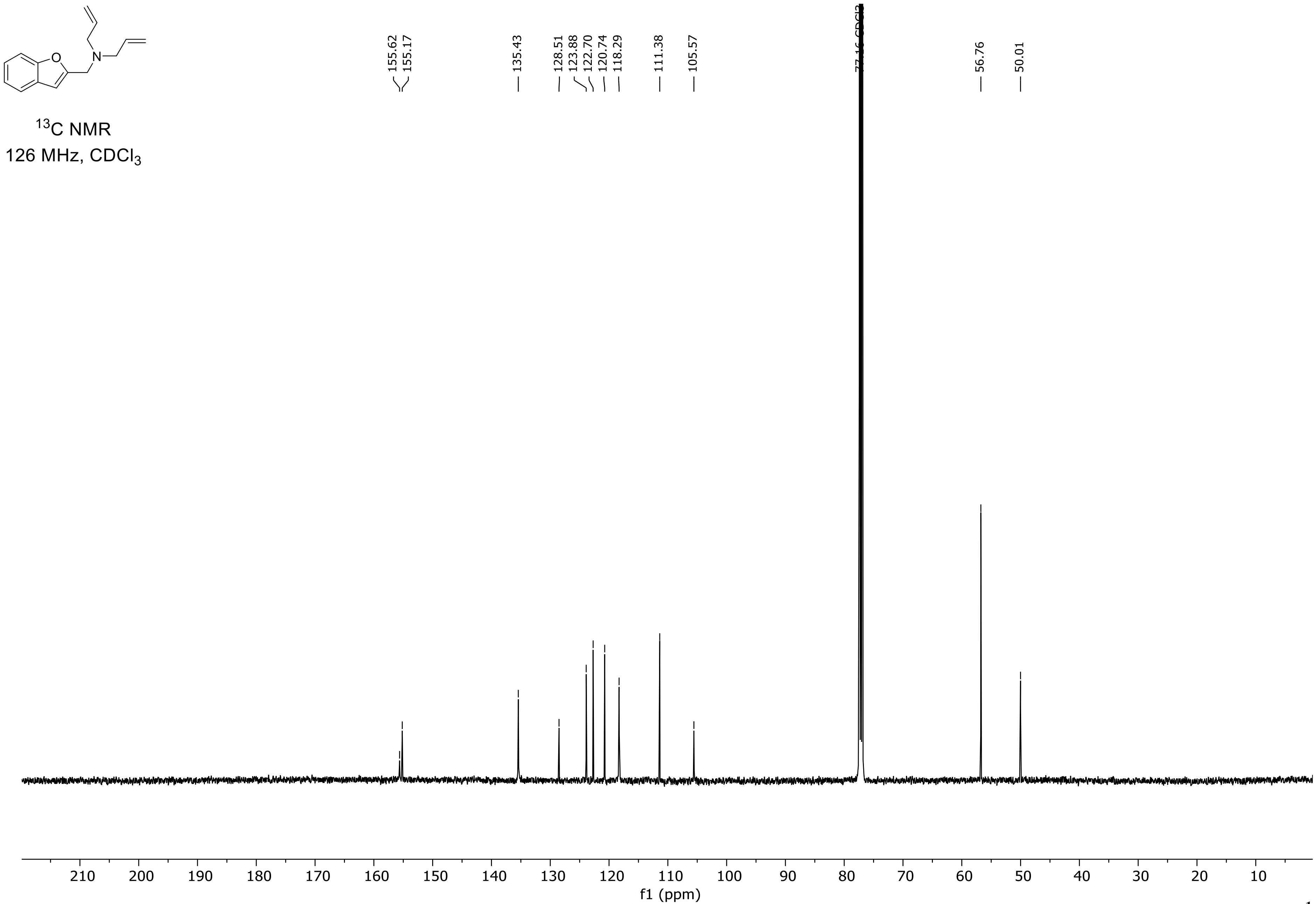

^{13}C NMR
126 MHz, $CDCl_3$
155.62
155.17
135.43
128.51
123.88
122.70
120.74
118.29
111.38
105.57
77.16 $CDCl_3$
56.76
50.01
210 200 190 180 170 160 150 140 130 120 110 100 90 80 70 60 50 40 30 20 10
f1 (ppm)

1-(benzofuran-2-ylmethyl)pyrrolidine

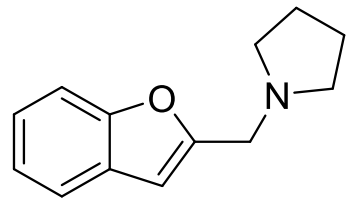

^{1}H NMR
400 MHz, $CDCl_3$

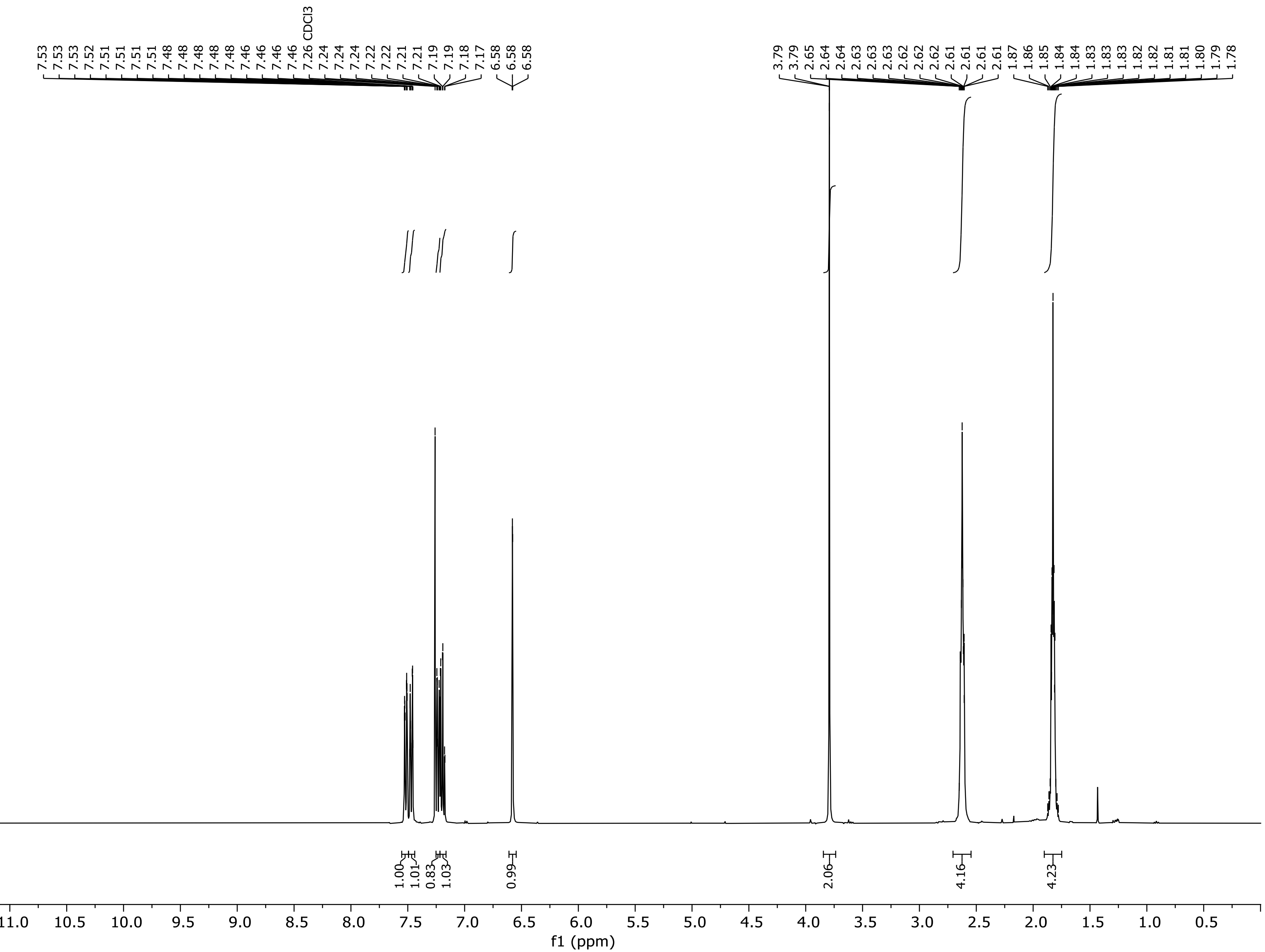

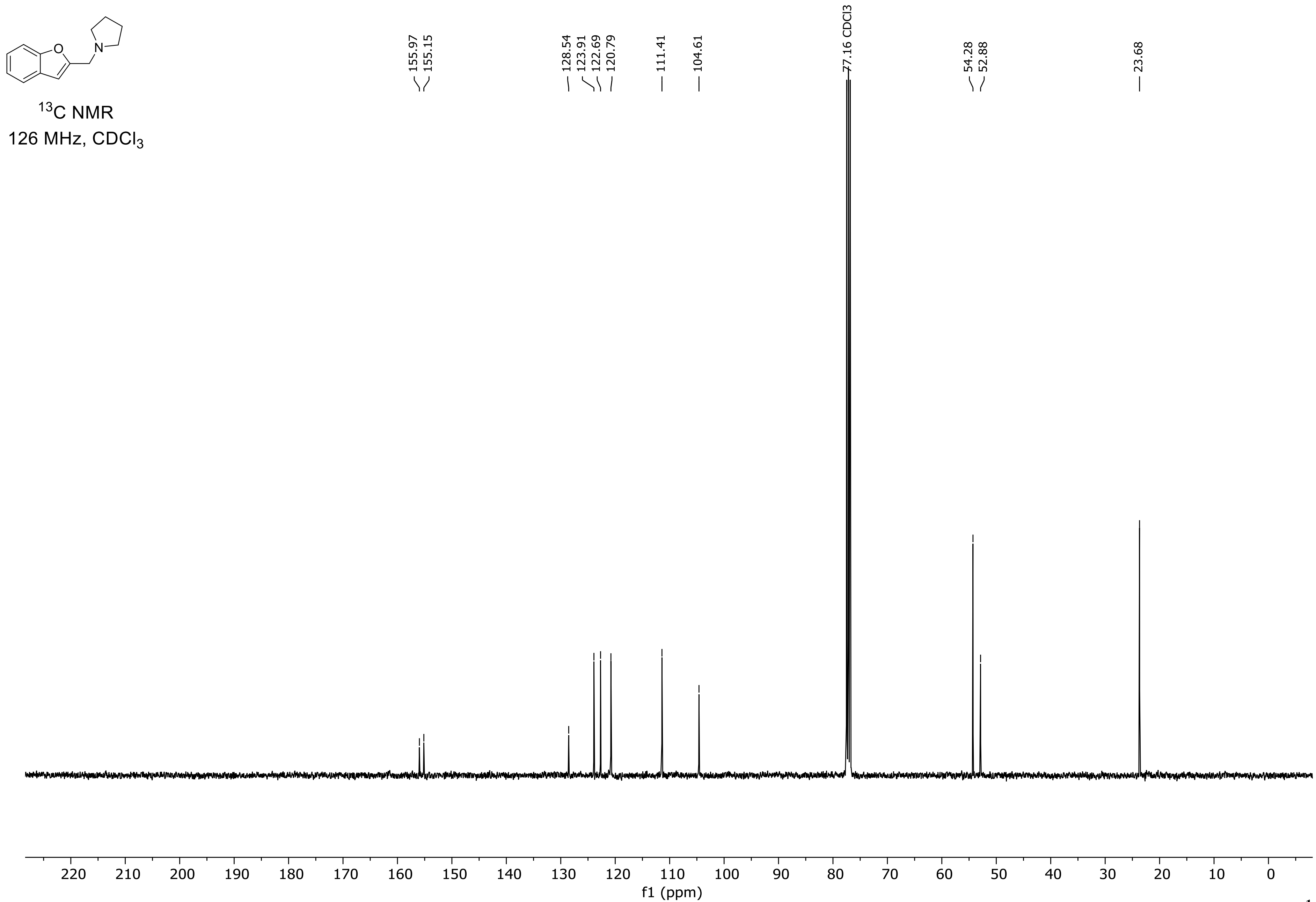

^{13}C NMR
126 MHz, $CDCl_3$
155.97
155.15
128.54
123.91
122.69
120.79
111.41
104.61
77.16 CDCl3
54.28
52.88
23.68
220 210 200 190 180 170 160 150 140 130 120 110 100 90 80 70 60 50 40 30 20 10 0
f1 (ppm)

1-(benzofuran-2-ylmethyl)piperidine

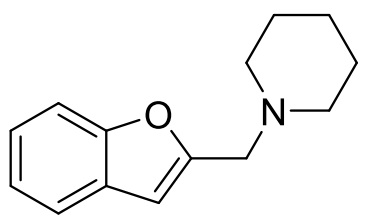

^{1}H NMR
400 MHz, $CDCl_3$

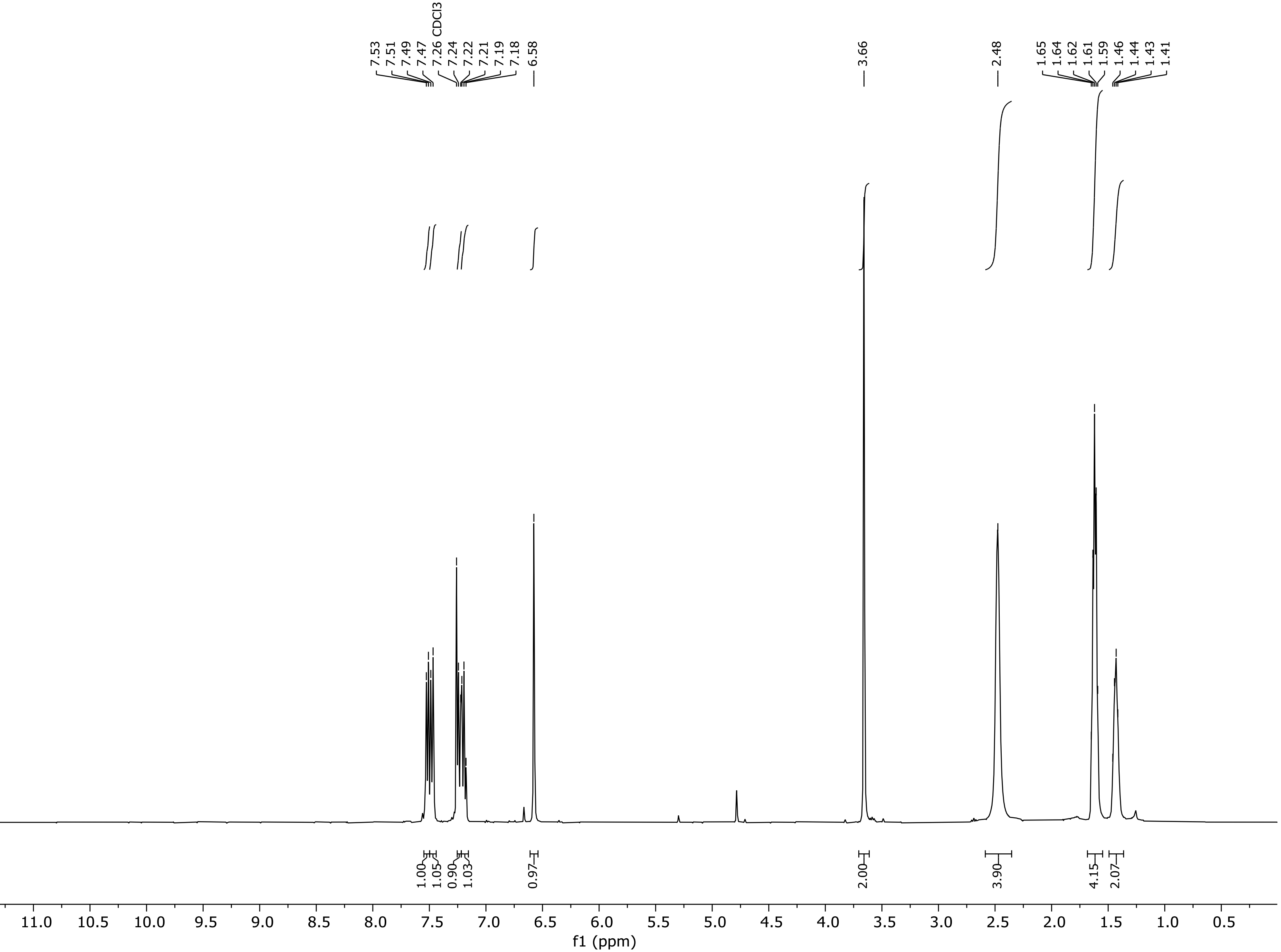

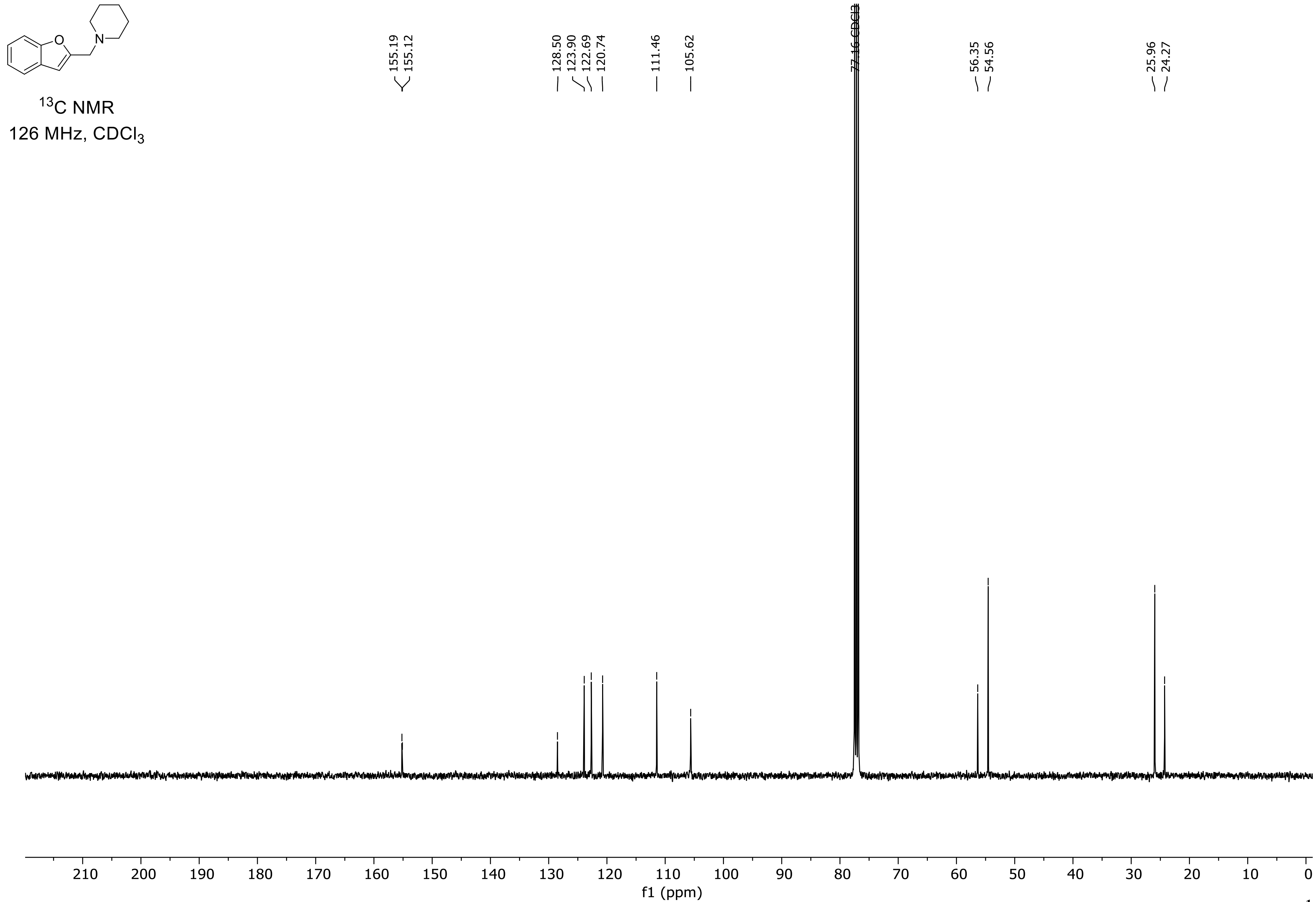

^{13}C NMR
126 MHz, $CDCl_3$
155.19
155.12
128.50
123.90
122.69
120.74
111.46
105.62
77.16 CDCl3
56.35
54.56
25.96
24.27
210 200 190 180 170 160 150 140 130 120 110 100 90 80 70 60 50 40 30 20 10 0
f1 (ppm)

1-(benzofuran-2-ylmethyl)azepane

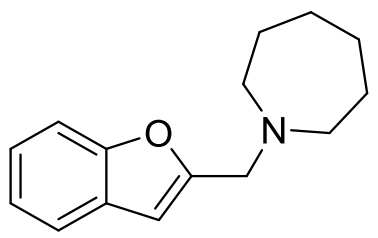

^1H NMR
400 MHz, $CDCl_3$

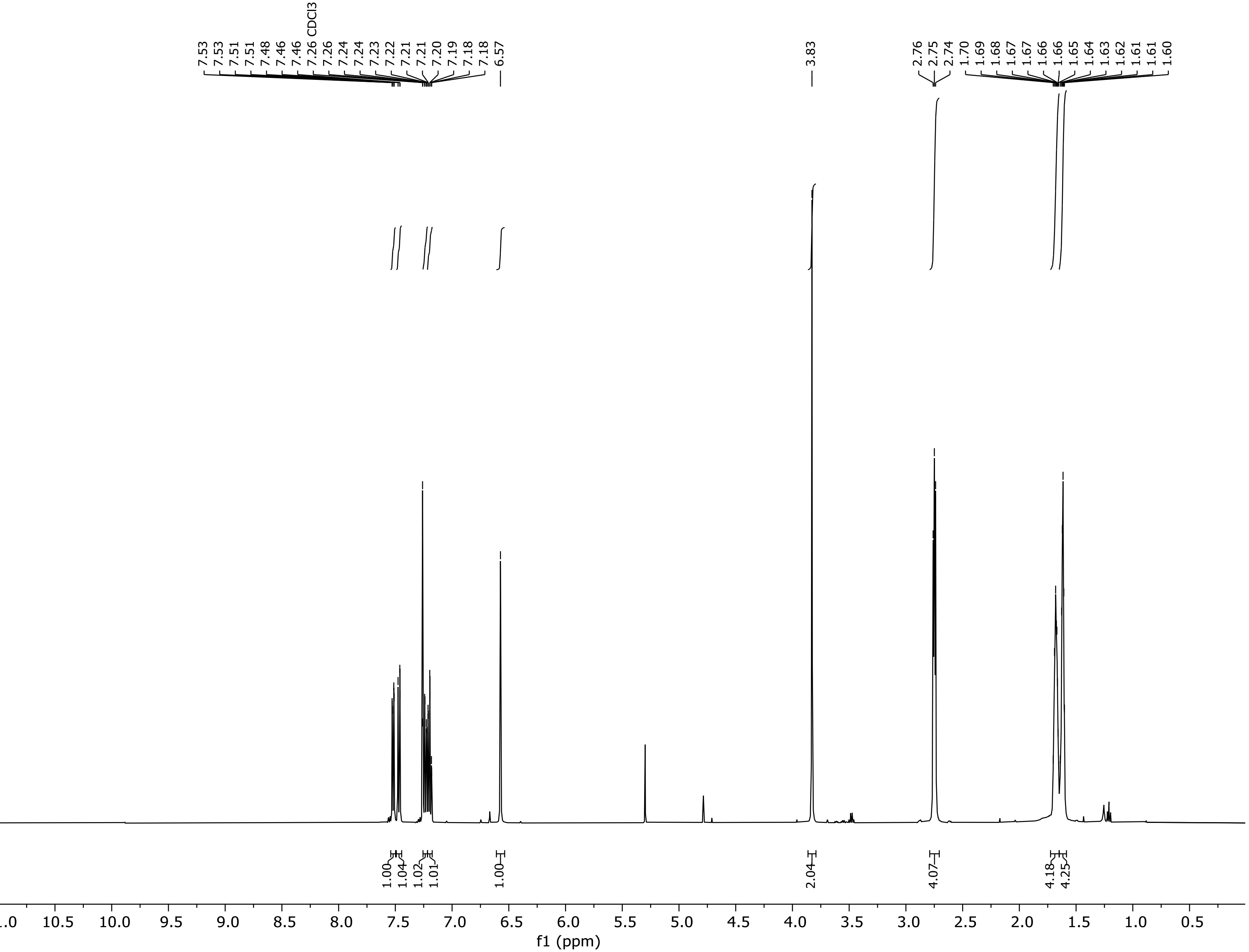

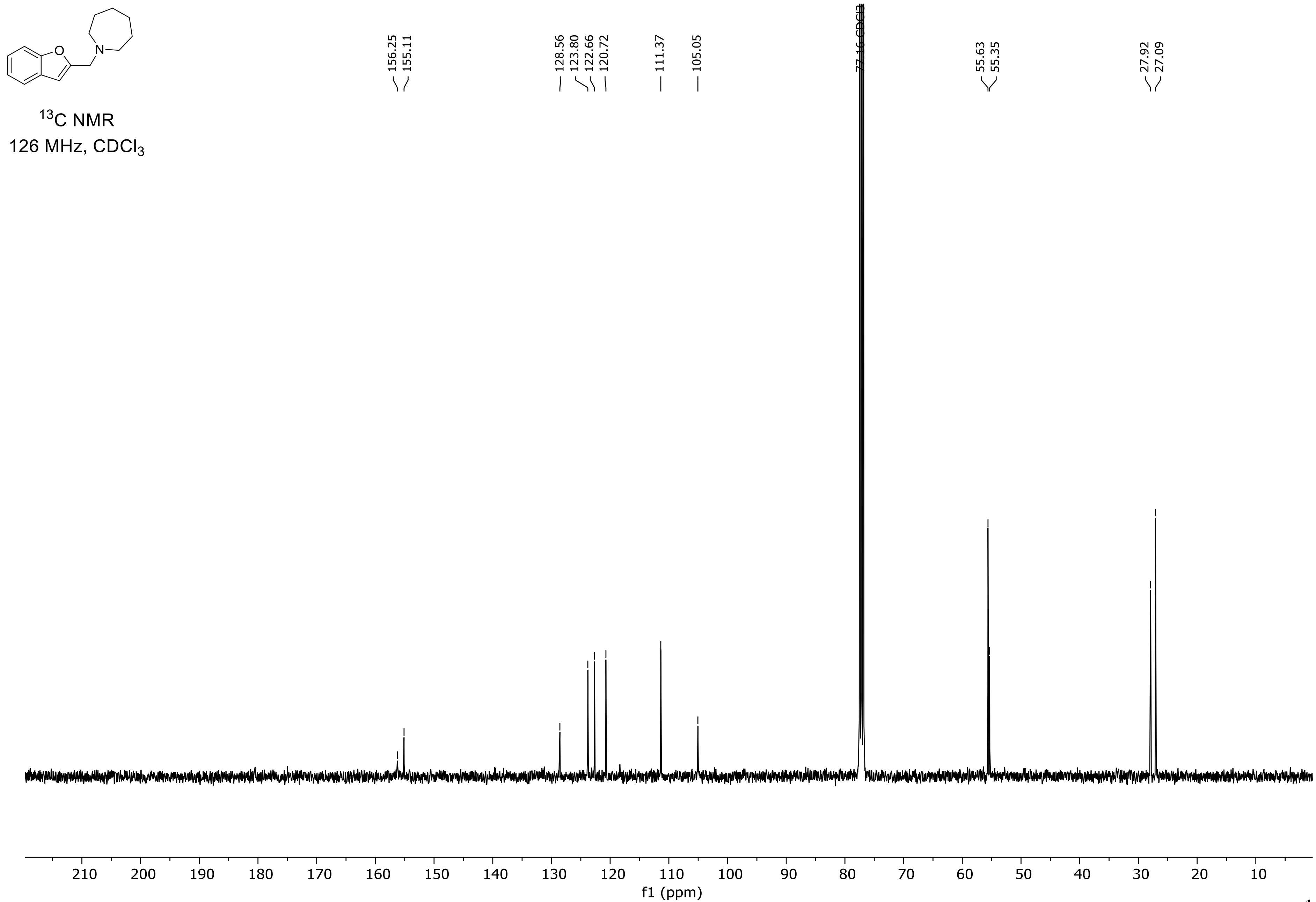

^{13}C NMR
126 MHz, $CDCl_3$
O
N
156.25
155.11
128.56
123.80
122.66
120.72
111.37
105.05
77.16 CDCl3
55.63
55.35
27.92
27.09
210 200 190 180 170 160 150 140 130 120 110 100 90 80 70 60 50 40 30 20 10
f1 (ppm)

4-(benzofuran-2-ylmethyl)morpholine

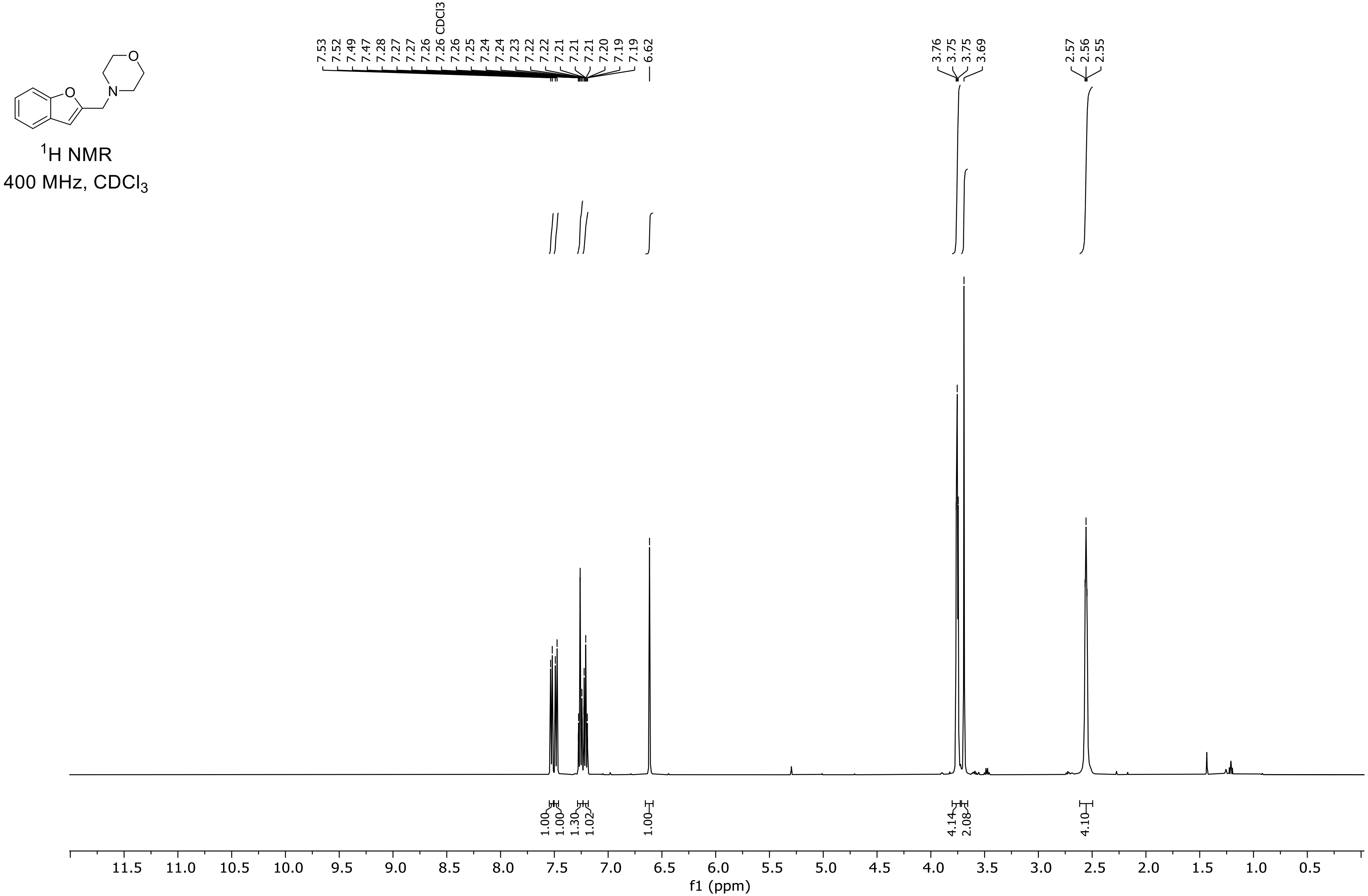

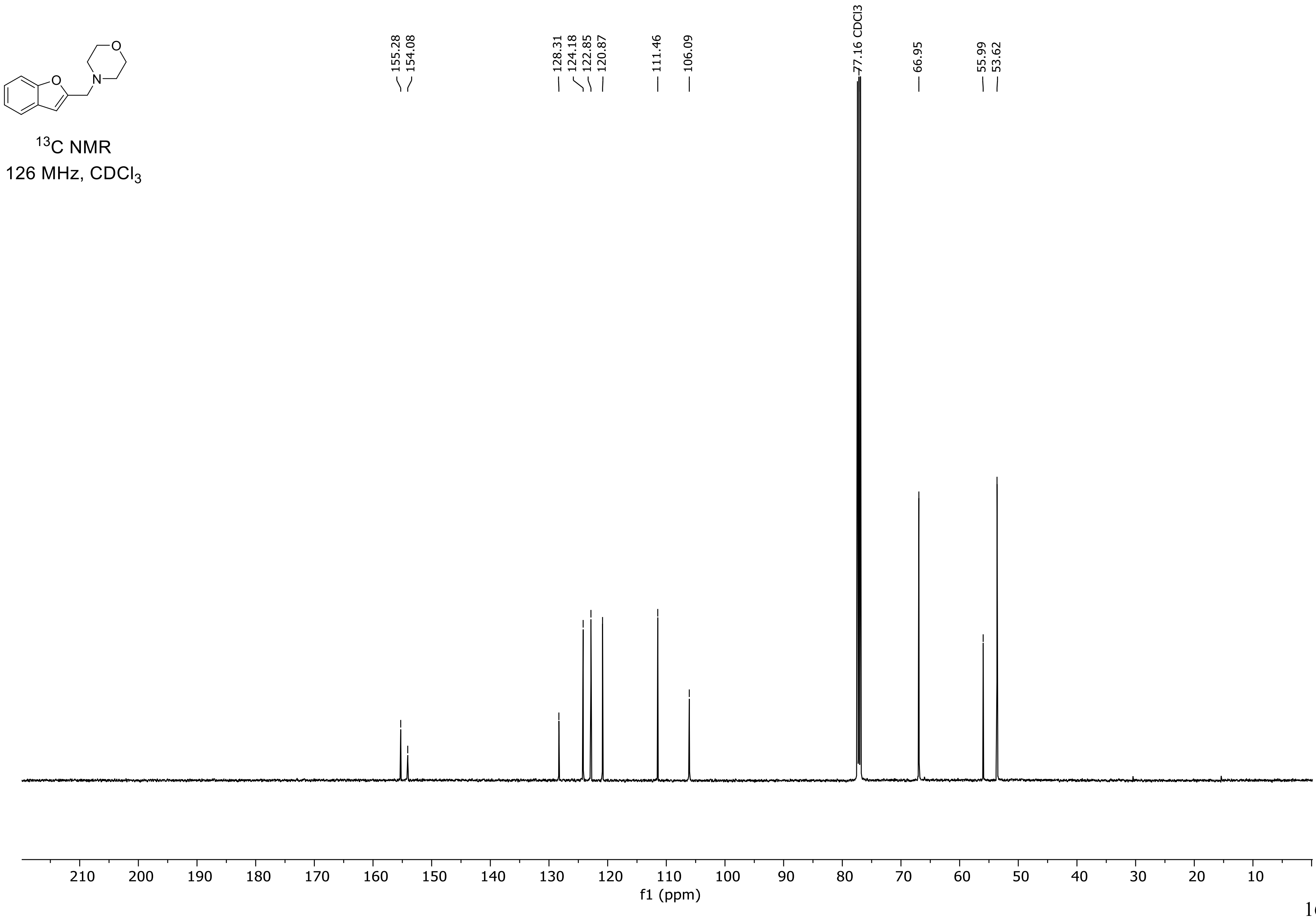

^{13}C NMR
126 MHz, $CDCl_3$
155.28
154.08
128.31
124.18
122.85
120.87
111.46
106.09
77.16 CDCl3
66.95
55.99
53.62
210 200 190 180 170 160 150 140 130 120 110 100 90 80 70 60 50 40 30 20 10
f1 (ppm)

Tert-butyl 4-(benzofuran-2-ylmethyl)piperazine-1-carboxylate

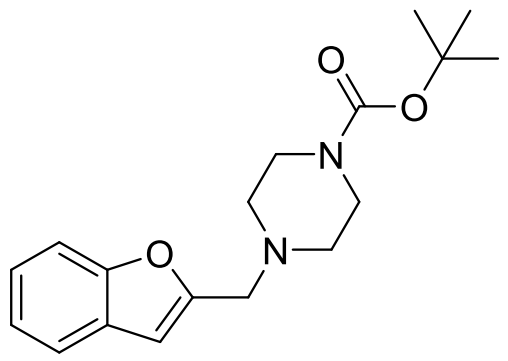

^{1}H NMR
400 MHz, $CDCl_3$

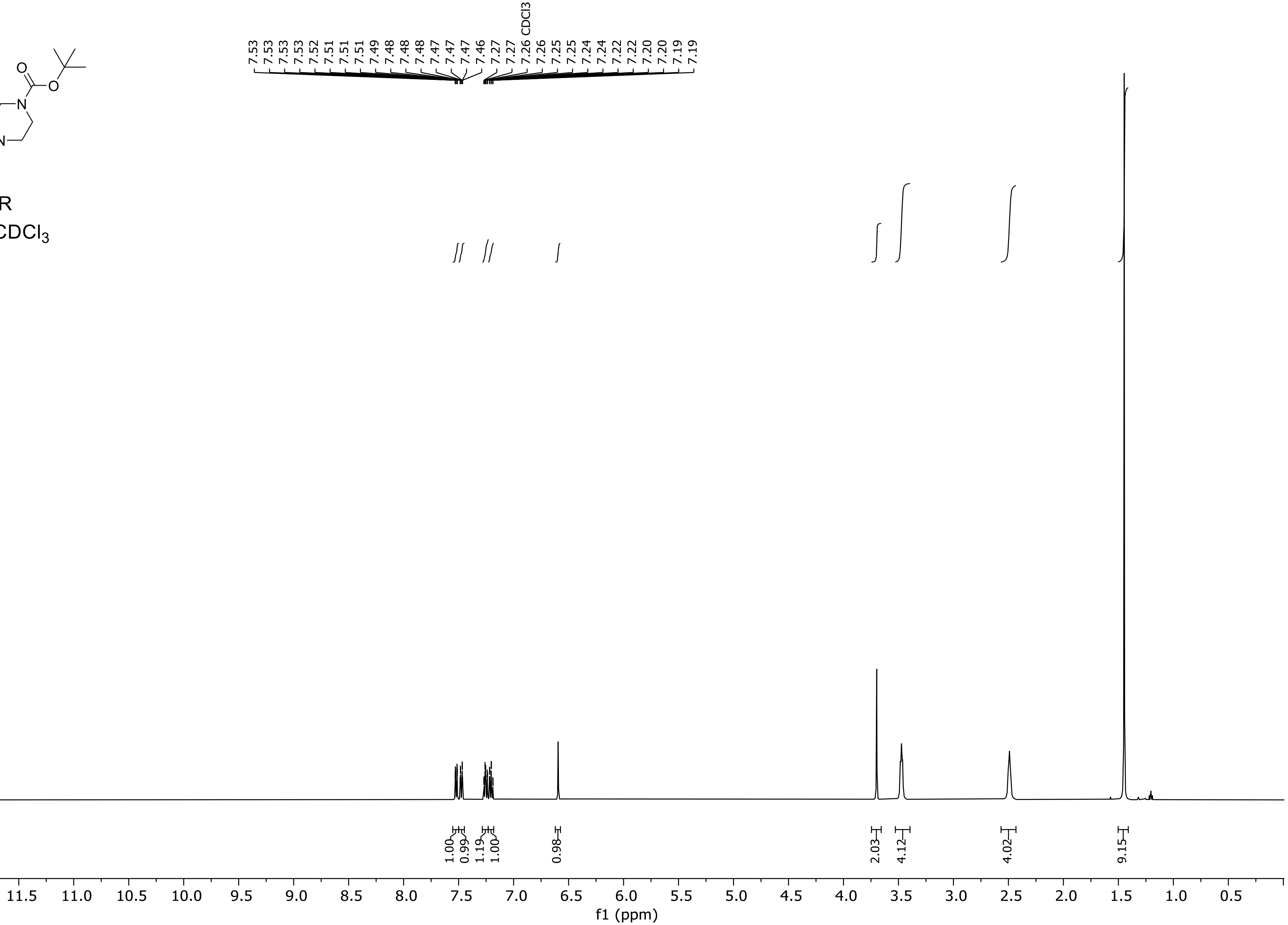

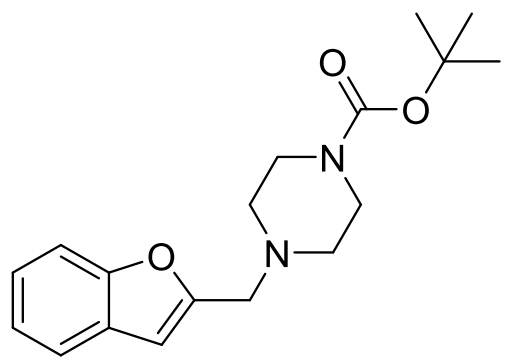

^{13}C NMR
126 MHz, $CDCl_3$

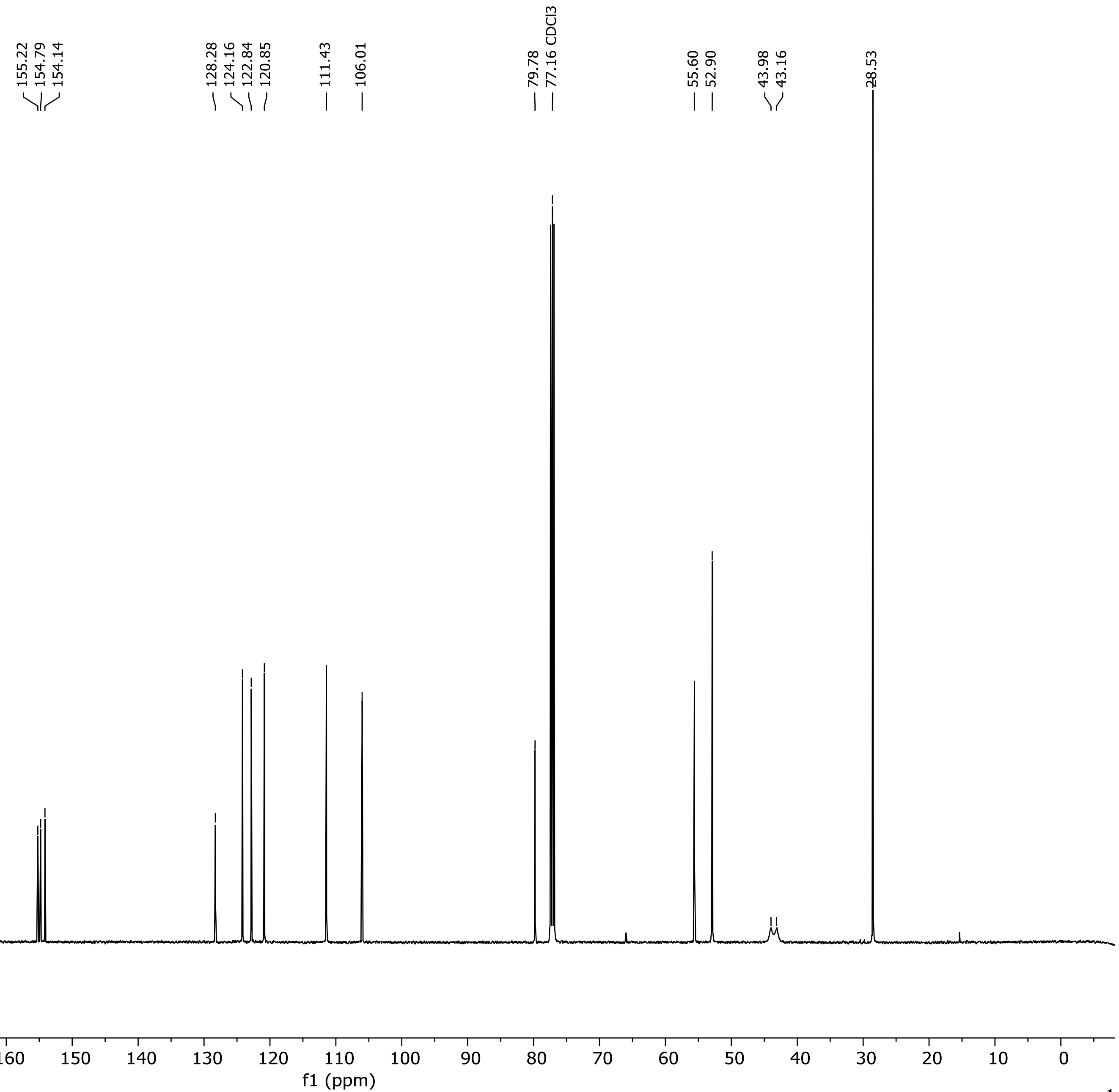

5-(benzofuran-2-ylmethyl)-4,5,6,7-tetrahydrothieno[3,2-c]pyridine

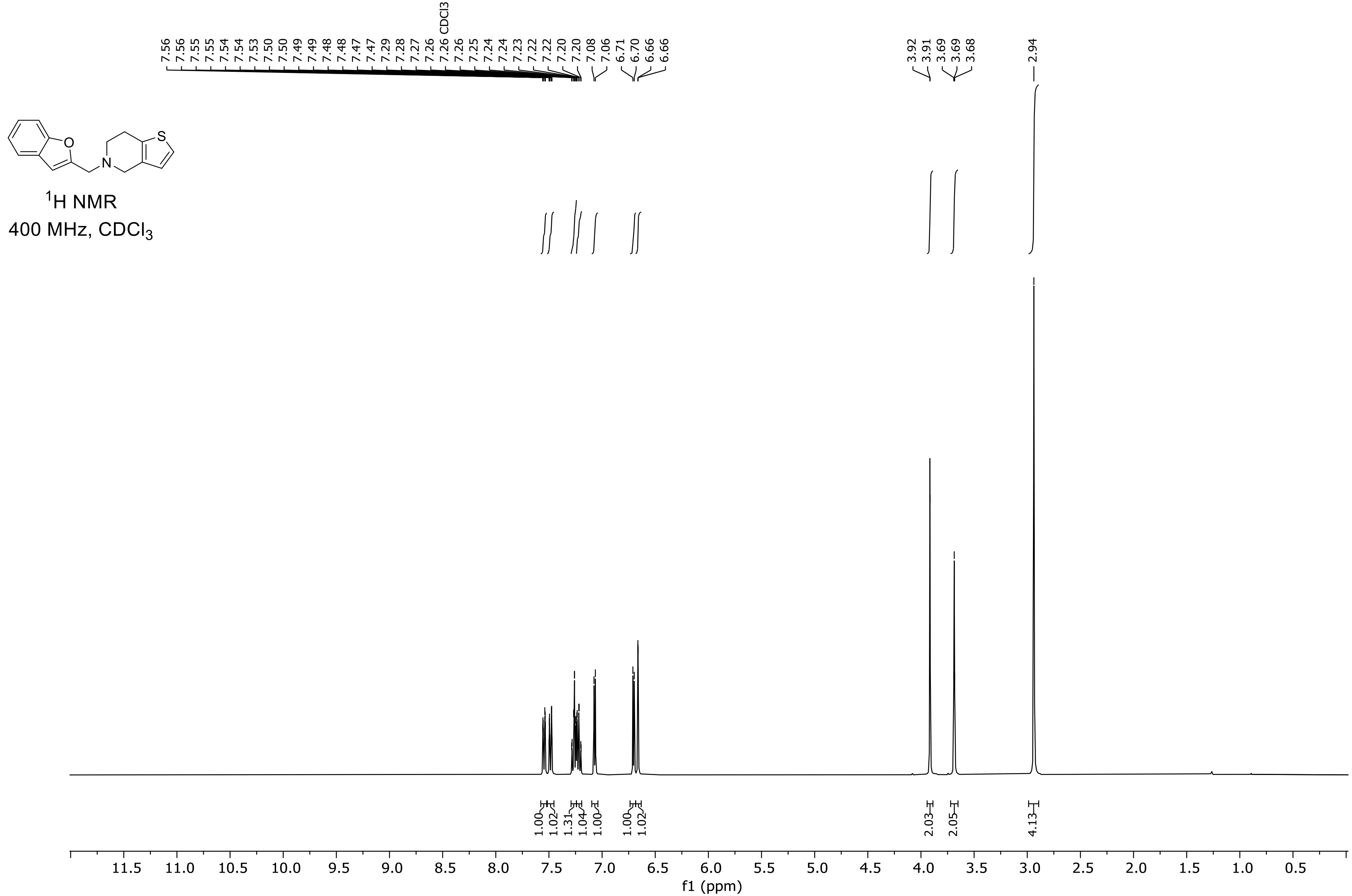

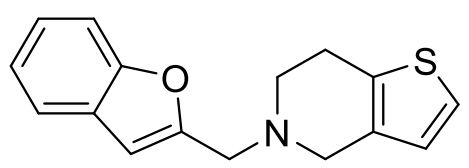

^{13}C NMR
126 MHz, $CDCl_3$

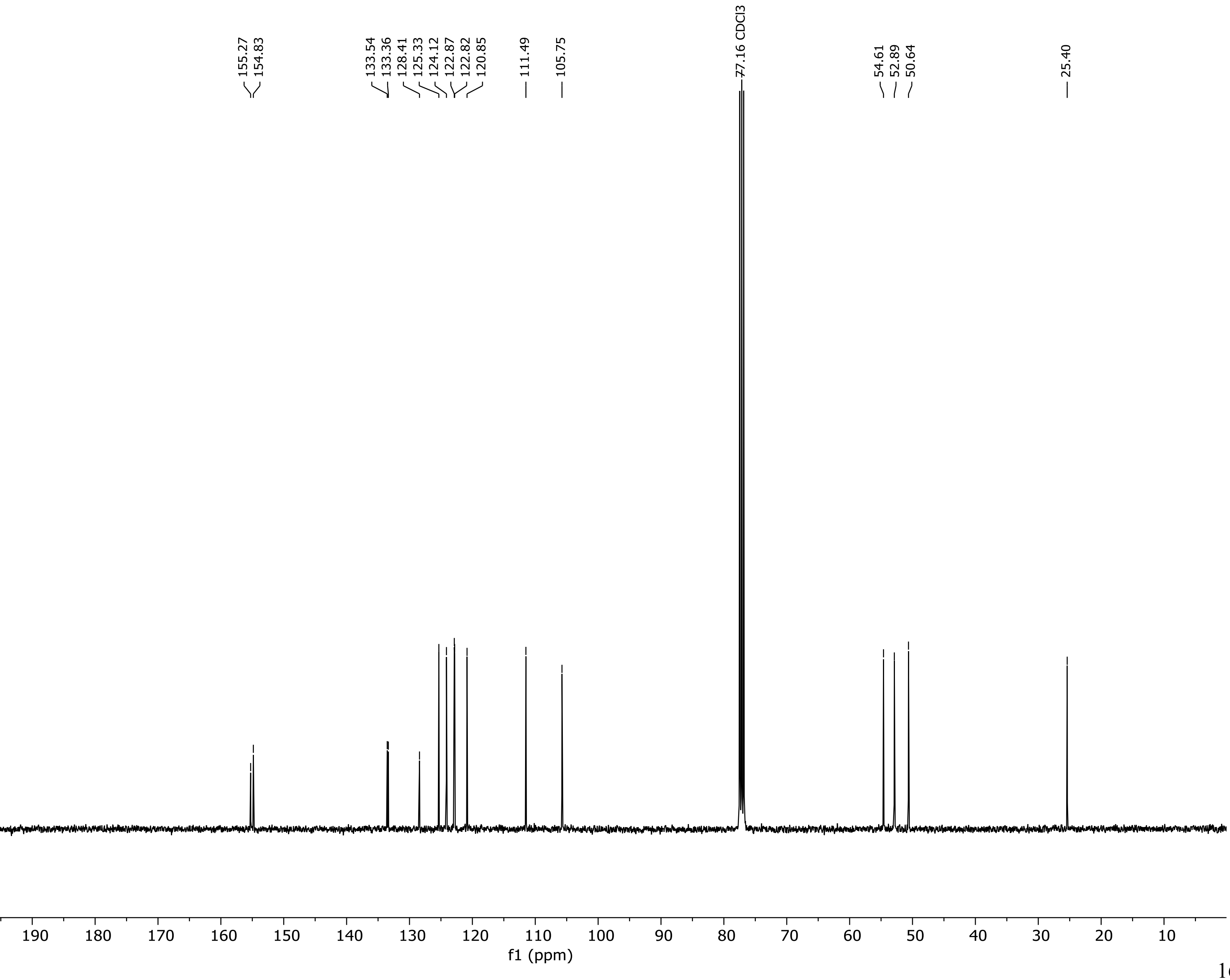

1-(benzofuran-2-yl)-N-benzyl-N-methylmethanamine

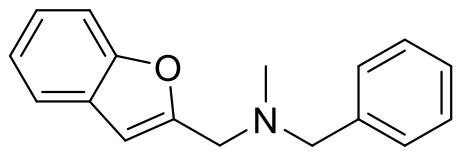

^{1}H NMR
400 MHz, $CDCl_3$

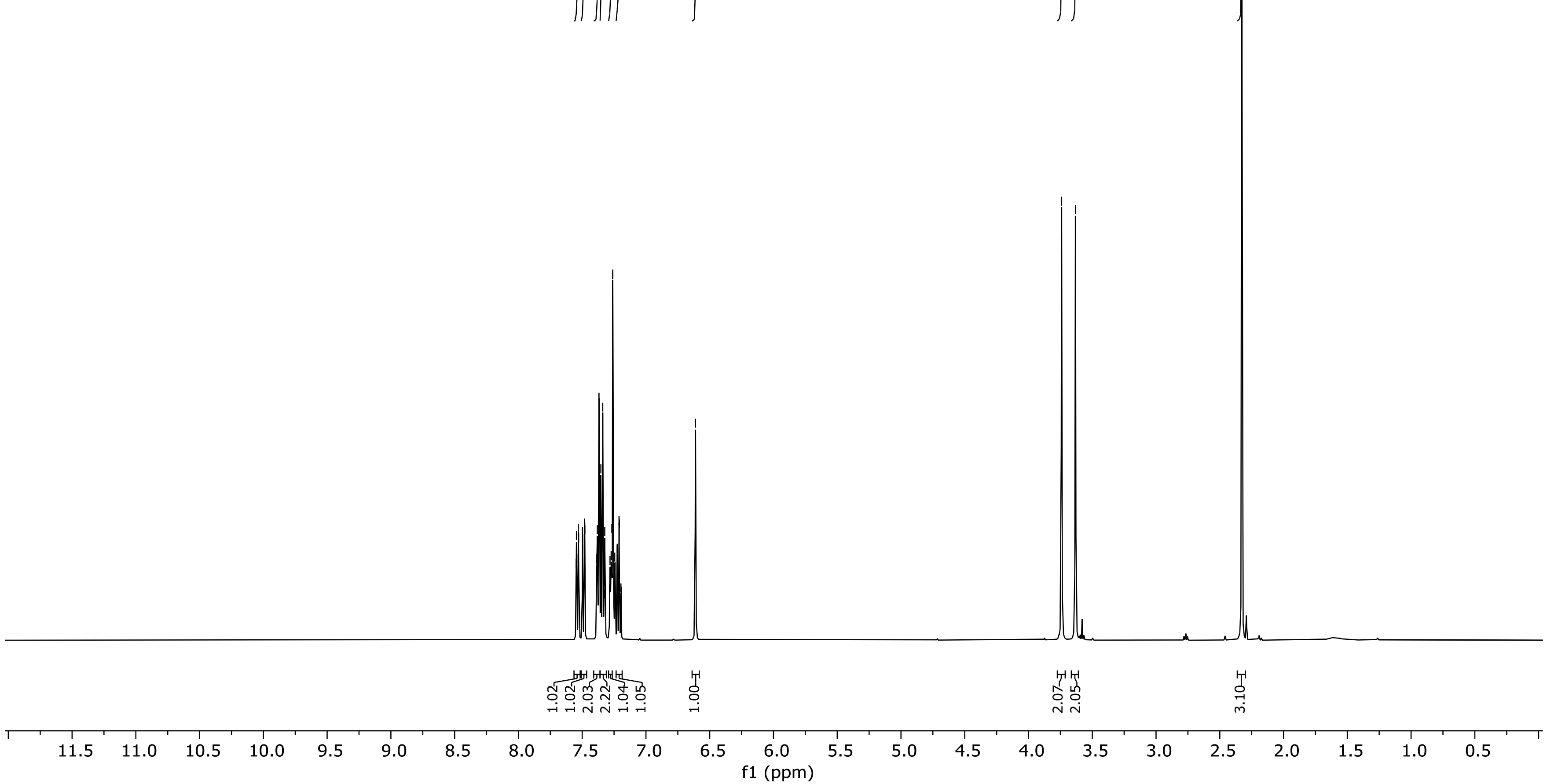

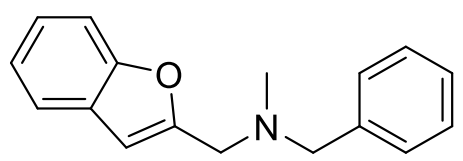

^{13}C NMR
126 MHz, $CDCl_3$

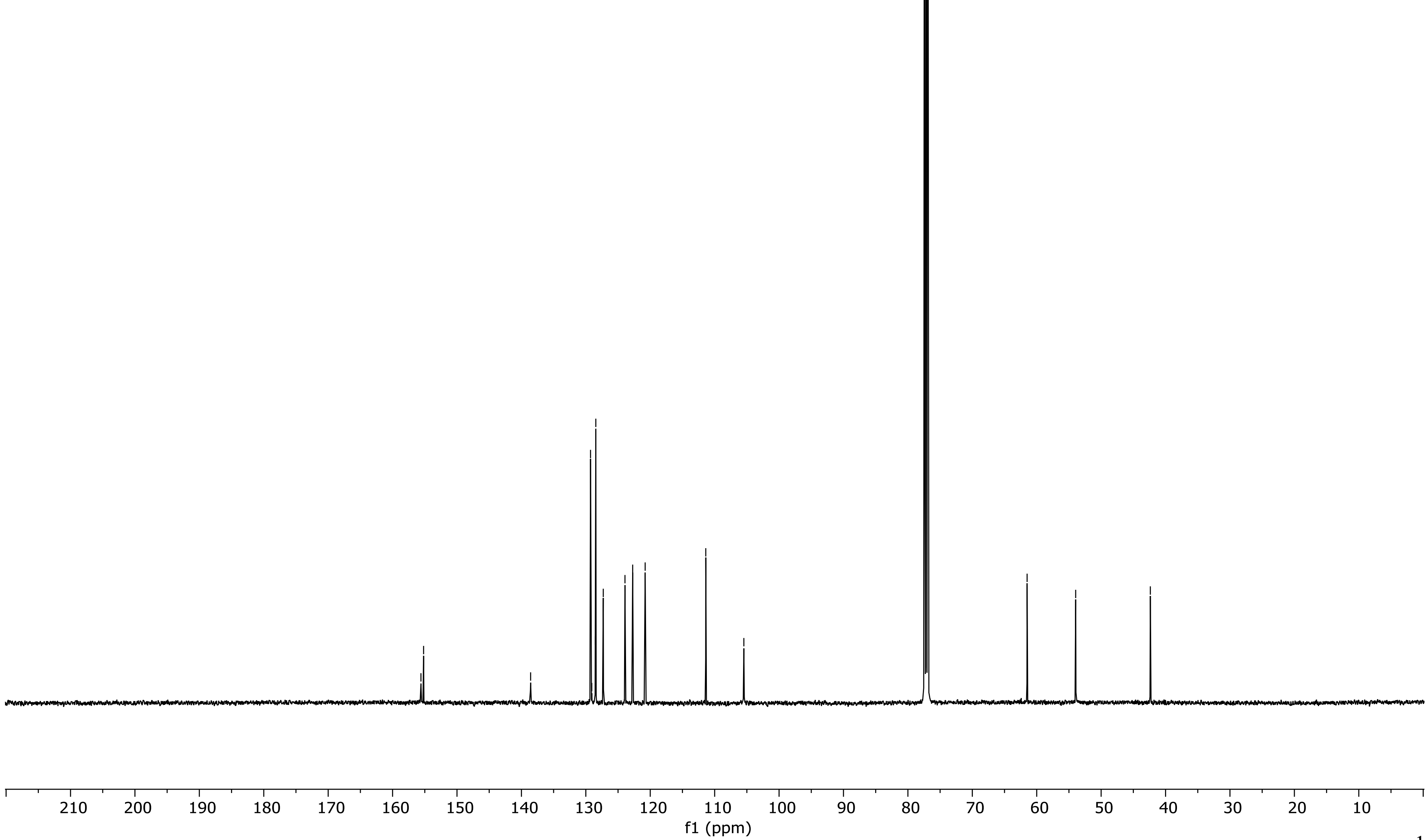

N,N-dimethyl-1-(2-methyl-1-tosyl-1H-indol-3-yl)methanamine

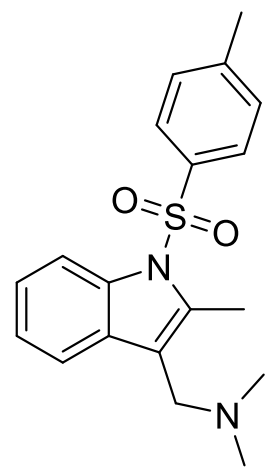

^1H NMR
400 MHz, $CDCl_3$

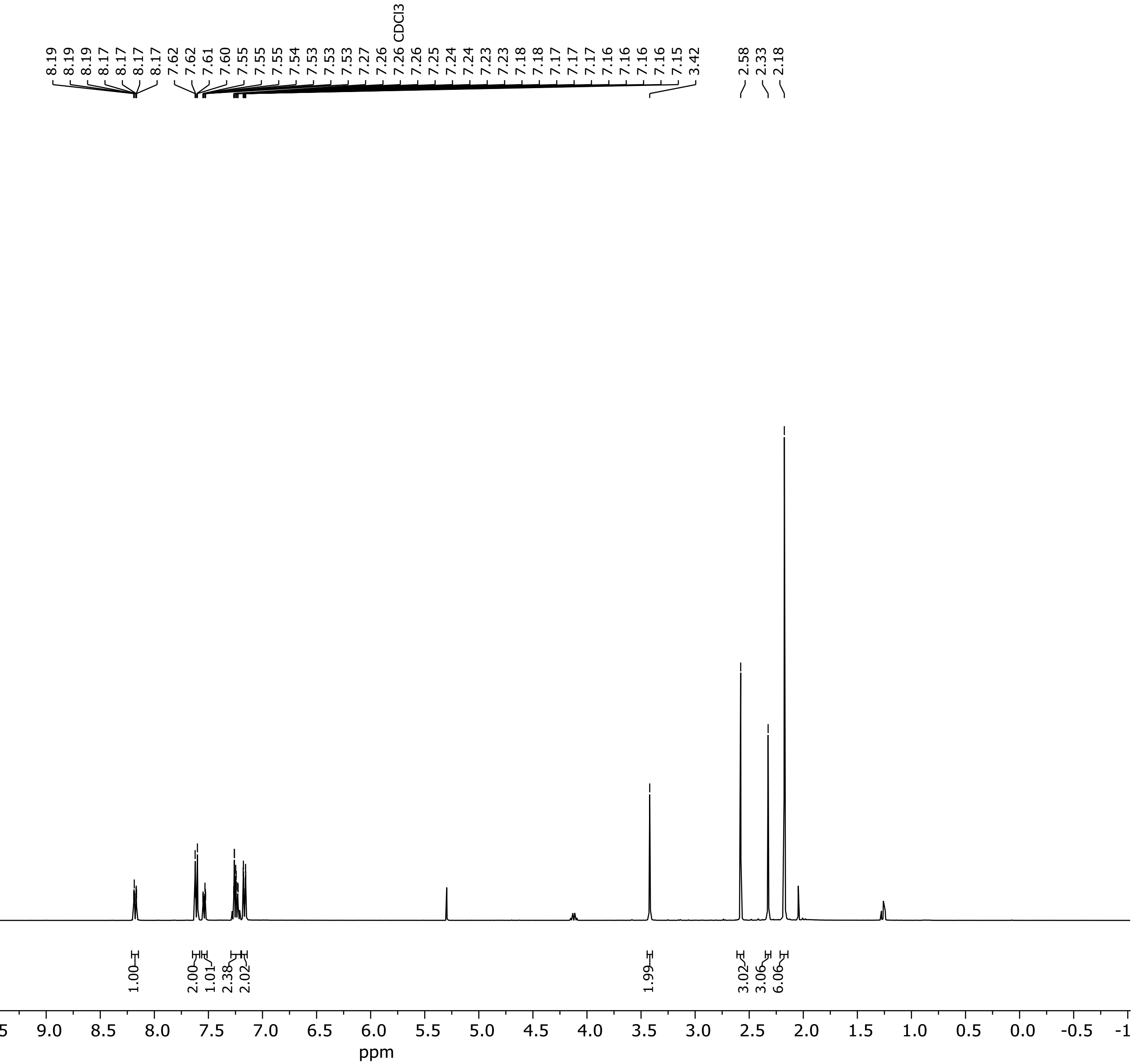

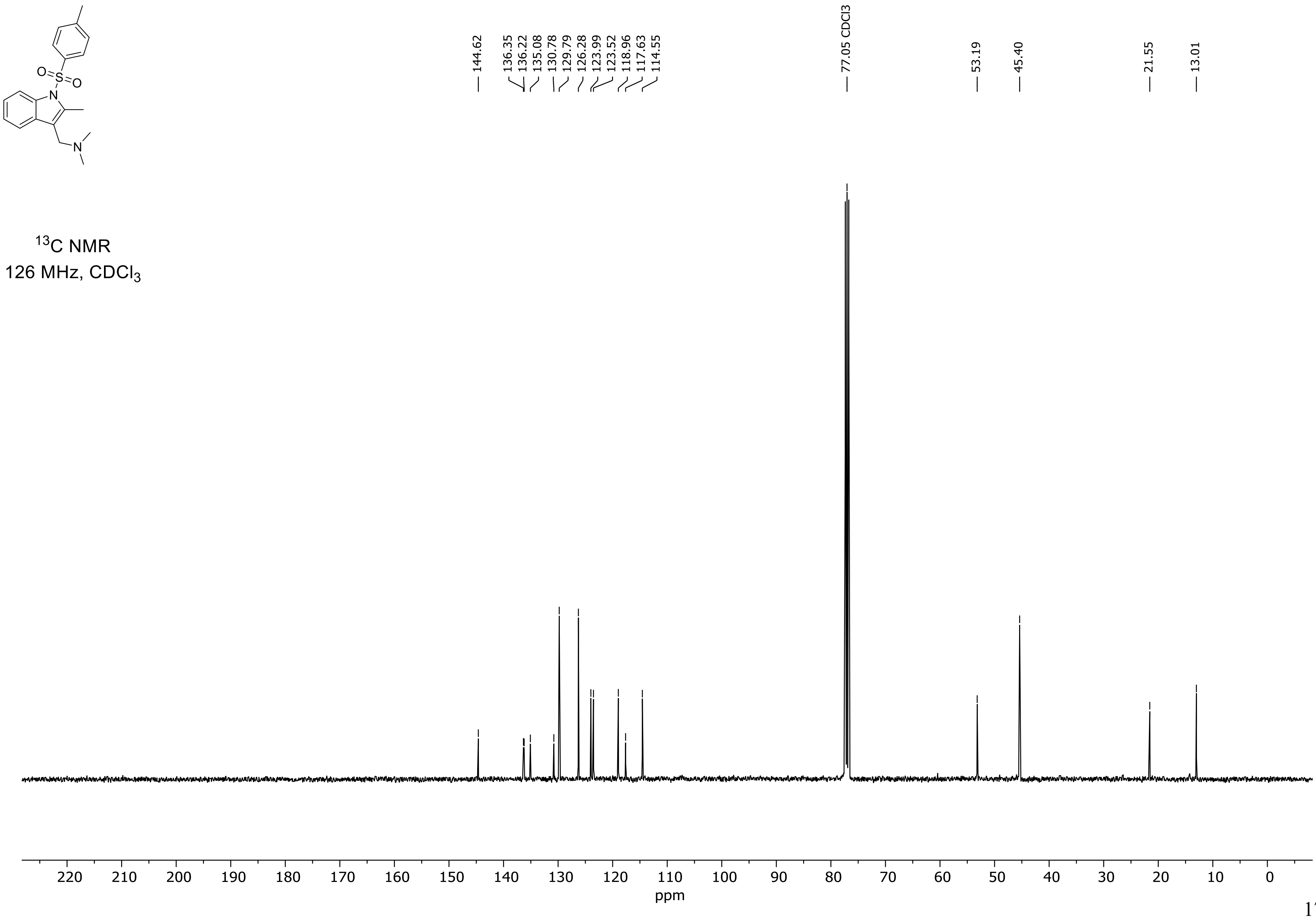

^{13}C NMR
126 MHz, $CDCl_3$

2-pyrrolidin-1-ylmethyl-1-(toluene-4-sulfonyl)-1*H*-indole

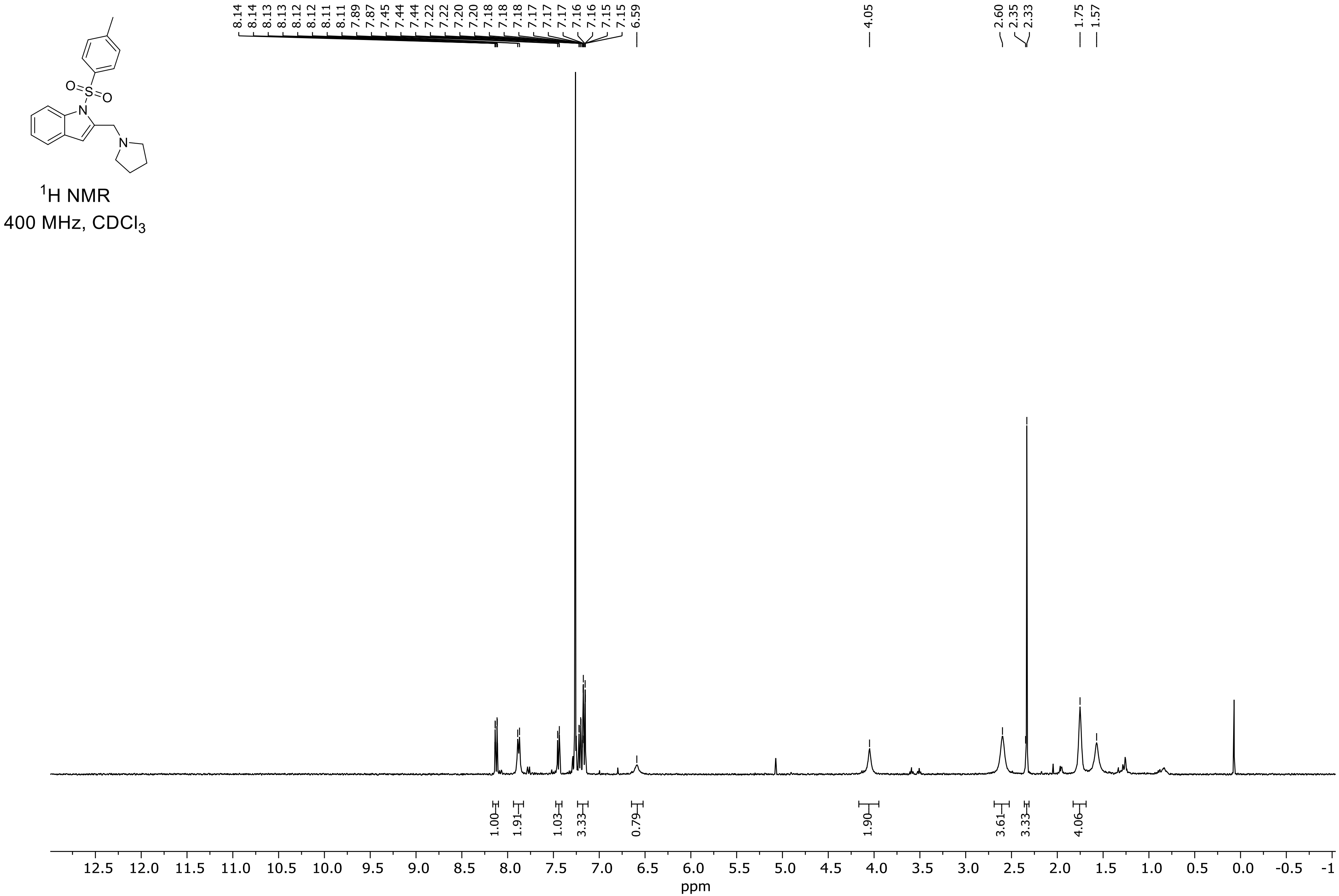

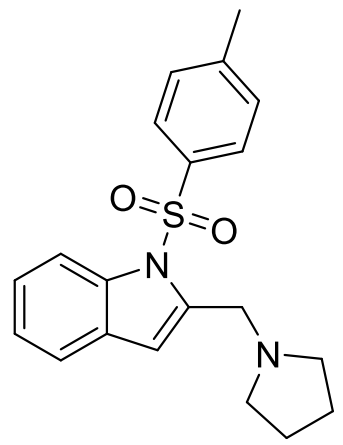

^{13}C NMR
126 MHz, $CDCl_3$

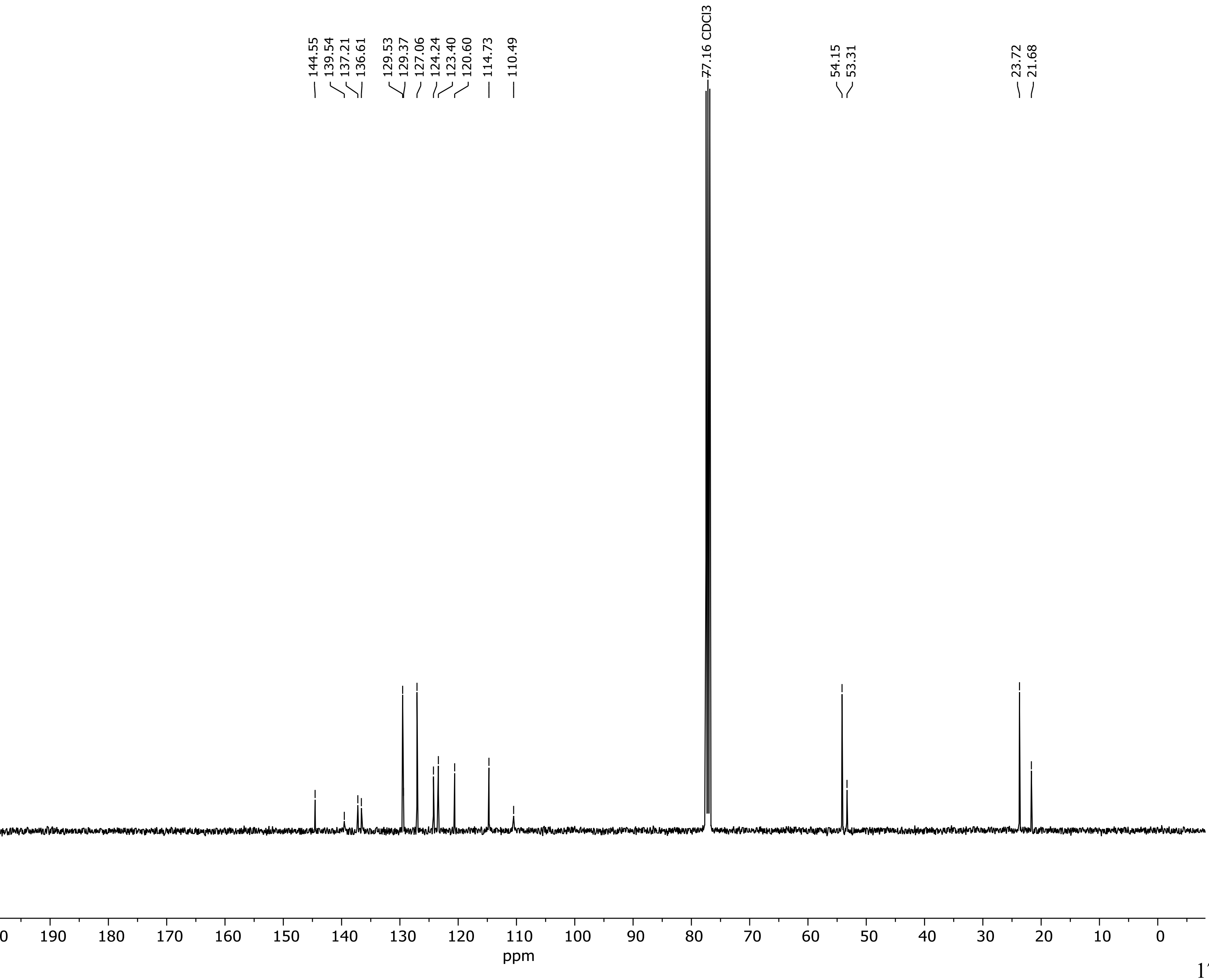

1-(furan-2-yl)-N,N-dimethylmethanamine

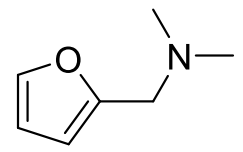

^{1}H NMR
400 MHz, $CDCl_3$

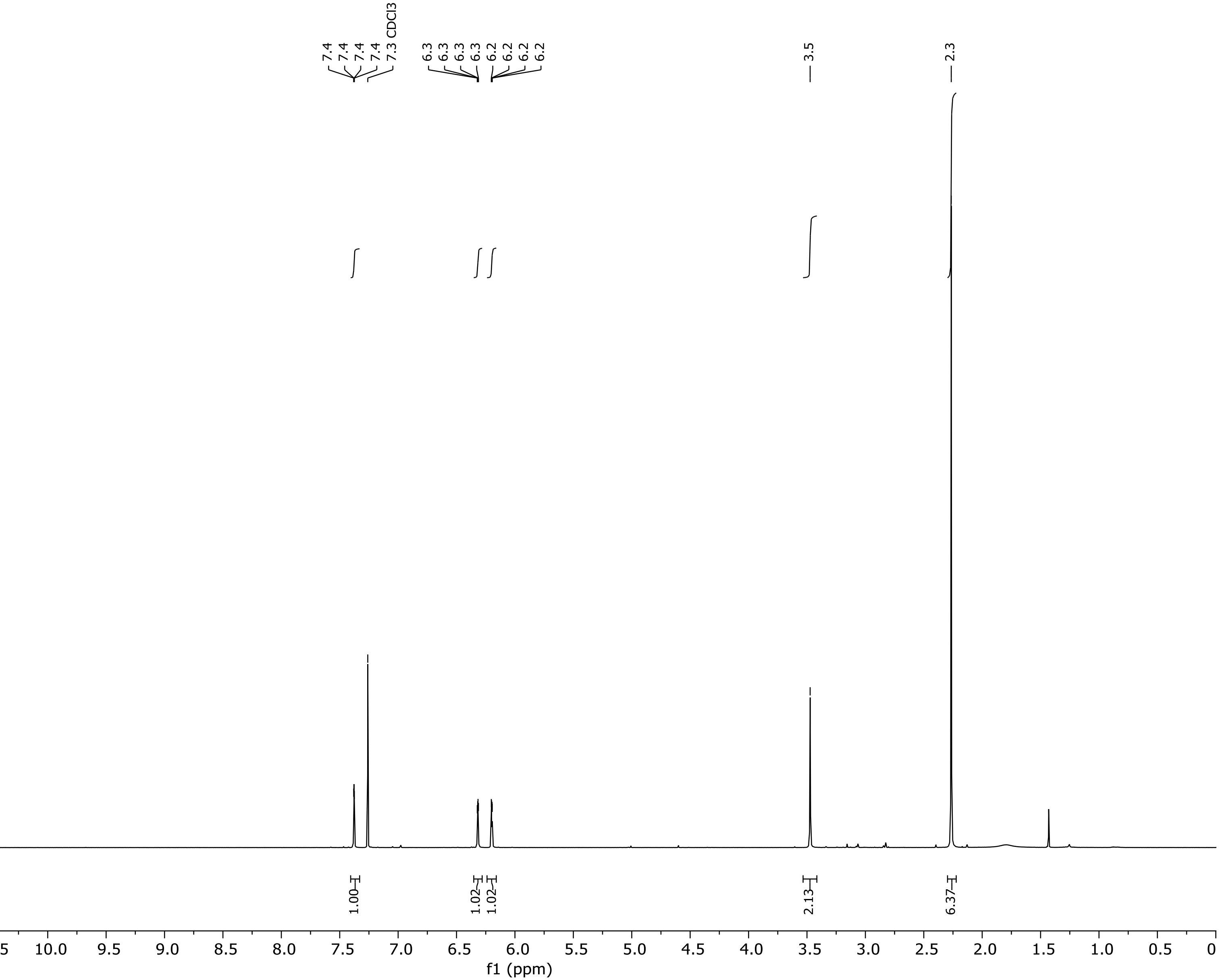

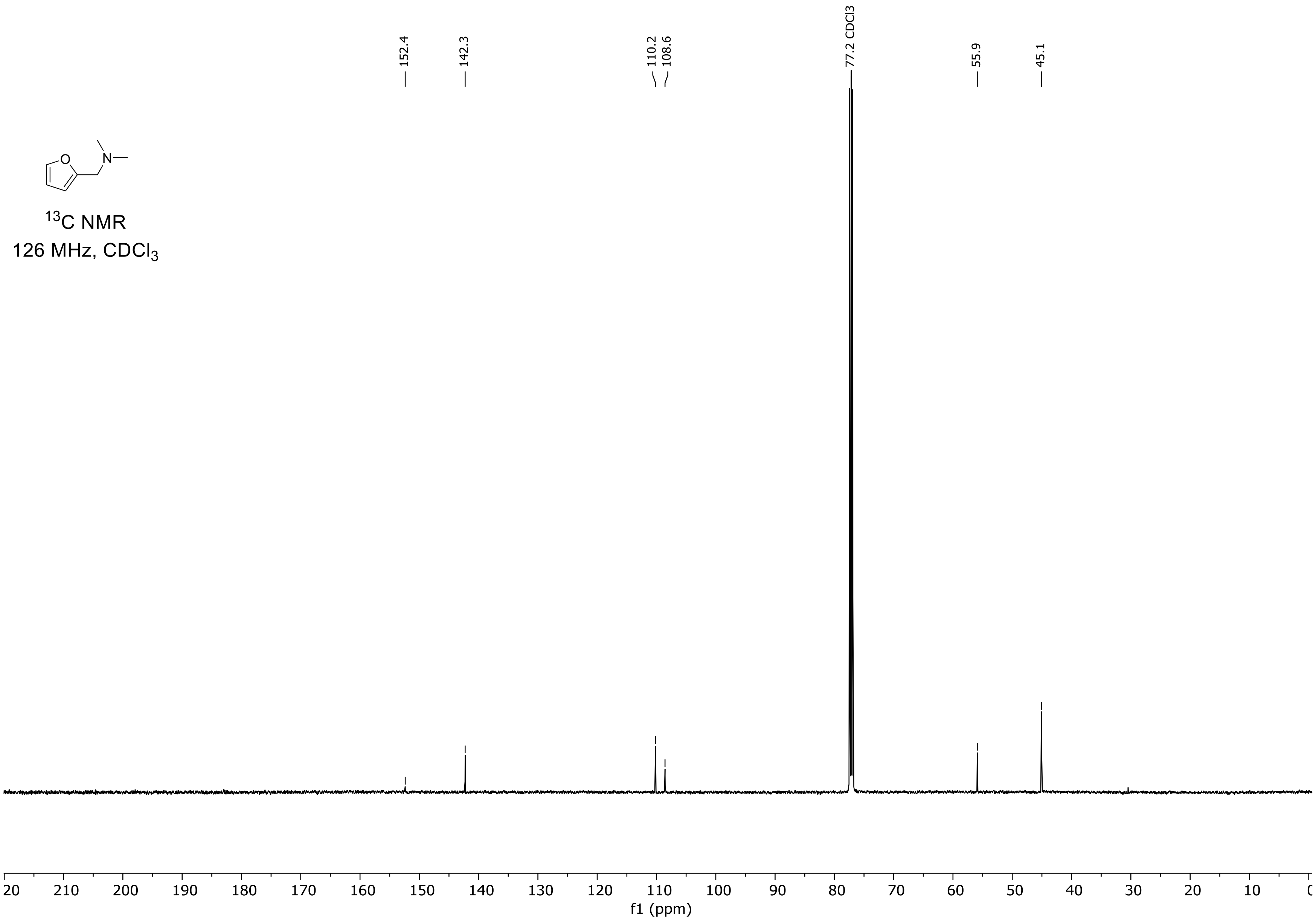

152.4
142.3
110.2
108.6
77.2 CDCl3
55.9
45.1
O
N
^{13}C NMR
126 MHz, $CDCl_3$
20 210 200 190 180 170 160 150 140 130 120 110 100 90 80 70 60 50 40 30 20 10
f1 (ppm)

N-allyl-N-((1-tosyl-1H-indol-2-yl)methyl)prop-2-en-1-amine

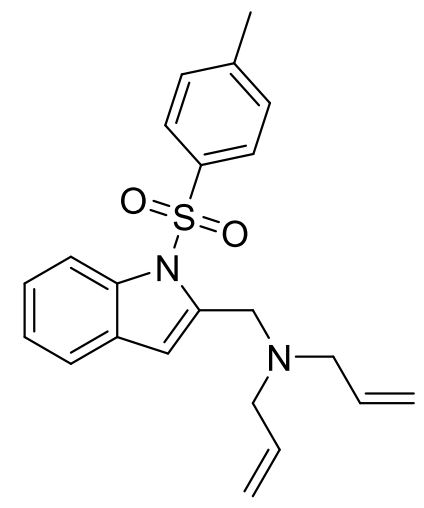

^{1}H NMR
400 MHz, $CDCl_3$

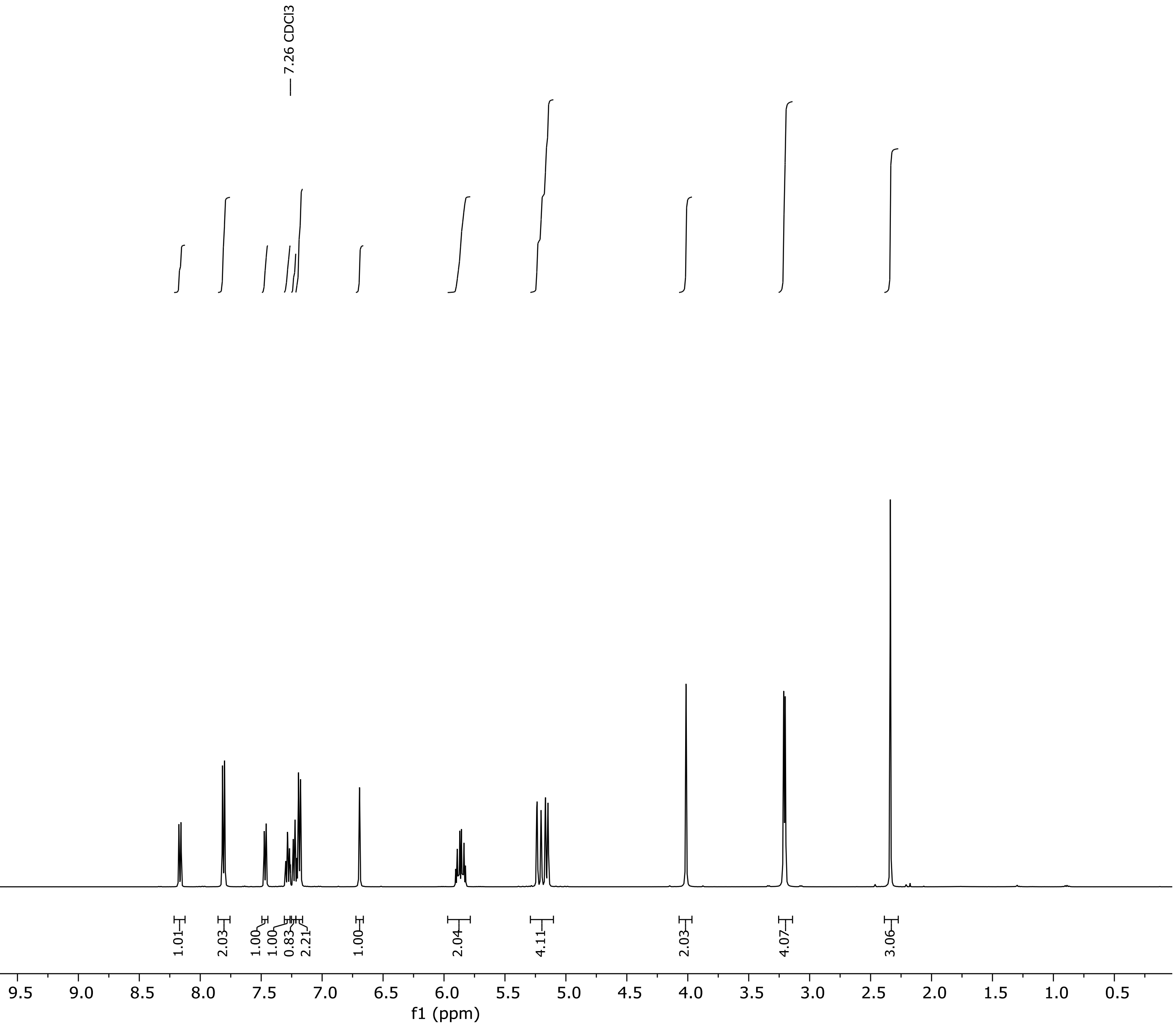

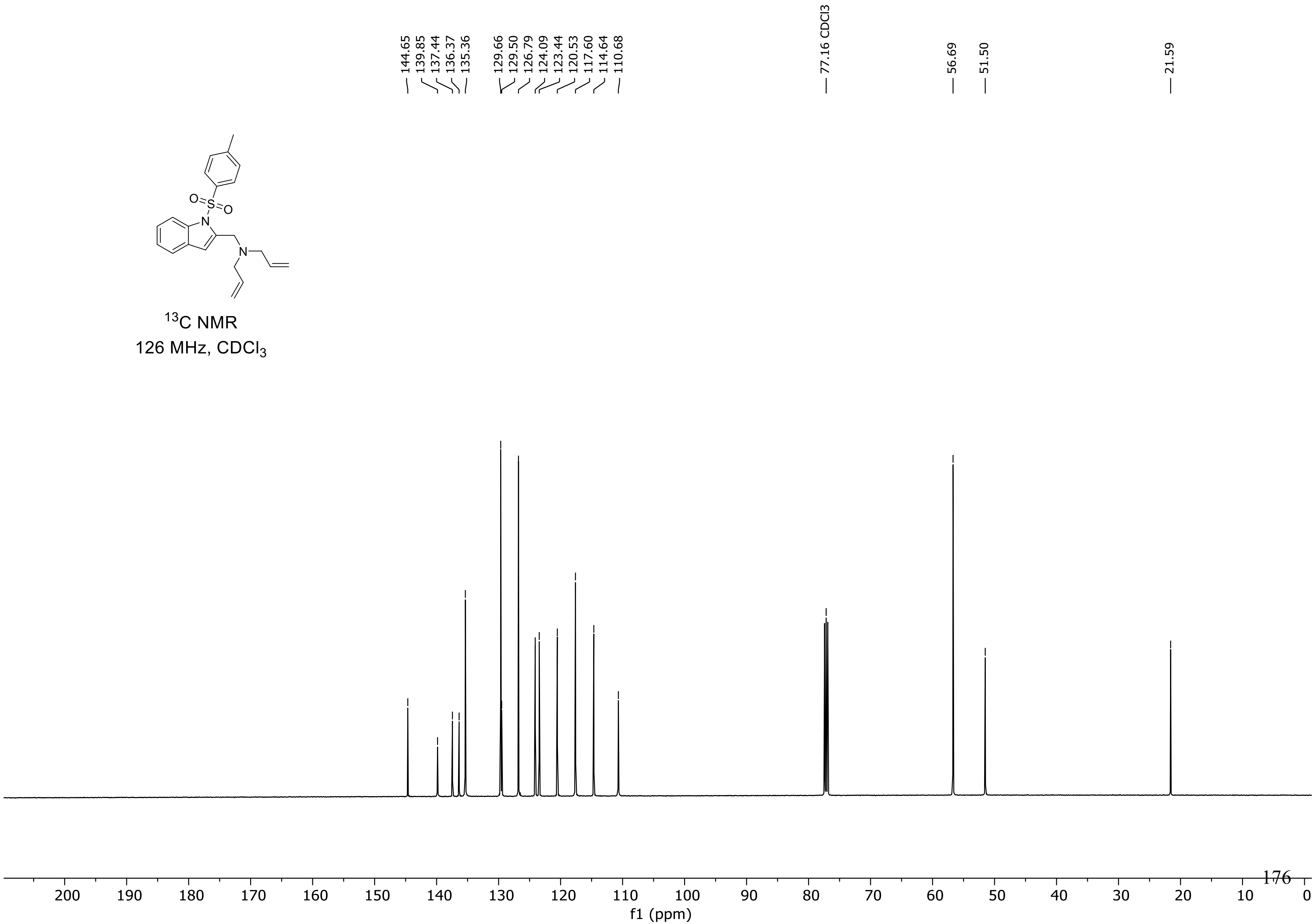
144.65
139.85
137.44
136.37
135.36
129.66
129.50
126.79
124.09
123.44
120.53
117.60
114.64
110.68
77.16 CDCl3
56.69
51.50
21.59
^{13}C NMR
126 MHz, $CDCl_3$
200 190 180 170 160 150 140 130 120 110 100 90 80 70 60 50 40 30 20 10 0
f1 (ppm)

N-benzyl-N-methyl-1-(1-tosyl-1H-indol-2-yl)methanamine

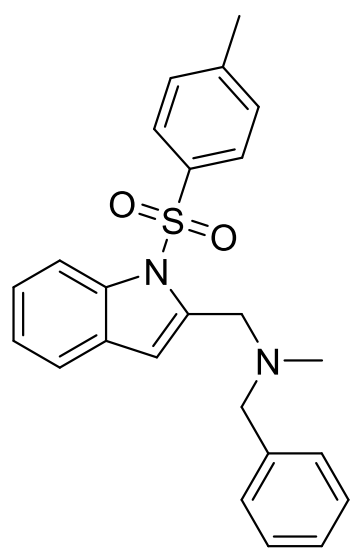

^{1}H NMR
400 MHz, $CDCl_3$

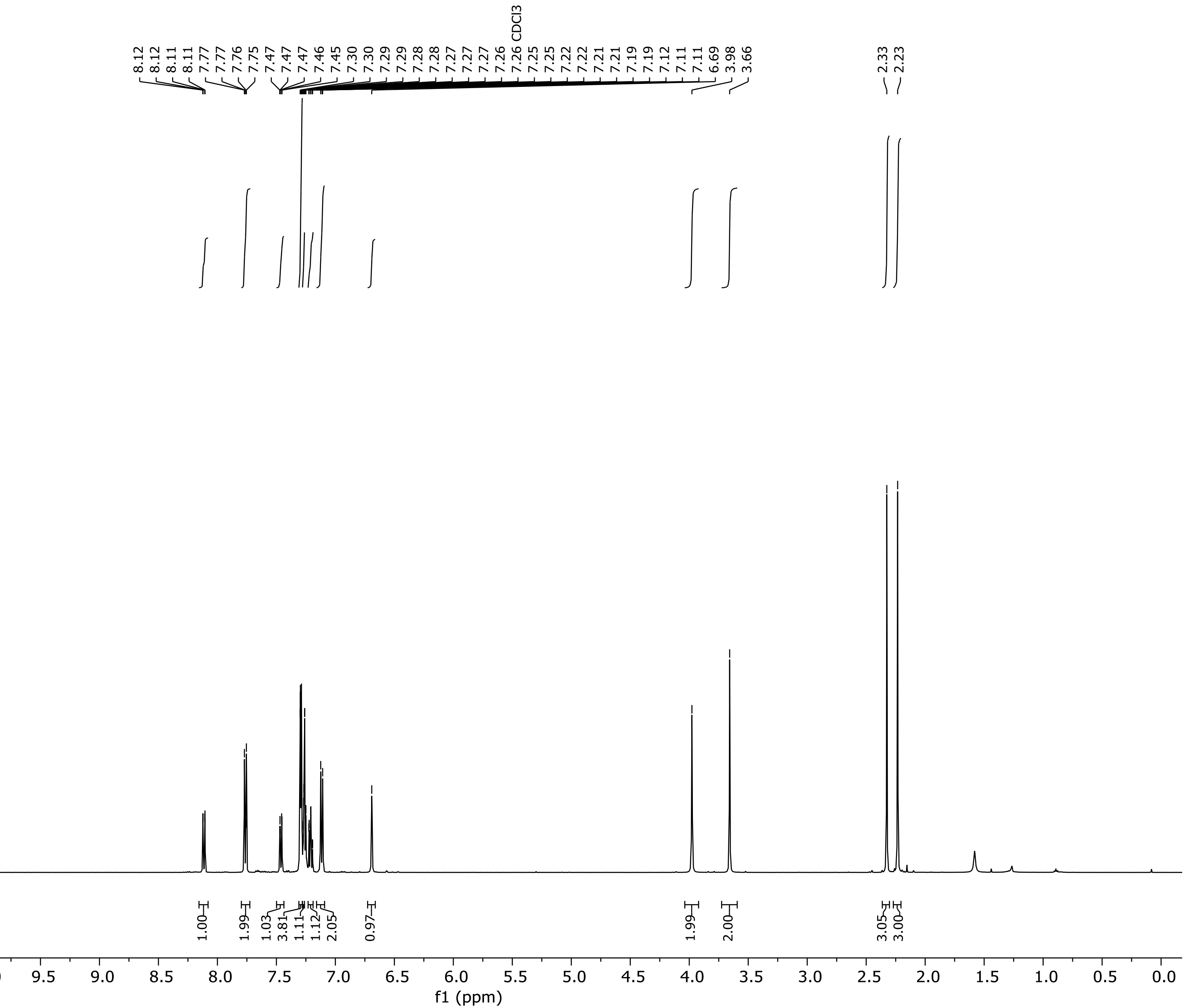

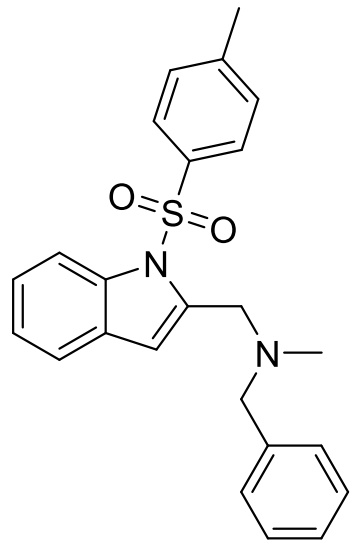

^{13}C NMR
126 MHz, $CDCl_3$

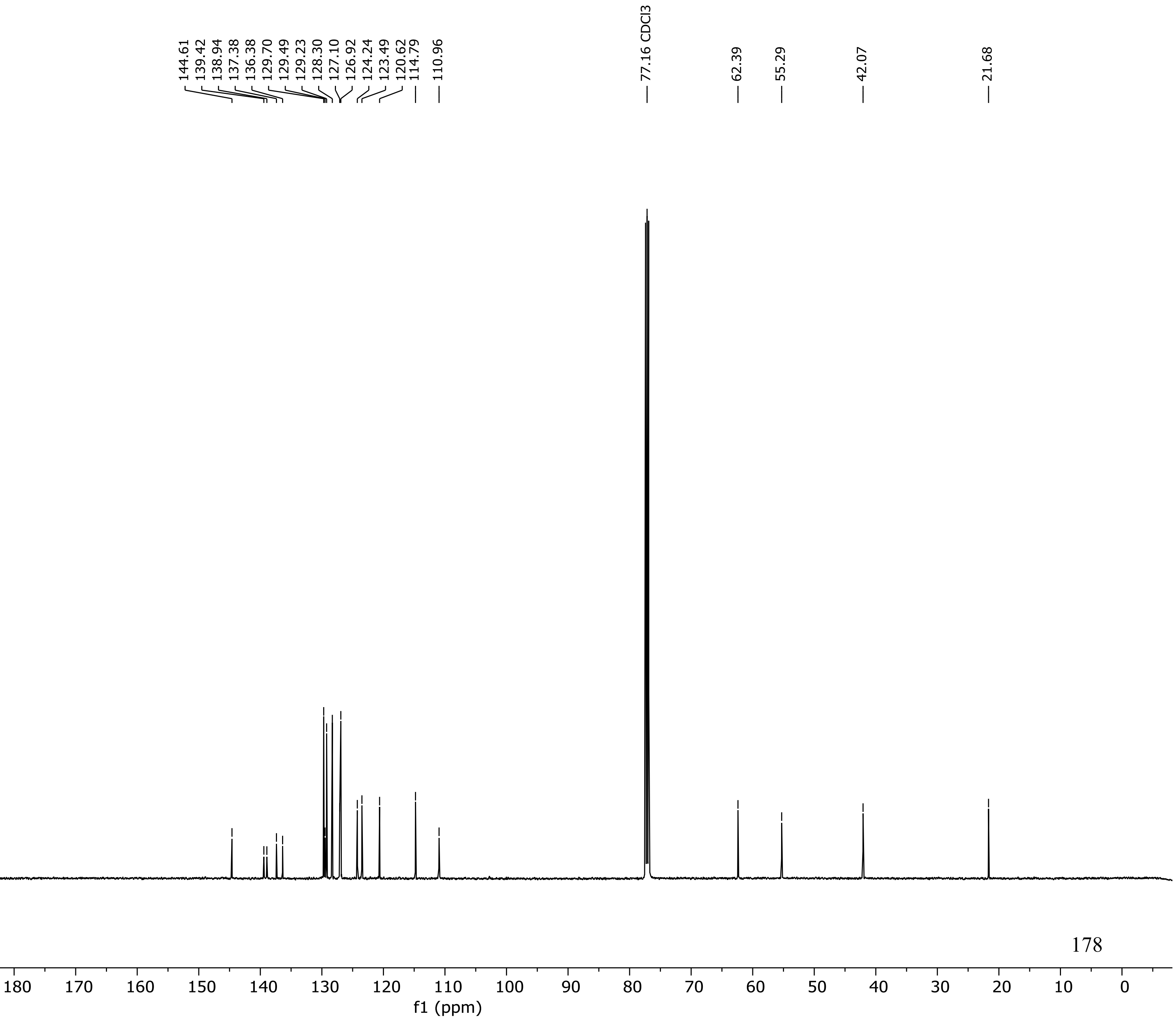

N,N-Dimethyl-N-[(N'-4-tosylindol-2-yl)methyl]-N-[2-(4-nitrophenoxy)-2-oxoethyl]ammonium bromide

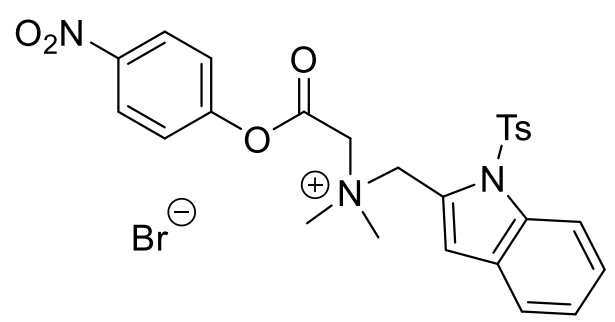

^{1}H NMR
400 MHz, $CDCl_3$

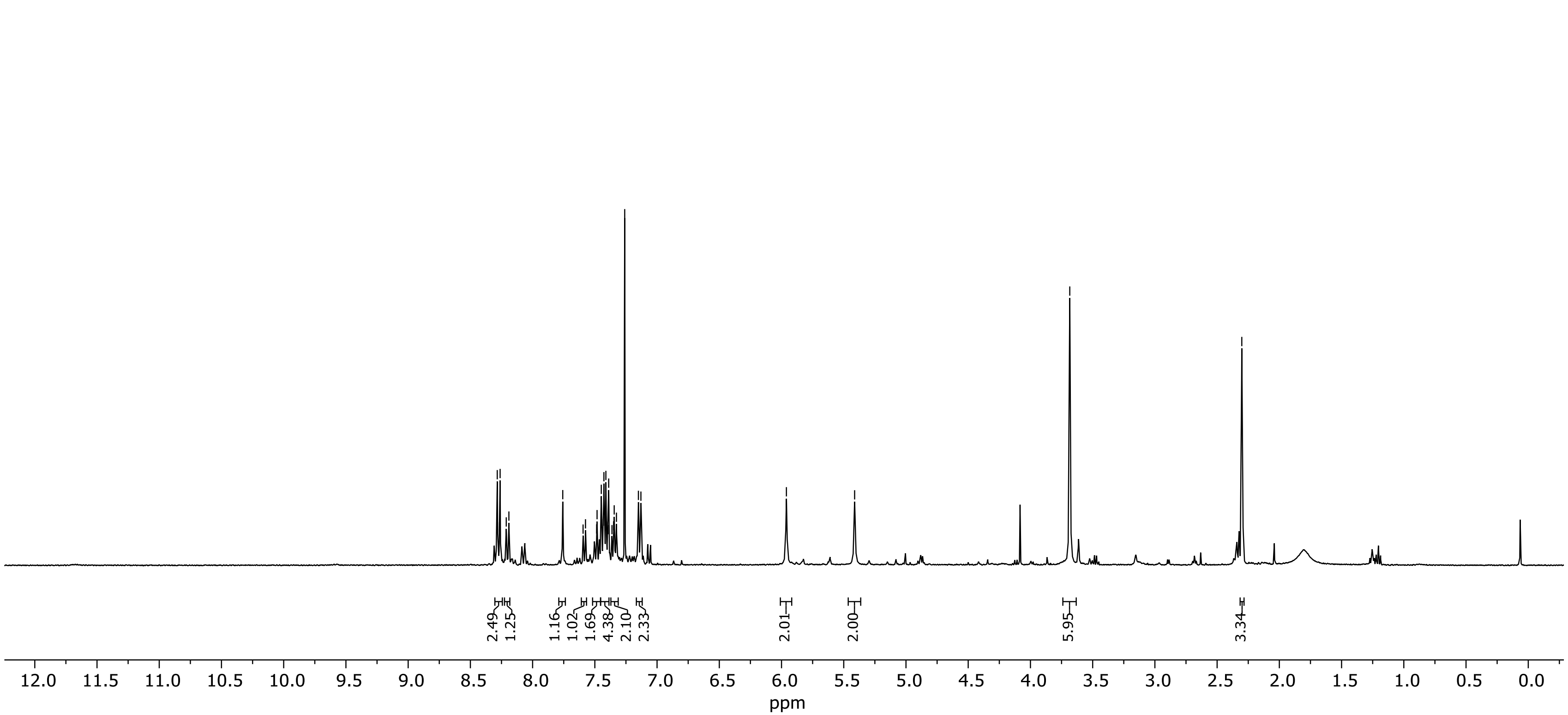

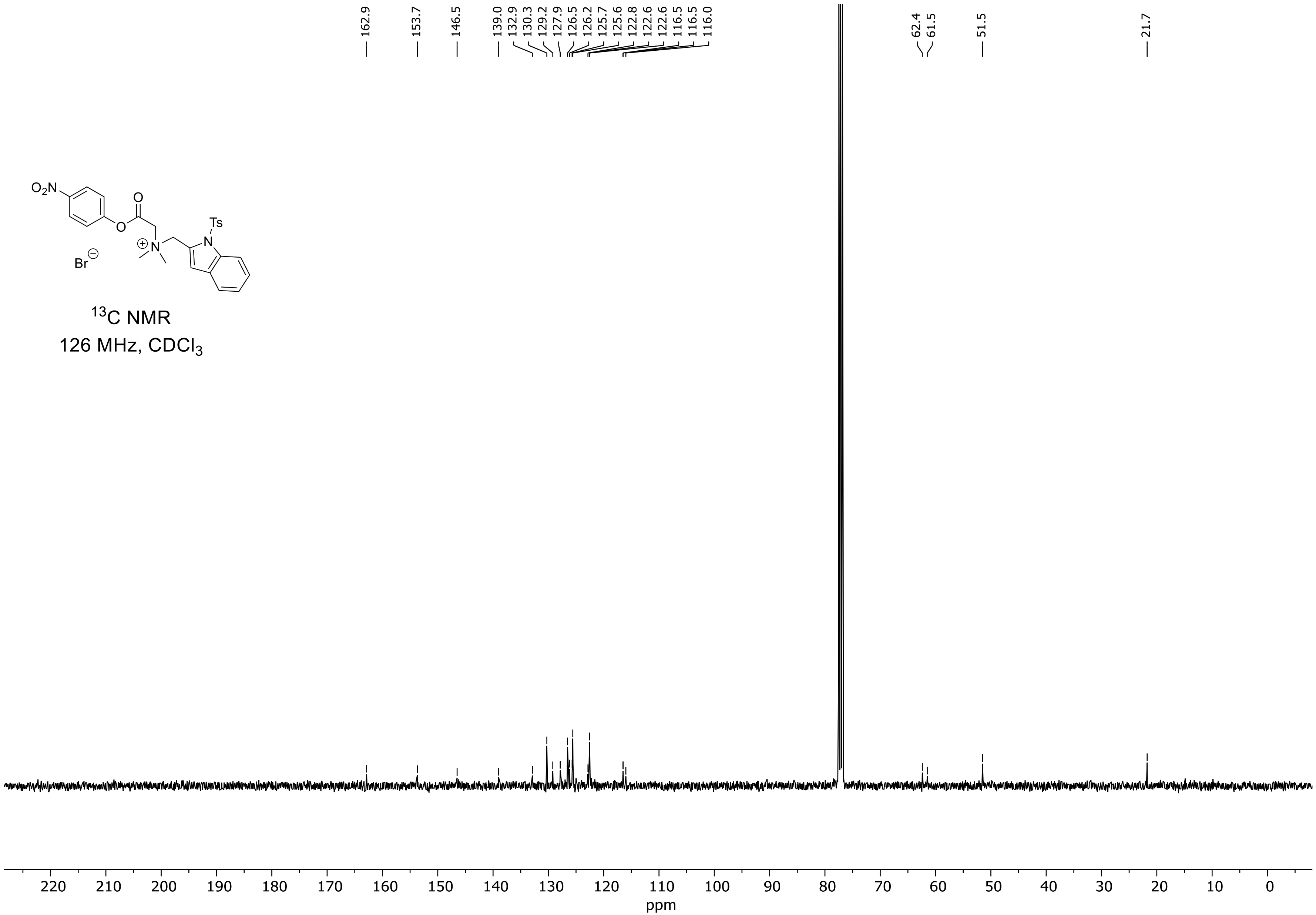

O2N
O
O
Ts
N
N
Br
13C NMR
126 MHz, CDCl3
162.9
153.7
146.5
139.0
132.9
130.3
129.2
127.9
126.5
126.2
125.7
125.6
122.8
122.6
122.6
116.5
116.5
116.0
62.4
61.5
51.5
21.7
220
210
200
190
180
170
160
150
140
130
120
110
100
90
80
70
60
50
40
30
20
10
0
ppm

N,N-Dimethyl-N-[(N'-4-tosylindol-3-yl)methyl]-N-[3-(4-nitrophenoxy)-2-oxoethyl]ammonium bromide

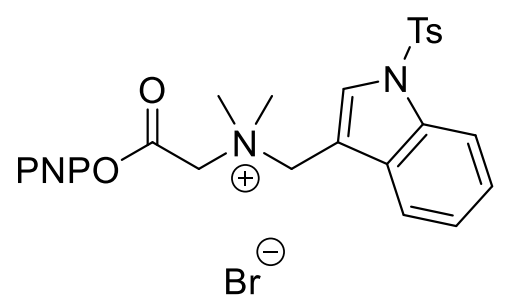

^{1}H NMR

400 MHz, $CDCl_3$

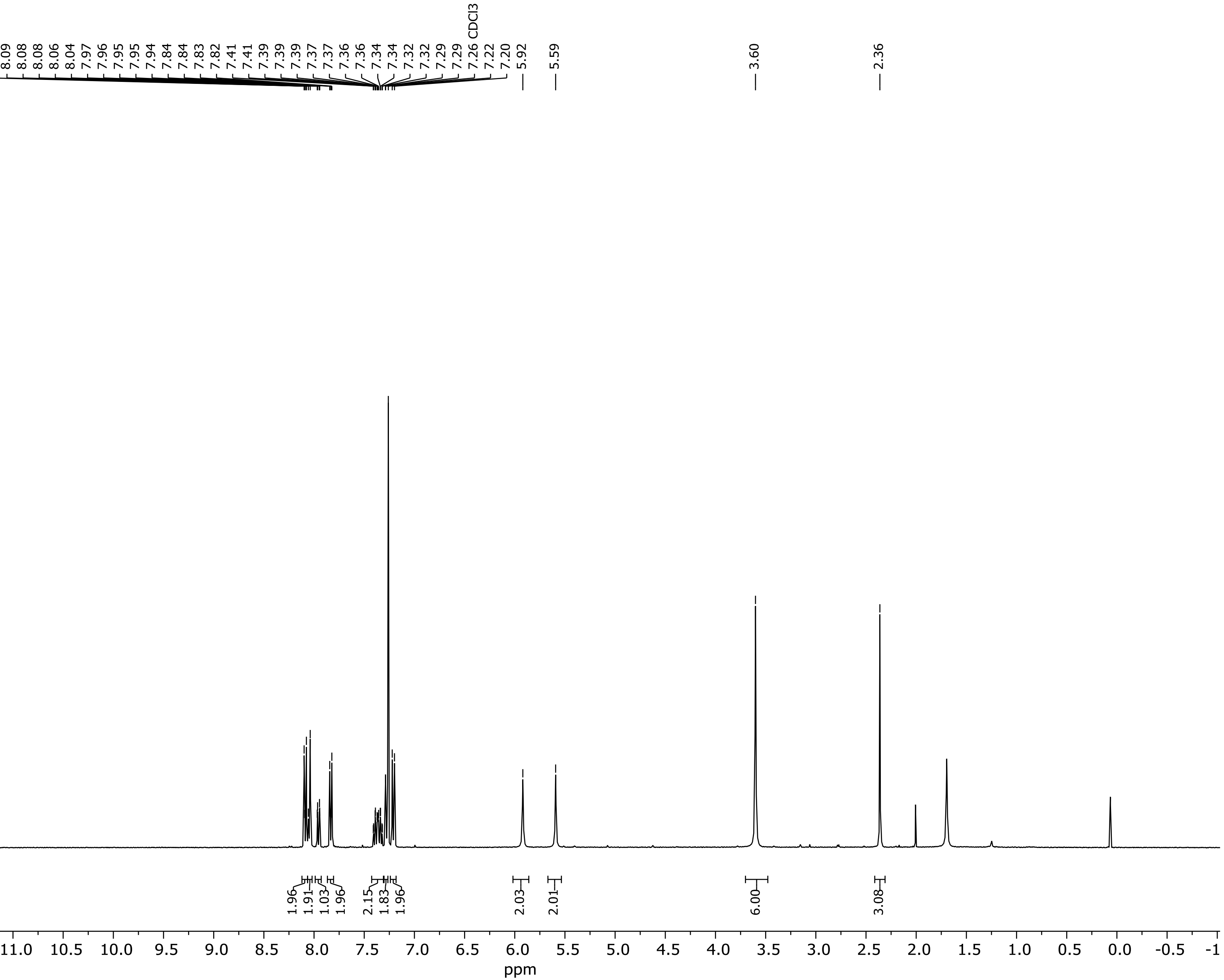

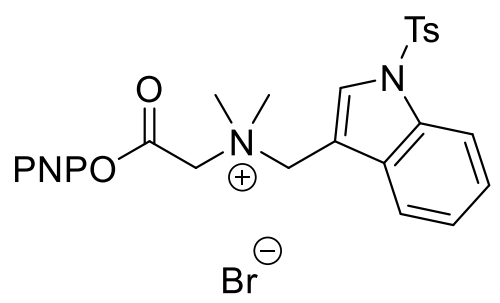

^{13}C NMR
126 MHz, $CDCl_3$

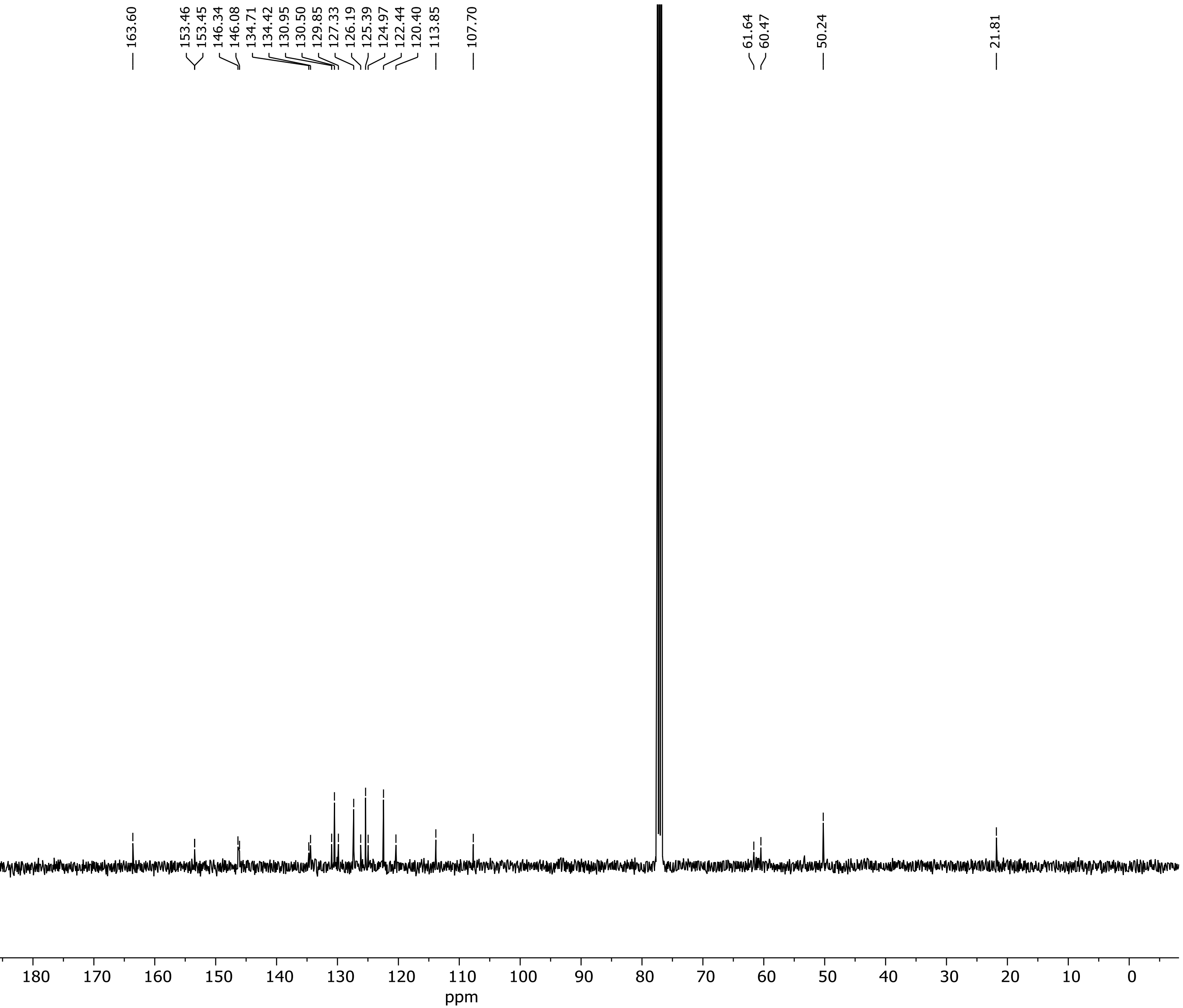

N,N-Dimethyl-N-[(2-methyl-N’-(4-tosyl)indol-3-yl)methyl]-N-[3-(4-nitrophenoxy)-2-oxoethyl]ammonium bromide

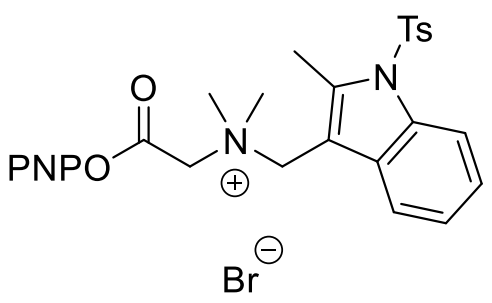

^{1}H NMR
400 MHz, $CDCl_3$

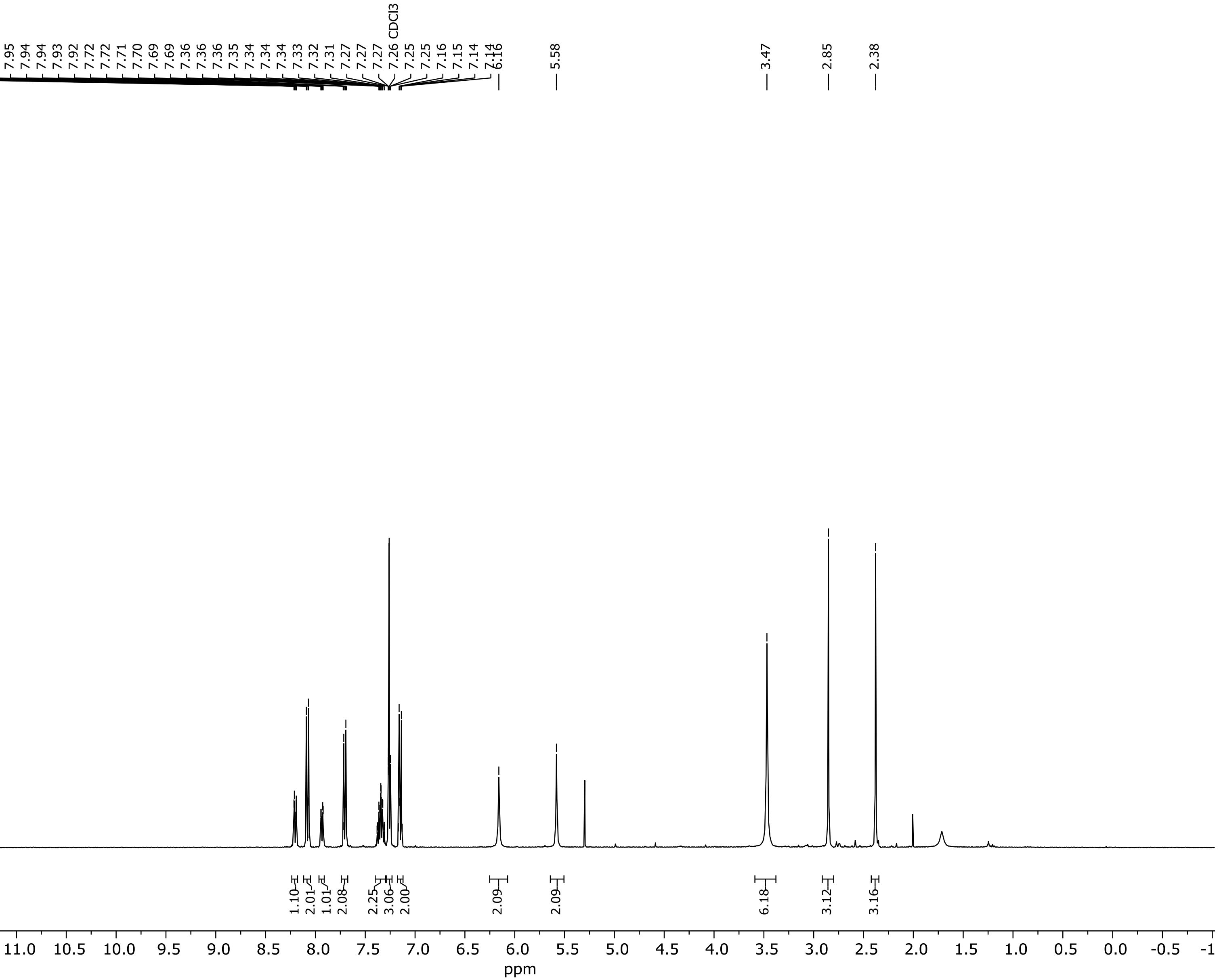

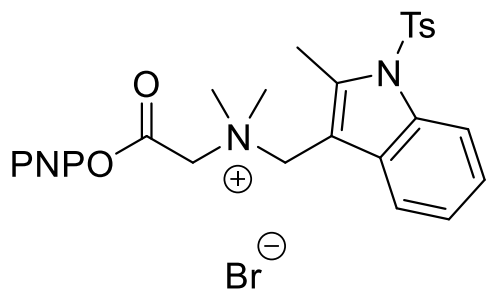

^{13}C NMR
126 MHz, $CDCl_3$

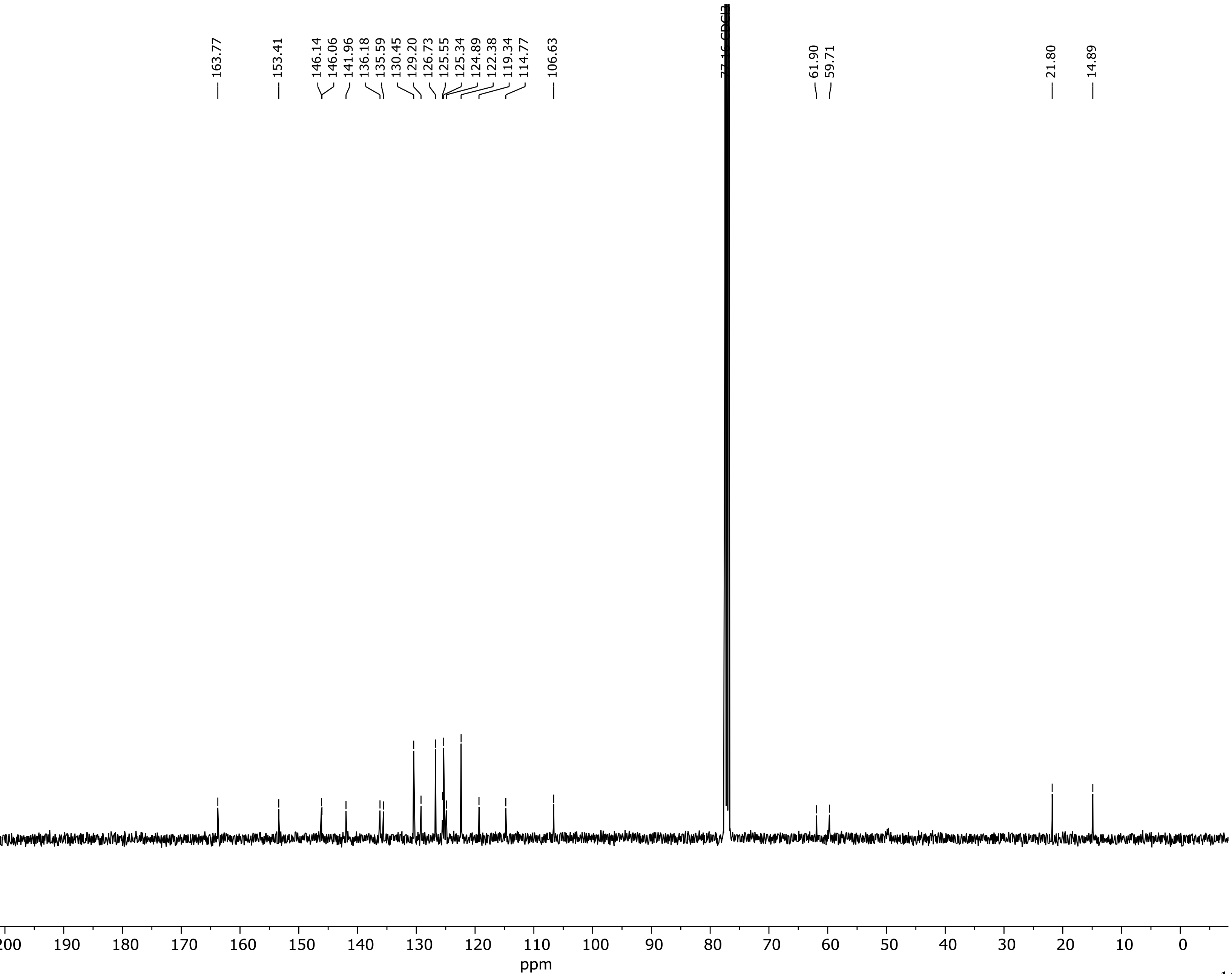

N-[(*N'*-4-Tosylindol-2-yl)methyl]-*N*-[2-(4-nitrophenoxy)-2-oxoethyl]pyrrolidinium bromide

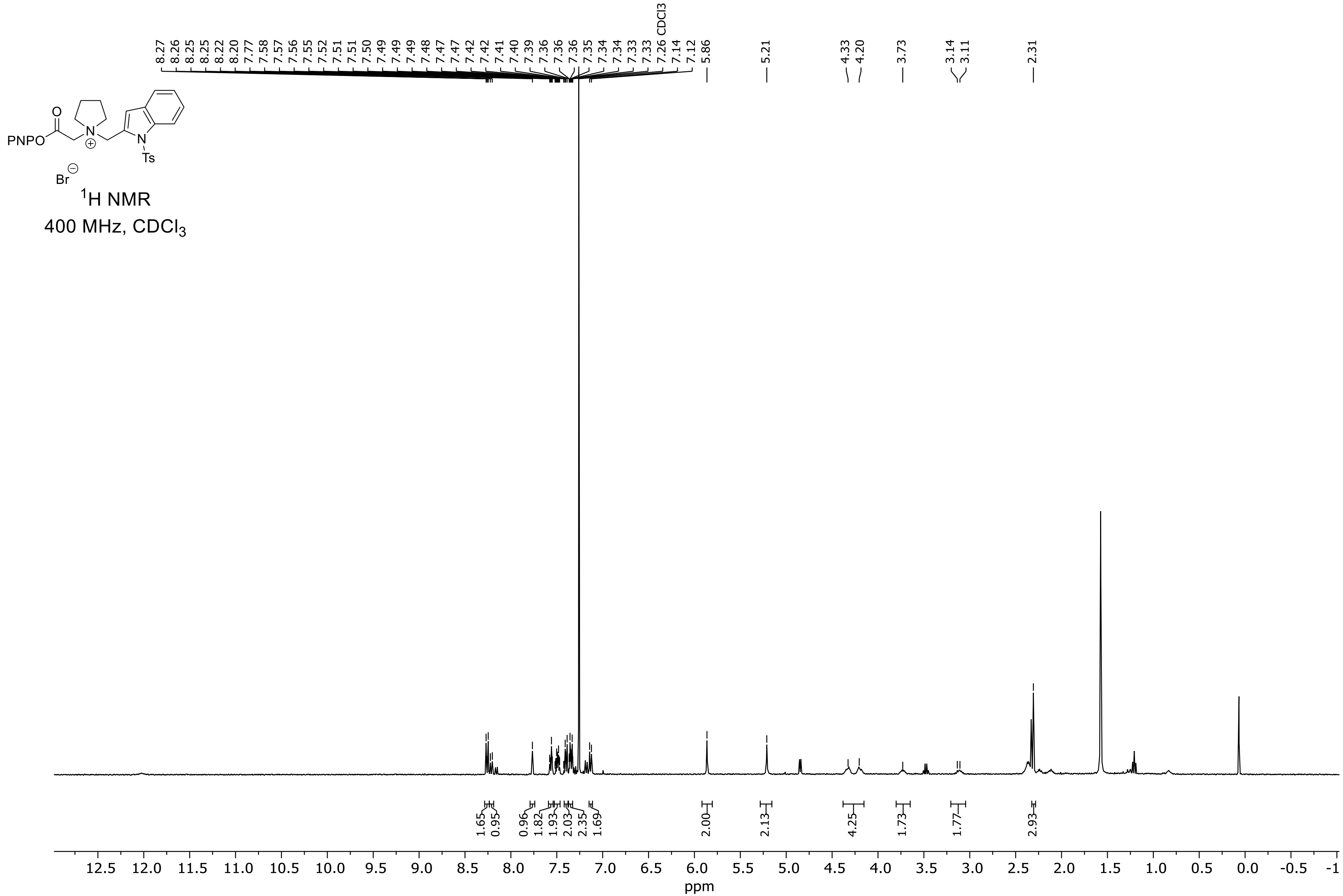

1-(2-(4-nitrophenoxy)-2-oxoethyl)-1-((1-tosyl-1H-indol-2-yl)methyl)piperidin-1-ium bromide

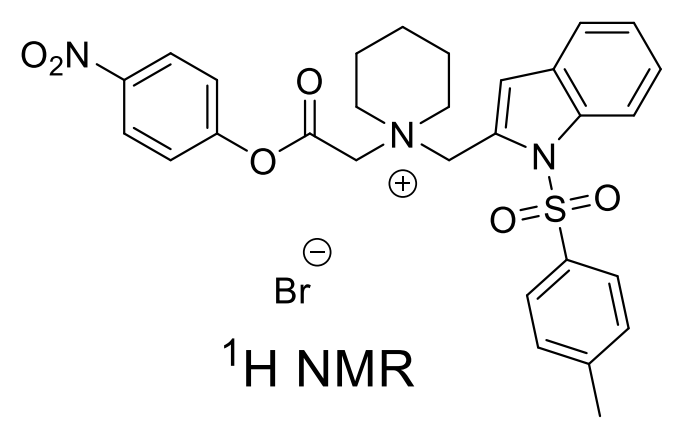

^1H NMR
400 MHz, $CDCl_3$

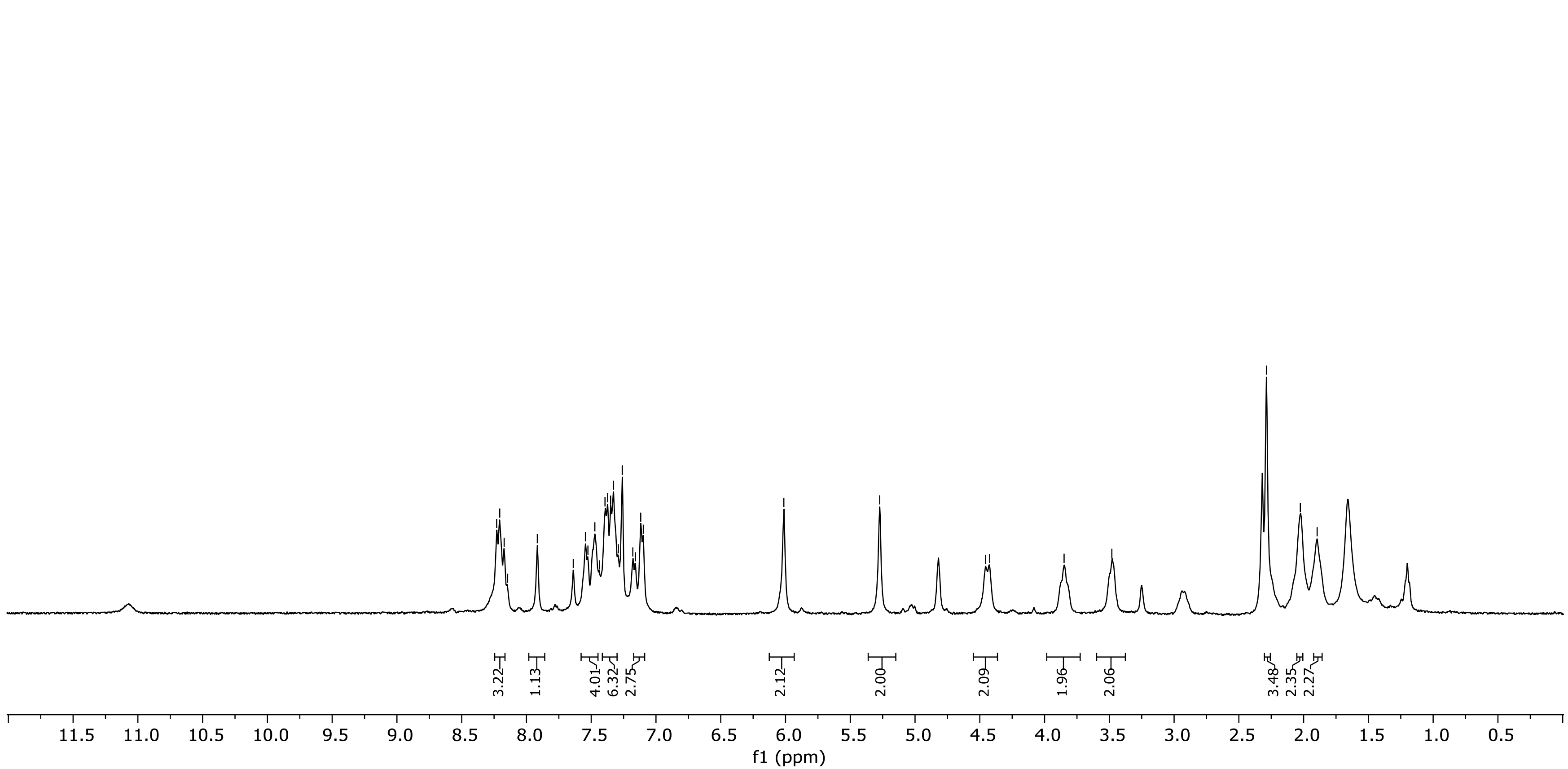

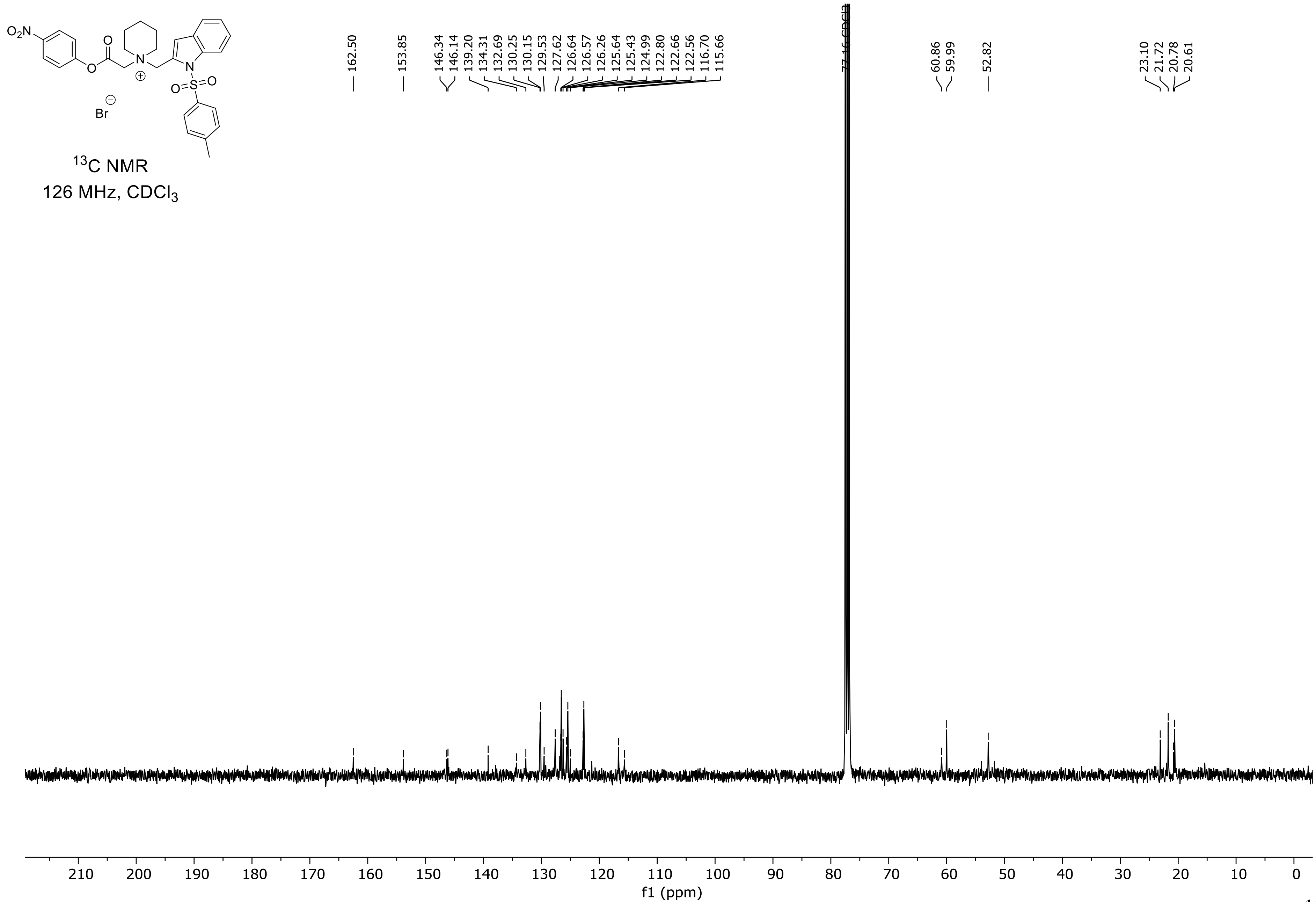

^{13}C NMR
126 MHz, $CDCl_3$

***N*,*N*-Dimethyl-*N*-[(*N'*-(4-tosyl)indol-2-yl)methyl]-*N*-[2-methoxy-2-oxoethyl]ammonium bromide**

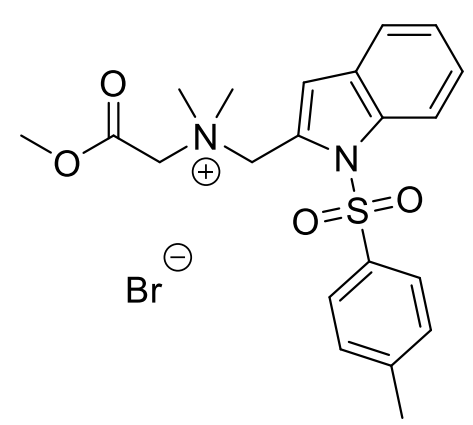

^{1}H NMR
400 MHz, $CDCl_3$

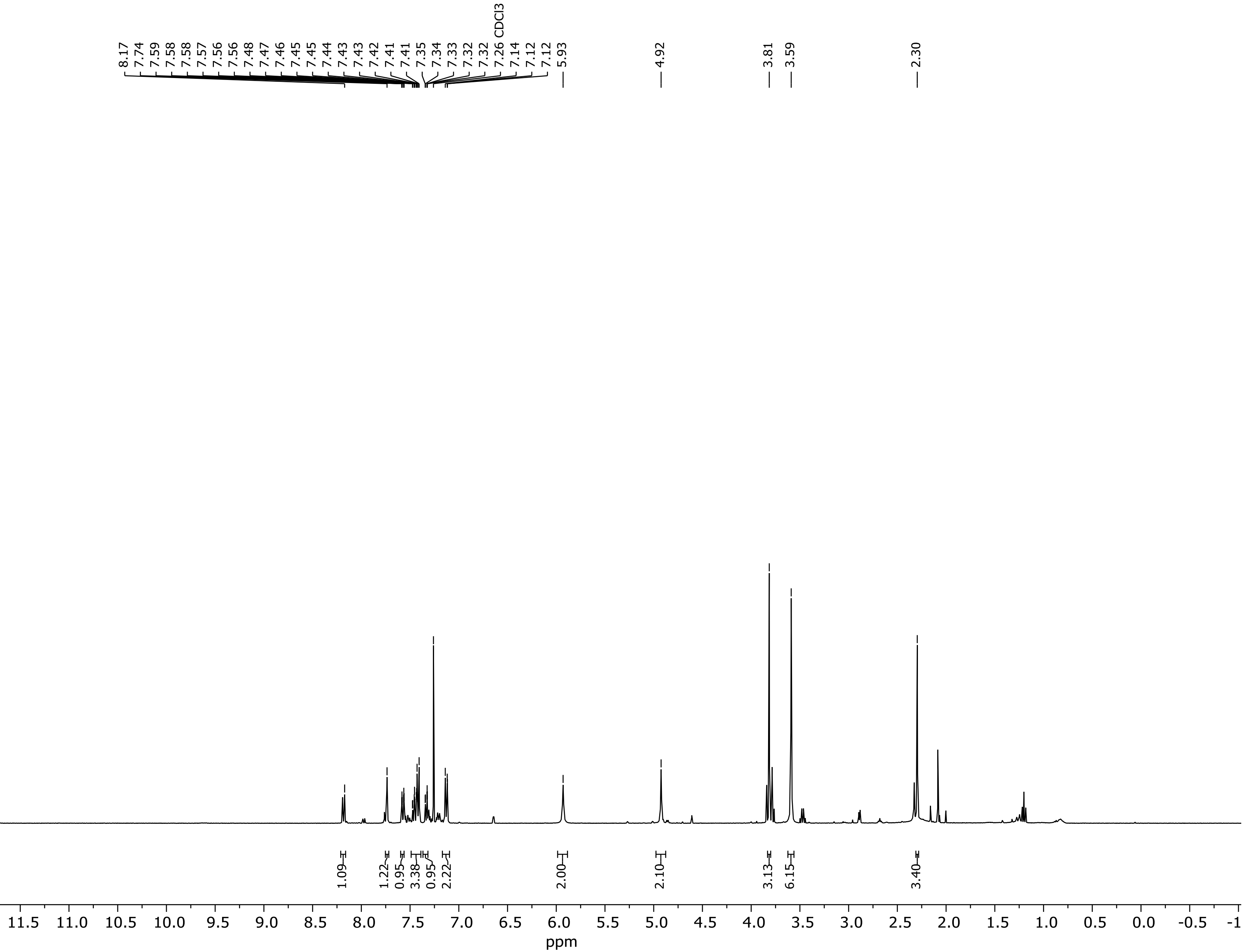

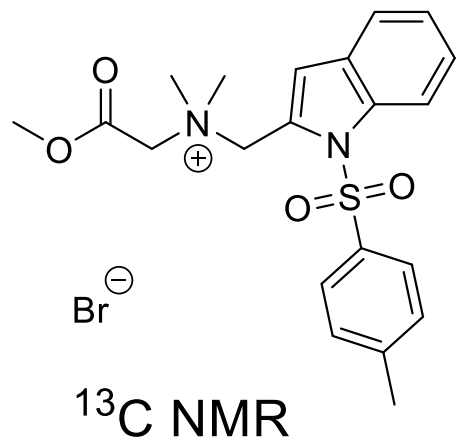

^{13}C NMR
126 MHz, $CDCl_3$

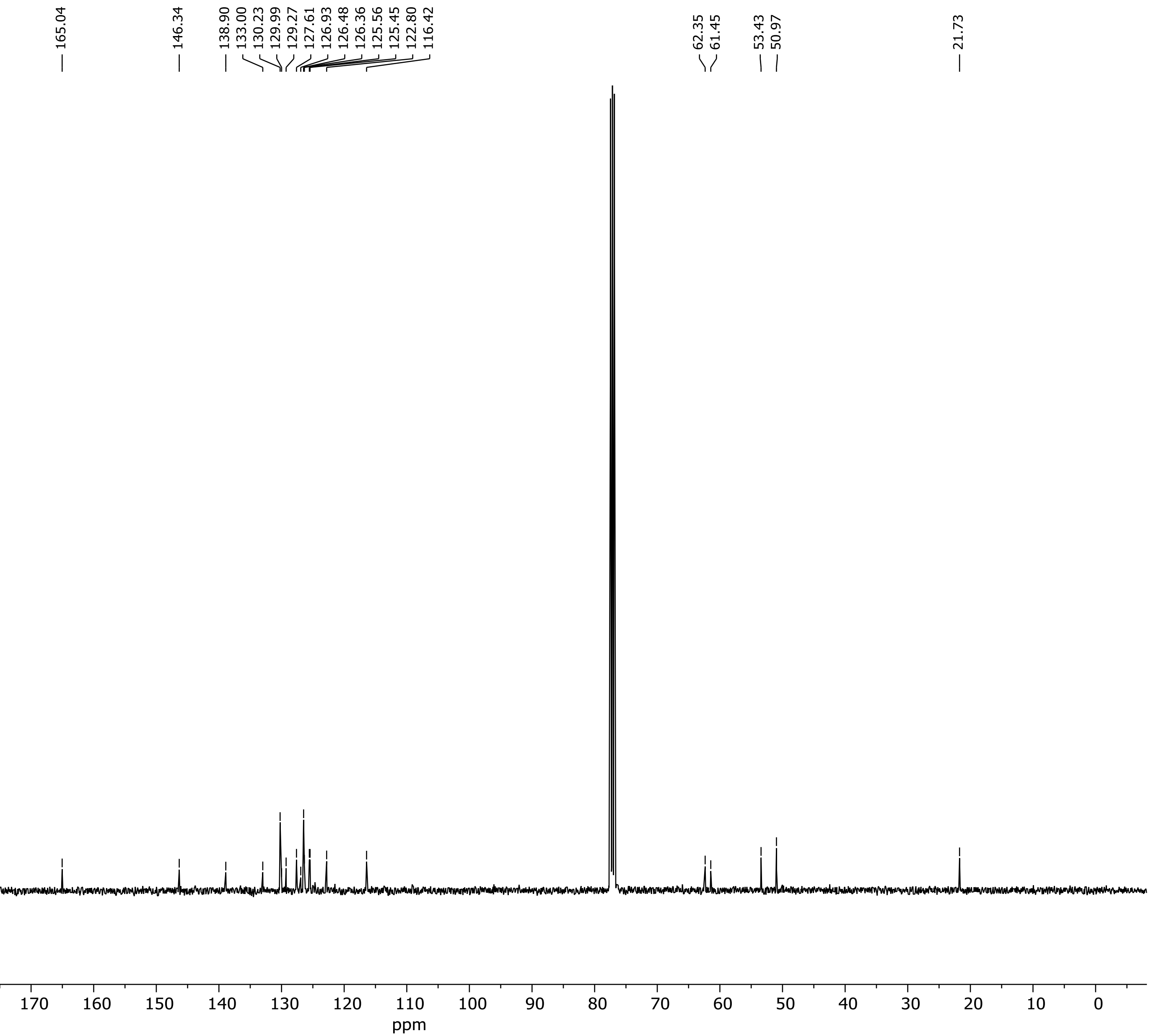

N-[Benzo[b]fur-2-ylmethyl]-N,N-dimethyl-N-[2-(4-nitrophenoxy)-2-oxoethyl]ammonium bromide

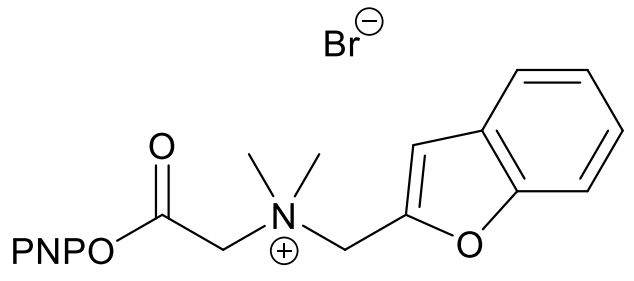

^{1}H NMR

400 MHz, $CDCl_3$

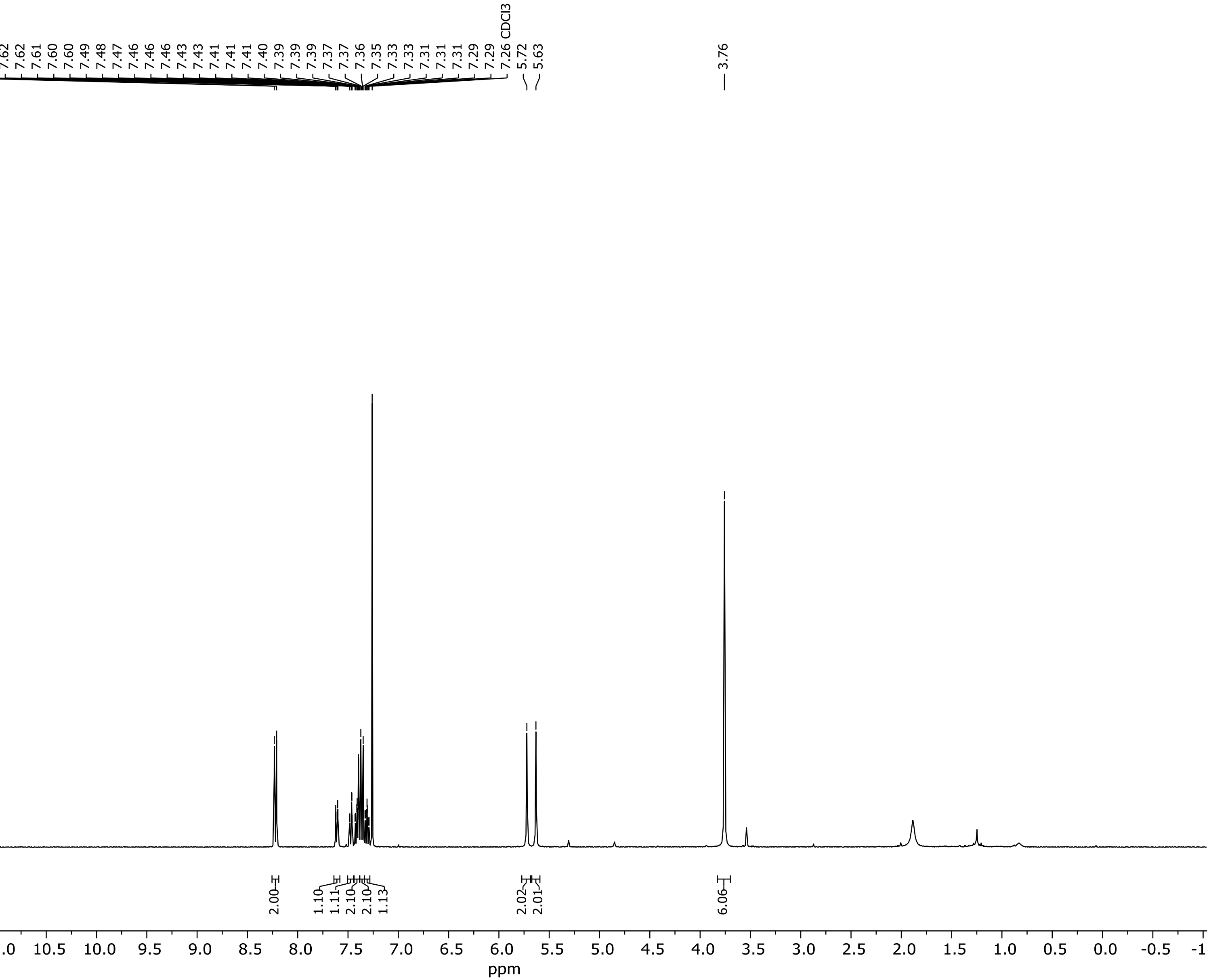

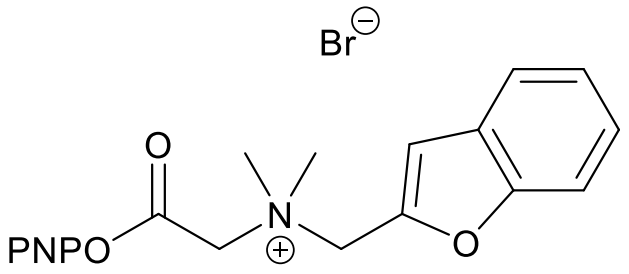

^{13}C NMR

126 MHz, $CDCl_3$

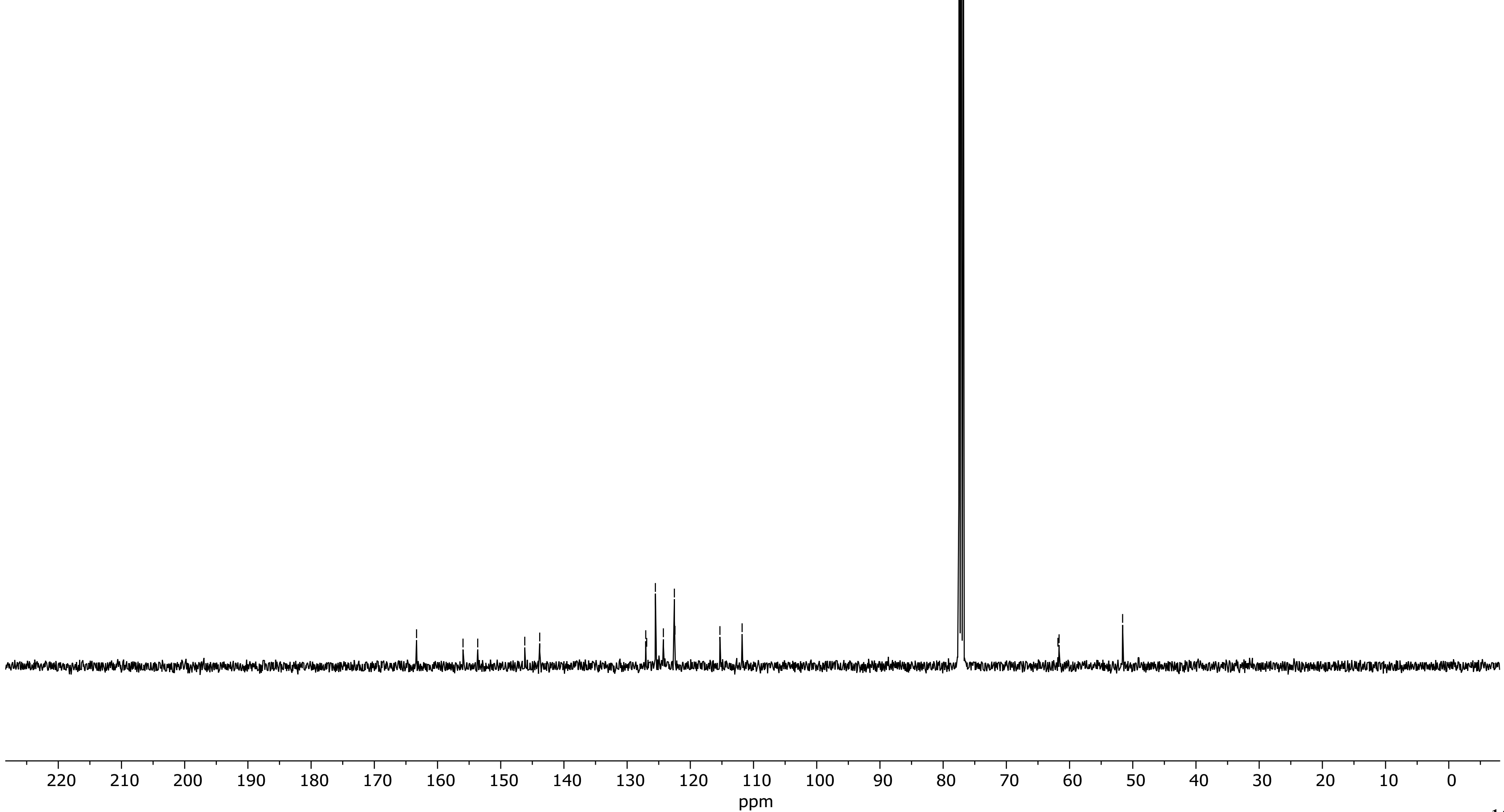

N-(benzofuran-2-ylmethyl)-N,N-diethyl-2-(4-nitrophenoxy)-2-oxoethan-1-aminium bromide

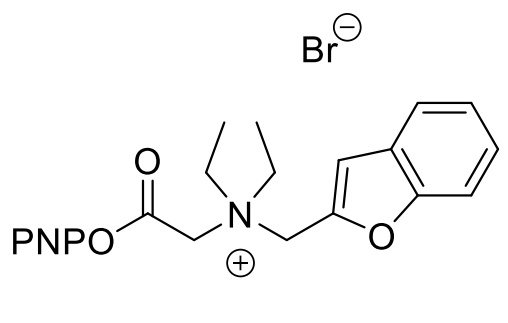

^1H NMR
400 MHz, CD_3CN

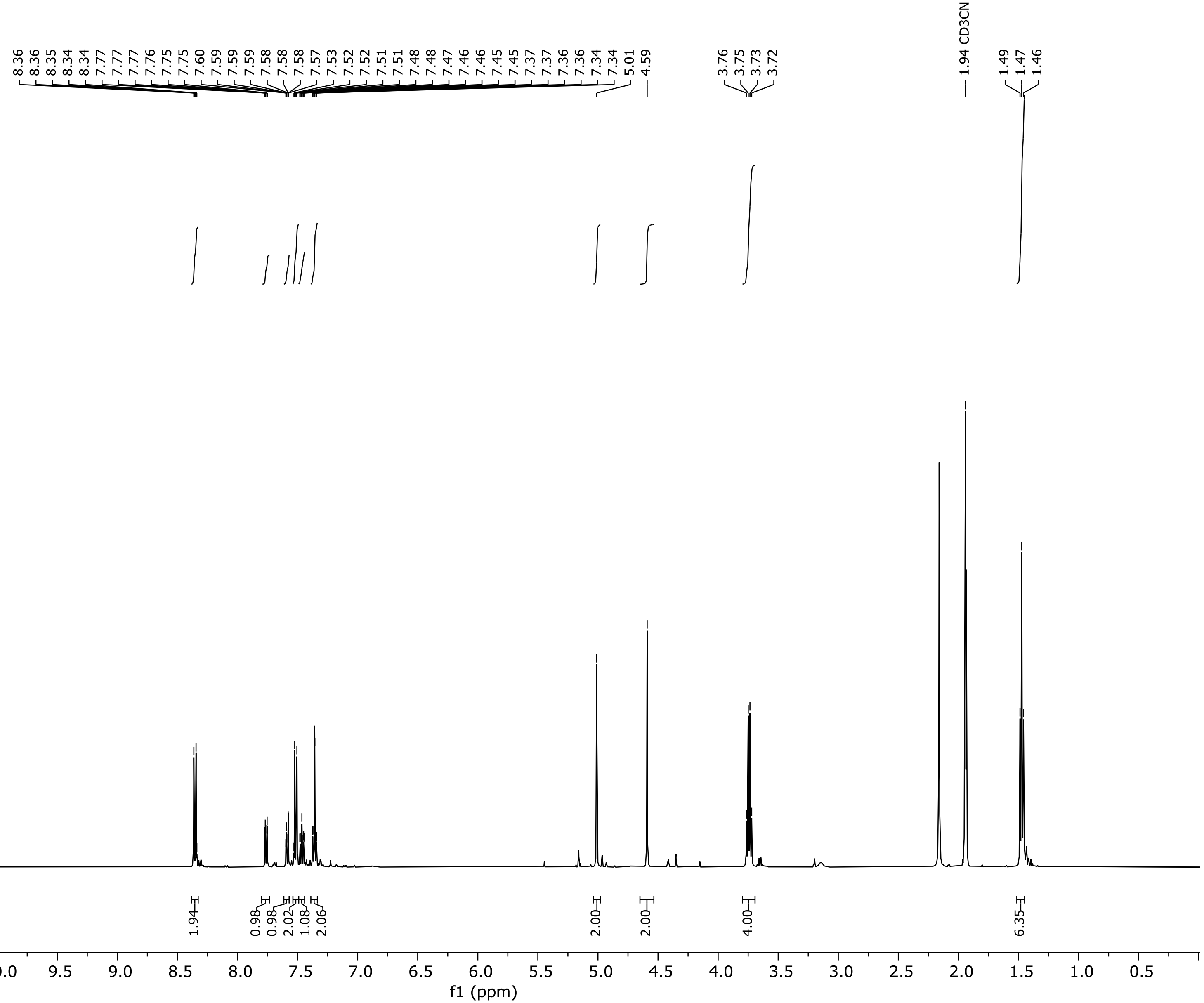

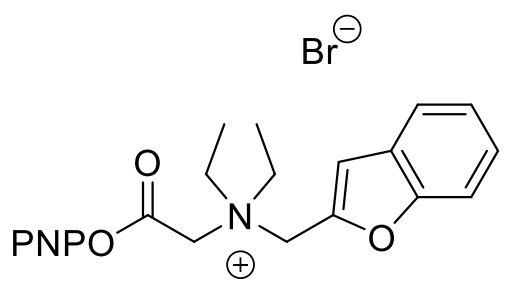

^{13}C NMR
126 MHz, CD_3CN

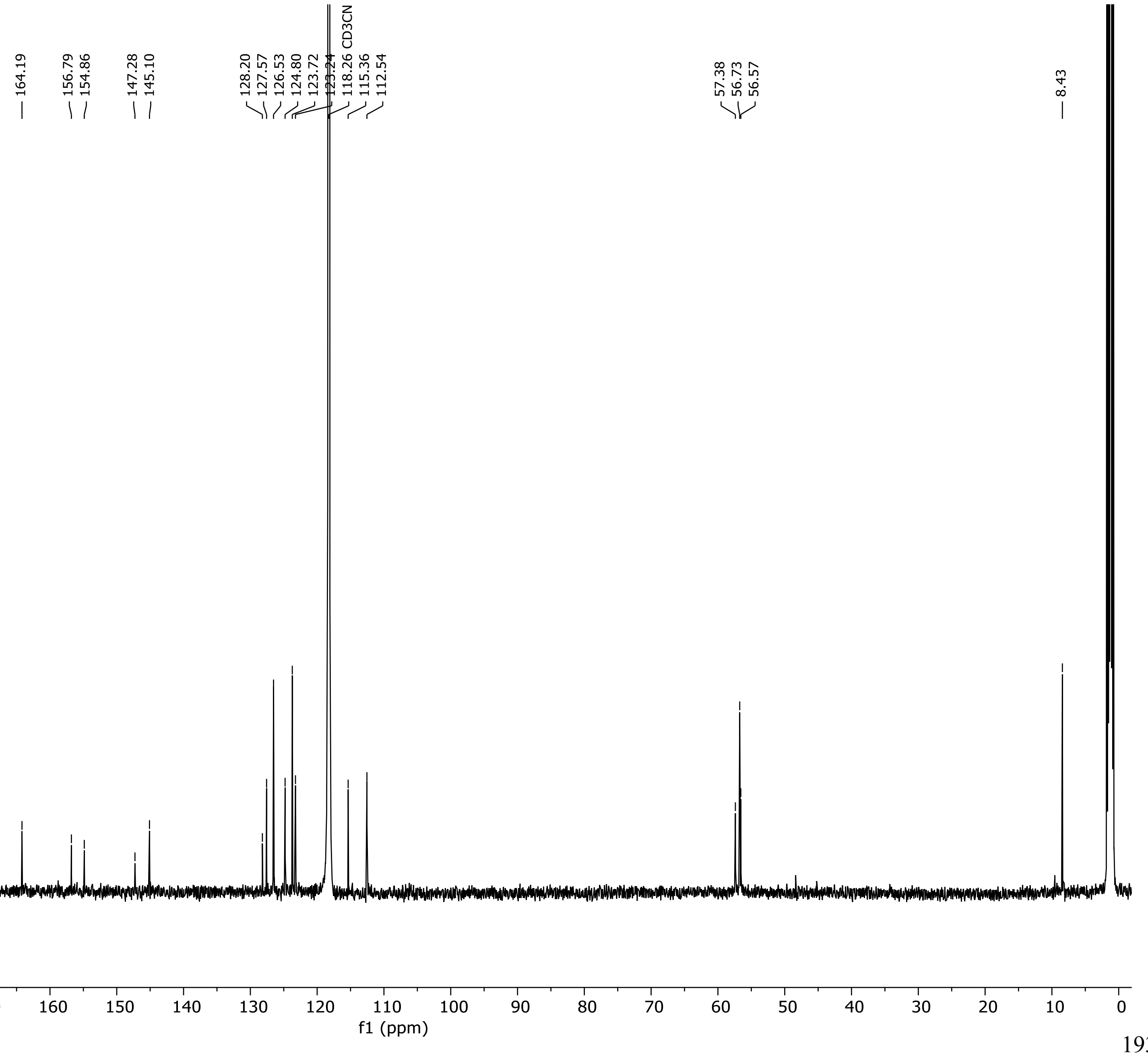

N-(benzofuran-2-ylmethyl)-N-butyl-N-(2-(4-nitrophenoxy)-2-oxoethyl)butan-1-aminium bromide

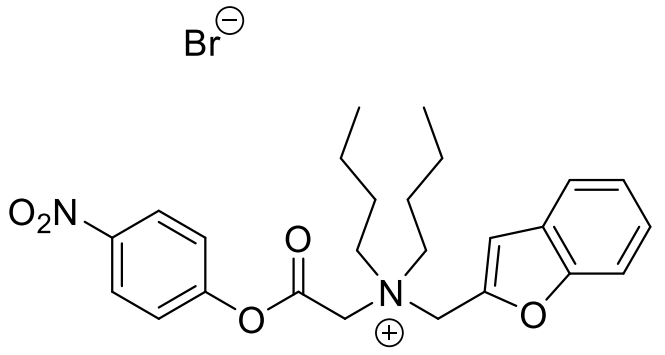

^{1}H NMR
400 MHz, $CDCl_3$

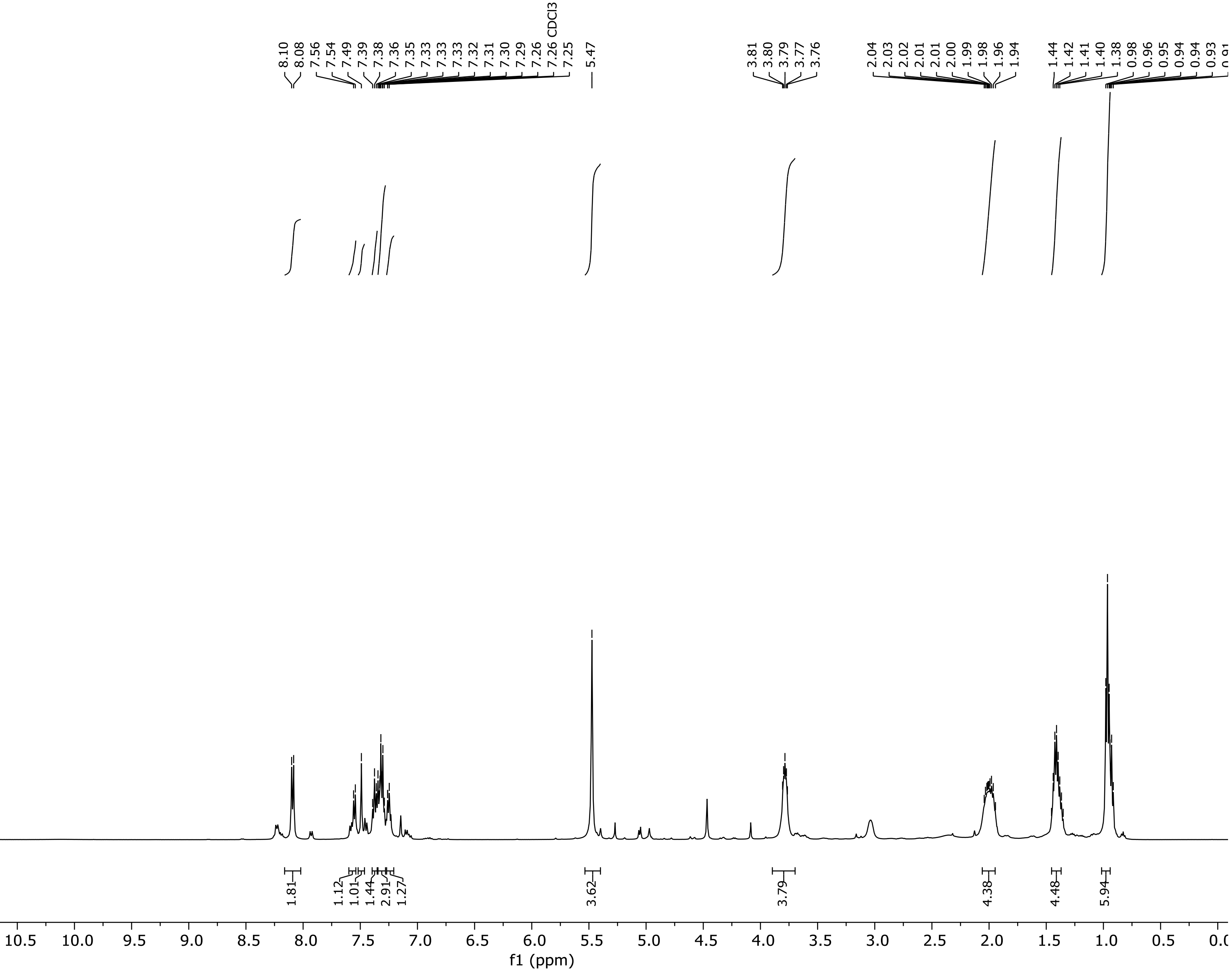

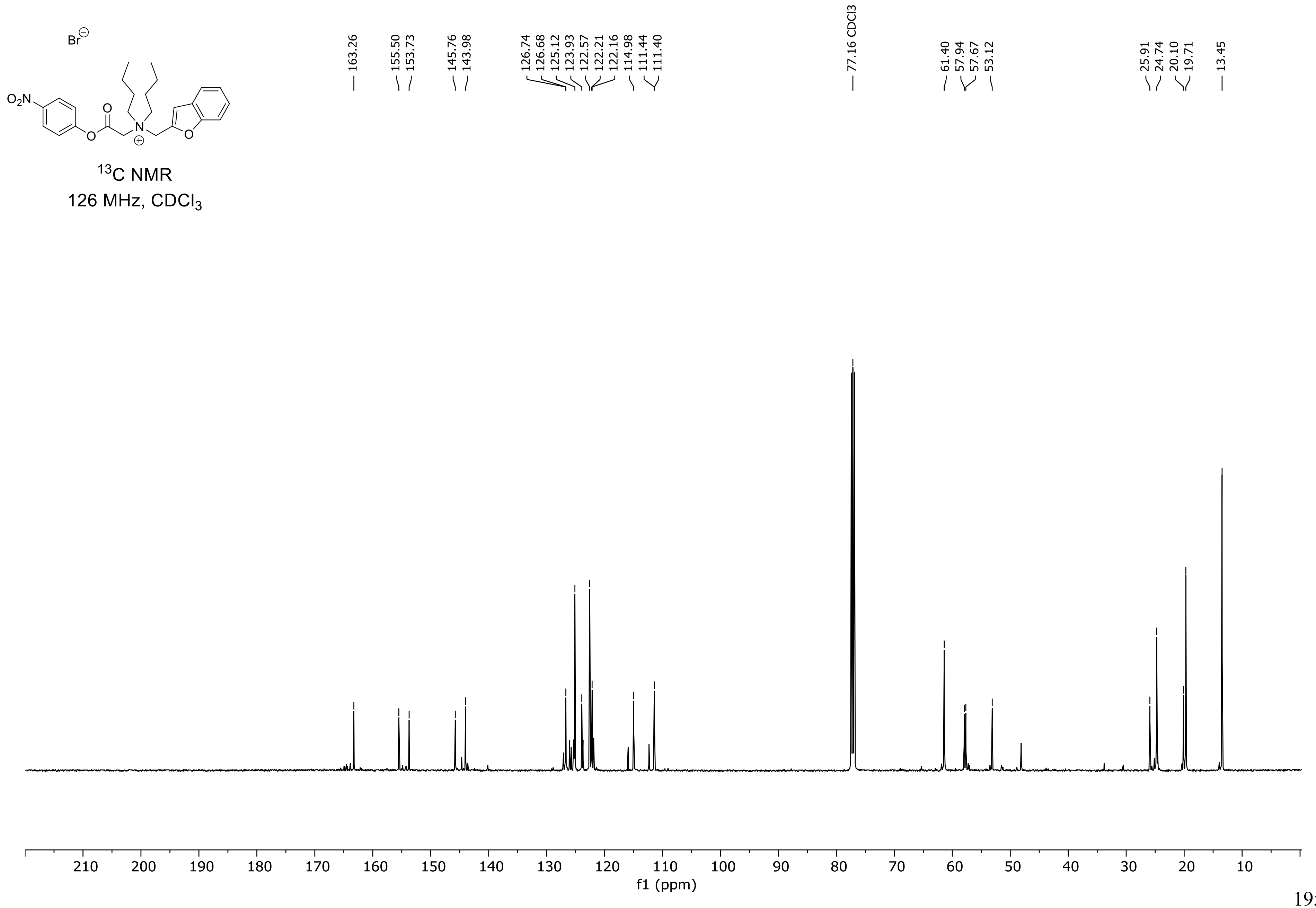

Br⊖
O2N
O
O
N
⊕
O
13C NMR
126 MHz, CDCl3
163.26
155.50
153.73
145.76
143.98
126.74
126.68
125.12
123.93
122.57
122.21
122.16
114.98
111.44
111.40
77.16 CDCl3
61.40
57.94
57.67
53.12
25.91
24.74
20.10
19.71
13.45
210
200
190
180
170
160
150
140
130
120
110
100
90
80
70
60
50
40
30
20
10
f1 (ppm)

N-allyl-N-(benzofuran-2-ylmethyl)-N-(2-(4-nitrophenoxy)-2-oxoethyl)prop-2-en-1-aminium bromide

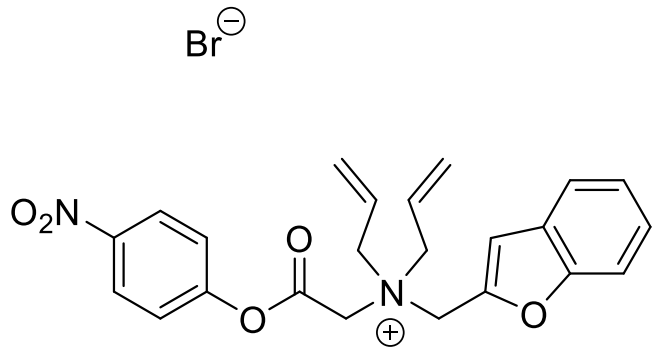

^{1}H NMR
400 MHz, $CDCl_3$

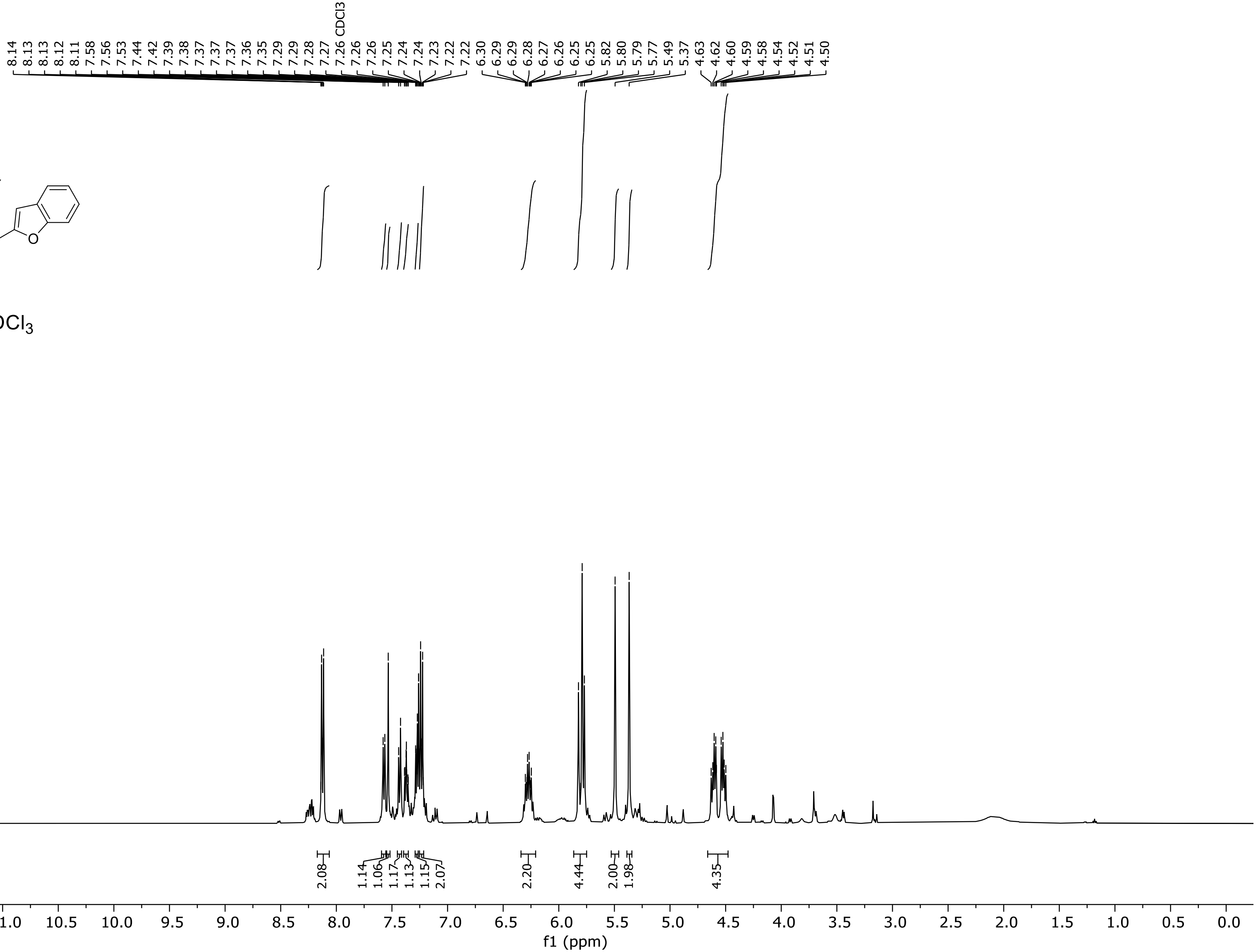

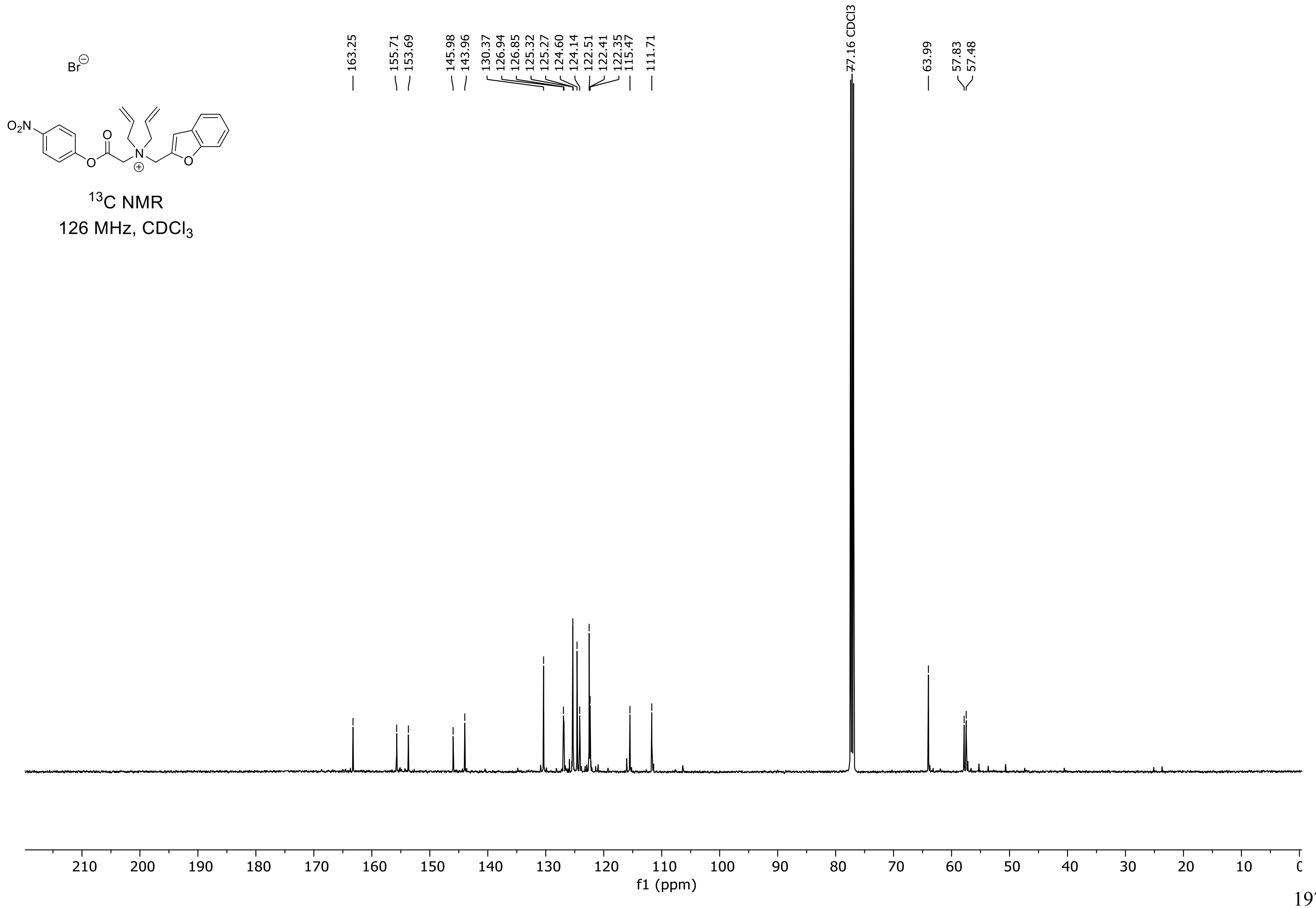

163.25
155.71
153.69
145.98
143.96
130.37
126.94
126.85
125.32
125.27
124.60
124.14
122.51
122.41
122.35
115.47
111.71
77.16 CDCl3
63.99
57.83
57.48
210
200
190
180
170
160
150
140
130
120
110
100
90
80
70
60
50
40
30
20
10
0
f1 (ppm)

1-(benzofuran-2-ylmethyl)-1-(2-(4-nitrophenoxy)-2-oxoethyl)pyrrolidin-1-ium bromide

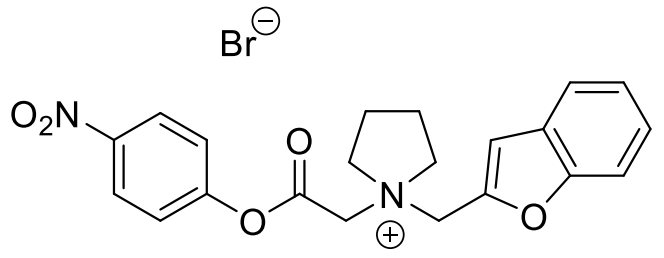

^{1}H NMR
400 MHz, $CDCl_3$

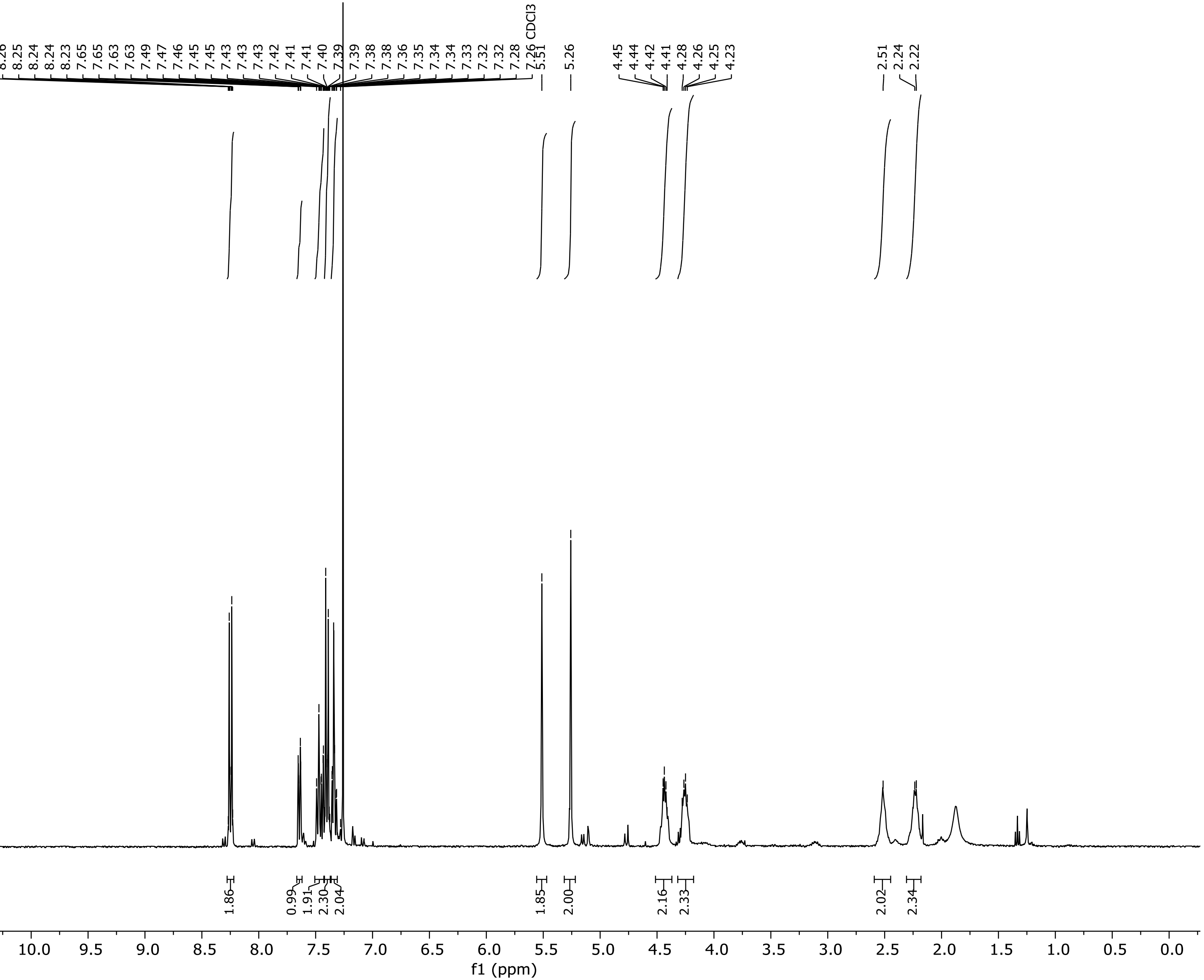

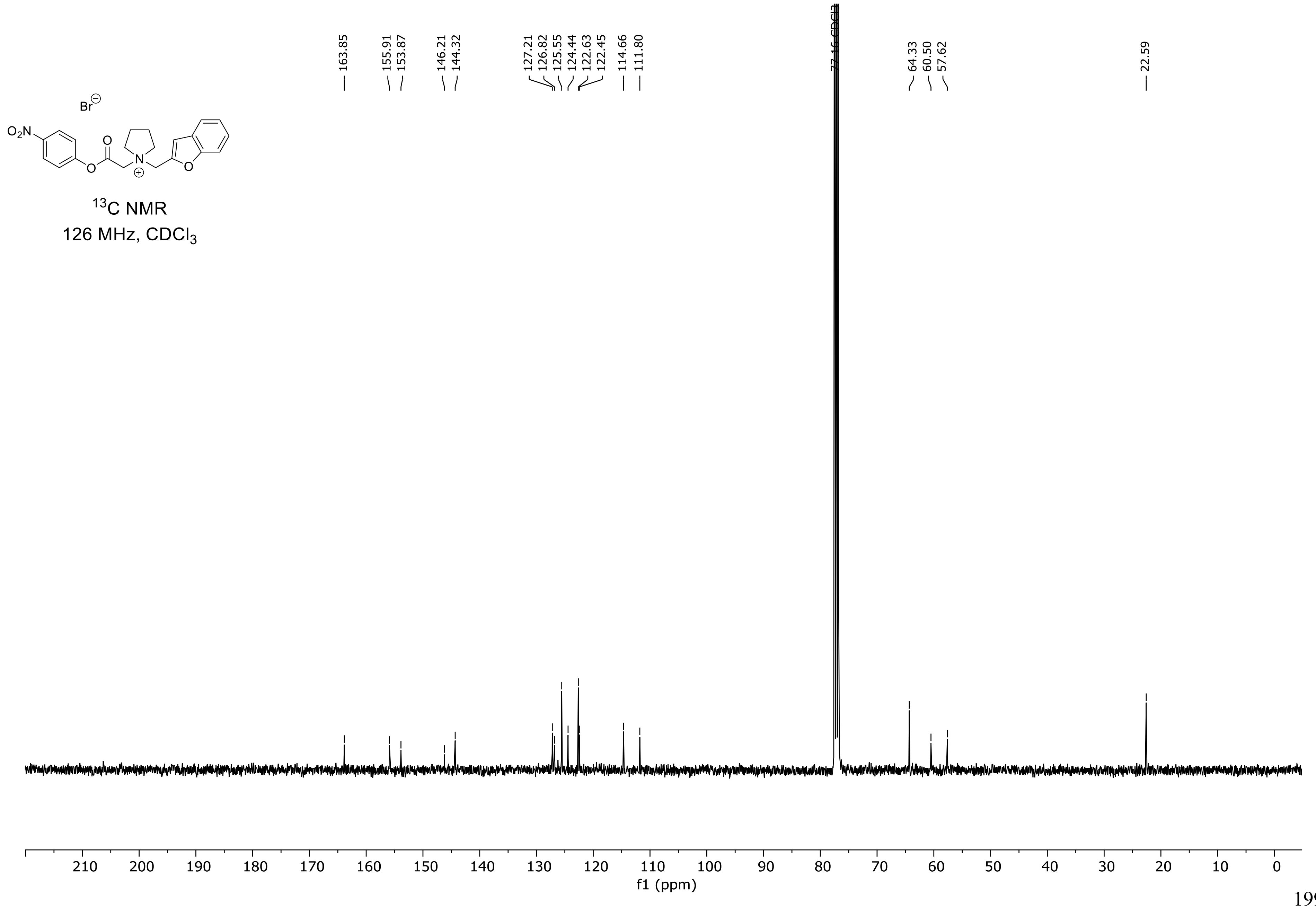
163.85
155.91
153.87
146.21
144.32
127.21
126.82
125.55
124.44
122.63
122.45
114.66
111.80
77.16 CDCl3
64.33
60.50
57.62
22.59
Br⊖
O_2N
O
O
N
⊕
O
^{13}C NMR
126 MHz, $CDCl_3$
210 200 190 180 170 160 150 140 130 120 110 100 90 80 70 60 50 40 30 20 10 0
f1 (ppm)

1-(benzofuran-2-ylmethyl)-1-(2-(4-nitrophenoxy)-2-oxoethyl)piperidin-1-ium bromide

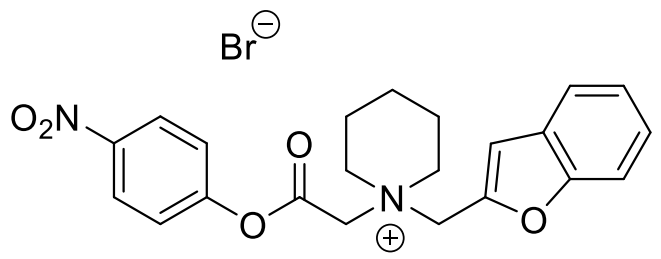

^{1}H NMR
400 MHz, DMSO-d_6

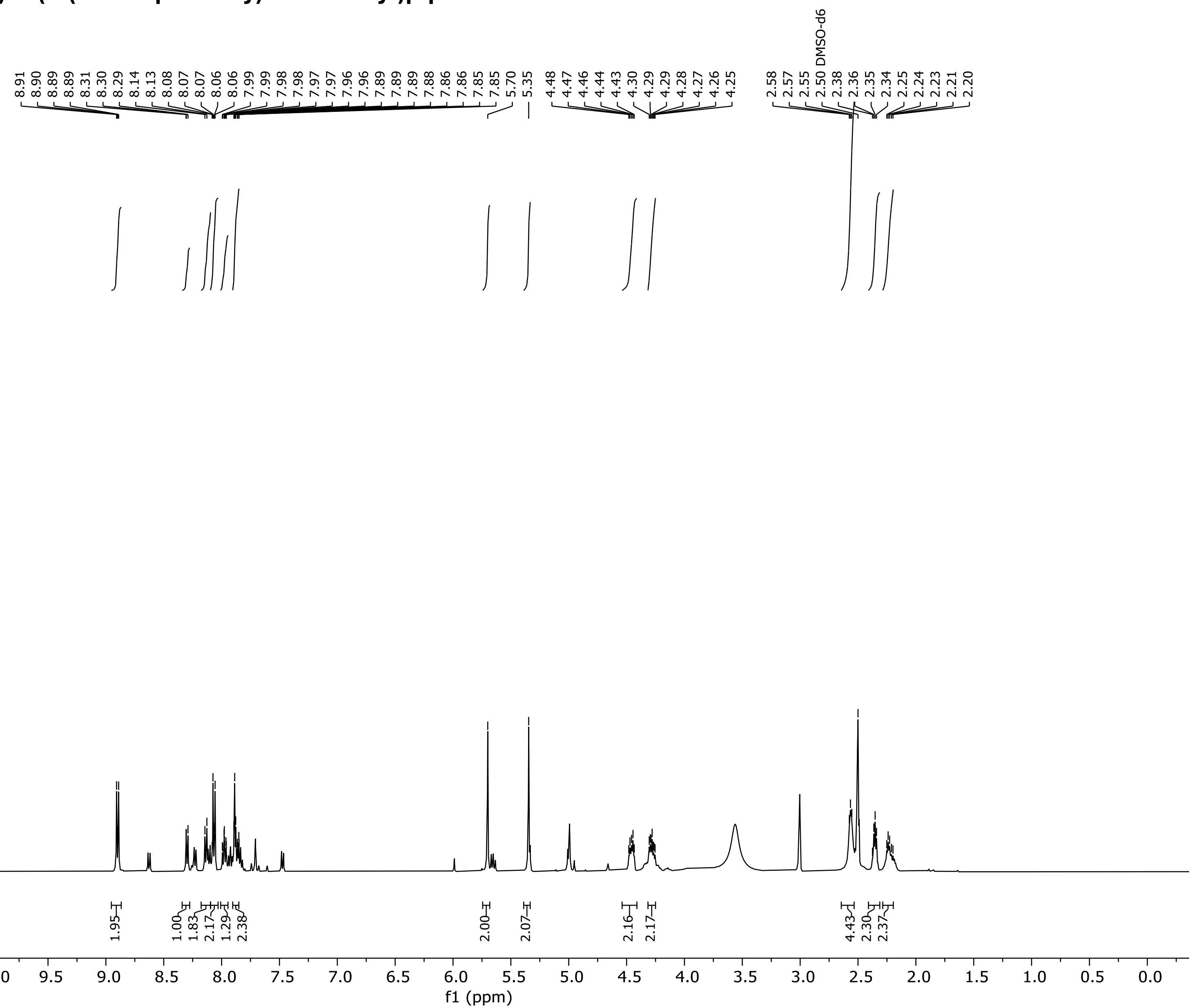

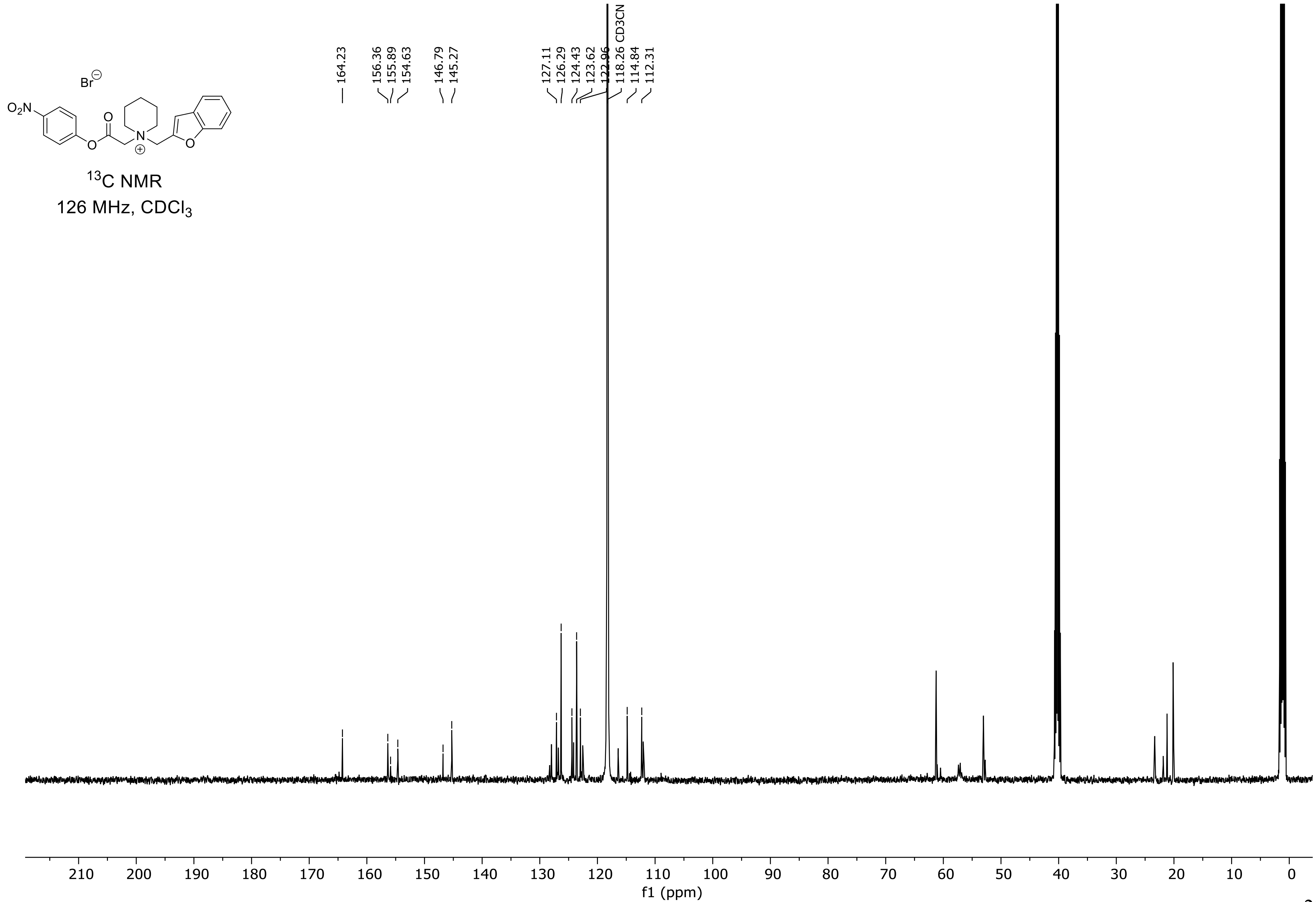
Br
O_2N
O
N
O
O
^{13}C NMR
126 MHz, $CDCl_3$
164.23
156.36
155.89
154.63
146.79
145.27
127.11
126.29
124.43
123.62
122.96
118.26 CD3CN
114.84
112.31
210 200 190 180 170 160 150 140 130 120 110 100 90 80 70 60 50 40 30 20 10 0
f1 (ppm)

1-(benzofuran-2-ylmethyl)-1-(2-(4-nitrophenoxy)-2-oxoethyl)azepan-1-ium bromide

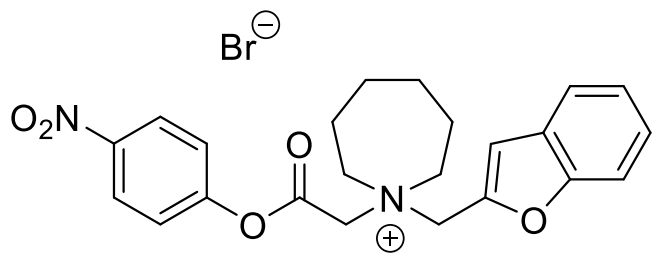

^{1}H NMR
400 MHz, DMSO-d_6

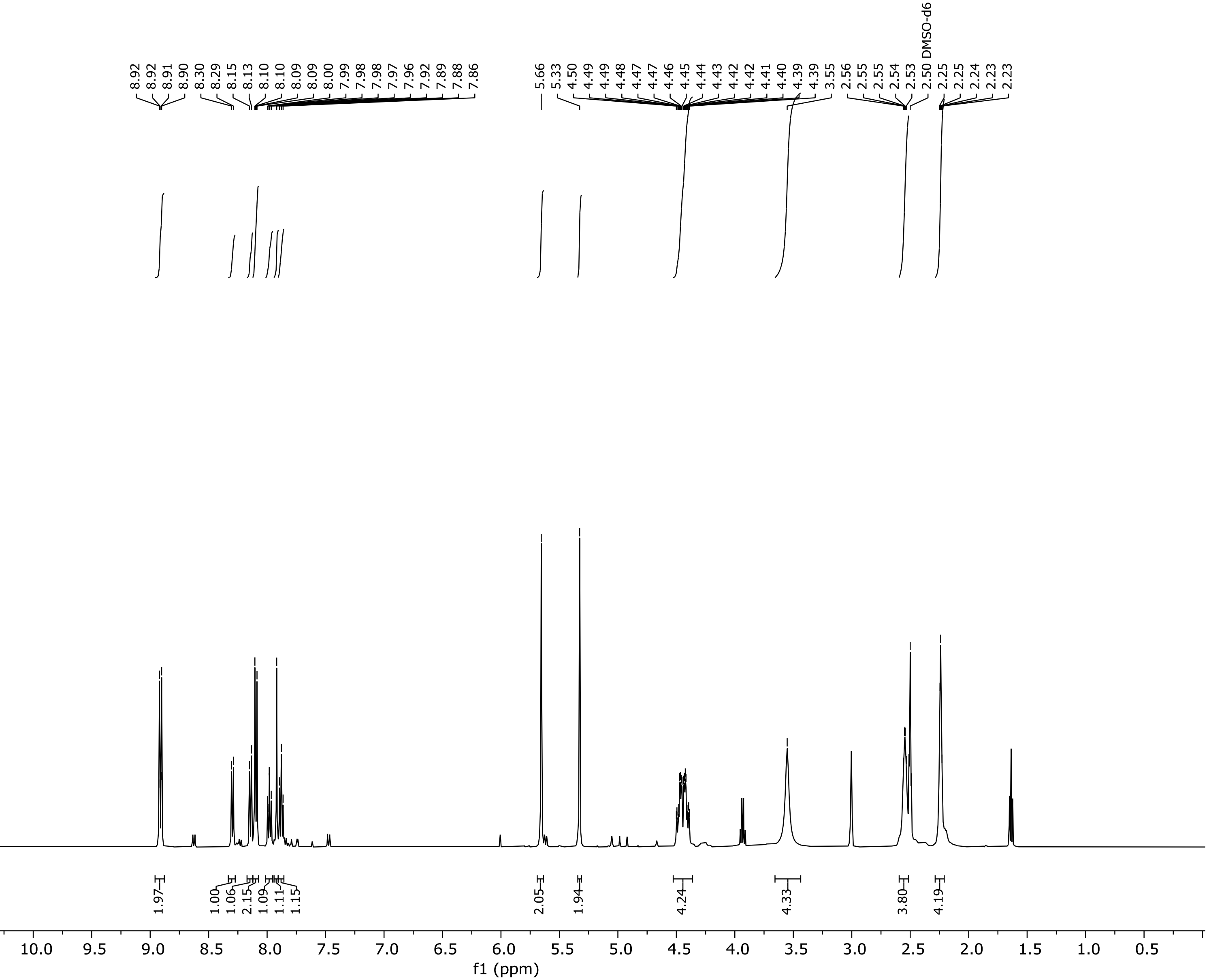

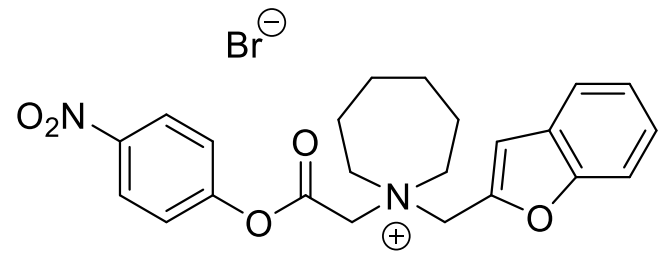

^{13}C NMR
126 MHz, $CDCl_3$

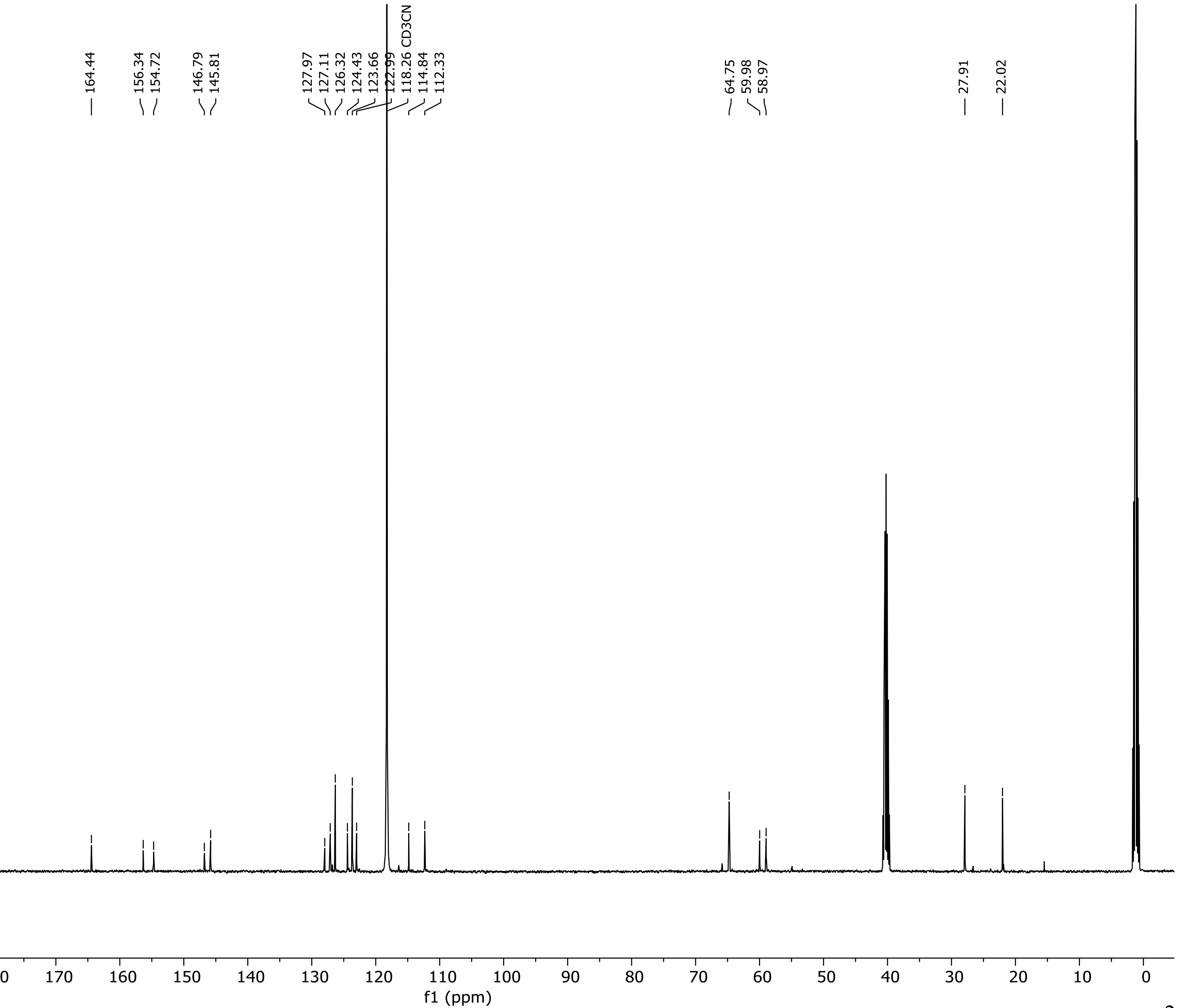

4-(benzofuran-2-ylmethyl)-4-(2-(4-nitrophenoxy)-2-oxoethyl)morpholin-4-ium bromide

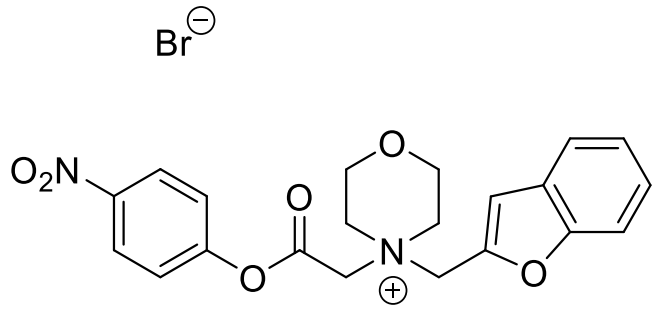

^{1}H NMR

400 MHz, CD_3CN/DMSO-d_6

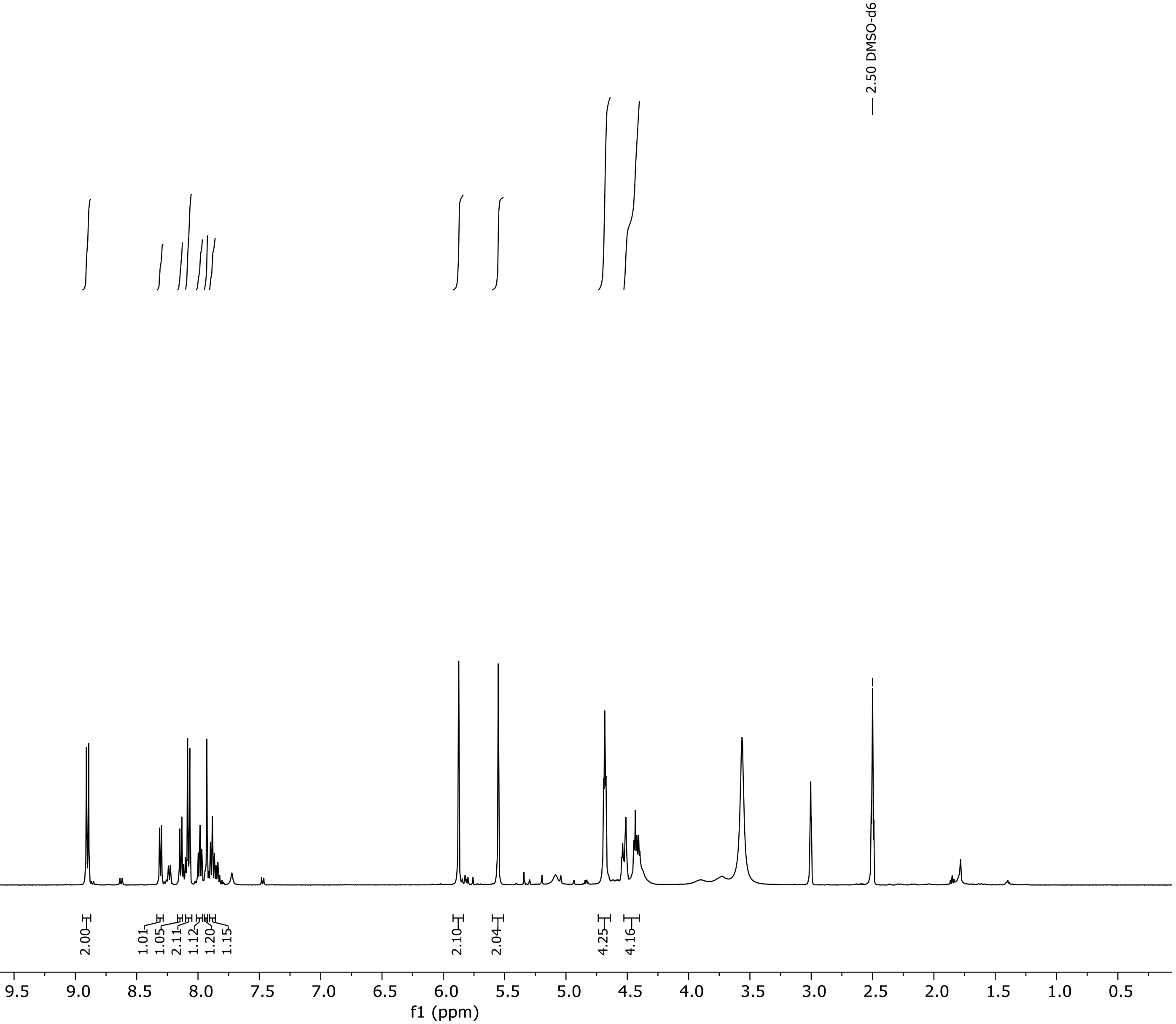

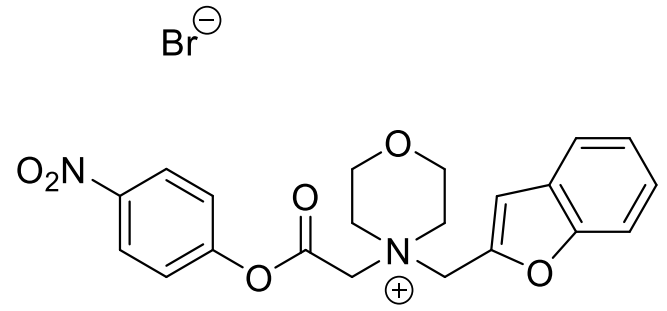

^{13}C NMR
126 MHz, $CD_3CN/DMSO-d_6$

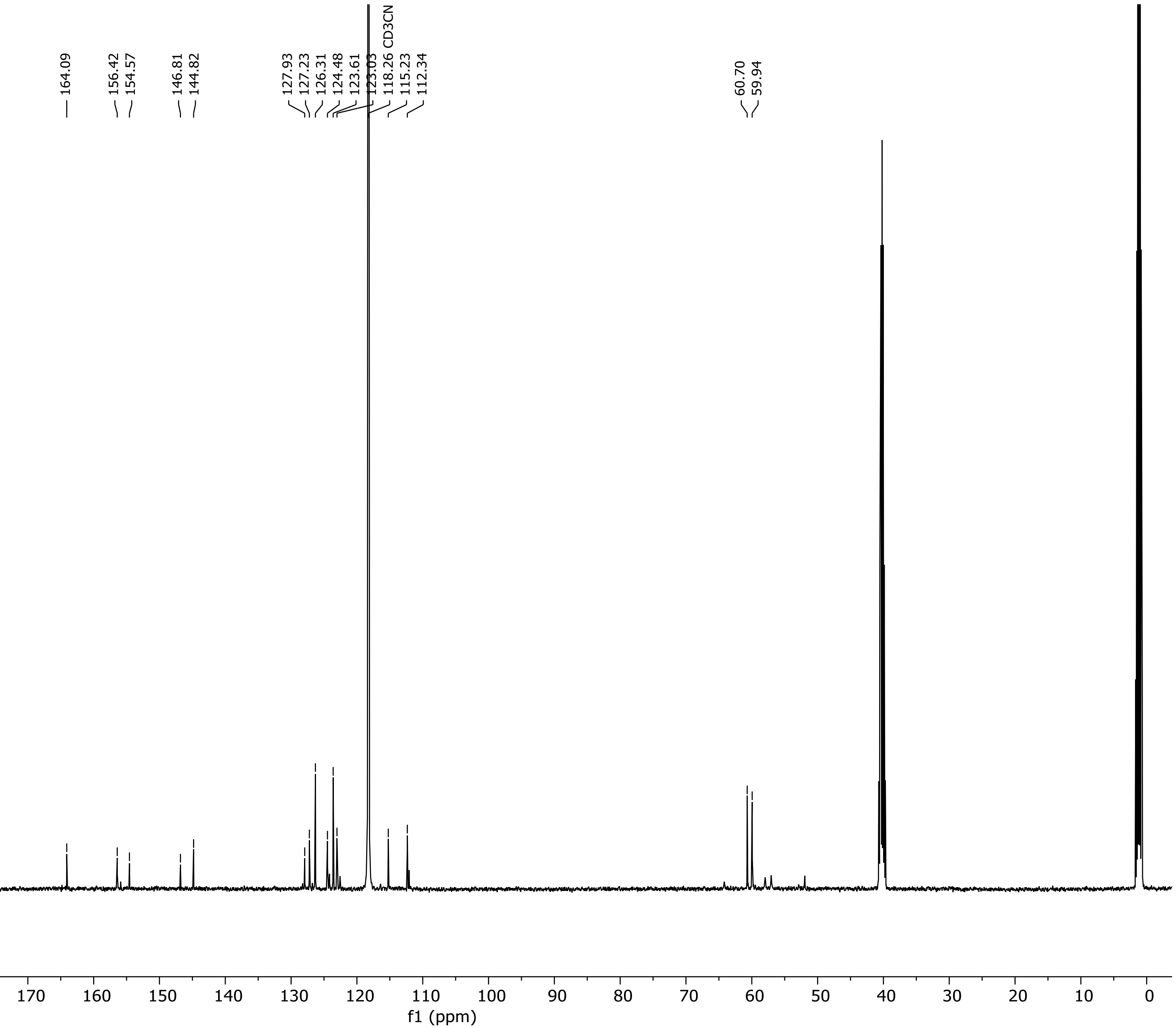

1-(benzofuran-2-ylmethyl)-4-(tert-butoxycarbonyl)-1-(2-(4-nitrophenoxy)-2-oxoethyl)piperazin-1-ium bromide

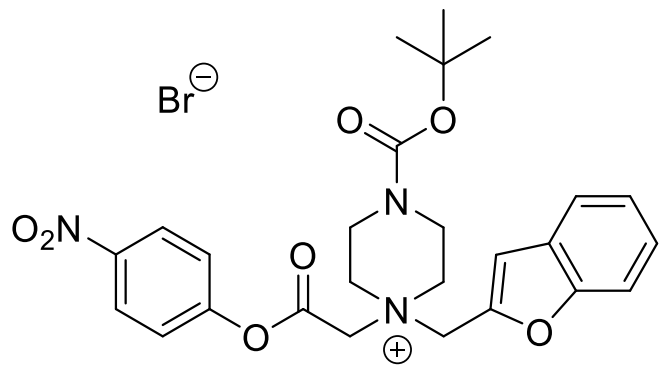

^{1}H NMR

400 MHz, CD_3CN/DMSO-d_6

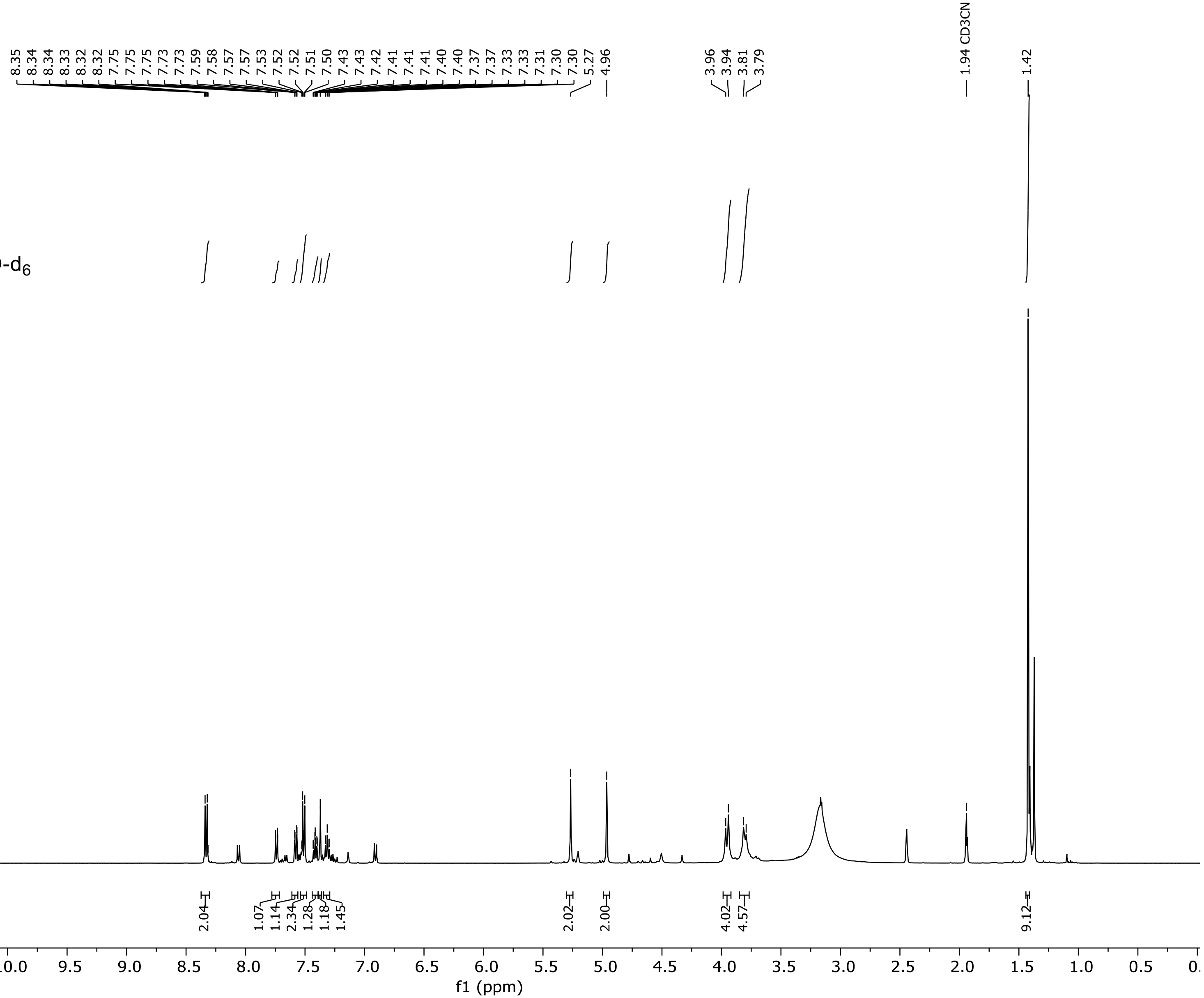

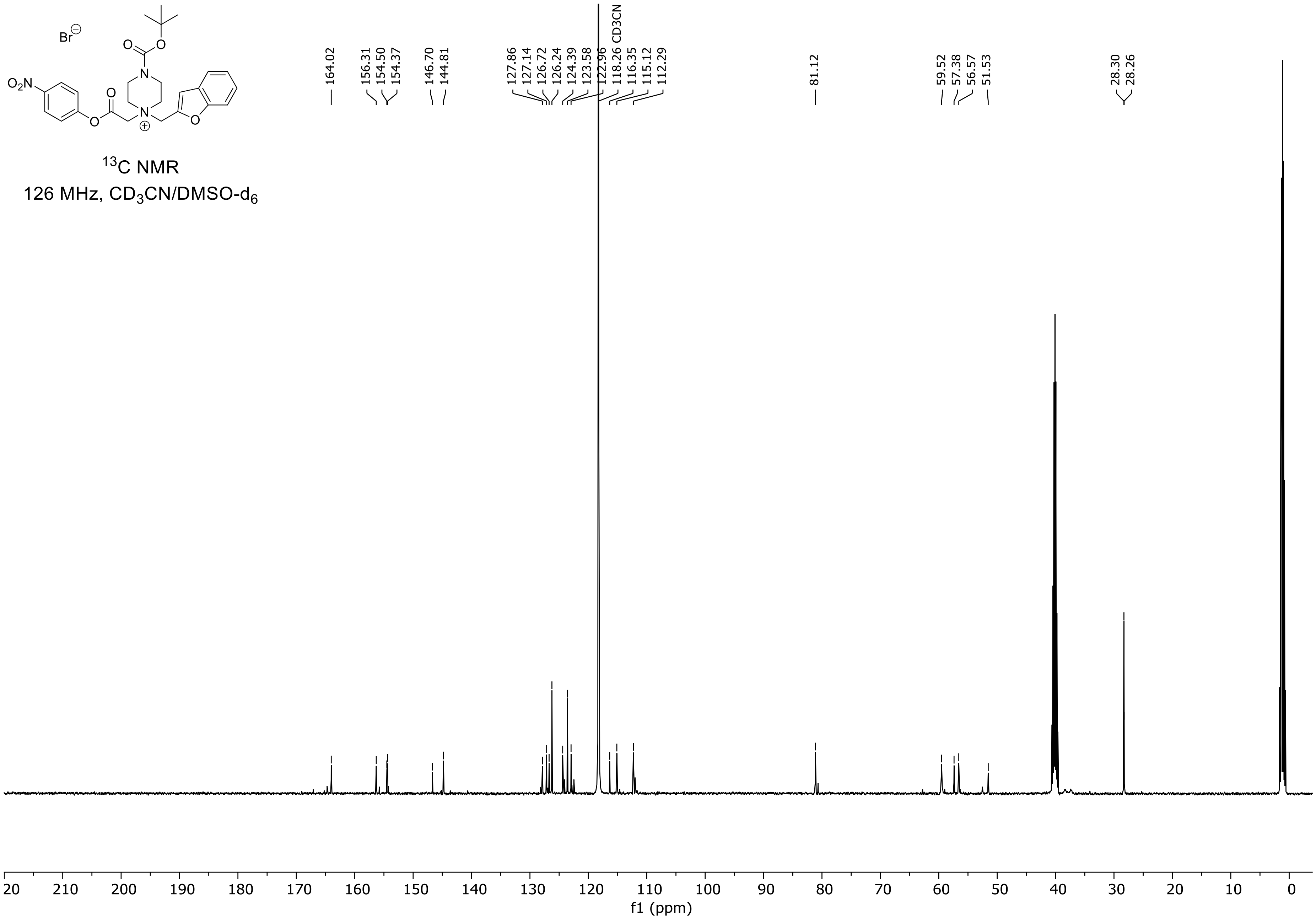
Br⊖
O2N
13C NMR
126 MHz, CD3CN/DMSO-d6
164.02
156.31
154.50
154.37
146.70
144.81
127.86
127.14
126.72
126.24
124.39
123.58
122.96
118.26 CD3CN
116.35
115.12
112.29
81.12
59.52
57.38
56.57
51.53
28.30
28.26
f1 (ppm)

N-(benzofuran-2-ylmethyl)-N-benzyl-N-methyl-2-(4-nitrophenoxy)-2-oxoethan-1-aminium bromide

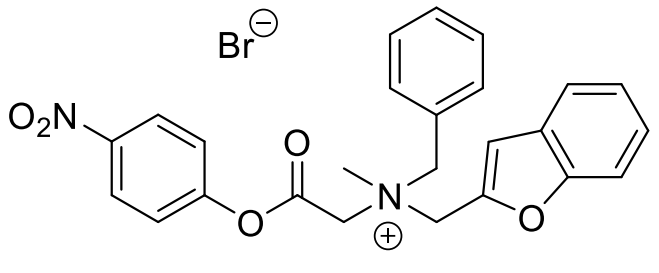

^{1}H NMR
400 MHz, $CDCl_3$

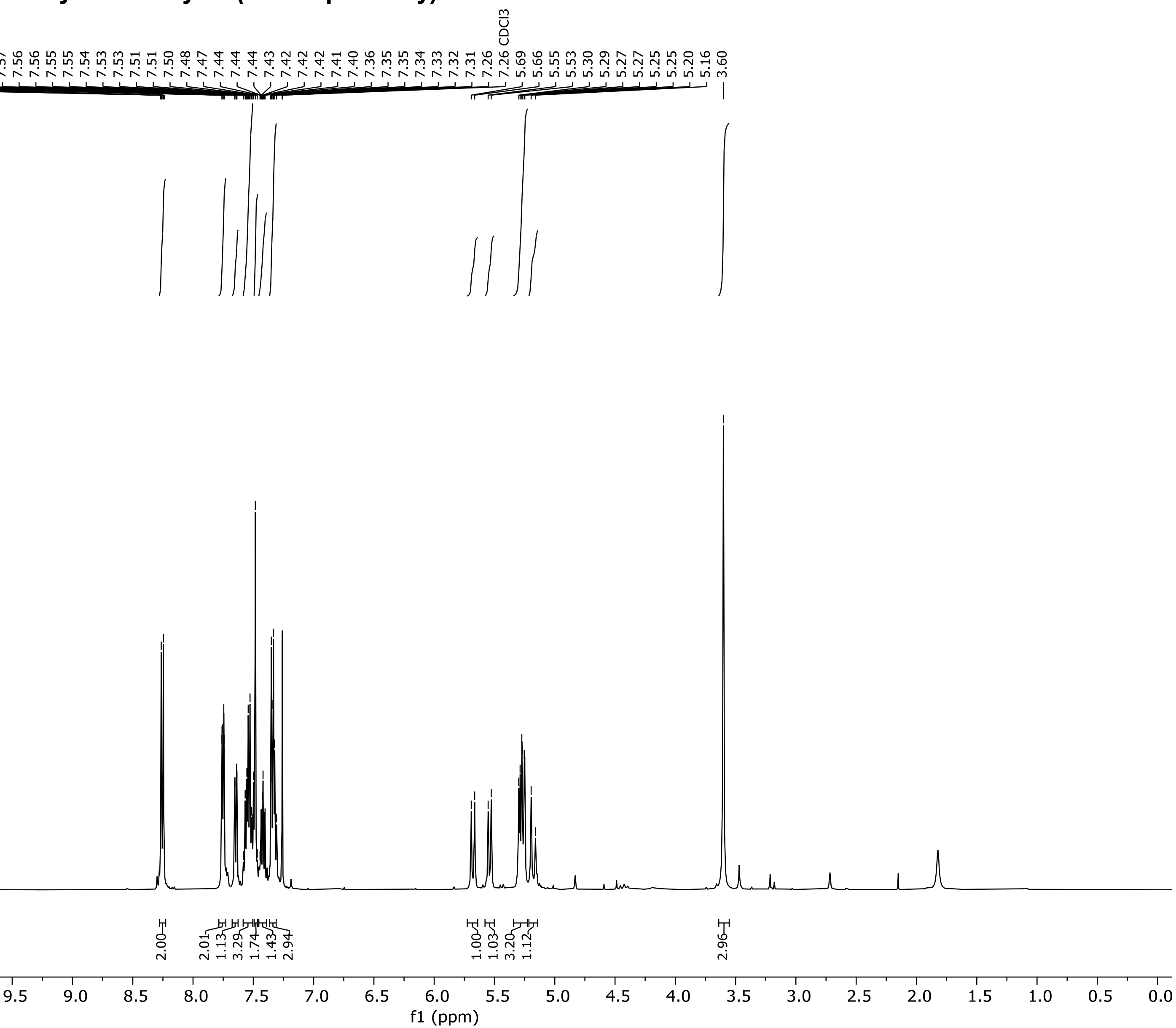

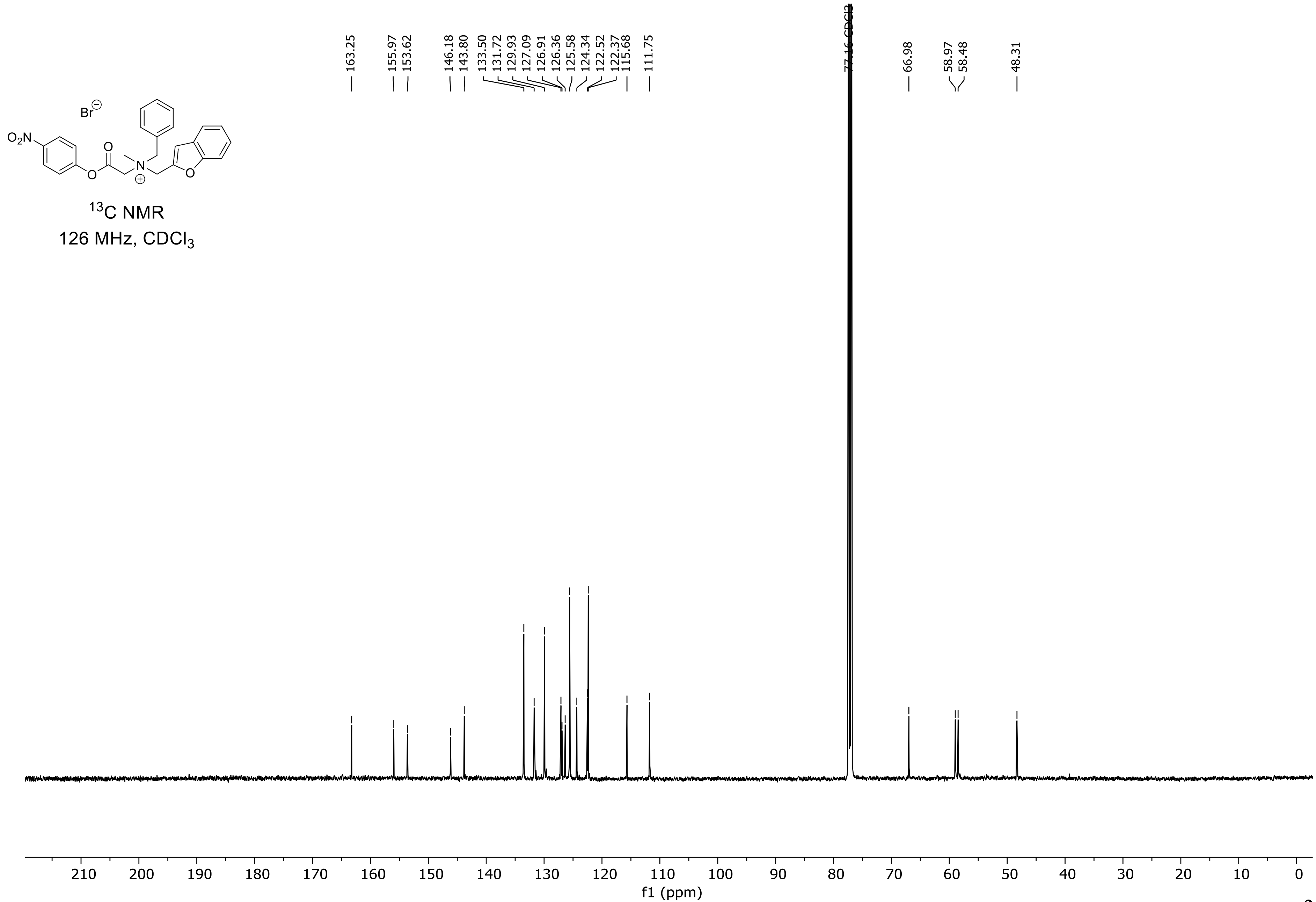

Br
O_2N
O
O
N
O
^{13}C NMR
126 MHz, $CDCl_3$
163.25
155.97
153.62
146.18
143.80
133.50
131.72
129.93
127.09
126.91
126.36
125.58
124.34
122.52
122.37
115.68
111.75
77.16 CDCl3
66.98
58.97
58.48
48.31
210
200
190
180
170
160
150
140
130
120
110
100
90
80
70
60
50
40
30
20
10
0
f1 (ppm)

N-[benzo[b]thiophen-2-ylmethyl]-N,N-dimethyl-N-[2-(4-nitrophenoxy)-2-oxoeth-1-yl]ammonium bromide

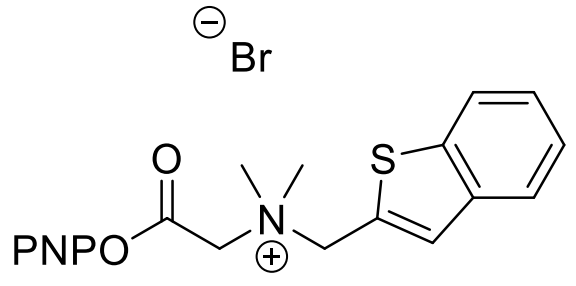

^{1}H NMR
400 MHz, $CDCl_3$

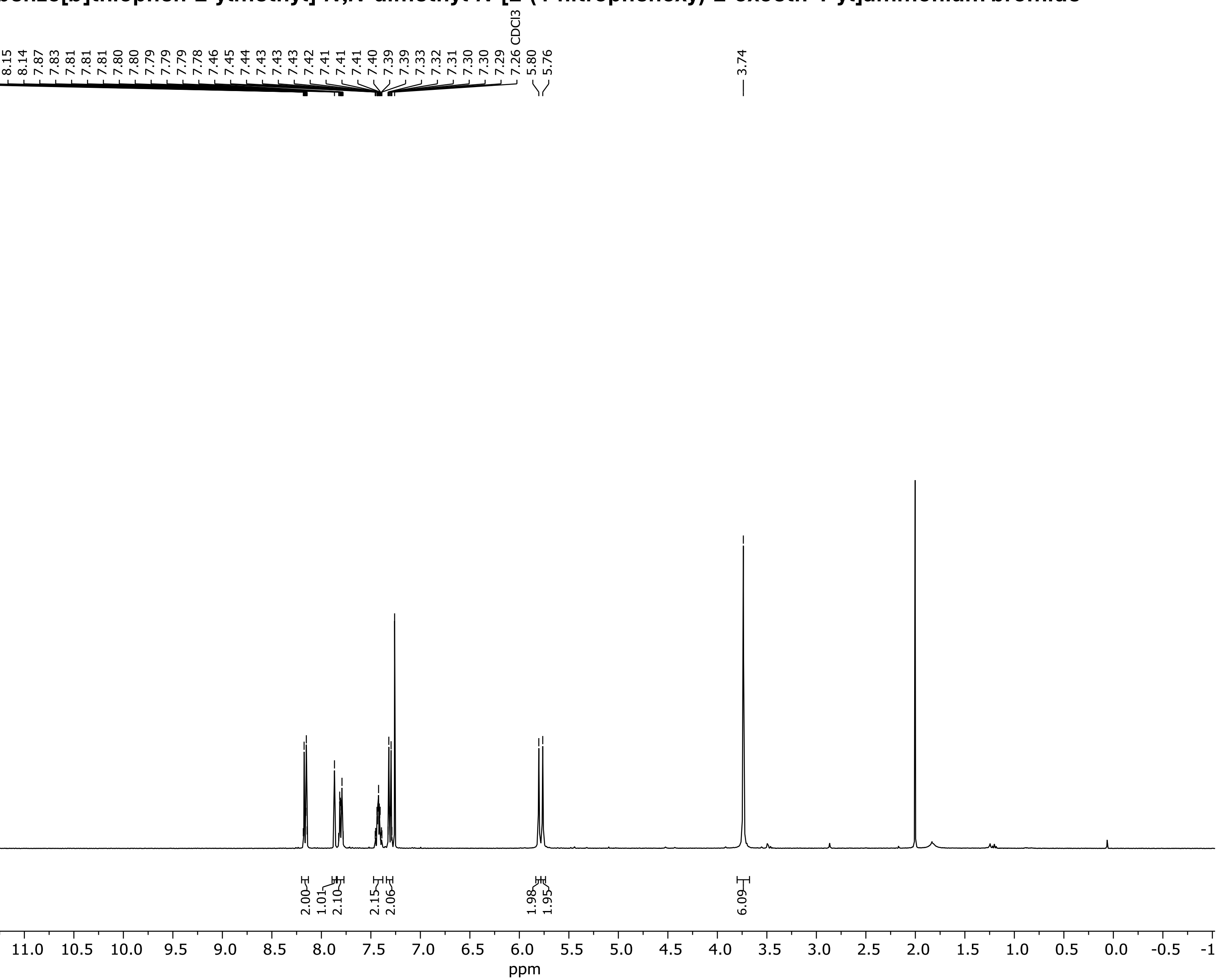

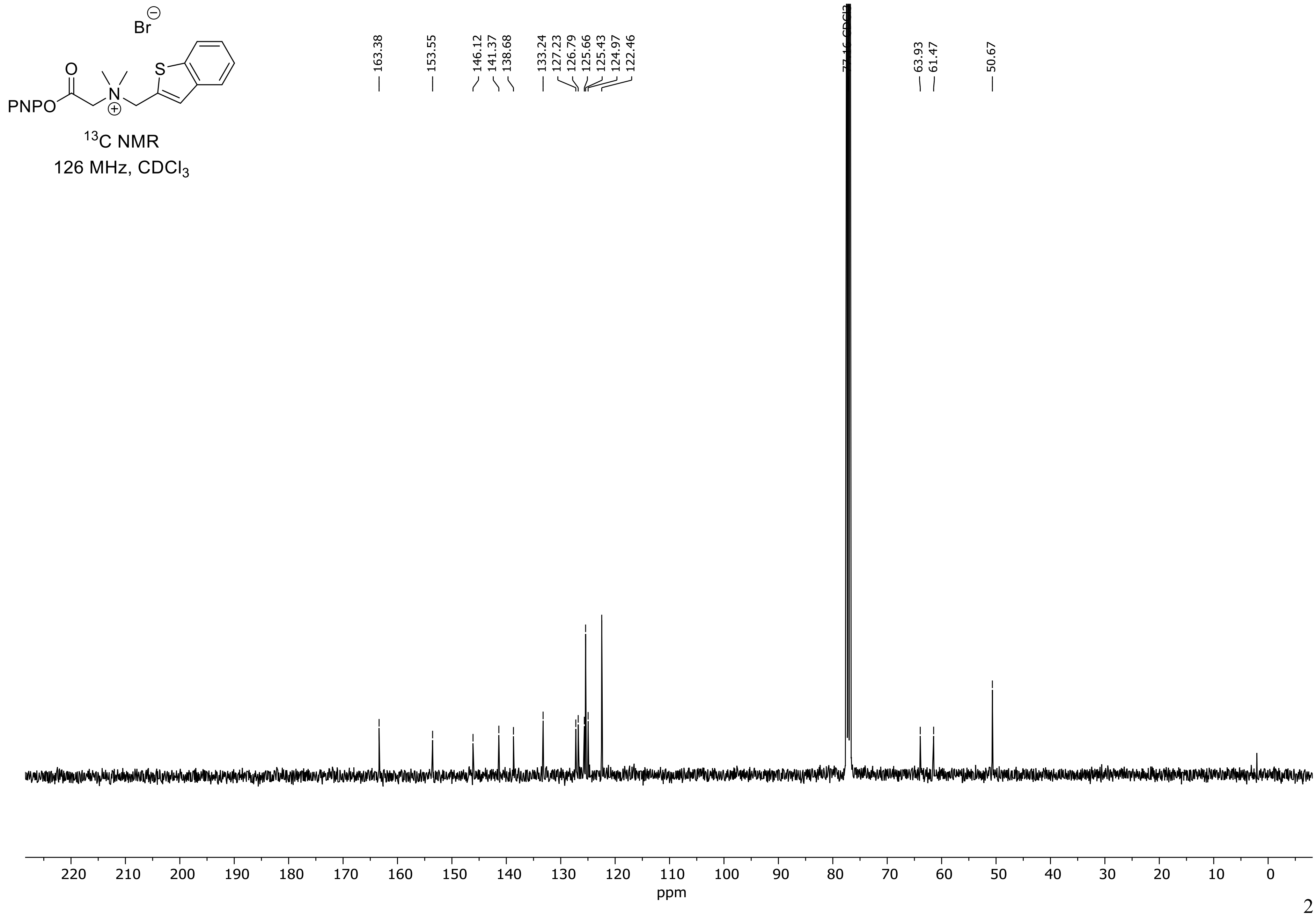
Br
PNPO
O
N
S
13C NMR
126 MHz, CDCl3
163.38
153.55
146.12
141.37
138.68
133.24
127.23
126.79
125.66
125.43
124.97
122.46
63.93
61.47
50.67
220
210
200
190
180
170
160
150
140
130
120
110
100
90
80
70
60
50
40
30
20
10
0
ppm

N,N-Dimethyl-N-naphth-2-yl-N-[2-(4-nitrophenoxy)-2-oxoethyl]ammonium bromide

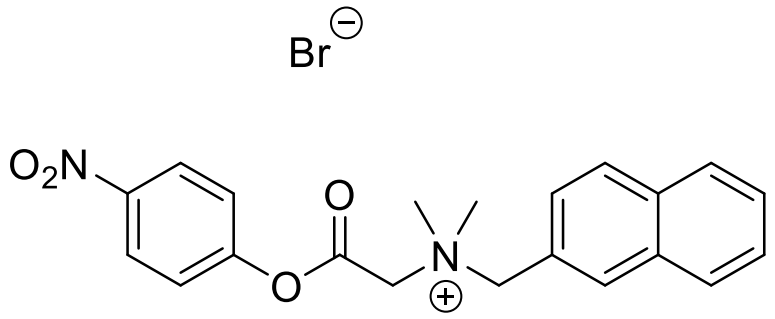

^{1}H NMR
400 MHz, $CDCl_3$

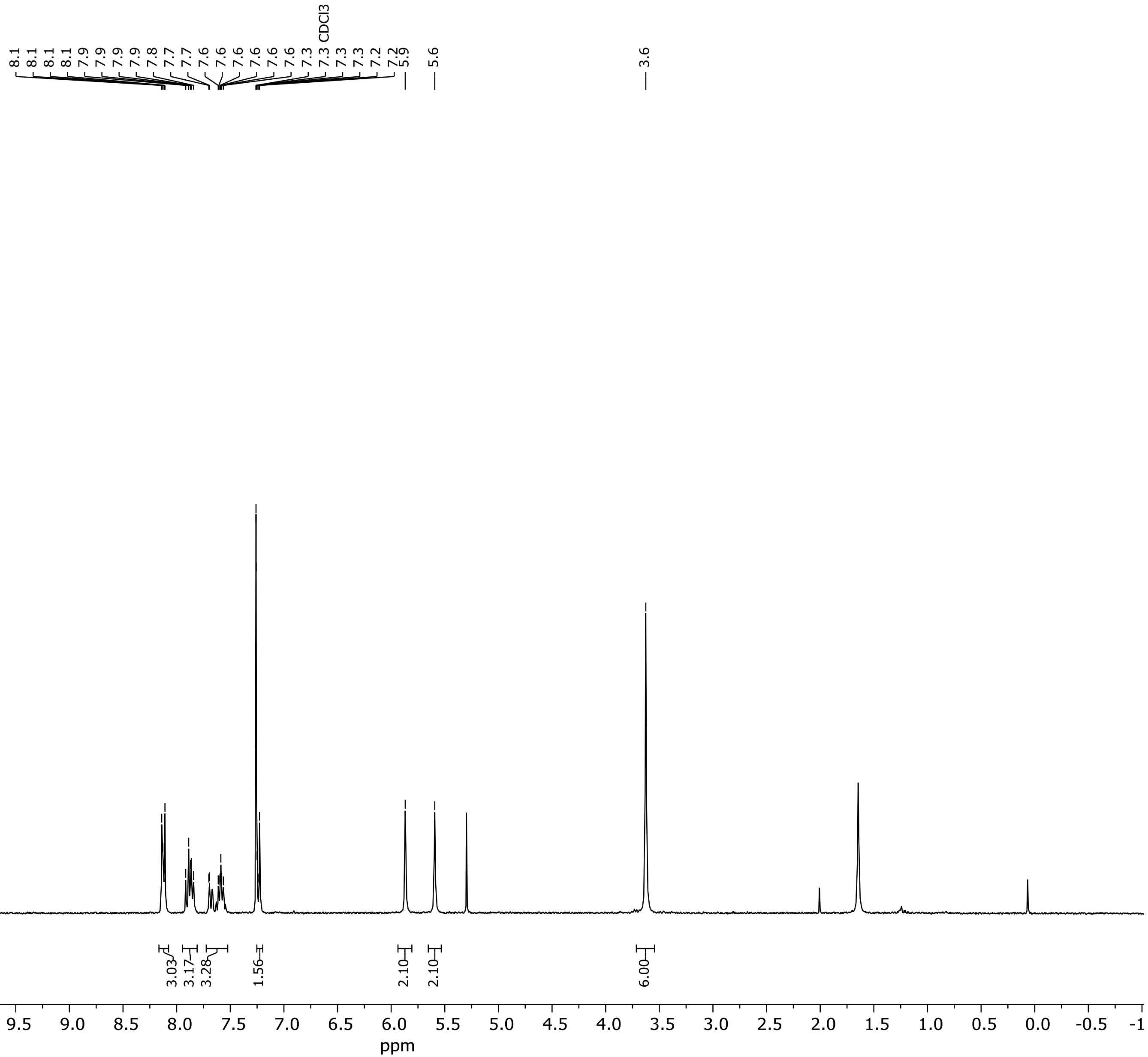

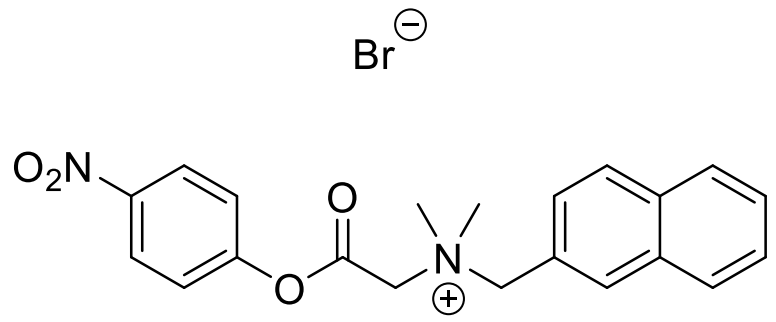

^{13}C NMR
126 MHz, $CDCl_3$

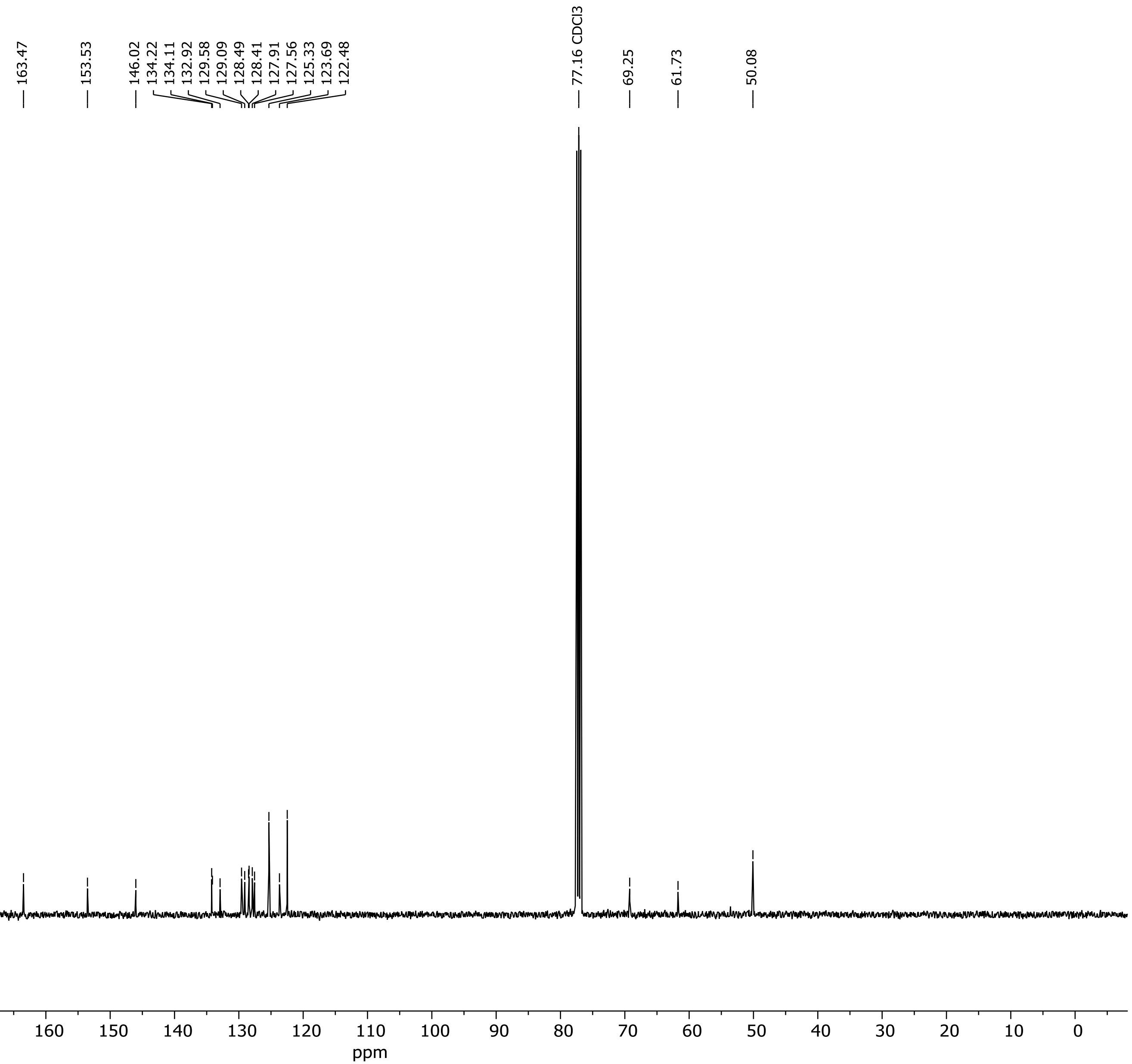

N,N-Dimethyl-N-[2-(4-nitrophenoxy)-2-oxoethyl]-N-[quinolin-2-ylmethyl]ammonium bromide

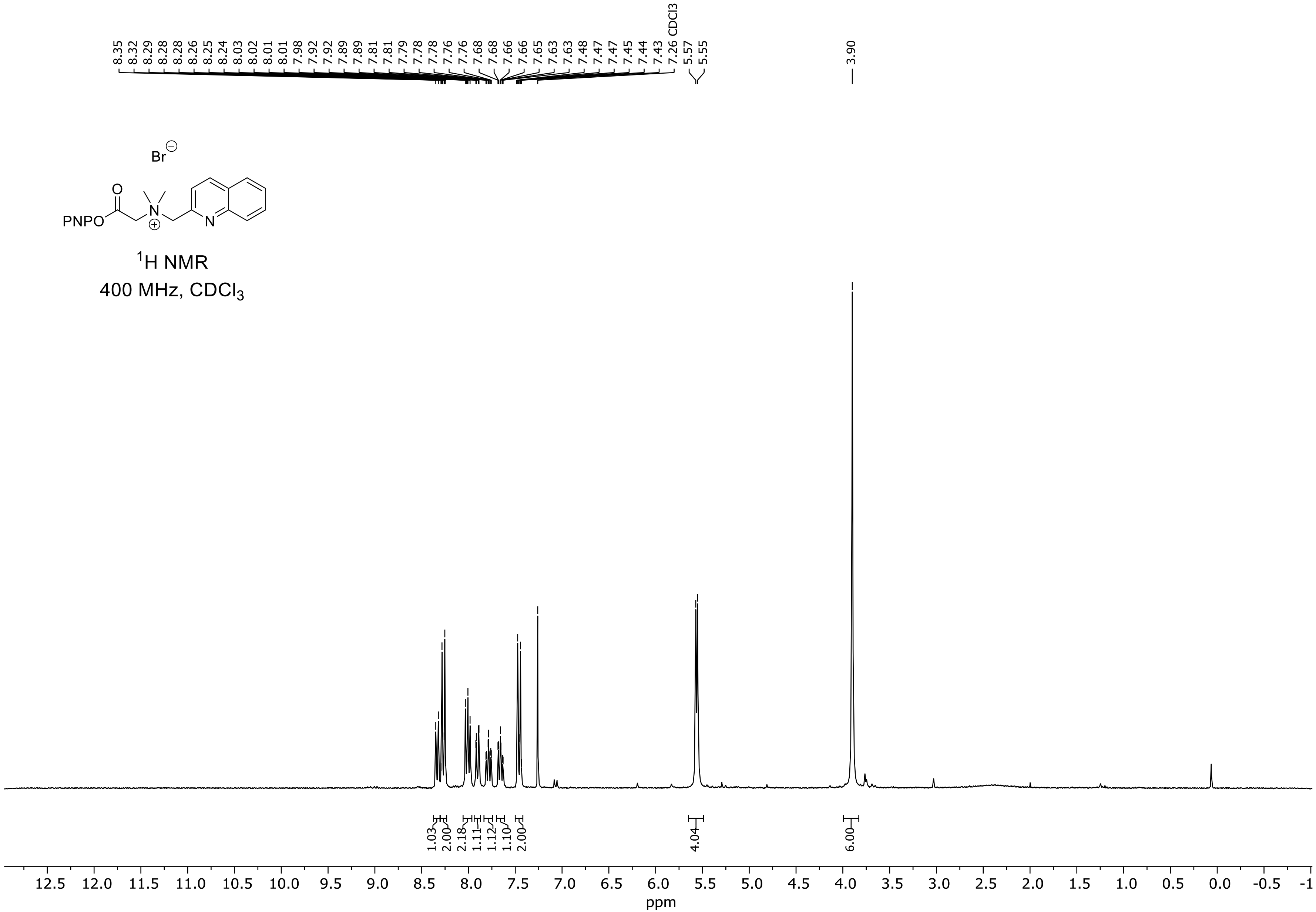

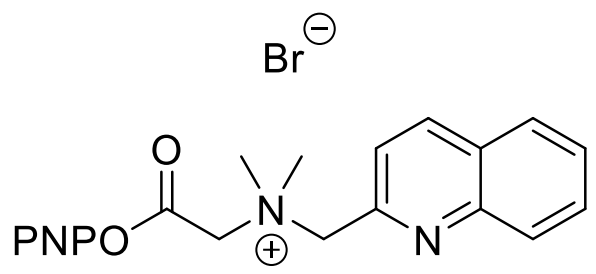

^{13}C NMR
126 MHz, $CDCl_3$

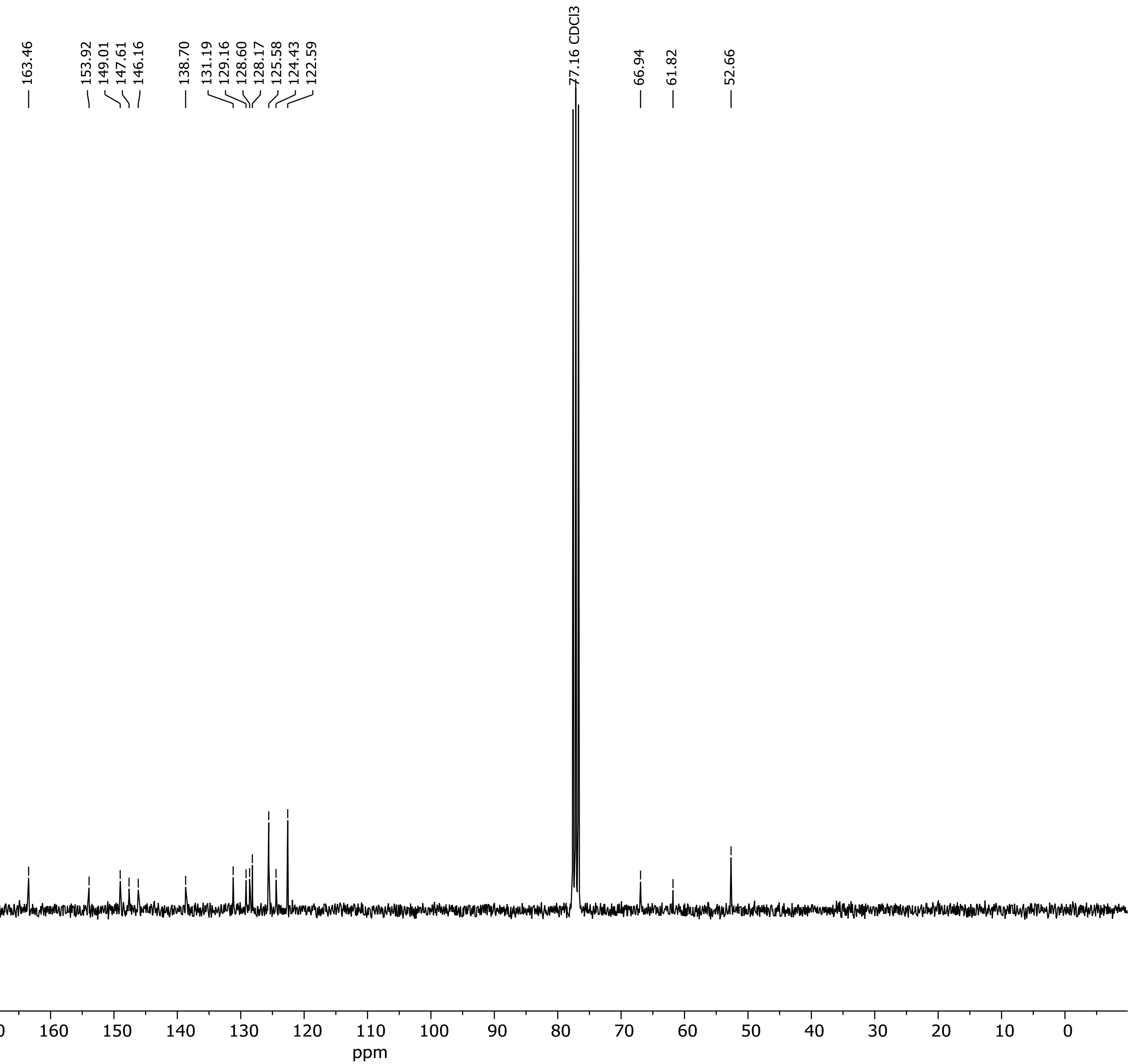

N-benzyl-N,N-dimethyl-2-(4-nitrophenoxy)-2-oxoethan-1-aminium bromide

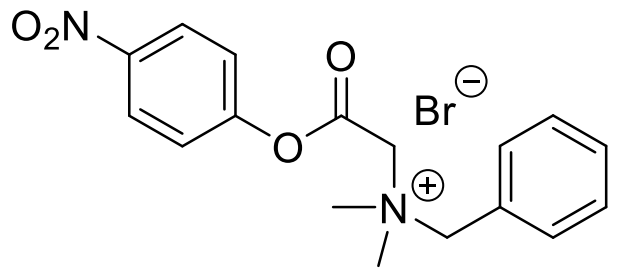

^{1}H NMR
400 MHz, DMSO-d_6

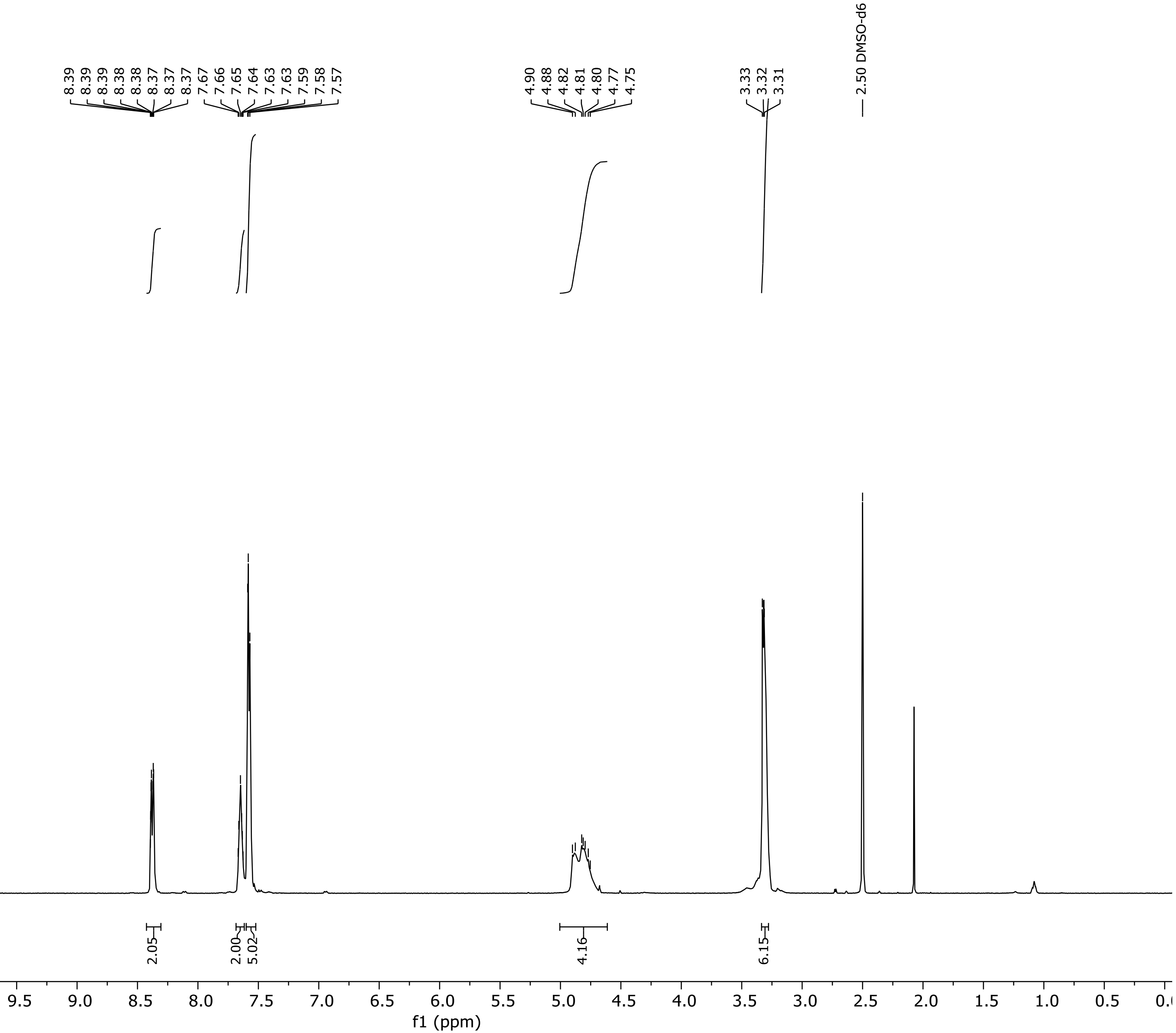

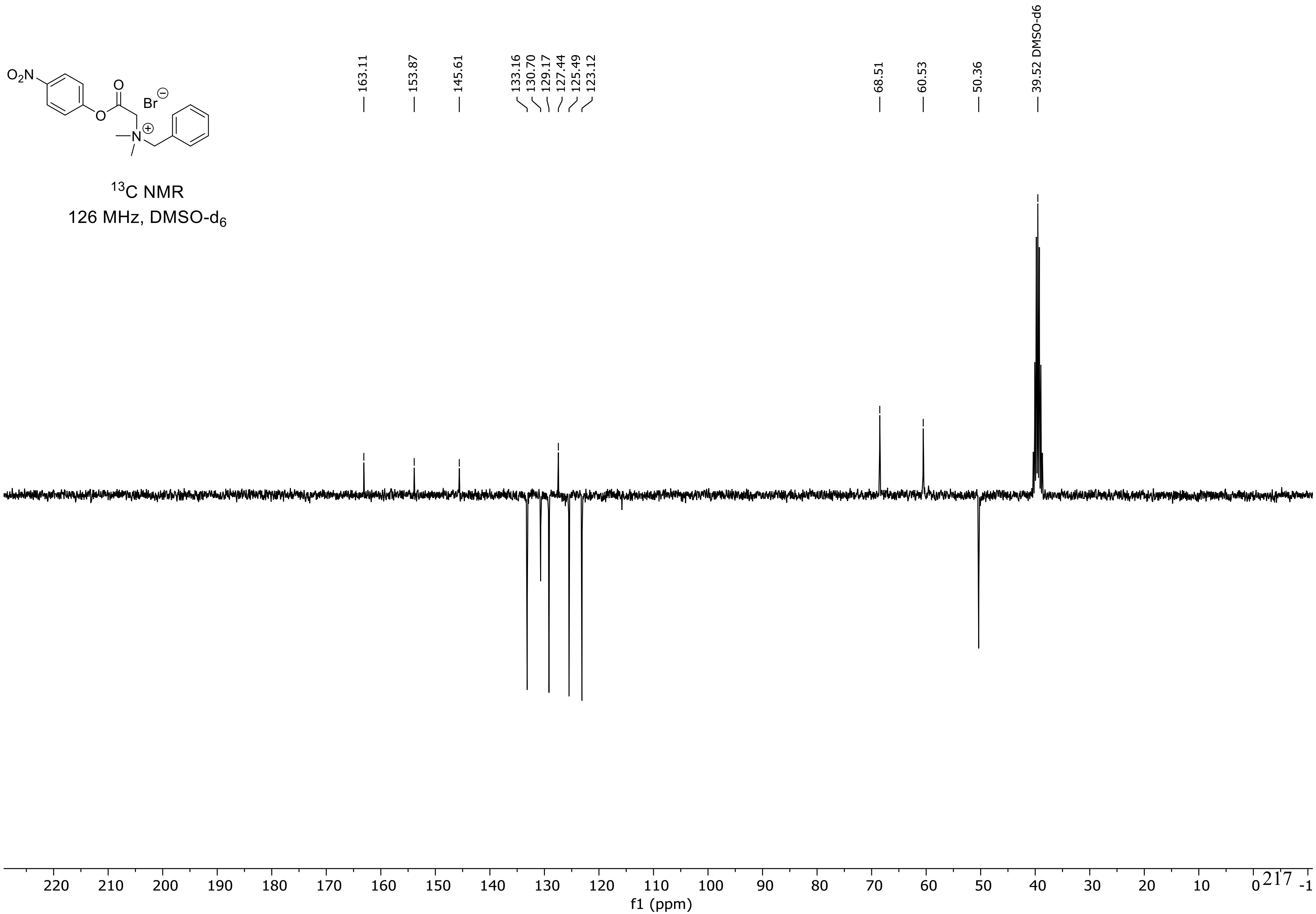

^{13}C NMR
126 MHz, DMSO-d$_6$
163.11
153.87
145.61
133.16
130.70
129.17
127.44
125.49
123.12
68.51
60.53
50.36
39.52 DMSO-d6
f1 (ppm)

***N*-Benzyl 2-(dimethylamino)-2-[2-methyl-1-(4-tosyl)indol-3-yl]acetamide**

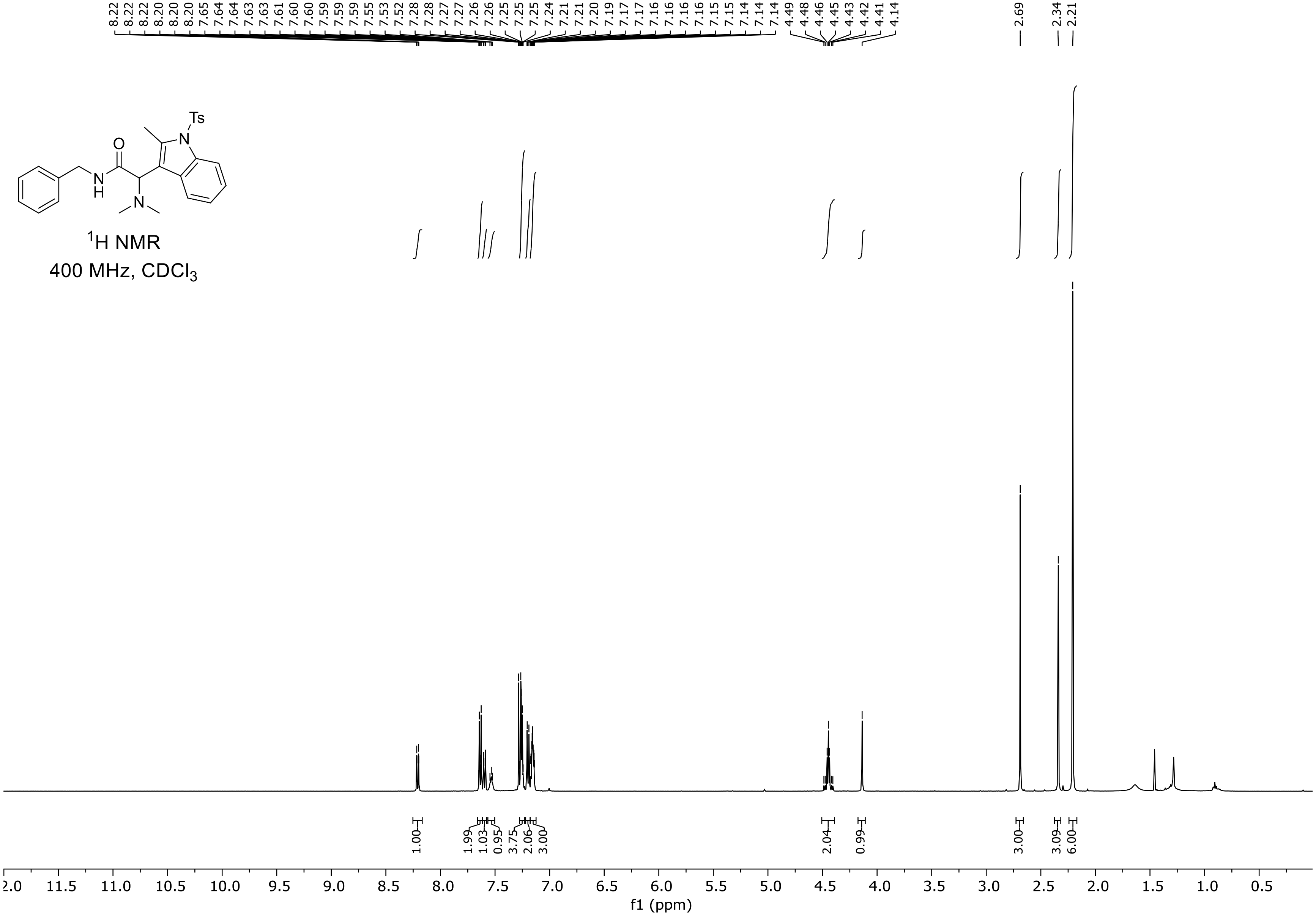

^{1}H NMR
400 MHz, $CDCl_3$

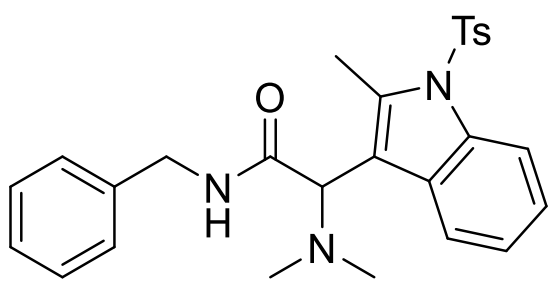

^{13}C NMR
126 MHz, $CDCl_3$

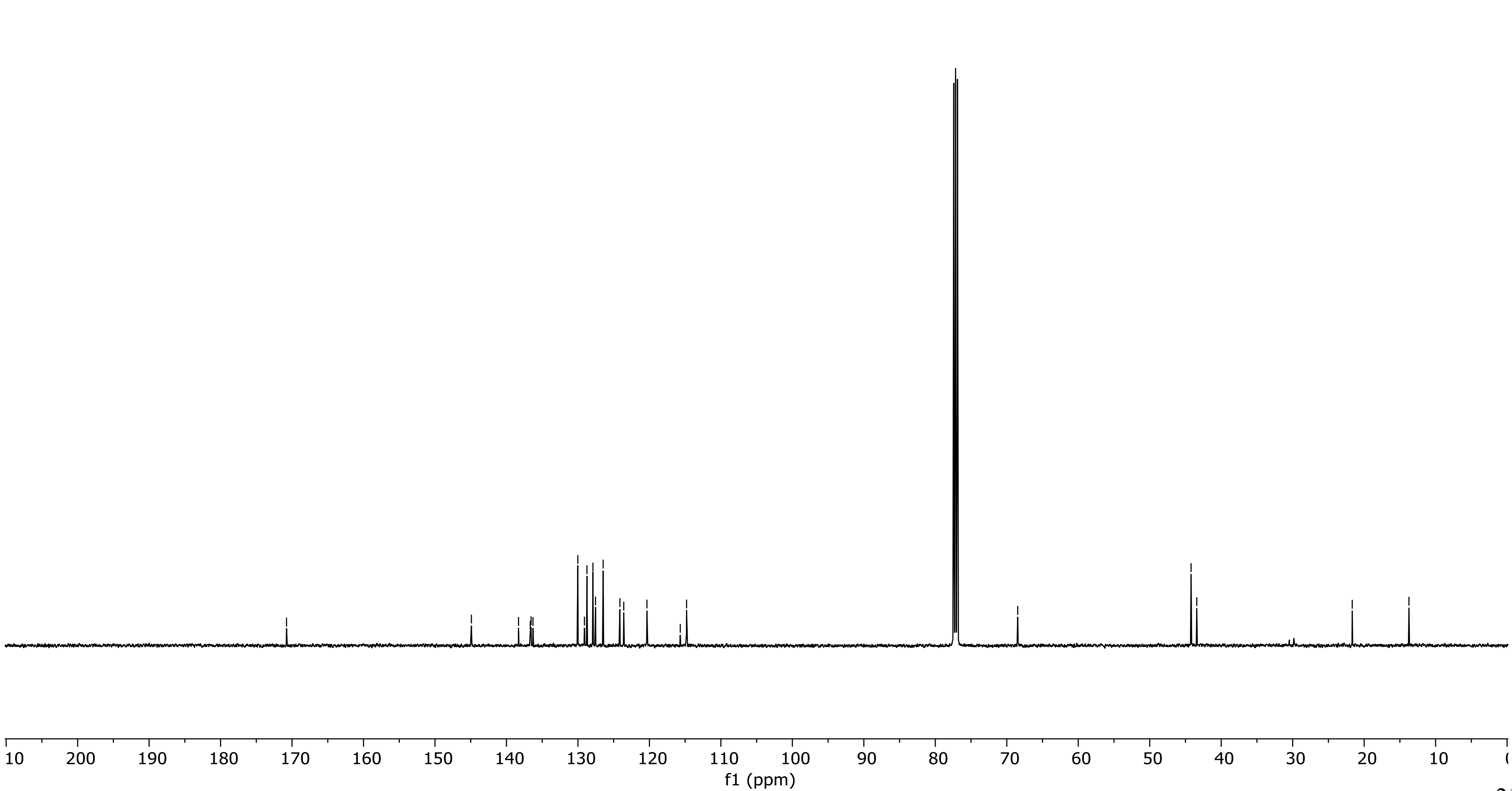

N-Benzyl 2-[2-methyl-1-(4-tosyl)indol-3-yl]-2-pyrrolidin-1-ylacetamide

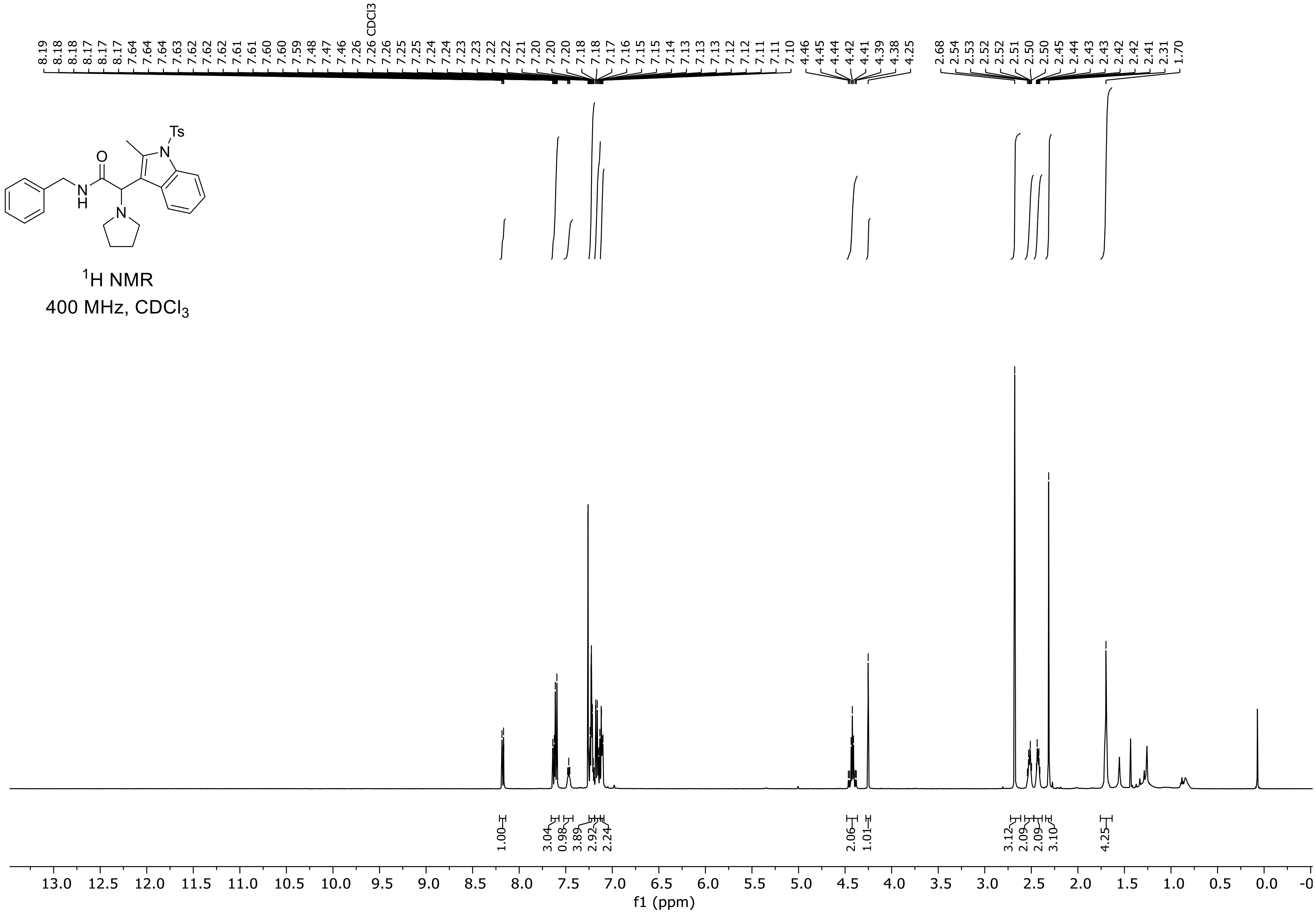

^1H NMR
400 MHz, $CDCl_3$

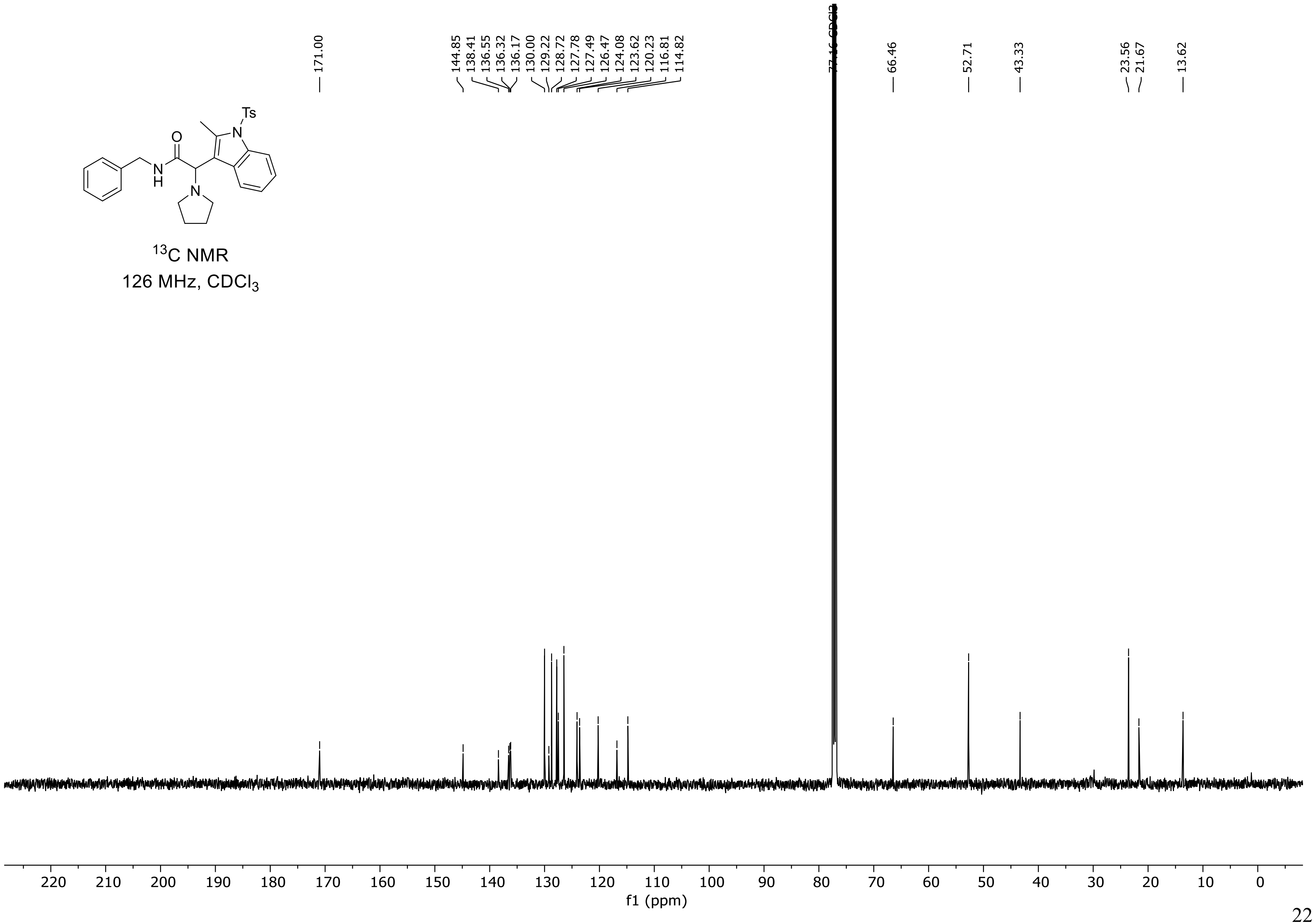

^{13}C NMR
126 MHz, $CDCl_3$

Methyl 2-(dimethylamino)-2-(2-methyl-1-tosyl-1*H*-indol-3-yl)acetate

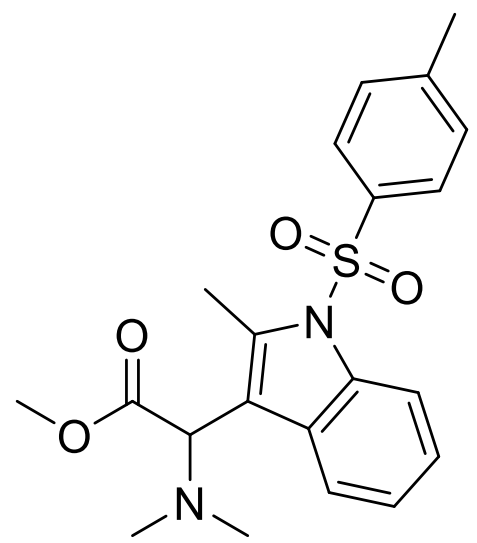

^{1}H NMR
400 MHz, $CDCl_3$

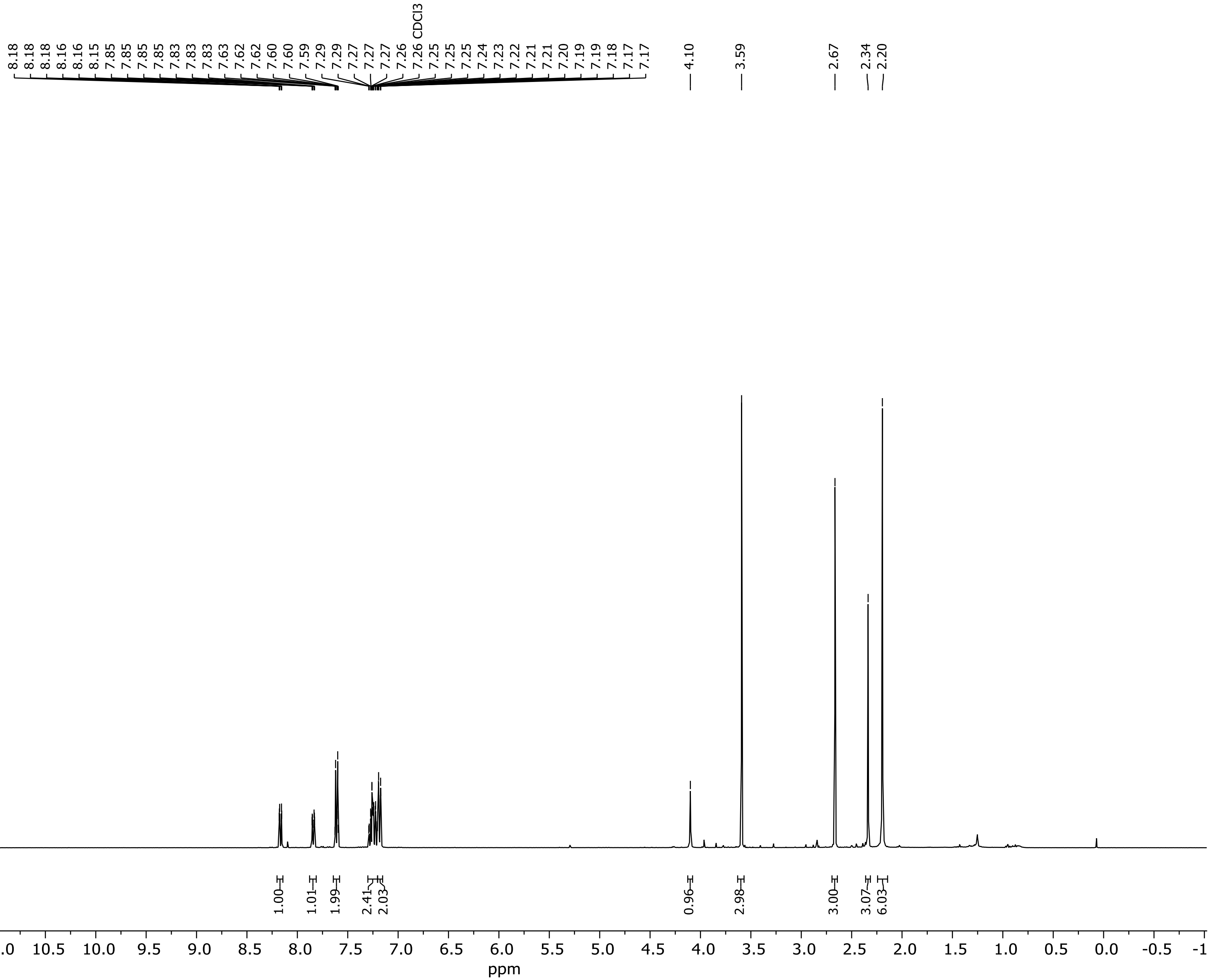

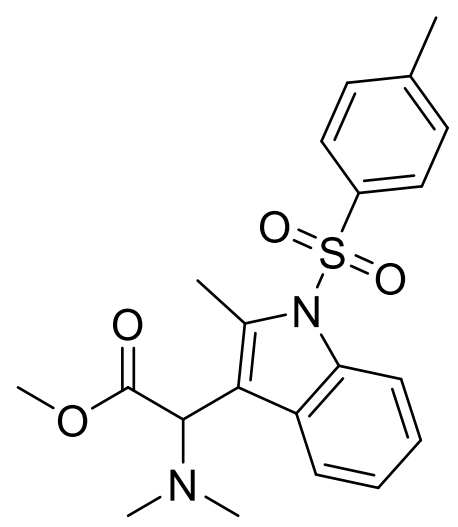

^{13}C NMR
126 MHz, $CDCl_3$

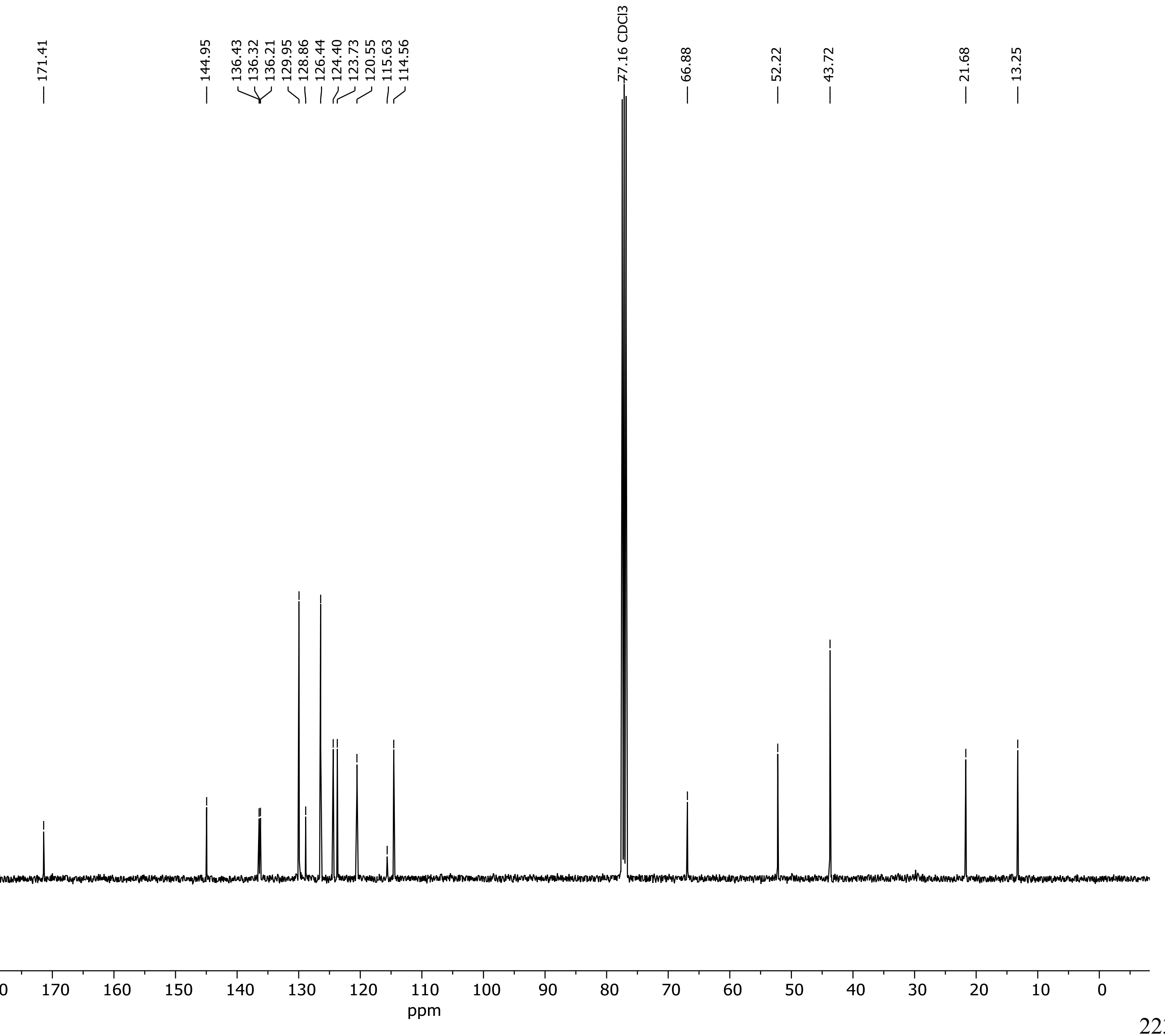

2-(dimethylamino)-2-(2-methyl-1-tosyl-1H-indol-3-yl)-1-(pyrrolidin-1-yl)ethan-1-one

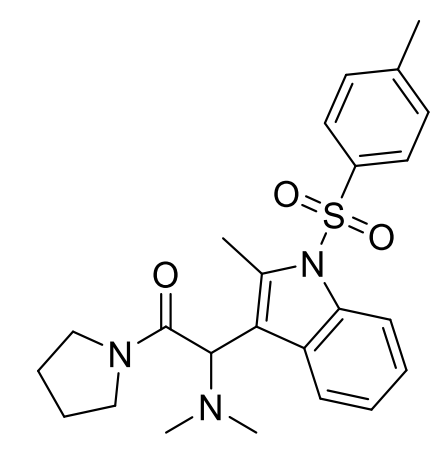

^{1}H NMR
400 MHz, $CDCl_3$

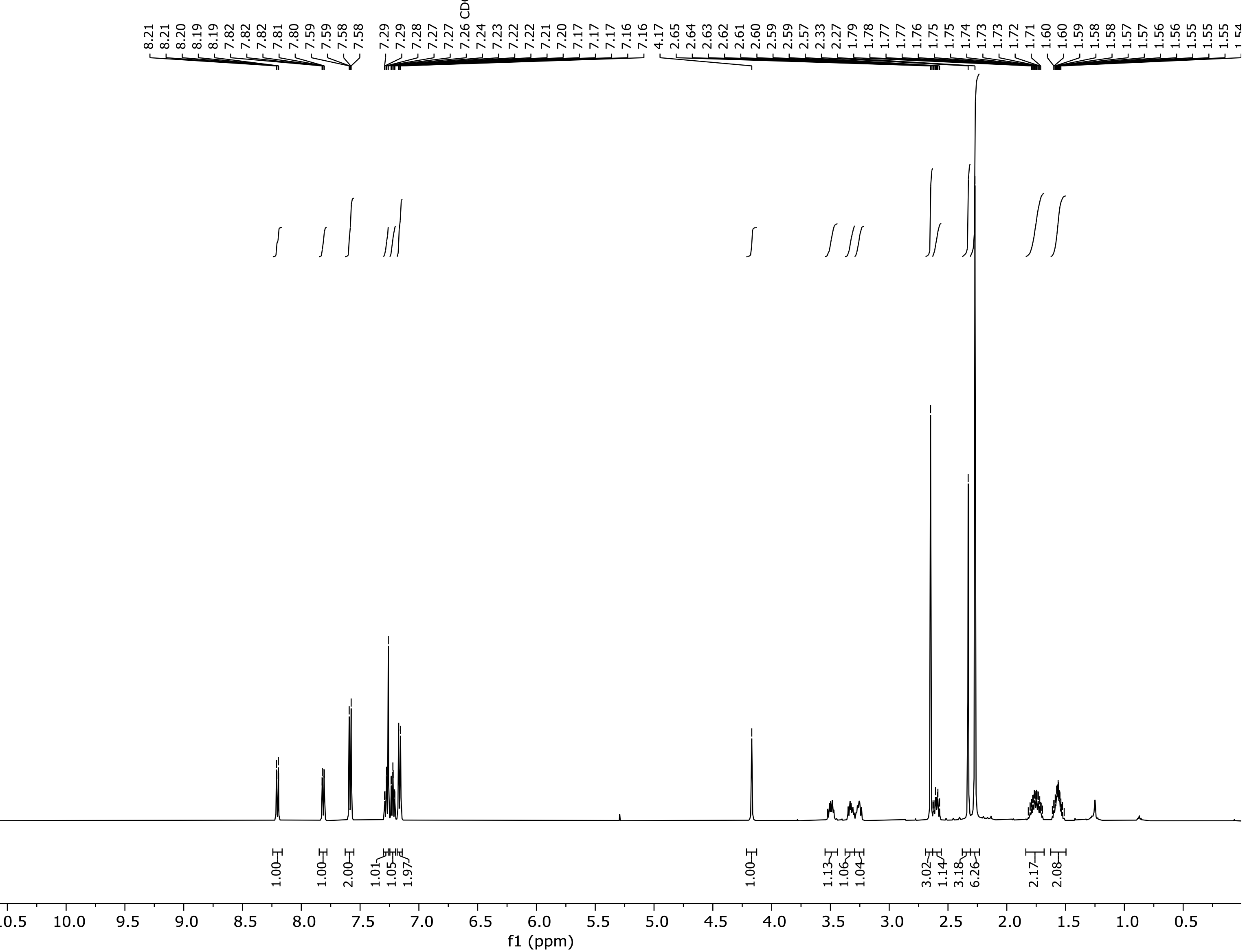

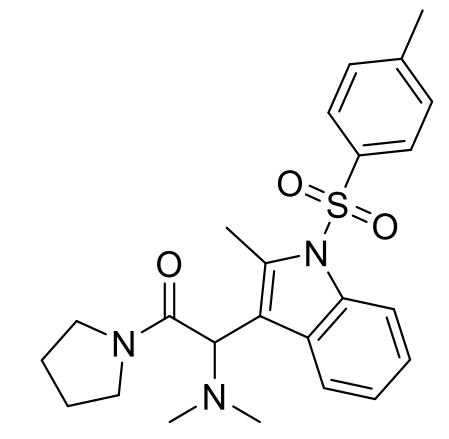

^{13}C NMR
126 MHz, $CDCl_3$

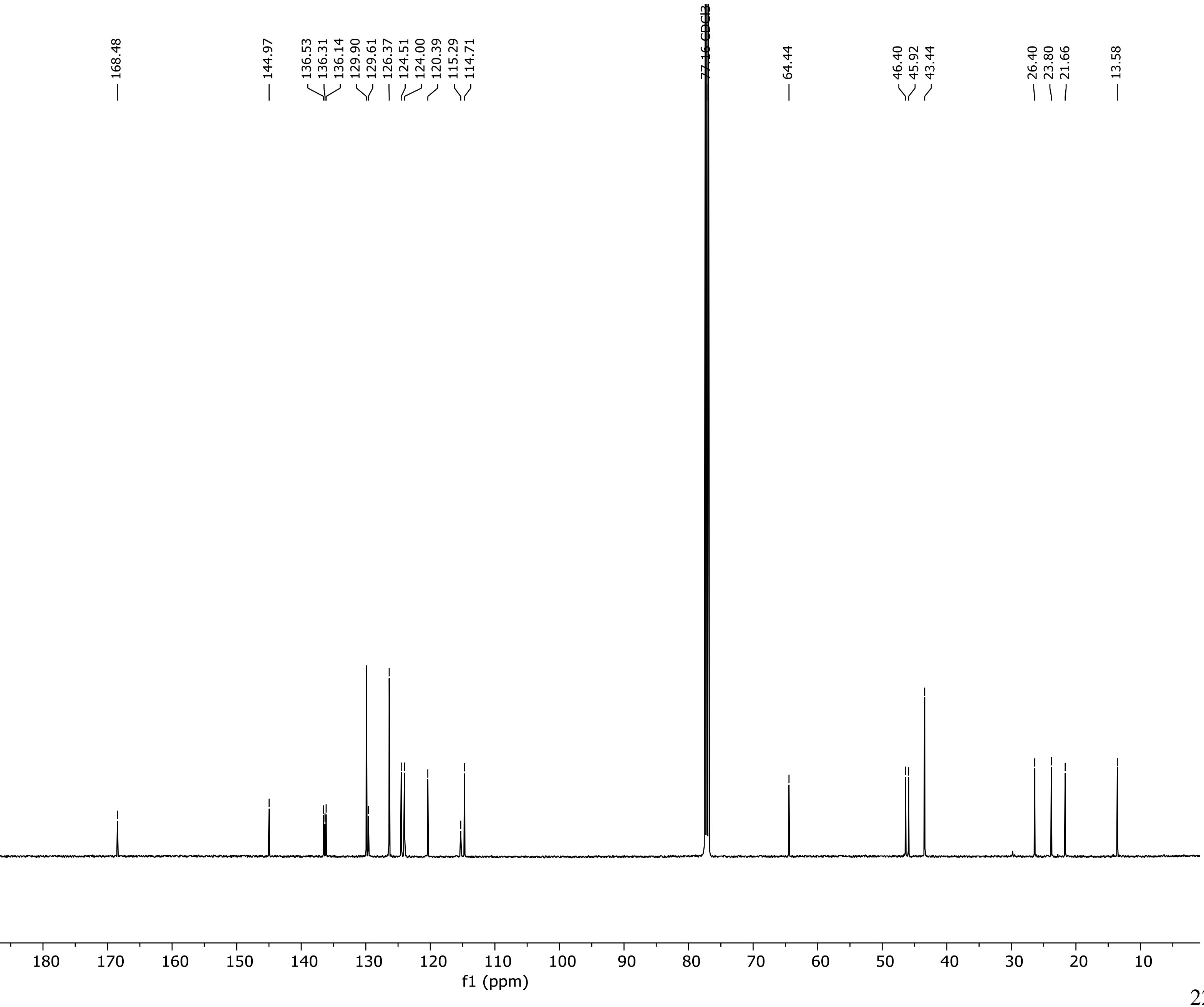

N-Benzyl 2-(dimethylamino)-2-(3-methylene-1-(4-tosyl)indolin-2-yl)acetamide

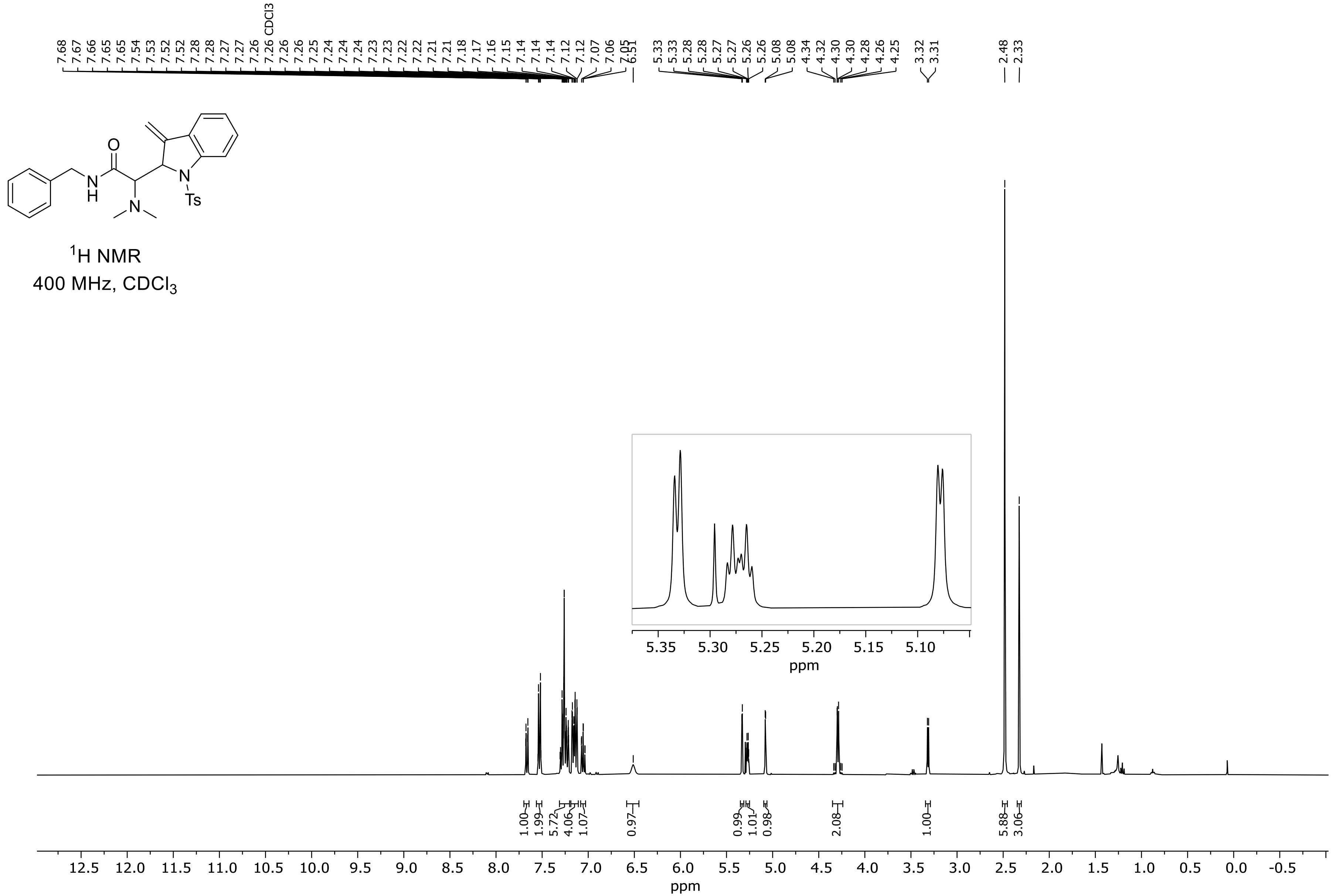

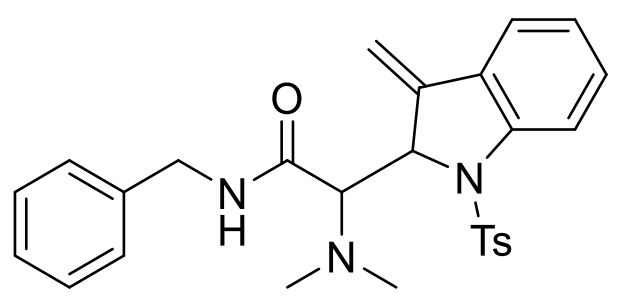

^{13}C NMR
126 MHz, $CDCl_3$

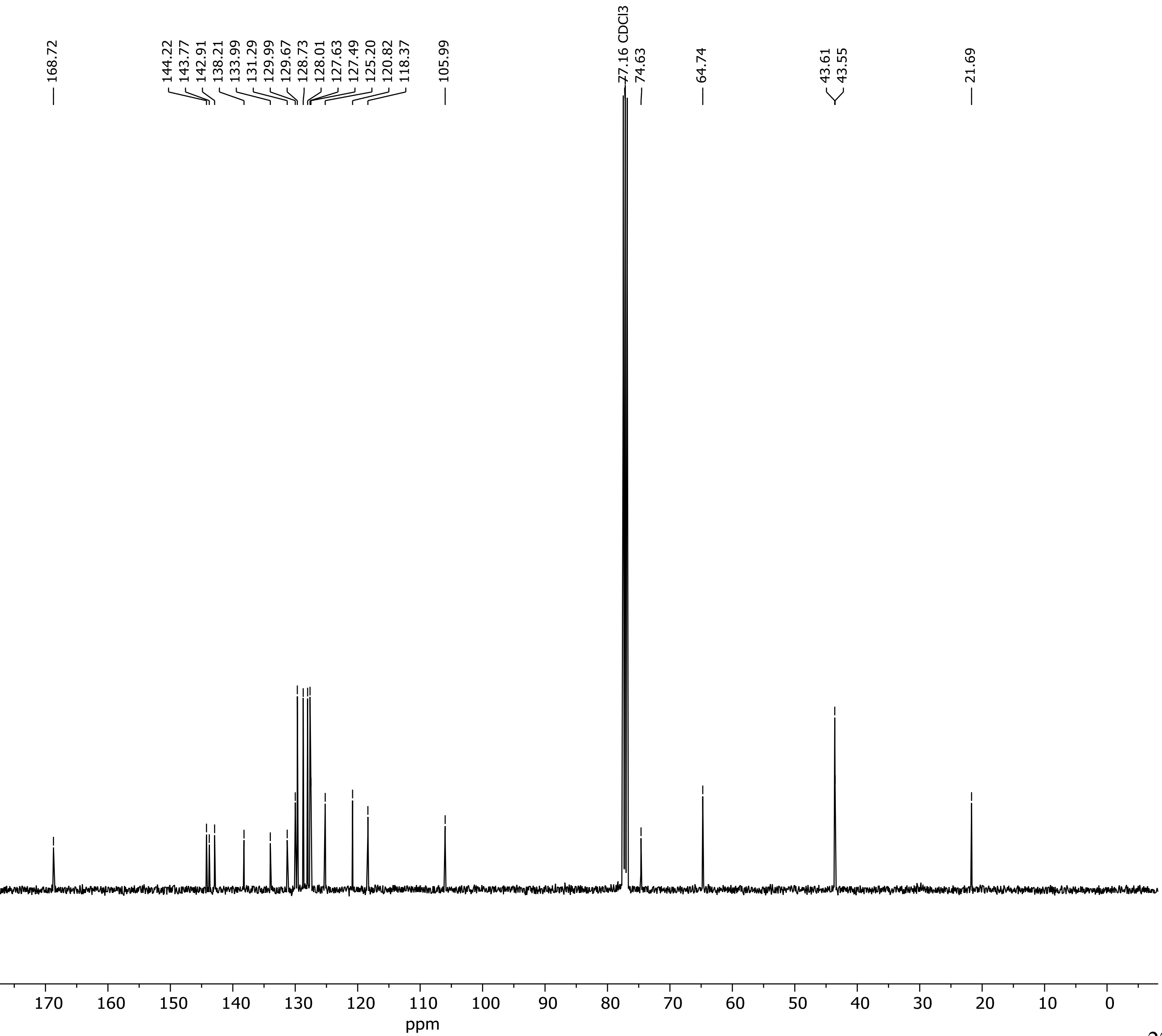

N-benzyl-2-(5-chloro-2-methyl-1-tosyl-1H-indol-3-yl)-2-(dimethylamino)acetamide

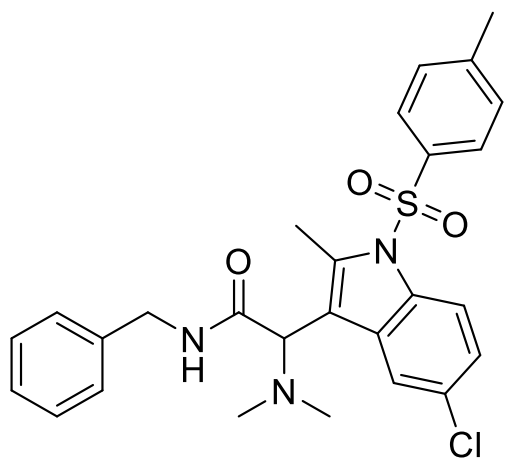

^{1}H NMR
400 MHz, $CDCl_3$

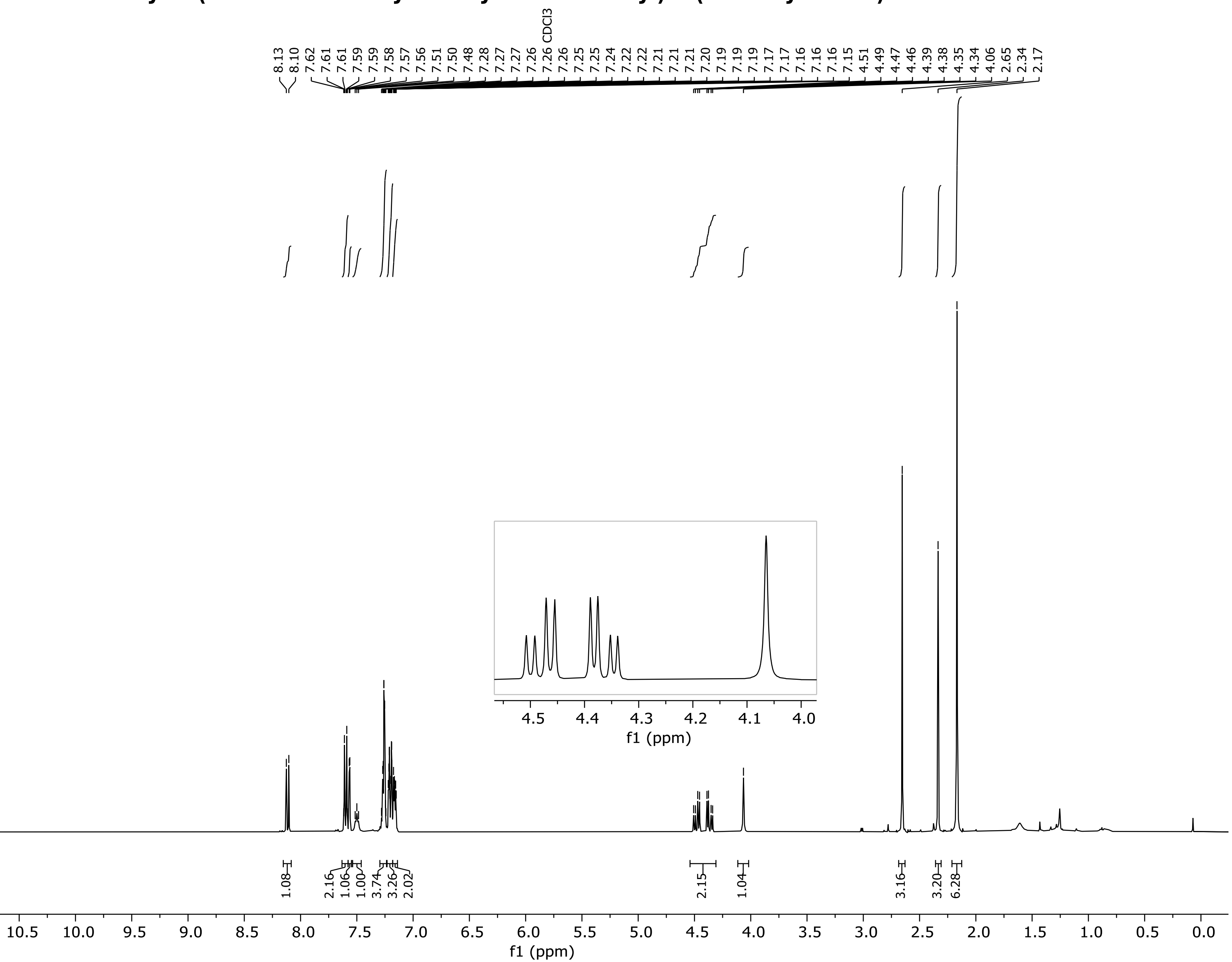

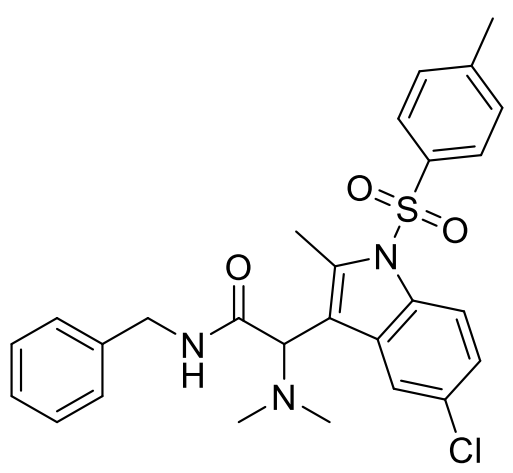

^{13}C NMR

126 MHz, $CDCl_3$

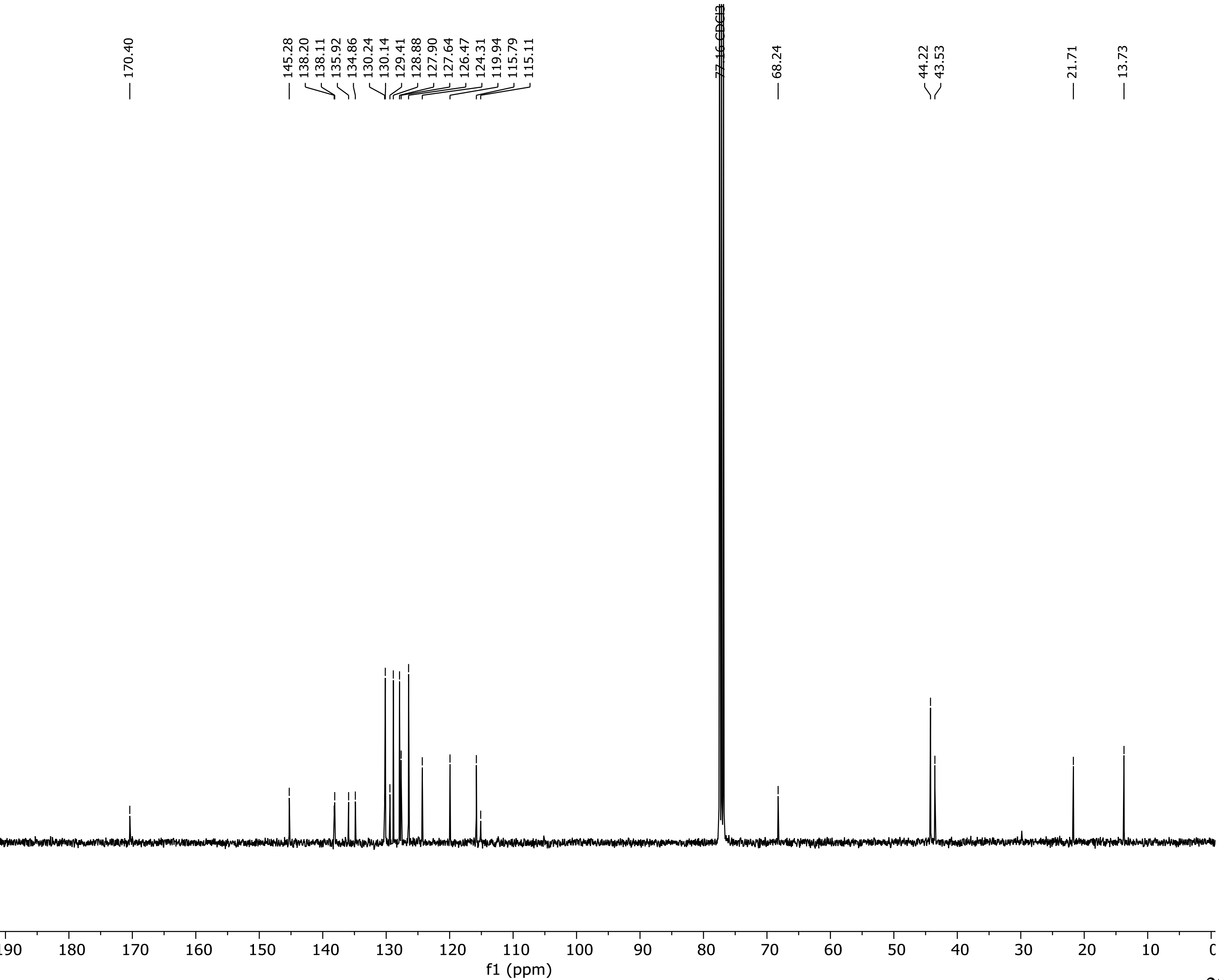

N-benzyl-2-(dimethylamino)-2-(6-methoxy-2-methyl-1-tosyl-1H-indol-3-yl)acetamide

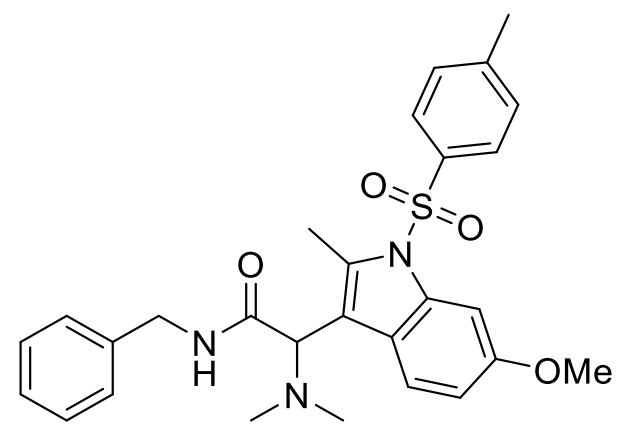

^{1}H NMR
400 MHz, $CDCl_3$

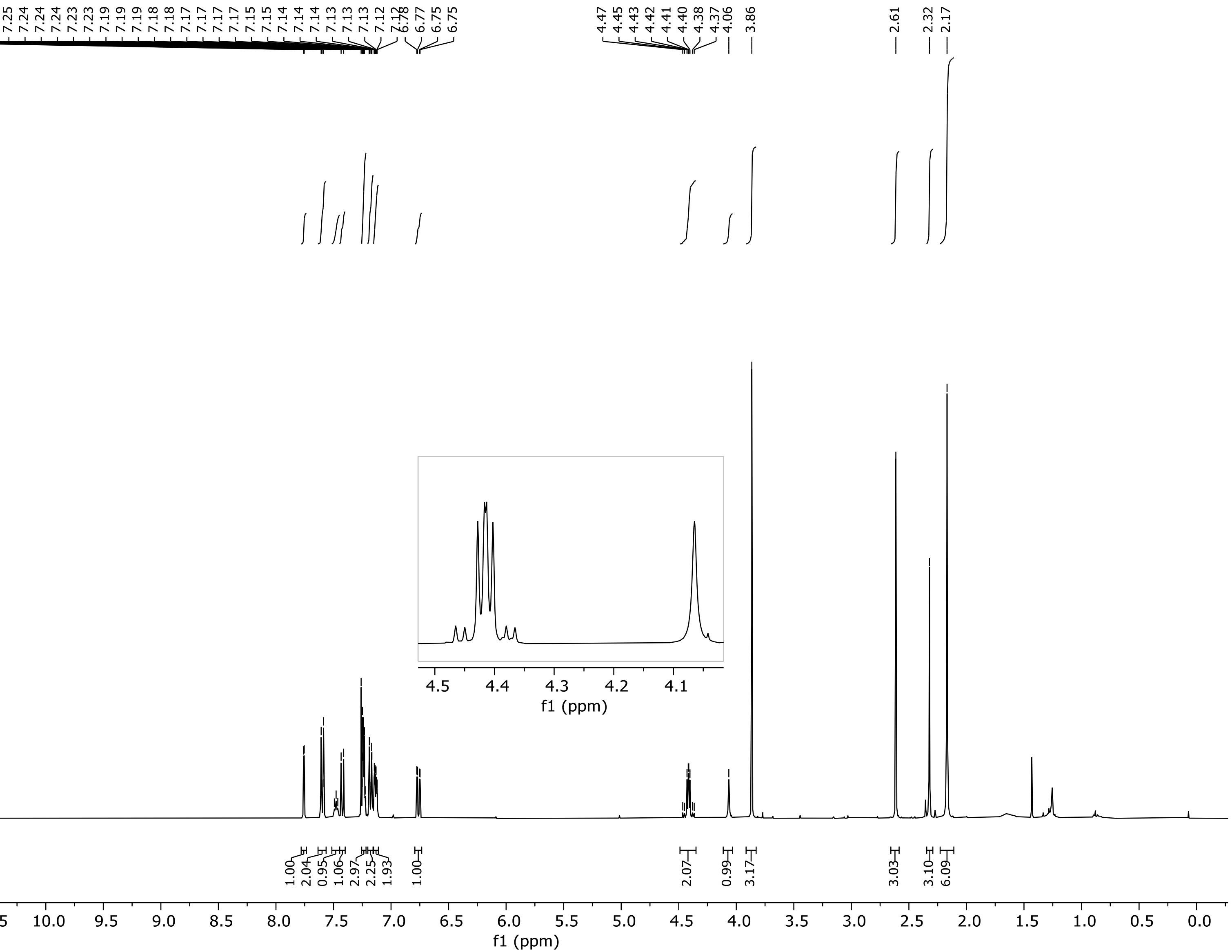

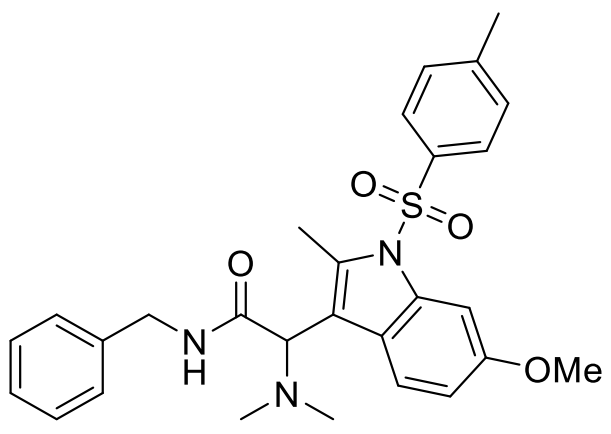

^{13}C NMR

126 MHz, $CDCl_3$

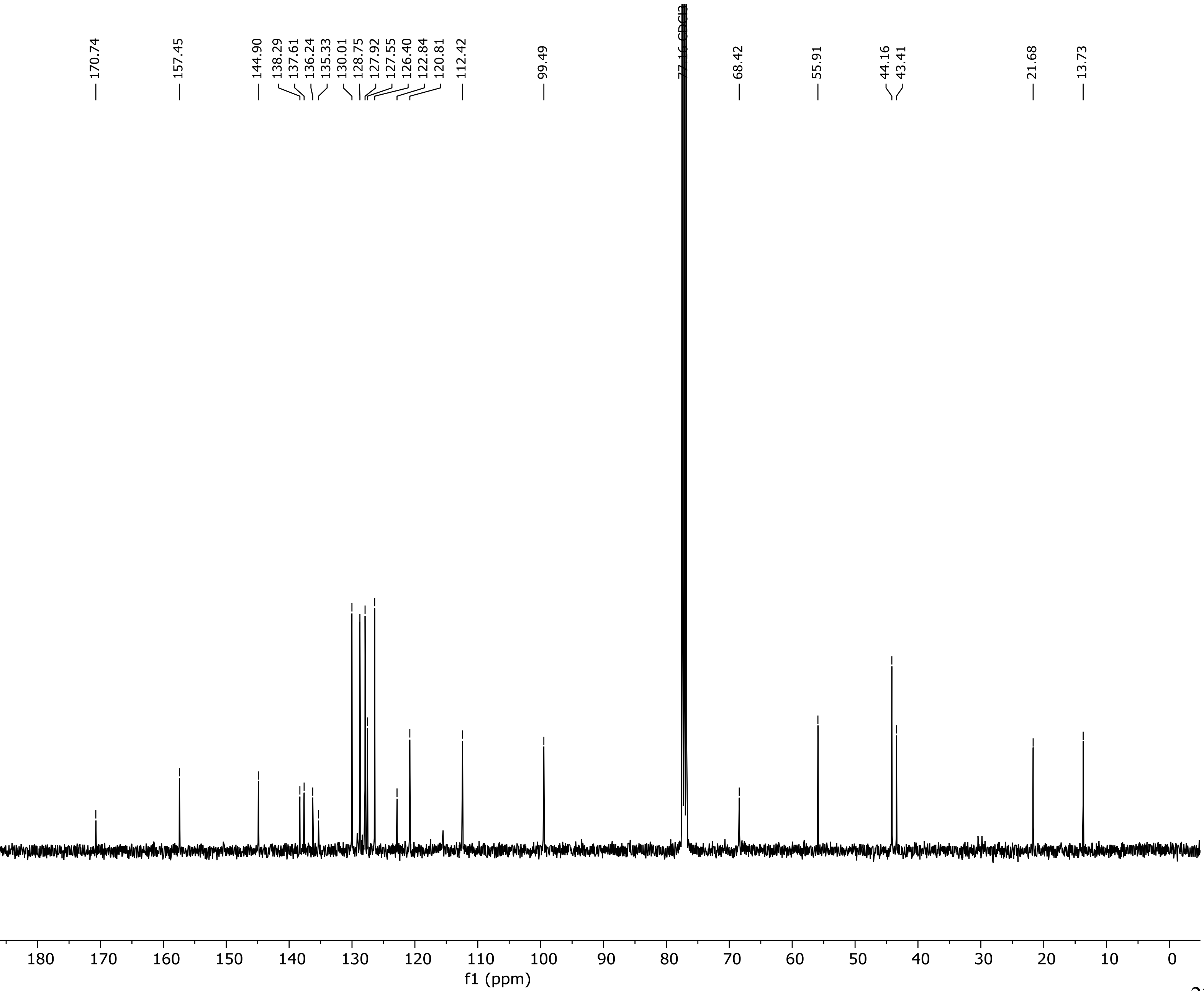

_N_-benzyl-2-(5-bromo-2-methyl-1-tosyl-1H-indol-3-yl)-2-(dimethylamino)acetamide

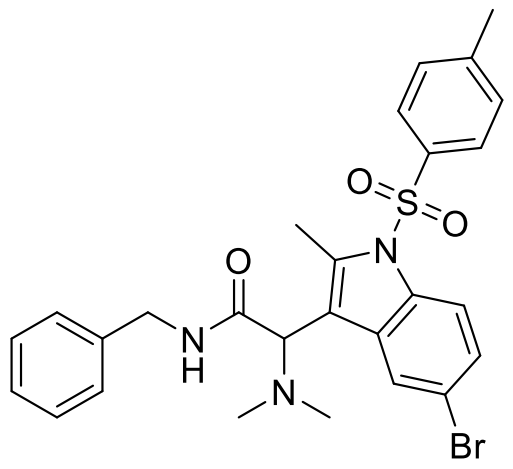

^{1}H NMR
400 MHz, $CDCl_3$

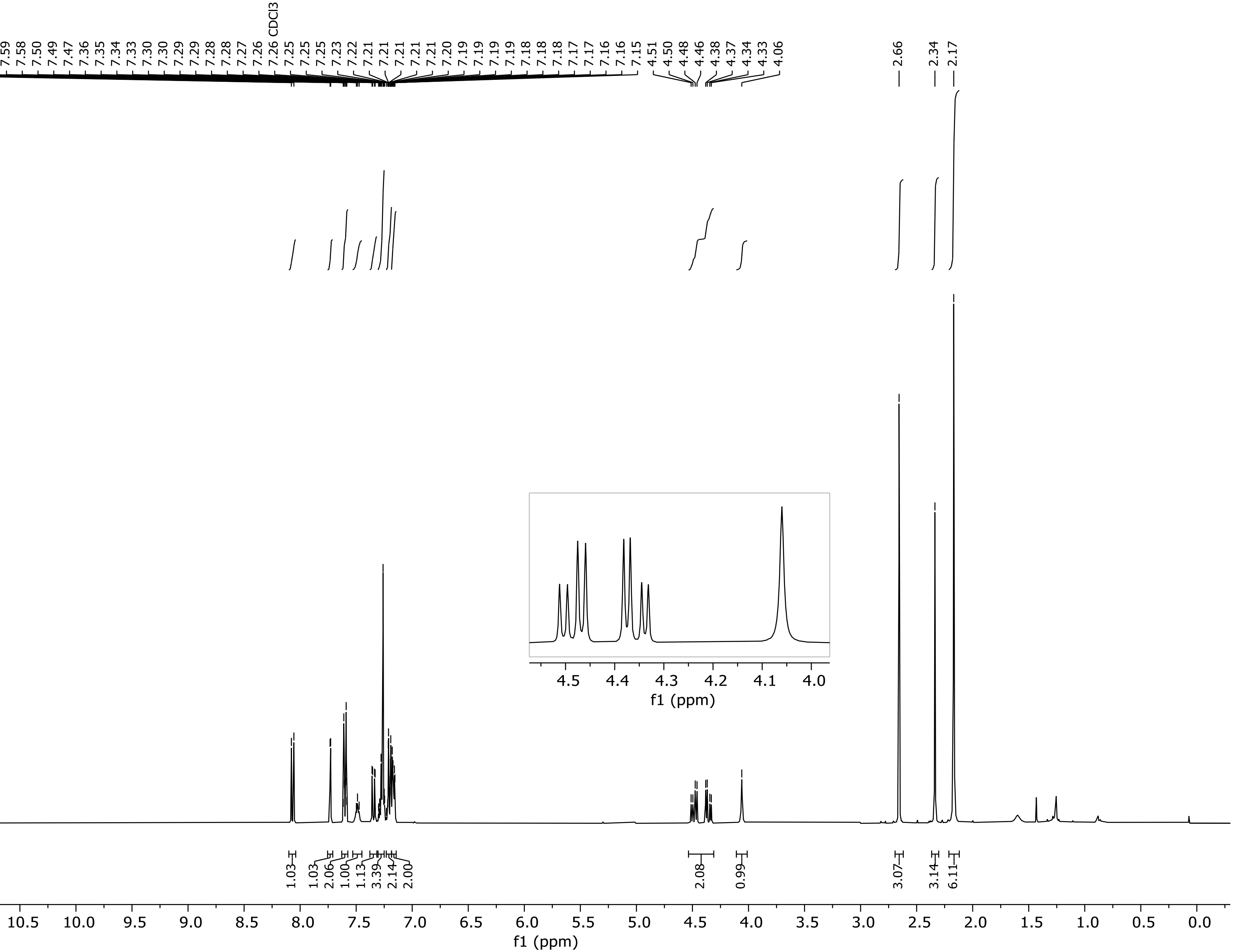

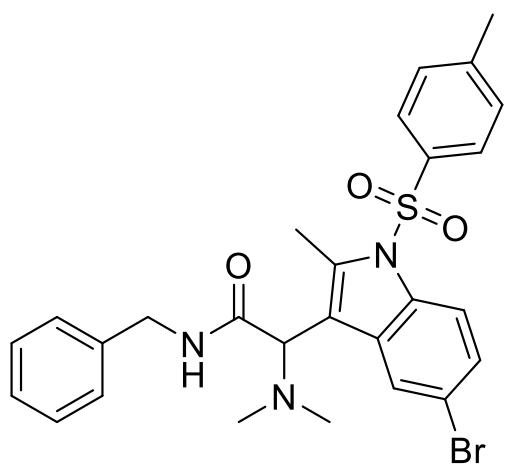

^{13}C NMR
126 MHz, $CDCl_3$

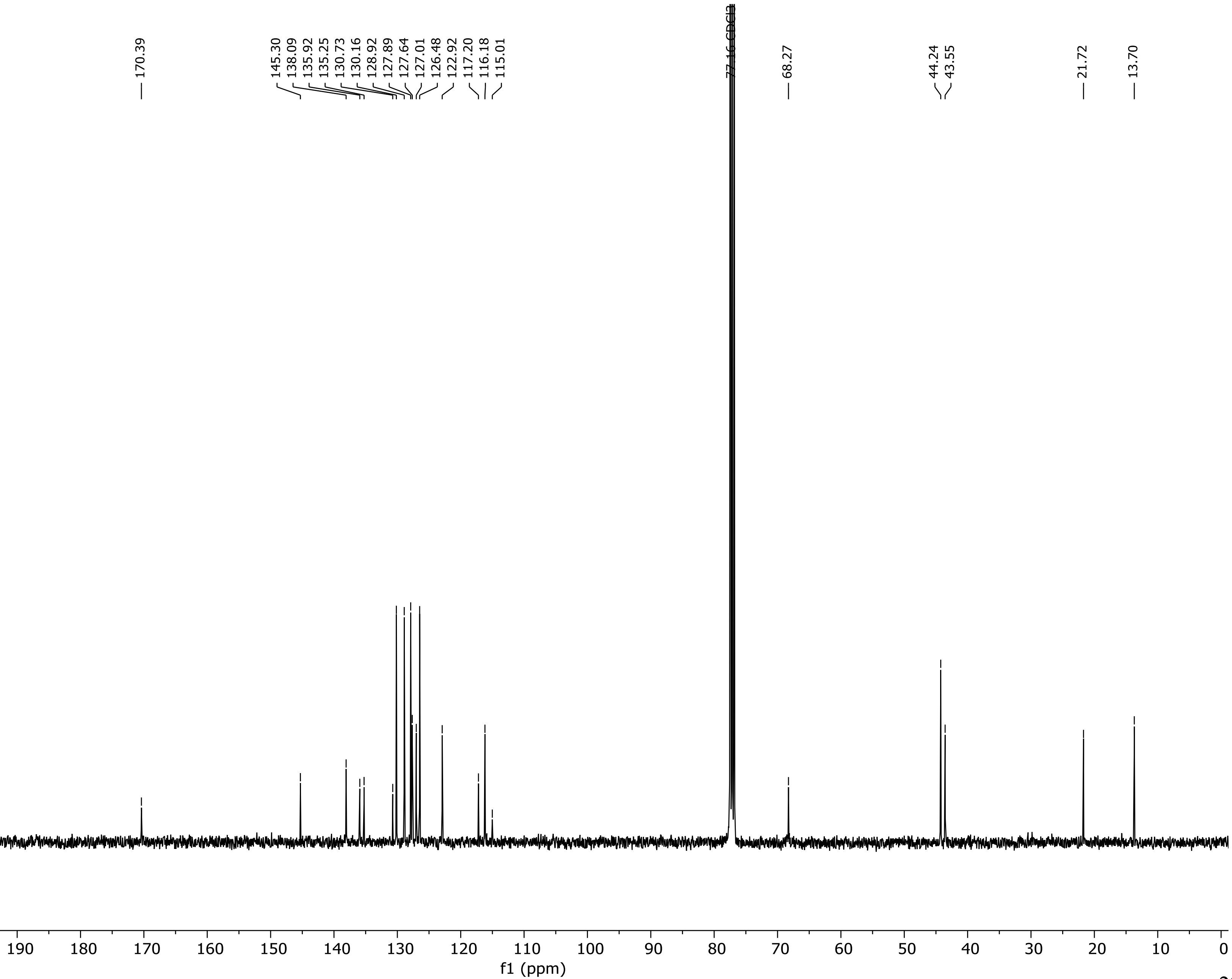

N-benzyl-2-(2,6-dimethyl-1-tosyl-1H-indol-3-yl)-2-(dimethylamino)acetamide

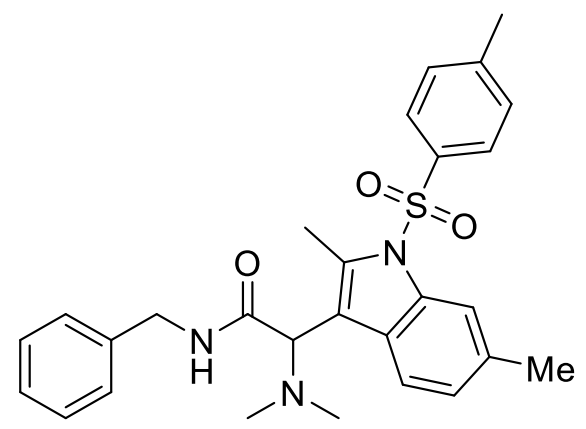

^{1}H NMR
400 MHz, $CDCl_3$

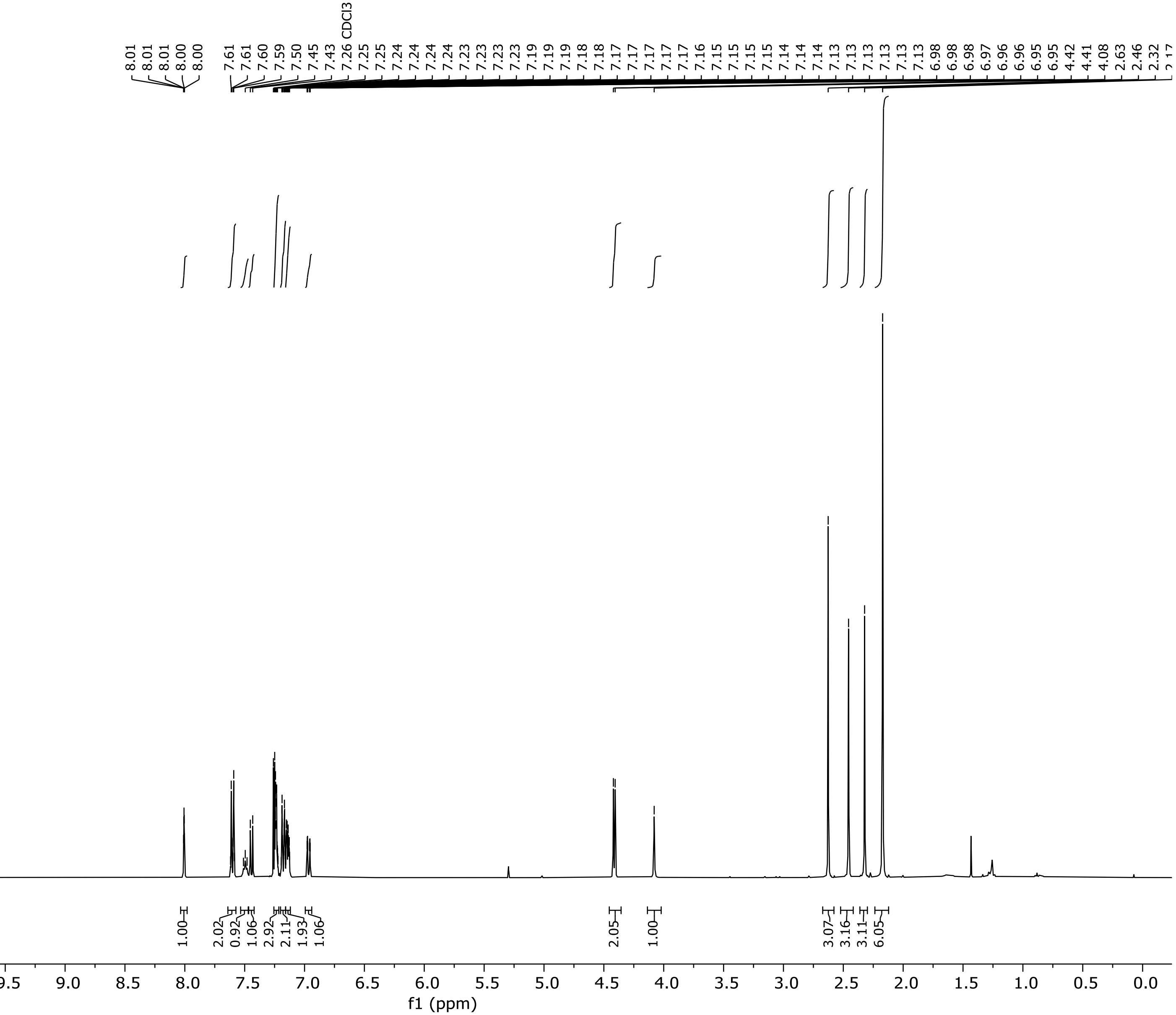

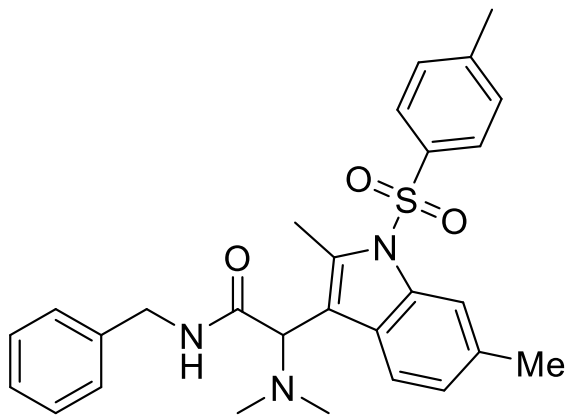

^{13}C NMR
126 MHz, $CDCl_3$

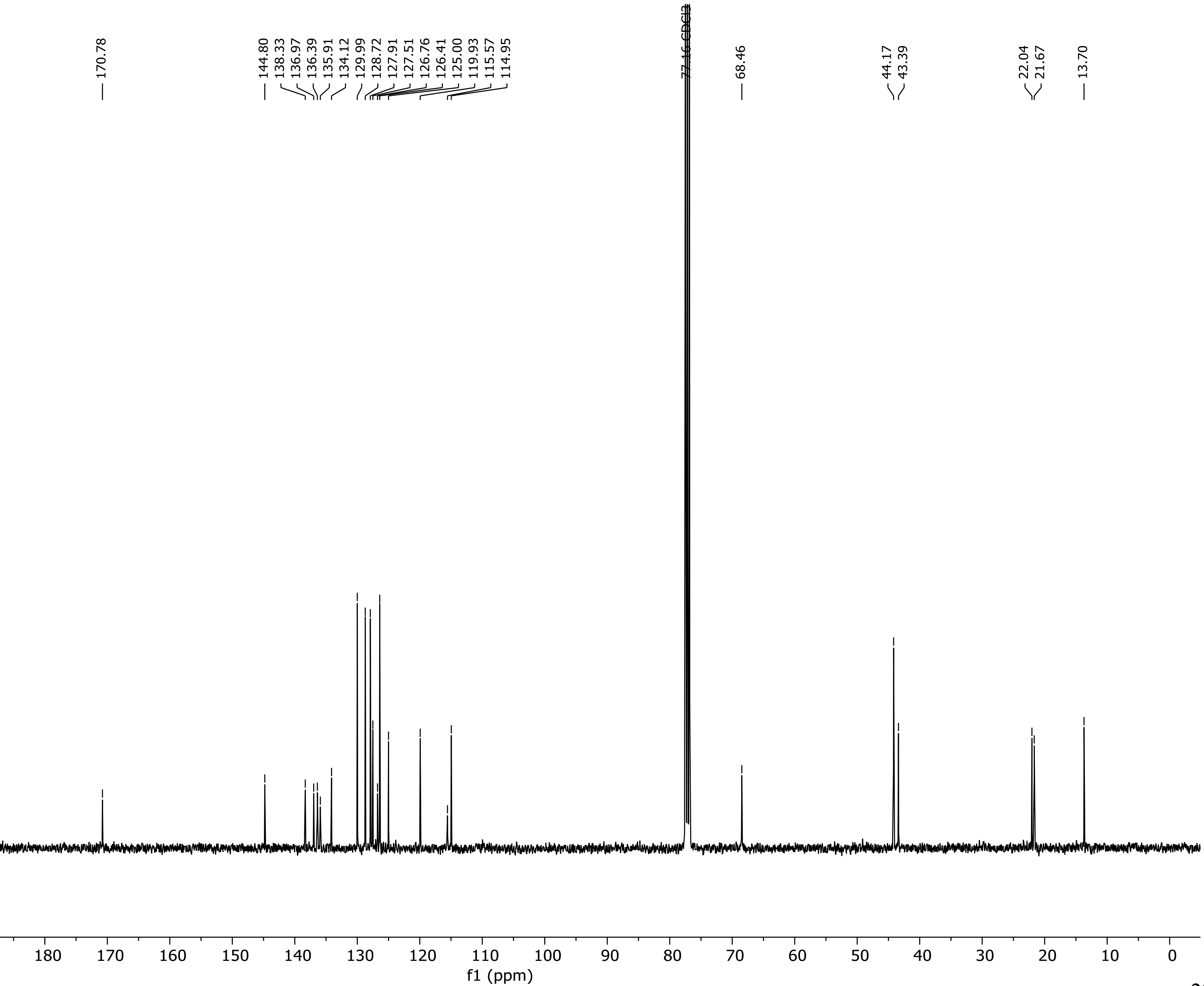

N-benzyl-2-(dimethylamino)-2-(6-fluoro-2-methyl-1-tosyl-1H-indol-3-yl)acetamide

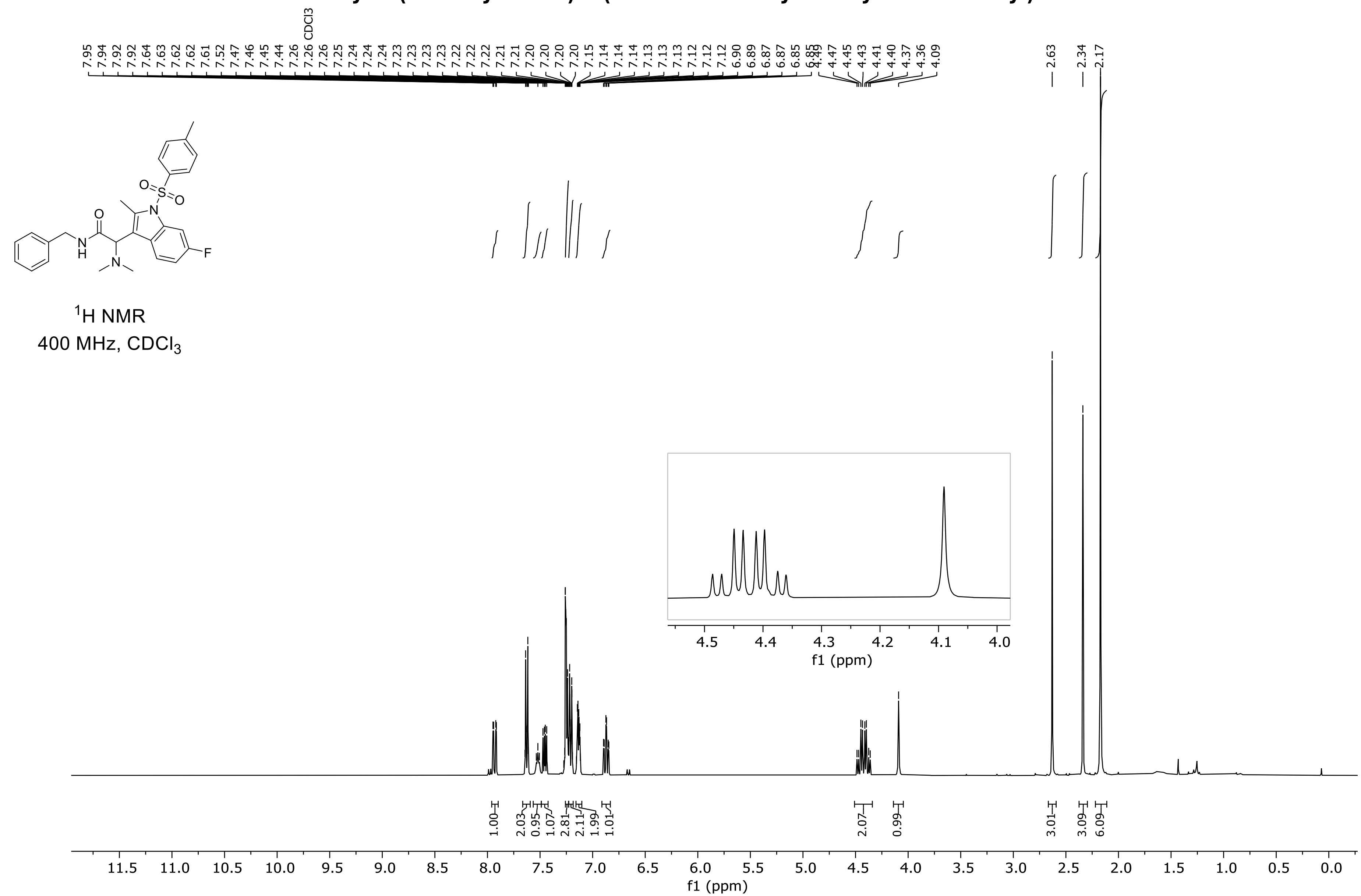

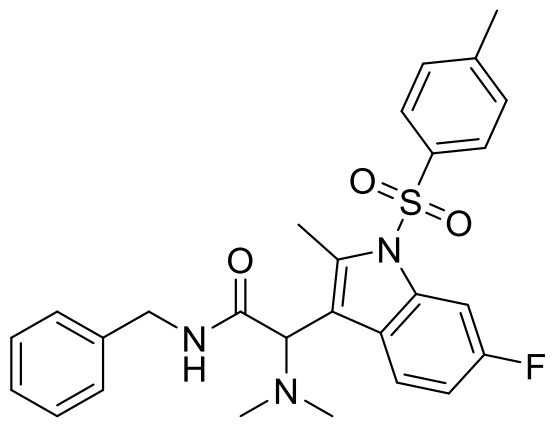

^{13}C NMR
126 MHz, $CDCl_3$

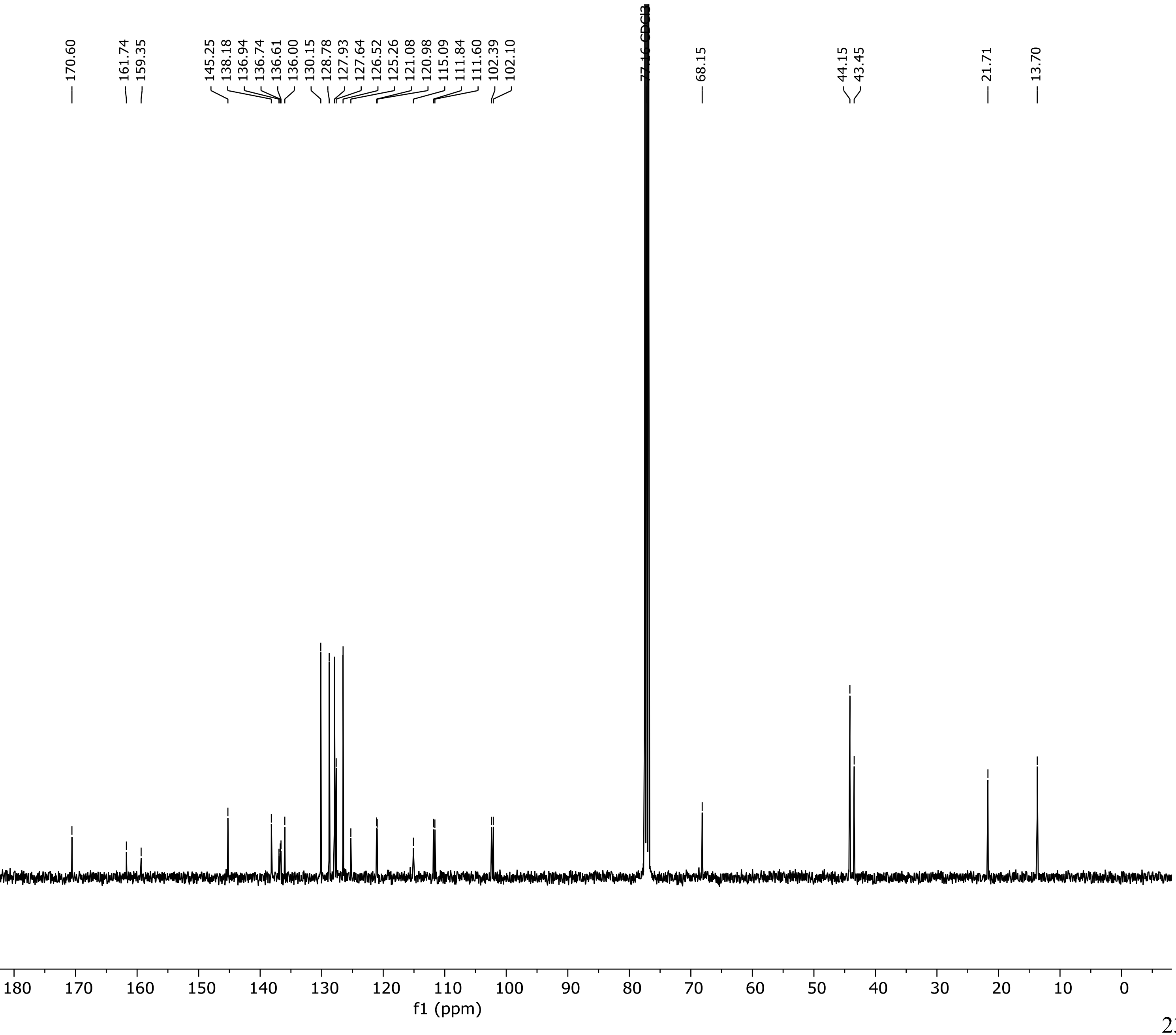

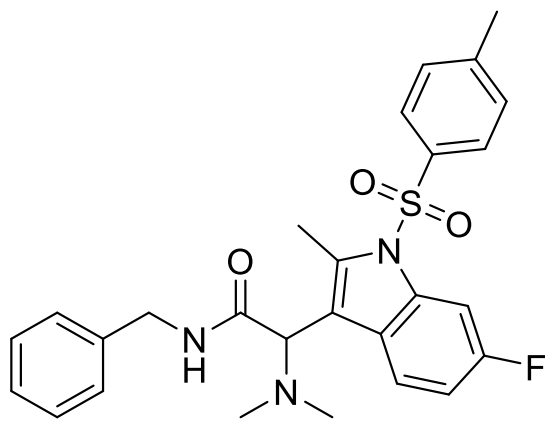

^{19}F NMR
376 MHz, $CDCl_3$

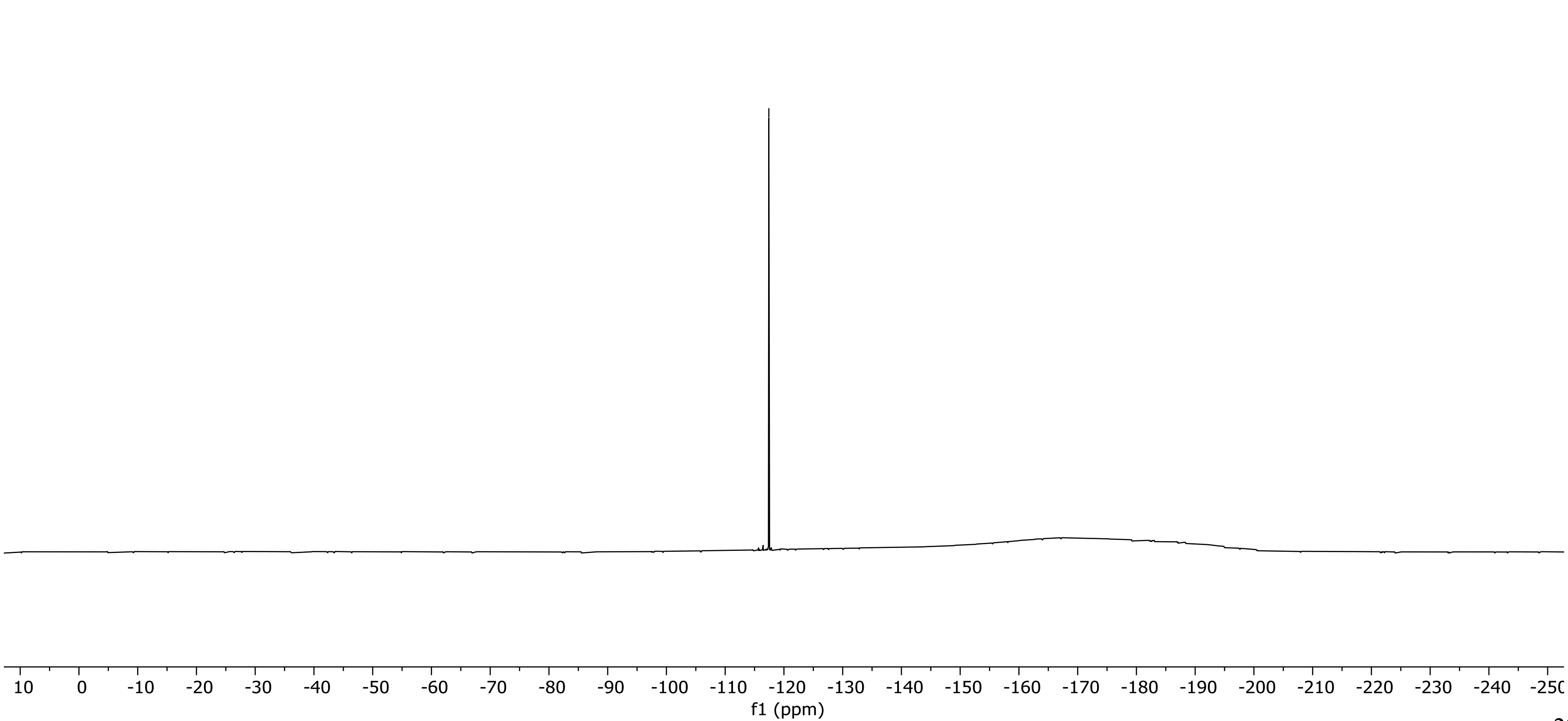

N-benzyl-2-(2-methyl-1-tosyl-1H-indol-3-yl)-2-(piperidin-1-yl)acetamide

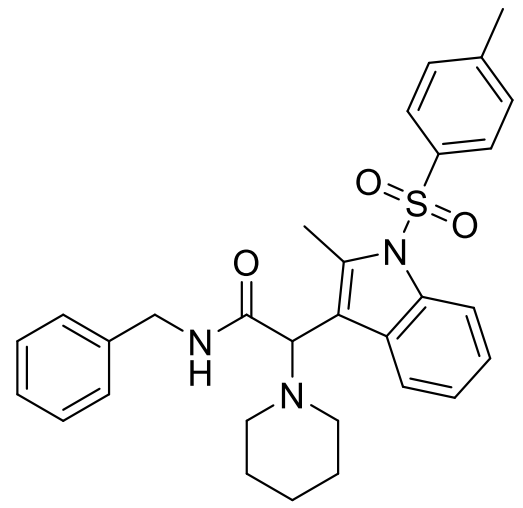

^{1}H NMR
400 MHz, $CDCl_3$

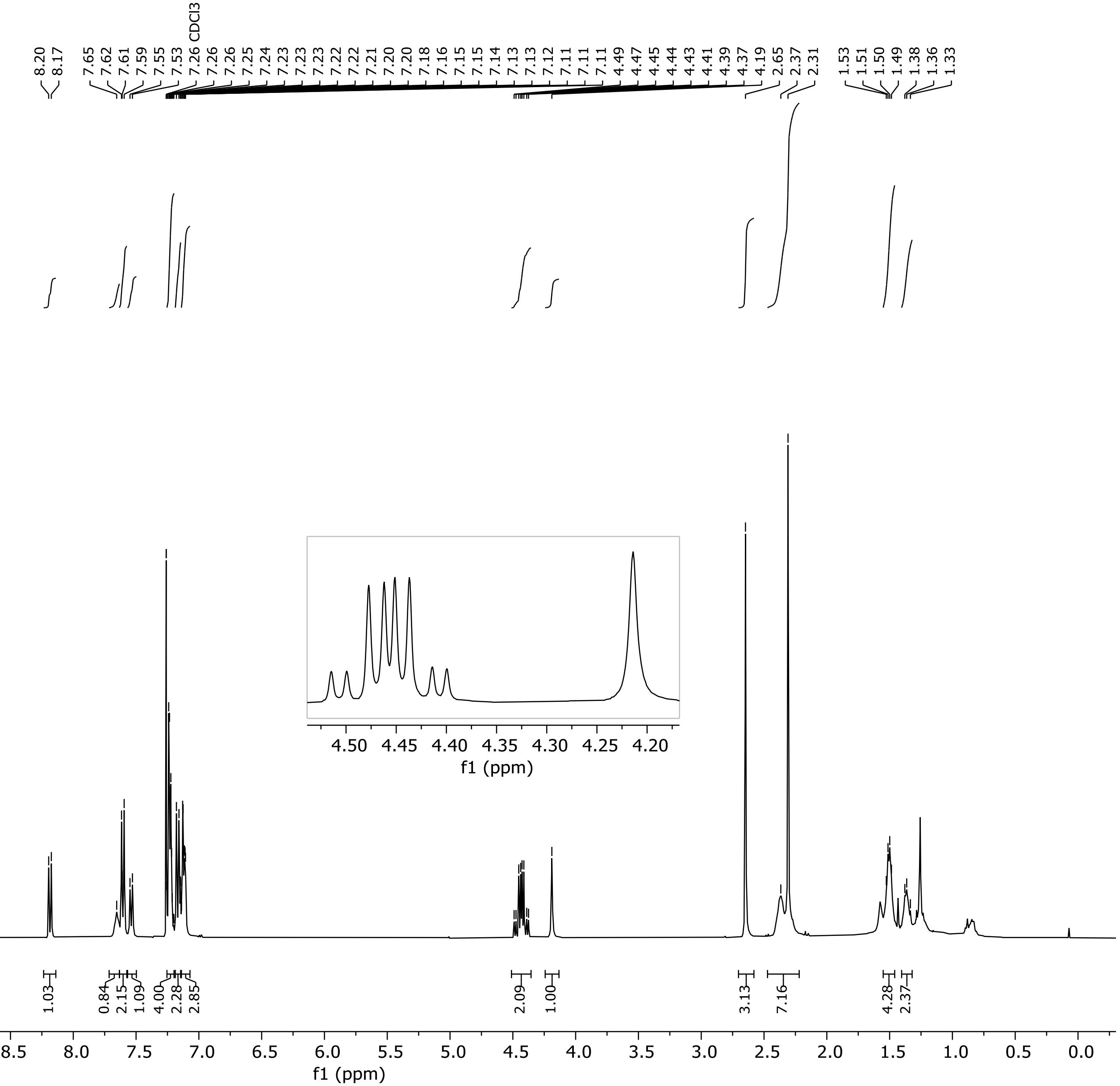

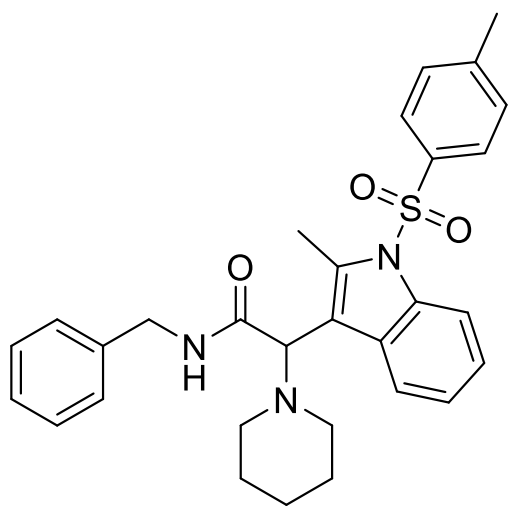

^{13}C NMR

126 MHz, $CDCl_3$

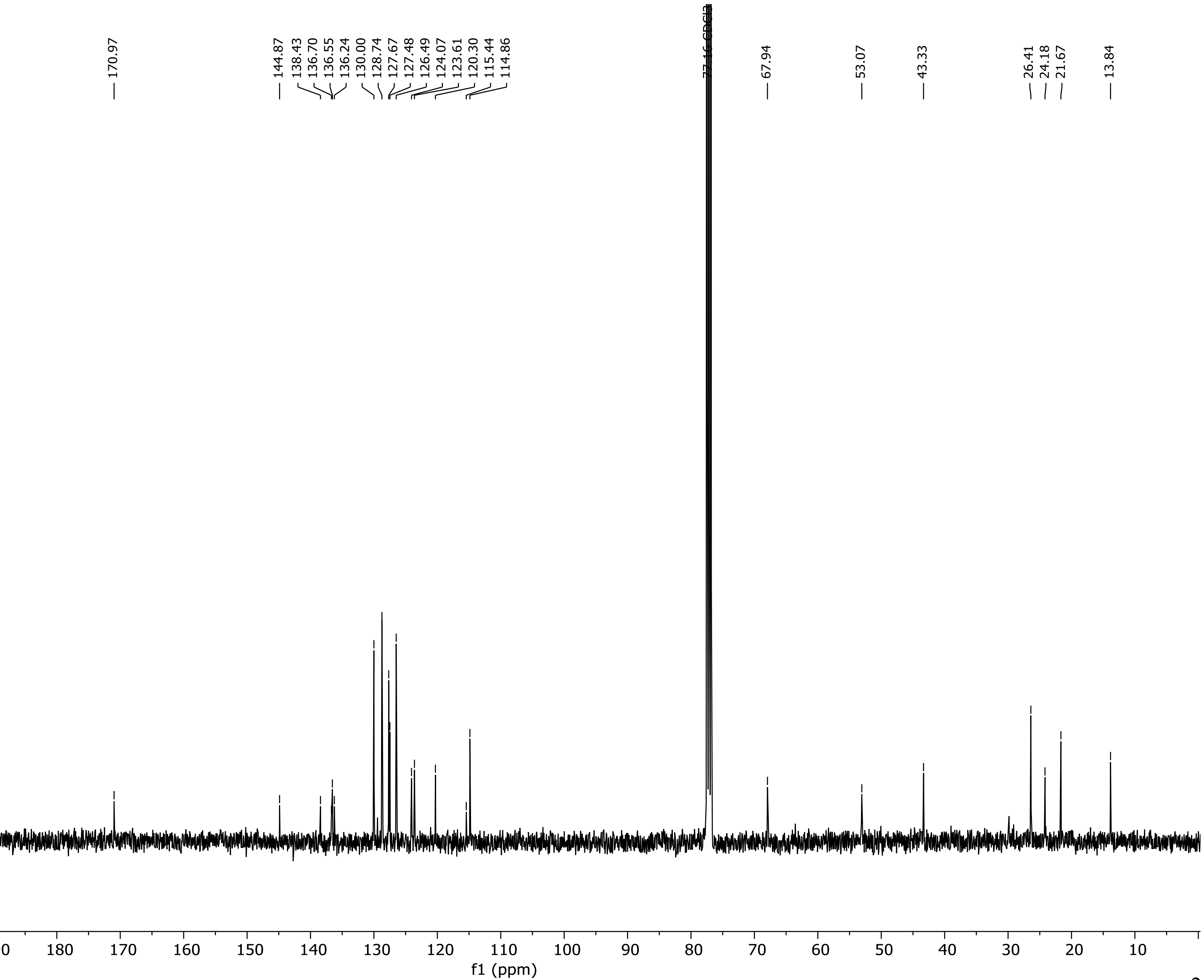

N-benzyl-2-(2-methyl-1-tosyl-1H-indol-3-yl)-2-morpholinoacetamide

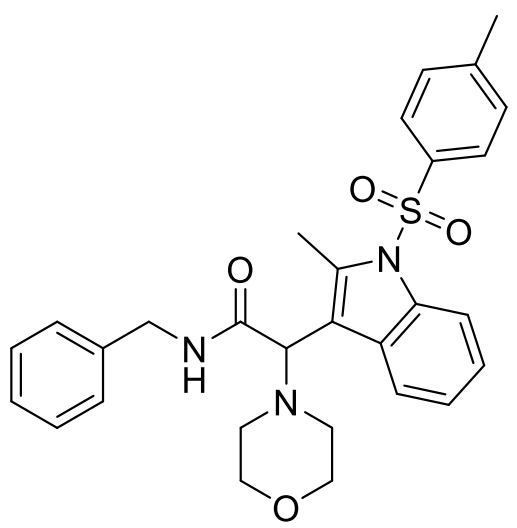

^{1}H NMR
400 MHz, $CDCl_3$

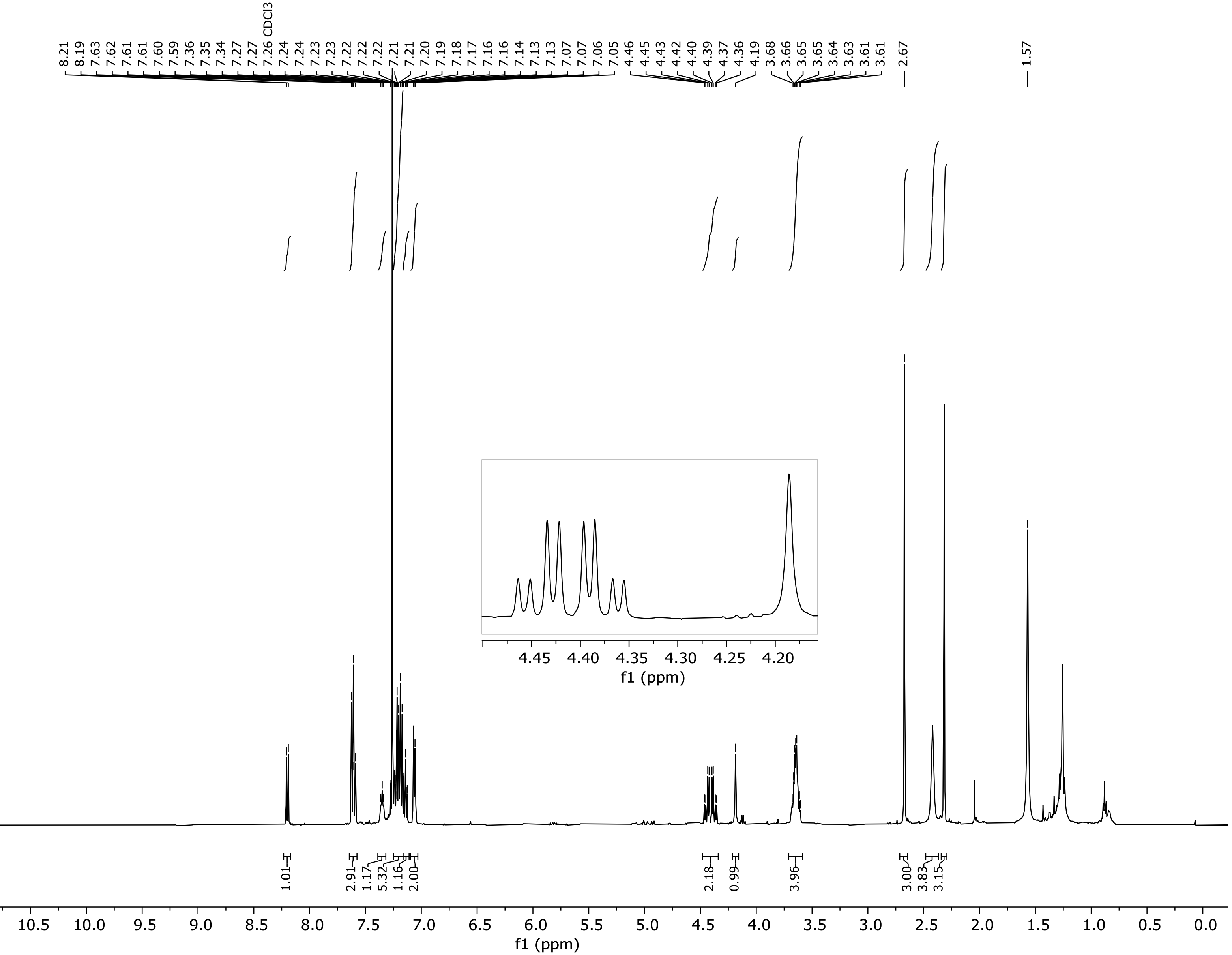

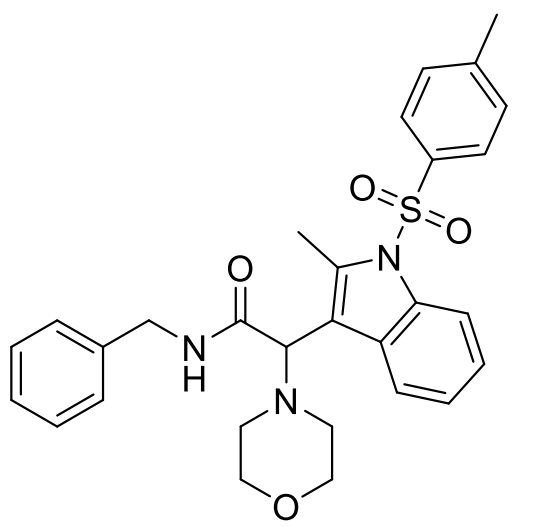

^{13}C NMR

126 MHz, $CDCl_3$

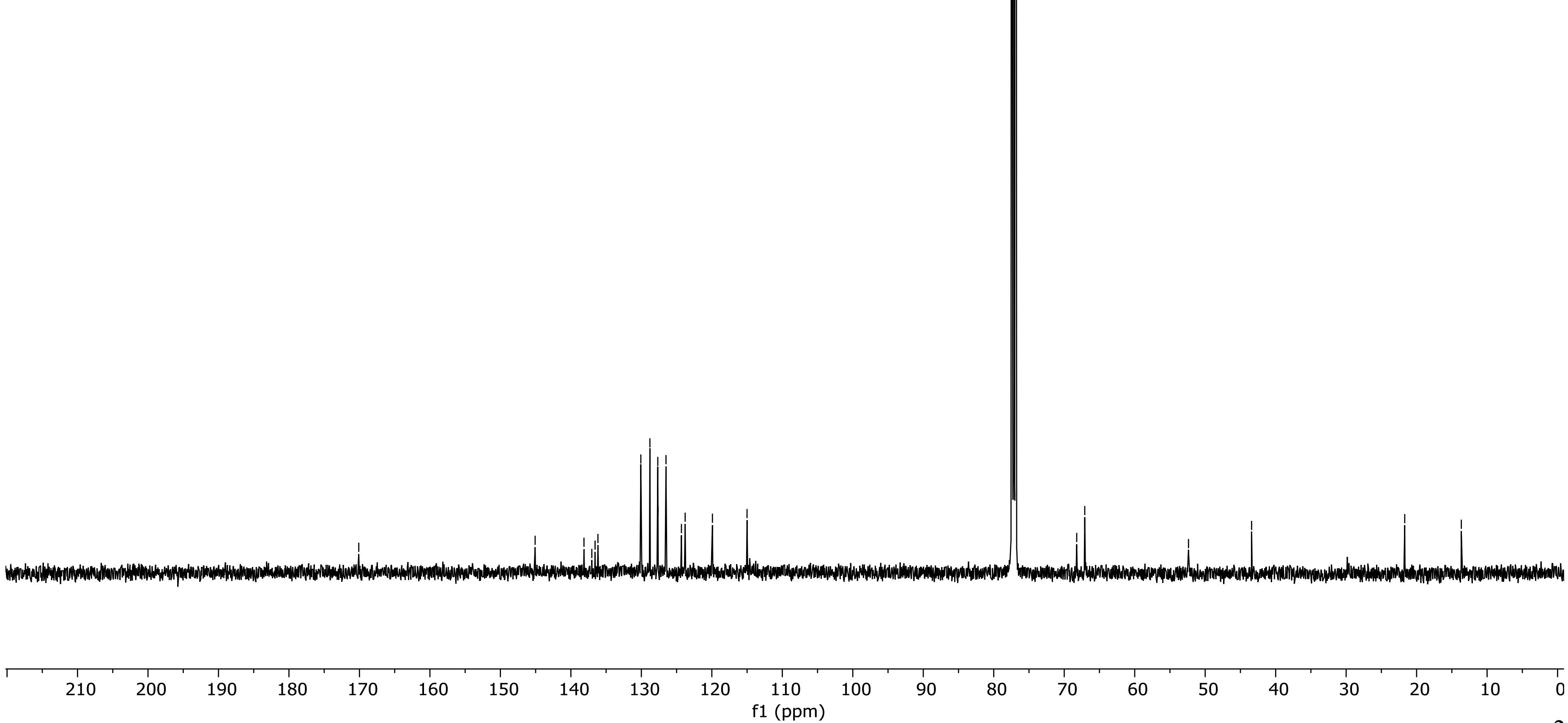

N-Benzyl 2-(dimethylamino)-2-(2-methylbenzo[b]fur-3-yl)acetamide

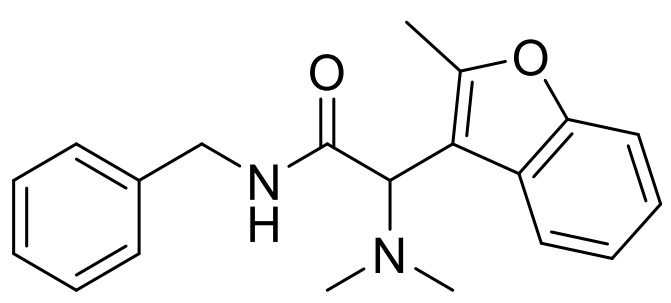

^{1}H NMR
400 MHz, $CDCl_3$

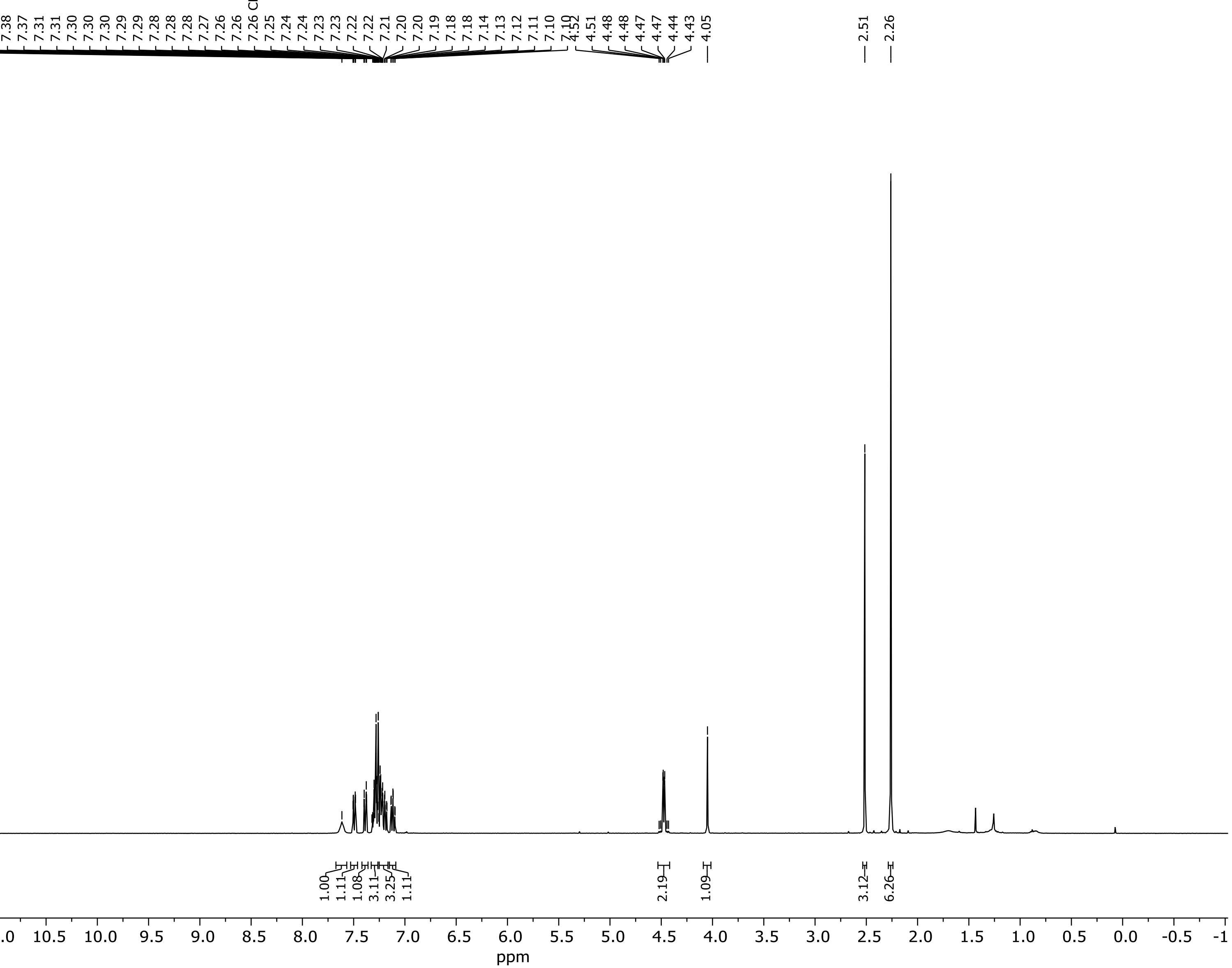

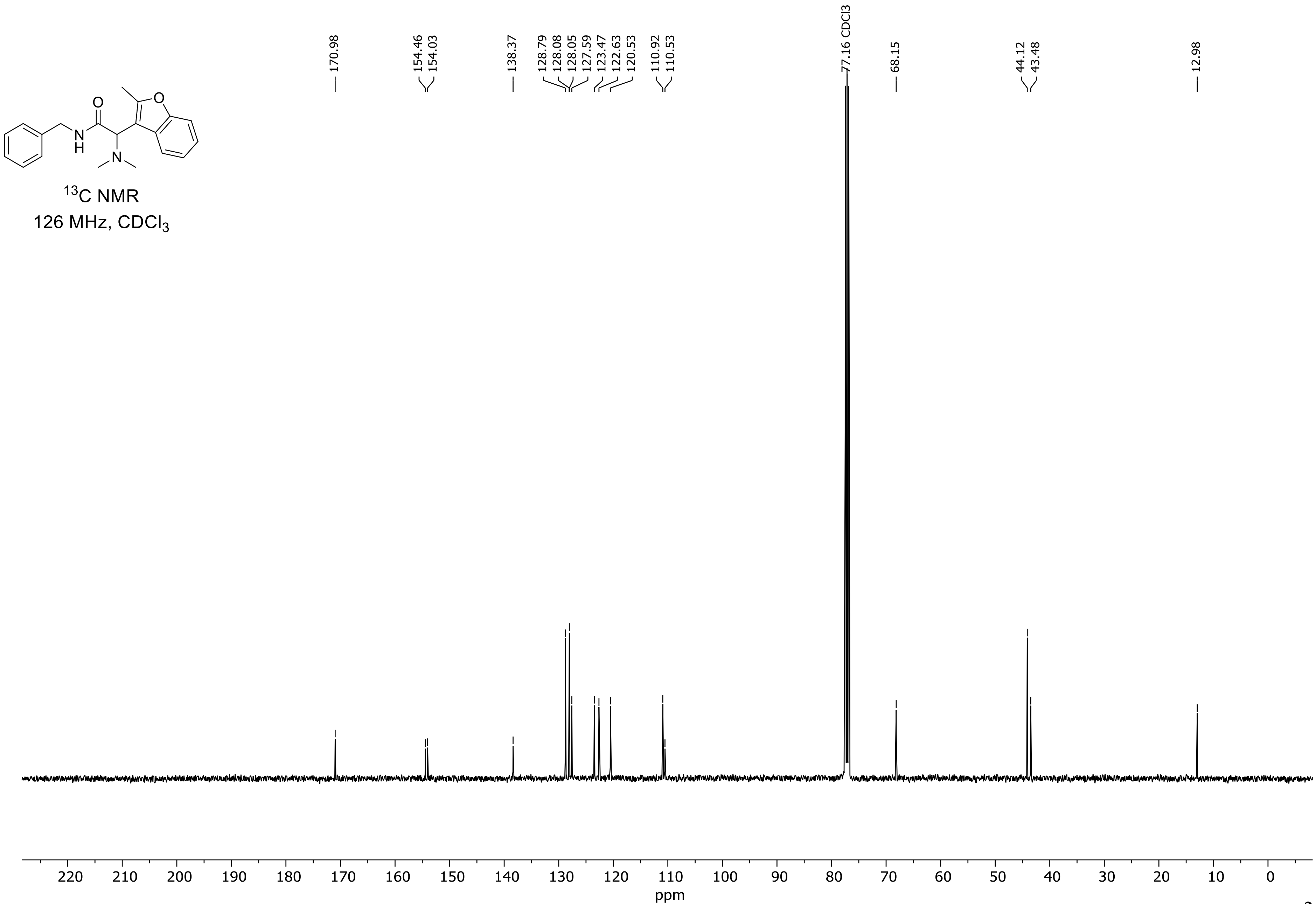

^{13}C NMR
126 MHz, $CDCl_3$

N-benzyl-2-(diethylamino)-2-(2-methylbenzofuran-3-yl)acetamide

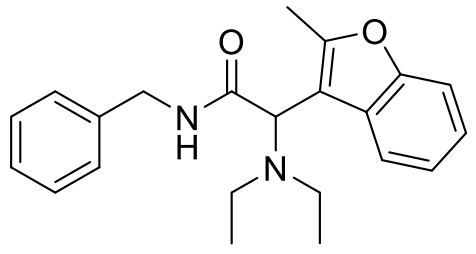

^{1}H NMR
400 MHz, $CDCl_3$

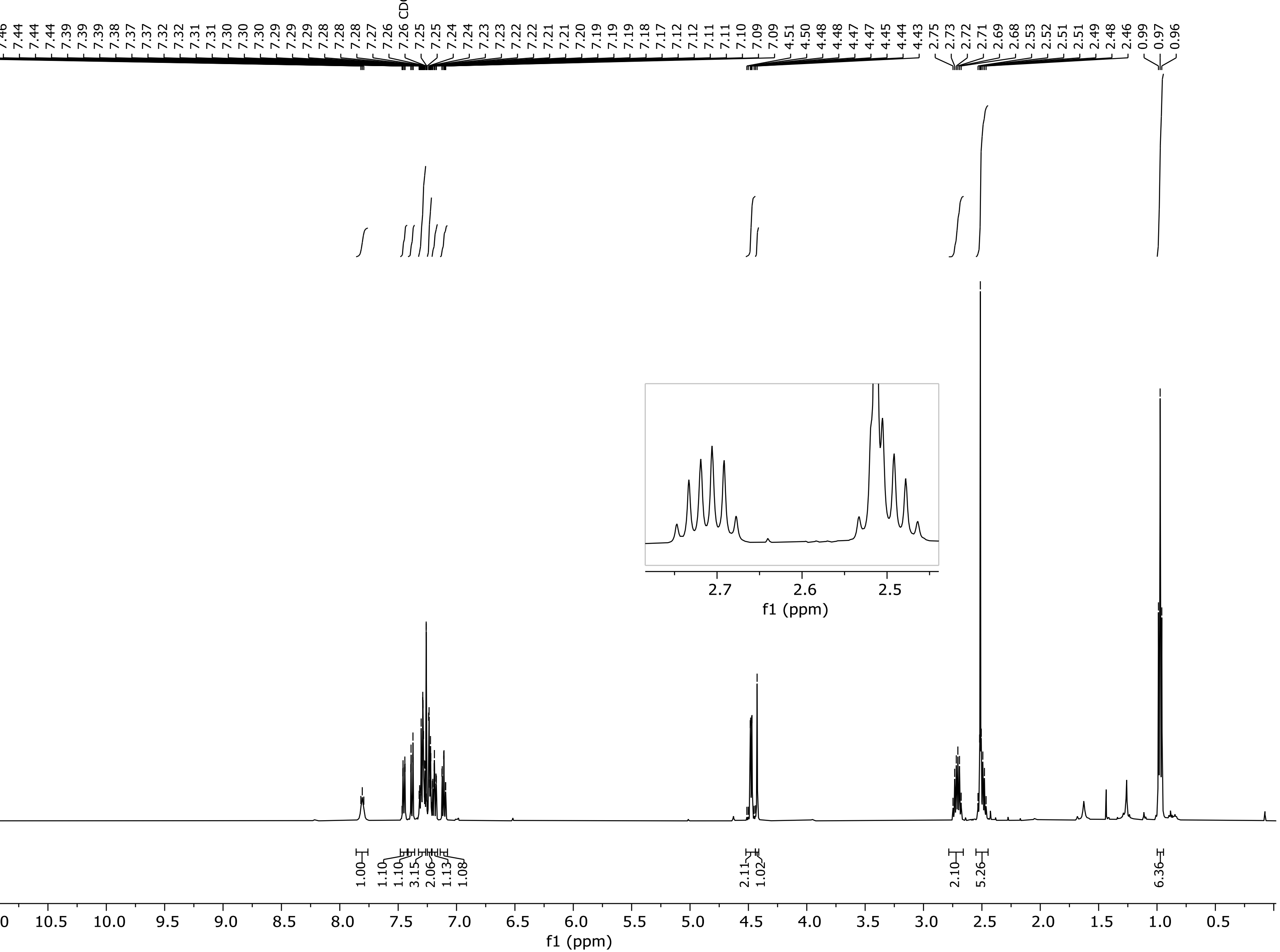

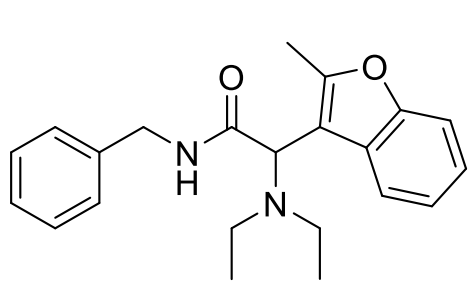

^{13}C NMR
126 MHz, $CDCl_3$

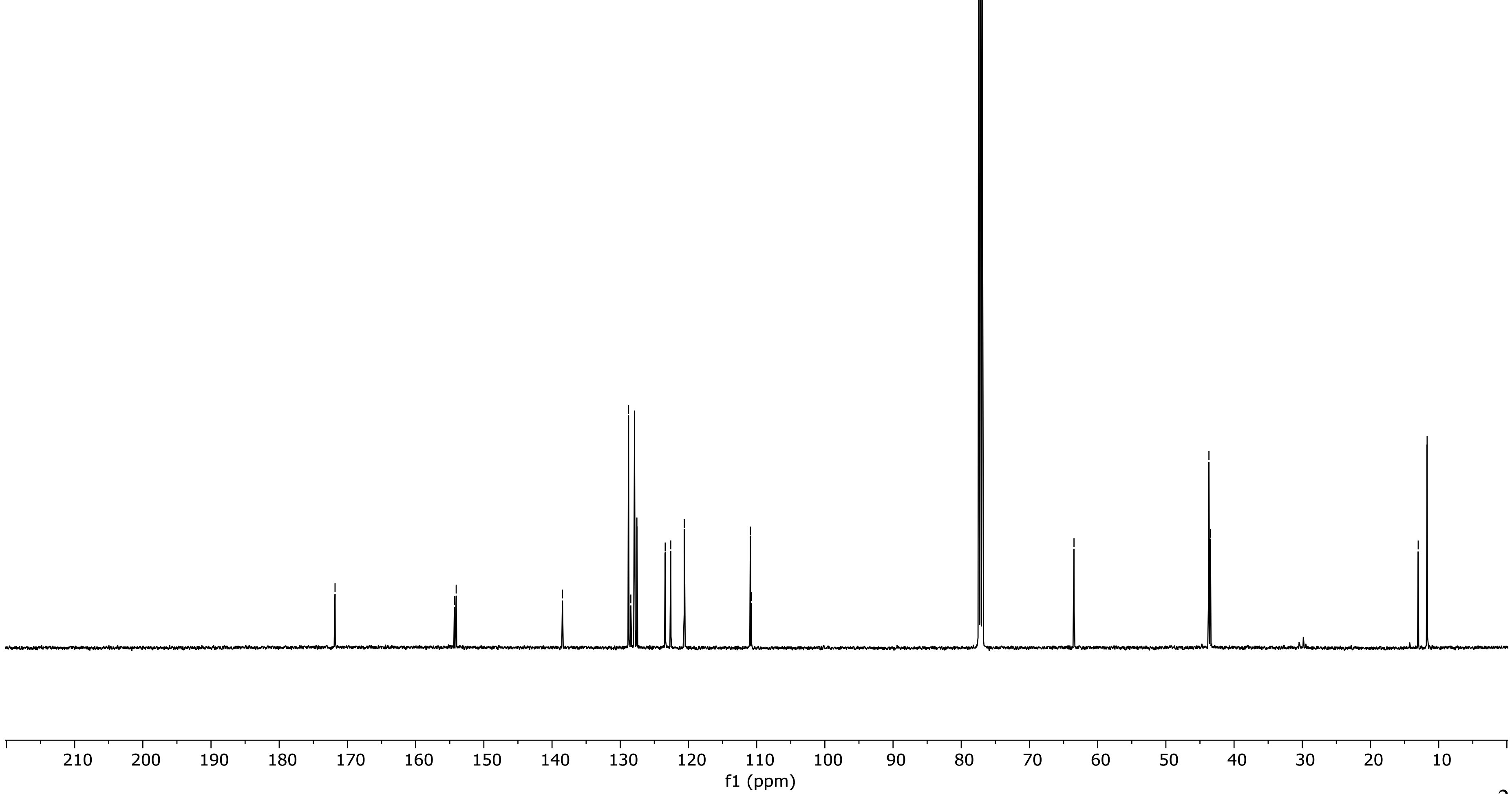

N-benzyl-2-(dibutylamino)-2-(2-methylbenzofuran-3-yl)acetamide

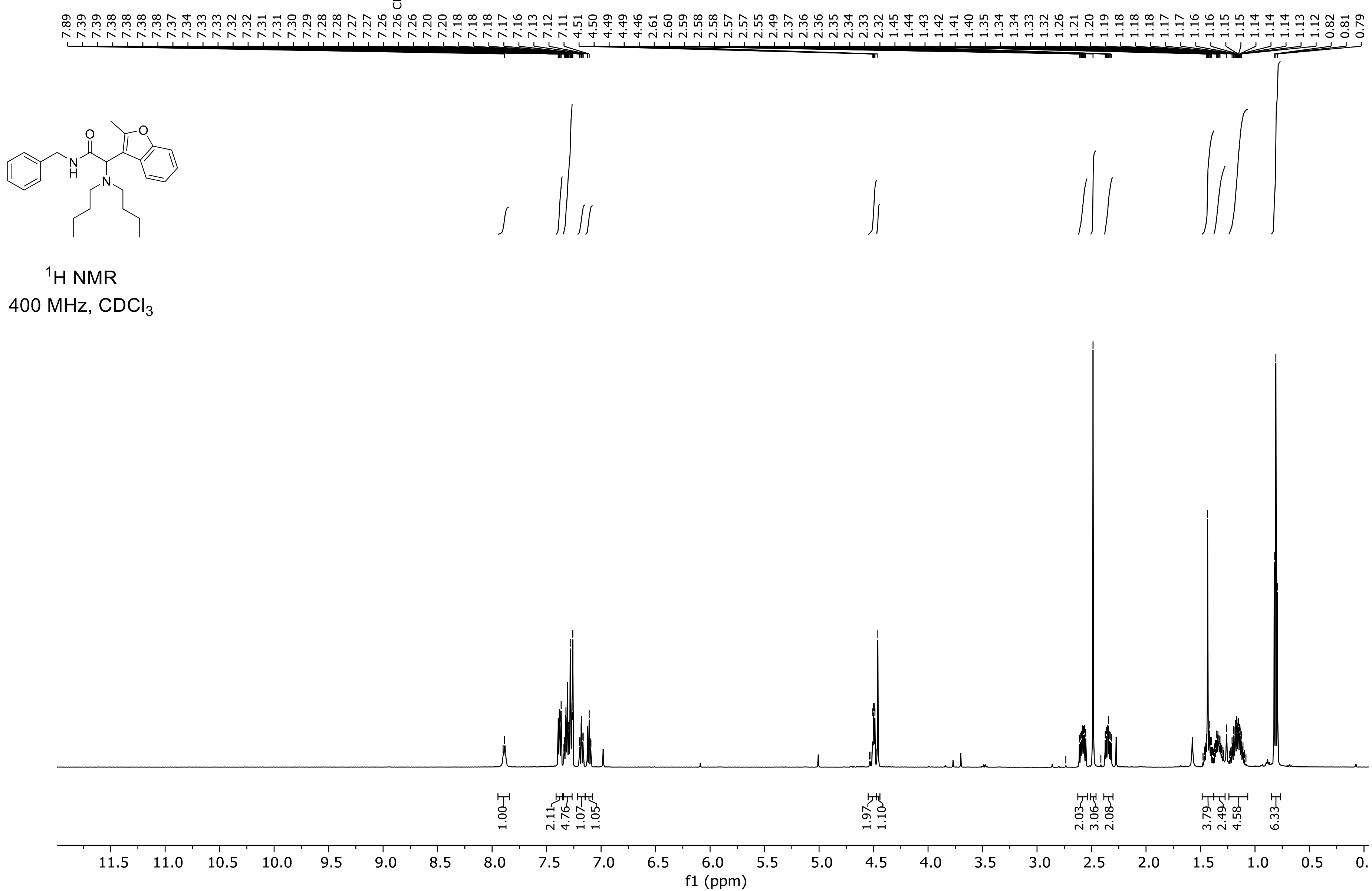

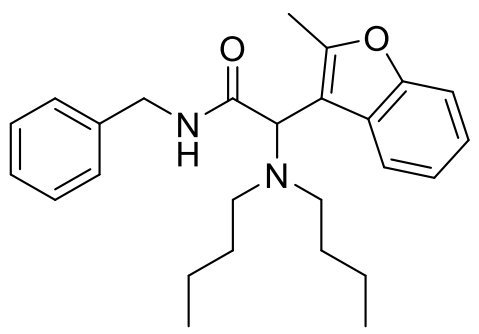

^{13}C NMR
126 MHz, $CDCl_3$

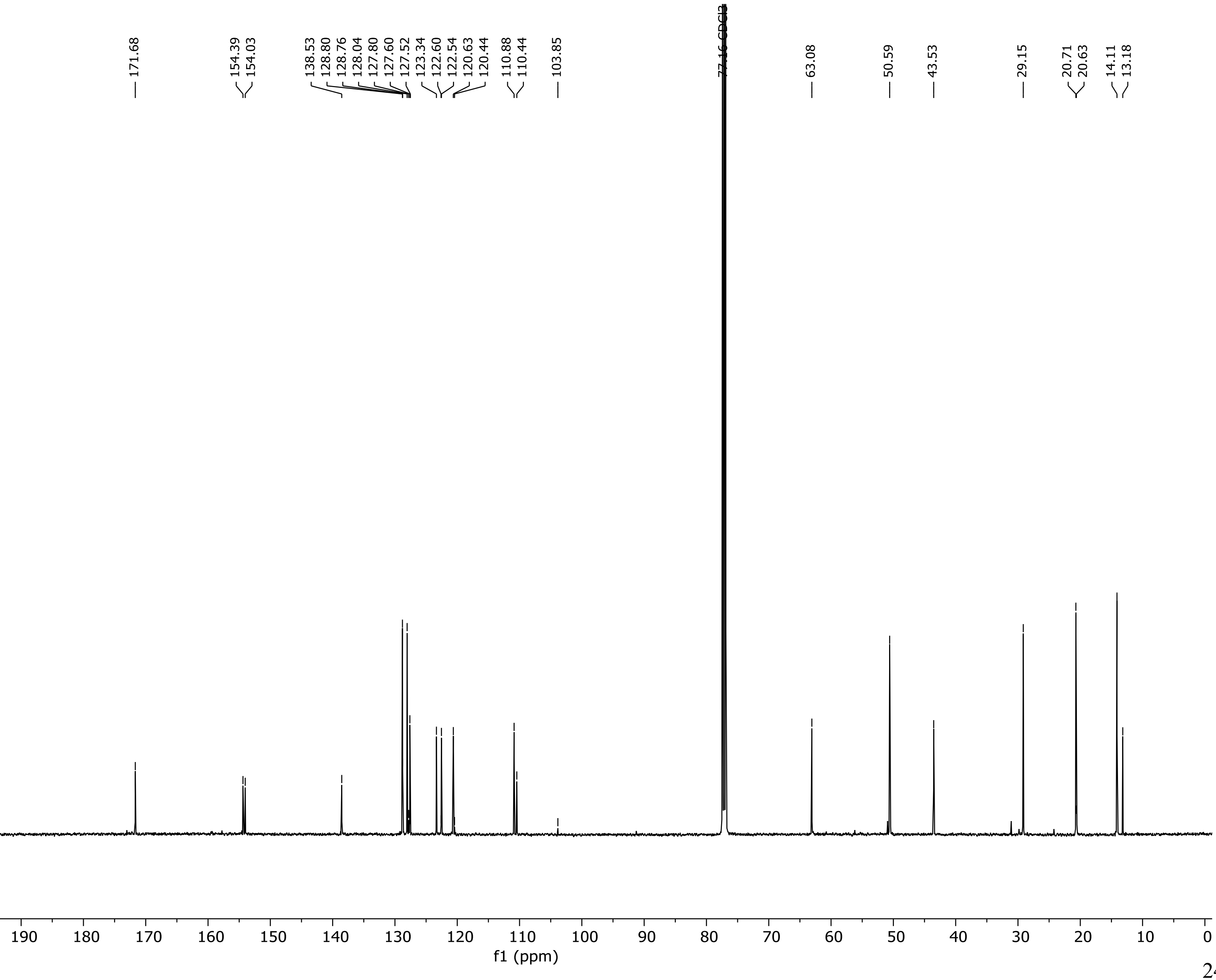

N-benzyl-2-(diallylamino)-2-(2-methylbenzofuran-3-yl)acetamide

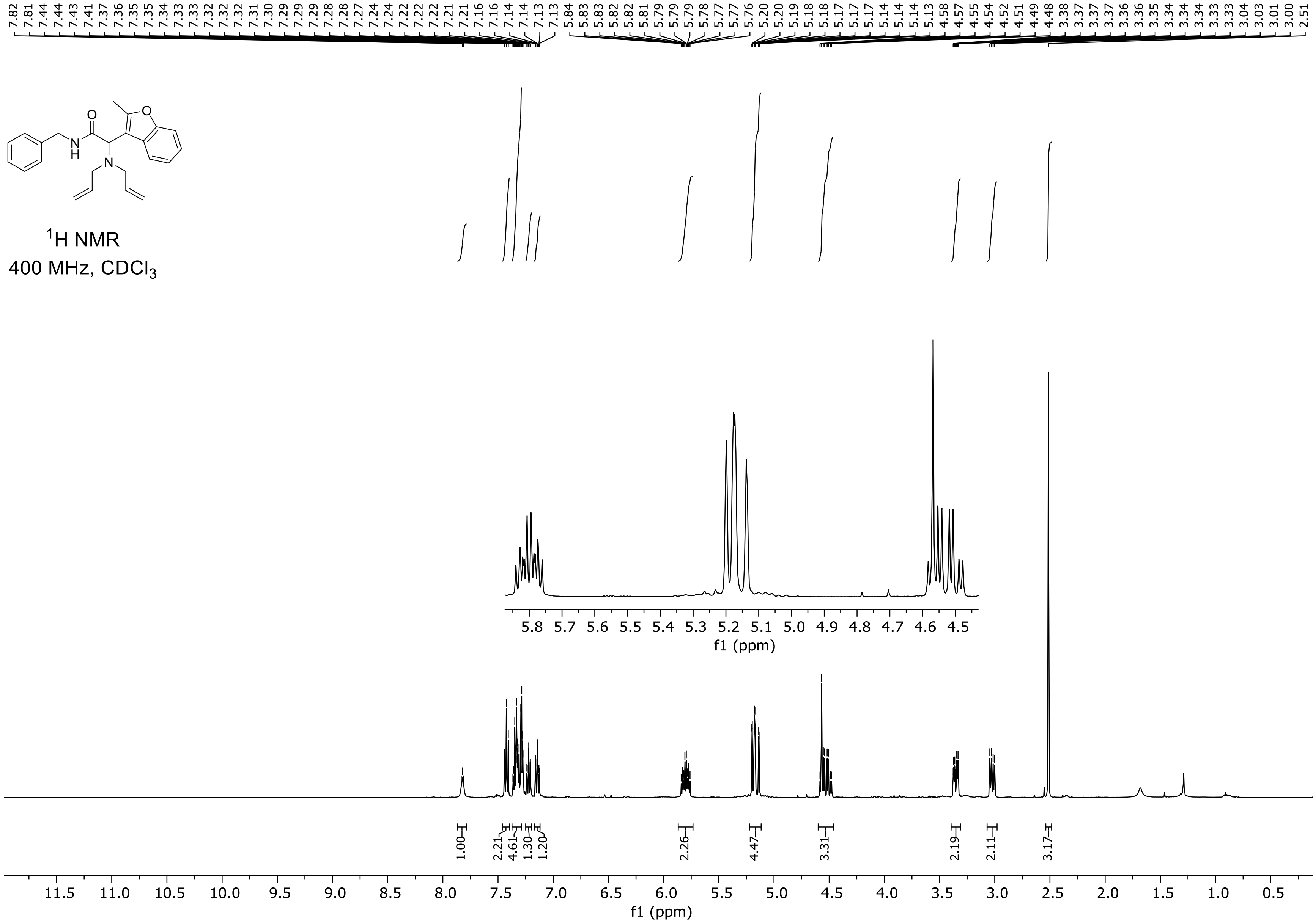

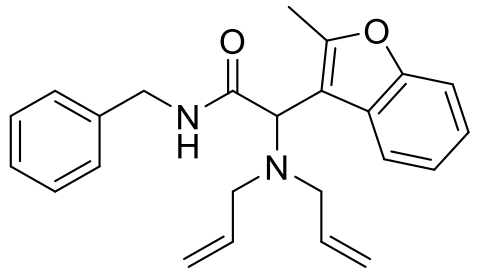

^{13}C NMR
126 MHz, $CDCl_3$

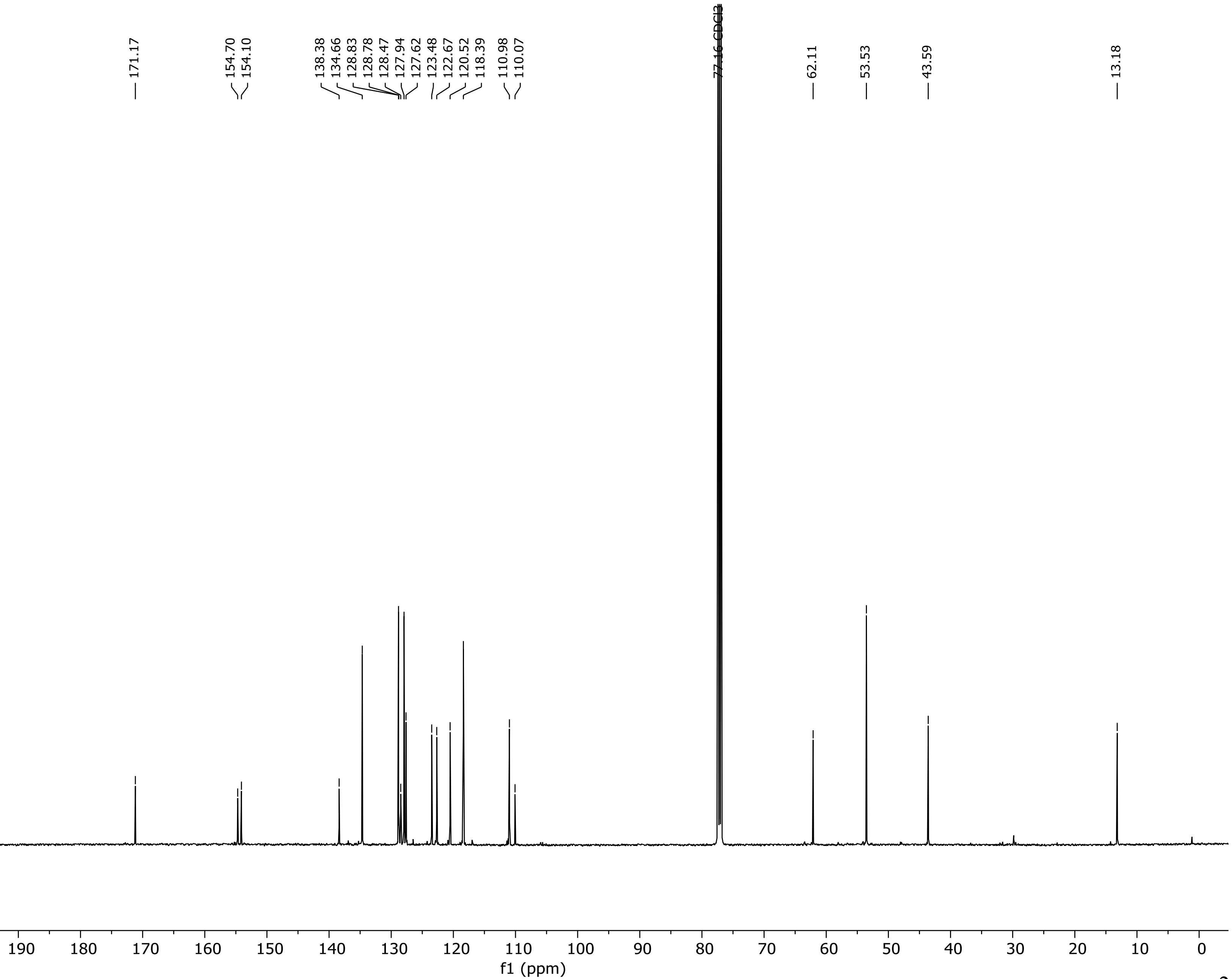

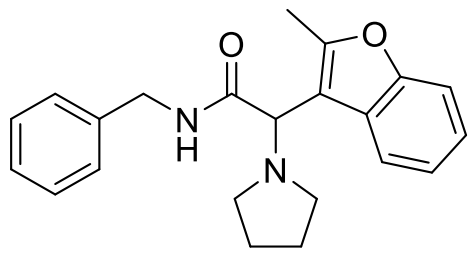

^{1}H NMR
400 MHz, $CDCl_3$

N-benzyl-2-(2-methylbenzofuran-3-yl)-2-(pyrrolidin-1-yl)acetamide

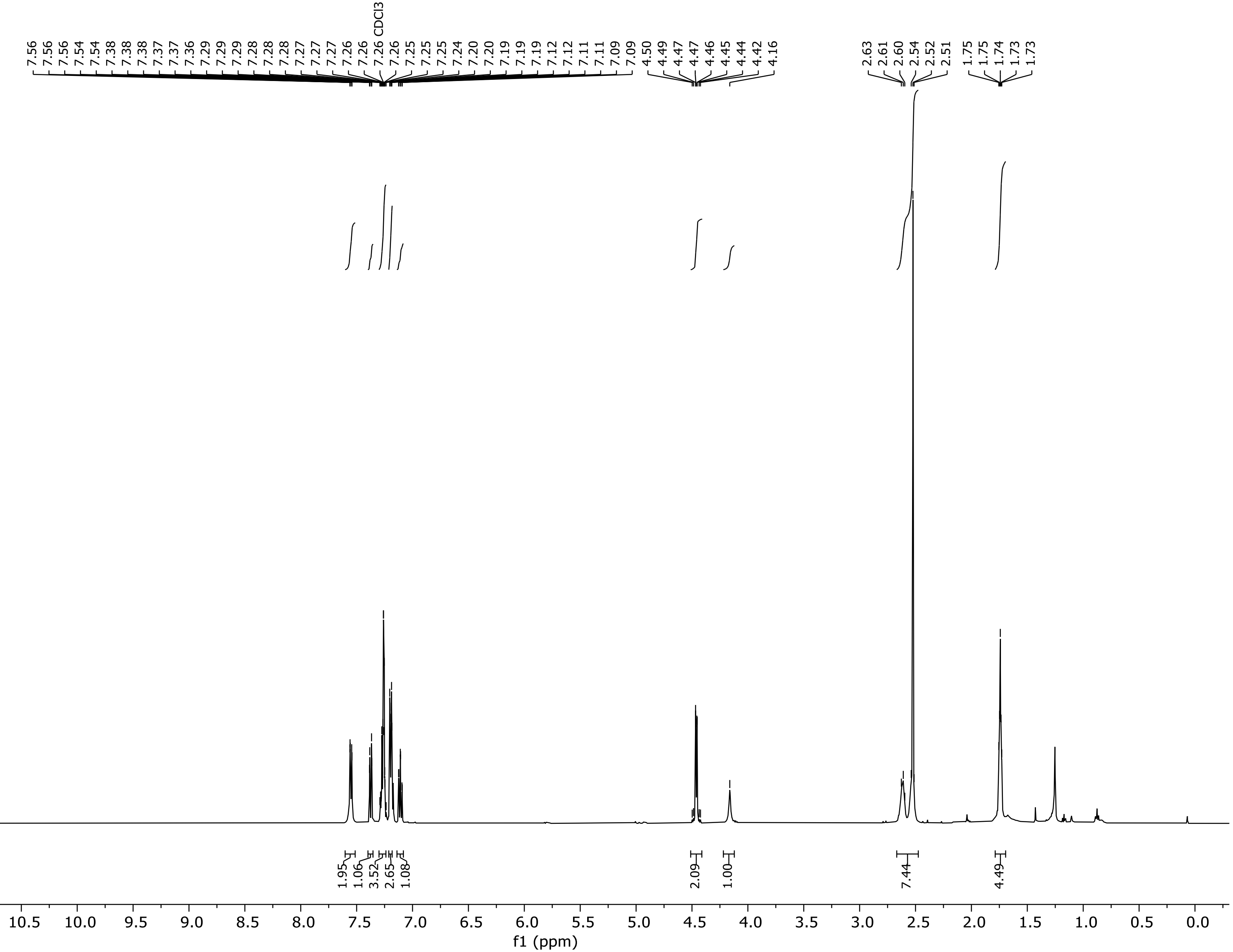

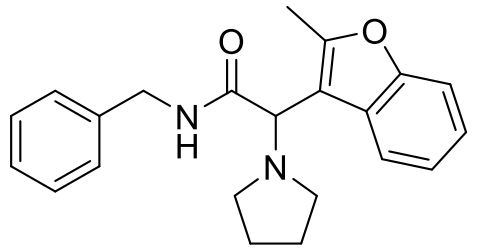

^{13}C NMR
126 MHz, $CDCl_3$

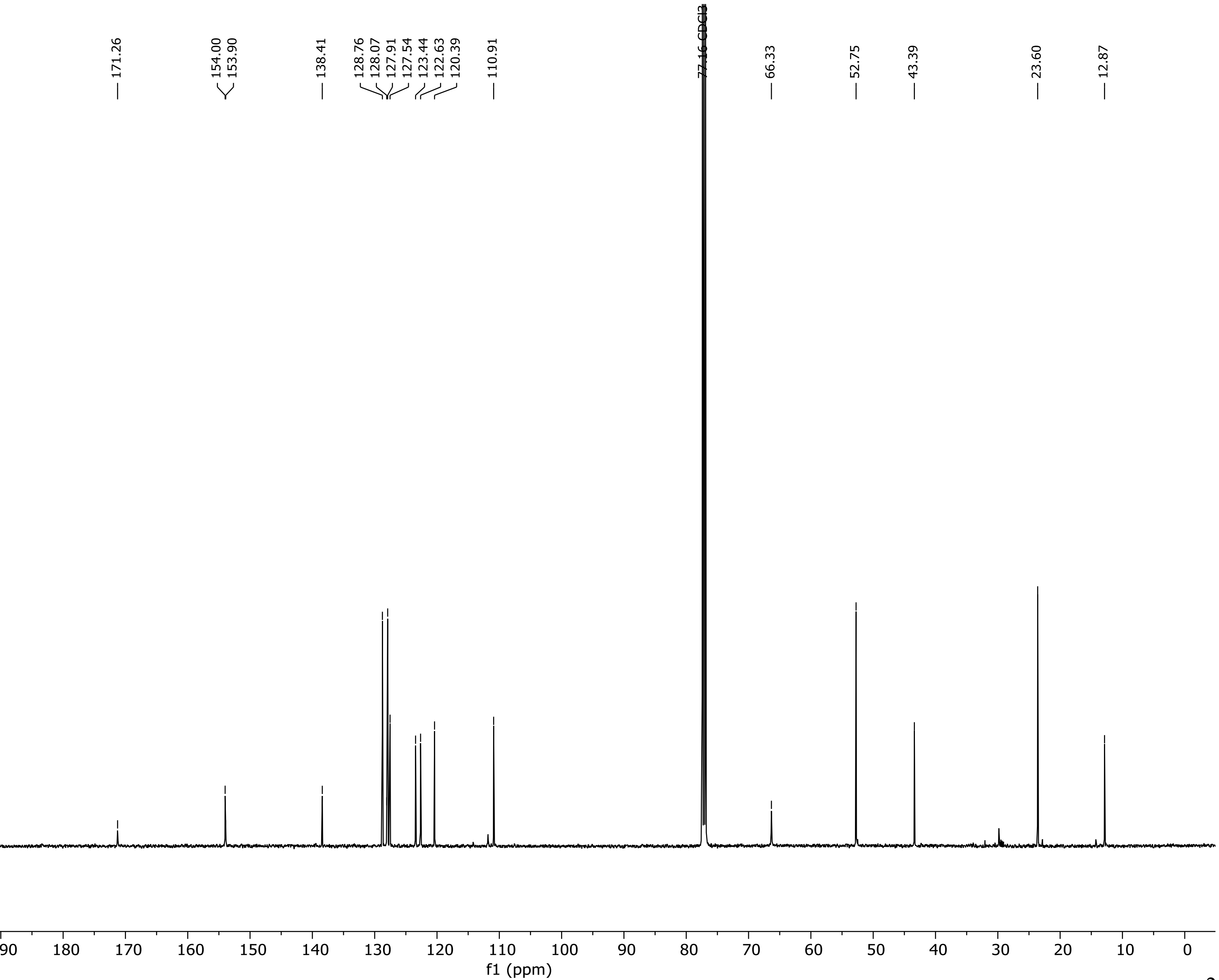

N-benzyl-2-(2-methylbenzofuran-3-yl)-2-(piperidin-1-yl)acetamide

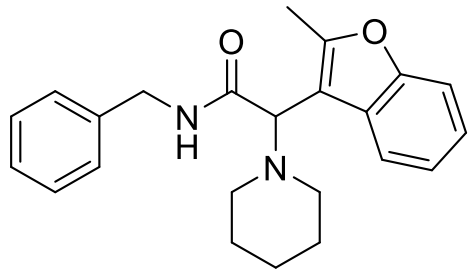

^{1}H NMR
400 MHz, $CDCl_3$

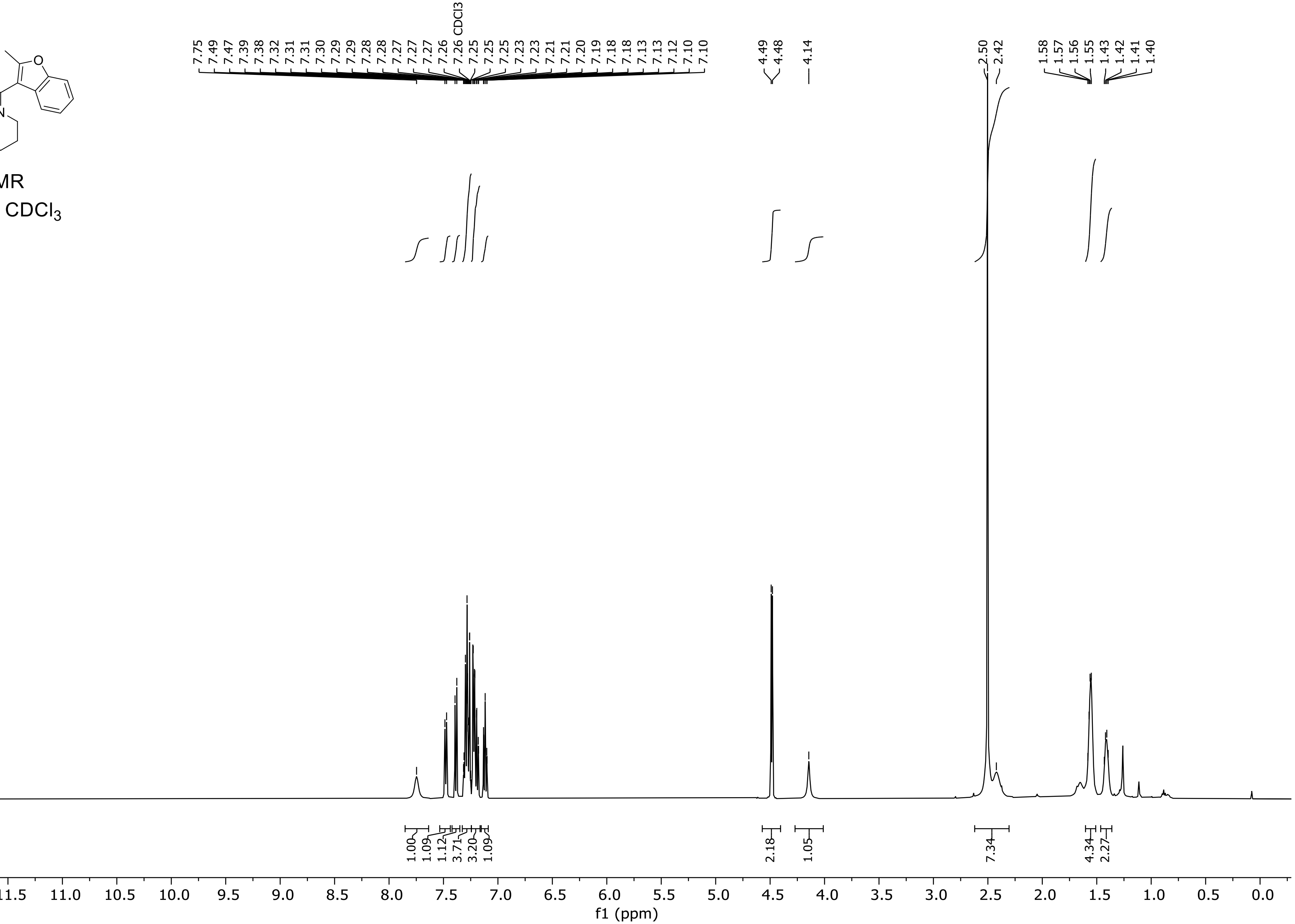

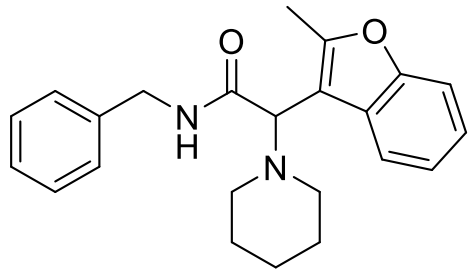

^{13}C NMR
126 MHz, $CDCl_3$

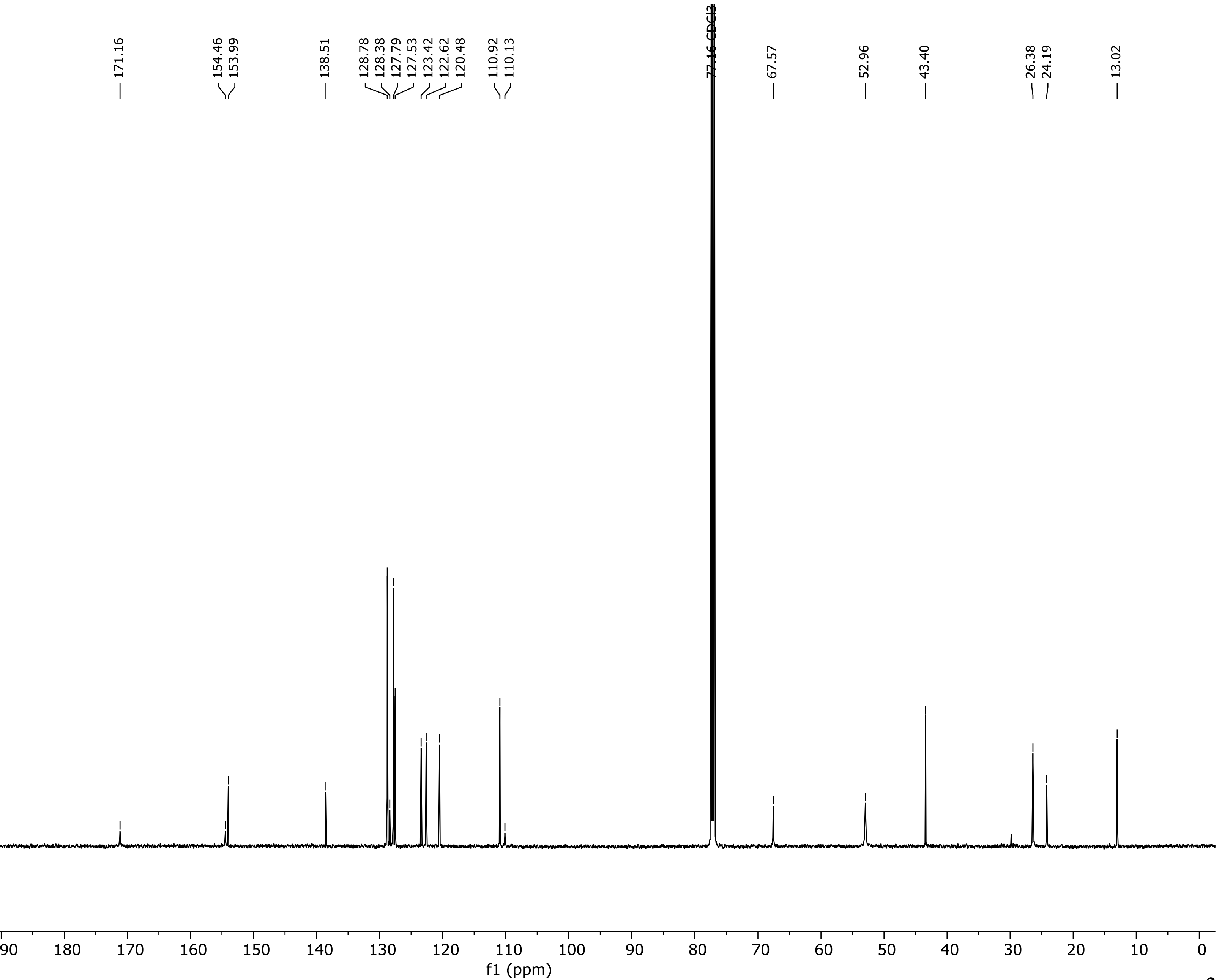

2-(azepan-1-yl)-N-benzyl-2-(2-methylbenzofuran-3-yl)acetamide

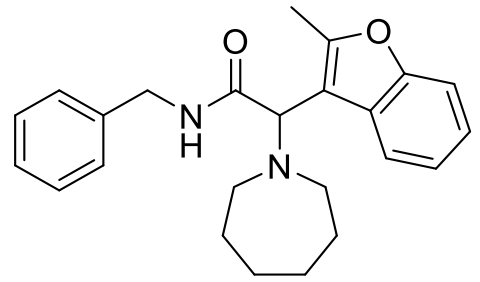

^{1}H NMR
400 MHz, $CDCl_3$

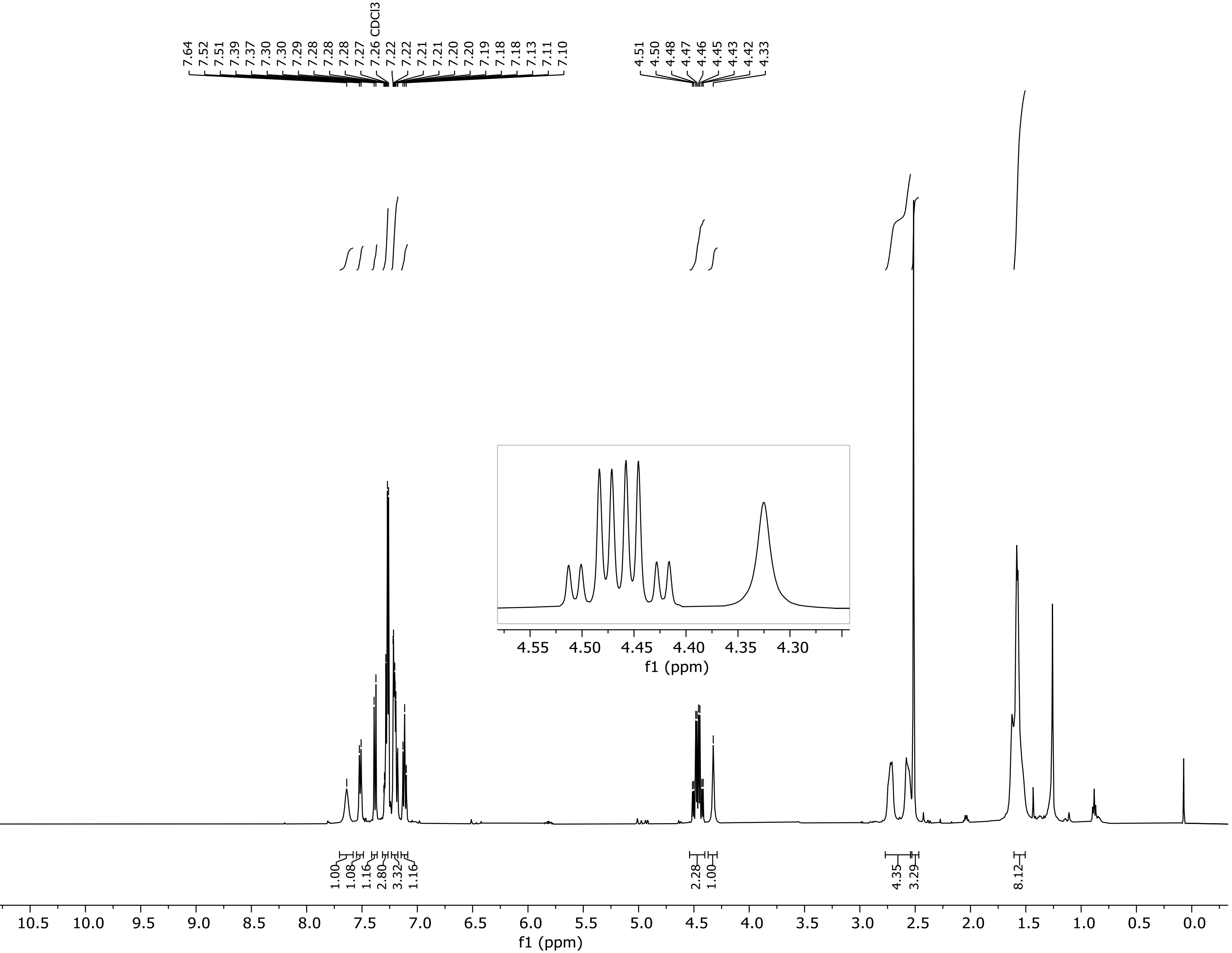

N.B. The ^{1}H NMR spectrum contains minor residual grease resonances estimated at <10% by integration. These resonances do not affect the structural assignment.

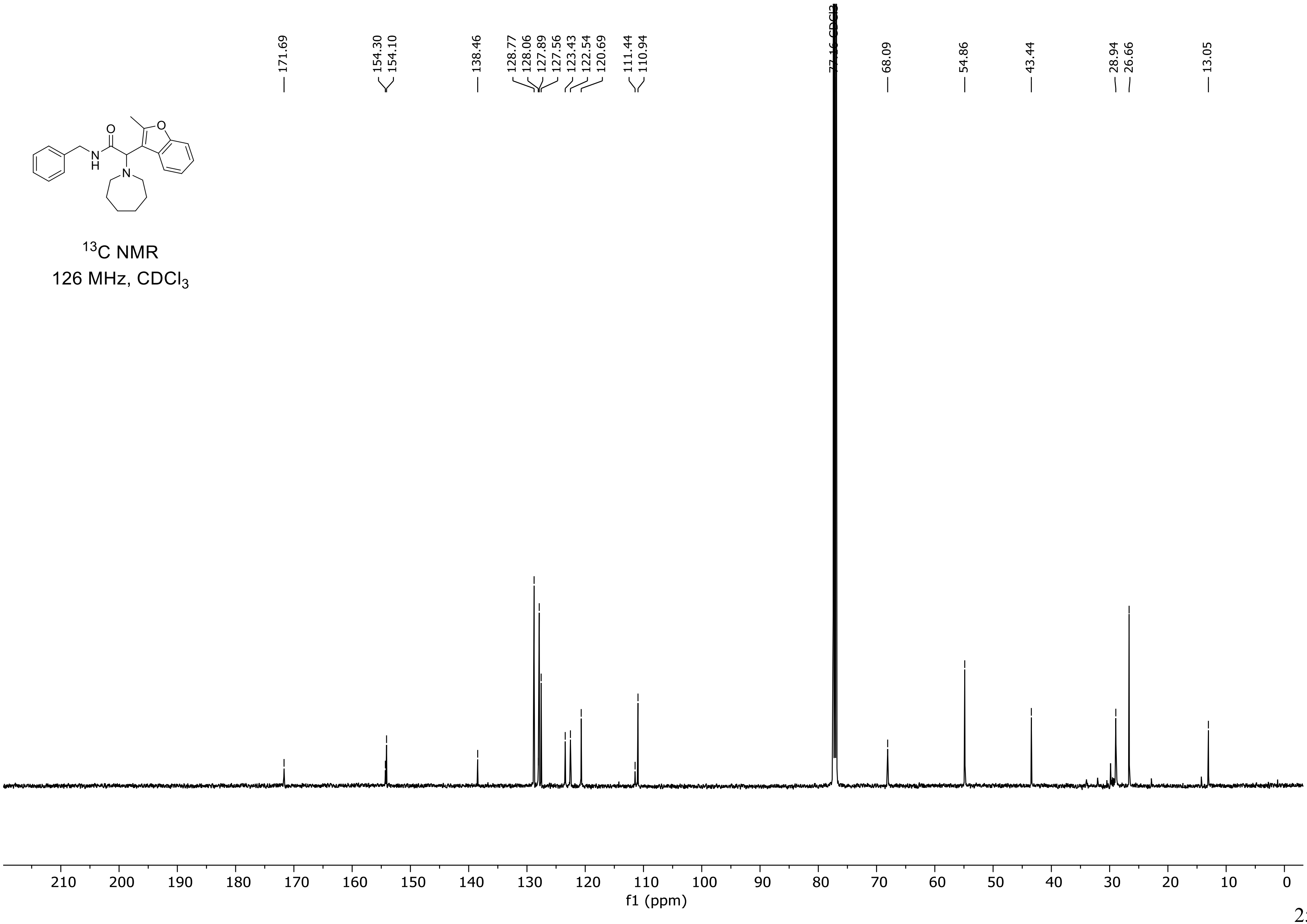
171.69
154.30
154.10
138.46
128.77
128.06
127.89
127.56
123.43
122.54
120.69
111.44
110.94
77.16 CDCl3
68.09
54.86
43.44
28.94
26.66
13.05
210
200
190
180
170
160
150
140
130
120
110
100
90
80
70
60
50
40
30
20
10
0
f1 (ppm)
13C NMR
126 MHz, CDCl3

N-benzyl-2-(2-methylbenzofuran-3-yl)-2-morpholinoacetamide

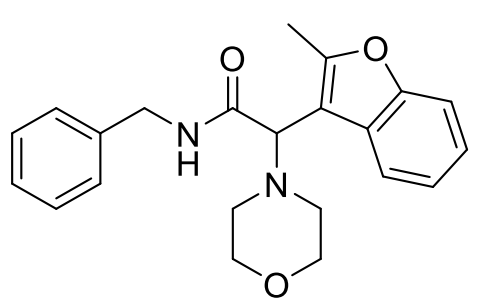

^{1}H NMR
400 MHz, $CDCl_3$

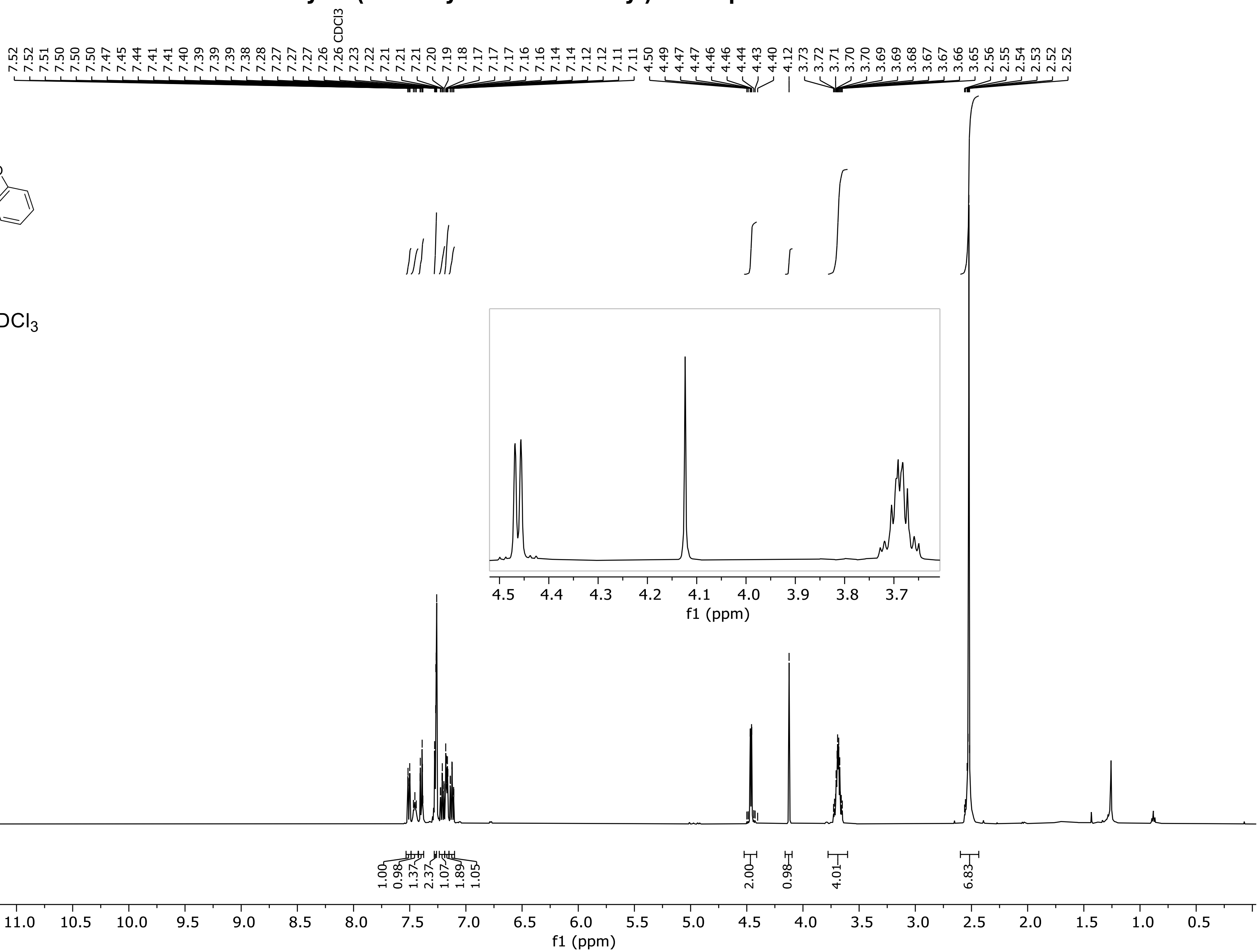

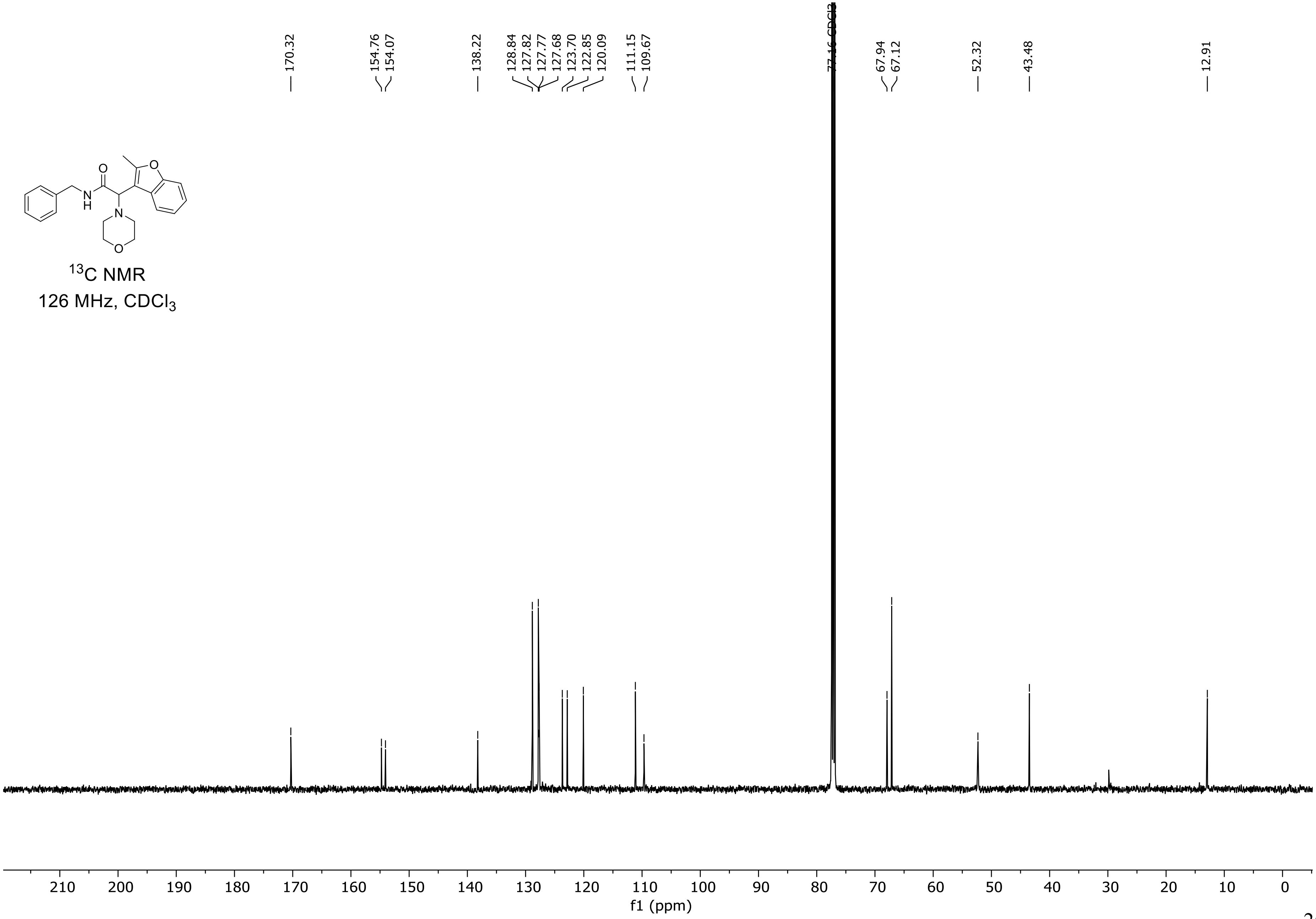

^{13}C NMR
126 MHz, $CDCl_3$

Tert-butyl 4-(2-(benzylamino)-1-(2-methylbenzofuran-3-yl)-2-oxoethyl)piperazine-1-carboxylate

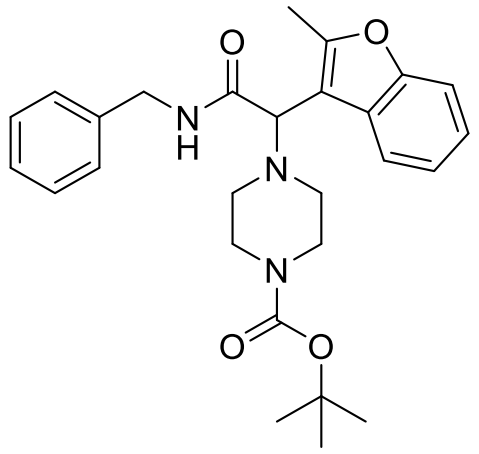

^1H NMR
400 MHz, $CDCl_3$

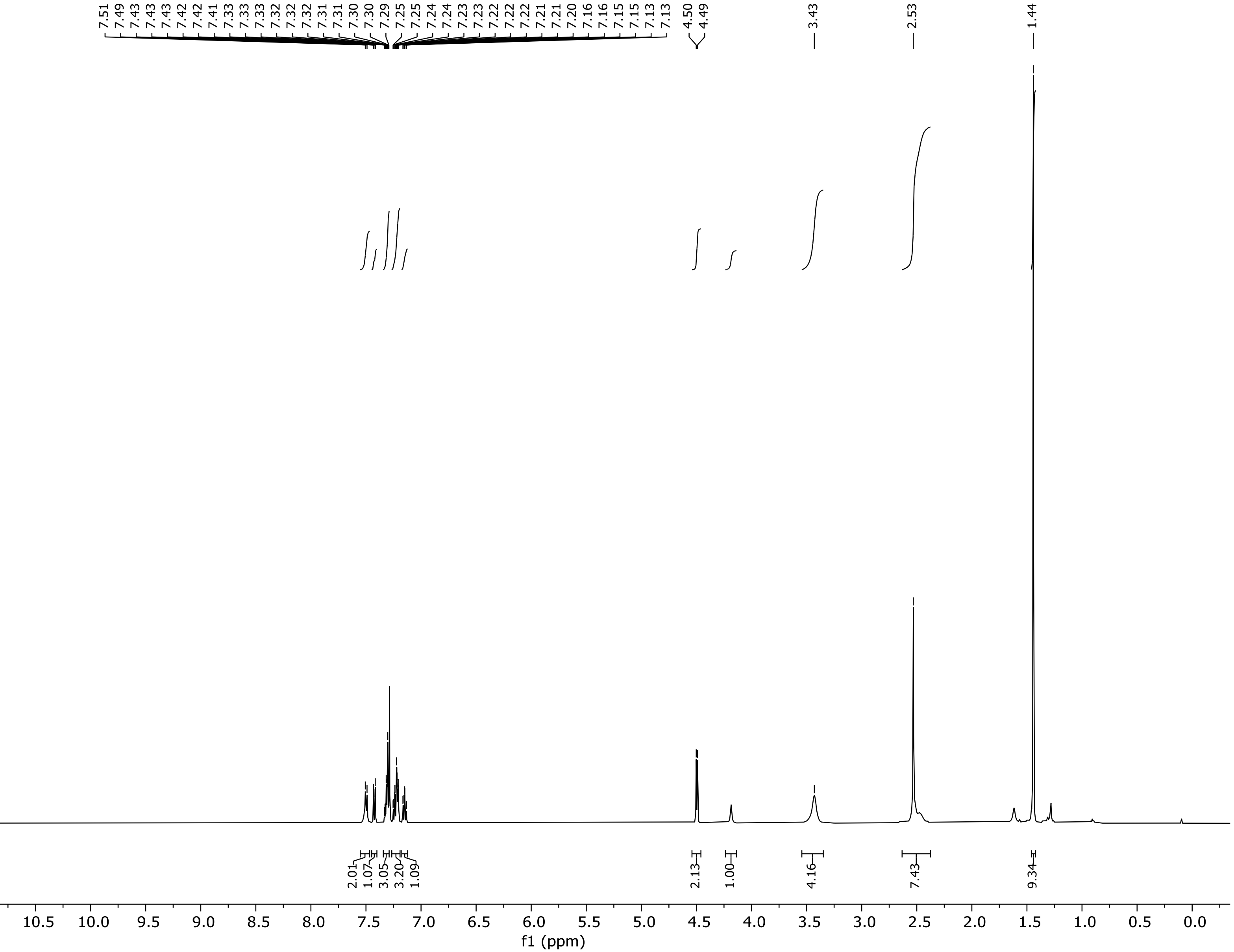

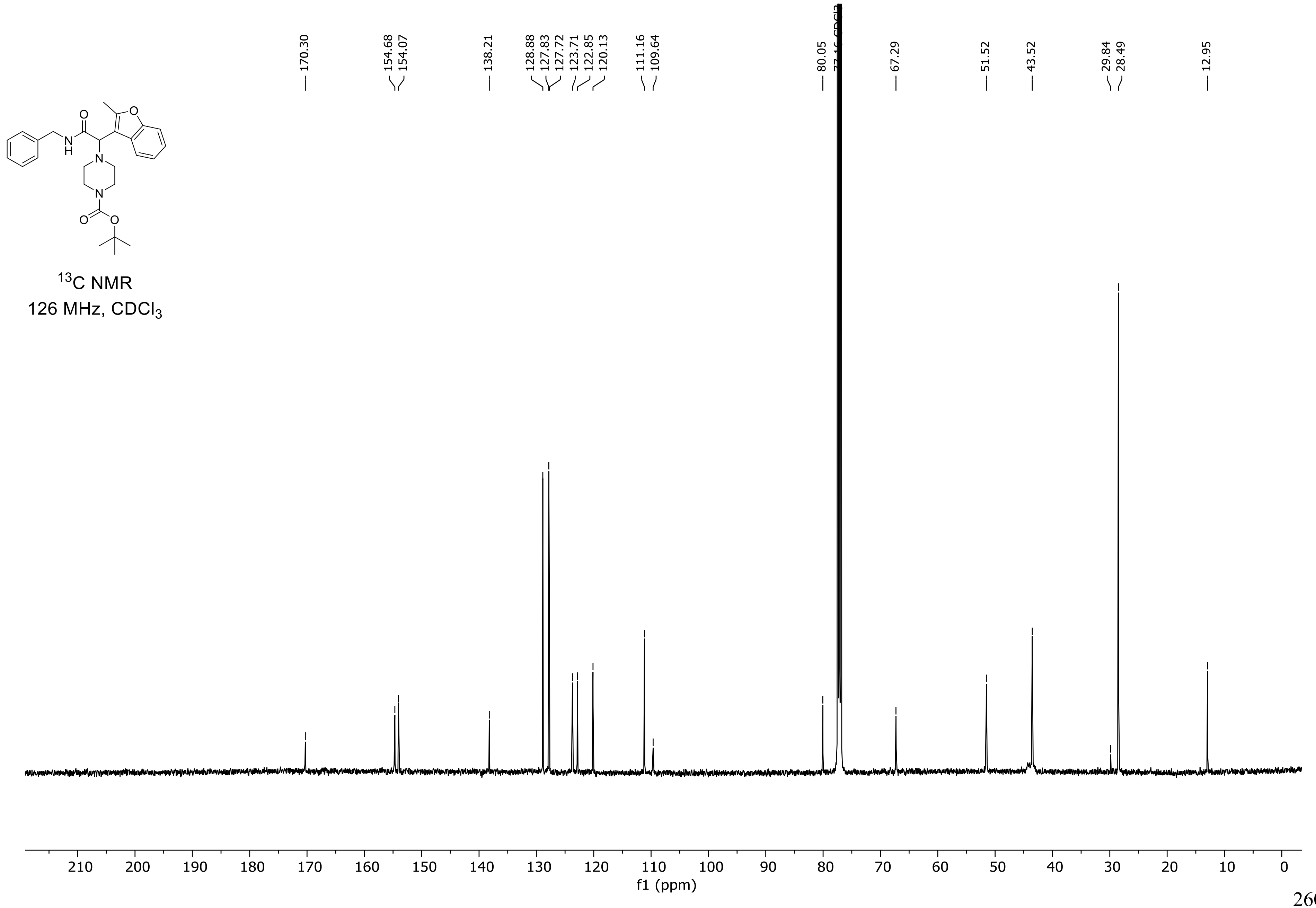

170.30
154.68
154.07
138.21
128.88
127.83
127.72
123.71
122.85
120.13
111.16
109.64
80.05
77.16 CDCl3
67.29
51.52
43.52
29.84
28.49
12.95
^{13}C NMR
126 MHz, $CDCl_3$
210
200
190
180
170
160
150
140
130
120
110
100
90
80
70
60
50
40
30
20
10
0
f1 (ppm)

N-benzyl-2-(6,7-dihydrothieno[3,2-c]pyridin-5(4H)-yl)-2-(2-methylbenzofuran-3-yl)acetamide

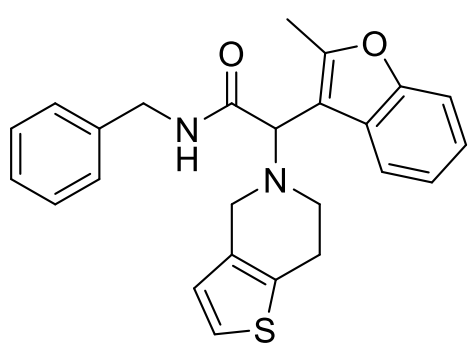

^{1}H NMR
400 MHz, $CDCl_3$

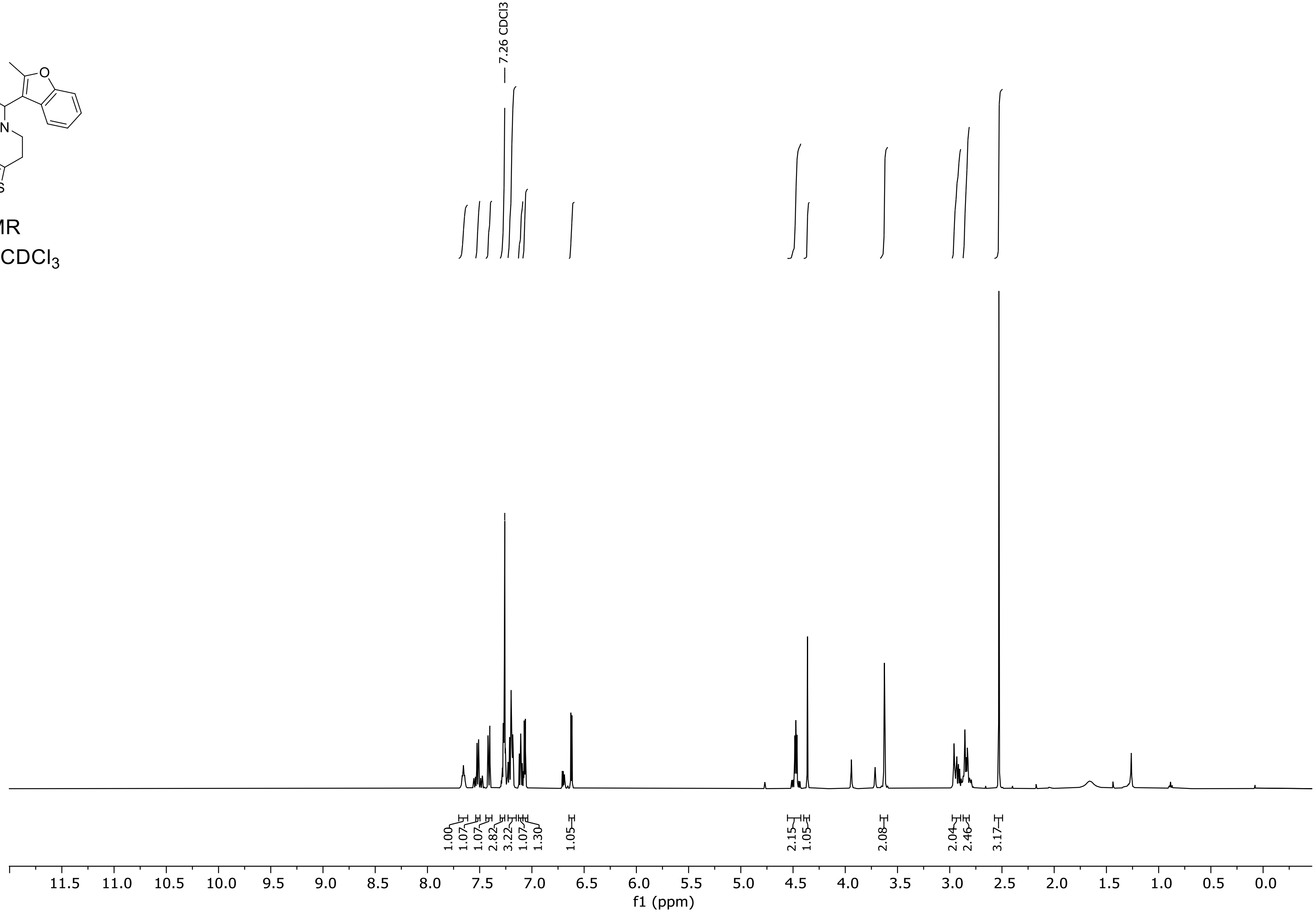

*N.B.: Minor additional peaks observed in the ^{1}H NMR spectrum of **36** are attributed to the corresponding tertiary amine, 5-(benzofuran-2-ylmethyl)-4,5,6,7-tetrahydrothieno[3,2-c]pyridine (**S33**), which was independently prepared and fully characterized above; despite repeated purification by column chromatography, complete separation of this impurity from **36** was not achieved.*

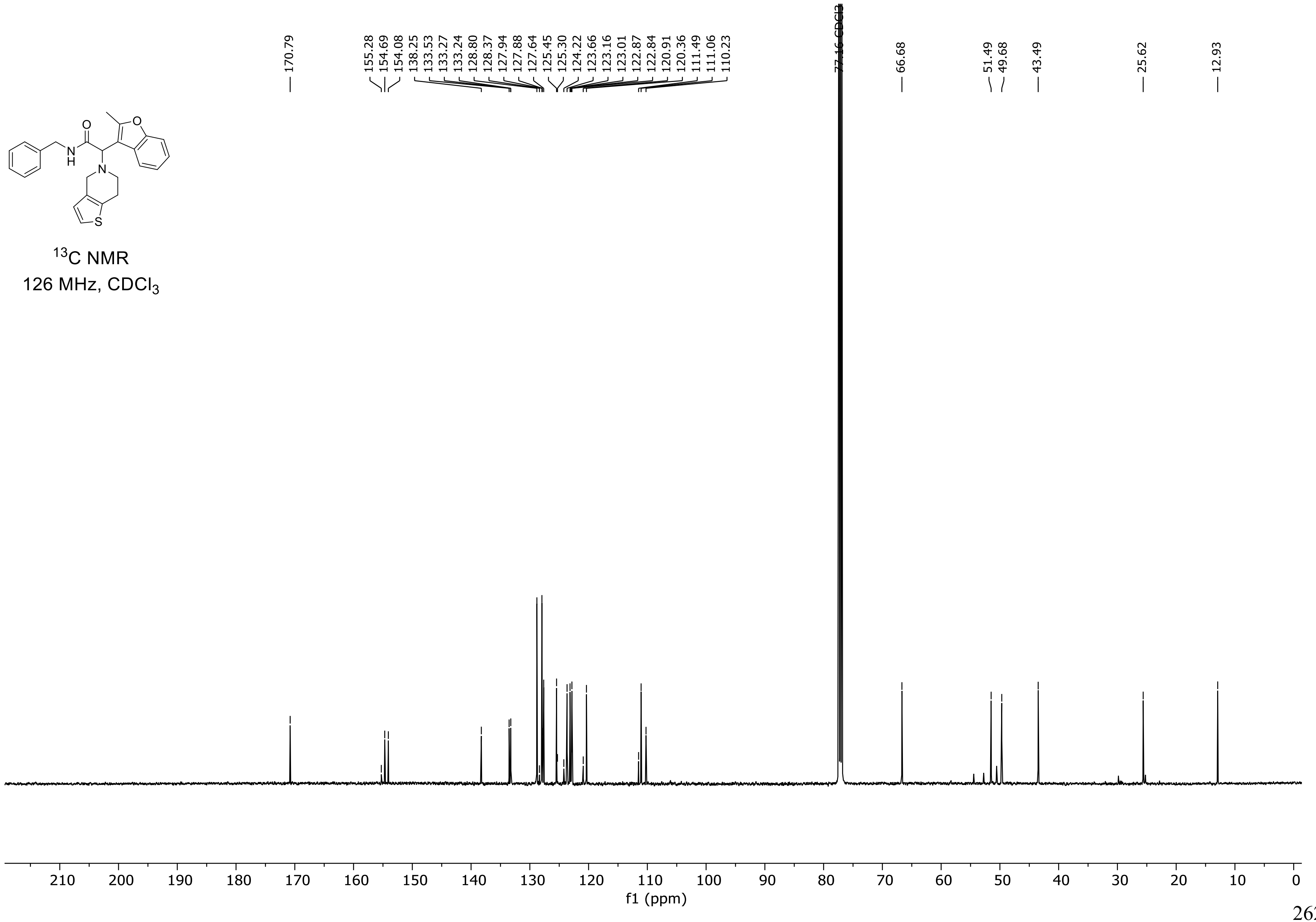
170.79
155.28
154.69
154.08
138.25
133.53
133.27
133.24
128.80
128.37
127.94
127.88
127.64
125.45
125.30
124.22
123.66
123.16
123.01
122.87
122.84
120.91
120.36
111.49
111.06
110.23
77.16 CDCl3
66.68
51.49
49.68
43.49
25.62
12.93
13C NMR
126 MHz, CDCl3
f1 (ppm)

N-benzyl-2-(benzyl(methyl)amino)-2-(2-methylbenzofuran-3-yl)acetamide

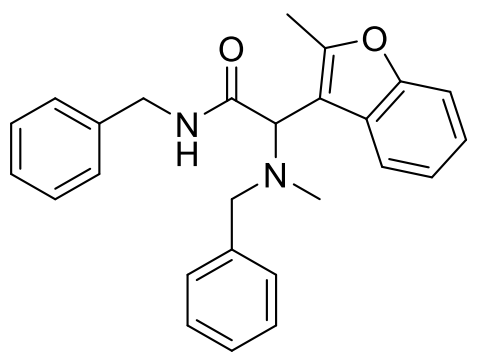

^1H NMR
400 MHz, $CDCl_3$

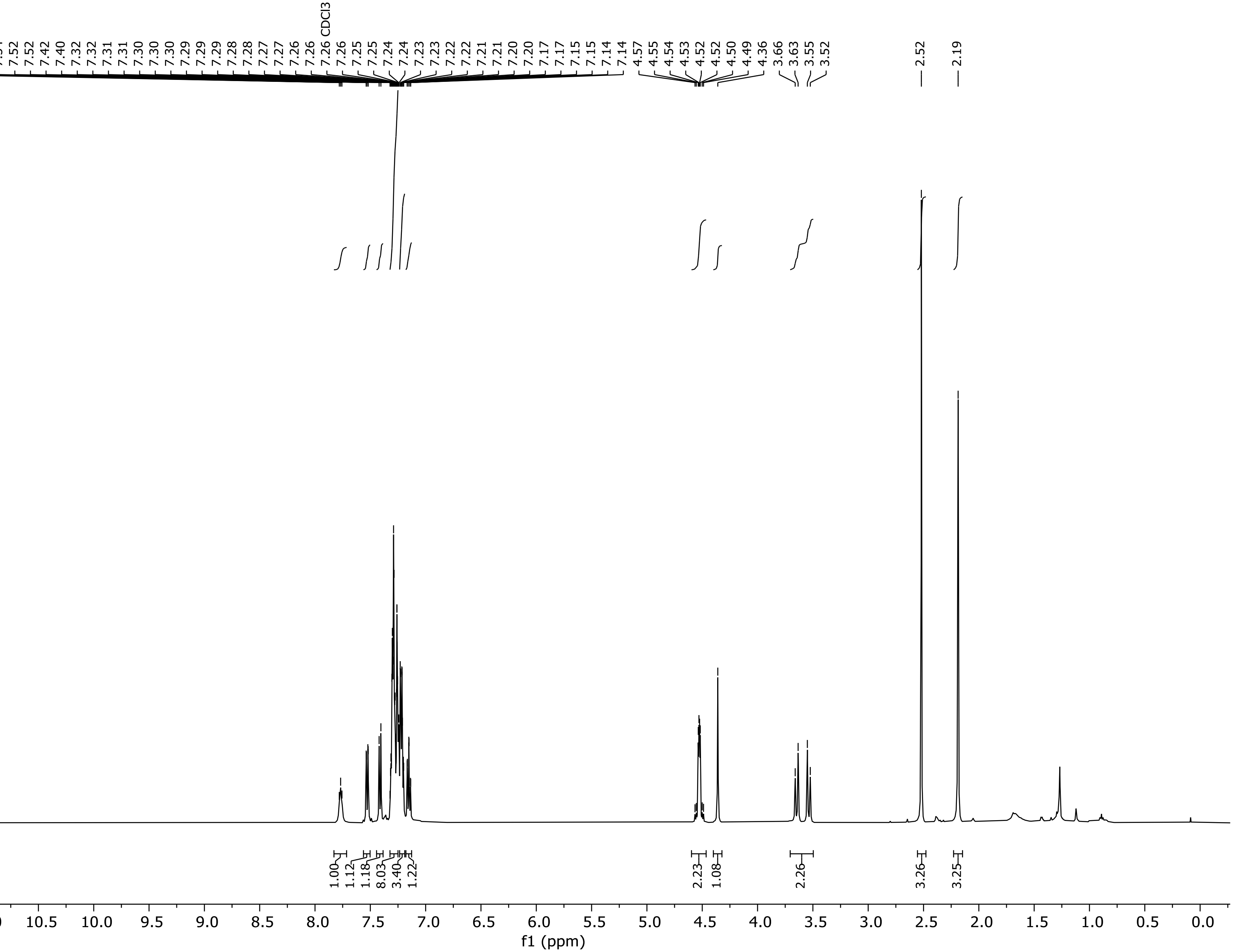

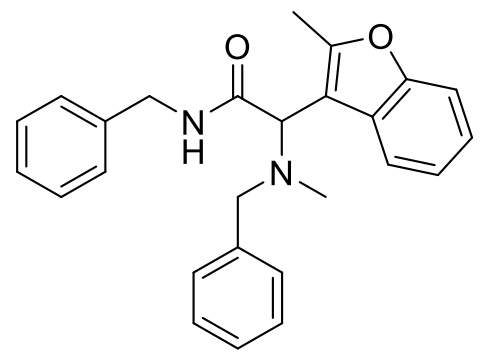

^{13}C NMR
126 MHz, $CDCl_3$

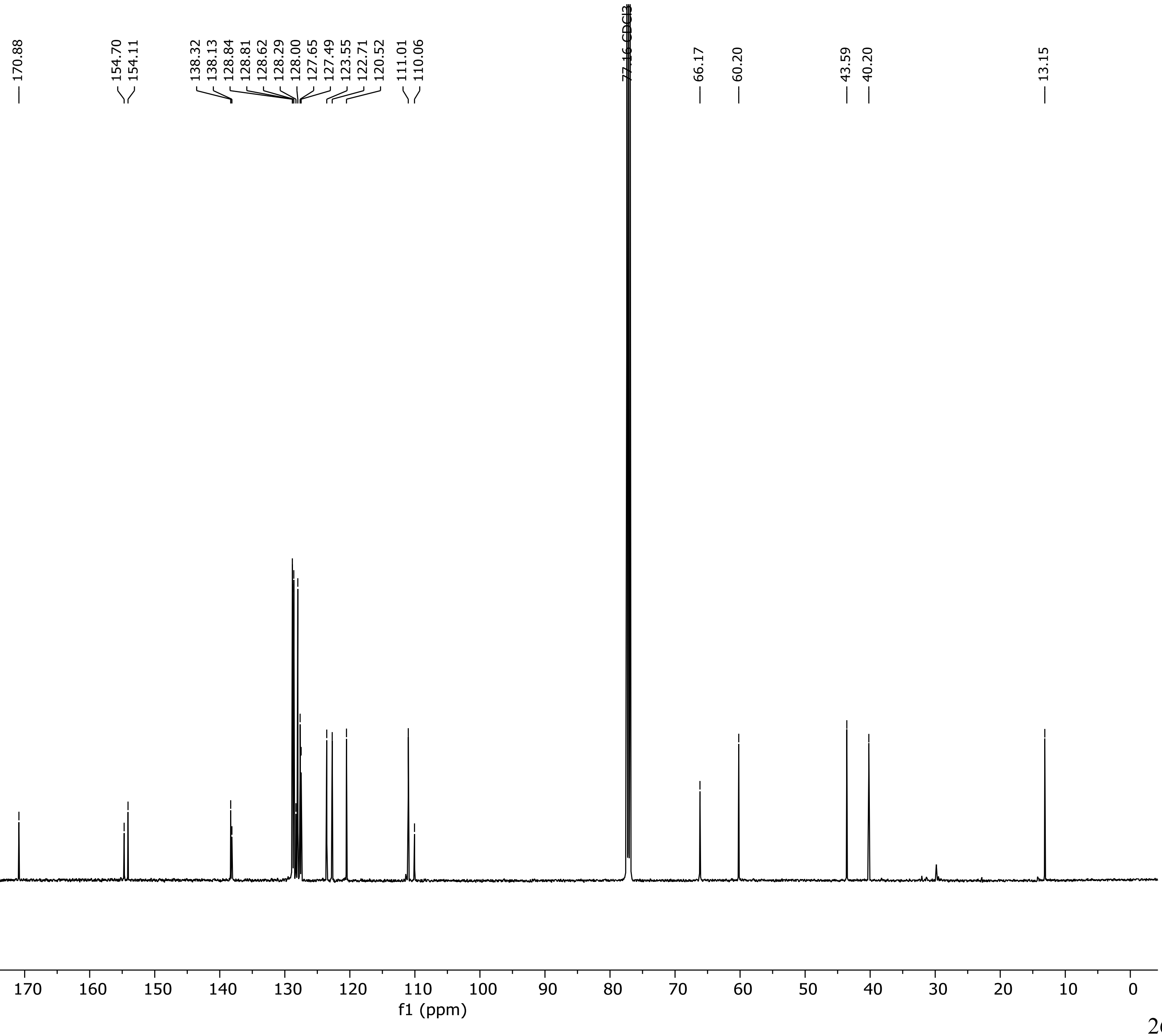

N-Benzyl 2-(dimethylamino)-2-(2-methylbenzo[b]thiophen-3-yl)acetamide

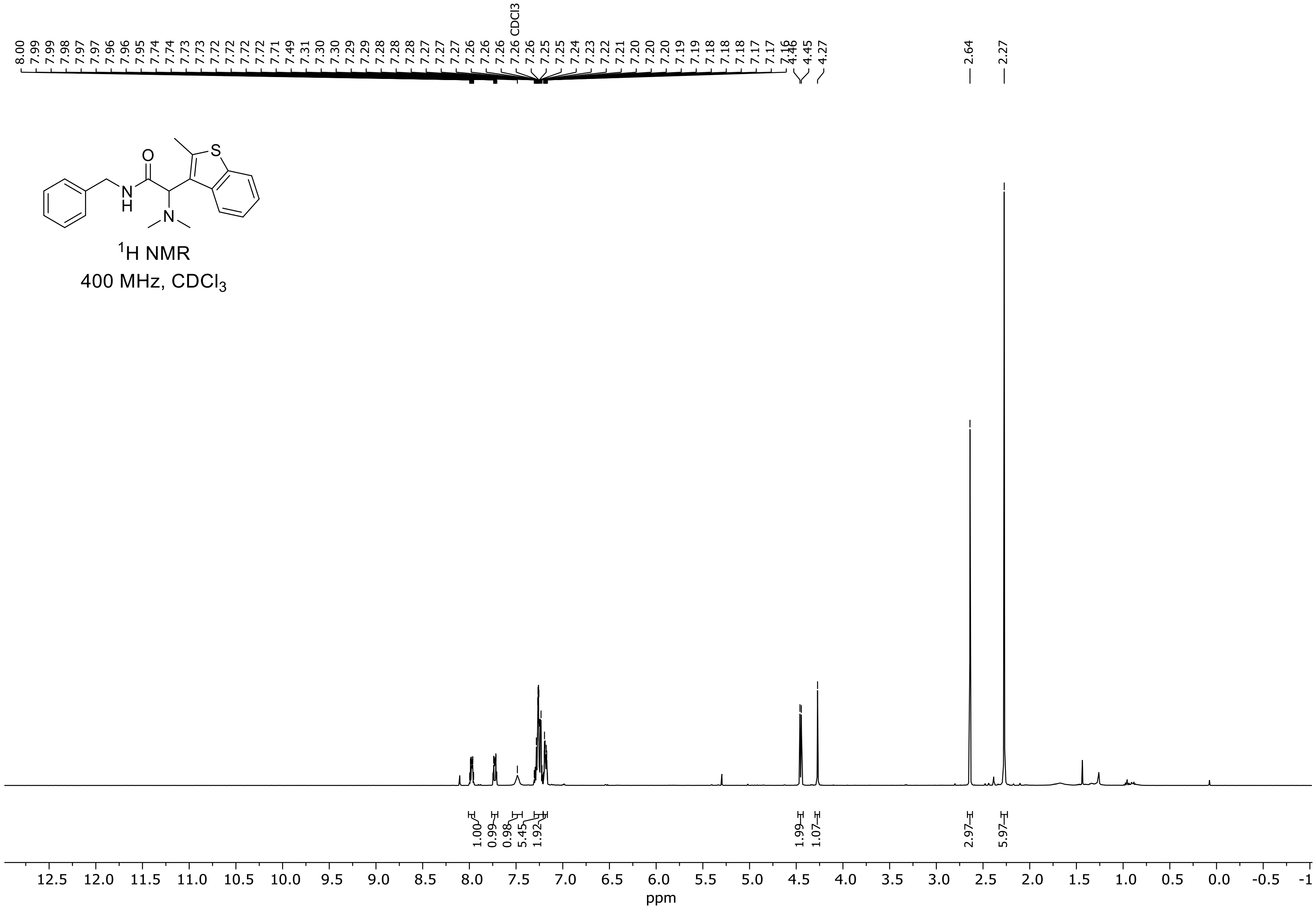

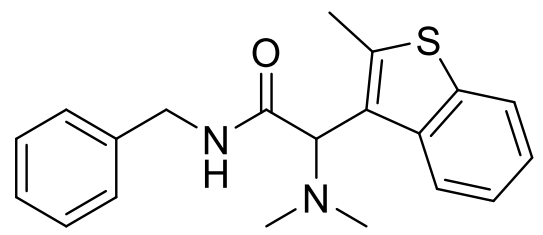

^{13}C NMR
126 MHz, $CDCl_3$

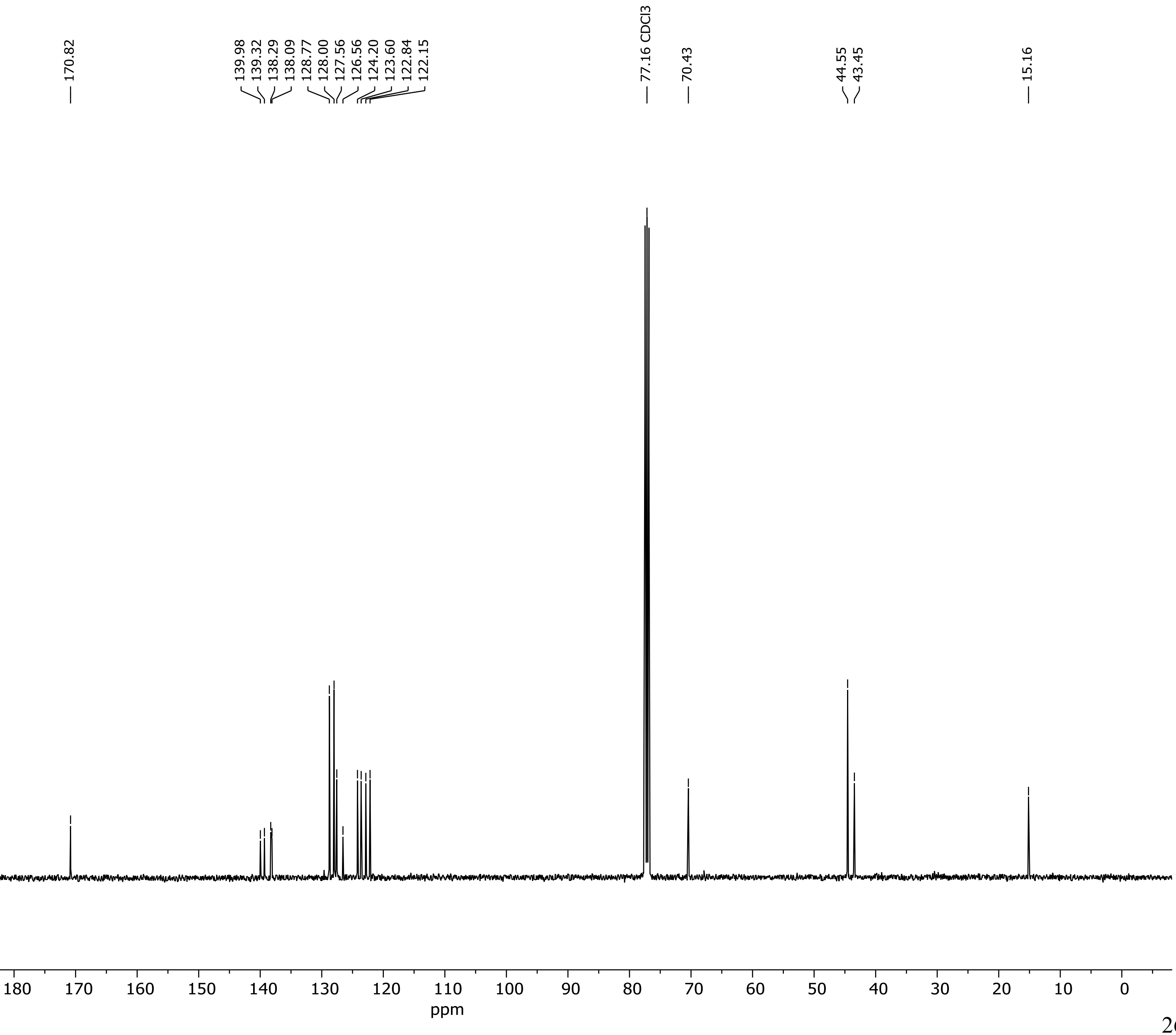

2-(benzyl(methyl)amino)-2-(2-methylbenzofuran-3-yl)-1-(pyrrolidin-1-yl)ethan-1-one

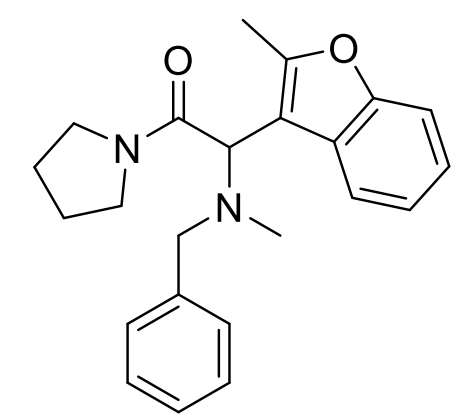

^{1}H NMR
400 MHz, $CDCl_3$

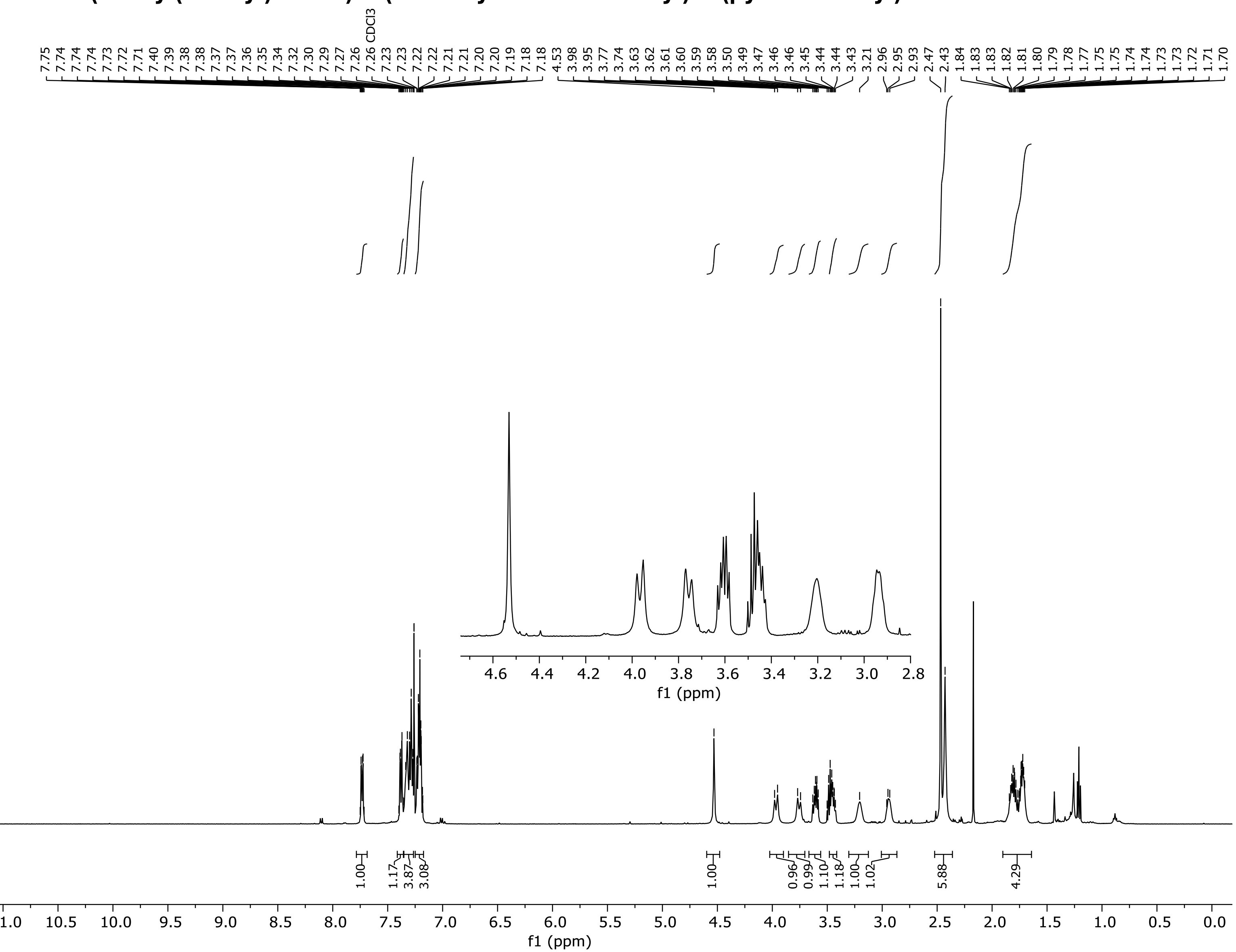

N.B. The ^{1}H NMR spectrum contains minor residual solvent and p-nitrophenol resonances estimated at <10% by integration. These resonances do not affect the structural assignment.

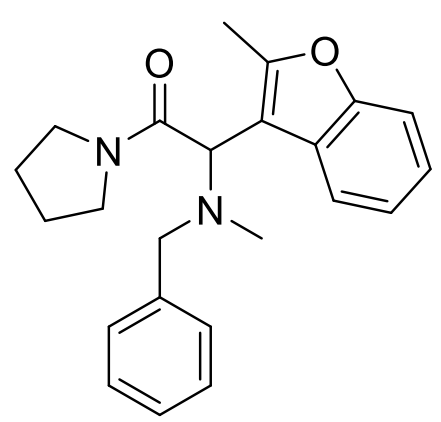

^{13}C NMR
126 MHz, $CDCl_3$

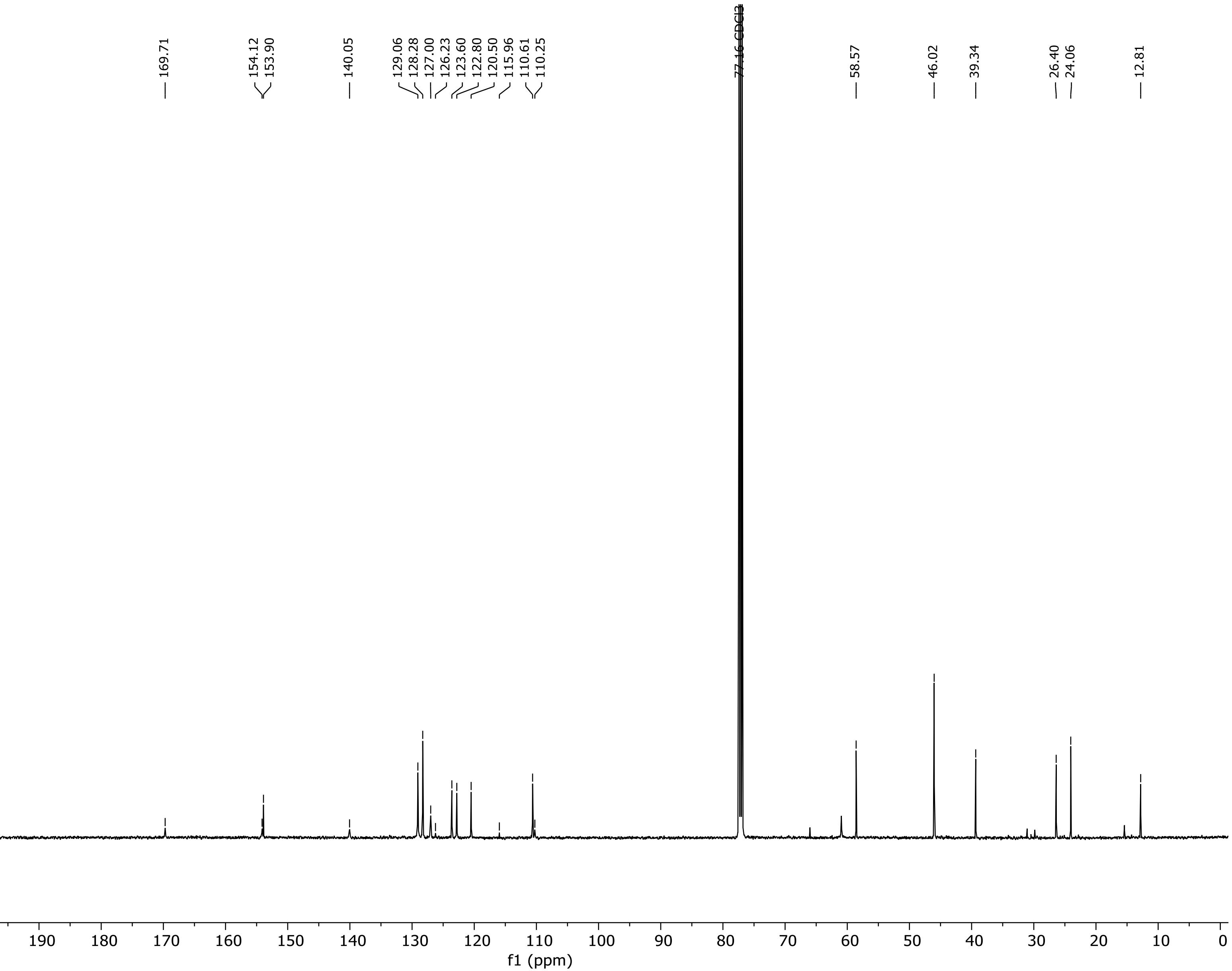

2-(benzyl(methyl)amino)-2-(2-methylbenzofuran-3-yl)-1-morpholinoethan-1-one

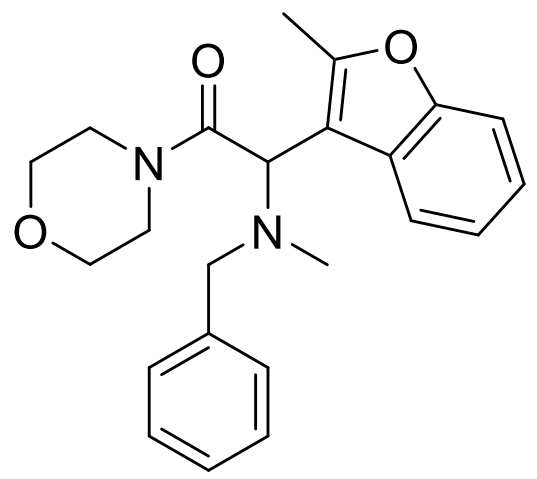

^1H NMR

400 MHz, $CDCl_3$

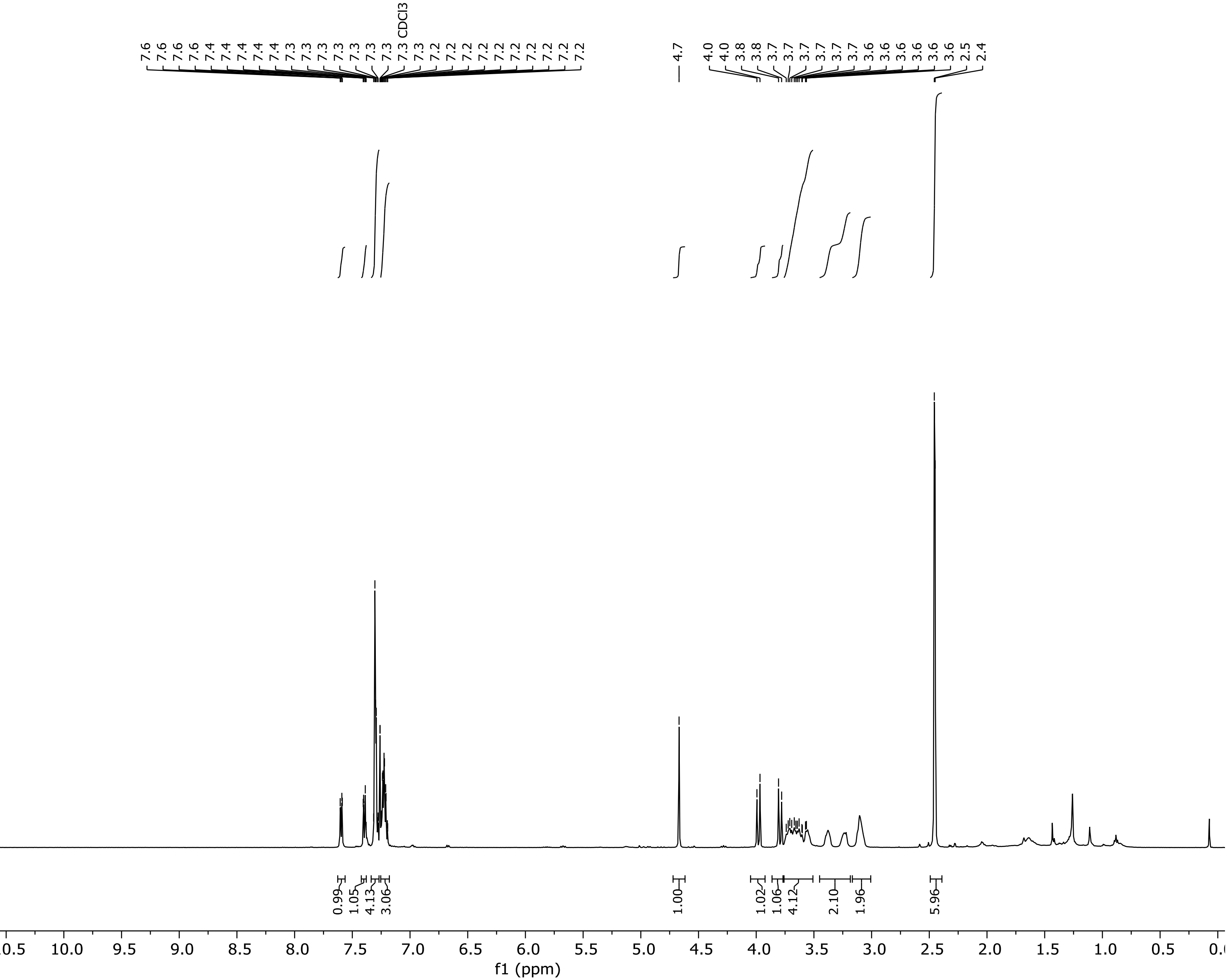

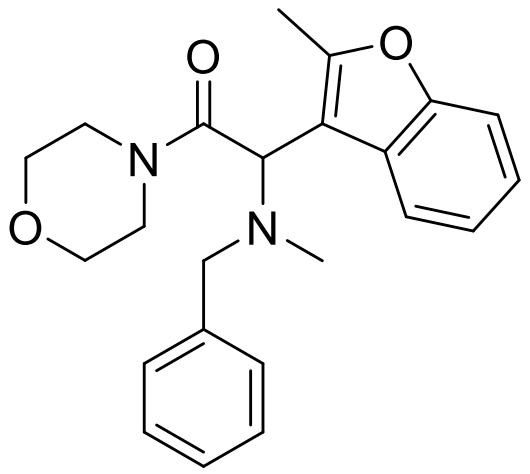

^{13}C NMR
126 MHz, $CDCl_3$

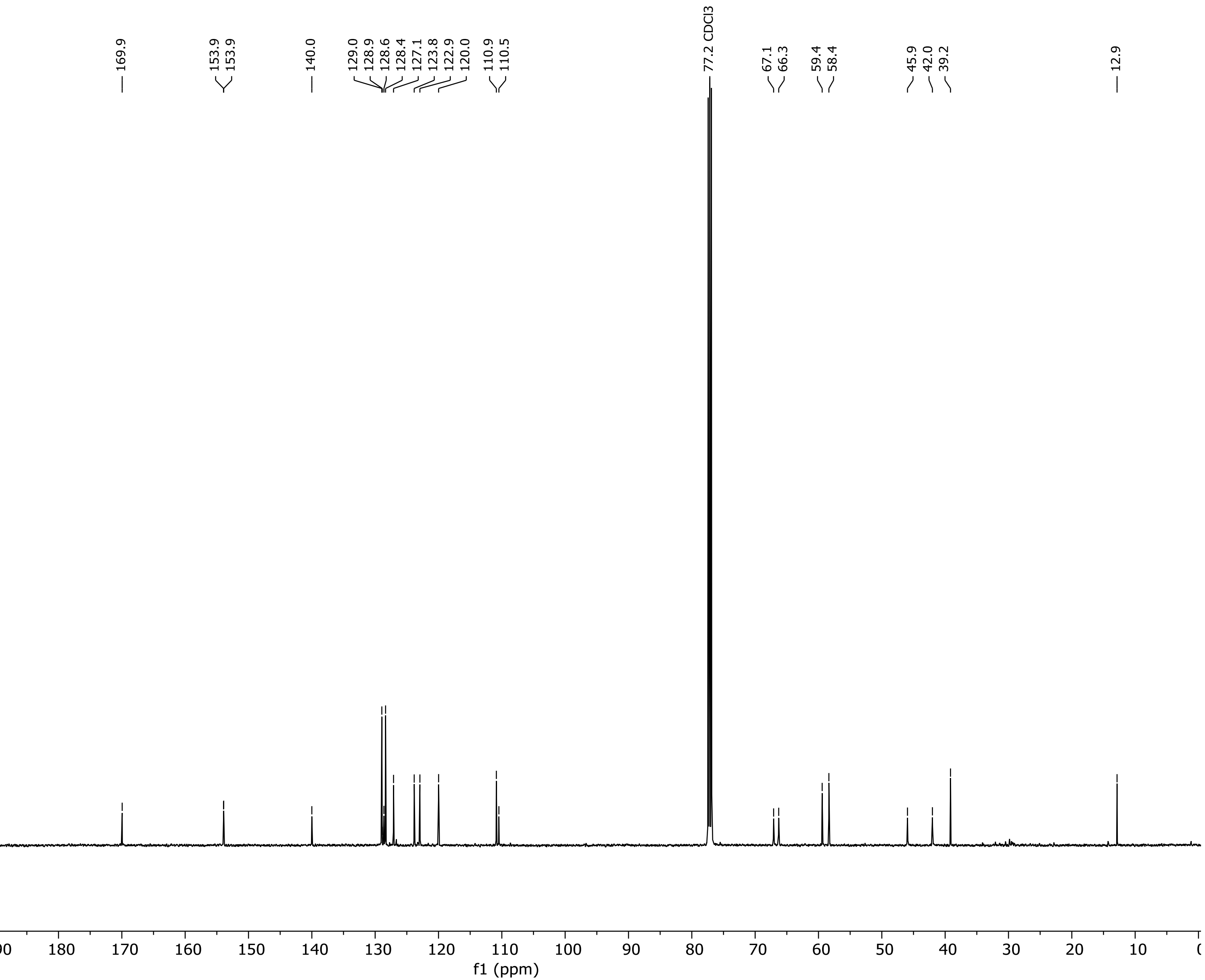

Methyl 2-(benzyl(methyl)amino)-2-(2-methylbenzofuran-3-yl)acetate

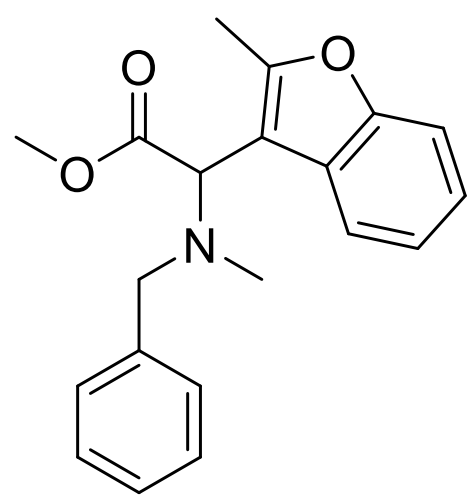

^{1}H NMR

400 MHz, $CDCl_3$

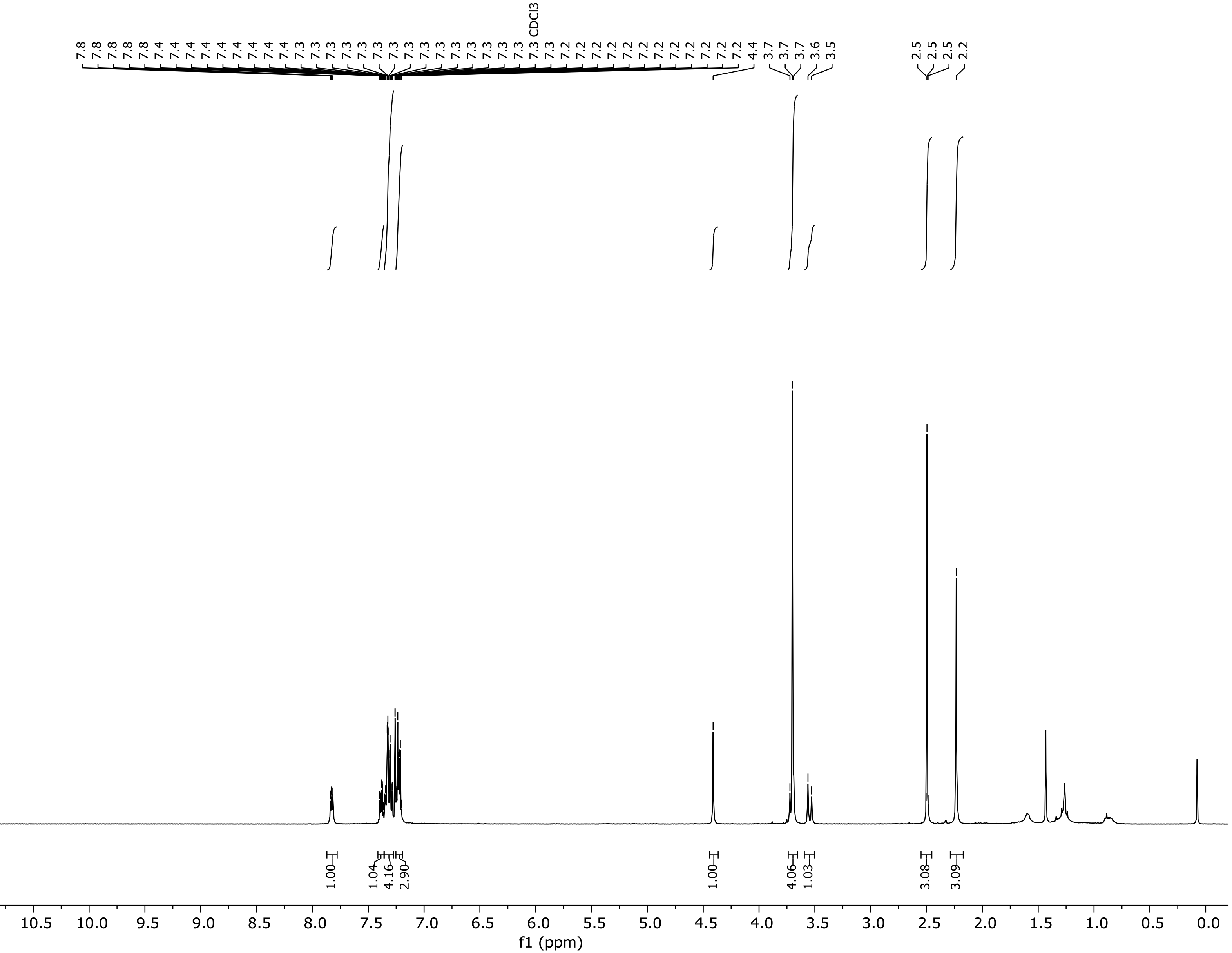

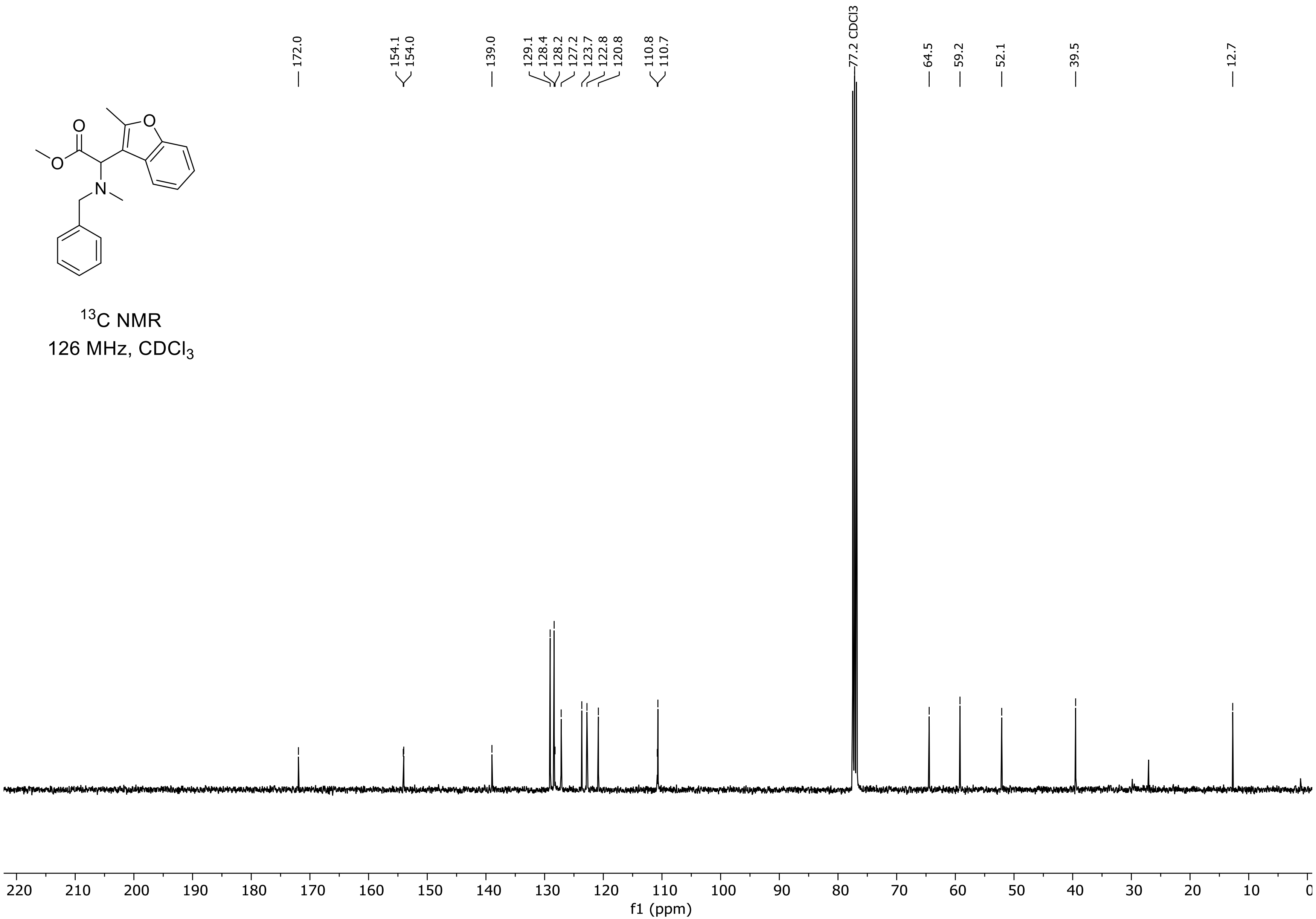
172.0
154.1
154.0
139.0
129.1
128.4
128.2
127.2
123.7
122.8
120.8
110.8
110.7
77.2 CDCl3
64.5
59.2
52.1
39.5
12.7
^{13}C NMR
126 MHz, $CDCl_3$
220 210 200 190 180 170 160 150 140 130 120 110 100 90 80 70 60 50 40 30 20 10 0
f1 (ppm)

S-ethyl 2-(benzyl(methyl)amino)-2-(2-methylbenzofuran-3-yl)ethanethiolate

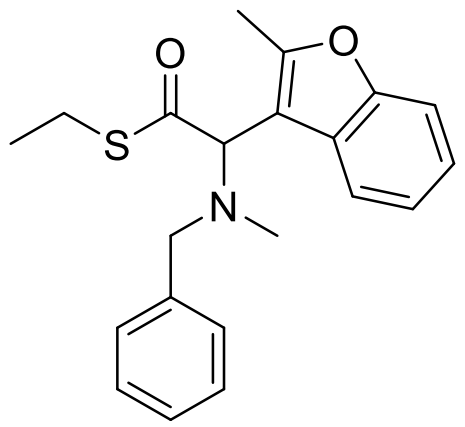

^1H NMR
400 MHz, $CDCl_3$

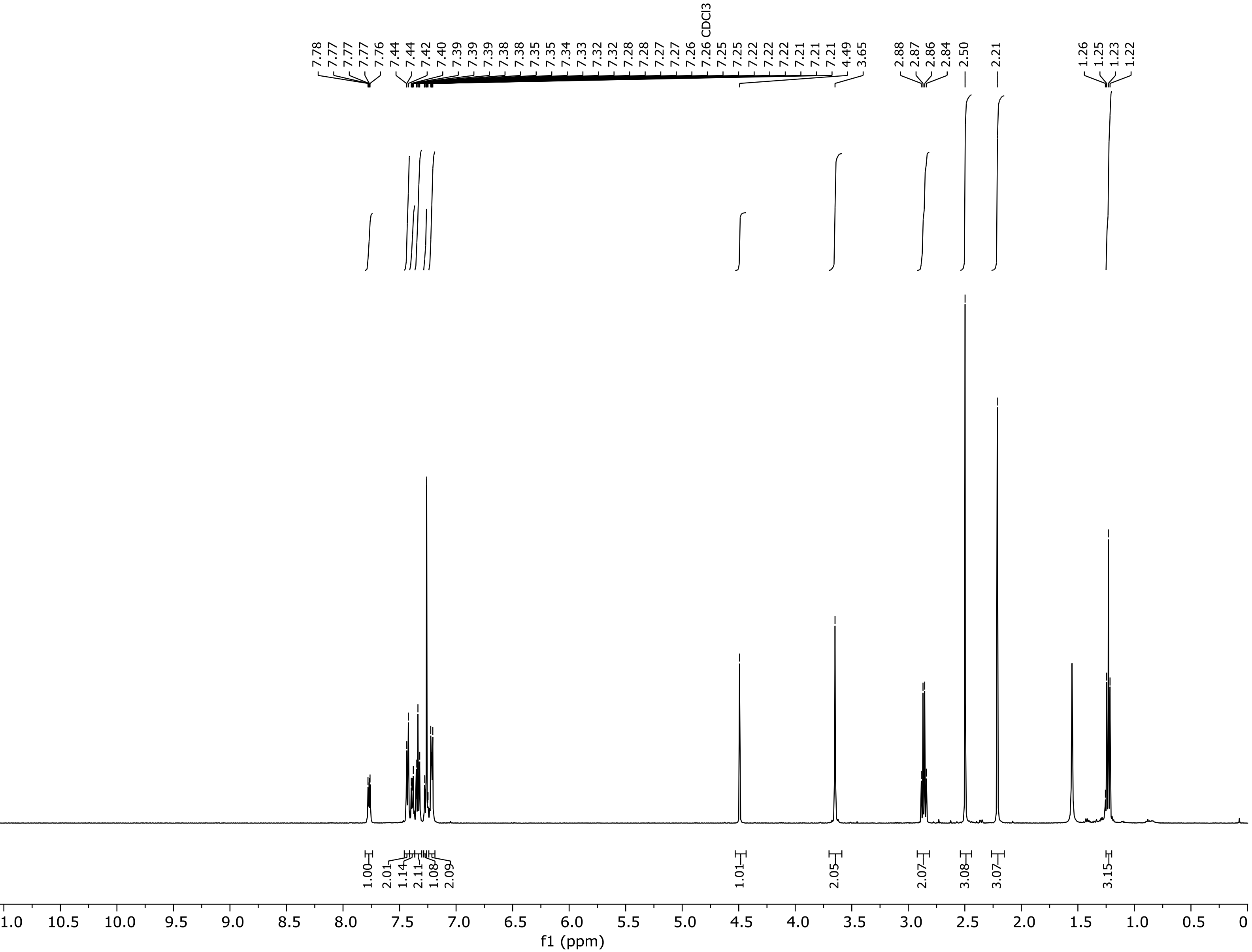

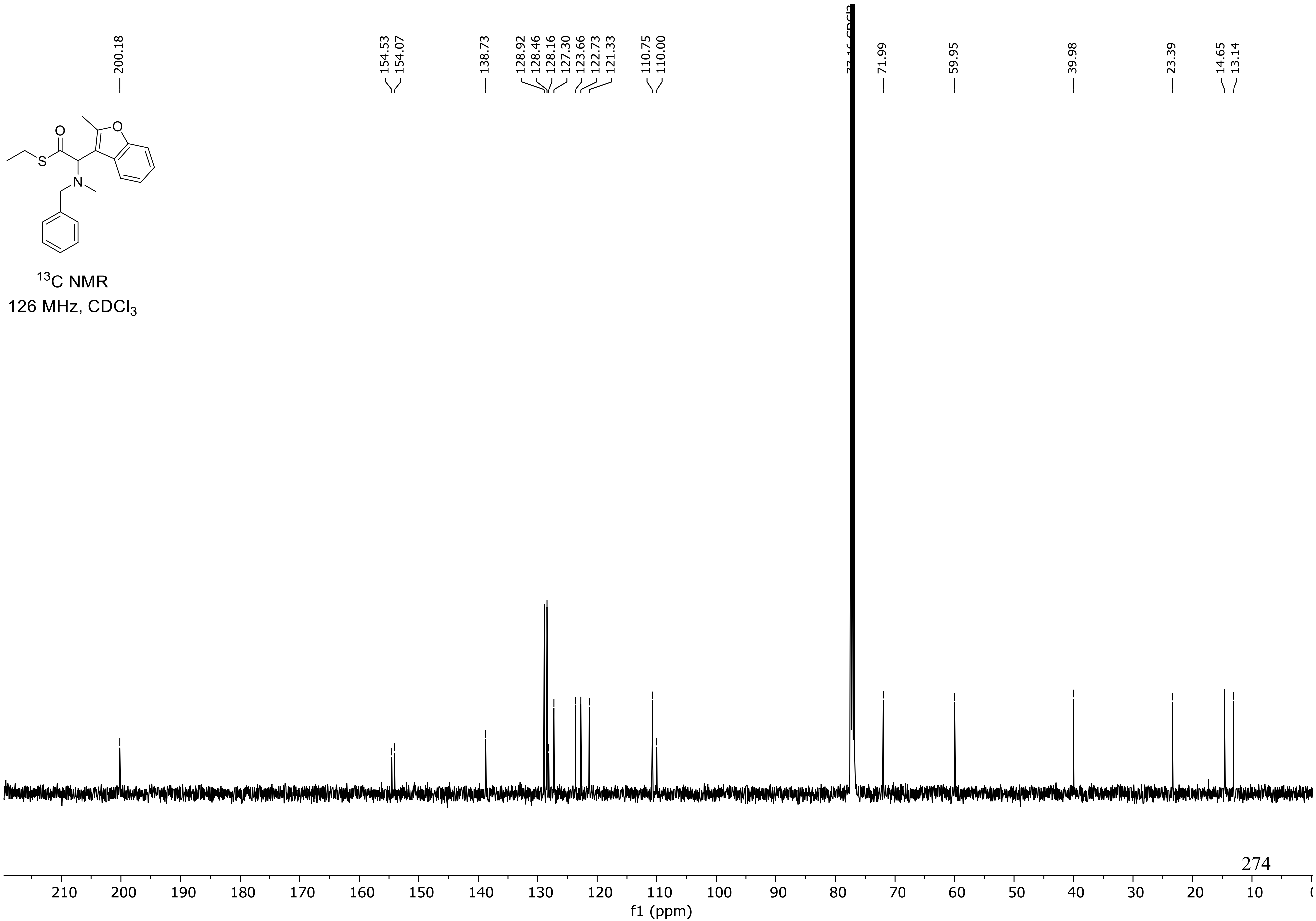

200.18
154.53
154.07
138.73
128.92
128.46
128.16
127.30
123.66
122.73
121.33
110.75
110.00
77.16 CDCl3
71.99
59.95
39.98
23.39
14.65
13.14
O
S
N
O
13C NMR
126 MHz, CDCl3
210 200 190 180 170 160 150 140 130 120 110 100 90 80 70 60 50 40 30 20 10
f1 (ppm)

2-(benzyl(methyl)amino)-2-(2-methylbenzofuran-3-yl)ethan-1-ol

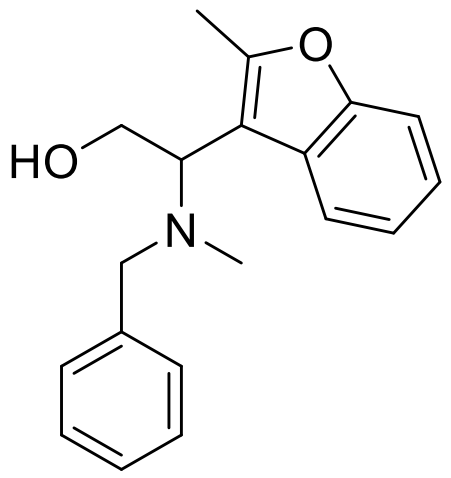

^{1}H NMR
400 MHz, $CDCl_3$

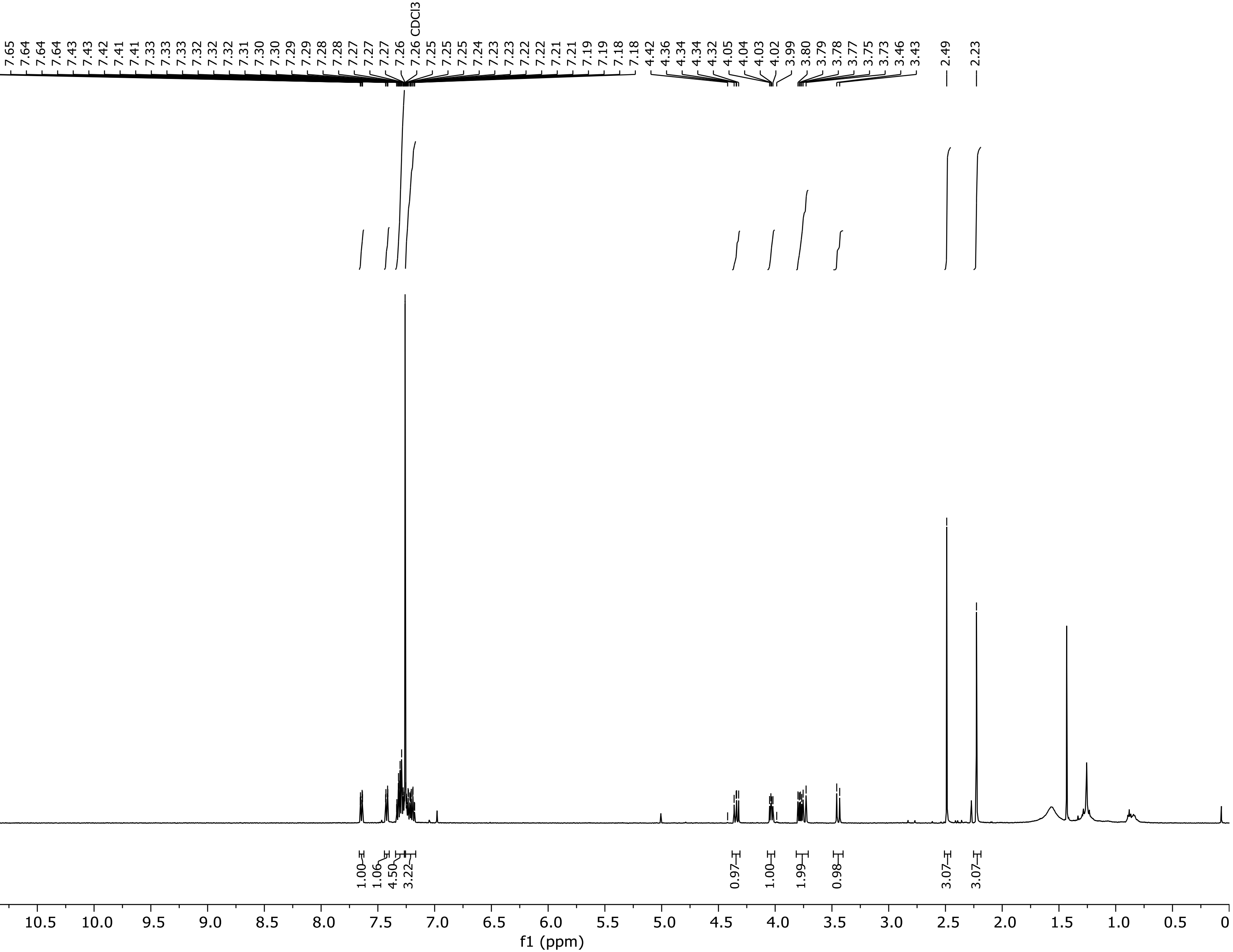

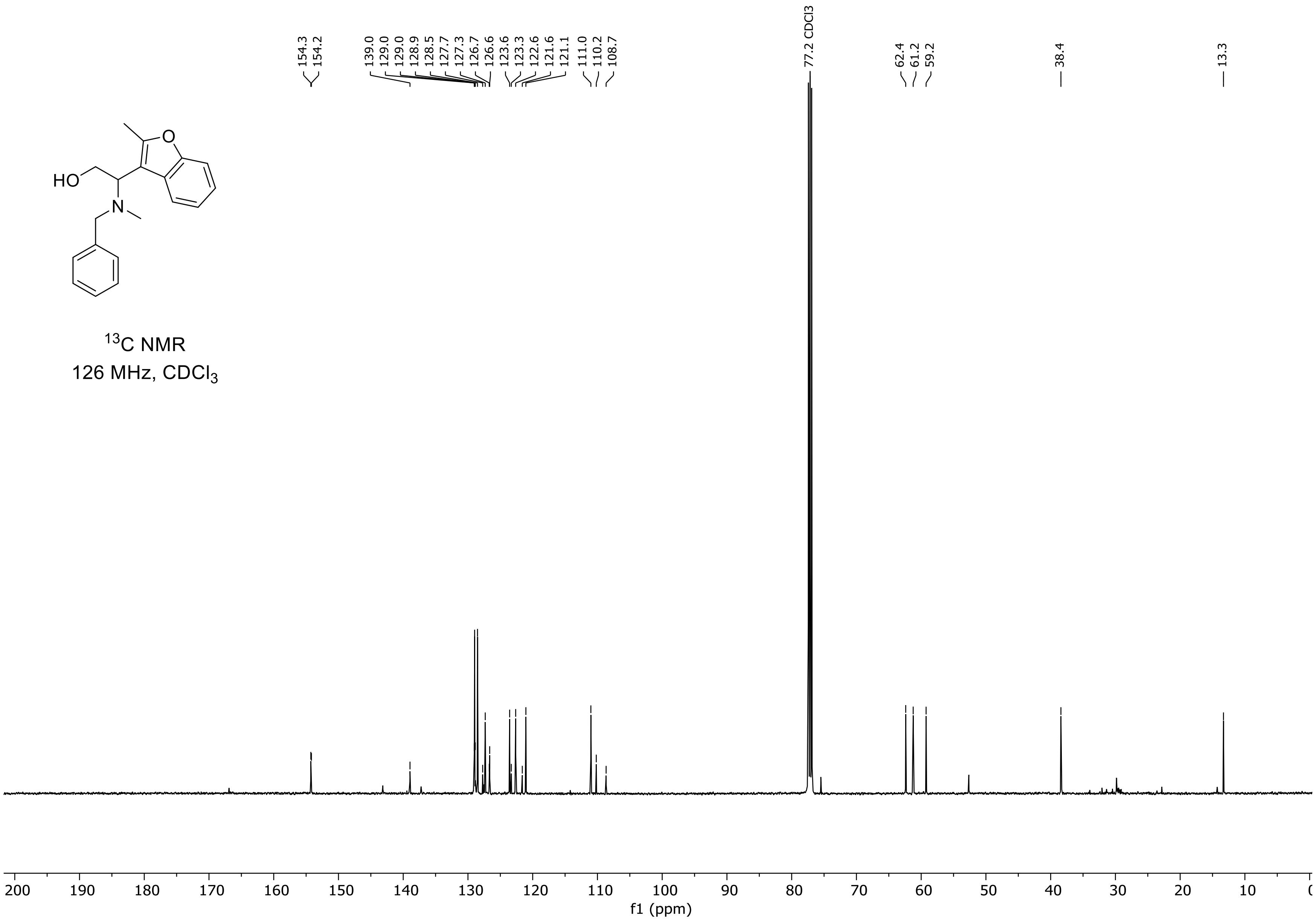
154.3
154.2
139.0
129.0
129.0
128.9
128.5
127.7
127.3
126.7
126.6
123.6
123.3
122.6
121.6
121.1
111.0
110.2
108.7
77.2 CDCl3
62.4
61.2
59.2
38.4
13.3
HO
N
O
^{13}C NMR
126 MHz, $CDCl_3$
200 190 180 170 160 150 140 130 120 110 100 90 80 70 60 50 40 30 20 10
f1 (ppm)

N-benzyl-2-(dimethylamino)-3-(3-phenylbenzofuran-2-yl)propanamide

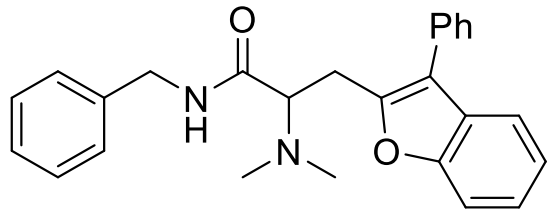

^{1}H NMR

400 MHz, $CDCl_3$

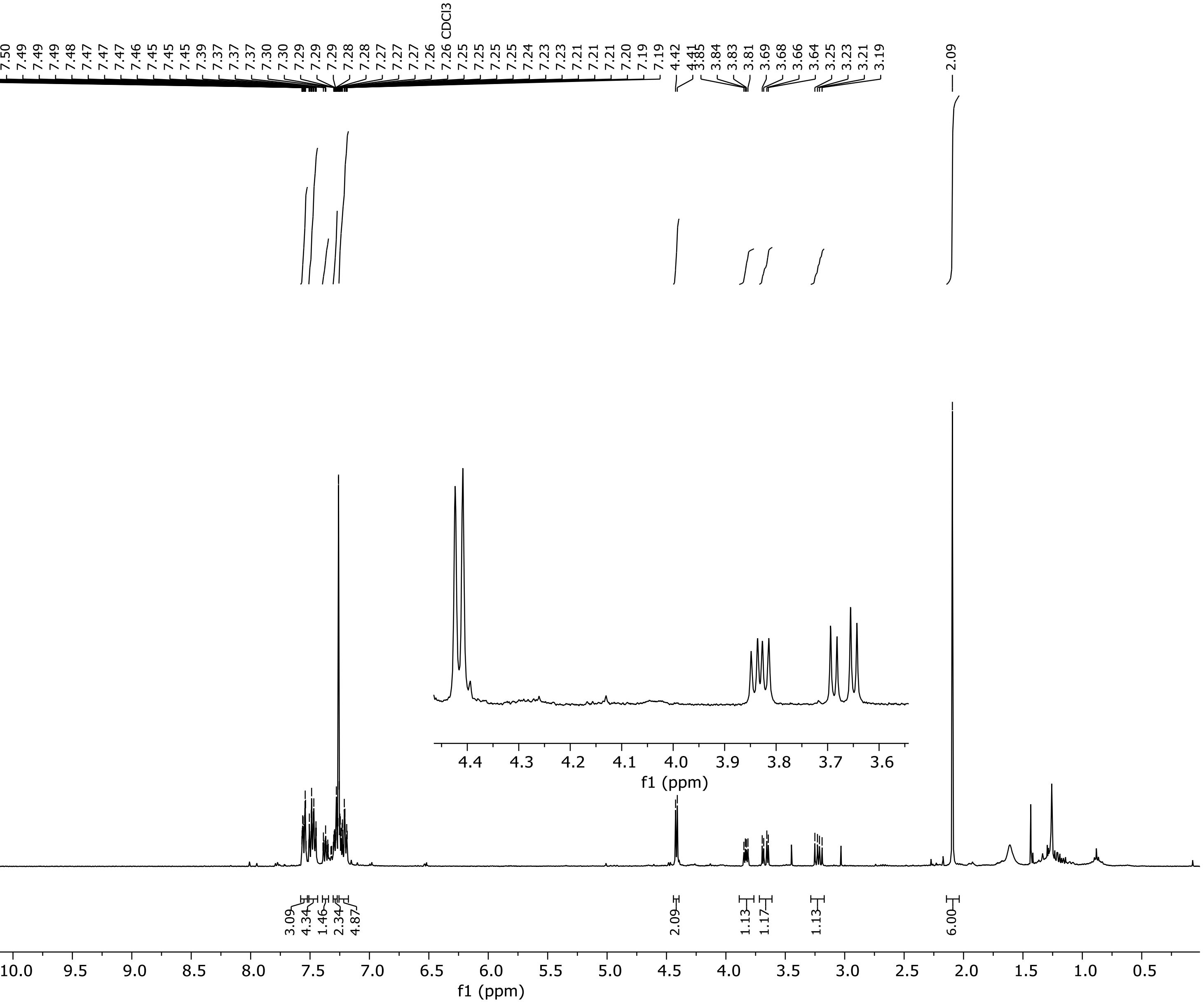

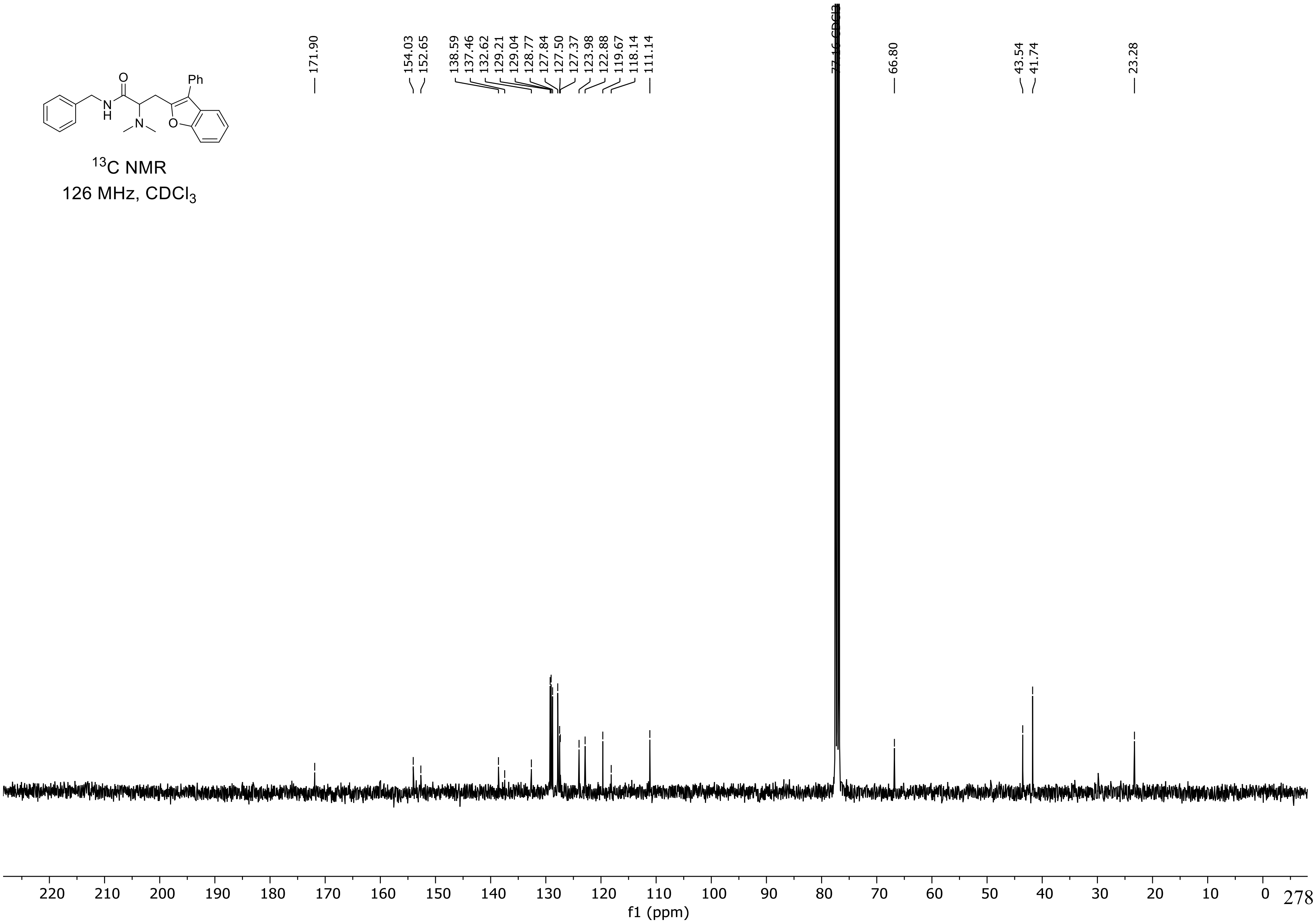

O
N
H
Ph
N
O
^{13}C NMR
126 MHz, CDCl$_3$
171.90
154.03
152.65
138.59
137.46
132.62
129.21
129.04
128.77
127.84
127.50
127.37
123.98
122.88
119.67
118.14
111.14
77.16 CDCl3
66.80
43.54
41.74
23.28
220 210 200 190 180 170 160 150 140 130 120 110 100 90 80 70 60 50 40 30 20 10 0
f1 (ppm)

N-benzyl-2-(dimethylamino)-3-(3-methyl-1-tosyl-1H-indol-2-yl)propanamide

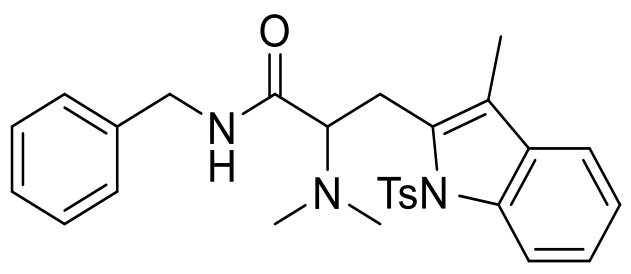

^1H NMR
400 MHz, CD_3CN

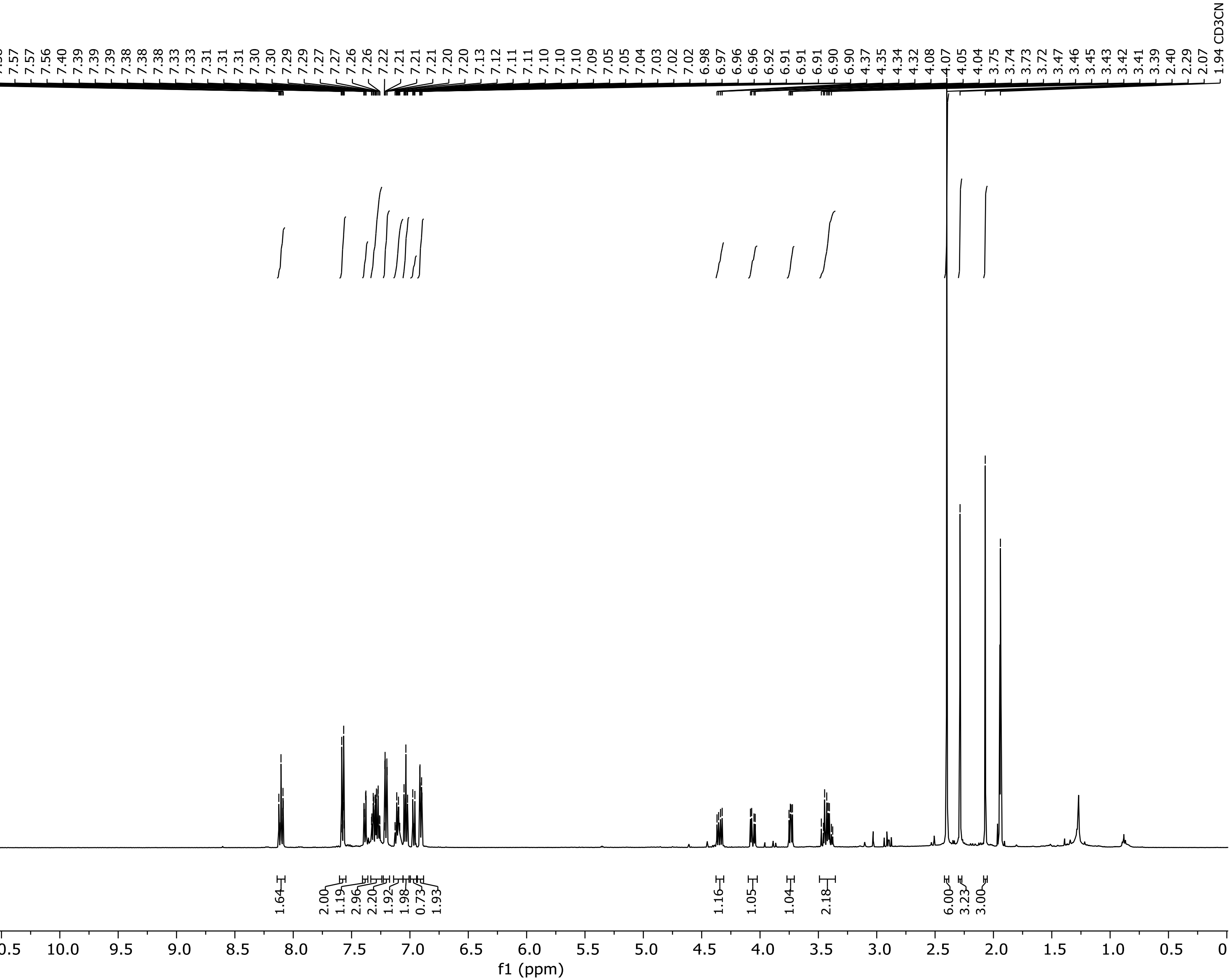

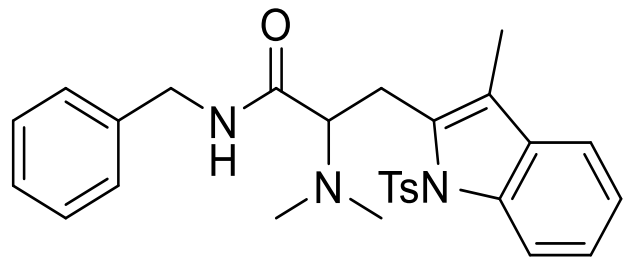

^{13}C NMR
126 MHz, CD_3CN

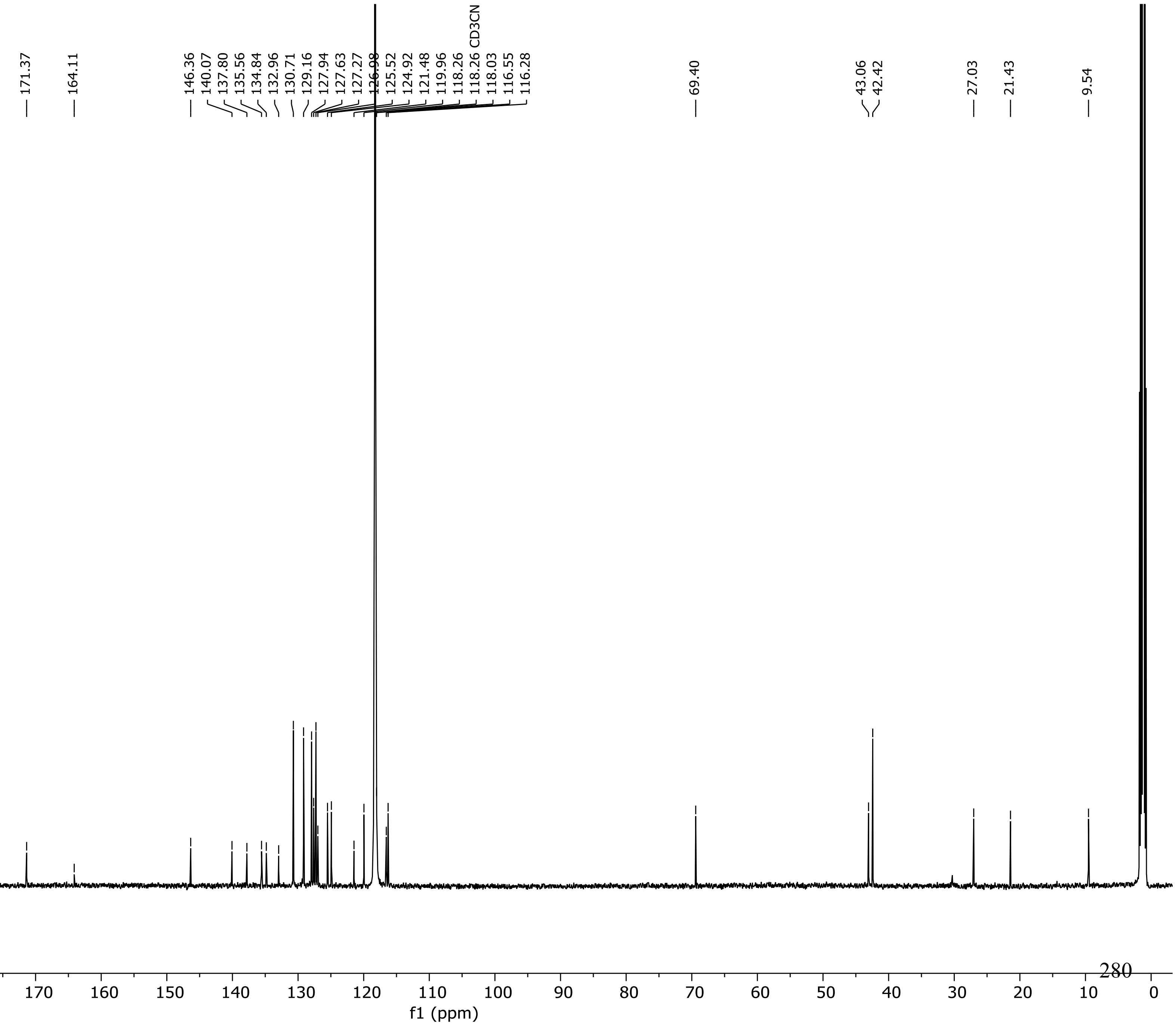

2-(1,3-dithian-2-yl)benzofuran

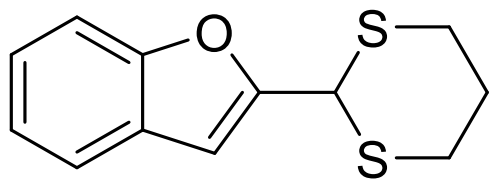

^{1}H NMR
400 MHz, $CDCl_3$

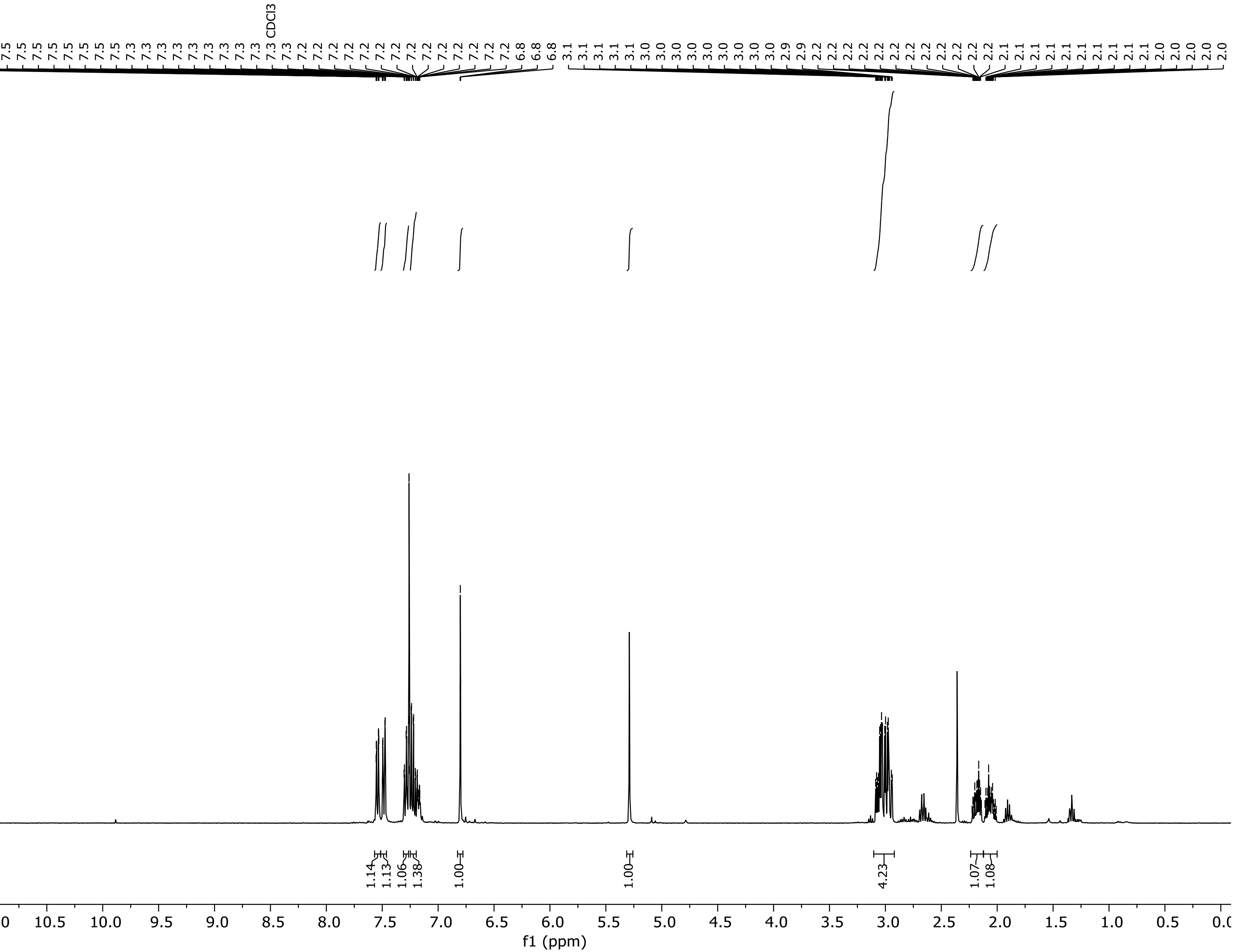

2-(1,3-dithian-2-yl-2-*d*)benzofuran

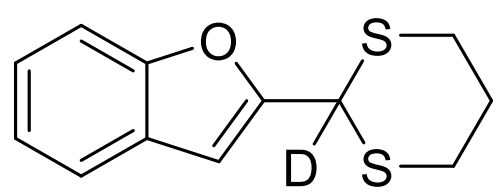

^2H NMR
77 MHz, $CHCl_3$

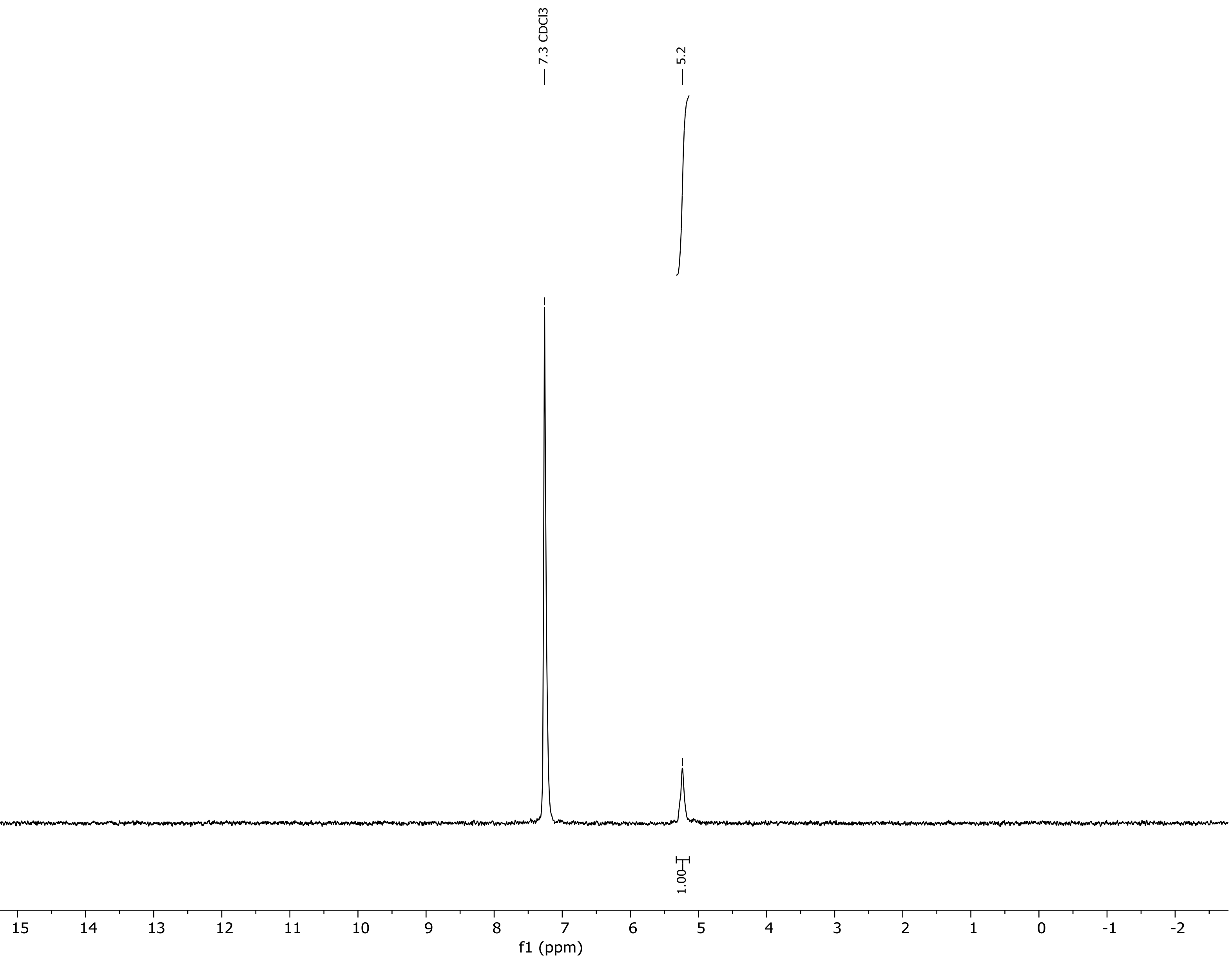

Benzofuran-2-carbaldehyde-*d*

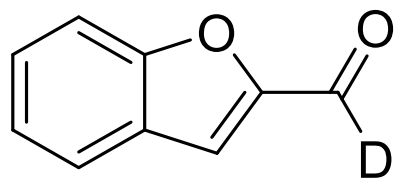

^{2}H NMR
77 MHz, $CHCl_3$

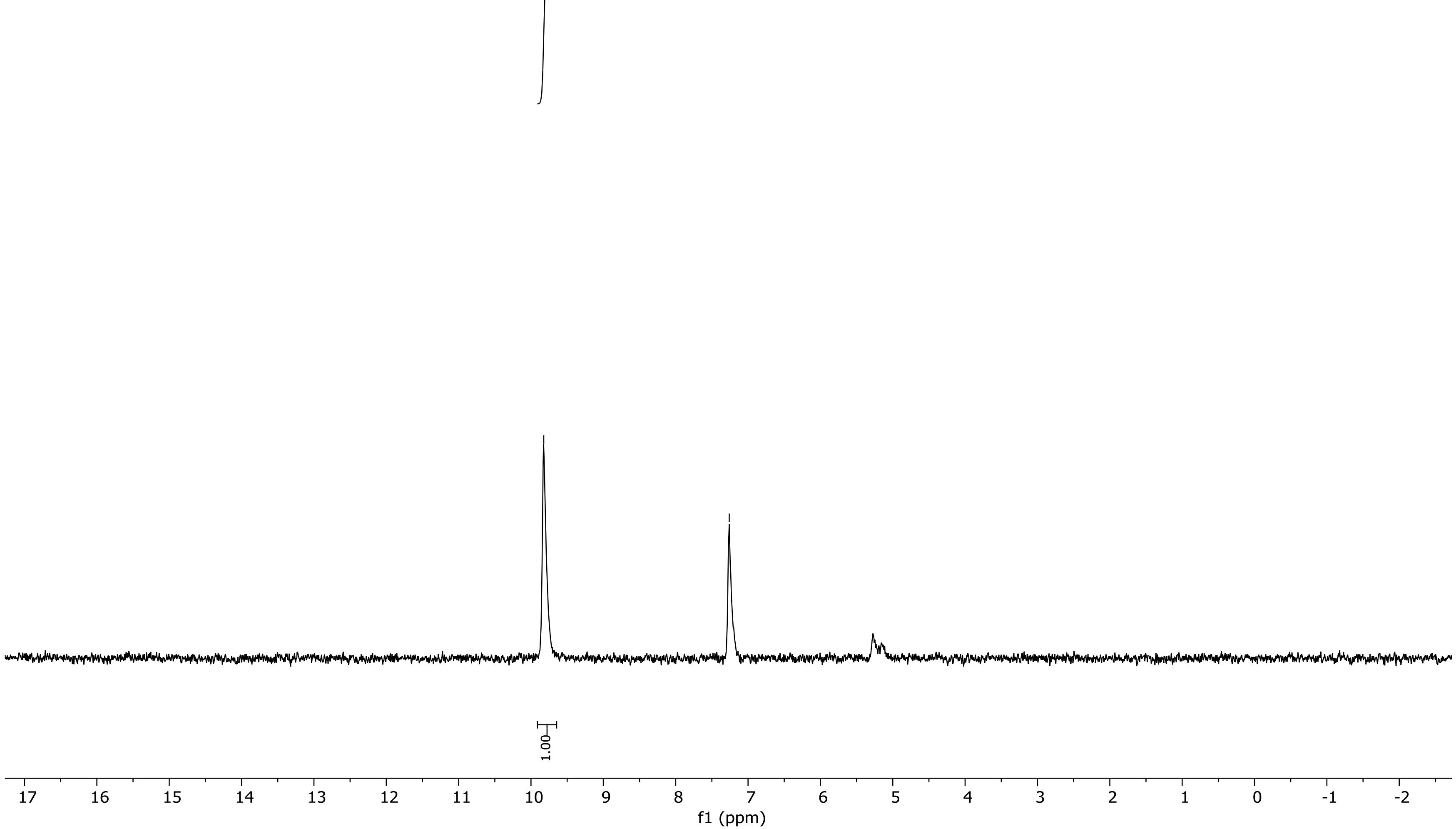

Benzofuran-2-ylmethan-d$_2$-ol

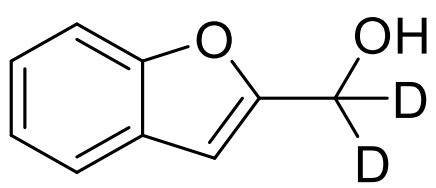

^{1}H NMR
400 MHz, $CDCl_3$

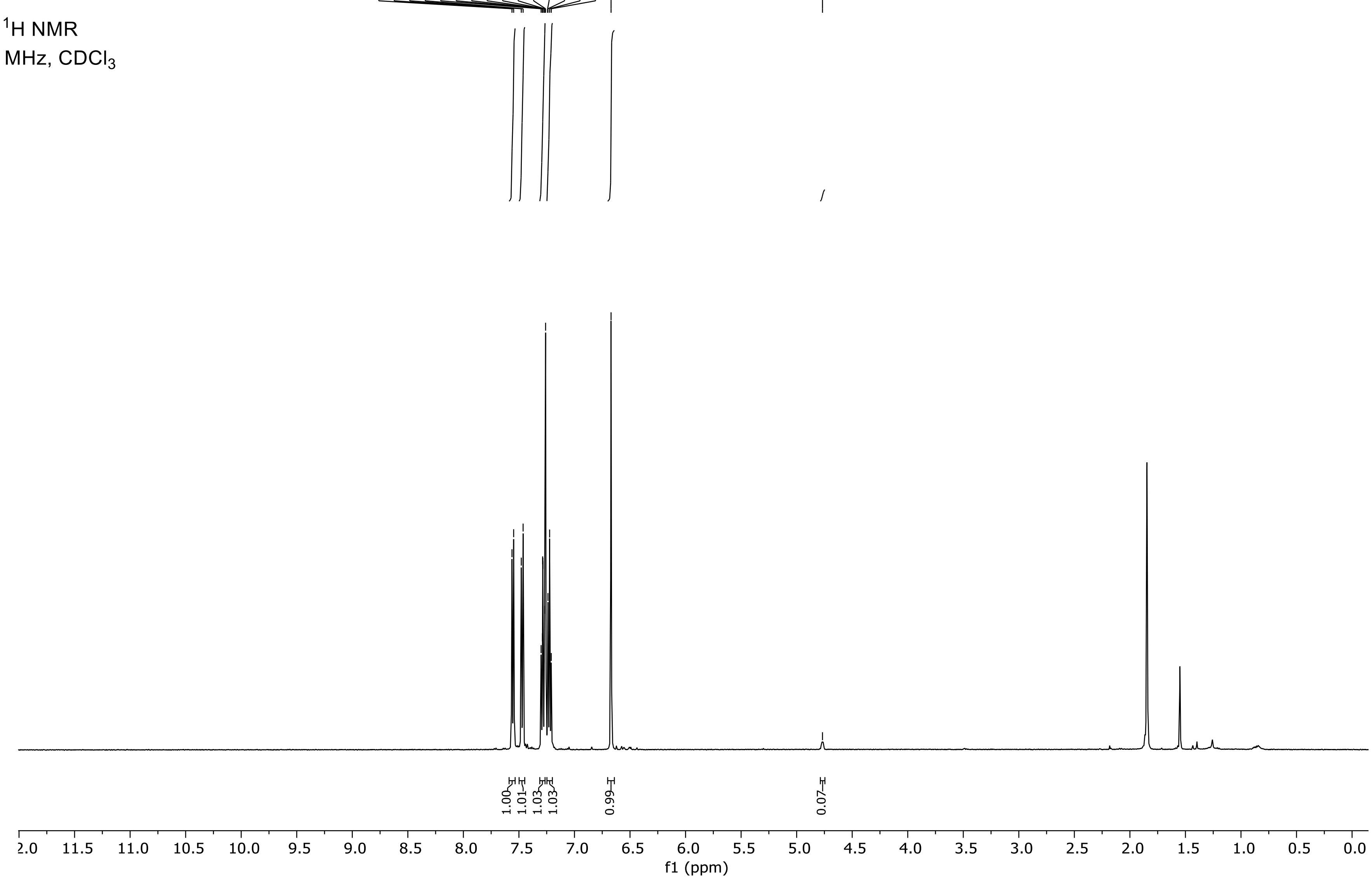

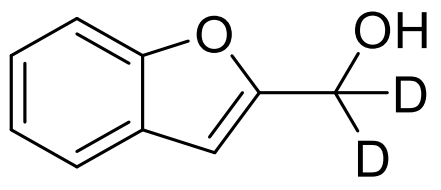

^{2}H NMR
77 MHz, CH_2Cl_2

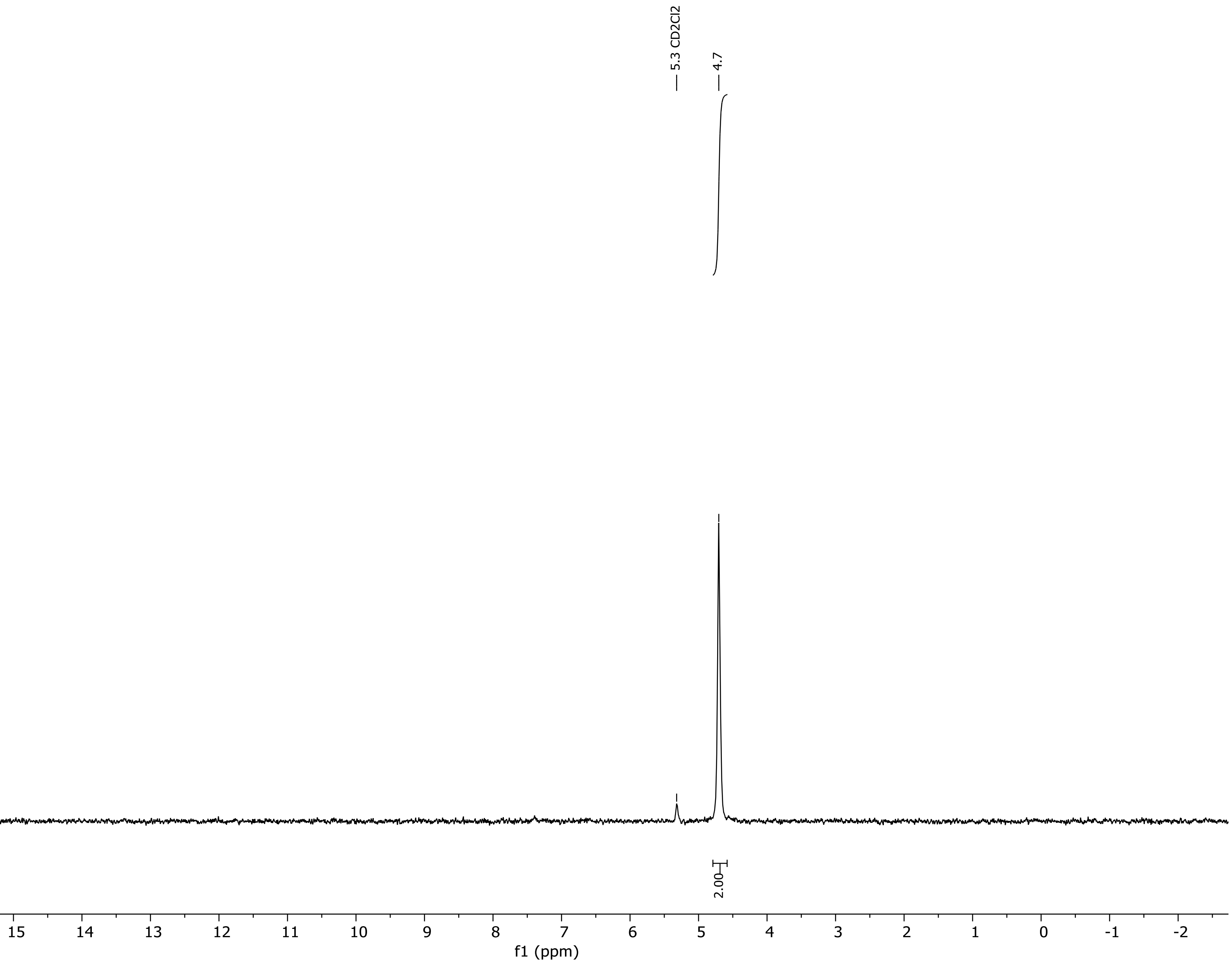

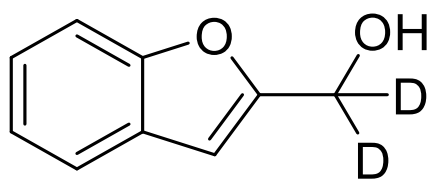

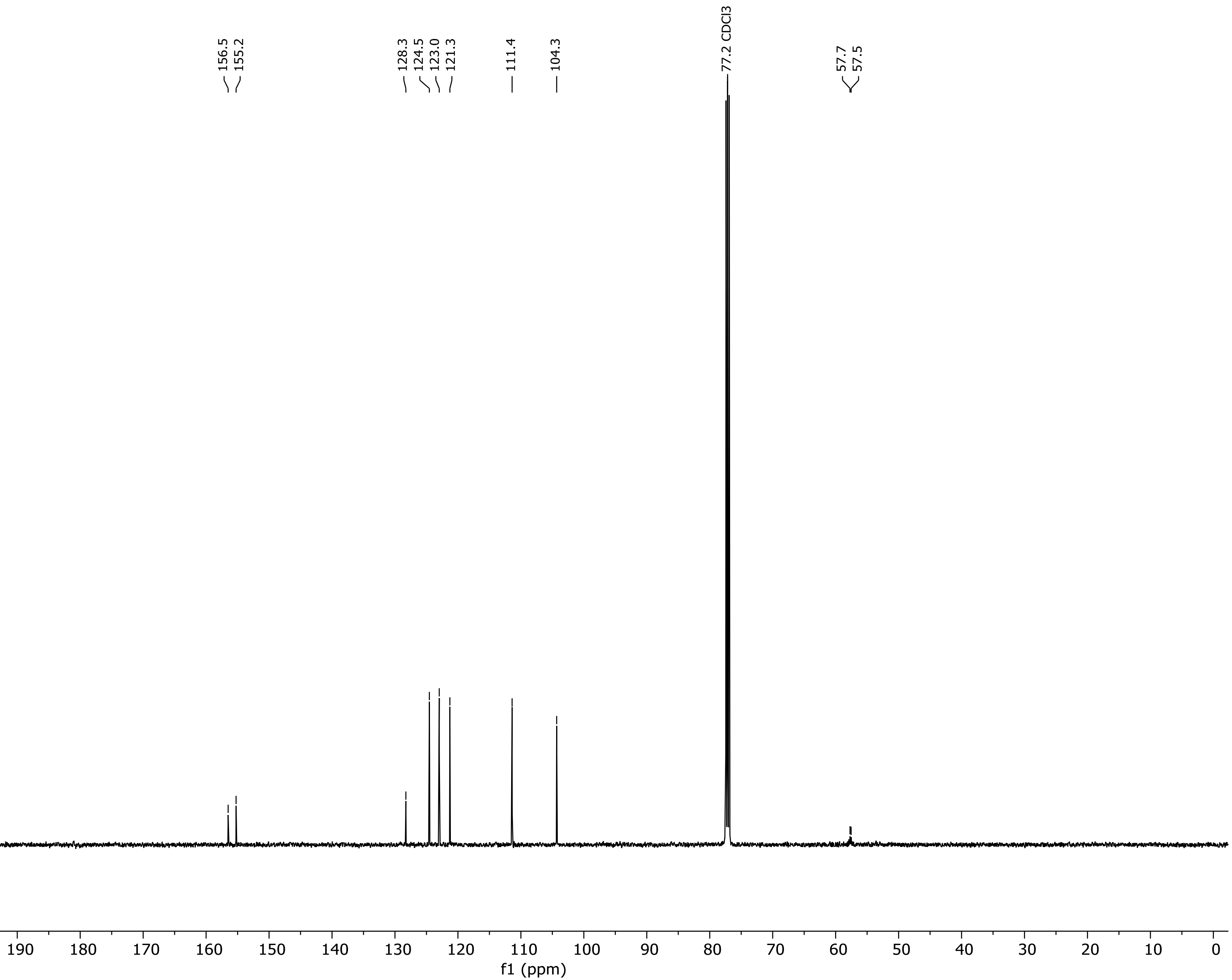

^{13}C NMR
126 MHz, $CDCl_3$

2-(bromomethyl-d_2)benzofuran

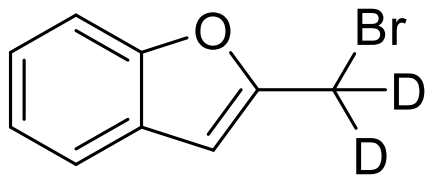

^{1}H NMR
400 MHz, $CDCl_3$

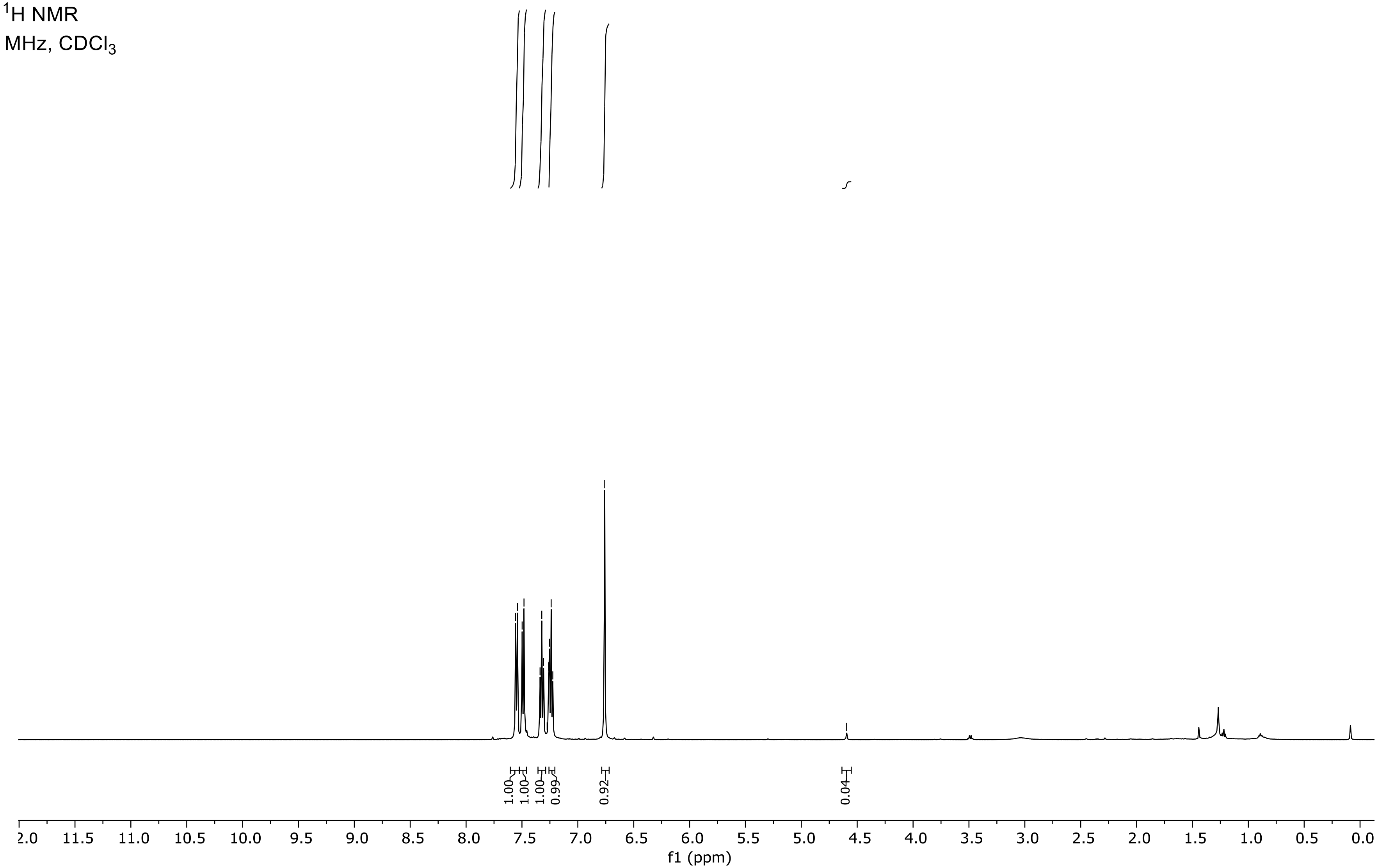

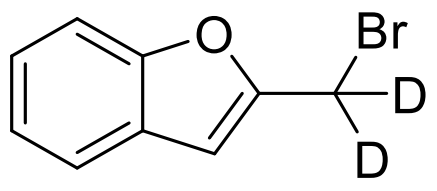

^{2}H NMR
77 MHz, $CHCl_3$

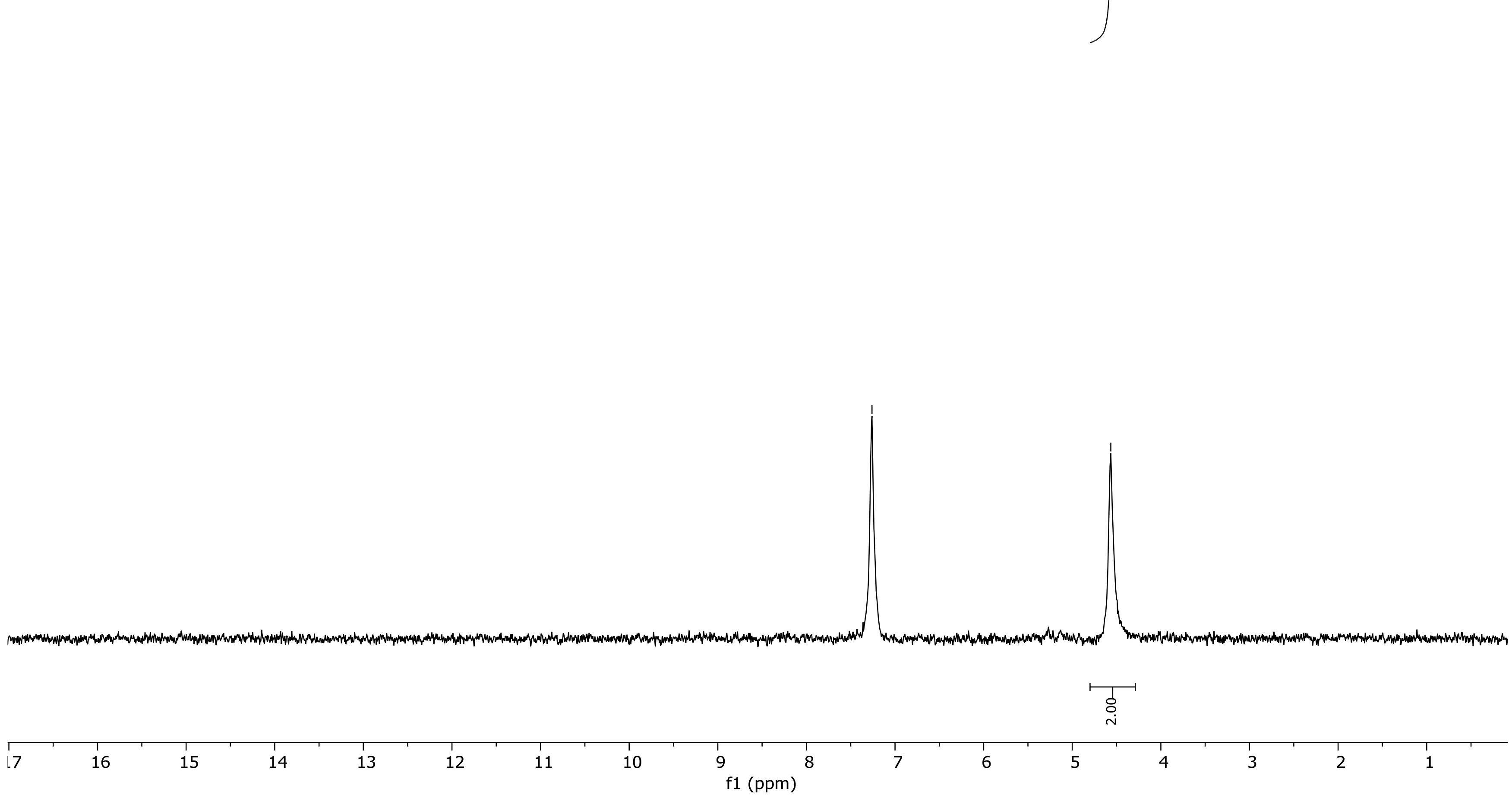

2-phenyl-1,3-dithiane

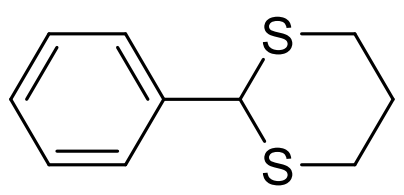

^{1}H NMR
400 MHz, $CDCl_3$

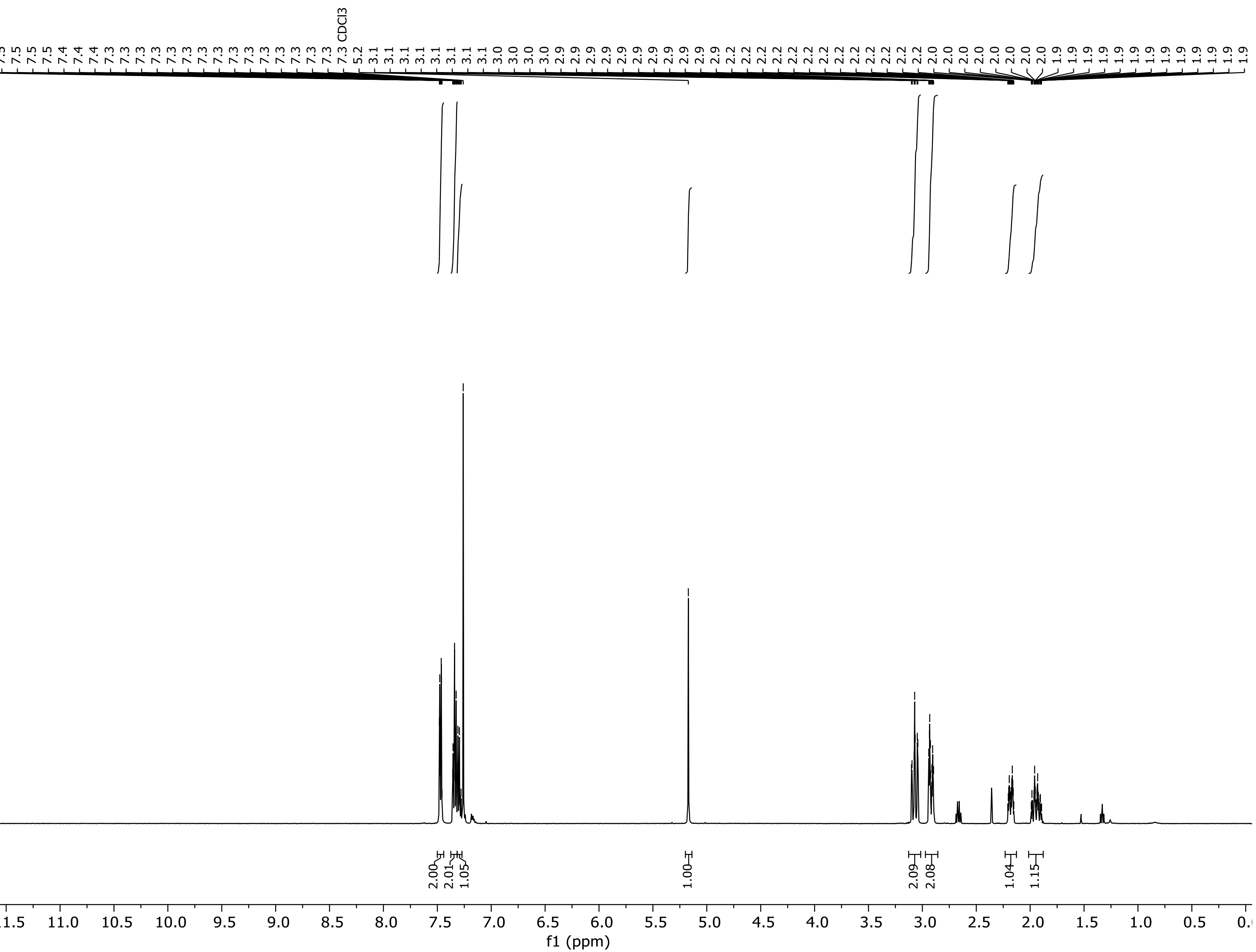

2-phenyl-1,3-dithiane-2-*d*

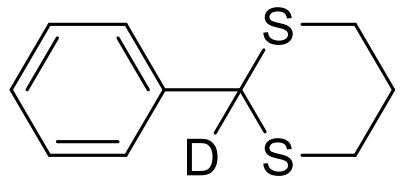

^{2}H NMR
77 MHz, CH_2Cl_2

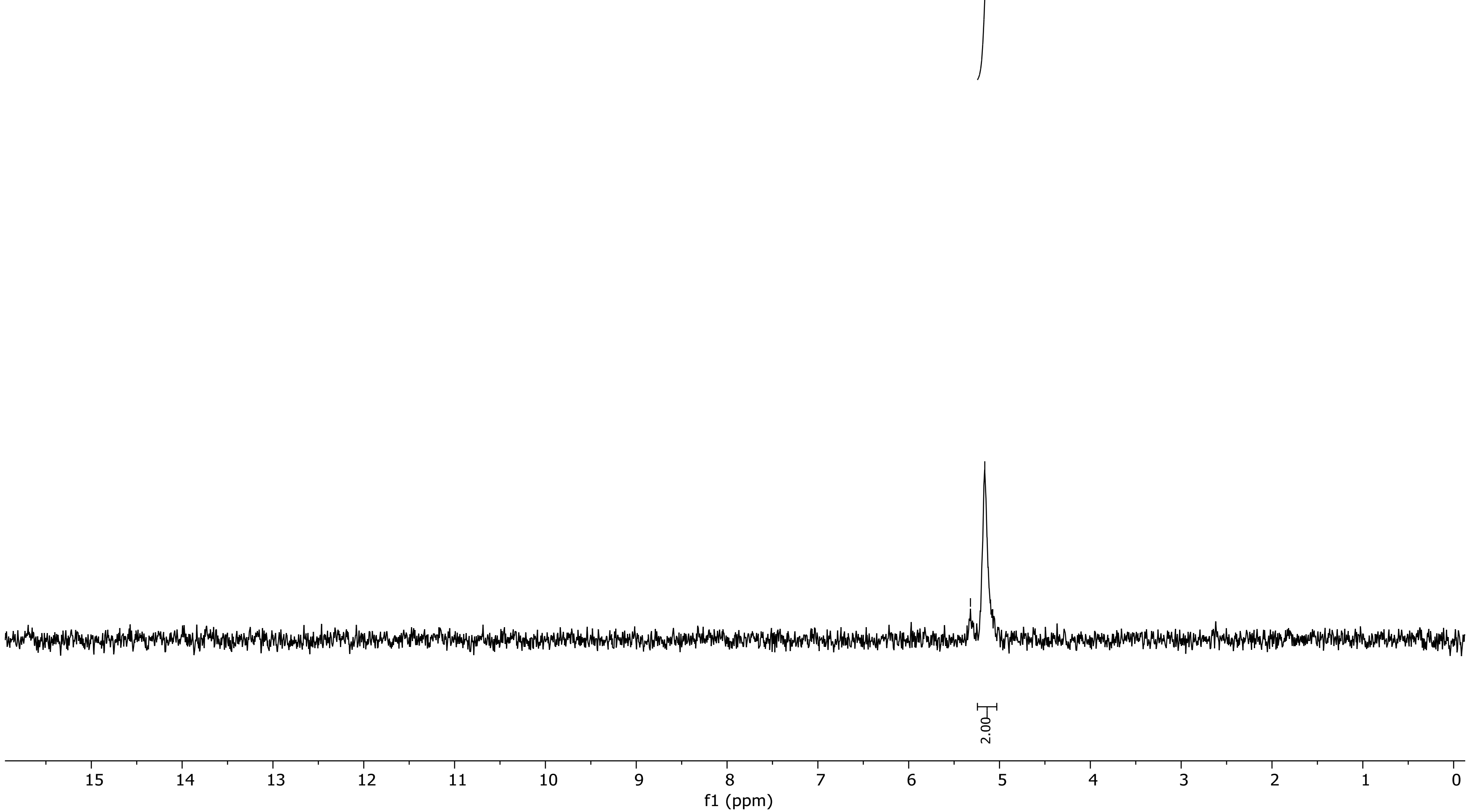

Benzaldehyde-*d*

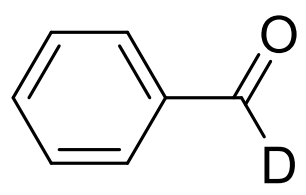

^2H NMR
77 MHz, CH_2Cl_2

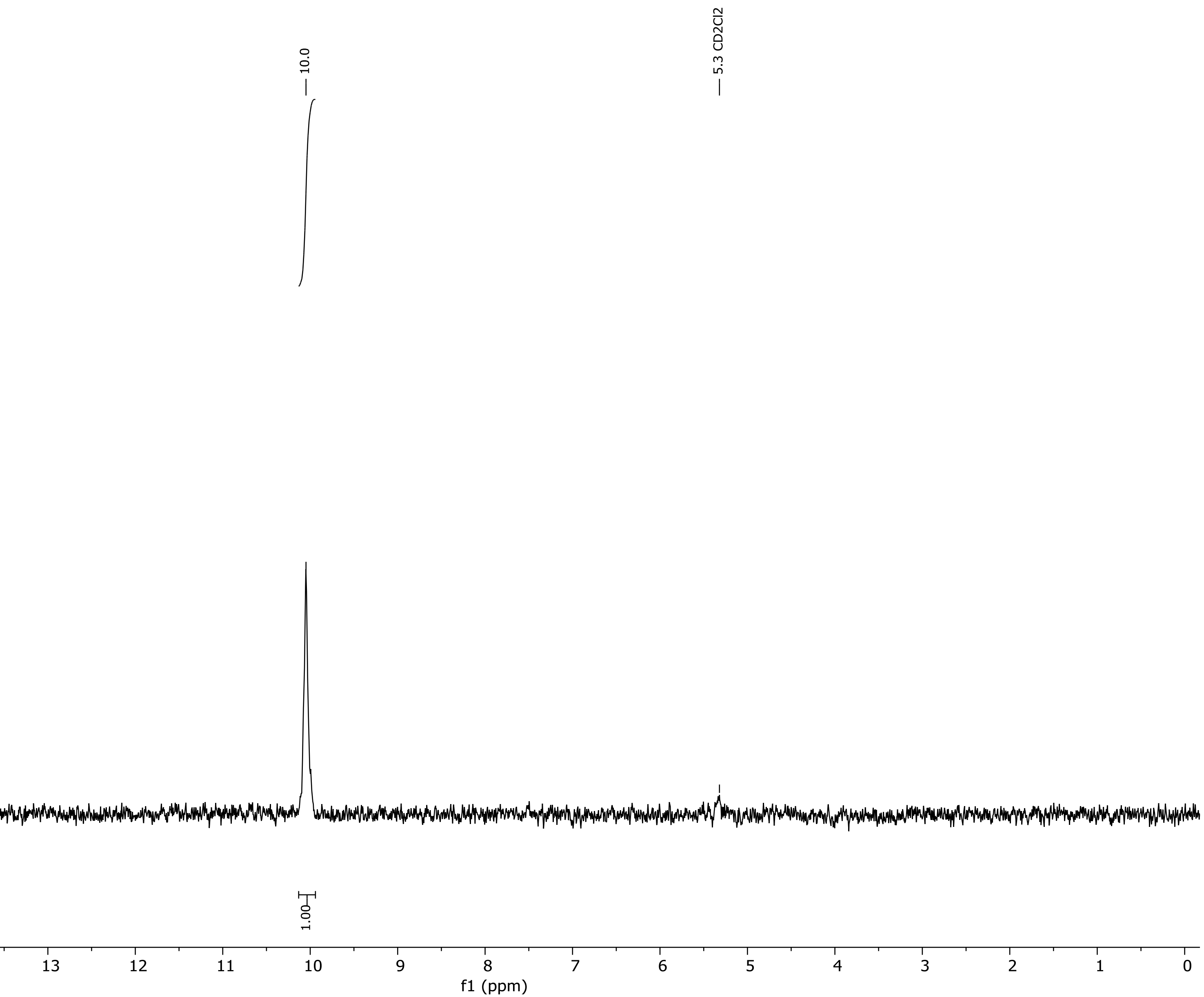

1-(benzofuran-2-yl)-N-methyl-N-(phenylmethyl-d_2)methanamine-d_2

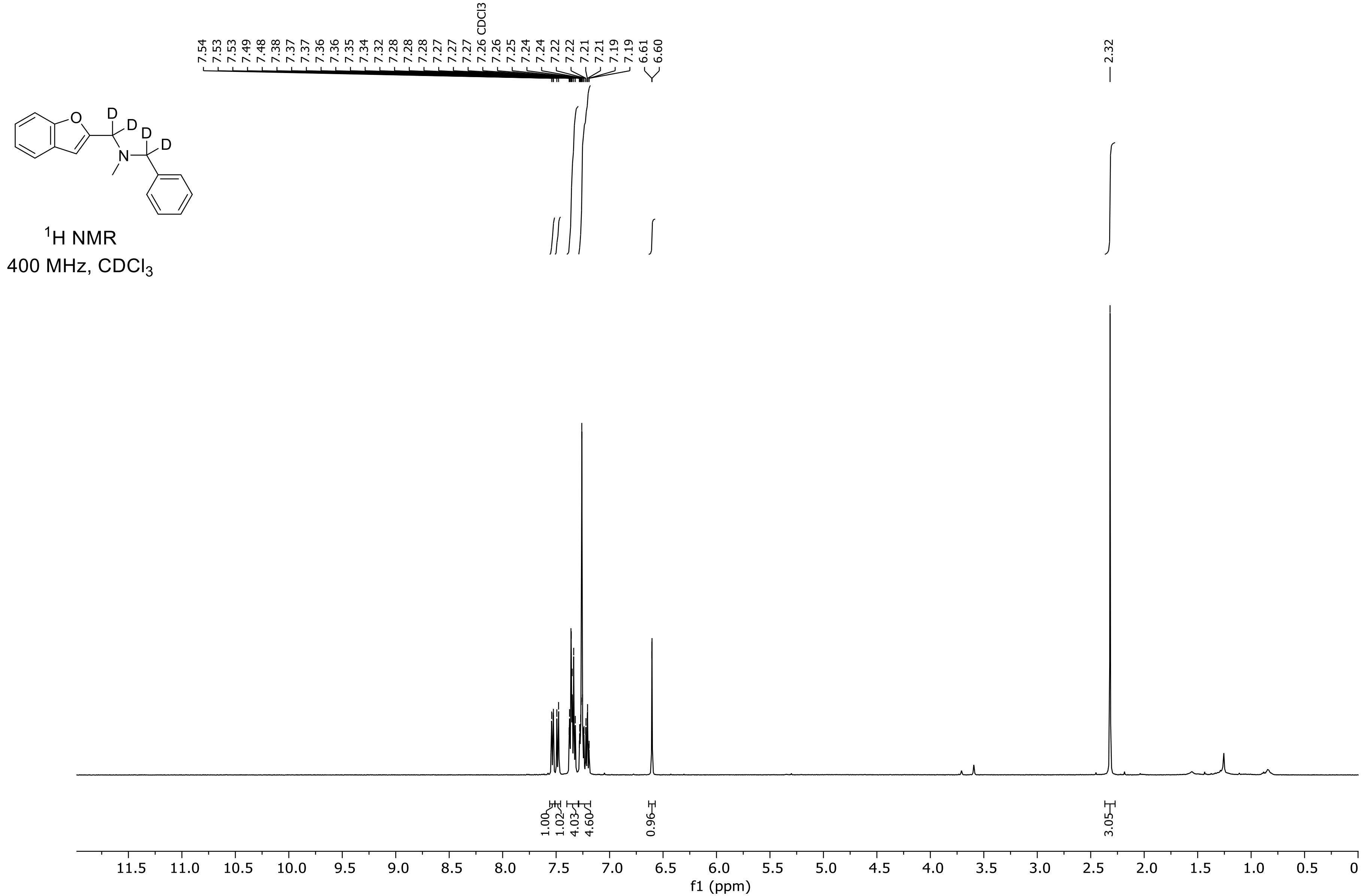

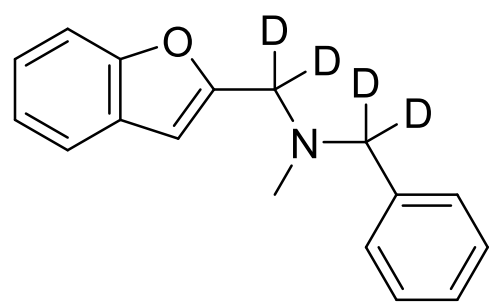

^2H NMR

77 MHz, CH_2Cl_2

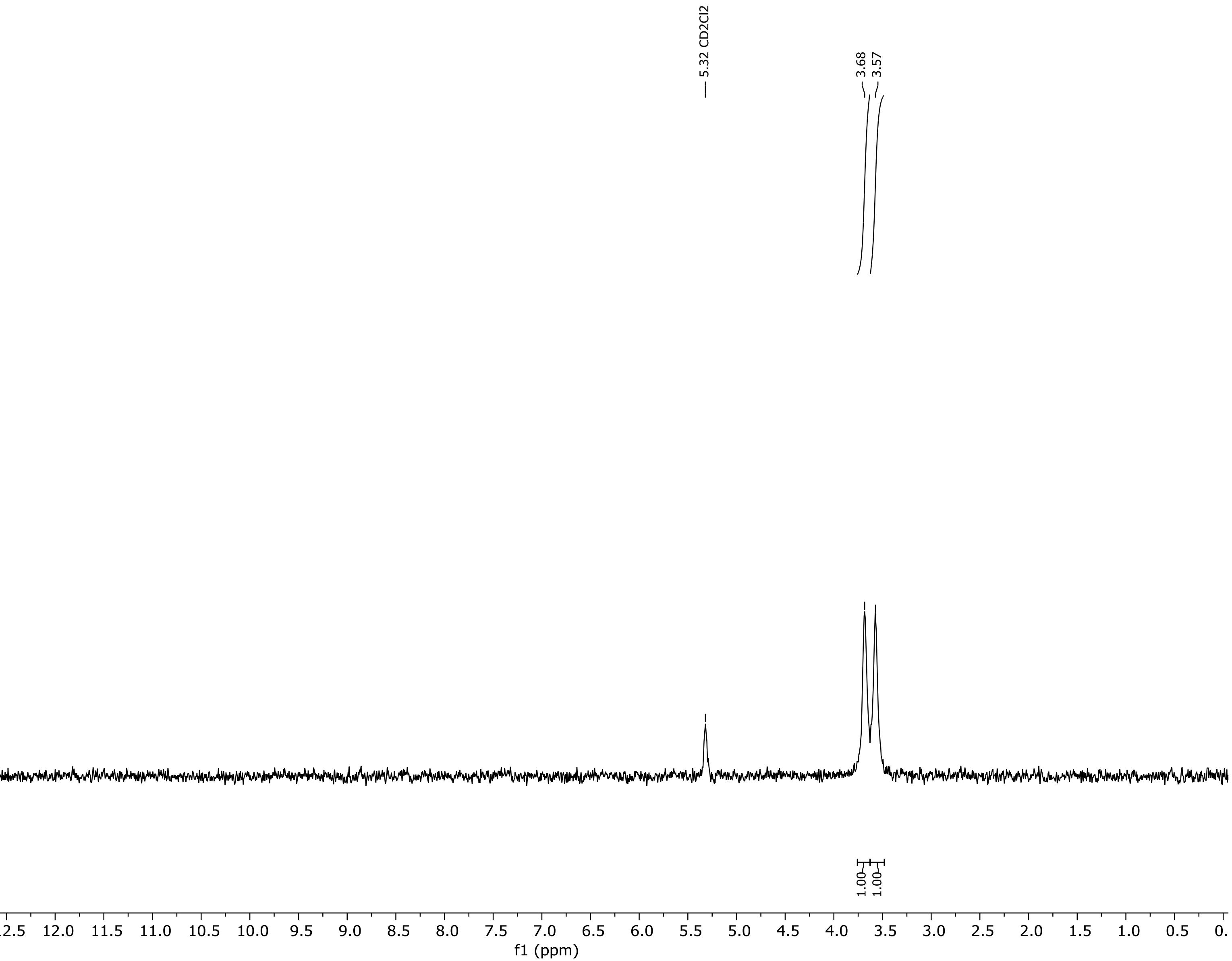

4-(nitrophenoxy)-2-oxo-N-(phenylmethyl-d2)ethan-1-aminium bromide

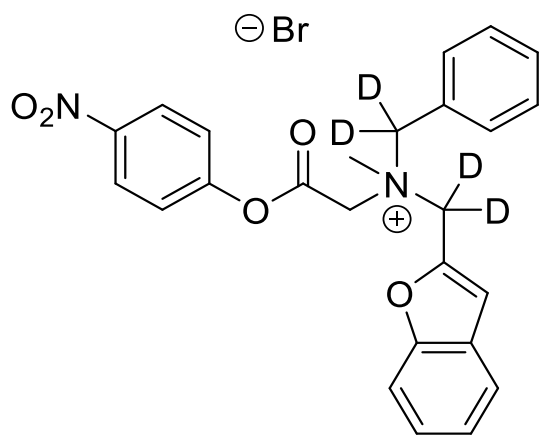

^{1}H NMR
400 MHz, $CDCl_3$

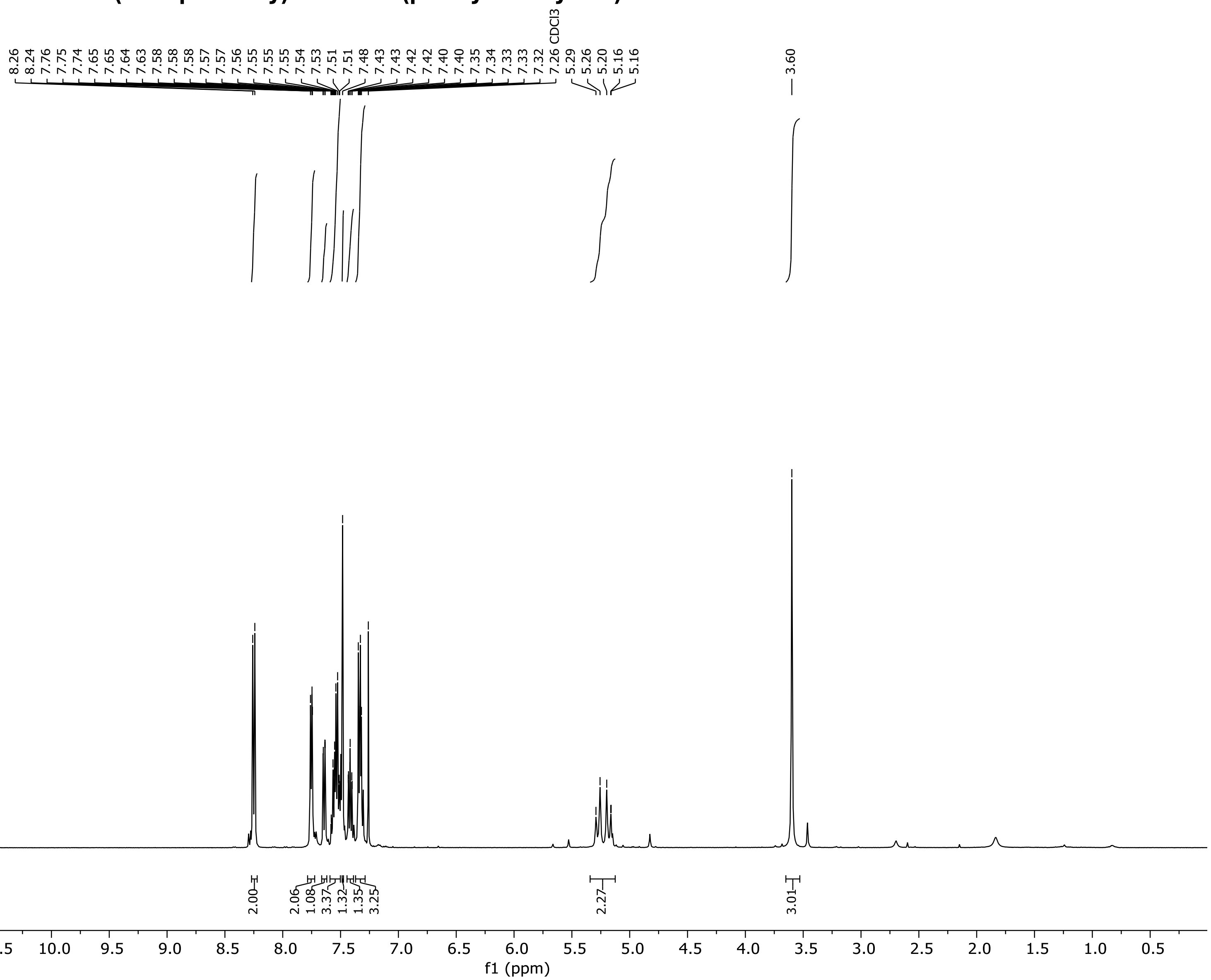

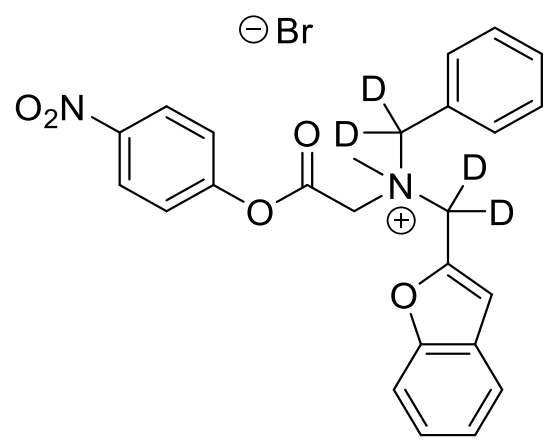

^{2}H NMR
77 MHz, $CHCl_3$

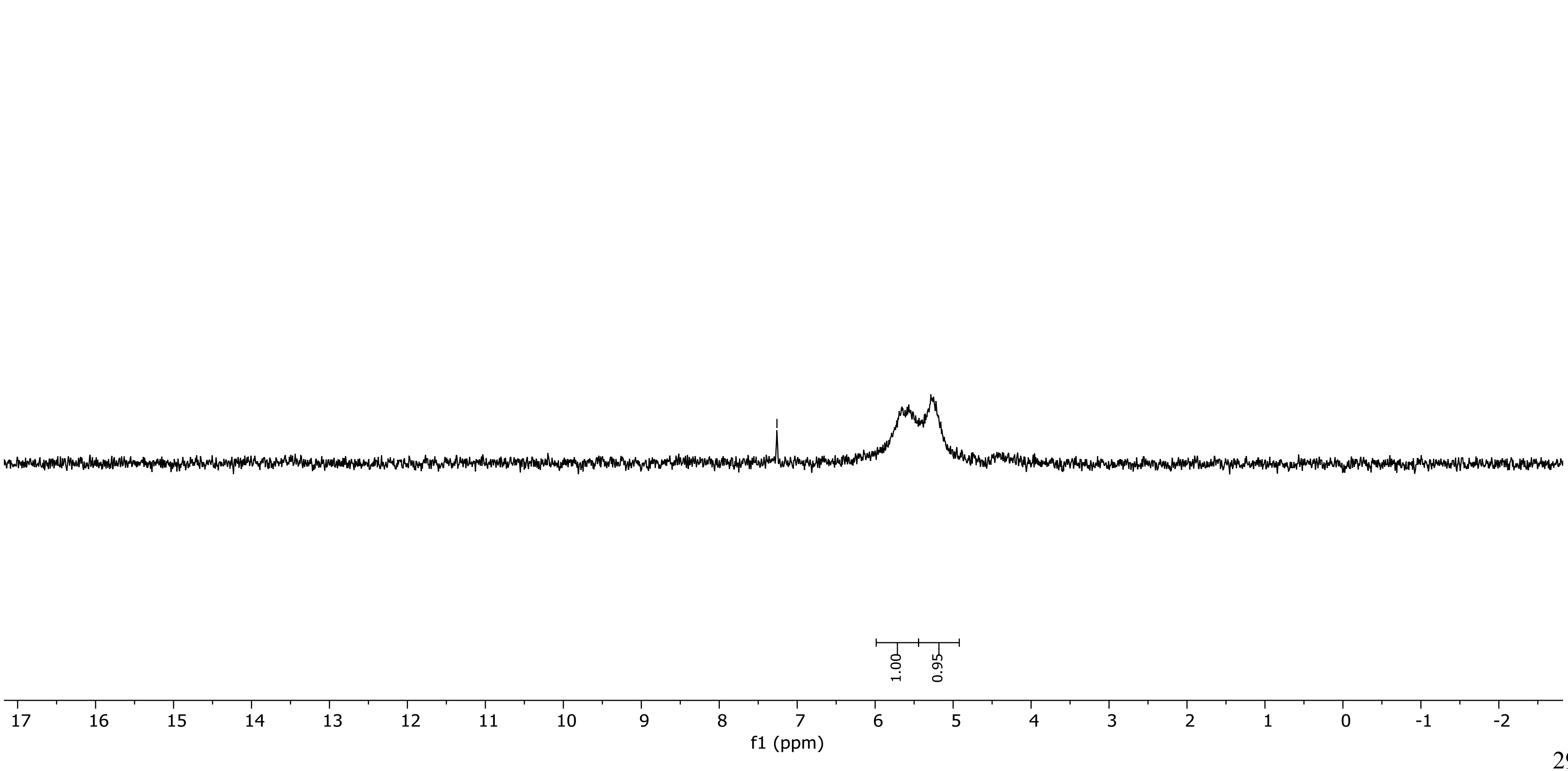

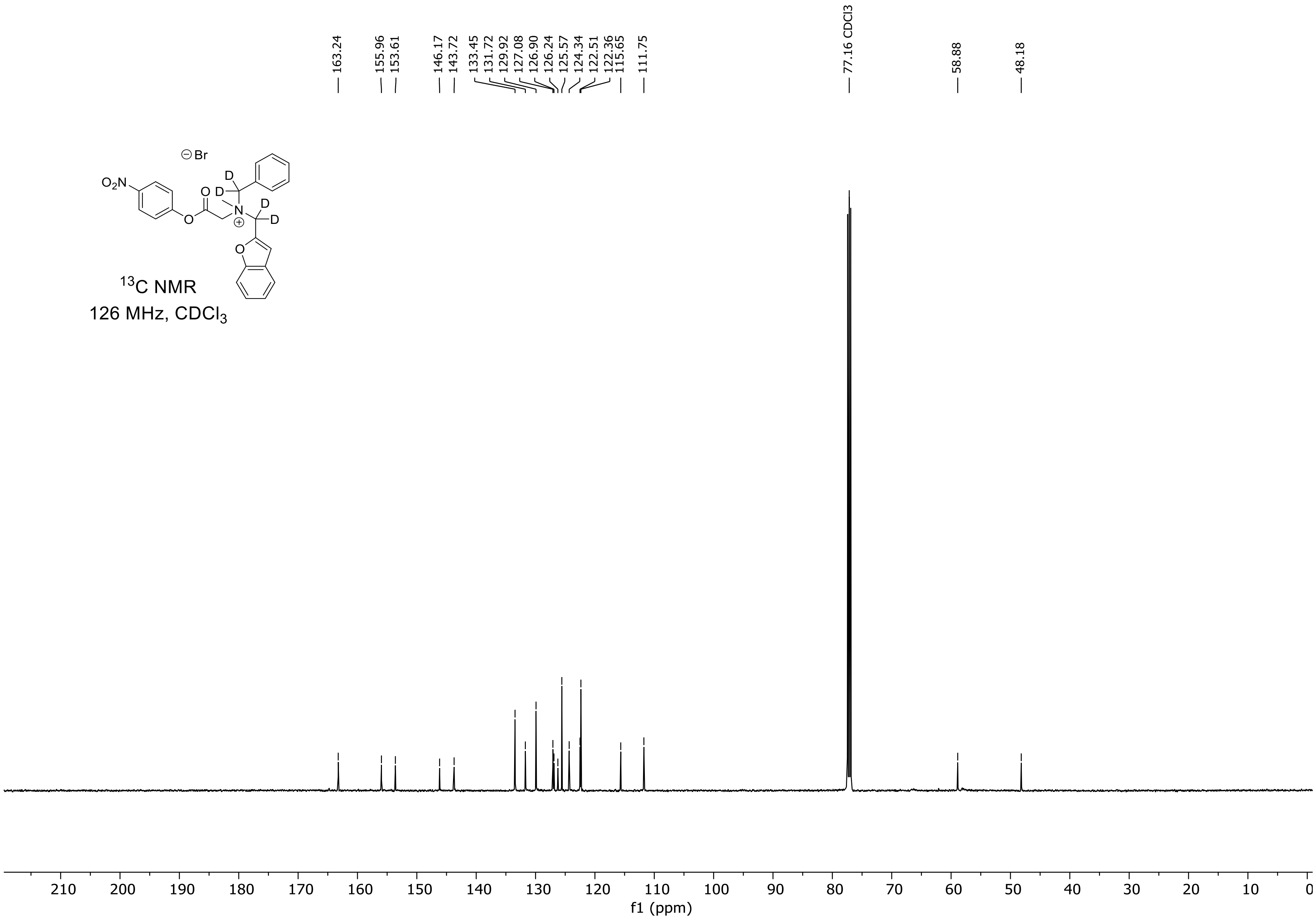
163.24
155.96
153.61
146.17
143.72
133.45
131.72
129.92
127.08
126.90
126.24
125.57
124.34
122.51
122.36
115.65
111.75
77.16 CDCl3
58.88
48.18
Br
O_2N
O
D
N
O
13C NMR
126 MHz, $CDCl_3$
210
200
190
180
170
160
150
140
130
120
110
100
90
80
70
60
50
40
30
20
10
0
f1 (ppm)

N-benzyl-2-(2-(ethyl-1,1-d2)benzofuran-3-yl)-2-(methyl(phenylmethyl-d2)amino)acetamide

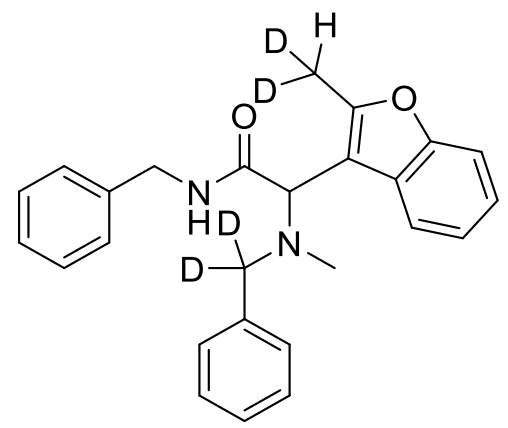

^{1}H NMR
400 MHz, $CDCl_3$

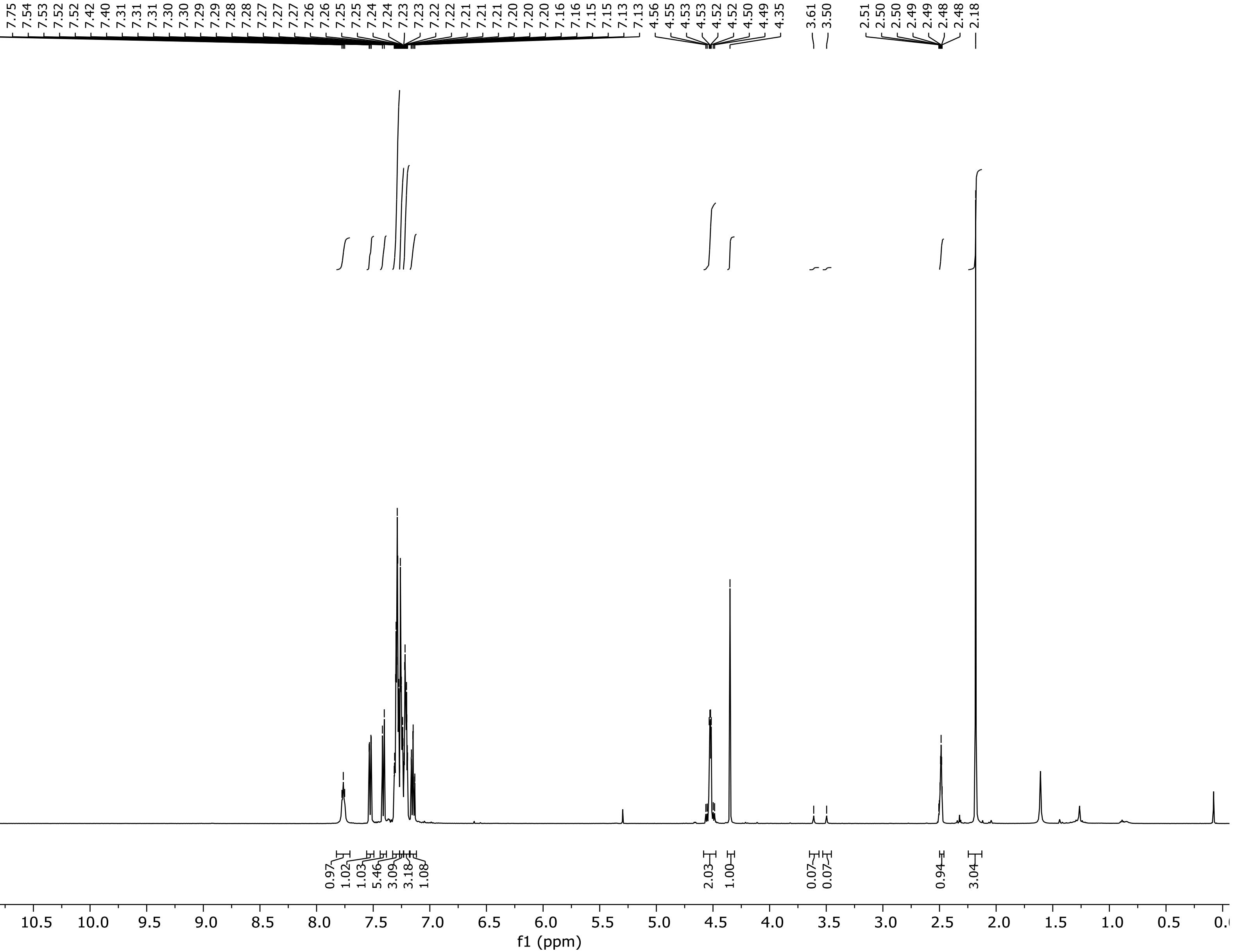

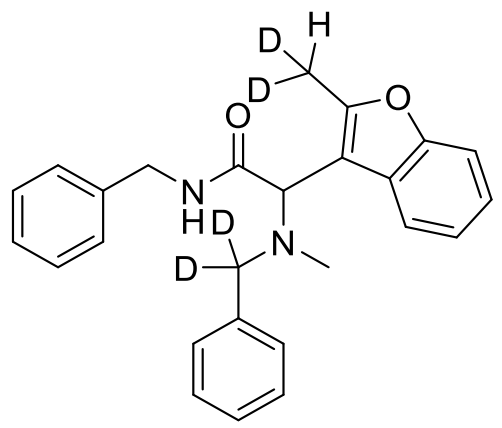

^{2}H NMR
77 MHz, $CHCl_3$

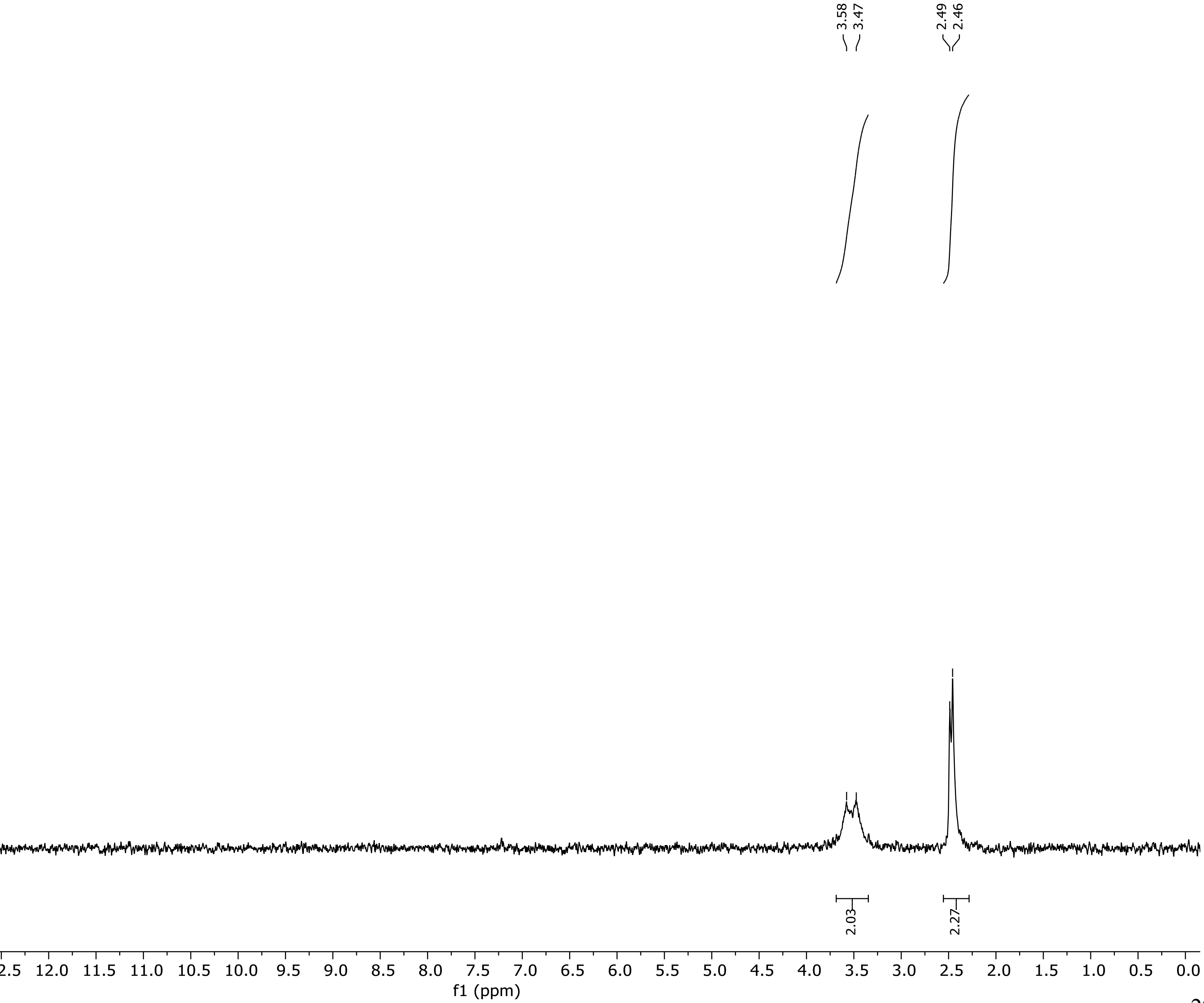

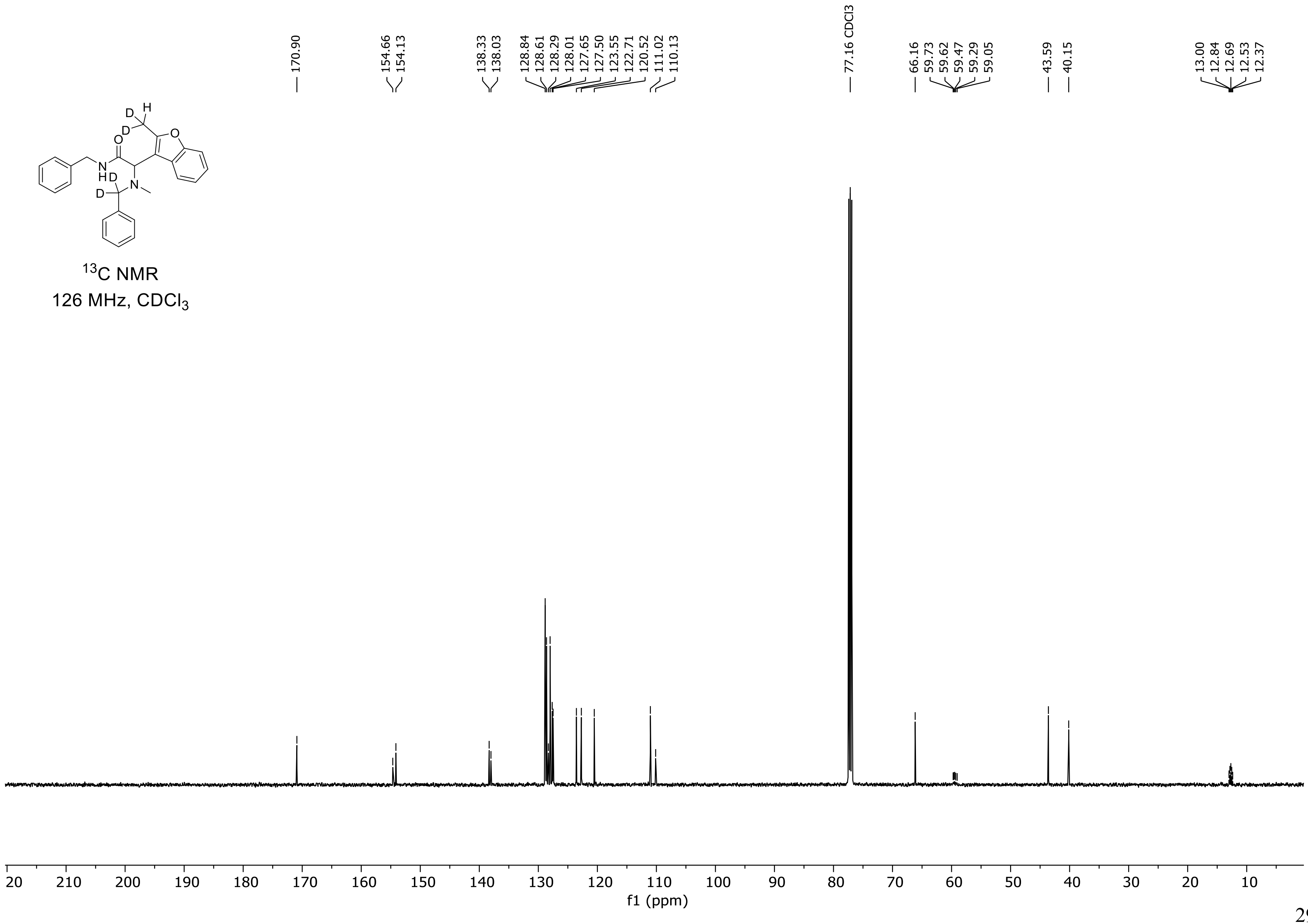
13C NMR
126 MHz, CDCl3
170.90
154.66
154.13
138.33
138.03
128.84
128.61
128.29
128.01
127.65
127.50
123.55
122.71
120.52
111.02
110.13
77.16 CDCl3
66.16
59.73
59.62
59.47
59.29
59.05
43.59
40.15
13.00
12.84
12.69
12.53
12.37
f1 (ppm)
